# Cooperative benzylic–oxyallylic stabilized cations: regioselective construction of α-quaternary centers in ketone-derived compounds†
†Electronic supplementary information (ESI) available: Experimental procedures and spectral data of new compounds. CCDC 1057455–1057460. For ESI and crystallographic data in CIF or other electronic format see DOI: 10.1039/c5sc01914a


**DOI:** 10.1039/c5sc01914a

**Published:** 2015-07-22

**Authors:** Nitin S. Dange, Jacob R. Stepherson, Caitlan E. Ayala, Frank R. Fronczek, Rendy Kartika

**Affiliations:** a Department of Chemistry , Louisiana State University , 232 Choppin Hall , Baton Rouge , LA 70810 , USA . Email: rkartika@lsu.edu

## Abstract

We describe a novel reactivity of benzylic–stabilized oxyallyl cations towards regioselective construction of carbon quaternary centers. These synthetically useful intermediates were readily generated upon ionization of aryl substituted α-hydroxy methylenol ethers with catalytic, mild Brønsted acid. The emerging unsymmetrical oxyallyl cations were then directly captured by indoles and other nucleophiles with exquisite control of regioselectivity, predictably at the electrophilic carbon bearing the alkyl substituent to produce highly functionalized, value-added enol ethers.

## Abstract 2

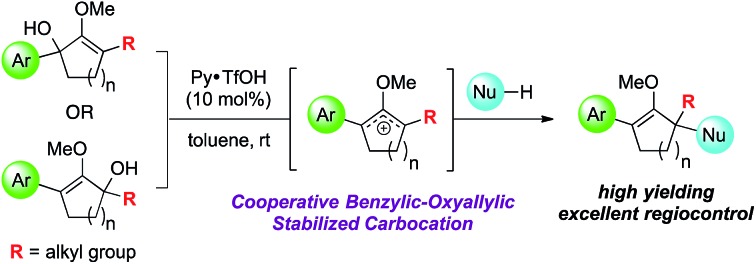
Herein we describe a direct capture of benzylic–oxyallylic stabilized carbocations with high value nucleophiles towards regioselective construction of α-quaternary centers.

## Introduction

**Scheme 1:**
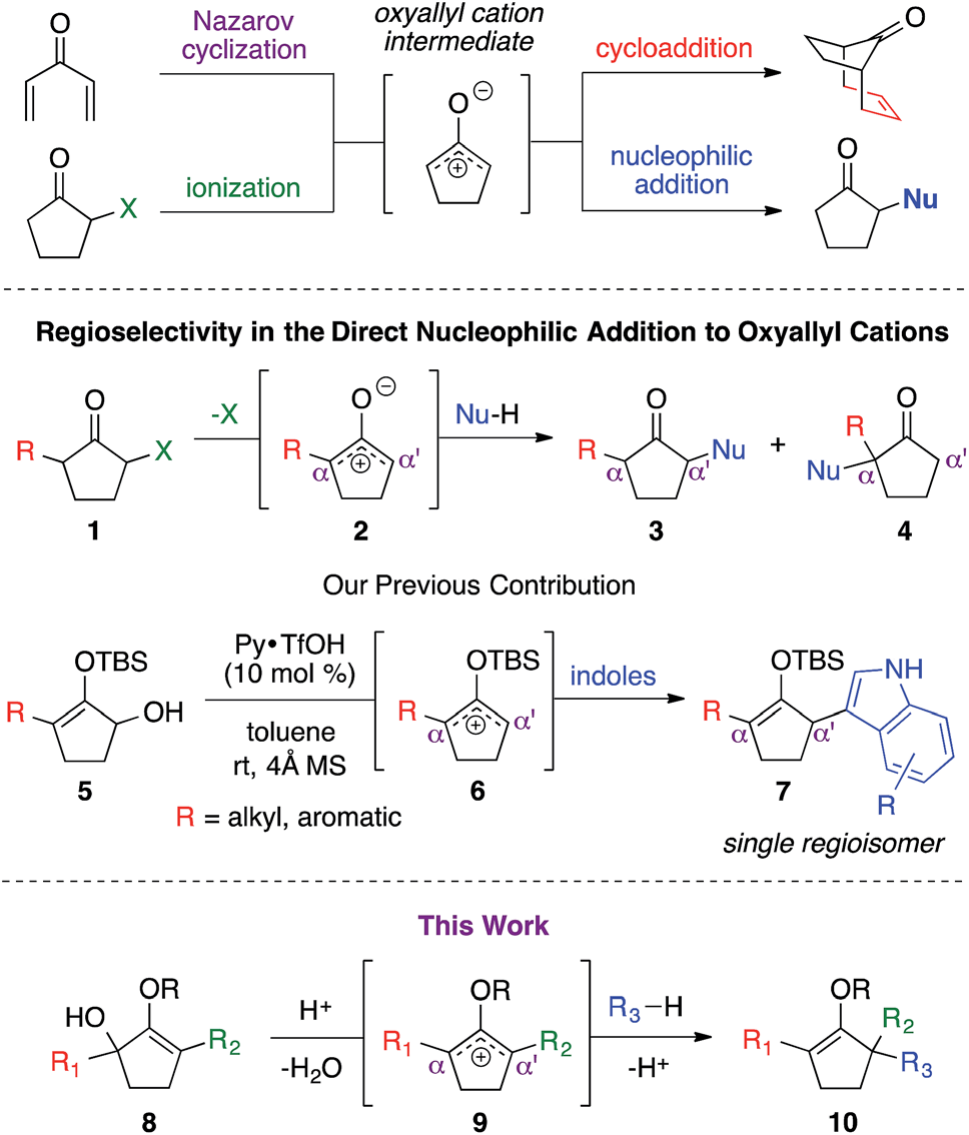
Direct nucleophilic addition to oxyallyl cation.

Oxyallyl cation is a reactive intermediate with profound roles in many synthetic applications. It exhibits unique reactivity due to the distribution of its electrophilic character over three carbon atoms. For example, this species is universally regarded as the participating intermediate in the Nazarov cyclization,^1^ involving electrocylic ring closure of divinyl ketone (Scheme 1), which could be intercepted *via* facile processes, such as [4+3] cycloaddition1b,^2^ and [3+2] cycloaddition,^3^ to expediently generate a variety of complex molecular architectures. Recently, studies on the generation of oxyallyl cations *via* acid or base-induced departure of a leaving group at the α-carbon of ketone-derived compounds, followed by immediate nucleophilic capture began to emerge in the literature.^4^ These new bond-forming processes successfully introduced various α-functionalities that otherwise would not have been feasible using classical strategies. While these seminal reports largely employed symmetrical systems, the use of unsymmetrical oxyallyl cations **2** appeared to be rather problematic, as addition of nucleophiles to this intermediate had been shown to occur competitively at both α and α′ positions, producing a mixture of regioisomers. Our group recently contributed a solution to this fundamental problem.^5^ We discovered that simple protection of oxyallyl cation **2** to the corresponding *O*-TBS variant **6** enabled a highly regioselective addition of indoles to furnish α,α′-disubstituted silylenol ethers **7**.

Driven by our interest in developing *de novo* synthesis of quaternary carbon centers, our unique tactic in controlling regioselectivity under exceedingly mild conditions encouraged us to investigate a direct nucleophilic addition to much more complex unsymmetrical α,α′-disubstituted oxyallyl cations, *viz.***9**. In fact, the success of this novel chemistry would introduce a powerful paradigm in the regioselective construction of an α-quaternary carbon center in ketone-derived compounds, which remain a significant synthetic challenge.^6^ While formation of quaternary centers *via* nucleophilic trapping of α,α′-disubstituted oxyallyl cations generated *via* Nazarov cyclization are precedented,^7^ there are only a very few examples on the use of unsymmetrical oxyallyl cations that proceeded in a regioselective fashion. Our hypothesis followed the assumption that ionization of disubstituted α-hydroxy enol ether **8** promoted by Brønsted acid in a non-polar medium should generate oxyallyl cation **9**. Assuming that the substituents in the α and α′ positions are electronically dissimilar, we proposed that the ensuing nucleophilic addition to this reactive intermediate should proceed with a distinct regioselective preference.

## Results and discussion

**Scheme 2:**
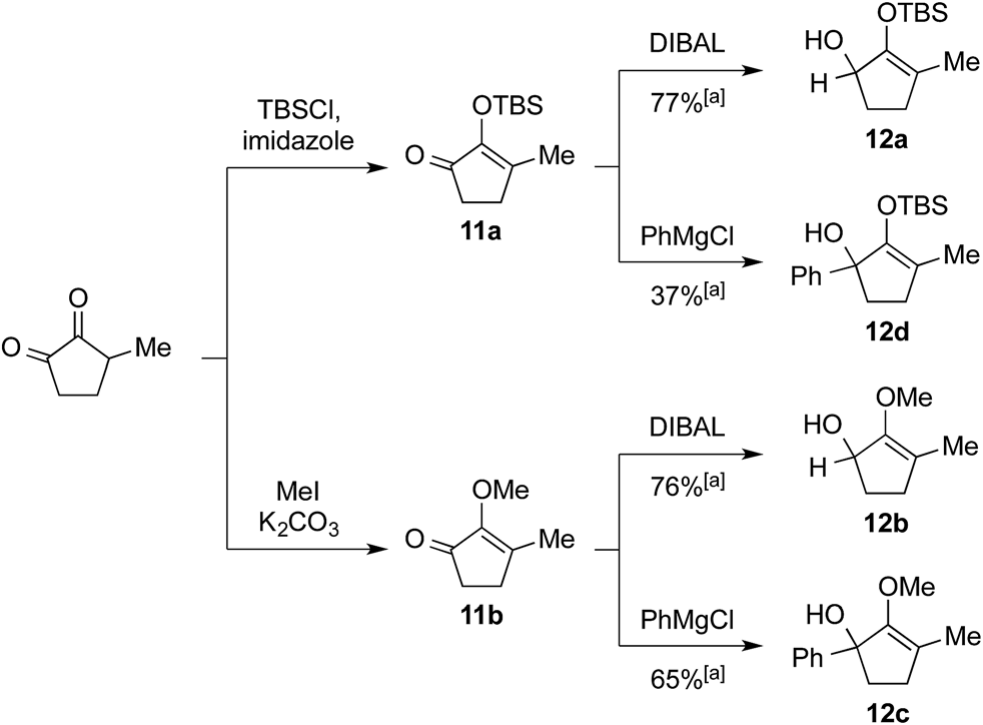
Synthesis of starting materials **12a–12d**. [a] Isolated yield over two steps after flash chromatography.

We commenced our investigation by rapidly assembling model substrates **12a–12d** in a divergent manner from 3-methyl-cyclopentane-1,2-dione (Scheme 2). This commercially available compound was subjected to an initial treatment with TBSCl and imidazole to give silylenol ether **11a**, or methyl iodide and potassium carbonate to yield the methoxy variant **11b**. The residual ketone functionality in these adducts was then either reduced with DIBAL to generate secondary alcohol **12a** and **12b**, or reacted with phenylmagnesium chloride to afford tertiary alcohol substrates **12c** and **12d**.

With the availability of these crucial starting materials, we then proceeded towards screening studies. As depicted in Table 1, entries 1 and 2, we began by deliberately increasing the catalyst loading to 50 mol% and the reaction concentration to 0.2 M from the previously established conditions to accelerate the reaction.^5^ We suspected that the TBS enol ether moiety impeded the rate of reaction, perhaps through destabilization of the emerging silyloxyallyl cation intermediate **15** by its electron-withdrawing character. To test this hypothesis, we subjected methylenol ether analogue **12b** to identical conditions, and activation of this electron rich substrate was indeed complete in just one hour. Surprisingly, the corresponding methylenol ether adducts were produced in an essentially 1 : 1 mixture of regioisomers, indicating that addition of indole to the presumed oxyallyl cation **16** was not selective.

**Table 1:** Screening studies

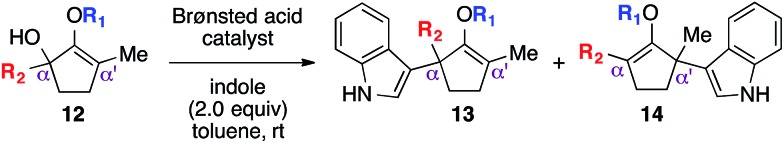									
Entry	Substrate	R_1_	R_2_	Catalyst	Loading (mol%)	Conc.^*a*^ (M)	Time (h)	Yield^*b*^	**13** : **14**^*c*^
1	**12a**	–TBS	–H	Py·TfOH	10	0.05	66	91%	99 : 1
2	**12a**	–TBS	–H	Py·TfOH	50	0.2	3	70%	99 : 1
3	**12b**	–Me	–H	Py·TfOH	50	0.2	1	71%^*d*^	56 : 44^*e*^
4	**12c**	–Me	–Ph	Py·TfOH	50	0.2	1	86%	1 : 99
5	**12c**	–Me	–Ph	Py·TfOH	10	0.2	2	81%	1 : 99
6	**12c**	–Me	–Ph	Py·TsOH	10	0.2	2	78%	1 : 99
7	**12c**	–Me	–Ph	CSA	10	0.2	2	40%	1 : 99
8	**12c**	–Me	–Ph	TfOH	10	0.2	1	14%	1 : 99
9	**12d**	–TBS	–Ph	Py·TfOH	10	0.2	110	38%^*f*^	1 : 99
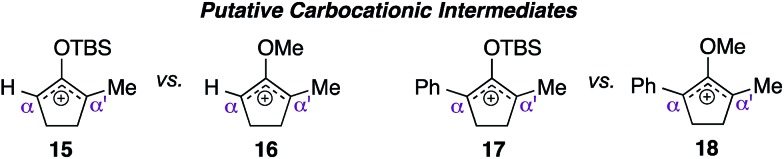									

^*a*^Reaction concentration was based on starting material **12**.

^*b*^Isolated yield of products **13** or **14** after flash chromatography.

^*c*^The ratio of regioisomers was determined by ^1^H NMR of the crude reaction mixture.

^*d*^Combined yield for both regioisomers as they were not separable by flash chromatography.

^*e*^The corresponding ketones were isolated upon aqueous workup.

^*f*^Starting material **12d** was never fully consumed.

Despite the complete loss of regioselectivity, we were enthused to observe a possible formation of quaternary center **14**. We subsequently incorporated an aromatic ring in the R_2_ position *via* starting material **12c**, believing that the α-phenyl and α′-methyl substituents would provide sufficient electronic bias to differentiate the two electrophilic centers in unsymmetrical oxyallyl cation **18** towards nucleophilic attack. Indeed, exposure of substrate **12c** to the activation conditions afforded quaternary center **14** in 86% yield, remarkably as a single regioisomer, where indole addition occurred exclusively at the α′-position (entry 4). Gratifyingly, reducing the catalyst loading back to 10 mol% essentially furnished the same product with an identical isolation yield and regioselectivity, albeit with a slightly longer but manageable reaction time (entry 5).

As indicated in entry 6, activation of starting material **12c** with 10 mol% pyridium tosylate produced the corresponding indole adduct **14** in an essentially identical yield from that obtained using catalytic pyridinium triflate. This result suggested that the counter anion perhaps merely served as a spectator in this methodology. Nevertheless, the quality of our reaction appeared to be directly dependent on the acidity of the catalysts. For instance, the use of stronger Brønsted acids,^8^ such as CSA (entry 7) and triflic acid (entry 8) led to significant decomposition of materials, thus leading to much lower isolation yields of the target product. These studies also demonstrated that free pyridine, liberated from pyridinium ion catalyst upon ionization, did not play a role in controlling regioselectivity,^5^ as addition of indole to starting material **12c**, catalyzed by either CSA or triflic acid, also produced the corresponding quaternary center **14** as a single regioisomer. To further signify the role of *O*-methyl group in accelerating the rate of reaction, we then exposed TBS enol ether variant **12d** to the optimal reaction conditions. This compound, which presumably proceeded to generate electron deficient silyloxyallyl cation **17**, severely lacked reactivity and in fact failed to reach completion even after 110 hours of reaction time (entry 9).

With these preliminary results in hand, our investigation continued with a scope of indoles towards α′-quaternarization of methylenol ether **19** (Table 2).^9^ Electron-donating substituents, such as methyl and methoxy groups, produced quaternary stereocenters **21b** and **21c** in 90% and 74% yields, respectively. *N*-Methyl indole was also a suitable nucleophile, which afforded product **21d** in 74% yield. Halogen substituents, such as 6-chloro and 5-bromo indoles, were found robust under the optimized reaction conditions, leading to formation of the corresponding methylenol ethers **21e** and **21f** in 75% and 85% yields, respectively. Electron-deficient methyl-5-carboxylate indole furnished quaternary center **21g** in 68% yield despite a longer reaction time. Structurally elaborate 5-methoxy-1*H*-benzo[*g*]indole was also found to produce the corresponding quaternary center **21h** in near quantitative yield.

**Table 2:** Scope of indoles^*a*^

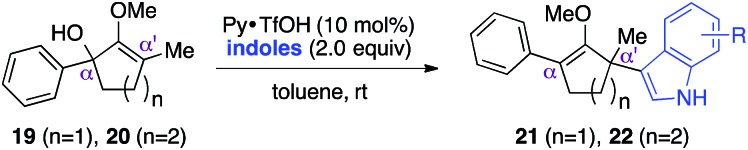
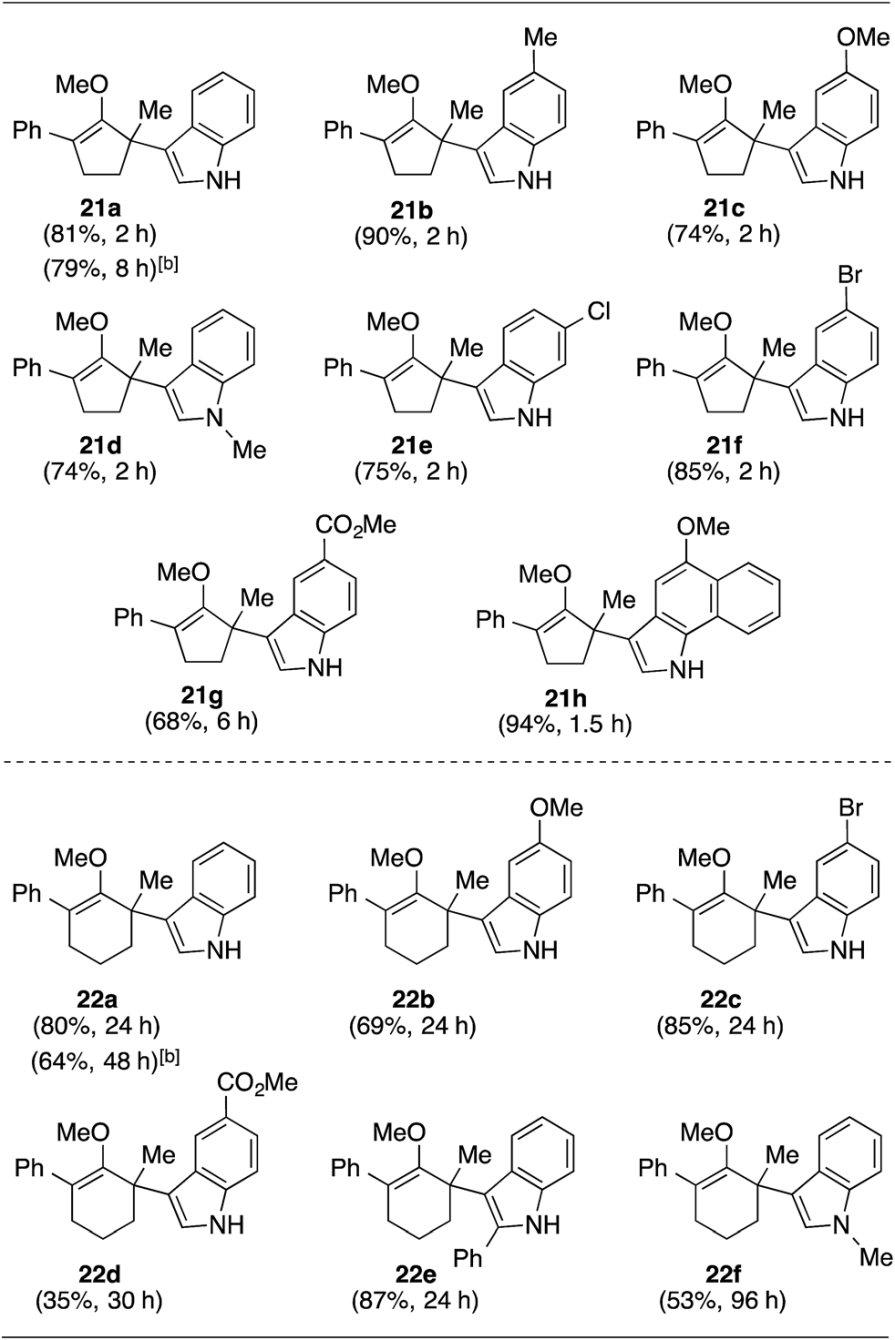

^*a*^Isolated yield of products **21** or **22** after flash chromatography.

^*b*^1.1 equivalents of indole was employed.

This synthetic method was also applicable to analogous 6-membered α-hydroxy methylenol ether **20**, which yielded the corresponding α′-quaternary center in methylenol ether **22** in high yields as a single regioisomer despite the longer reaction time.^10^ Functionalization of starting material **20** with indole cleanly produced α′-indoyl product **22a** in 80% yield.^9^ The use of electron donating 5-methoxyindole and halogenated 5-bromoindole furnished the corresponding methylenol ethers **22b** and **22c** in 69% and 85%. The structure of compound **21c** and **22b** was conclusively confirmed by X-ray analysis.^11^ Interestingly, electron deficient methylindole-5-carboxylate led to an isolation of the resulting product **22d** in modest 35% yield. We also explored the applicability of sterically congested 2-phenylindole and *N*-methyl indole, which led to an installation of α′-quaternary center in **22e** and **22f** in good yields.

The role of aromatic and alkyl substituents at the α and α′-positions, respectively, was then examined. As shown in Table 3, we proposed that ionization of either α-hydroxy methylenol ether **23** or α′-hydroxy methylenol ether **24** with pyridinium triflate should both generate methyloxyallyl cation **25**, which would be captured by indole, predictably at the α′-position, to give the corresponding product **26**. As illustrated in entries 1–6, various aromatic rings, such as electron-donating *p*-anisole **23a** and *p*-toluene **23b**, cleanly furnished α′-indoyl adducts **26a** and **26b** in 90% and 84% yields, respectively. Halogen substituents, such as 4-fluorophenyl **23c** and 4-chlorophenyl **23d**, were also tolerated to produce methylenol ethers **26c** and **26d** in excellent yields. An aromatic heterocycle, such as 3-methylthiophene **23e**, furnished quaternary center **26e** in near quantitative yield. A larger aromatic system, such as the 2-napthyl group in **23f**, was also found to be suitable in this chemistry.

**Table 3:** Scope of aromatic and alkyl substituents

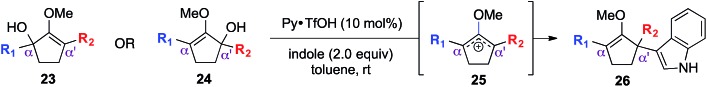			
Entry	Starting material	Product	Yield^*a*^
1	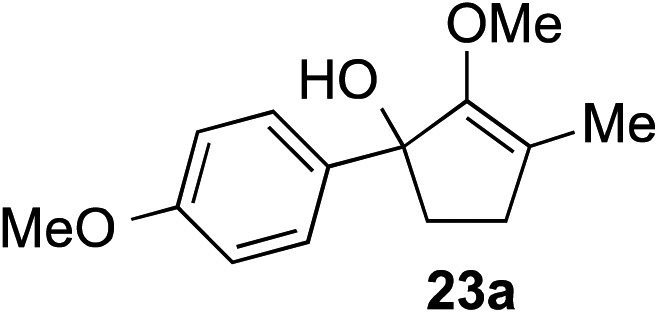	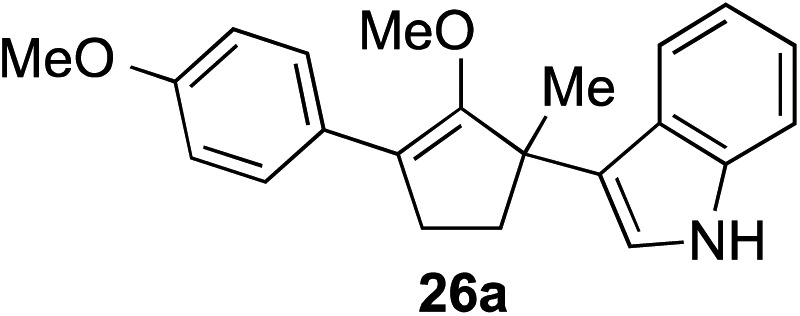	90% (1 h)
2	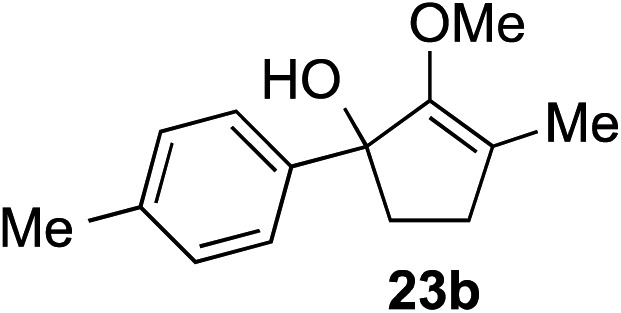	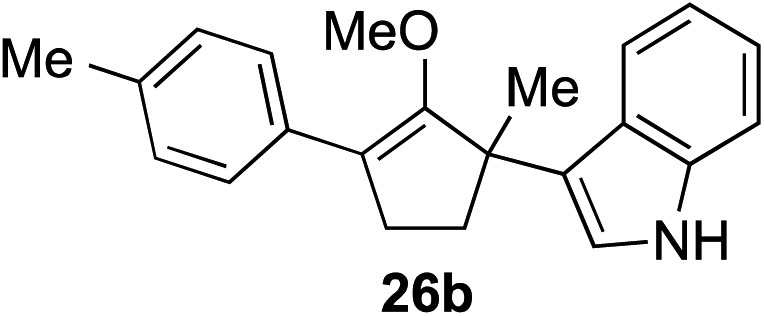	84% (1 h)
3	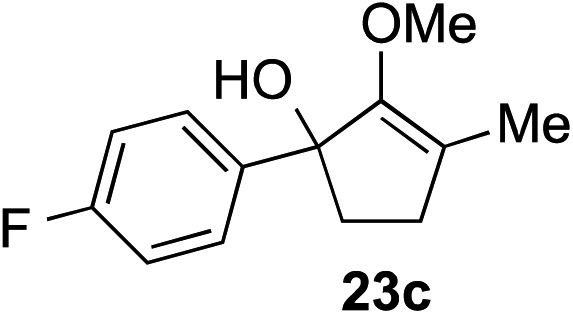	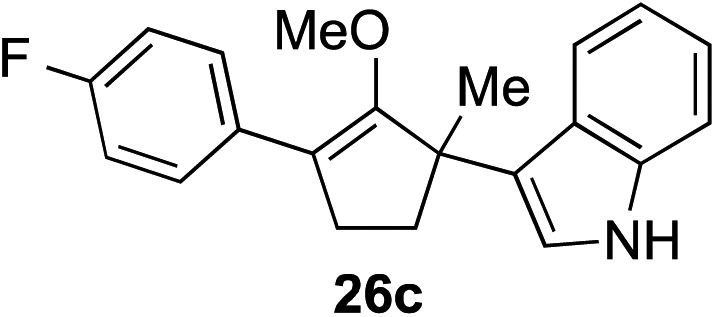	88% (1 h)
4	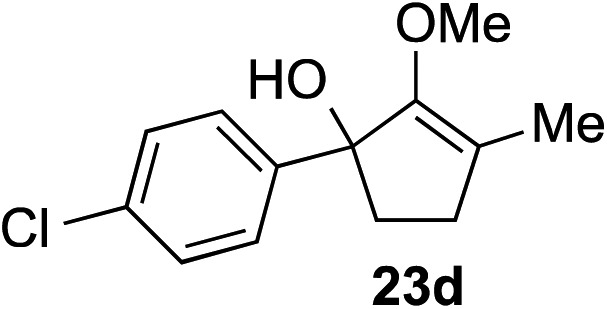	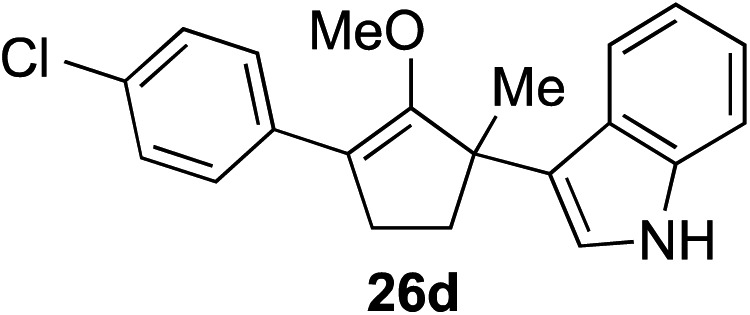	90% (1 h)
5	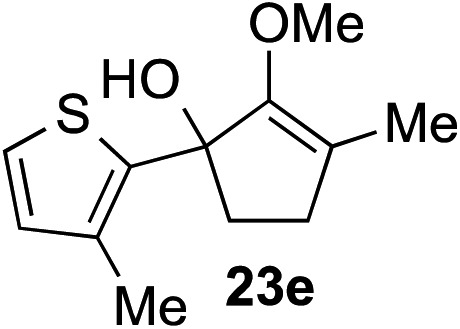	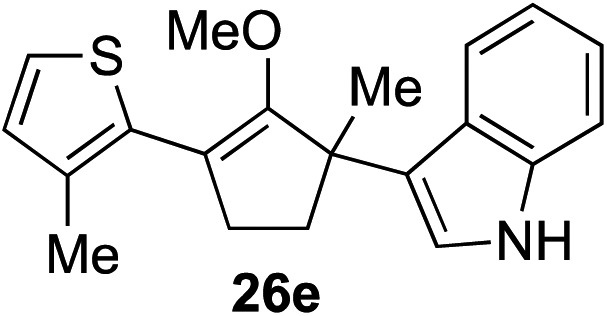	93% (1 h)
6	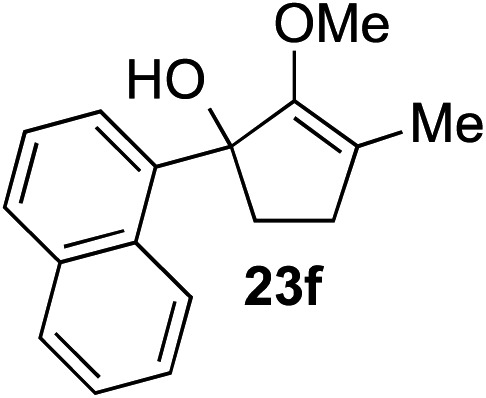	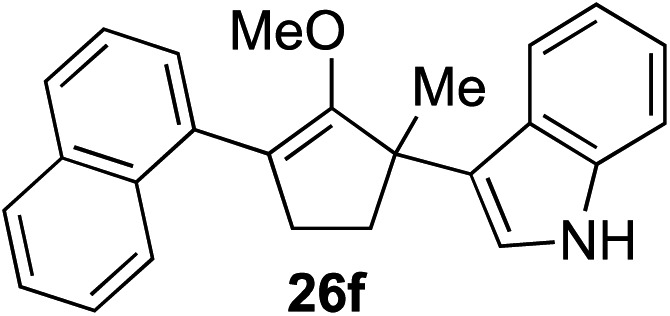	31%^*b*^ (1 h)
7	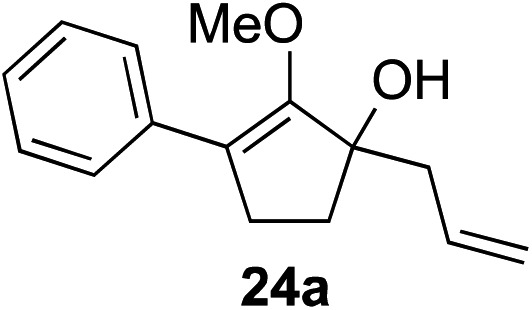	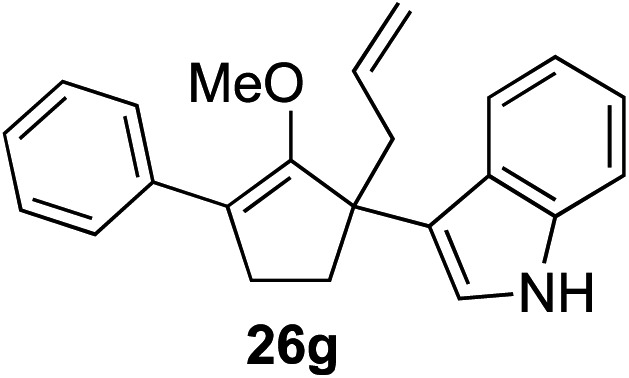	80% (1 h)
8	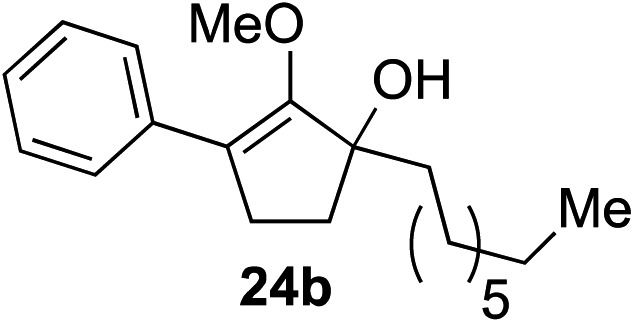	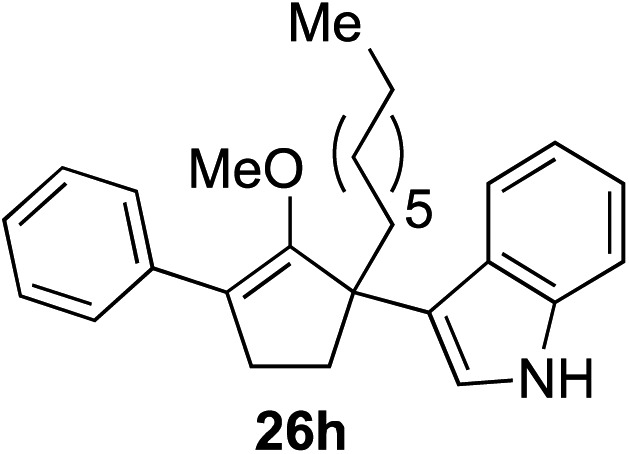	80% (18 h)
9	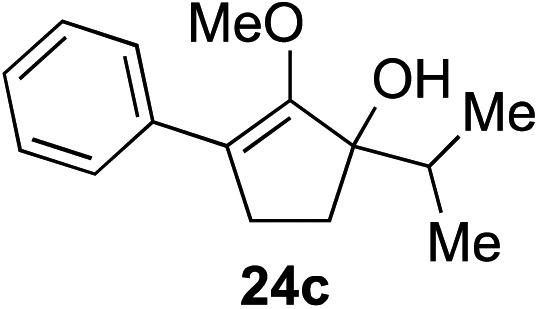	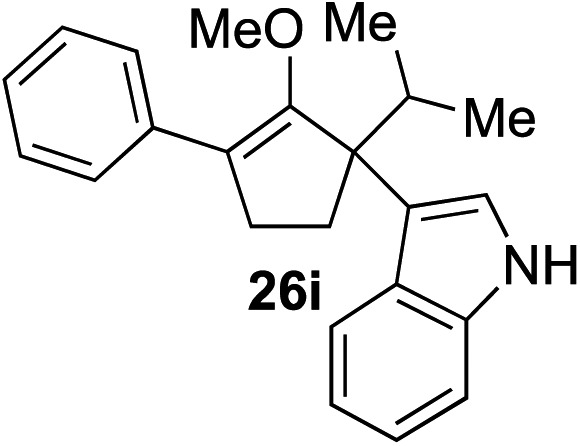	46%^*c*^ ^,^^*d*^ 3 : 2 rr^*e*^ (26 h)
10	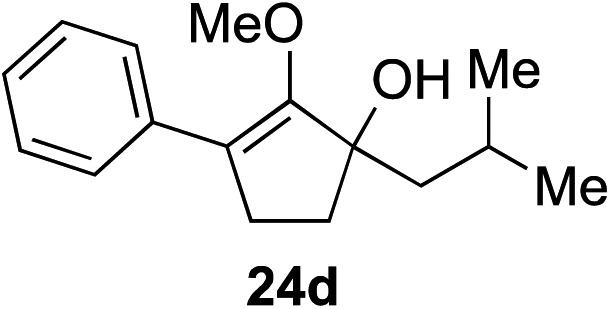	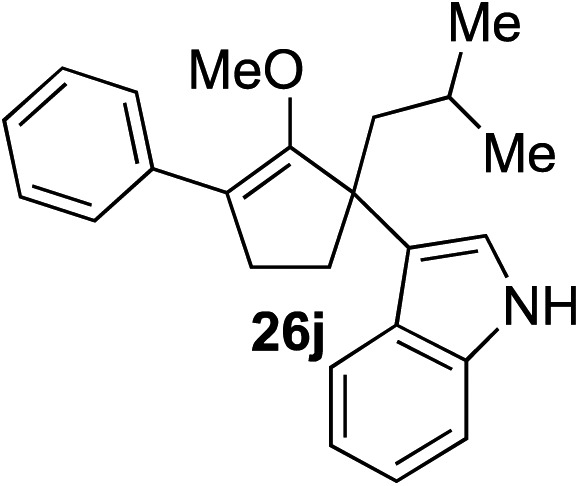	65%^*c*^ 1 : 1 rr^*e*^ (22 h)
11	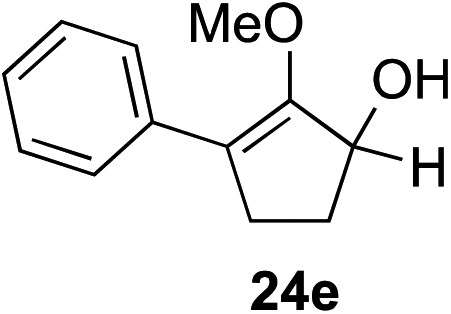	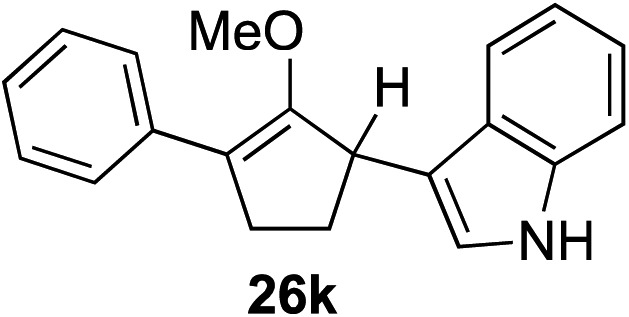	88% (1 h)
12	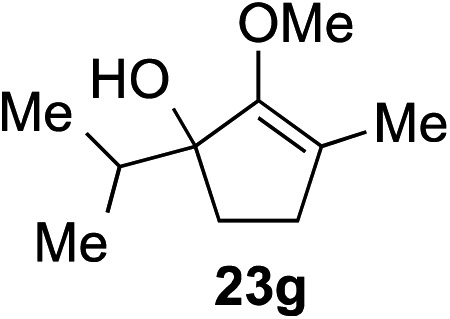	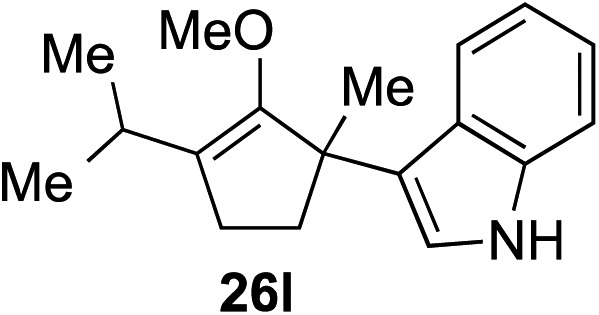	80%^*c*^ 3 : 1 rr^*e*^ (1 h)
13	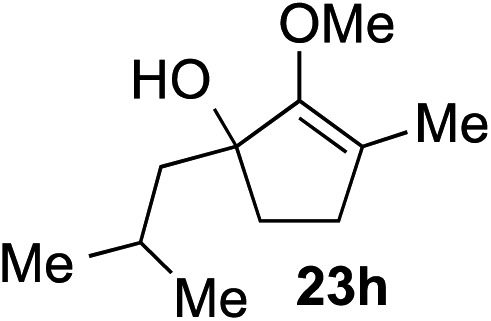	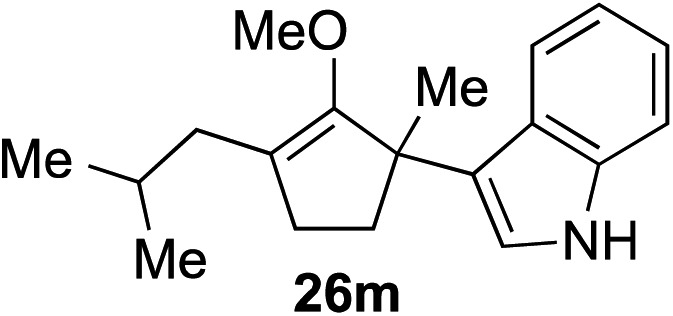	78%^*c*^ 4 : 1 rr^*e*^ (1 h)

^*a*^Isolated yield of products **26** after flash chromatography.

^*b*^The low yield was attributed to poor solubility of the product in most organic solvents.

^*c*^Combined yield for both regioisomers.

^*d*^The major regioisomer was not assigned, as these compounds were not separable by chromatography.

^*e*^The ratio of regioisomers was determined by ^1^H NMR of the crude reaction mixture.

Our investigation continued on with efforts to identify the effect of various α′-alkyl substituents in this enol ether functionalization reaction. As shown in Table 3, entries 7 and 8, allylic and aliphatic side chains **24a** and **24b** gave the corresponding α′-indoyl enol ethers **26g** and **26h**, respectively, in excellent yields as a single regioisomer. The regioselectivity induction in this methodology appeared to be sensitive to simple modulation in the steric effect. For instance, a significant erosion in regioselectivity was observed when sterically encumbered isopropyl or isobutyl groups were incorporated to the α′-position in starting materials **24c** and **24d**. The longer reaction time involving substrates **24b–24d** suggested that an increasing size of the alkyl substituents substantially impeded the rate of reaction.

As opposed to the methyl variant **12b** (Scheme 1, entry 3), α-phenyl substituted starting material **24e** exclusively produced α-phenyl-α′-indole **26k** as a single regioisomer in quantitative yield. This astounding result strongly suggests that the unusual benzylic-oxyallylic carbocation stabilization appeared to have cooperatively directed in these direct nucleophilic addition reactions.^12^ The involvement of the aromatic substituents in facilitating regioselectivity control was further confirmed by the use of starting materials bearing α-isopropyl **23g** and α-isobutyl **23h** (entries 12 and 13). Exposure of these substrates to the reaction conditions produced the corresponding methylenol ether adducts **26l** and **26m** with the indole addition occurring at the less sterically congested methylated α′-carbon. The observed regioselectivity in these products, however, was rather modest, suggesting that while steric influence readily offered some degrees of regiochemical induction, these effects alone were not sufficient in furnishing exclusive regioselectivity in the addition of nucleophiles to the putative oxyallyl cation intermediates without reinforcement from the benzylic-type stabilization provided by aryl substituents.

While we and others have extensively utilized substituted indoles in studies concerning a direct nucleophilic addition to oxyallyl cations,4f,4g,^5^ there are only a few precedents on the utility of other carbon and heteroatom nucleophiles in this type of chemistry.4g,4h As a guideline for us to judiciously select other potential carbon nucleophiles beyond indole, we consulted Mayr's nucleophilicity parameters (*N* values)^13^ and extracted the *N* values of various substituted indoles, which ranged from *N* = 2.2 for electron poor indoles to *N* = 7.2 for electron rich indoles,13*d* as a reference. As illustrated in Table 4, this strategy enabled us to identify that pyrrole (*N* = 4.6) successfully reacted with starting materials **19** and **20** to afford the corresponding methylenol ethers **27a** and **28a** in 45% and 89% yields, respectively, although these reactions were rather sluggish.13*e* In contrast, the use of much more nucleophilic 2,4-dimethylpyrrole (*N* = 10.7) rapidly converted five-membered methylenol ether **19** to the corresponding quaternary center **27b** in 83% yield within 30 minutes.13*f* Azulene (*N* = 6.7), a highly nucleophilic neutral aromatic hydrocarbon, was found to react in this method and yielded methylenol ethers **27c** and **28c** as a single regioisomer.13*h* We were also able to demonstrate the utility of 2-(trimethylsiloxy)furan (*N* = 7.2) in this methodology.13*c* This nucleophile readily functionalized starting material **19** to furnish stereochemically congested adduct **27d** in 62% yield, surprisingly as a single diastereomer.^11^

**Table 4:** Scope of nucleophiles

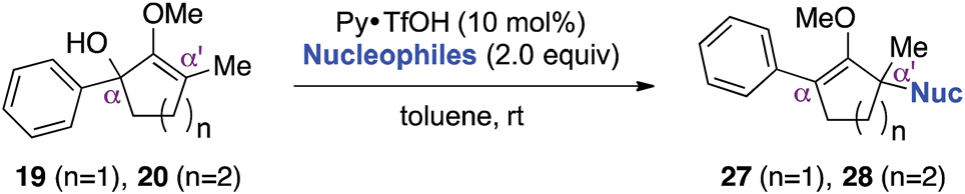			
Entry	Nucleophile	Product	Yield^*a*^
1	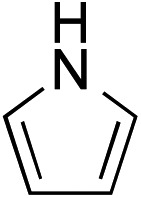	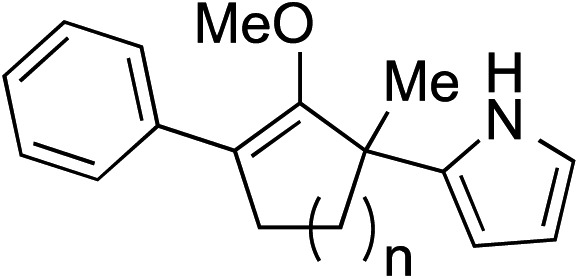	**27a** 45% (1.5 h), **28a** 89%^*b*^ (18 h)
2	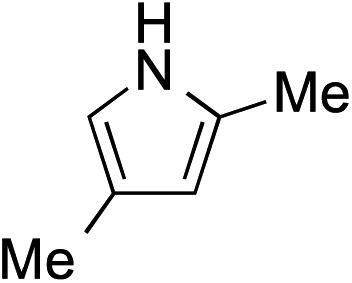	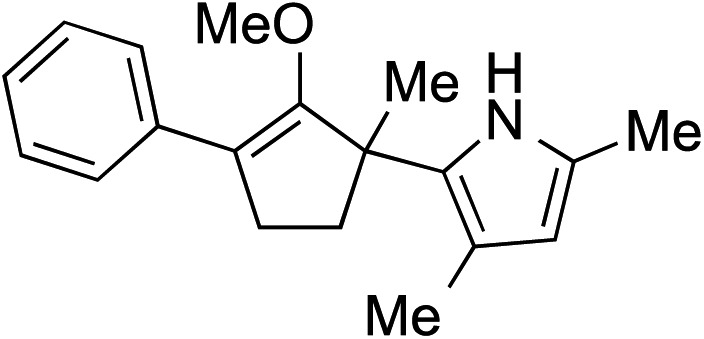	**27b** 83% (0.5 h)
3	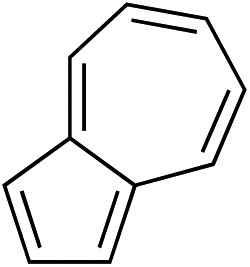	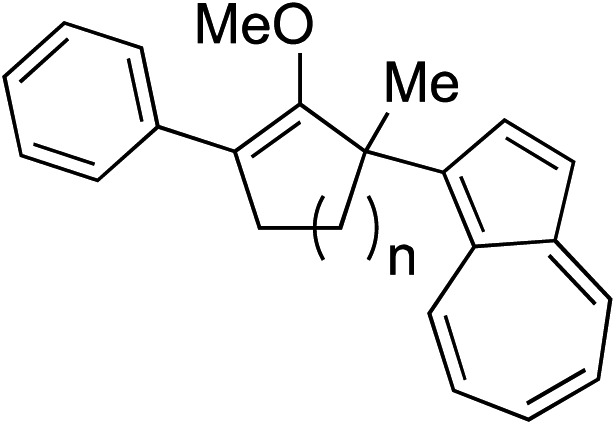	**27c** 62%^*c*^, (0.5 h), **28c** 39%^*b*^ ^,^^*c*^ ^,^^*d*^ (39 h)
4	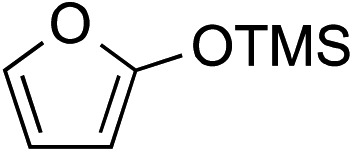	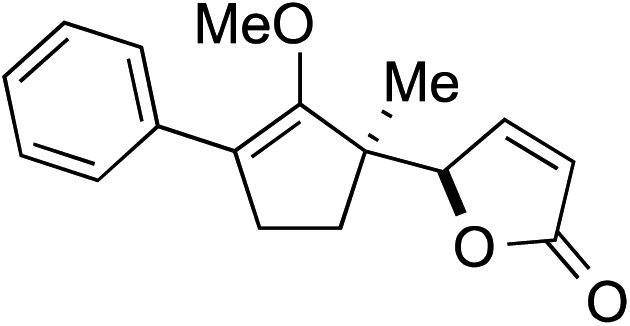	**27d** 62%, 20 : 1 dr (72 h)
5	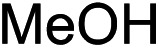	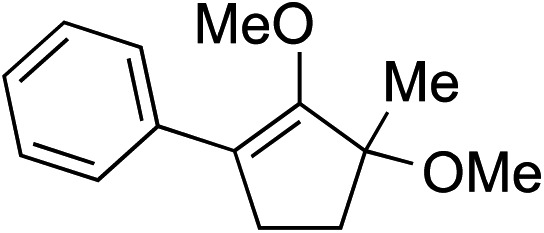	**27e** 69%^*e*^ (23 h)
6	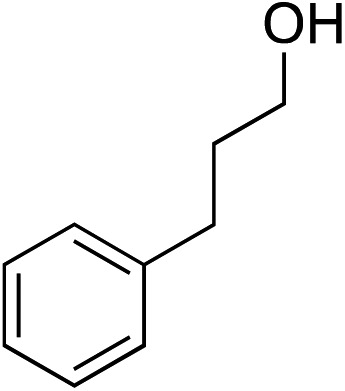	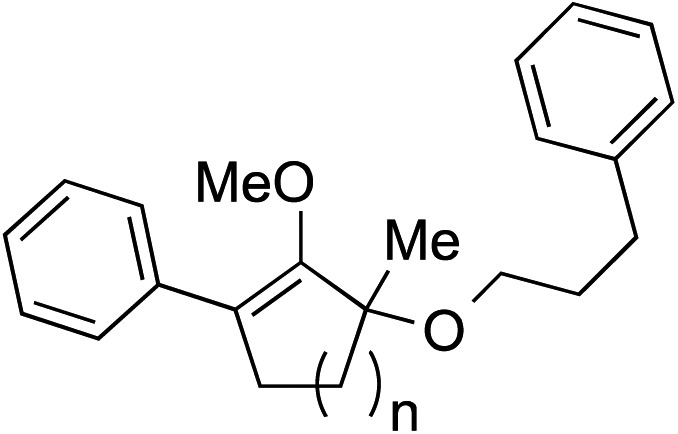	**27f** 39% (1 h), **28f** 94% (4 h)
7	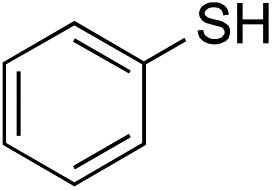	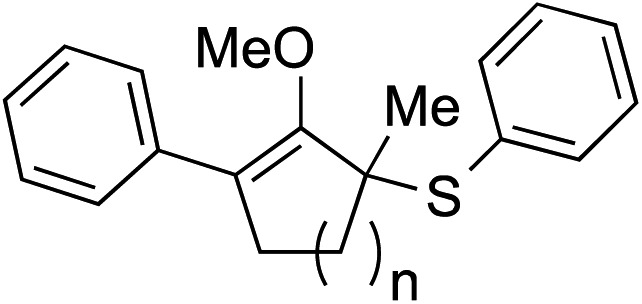	**27g** 78% (1.5 h), **28g** 93% (20 h)

^*a*^Isolated yield of products **27** and **28** after flash chromatography.

^*b*^50 mol% of pyridinium triflate was added.

^*c*^1.1 equivalent of azulene was employed.

^*d*^The starting material was not fully consumed.

^*e*^4 Å molecular sieves were added.

We recognized the challenge of using heteroatom-centered nucleophiles to capture carbocations in Brønsted acid catalyzed reactions due to the potentially reversible ionization of the newly generated carbon-heteroatom bonds promoted by the catalyst.^12^,^14^ We explored the applicability of such nucleophiles by initially focusing on alcohols.^15^ Interestingly, a reaction between α-hydroxy enol ether **19** and methanol produced the corresponding methanol adduct **27e** in 69% yield as a single regioisomer. While the use of 3-phenyl-1-propanol afforded methylenol ether **27f** in modest 39% yield, the addition of this primary alcohol to six-membered substrate **20** formed quaternary center **28f** in near quantitative yield. An attempt to incorporate thiophenol was also successful to produce the respective mercaptan adducts **27g** and **28g** in high yields as a single regioisomer.

**Scheme 3:**
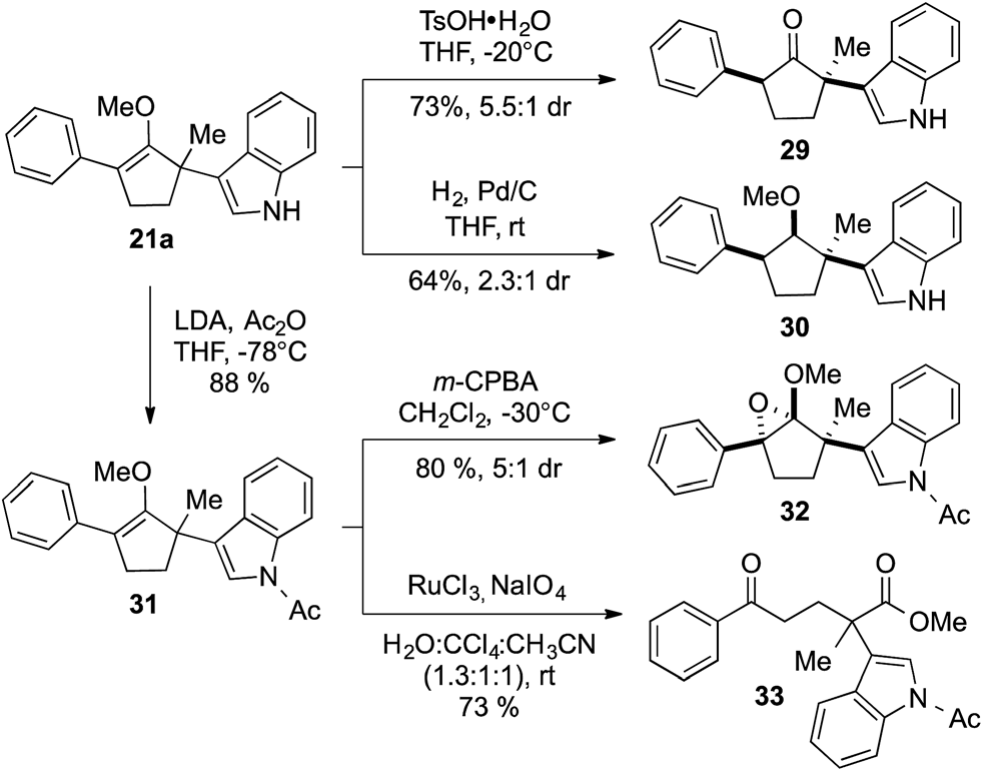
Synthetic applications of methylenol ethers.

Construction of these highly functionalized enol ethers provided a unique venue to access various structurally formidable quaternary center-containing molecular architectures (Scheme 3). For example, treatment of methylenol ether **21a** to stoichiometric toluenesulfonic acid monohydrate in THF at –20 °C readily produced the corresponding stereochemically elaborate ketone **29** in 73% yield with 5.5 : 1 diastereoselection. Our ability to install two aromatic substituents, each at the α- and α′-positions of ketone, while simultaneously introducing an α-quaternary center, clearly demonstrated the strength of our methodology. Methylenol ether **21a** could also be subjected to palladium-catalyzed hydrogenation. These conditions introduced a stereotriad in methylated cyclopentanol **30** in a good yield with a decent control of diastereoselectivity.

The indole ring in compound **21a** could also be easily protected without compromising the methylenol ether functionality *via* treatment with LDA and acetic anhydride. The resulting acetate-protected product **31** could be further subjected to another diastereoselective transformation, such as oxidation of the carbon–carbon double bond using *m*-CPBA.^16^ Interestingly, this reaction cleanly produced structurally intricate epoxide **32** with three contiguous quaternary centers, in 80% yield as a 5 : 1 mixture of diastereomers. Similarly, the stereochemical identity of compounds **29**, **30**, and **32** was confirmed by single crystal X-ray diffraction.^11^ We were also able to oxidatively cleave the carbon–carbon double bond in methylenol ether **31** using RuCl_3_/NaIO_4_ to furnish 1,5-ketoester **33**.^17^ In addition to the generation an sp^3^–sp^2^ connectivity, chemoselective α-quaternarization of ester in the presence ketone are challenging due to the subtle acidity differences between the relevant α-hydrogens.^8^

## Conclusions

In conclusion, we detailed a new method to generate benzylic–oxyallylic stabilized carbocations under mild Brønsted acid catalysis and discovered that the reactivity of these novel intermediates could be harnessed towards a regioselective construction of quaternary centers through a direct capture with indoles and other high value nucleophiles. Overall, this chemistry efficiently furnished highly functionalized enol ethers that could be conveniently derivatized to other complex molecular architectures. Detailed mechanistic investigations on the origin of this unprecedented control of regioselectivity are ongoing in our laboratory, and the results will be reported in due course.

## Supplementary Material

Supplementary information

Crystal structure data
