# Supplementary material for: Cooperative benzylic–oxyallylic stabilized cations: regioselective construction of α-quaternary centers in ketone-derived compounds
Source: Chem Sci. 2015 Jul 22;6(11):6312–9. doi: 10.1039/c5sc01914a (PMC6054113; doi:10.1039/c5sc01914a)
Supplement: Supplementary file 1 [file SC-006-C5SC01914A-s001.pdf]

Electronic Supplementary Material (ESI) for Chemical Science.
This journal is © The Royal Society of Chemistry 2015

Cooperative Benzylic-Oxyallylic Stabilized Cations: Regioselective Construction of α-Quaternary Centers in Ketone-Derived Compounds

Nitin S. Dange, Jacob R. Stepherson, Caitlan E. Ayala, Frank R. Fronczek, and Rendy Kartika*

Department of Chemistry

232 Choppin Hall

Louisiana State University

Baton Rouge, LA 70803, United States

SUPPORTING INFORMATION

1. General Information …………………………………………………………….…. S-2
2. Characterization of New Compounds…..…………………………………………...…… S-3
3. X-Ray Crystal Data ………………………………………………………………….S-57
4. ^{1}H and ^{13}C NMR Spectra ………………………………………….………...… S-107

GENERAL INFORMATION

Unless otherwise noted, all materials were used as received from commercial suppliers without further purification. All anhydrous reactions were performed using oven-dried or flame-dried glassware, which was then cooled under vacuum and purged with nitrogen gas. Tetrahydrofuran (THF), dichloromethane (CH_2Cl_2), acetonitrile, toluene, and diethyl ether (Et_2O) were filtered through activated 3Å molecular sieves under nitrogen contained in an M-Braun Solvent Purification System. All reactions were monitored by EMD analytical thin layer chromatography (TLC Silica Gel 60 F_{254}, Glass Plates) and analyzed with 254 nm UV light and / or anisaldehyde – sulfuric acid or potassium permanganate treatment. Silica gel for column chromatography was purchased from Dynamic Adsorbents, Inc. or Sigma Aldrich (Flash Silica Gel 32-63u).

Unless otherwise noted, all ^1H and ^{13}C NMR spectra were recorded in $CDCl_3$ using a Bruker Ascend 400 spectrometer operating at 400 MHz for ^1H and 100 MHz for ^{13}C or Bruker Ascend 500 spectrometer operating at 500 MHz for ^1H and 125 MHz for ^{13}C. Chemical shifts (δ) are reported in ppm relative to residual $CHCl_3$ as an internal reference (^1H: 7.26 ppm, ^{13}C: 77.23 ppm). Coupling constants (J) are reported in Hertz (Hz). Peak multiplicity is indicated as follows: s (singlet), d (doublet), t (triplet), q (quartet), p (pentet), x (septet), h (heptet), b (broad), and m (multiplet). FT-IR spectra were recorded on Bruker Tensor 27 spectrometer and OPUS 6.5 Data Collection Program, and absorption frequencies were reported in reciprocal centimeters (cm^{-1}). High Resolution Mass Spectrometry – Electron Spray Ionization (HRMS-ESI) analyses were performed by the Louisiana State University Mass Spectrometry Facility using an Agilent 6210 Instrument. X-ray structure analyses were performed by the Louisiana State University X-

ray Structure Facility using a Bruker APEX-II CCD diffractometer. Gas Chromatography – Mass Spectrometry (GC-MS) were conducted on an Agilent Technologies 6890N Network GC System model number G1530N with 7683B series injector. The column used for this system was an Agilent HP-5MS 5% phenyl methyl siloxane (model number 19091S-433), which was 30 meters in length. The column had an internal diameter of 250 mm and film thickness of 0.25 mm. Solvent delay was set to 3.50 minutes for each trial. Low and high mass readings were set to parameters of 40 to 800 m/z, respectively. Oven, inlet, and detector temperatures were set to 250°C, and helium was used as the inert carrier gas.

CHARACTERIZATION OF NEW COMPOUNDS

(±)-*2-methoxy-3-methylcyclopent-2-enol* (12b)

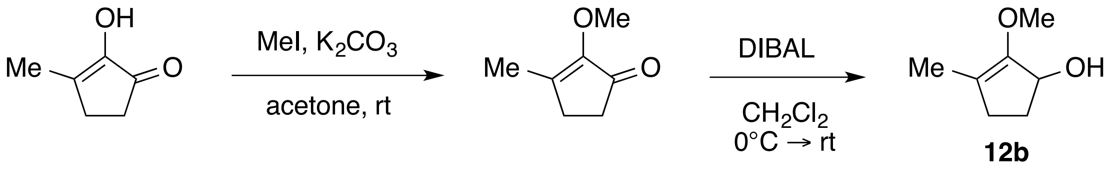

2-hydroxy-3-methylcyclopent-2-enone (4.00 g, 35.71 mmol) was dissolved in anhydrous acetone (180 mL). K_2CO_3 (9.90 g, 71.43 mmol) and then methyl iodide (4.2 mL, 71.41 mmol) were added. The reaction mixture was stirred at room temperature for 48 hours until the completion of reaction, as monitored by TLC. After concentrating the reaction mixture in *vacuo*, the crude residue was partitioned in EtOAc/H_2O (200 mL, 1:1). The aqueous layer extracted with EtOAc (3 x 100 mL). The combined organic layers were then washed with brine, dried over Na_2SO_4, and concentrated in *vacuo* to yield crude 2-methoxy-3-methylcyclopent-2-en-1-one (4.50 g).

Crude 2-methoxy-3-methylcyclopent-2-en-1-one (4.50 g) was dissolved in CH_2Cl_2 (180 mL) and cooled to 0ºC. DIBAL (53 mL, 1 M solution in toluene) was then added dropwise. The reaction mixture was warmed to room temperature and stirred for 1 hour until the completion of reaction, as monitored by TLC. After recooling the reaction mixture to 0ºC, EtOAc (50 mL) was added slowly, followed by water (100 mL). The mixture was vigorously stirred for 30 minutes. The resulting solid precipitate was then filtered through pad of celite. Upon separation of layers, the aqueous layer was extracted with CH_2Cl_2 (3 x 100 mL). The combined organic layers were then washed with brine, dried over Na_2SO_4, and concentrated in *vacuo*. The crude material was purified with flash column chromatography with 80 : 20 hexanes : EtOAc to give product **12b** (3.50 g, 76% yield over 2 steps) as colorless oil.

^{1}H NMR (500 MHz, $CDCl_3$): δ (ppm) = 4.82 – 4.73 (m, 1H), 3.71 (s, 3H), 2.38 – 2.32 (m, 1H), 2.26 – 2.19 (m, 1H), 2.12 – 2.06 (m, 1H), 1.71 – 1.68 (m, 2H), 1.65 (s, 3H). ^{13}C NMR (125 MHz, $CDCl_3$): δ (ppm) = 152.28, 116.85, 73.58, 57.41, 31.33, 30.99, 12.31. IR (cm^{-1}): 3353, 2928, 2850, 1690, 1453, 1332, 1192, 964, 696. HRMS $(M + H)^+$ = 127.0754 calculated for $C_7H_{11}O_2$; experimental = 127.0749.

(±)-*2-methoxy-3-methyl-1-phenylcyclopent-2-enol* (12c)

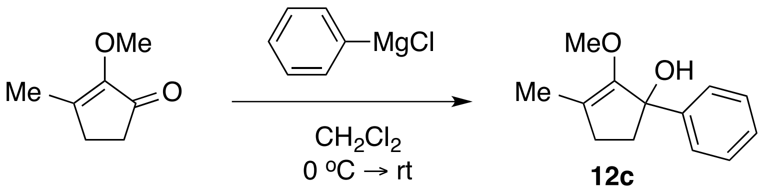

Crude 2-methoxy-3-methylcyclopent-2-enone (500 mg, 3.97 mmol) was dissolved in CH_2Cl_2 (10 mL) and cooled to 0ºC. Phenylmagnesium chloride (5.9 mL, 2 M solution in THF) was then added dropwise, and the mixture was allowed to warm to room temperature. After

stirring for 1 hour, the reaction was quenched with H_2O (15 mL). The aqueous layer was then extracted with EtOAc (3 x 20 mL). The combined organic layers were dried over Na_2SO_4 and then concentrated under vacuum. The crude material was purified with flash column chromatography (buffered with 2% TEA) with 85 : 15 hexanes : EtOAc to give product **12c** (524 mg, 65% yield) as colorless oil.

^{1}H NMR (400 MHz, $CDCl_3$): δ (ppm) = 7.47 – 7.41 (m, 2H), 7.34 (dd, J = 8.5, 6.9 Hz, 2H), 7.28 – 7.20 (m, 1H), 3.63 (s, 3H), 2.39 – 2.25 (m, 2H), 2.23 – 2.17 (m, 2H), 1.84 (s, 3H). ^{13}C NMR (100 MHz, $CDCl_3$): δ (ppm) = 153.39, 146.36, 128.43, 126.97, 125.09, 116.99, 85.20, 59.67, 40.64, 31.14, 13.30. IR (cm^{-1}): 3449, 2937, 2847, 1685, 1447, 1325, 1214, 1029, 761, 674. HRMS $(M + Na)^+$ = 227.1043 calculated for $C_{13}H_{16}NaO_2$; experimental = 227.1035.

(±)-*2-(tert-butyldimethylsilyloxy)-3-methyl-1-phenylcyclopent-2-enol* (12d)

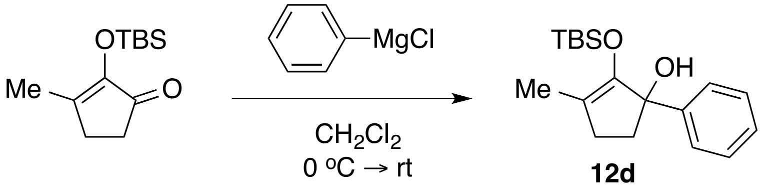

Crude 2-((tert-butyldimethylsilyl)oxy)-3-methylcyclopent-2-en-1-one [1] (500 mg, 2.21 mmol) was dissolved in CH_2Cl_2 (5.5 mL) and cooled to 0ºC. Phenylmagnesium chloride (2.2 mL, 2 M solution in THF) was then added dropwise, and the mixture was allowed to warm to room temperature. After stirring for 1 hour, the reaction was quenched with H_2O (15 mL). The aqueous layer was then extracted with EtOAc (3 x 20 mL). The combined organic layers were dried over Na_2SO_4 and then concentrated under vacuum. The crude material was purified with

[1] Ayala, C. E.; Dange, N. S.; Fronczek, F. R.; Kartika, R. *Angew. Chem. Int. Ed.* **2015**, *54*, 4641

flash column chromatography (buffered with 2% TEA) with 85 : 15 hexanes : EtOAc to give product **12d** (250 mg, 37% yield) as colorless oil.

^{1}H NMR (400 MHz, $CDCl_3$): δ (ppm) = 7.46 – 7.36 (m, 2H), 7.36 – 7.29 (m, 2H), 7.25 – 7.18 (m, 1H), 2.39 – 2.27 (m, 2H), 2.25 – 2.20 (m, 2H), 2.14 (s, 1H), 1.73 (s, 3H), 0.81 (s, 9H), 0.04 (s, 3H), -0.16 (s, 3H). ^{13}C NMR (100 MHz, $CDCl_3$): δ (ppm) = 149.23, 146.39, 128.21, 126.87, 125.48, 116.00, 84.96, 40.17, 30.45, 26.09, 25.93, 18.50, 13.21, -3.66, -3.86. IR (cm^{-1}): 2929, 2855, 1686, 1327, 1251, 1214, 1068, 1004, 855, 836, 779, 699. HRMS $(M + Na)^+$ = 327.1751 calculated for $C_{18}H_{28}NaO_2Si$; experimental = 327.1765.

(±)-*3-(2-(tert-butyldimethylsilyloxy)-1-methyl-3-phenylcyclopent-2-enyl)-1H-indole* (**14d**)

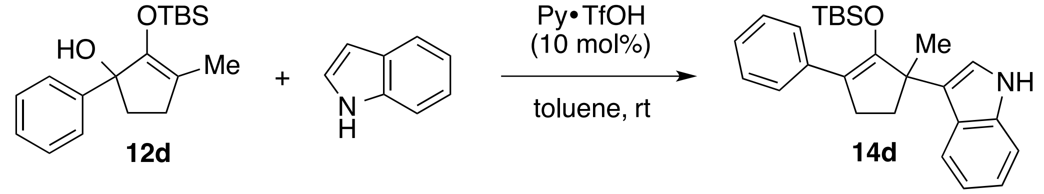

Compound **12d** (100 mg, 0.329 mmol) was dissolved in toluene (1.6 mL). Indole (77 mg, 0.657 mmol) and then pyridinium triflate (8 mg, 0.033 mmol) were added. Upon stirring at room temperature for 110 hours, the reaction mixture was concentrated under vacuum and then directly purified with flash column chromatography with 90 : 10 hexanes : Et_2O to give product **14d** (50 mg, 38% yield) as green solid.

^{1}H NMR (500 MHz, $CDCl_3$): δ (ppm) = 7.90 (bs, 1H), 7.83 (d, J = 8.0 Hz, 1H), 7.56 (dd, J = 8.1, 1.1 Hz, 2H), 7.42 – 7.33 (m, 3H), 7.26 – 7.18 (m, 2H), 7.16 – 7.09 (m, 1H), 7.07 (d, J = 2.4 Hz, 1H), 2.88 – 2.72 (m, 2H), 2.51 (ddd, J = 12.8, 8.8, 6.4 Hz, 1H), 2.08 (ddd, J = 13.0, 8.6, 4.6 Hz, 1H), 1.71 (s, 3H), 0.72 (s, 9H), -0.28 (s, 3H), -0.58 (s, 3H). ^{13}C NMR (125 MHz, $CDCl_3$): δ (ppm) = 154.45, 138.18, 137.18, 128.27, 128.09, 126.71, 126.04, 123.50, 121.82,

121.24, 121.02, 119.24, 114.75, 111.20, 48.53, 37.62, 30.71, 26.13, 25.09, 18.62, -3.37, -3.46. IR (cm^{-1}): 3422, 2929, 2855, 1638, 1471, 1338, 1252, 1078, 1063, 1013, 835, 737. HRMS $(M + H)^+$ = 404.2410 calculated for $C_{26}H_{34}NOSi$; experimental = 404.2410.

2-methoxy-3-methyl-1-phenylcyclohex-2-enol (**20**)

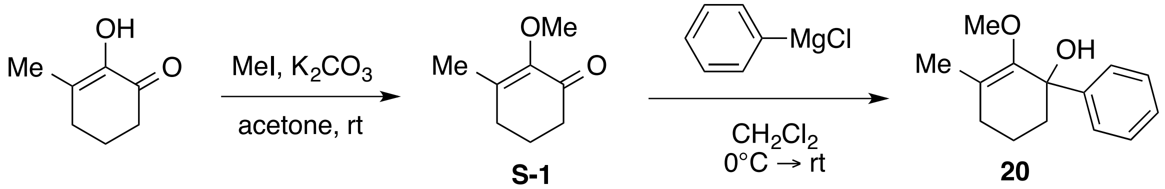

2-hydroxy-3-methylcyclohex-2-en-1-one (2.50 g, 19.84 mmol) was dissolved in anhydrous acetone (100 mL). K_2CO_3 (8.21 g, 59.52 mmol) and then methyl iodide (2.4 mL, 39.65 mmol) were added. The reaction mixture was stirred at room temperature for 96 hours until the completion of reaction, as monitored by TLC. After concentrating the reaction mixture in *vacuo*, the crude residue was partitioned in EtOAc/H_2O (60 mL, 1:1). The aqueous layer extracted with EtOAc (3 x 50 mL). The combined organic layers were then washed with brine, dried over Na_2SO_4, and concentrated in *vacuo*. The crude material was purified with flash column chromatography with 85 : 15 hexanes : EtOAc to give product **S-1** (2.00 g, 72% yield) as colorless oil.

^{1}H NMR (400 MHz, $CDCl_3$): δ (ppm) = 3.56 (s, 3H), 2.37 (t, J = 8.0 Hz, 2H), 2.33 (t, J = 6.0 Hz, 2H), 1.93 – 1.82 (m, 5H). ^{13}C NMR (100 MHz, $CDCl_3$): δ (ppm) = 194.72, 149.27, 146.06, 59.83, 38.71, 31.43, 22.16, 17.54. IR (cm-1): 2929, 1672, 1632, 1430, 1376, 1304, 1205, 1186, 1144, 1132, 1032, 999, 926, 847, 731. HRMS $(M + H)^+$ = 141.091calculated for $C_8H_{13}O_2$; experimental = 141.0908.

Ketone **S-1** (1.00 g, 7.14 mmol) was dissolved in CH_2Cl_2 (18 mL) and cooled to 0ºC. Phenylmagnesium chloride (5.5 mL, 2 M solution in THF) was then added dropwise, and the mixture was allowed to warm to room temperature. After stirring for 1 hour, the reaction was quenched with H_2O (50 mL). The aqueous layer was then extracted with EtOAc (3 x 50 mL). The combined organic layers were dried over Na_2SO_4 and then concentrated under vacuum. The crude material was purified with flash column chromatography with 90 : 10 hexanes : EtOAc to give product **20** (1.10 g, 70% yield) as colorless oil.

^{1}H NMR (400 MHz, $CDCl_3$): δ (ppm) = 7.55 – 7.49 (m, 2H), 7.38 – 7.31 (m, 2H), 7.29 – 7.21 (m, 1H), 3.52 (s, 3H), 2.74 (bs, 1H), 2.26 – 2.08 (m, 2H), 2.07 – 1.86 (m, 2H), 1.81 (s, 3H), 1.71 – 1.61 (m, 1H), 1.58 – 1.46 (m, 1H). ^{13}C NMR (100 MHz, $CDCl_3$): δ (ppm) = 150.43, 146.92, 128.03, 127.05, 126.32, 121.23, 76.25, 61.48, 41.01, 31.28, 18.97, 16.87. IR (cm-1): 3456, 2934, 2832, 1446, 1273, 1198, 1152, 1090, 1071, 759, 699. HRMS $(M + Na)^+$ = 241.1199 calculated for $C_{14}H_{18}NaO_2$; experimental = 241.1191.

(±)-*3-(2-methoxy-1-methyl-3-phenylcyclopent-2-enyl)-1H-indole* (**21a**)

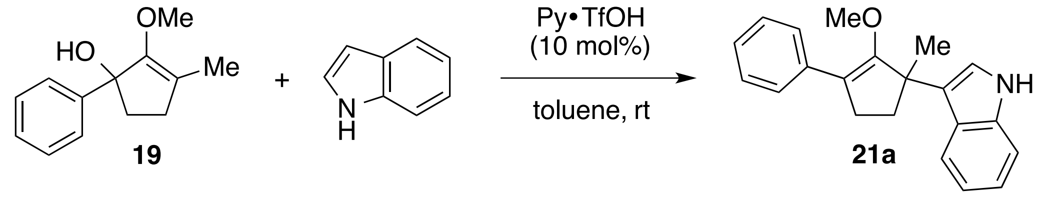

Compound **19** (50 mg, 0.245 mmol) was dissolved in toluene (1.2 mL). Indole (57 mg, 0.490 mmol) and then pyridinium triflate (6 mg, 0.024 mmol) were added. Upon stirring at room temperature for 2 hours, the reaction mixture was concentrated under vacuum and then directly purified with flash column chromatography with 90 : 10 hexanes : Et_2O to give product **21a** (60 mg, 81% yield) as green solid.

^{1}H NMR (500 MHz, $CDCl_3$): δ (ppm) = 7.96 (bs, 1H), 7.78 (d, J = 8.0 Hz, 1H), 7.63 (d, J = 8 Hz, 2H), 7.37 (dd, J = 6, 6 Hz, 3H), 7.24 – 7.17 (m, 2H), 7.12 – 7.02 (m, 2H), 3.44 (s, 3H), 2.88 (dt, J = 15, 8.0 Hz, 1H), 2.78 (ddd, J = 15, 9.0, 3.4 Hz, 1H), 2.52 (dt, J = 12.8, 9.0 Hz, 1H), 2.04 (ddd, J = 12.4, 9.0, 3.4 Hz, 1H), 1.74 (s, 3H). ^{13}C NMR (125 MHz, $CDCl_3$): δ (ppm) = 160.15, 137.26, 137.22, 128.25, 127.61, 126.43, 126.21, 123.46, 122.01, 120.87, 120.72, 119.45, 115.40, 111.39, 59.10, 48.30, 37.63, 29.99, 25.07. IR (cm^{-1}): 3412, 2960, 2933, 2844, 1629, 1598, 1456, 1416, 1335, 1260, 1128, 1078, 739, 697. HRMS $(M + Na)^+$ = 326.1515 calculated for $C_{21}H_{21}NNaO$; experimental = 326.1519.

(±)-*3-(2-methoxy-1-methyl-3-phenylcyclopent-2-enyl)-5-methyl-1H-indole* (21b)

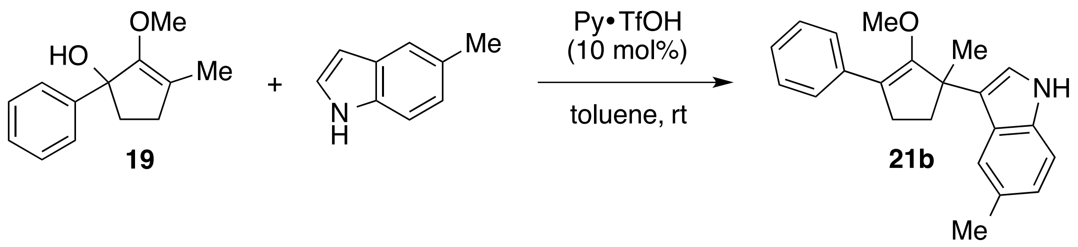

Compound **19** (50 mg, 0.245 mmol) was dissolved in toluene (1.2 mL). 5-Methylindole (64 mg, 0.490 mmol) and then pyridinium triflate (6 mg, 0.024 mmol) were added. Upon stirring at room temperature for 2 hours, the reaction mixture was concentrated under vacuum and then directly purified with flash column chromatography with 90 : 10 hexanes : Et_2O to give product **21b** (70 mg, 90% yield) as a colorless oil.

^{1}H NMR (500 MHz, $CDCl_3$): δ (ppm) = 7.86 (bs, 1H), 7.63 (d, J = 7.5 Hz, 2H), 7.55 (s, 1H), 7.37 (dd, J = 7.5, 7.5 Hz, 2H), 7.25 – 7.21 (m, 2H), 7.06 (d, J = 2.4 Hz, 1H), 7.01 (d, J = 8.2 Hz, 1H), 3.45 (s, 3H), 2.92 – 2.82 (m, 1H), 2.80 – 2.71 (m, 1H), 2.51 (ddd, J = 12.5, 9.0, 7.2 Hz, 1H), 2.42 (s, 3H) 2.03 (ddd, J = 12.5, 9.0, 3.7 Hz, 1H), 1.73 (s, 3H). ^{13}C NMR (125 MHz, $CDCl_3$): δ (ppm) = 160.26, 137.40, 135.61, 128.52, 128.25, 127.61, 126.76, 126.65, 126.15,

123.63, 122.90, 120.91, 120.52, 115.31, 111.04, 59.17, 48.33, 37.62, 30.05, 25.09, 21.98. IR (cm^{-1}): 3403, 2928, 2853, 1693, 1628, 1595, 1491, 1449, 1259, 1213, 1168, 1100, 1032, 780, 76. HRMS $(M + Na)^+$ = 340.1672 calculated for $C_{22}H_{23}NNaO$; experimental = 340.1678.

(±)-*5-methoxy-3-(2-methoxy-1-methyl-3-phenylcyclopent-2-enyl)-1H-indole* (21c)

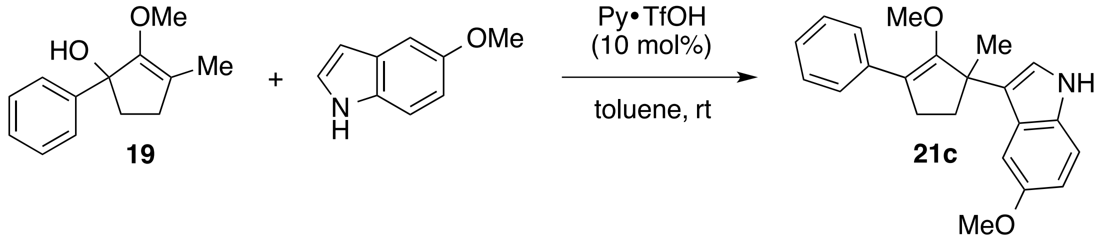

Compound **19** (50 mg, 0.245 mmol) was dissolved in toluene (1.2 mL). 5-Methoxyindole (64 mg, 0.490 mmol) and then pyridinium triflate (6 mg, 0.024 mmol) were added. Upon stirring at room temperature for 2 hours, the reaction mixture was concentrated under vacuum and then directly purified with flash column chromatography with 85 : 15 hexanes : Et_2O to give product **21c** (58 mg, 74% yield) as white solid.

^1H NMR (500 MHz, $CDCl_3$): δ (ppm) = 7.86 (bs, 1H), 7.63 (d, J = 7.4 Hz, 2H), 7.37 (dd, J = 7.7, 7.7 Hz, 2H), 7.27 – 7.18 (m, 3H), 7.06 (d, J = 2.0 Hz, 1H), 6.86 (dd, J = 8.8, 2.0 Hz, 1H), 3.76 (s, 3H), 3.46 (s, 3H), 2.91 (dt, J = 15.2, 7.8 Hz, 1H), 2.79 (ddd, J = 14.6, 9.0, 3.6 Hz, 1H), 2.50 (dt, J = 13, 9.0 Hz, 1H), 2.05 (ddd, J = 13, 9.0, 3.6 Hz, 1H), 1.74 (s, 3H). ^{13}C NMR (125 MHz, $CDCl_3$): δ (ppm) = 159.98, 153.77, 137.25, 132.43, 128.24, 127.58, 126.78, 126.22, 123.14, 121.52, 115.44, 112.00, 111.98, 102.81, 59.17, 56.00, 48.24, 37.42, 30.23, 25.00. IR (cm^{-1}): 3417, 2958, 2938, 1626, 1483, 1454, 1212, 1167, 1103, 762. HRMS $(M + Na)^+$ = 356.1621 calculated for $C_{22}H_{23}NNaO_2$; experimental = 356.1616.

(±)-*3-(2-methoxy-1-methyl-3-phenylcyclopent-2-enyl)-1-methyl-1H-indole* (21d)

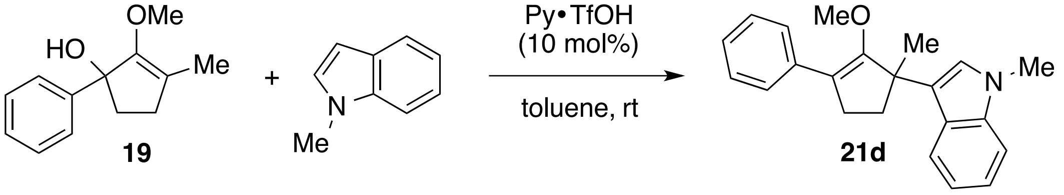

Compound **19** (50 mg, 0.245 mmol) was dissolved in toluene (1.2 mL). N-methylindole (64 mg, 0.490 mmol) and then pyridinium triflate (6 mg, 0.024 mmol) were added. Upon stirring at room temperature for 2 hours, the reaction mixture was concentrated under vacuum and then directly purified with flash column chromatography with 85 : 15 hexanes : Et_2O to give product **21d** (58 mg, 74% yield) as white solid.

^{1}H NMR (400 MHz, $CDCl_3$): δ (ppm) = 7.77 (d, J = 8.0 Hz, 1H), 7.64 (d, J = 7.5 Hz, 2H), 7.38 (dd, J = 7.6, 7.6 Hz, 2H), 7.31 (d, J = 8.2 Hz, 1H), 7.23 (dd, J = 7.4, 6.5 Hz, 2H), 7.07 (dd, J = 7.5, 7.5 Hz, 1H), 6.96 (s, 1H), 3.78 (s, 3H), 3.46 (s, 3H), 2.88 (dt, J = 15.4, 7.8 Hz, 1H), 2.83 – 2.71 (m, 1H), 2.51 (dt, J = 16.0, 8.2 Hz, 1H), 2.05 (ddd, J = 12.5, 8.7, 3.5 Hz, 1H), 1.74 (s, 3H). ^{13}C NMR (100 MHz, $CDCl_3$): δ (ppm) = 160.30, 137.92, 137.27, 128.24, 127.63, 126.78, 126.19, 125.63, 121.83, 121.56, 120.93, 118.86, 115.30, 109.44, 59.28, 48.32, 37.87, 32.88, 30.02, 25.13. IR (cm^{-1}): 2959, 2930, 2845, 1630, 1462, 1373, 1342, 1327, 1260, 1221, 1079, 1044, 737. HRMS $(M + H)^+$ = 318.1852 calculated for $C_{22}H_{24}NO$; experimental = 318.1845.

(±)-*5-chloro-3-(2-methoxy-1-methyl-3-phenylcyclopent-2-enyl)-1H-indole* (21e)

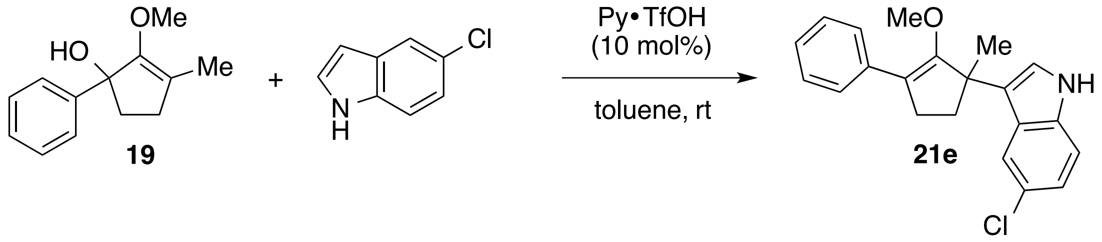

Compound **19** (50 mg, 0.245 mmol) was dissolved in toluene (1.2 mL). 5-Chloroindole (74 mg, 0.490 mmol) and then pyridinium triflate (6 mg, 0.024 mmol) were added. Upon stirring at room temperature for 2 hours, the reaction mixture was concentrated under vacuum and then directly purified with flash column chromatography with 90 : 10 hexanes : Et_2O to give product **21e** (62 mg, 75% yield) as white solid.

^{1}H NMR (500 MHz, $CDCl_3$): δ (ppm) = 7.94 (bs, 1H), 7.68 (d, J = 8.6 Hz, 1H), 7.62 (d, J = 7.7 Hz, 2H), 7.38 (t, J = 7.7 Hz, 2H), 7.34 (s, 1H), 7.24 (t, J = 7.4 Hz, 1H), 7.11 – 7.01 (m, 2H), 3.44 (s, 3H), 2.90 (dt, J = 15.6, 7.9 Hz, 1H), 2.77 (ddd, J = 14.7, 9.2, 3.4 Hz, 1H), 2.45 (ddd, J = 12.9, 9.1, 7.4 Hz, 1H), 2.05 (ddd, J = 12.6, 8.7, 3.4 Hz, 1H), 1.72 (s, 3H). ^{13}C NMR (125 MHz, $CDCl_3$): δ (ppm) = 159.83, 137.62, 137.04, 128.30, 128.05, 127.62, 126.36, 125.02, 123.67, 121.67, 121.37, 120.21, 115.61, 111.29, 59.22, 48.22, 37.63, 30.03, 24.91. IR (cm^{-1}): 3419, 2960, 2928, 2849, 1620, 1454, 1260, 1133, 1099, 906, 808. HRMS $(M + H)^+$ = 338.1306 calculated for $C_{21}H_{21}ClNO$; experimental = 338.1300.

(±)-*5-bromo-3-(2-methoxy-1-methyl-3-phenylcyclopent-2-enyl)-1H-indole* (21f)

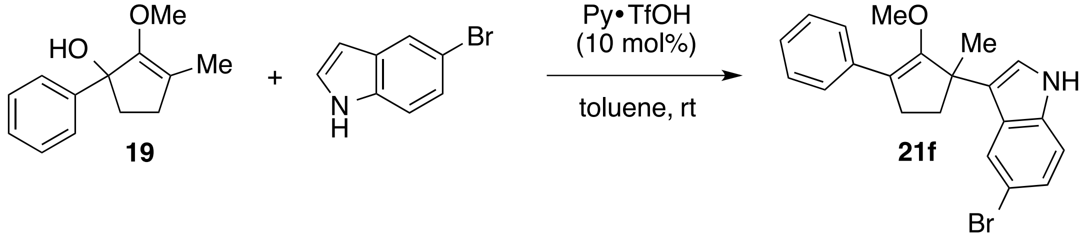

Compound **19** (50 mg, 0.245 mmol) was dissolved in toluene (1.2 mL). 5-Bromoindole (96 mg, 0.490 mmol) and then pyridinium triflate (6 mg, 0.024 mmol) were added. Upon stirring at room temperature for 2 hours, the reaction mixture was concentrated under vacuum and then directly purified with flash column chromatography with 85 : 15 hexanes : Et_2O to give product **21f** (80 mg, 85% yield) as white solid.

^{1}H NMR (500 MHz, $CDCl_3$): δ (ppm) = 7.99 (bs, 1H), 7.91 (d, J = 1.6 Hz, 1H), 7.60 (d, J = 7.1 Hz, 2H), 7.39 (dd, J = 7.7, 7.7 Hz, 2H), 7.31 – 7.18 (m, 3H), 7.09 (d, J = 2.4 Hz, 1H), 3.45 (s, 3H), 2.87 (ddd, J = 15.0, 9.0, 7.0 Hz, 1H), 2.75 (ddd, J = 15.0, 9.0, 3.7 Hz, 1H), 2.44 (ddd, J = 13.0, 9.0, 7.0 Hz, 1H), 2.05 (ddd, J = 13.0, 9.0, 3.7 Hz, 1H), 1.72 (s, 3H). ^{13}C NMR (125 MHz, $CDCl_3$): δ (ppm) = 159.59, 137.18, 135.83, 128.28, 128.11, 127.77, 126.38, 124.89, 123.36, 123.15, 122.07, 115.62, 112.81, 112.74, 59.51, 48.31, 37.48, 30.34, 24.99. IR (cm^{-1}): 3421, 2959, 2930, 2850, 1637, 1459, 1325, 1239, 1105, 796, 762, 698. HRMS $(M + Na)^+$ = 404.0620 calculated for $C_{21}H_{20}BrNNaO$; experimental = 404.0622.

(±)-*methyl 3-(2-methoxy-1-methyl-3-phenylcyclopent-2-enyl)-1H-indole-5-carboxylate* (**21g**)

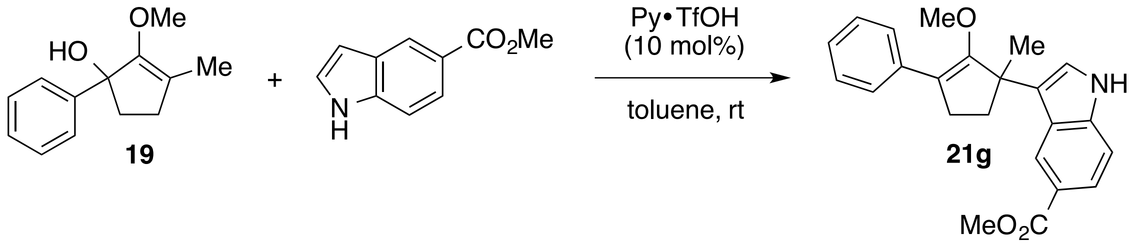

Compound **19** (50 mg, 0.245 mmol) was dissolved in toluene (1.2 mL). Methyl indole-5-carboxylate (86 mg, 0.490 mmol) and then pyridinium triflate (6 mg, 0.024 mmol) were added. Upon stirring at room temperature for 6 hours, the reaction mixture was concentrated under vacuum and then directly purified with flash column chromatography with 65 : 35 hexanes : Et_2O to give product **21g** (60 mg, 68% yield) as white solid.

^{1}H NMR (500 MHz, $CDCl_3$): δ (ppm) = 8.61 (s, 1H), 8.18 (bs, 1H), 7.90 (d, J = 10.0 Hz, 1H), 7.61 (d, J = 7.2 Hz, 2H), 7.43 – 7.32 (m, 3H), 7.24 (dd, J = 7.4, 7.4 Hz, 1H), 7.15 (d, J = 2.3, 2.3 Hz, 1H), 3.89 (s, 3H), 3.44 (s, 3H), 2.87 (ddd, J = 15.0, 8.5, 6.6 Hz, 1H), 2.83 – 2.74 (m, 1H), 2.44 (ddd, J = 13, 8.5, 6.6 Hz, 1H), 2.10 (ddd, J = 13, 8.5, 4.2 Hz, 1H), 1.74 (s, 3H). ^{13}C NMR (125 MHz, $CDCl_3$): δ (ppm) = 168.45, 159.42, 139.82, 137.40, 128.20, 127.96, 126.39, 125.97, 124.96, 123.90, 123.49, 122.05, 121.53, 115.70, 111.08, 59.69, 51.98, 48.47, 37.77, 30.85, 25.15. IR (cm^{-1}): 3337, 2958, 2927, 2849, 1691, 1616, 1435, 1315, 1296, 1257, 1096, 987, 907, 801, 754. HRMS $(M + H)^+$ = 362.1751 calculated for $C_{22}H_{24}NO_3$; experimental = 362.1754.

(±)-*5-methoxy-3-(2-methoxy-1-methyl-3-phenylcyclopent-2-en-1-yl)-1H-benzo[g]indole* (21h)

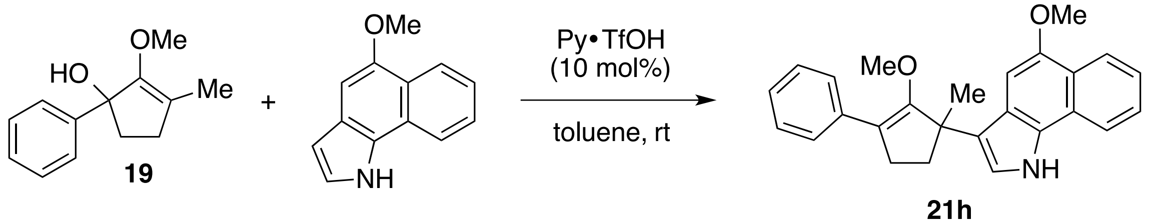

Compound **19** (50 mg, 0.245 mmol) was dissolved in toluene (1.2 mL). 5-methoxy-1H-benzo[*g*]indole (97 mg, 0.490 mmol) and then pyridinium triflate (6 mg, 0.025 mmol) were added. Upon stirring at room temperature for 1.5 hours, the reaction mixture was then directly purified with flash column chromatography with 100% hexanes → 90 : 10 hexanes : Et_2O → 80 : 20 hexanes : Et_2O to give product **21h** (89 mg, 94% yield) as colorless oil.

^{1}H NMR (500 MHz, $CDCl_3$): δ = 8.81 (bs, 1H), 8.31 (d, J = 8.3 Hz, 1H), 7.94 (d, J = 8.1 Hz, 1H), 7.62-7.60 (m, 2H), 7.53 (ddd, J = 8.2, 6.9, 1.3 Hz, 1H), 7.42-7.36 (m, 3H), 7.27-7.23 (m, 1H), 7.03 (s, 1H), 6.45 (d, J = 2.3 Hz, 1H), 4.03 (s, 3H), 3.54 (s, 3H), 2.83-2.80 (m, 2H), 2.95 (ddd, J = 12.8, 7.1, 7.1 Hz, 1H), 2.20-2.15 (m, 1H), 1.75 (s, 3H). ^{13}C NMR (125 MHz, $CDCl_3$): δ = 158.04, 150.25, 143.38, 136.15, 129.04, 128.16, 127.49, 126.56, 125.95, 123.52,

123.36, 123.14, 122.95, 122.03, 119.09, 116.86, 99.49, 98.08, 59.23, 55.87, 48.73, 37.53, 29.73, 24.96. IR (neat): cm^{-1}; 3449, 2960, 2936, 2844, 1631, 1598, 1517, 1493, 1479, 1445, 1381, 1342, 1326, 1308, 1291, 1273, 1258, 1217, 1172, 1160, 1126, 1099, 1079, 1035, 1003, 986, 909, 834, 761, 731, 698, 463. HRMS $(M + H)^+$ = 384.1958 calculated for $C_{26}H_{26}NO_2$; experimental = 384.1952.

(±)-*3-(2-methoxy-1-methyl-3-phenylcyclohex-2-enyl)-1H-indole* (22a)

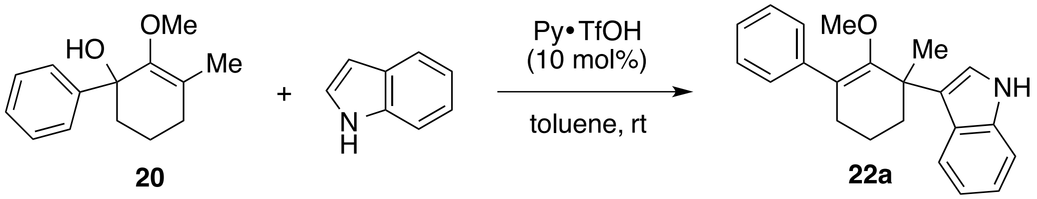

20 **22a**

Compound **20** (50 mg, 0.229 mmol) was dissolved in toluene (1.1 mL). Indole (54 mg, 0.459 mmol) and then pyridinium triflate (5 mg, 0.023 mmol) were added. Upon stirring at room temperature for 24 hours, the reaction mixture was concentrated under vacuum and then directly purified with flash column chromatography with 85 : 15 hexanes : Et_2O to give product **22a** (58 mg, 80% yield) as white solid.

^{1}H NMR (500 MHz, $CDCl_3$): δ (ppm) = 7.94 (bs, 1H), 7.89 (d, J = 8.0 Hz, 1H), 7.49 (d, J = 7.1 Hz, 2H), 7.35 (dd, J = 7.6, 7.6 Hz, 3H), 7.24 (dd, J = 7.4, 7.4 Hz, 1H), 7.19 (dd, J = 7.9, 7.9 Hz, 1H), 7.12 (dd, J = 7.9, 7.9 Hz, 1H), 7.09 (d, J = 2.4 Hz, 1H), 3.09 (s, 3H), 2.65 (dt, J = 16.8, 5.6 Hz, 1H), 2.57 (ddd, J = 16.8, 7.7, 5.2 Hz, 1H), 2.48 – 2.38 (m, 1H), 1.88 – 1.76 (m, 3H), 1.73 (s, 3H). ^{13}C NMR (125 MHz, $CDCl_3$): δ (ppm) = 158.06, 141.61, 137.25, 128.61, 128.35, 126.44, 125.99, 123.78, 122.11, 121.76, 121.00, 119.65, 119.20, 111.61, 61.19, 40.89, 39.22, 31.85, 25.05, 20.47. IR (cm^{-1}): 3412, 2928, 2858, 1490, 1457, 1335, 1192, 1132, 1011, 762, 739, 699. HRMS $(M + Na)^+$ = 340.1672 calculated for $C_{22}H_{23}NNaO$; experimental = 340.1668.

(±)-*5-methoxy-3-(2-methoxy-1-methyl-3-phenylcyclohex-2-enyl)-1H-indole* (22b)

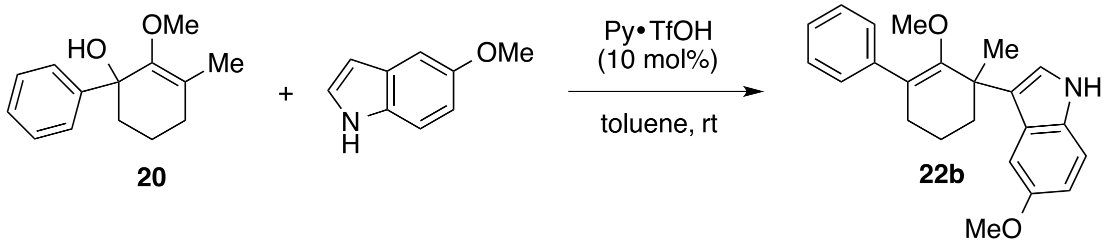

Compound **20** (50 mg, 0.229 mmol) was dissolved in toluene (1.1 mL). 5-Methoxyindole (67 mg, 0.459 mmol) and then pyridinium triflate (5 mg, 0.023 mmol) were added. Upon stirring at room temperature for 24 hours, the reaction mixture was concentrated under vacuum and then directly purified with flash column chromatography with 85 : 15 hexanes : Et_2O to give product **22a** (55 mg, 69% yield) as white solid.

^1H NMR (500 MHz, $CDCl_3$): δ (ppm) = 7.86 (bs, 1H), 7.49 (d, J = 7.3 Hz, 2H), 7.38 – 7.31 (m, 3H), 7.23 (dd, J = 9.0, 9.0 Hz, 2H), 7.07 (d, J = 2.4 Hz, 1H), 6.86 (dd, J = 9.0, 2.4 Hz, 1H), 3.82 (s, 3H), 3.09 (s, 3H), 2.68 (dt, J = 16.9, 4.4 Hz, 1H), 2.62 – 2.51 (m, 1H), 2.46 – 2.36 (m, 1H), 1.91 – 1.75 (m, 3H), 1.71 (s, 3H). ^{13}C NMR (125 MHz, $CDCl_3$): δ (ppm) = 158.08, 153.58, 141.47, 132.41, 128.61, 128.36, 126.50, 126.26, 123.49, 122.77, 119.65, 112.26, 112.09, 102.69, 61.22, 56.00, 40.75, 39.04, 31.98, 24.85, 20.55. IR (cm^{-1}): 3410, 2926, 2855, 2832, 1482, 1454, 1261, 1191, 1141, 1108, 1003, 907, 796, 729, 698. HRMS $(M + Na)^+$ = 370.1778 calculated for $C_{23}H_{25}NNaO_2$; experimental = 370.1767.

(±)-*5-bromo-3-(2-methoxy-1-methyl-3-phenylcyclohex-2-enyl)-1H-indole* (22c)

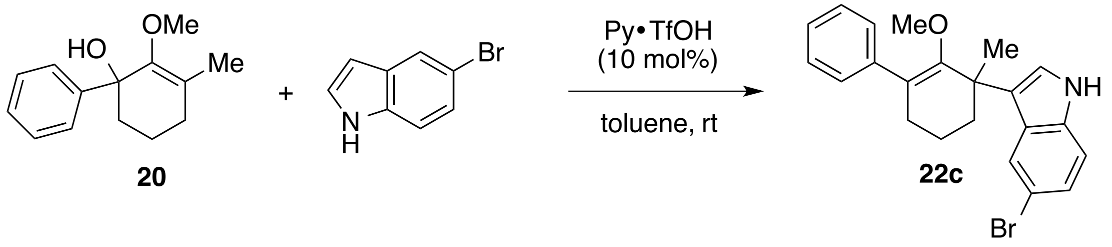

Compound **20** (50 mg, 0.229 mmol) was dissolved in toluene (1.1 mL). 5-Bromoindole (90 mg, 0.459 mmol) and then pyridinium triflate (5 mg, 0.023 mmol) were added. Upon stirring at room temperature for 24 hours, the reaction mixture was concentrated under vacuum and then directly purified with flash column chromatography with 95 : 5 hexanes : Et_2O to give product **22c** (77 mg, 85% yield) as white solid.

^{1}H NMR (500 MHz, $CDCl_3$): δ (ppm) = 8.01 (s, 1H), 7.99 (bs, 1H), 7.46 (d, J = 7.4 Hz, 2H), 7.37 (dd, J = 7.4, 7.4 Hz, 2H), 7.27 – 7.17 (m, 3H), 7.08 (s, 1H), 3.09 (s, 3H), 2.69 – 2.48 (m, 2H), 2.40 – 2.25 (m, 1H), 1.84 – 1.7 (m, 3H), 1.70 (s, 3H). ^{13}C NMR (125 MHz, $CDCl_3$): δ (ppm) = 157.39, 141.47, 135.80, 128.58, 128.46, 127.72, 126.58, 124.61, 123.61, 123.58, 123.32, 120.06, 112.96, 112.54, 61.26, 40.82, 39.28, 31.97, 25.03, 20.36. IR (cm^{-1}): 3423, 3323, 2927, 2856, 1460, 1261, 1136, 1106, 1015, 796, 762, 699. HRMS $(M + H)^+$ = 418.0777 calculated for $C_{22}H_{22}BrNNaO$; experimental = 418.077.

(±)-*methyl 3-(2-methoxy-1-methyl-3-phenylcyclohex-2-enyl)-1H-indole-5-carboxylate* (22d)

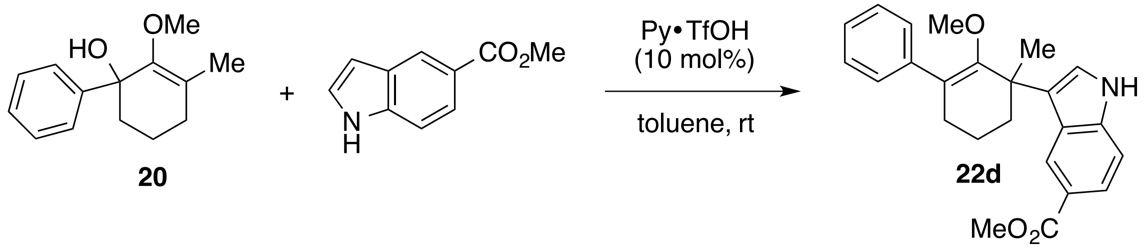

Compound **20** (50 mg, 0.229 mmol) was dissolved in toluene (1.1 mL). Methyl indole-5-carboxylate (80 mg, 0.459 mmol) and then pyridinium triflate (5 mg, 0.023 mmol) were added. Upon stirring at room temperature for 30 hours, the reaction mixture was concentrated under vacuum and then directly purified with flash column chromatography with 60 : 40 hexanes : Et_2O to give product **22d** (30 mg, 35% yield) as white solid.

^{1}H NMR (500 MHz, $CDCl_3$): δ (ppm) = 8.70 (s, 1H), 8.14 (bs, 1H), 7.90 (dd, J = 8.6, 1.5 Hz, 1H), 7.48 (d, J = 7.1 Hz, 2H), 7.39 – 7.31 (m, 3H), 7.23 (t, J = 7.4, 7.4 Hz, 1H), 7.17 (d, J = 2.3 Hz, 1H), 3.90 (s, 3H), 3.05 (s, 3H), 2.66 – 2.51 (m, 2H), 2.36 (ddd, J = 13.2, 9.9, 3.4 Hz, 1H), 1.86 – 1.75 (m, 3H), 1.73 (s, 3H). ^{13}C NMR (125 MHz, $CDCl_3$): δ (ppm) = 168.44, 157.17, 141.67, 139.78, 128.63, 128.36, 126.50, 125.71, 125.50, 124.14, 123.35, 123.24, 121.36, 120.26, 111.20, 61.24, 51.97, 40.89, 39.80, 32.16, 25.26, 20.40. IR (cm^{-1}): 3341, 2921, 2852, 1690, 1647, 1435, 1247, 1115, 1092, 906, 733. HRMS $(M + H)^+$ = 376.1907 calculated for $C_{24}H_{26}NO_3$; experimental = 398.1914.

(±)-*3-(2-methoxy-1-methyl-3-phenylcyclohex-2-enyl)-2-phenyl-1H-indole* (22e)

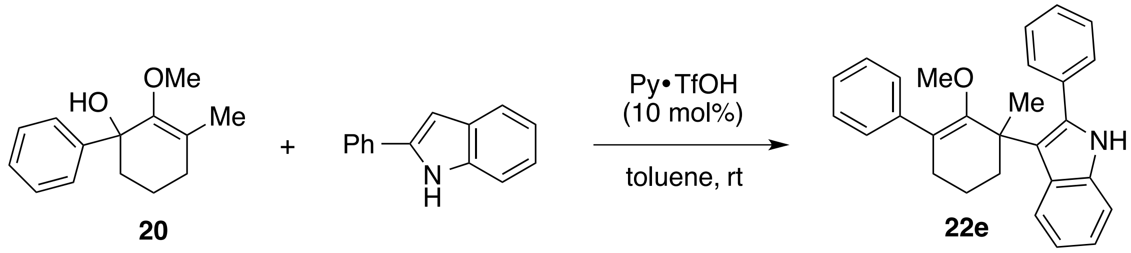

20 **22e**

Compound **20** (50 mg, 0.229 mmol) was dissolved in toluene (1.1 mL). 2-Phenylindole (88 mg, 0.459 mmol) and then pyridinium triflate (5 mg, 0.023 mmol) were added. Upon stirring at room temperature for 24 hours, the reaction mixture was concentrated under vacuum and then directly purified with flash column chromatography with 60 : 40 hexanes : Et_2O to give product **22e** (78 mg, 87% yield) as a white solid.

^{1}H NMR (500 MHz, $CDCl_3$): δ (ppm) = 8.01 (s, 1H), 7.99 (bs, 1H), 7.46 (d, J = 7.4 Hz, 2H), 7.37 (dd, J = 7.4, 7.4 Hz, 2H), 7.27 – 7.17 (m, 3H), 7.08 (s, 1H), 3.09 (s, 3H), 2.69 – 2.48 (m, 2H), 2.40 – 2.25 (m, 1H), 1.84 – 1.7 (m, 3H), 1.70 (s, 3H). ^{13}C NMR (125 MHz, $CDCl_3$): δ (ppm) = 157.39, 141.47, 135.80, 128.58, 128.46, 127.72, 126.58, 124.61, 123.61, 123.58, 123.32, 120.06, 112.96, 112.54, 61.26, 40.82, 39.28, 31.97, 25.03, 20.36. IR (cm^{-1}): 3423, 3323,

2927, 2856, 1460, 1261, 1136, 1106, 1015, 796, 762, 699. HRMS $(M + Na)^+$ = 416.1985 calculated for $C_{28}H_{27}NNaO$; experimental = 416.1983.

(±)-*3-(2-methoxy-1-methyl-3-phenylcyclohex-2-enyl)-1-methyl-1H-indole* (22f)

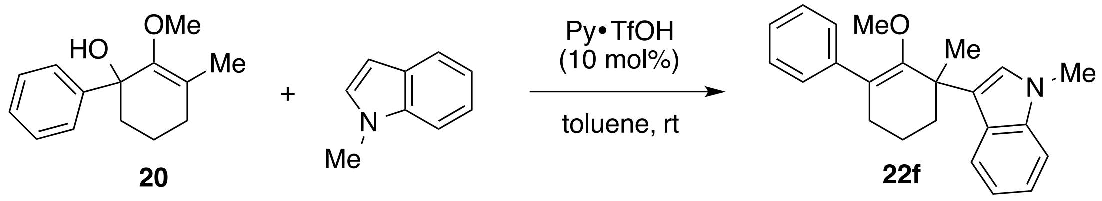

20

22f

Compound **20** (50 mg, 0.229 mmol) was dissolved in toluene (1.1 mL). N-Methylindole (60 mg, 0.459 mmol) and then pyridinium triflate (5 mg, 0.023 mmol) were added. Upon stirring at room temperature for 96 hours, the reaction mixture was concentrated under vacuum and then directly purified with flash column chromatography with 60 : 40 hexanes : Et_2O to give product **22f** (40 mg, 53% yield) as colorless oil.

^{1}H NMR (500 MHz, $CDCl_3$): δ (ppm) = 7.87 (d, J = 8.0 Hz, 1H), 7.49 (d, J = 8.3 Hz, 2H), 7.41 – 7.29 (m, 3H), 7.26 – 7.18 (m, 2H), 7.10 (dd, J = 7.5, 7.5 Hz, 1H), 6.98 (s, 1H), 3.79 (s, 3H), 3.11 (s, 3H), 2.70 – 2.61 (m, 1H), 2.55 (dt, J = 16.9, 6.5 Hz, 1H), 2.47 – 2.37 (m, 1H), 1.86 – 1.77 (m, 3H), 1.72 (s, 3H). ^{13}C NMR (125 MHz, $CDCl_3$): δ (ppm) = 158.23, 141.61, 137.87, 128.61, 128.33, 126.95, 126.41, 126.32, 122.17, 121.32, 121.07, 119.55, 118.63, 109.64, 61.26, 40.89, 39.49, 32.90, 31.84, 25.12, 20.51. IR (cm^{-1}): 2928, 1464, 1324, 1259, 1011, 907, 731, 697. HRMS $(M + Na)^+$ = 354.1828 calculated for $C_{23}H_{25}NNaO$; experimental = 354.1822.

(±)-*1-(4-methoxyphenyl)-2-methoxy-3-methylcyclopent-2-enol* (23a)

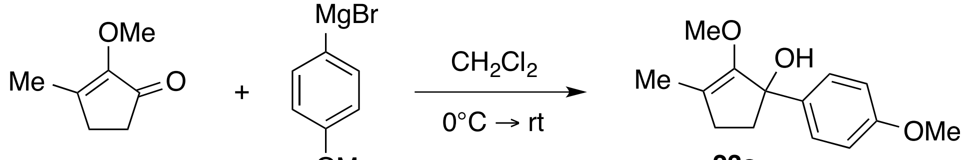

23a

Crude 2-methoxy-3-methylcyclopent-2-enone (250 mg, 1.98 mmol) was dissolved in CH_2Cl_2 (10 mL) and cooled to 0ºC. 4-Methoxyphenylmagnesium bromide (6.0 mL, 0.5 M solution in THF) was then added dropwise, and the mixture was allowed to warm to room temperature. After stirring for 1 hour, the reaction was quenched with H_2O (15 mL). The aqueous layer was then extracted with EtOAc (3 x 20 mL). The combined organic layers were dried over Na_2SO_4 and then concentrated under vacuum. The crude material was purified with flash column chromatography with 85 : 15 hexanes : EtOAc to give product **23a** (375 mg, 80% yield) as colorless oil.

^{1}H NMR (500 MHz, $CDCl_3$): δ (ppm) = 7.36 (d, J = 9.0 Hz, 2H), 6.88 (d, J = 9.0 Hz, 2H), 3.80 (s, 3H), 3.63 (s, 3H), 2.55 (s, 1H), 2.39 – 2.28 (m, 1H), 2.30 – 2.15 (m, 3H), 1.83 (s, 3H). ^{13}C NMR (125 MHz, $CDCl_3$): δ (ppm) = 158.47, 153.47, 138.49, 126.22, 116.70, 113.63, 84.70, 59.51, 55.30, 40.59, 30.90, 13.17. IR (cm^{-1}): 3465, 2935, 2845, 1684, 1609, 1508, 1243, 1171, 1031, 829, 544. HRMS $(M + Na)^+$ = 257.1148 calculated for $C_{14}H_{18}NaO_3$; experimental = 257.1142.

(±)-*2-methoxy-3-methyl-1-p-tolylcyclopent-2-enol* (23b)

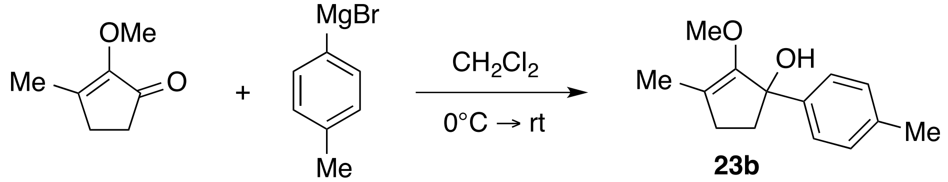

23b

Crude 2-methoxy-3-methylcyclopent-2-enone (500 mg, 3.97 mmol) was dissolved in CH_2Cl_2 (20 mL) and cooled to 0ºC. *p*-Tolylmagnesium bromide (11.9 mL, 0.5 M solution in Et_2O) was then added dropwise, and the mixture was allowed to warm to room temperature. After stirring for 1 hour, the reaction was quenched with H_2O (15 mL). The aqueous layer was then extracted with EtOAc (3 x 20 mL). The combined organic layers were dried over Na_2SO_4 and then concentrated under vacuum. The crude material was purified with flash column chromatography with 92.5 : 7.5 hexanes : EtOAc to give product **23b** (366 mg, 42% yield) as pale yellow oil.

^{1}H NMR (400 MHz, $CDCl_3$): δ (ppm) = 7.35 (d, J = 8.0 Hz, 2H), 7.17 (d, J = 8.0 Hz, 2H), 3.65 (s, 3H), 2.46 – 2.33 (m, 5H), 2.31 – 2.18 (m, 3H), 1.86 (s, 3H). ^{13}C NMR (100 MHz, $CDCl_3$): δ (ppm) = 153.46, 143.40, 136.44, 129.08, 124.99, 116.74, 85.03, 59.59, 40.62, 31.07, 21.19, 13.26. IR (cm^{-1}): 3453, 2967, 2936, 2847, 1685, 1511, 1441, 1407, 1379, 1325, 1067, 817, 639, 537. HRMS $(M + H)^+$ = 241.1199 calculated for $C_{14}H_{18}NaO_2$; experimental = 241.1215.

(±)-*1-(4-fluorophenyl)-2-methoxy-3-methylcyclopent-2-enol* (23c)

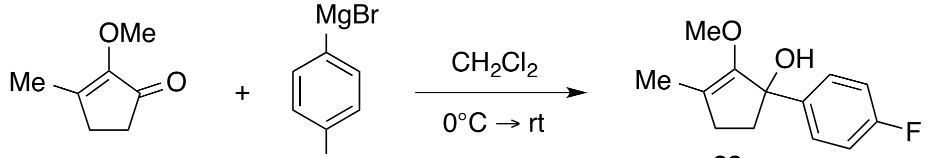

23c

Crude 2-methoxy-3-methylcyclopent-2-enone (250 mg, 1.98 mmol) was dissolved in CH_2Cl_2 (10 mL) and cooled to 0ºC. 4-Fluorophenylmagnesium bromide (3.0 mL, 1 M solution in THF) was then added dropwise, and the mixture was allowed to warm to room temperature. After stirring for 1 hour, the reaction was quenched with H_2O (15 mL). The aqueous layer was then extracted with EtOAc (3 x 20 mL). The combined organic layers were dried over Na_2SO_4

and then concentrated under vacuum. The crude material was purified with flash column chromatography with 92.5 : 7.5 hexanes : EtOAc to give product **23c** (420 mg, 94% yield) as colorless oil.

^{1}H NMR (400 MHz, $CDCl_3$): δ (ppm) = 7.40 (dd, J = 8.7, 5.5 Hz, 2H), 7.02 (t, J = 8.7 Hz, 2H), 3.64 (s, 3H), 2.64 – 2.00 (m, 5H), 1.84 (s, 3H). ^{13}C NMR (100 MHz, $CDCl_3$): δ (ppm) = 163.12, 160.69, 153.20, 142.09, 142.06, 126.87, 126.79, 117.09, 115.15, 114.94, 84.77, 59.62, 40.63, 30.99, 13.23. IR (cm^{-1}): 3443, 2968, 2937, 2849, 1684, 1601, 1506, 1216, 1068, 833, 538. HRMS $(M + Na)^+$ = 245.0948 calculated for $C_{13}H_{15}FNaO_2$; experimental = 245.0949.

(±)-*1-(4-chlorophenyl)-2-methoxy-3-methylcyclopent-2-enol* (23d)

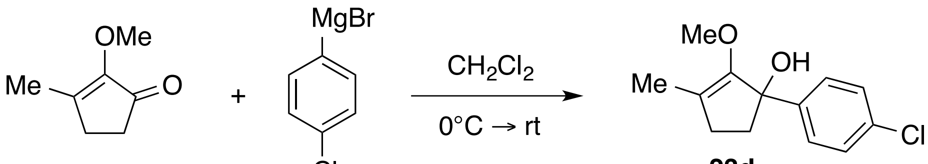

Crude 2-methoxy-3-methylcyclopent-2-enone (500 mg, 3.97 mmol) was dissolved in CH_2Cl_2 (20 mL) and cooled to 0ºC. 4-Chlorophenylmagnesium bromide (6.0 mL, 1 M solution in THF) was then added dropwise, and the mixture was allowed to warm to room temperature. After stirring for 1 hour, the reaction was quenched with H_2O (15 mL). The aqueous layer was then extracted with EtOAc (3 x 20 mL). The combined organic layers were dried over Na_2SO_4 and then concentrated under vacuum. The crude material was purified with flash column chromatography with 85 : 15 hexanes : EtOAc to give product **23d** (689 mg, 83% yield) as pale yellow oil.

^{1}H NMR (400 MHz, $CDCl_3$): δ (ppm) = 7.37 (d, J = 8.6 Hz, 2H), 7.30 (d, J = 8.6 Hz, 2H), 3.63 (s, 3H), 2.49 – 2.11 (m, 5H), 1.84 (s, 3H). ^{13}C NMR (100 MHz, $CDCl_3$): δ (ppm) =

153.04, 144.93, 132.70, 128.51, 126.69, 117.20, 84.86, 59.69, 40.59, 31.11, 13.30. IR (cm^{-1}): 3443, 2968, 2936, 2848, 1902, 1684, 1488, 1324, 1089, 1012, 827, 537. HRMS $(M + Na)^+$ = 261.0653 calculated for $C_{13}H_{15}ClNaO_2$; experimental= 261.0655.

(±)-*2-methoxy-3-methyl-1-(3-methylthiophen-2-yl)cyclopent-2-enol* (**23e**)

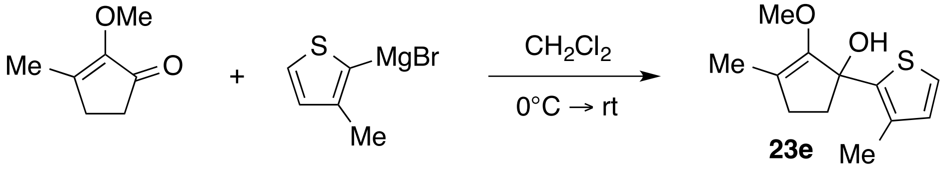

Crude 2-methoxy-3-methylcyclopent-2-enone (200 mg, 1.56 mmol) was dissolved in CH_2Cl_2 (8.0 mL) and cooled to 0ºC. 3-Methyl-2-thienylmagnesium bromide (6.4 mL, 0.5 M solution in THF) was then added dropwise, and the mixture was allowed to warm to room temperature. After stirring for 1 hour, the reaction was quenched with H_2O (15 mL). The aqueous layer was then extracted with EtOAc (3 x 20 mL). The combined organic layers were dried over Na_2SO_4 and then concentrated under vacuum. The crude material was purified with flash column chromatography (buffered with 2% TEA) with 85 : 15 hexanes : EtOAc to give product **23e** (250 mg, 70% yield) as yellow oil.

^1H NMR (500 MHz, $CDCl_3$): δ (ppm) = 7.05 (d, J = 5.1 Hz, 1H), 6.80 (d, J = 5.1 Hz, 1H), 3.71 (s, 3H), 2.56 (bs, 1H), 2.44 – 2.33 (m, 2H), 2.30 – 2.24 (m, 2H), 2.23 (s, 3H), 1.82 (s, 3H). ^{13}C NMR (125 MHz, $CDCl_3$): δ (ppm) = 152.55, 143.42, 132.14, 131.83, 121.70, 117.49, 83.97, 59.47, 39.25, 31.07, 14.29, 13.12. IR (cm^{-1}): 3441, 2969, 2848, 1684, 1440, 1324, 1262, 1214, 1175, 1066, 991, 925, 855, 707. HRMS $(M + Na)^+$ = 247.0763 calculated for $C_{12}H_{16}NaO_2S$; experimental = 247.0761.

(±)-*2-methoxy-3-methyl-1-(naphthalen-1-yl)cyclopent-2-enol* (23f)

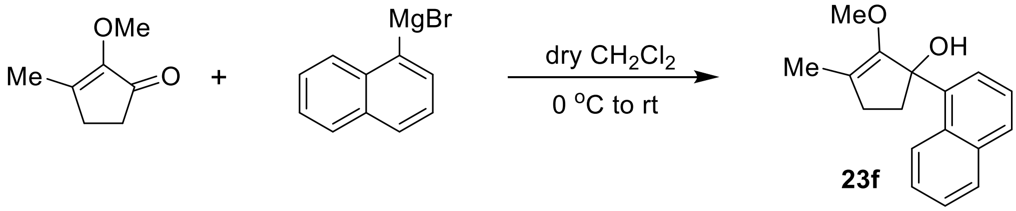

Crude 2-methoxy-3-methylcyclopent-2-enone (100 mg, 0.793 mmol) was dissolved in CH_2Cl_2 (4.0 mL) and cooled to 0ºC. 1-Napthylmagnesium bromide (12.6 mL, 0.25 M solution in THF) was then added dropwise, and the mixture was allowed to warm to room temperature. After stirring for 24 hours, the reaction was quenched with H_2O (15 mL). The aqueous layer was then extracted with EtOAc (3 x 20 mL). The combined organic layers were dried over Na_2SO_4 and then concentrated under vacuum. The crude material was purified with flash column chromatography (buffered with 2% TEA) with 85 : 15 hexanes : EtOAc to give product **23e** (153 mg, 76% yield) as colorless oil.

^{1}H NMR (400 MHz, $CDCl_3$): δ (ppm) = 8.58 (d, J = 8.2 Hz, 1H), 7.87 (d, J = 7.4 Hz, 1H), 7.78 (d, J = 8.1 Hz, 1H), 7.57 – 7.45 (m, 3H), 7.41 (dd, J = 7.7, 7.7 Hz, 1H), 3.83 (s, 3H), 2.69 (s, 1H), 2.59 (ddd, J = 12.3, 8.3, 2.3 Hz, 1H), 2.50 – 2.33 (m, 2H), 2.21 (dt, J = 14.5, 6.4 Hz, 1H), 1.94 (s, 3H). ^{13}C NMR (100 MHz, $CDCl_3$): δ (ppm) = 153.44, 140.47, 134.98, 131.07, 129.04, 128.61, 126.81, 125.62, 125.39, 124.96, 124.25, 116.33, 86.66, 59.99, 38.69, 31.53, 13.50. IR (cm^{-1}): 3403, 2935, 2844, 1687, 1508, 1348, 1260, 1212, 1073, 776. HRMS (M + Na)$^+$ = 277.1199 calculated for $C_{17}H_{18}NaO_2$; experimental = 277.1201.

(±)-*1-(isopropyl)-2-methoxy-3-methylcyclopent-2-enol* (23g)

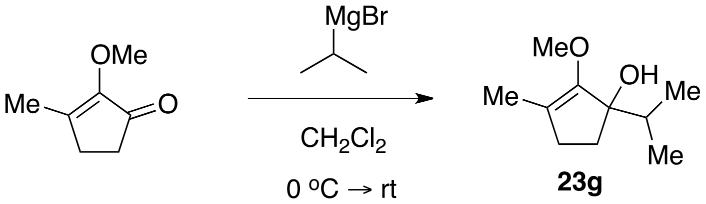

Crude 2-methoxy-3-methylcyclopent-2-enone (500 mg, 3.968 mmol) was dissolved in CH_2Cl_2 (10 mL) and cooled to 0ºC. Isopropylmagnesium bromide (6.0 mL, 2 M solution in THF) was then added dropwise, and the mixture was allowed to warm to room temperature. After stirring for 1 hour, the reaction was quenched with H_2O (15 mL). The aqueous layer was then extracted with EtOAc (3 x 20 mL). The combined organic layers were dried over Na_2SO_4 and then concentrated under vacuum. The crude material was purified with flash column chromatography with 92.5 : 7.5 hexanes : EtOAc to give product **23g** (218 mg, 32% yield) as a yellow oil.

^{1}H NMR (400 MHz, $CDCl_3$): δ (ppm) = 3.79 (s, 3H), 2.31 – 2.16 (m, 1H), 2.06 – 1.89 (m, 3H), 1.84 (s, 1H), 1.72 (s, 3H), 1.65 – 1.51 (m, 1H), 0.94 (d, J = 6.8 Hz, 3H), 0.78 (d, J = 6.9 Hz, 3H). ^{13}C NMR (100 MHz, $CDCl_3$): δ (ppm) = 153.24, 115.48, 87.32, 60.06, 34.47, 31.60, 29.80, 18.25, 16.51, 13.27. IR (cm^{-1}): 3453, 2956, 2847, 1686, 1466, 1382, 1327, 1266, 1124, 997, 932, 656. HRMS $(M + Na)^+$ = 193.1199 calculated for $C_{10}H_{18}NaO_2$; experimental = 193.1205.

(±)-*1-(isobutyl)-2-methoxy-3-methylcyclopent-2-enol* (23h)

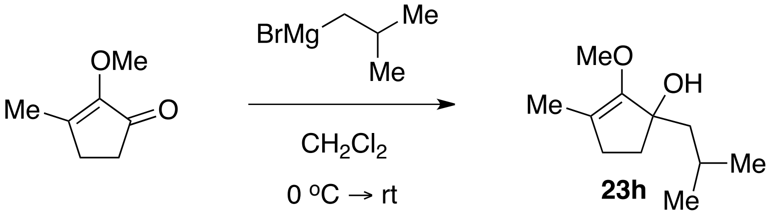

Crude 2-methoxy-3-methylcyclopent-2-enone (500 mg, 3.97 mmol) was dissolved in CH_2Cl_2 (10 mL) and cooled to 0ºC. Isobutylmagnesium bromide (3.9 mL, 2 M solution in diethyl ether) was then added dropwise, and the mixture was allowed to warm to room temperature. After stirring for 1 hour, the reaction was quenched with H_2O (15 mL). Reaction mixture was filtered through pad of celite. The aqueous layer was then extracted with CH_2Cl_2 (3 x 20 mL). The combined organic layers were dried over Na_2SO_4 and then concentrated under vacuum. The crude material was purified with flash column chromatography (buffered with 2% TEA) with 85 : 15 hexanes :diethyl ether to give product **23h** (324 mg, 44% yield) as colorless oil.

^{1}H NMR (500 MHz, $CDCl_3$): δ (ppm) = 3.79 (s, 3H), 2.28 – 2.19 (m, 1H), 2.10 – 2.01 (m, 2H), 1.85 – 1.77 (m, 2H), 1.71 (s, 3H), 1.62 (dd, J = 13.9, 5.6 Hz, 1H), 1.42 (dd, J = 13.9, 6.6 Hz, 1H), 0.94 (d, J = 6.7 Hz, 3H), 0.91 (d, J = 6.7 Hz, 3H). ^{13}C NMR (100 MHz, $CDCl_3$): δ (ppm) = 154.32, 114.56, 84.13, 60.01, 47.24, 34.87, 31.39, 24.96, 24.84, 24.18, 13.34. IR (cm^{-1}): 3445, 2952, 2847, 1685, 1462, 1384, 1325, 1258, 1213, 1045, 1001, 931. HRMS $(M + Na)^+$ = 207.1356 calculated for $C_{11}H_{20}NaO_2$; experimental = 207.1350.

(±)-*1-allyl-2-methoxy-3-phenylcyclopent-2-enol* (24a)

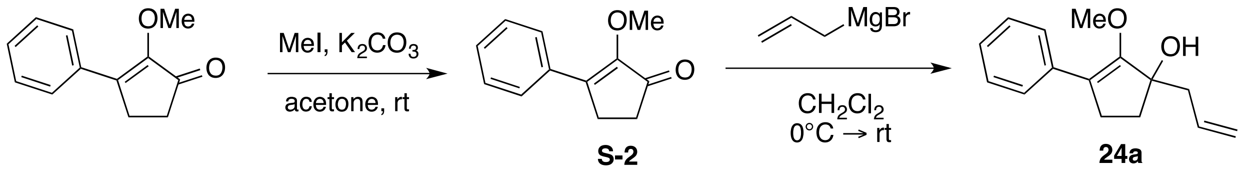

2-hydroxy-3-phenylcyclopent-2-enone[2] (104 mg, 0.59 mmol) was dissolved in anhydrous acetone (1.5 mL). K_2CO_3 (167 mg, 1.18 mmol) and then methyl iodide (75 μL, 1.18 mmol) were

[2] Jõgi, A.; Paju, A.; Pehk, T.; Kailas, T.; Müürisepp, A.-M.; Kanger, T.; Lopp, M. *Synthesis*, **2006**, *18*, 3031-3036

added. The reaction mixture was stirred at room temperature for 32 hours until the completion of reaction, as monitored by TLC. After concentrating the reaction mixture in *vacuo*, the crude residue was partitioned in EtOAc/H_2O (60 mL, 1:1). The aqueous layer extracted with EtOAc (3 x 25 mL). The combined organic layers were then washed with brine, dried over Na_2SO_4, and concentrated in *vacuo*. The crude material was purified with flash column chromatography with 85 : 15 hexanes : EtOAc to give product **S-2** (69 mg, 62% yield) as colorless oil.

^{1}H NMR (400 MHz, $CDCl_3$): δ (ppm) = 7.90 (dd, J = 8.2, 1.7 Hz, 2H), 7.49 – 7.37 (m, 3H), 4.06 (s, 3H), 3.19 – 2.72 (m, 2H), 2.70 – 2.40 (m, 2H). ^{13}C NMR (100 MHz, $CDCl_3$): δ (ppm) = 203.79, 152.47, 147.96, 134.18, 129.98, 128.70, 127.60, 58.33, 32.75, 23.84. IR (cm-1) : 2921, 2851, 1696, 1446, 1357, 1076, 971, 763, 691. HRMS $(M + H)^+$ = 189.091 calculated for $C_{12}H_{13}O_2$; experimental = 189.091.

Ketone **S-2** (60 mg, 0.32 mmol) was dissolved in CH_2Cl_2 (1.6 mL) and cooled to 0ºC. Allylmagnesium bromide (480 μL, 1 M solution in Et_2O) was then added dropwise, and the mixture was allowed to warm to room temperature. After stirring for 1 hour, the reaction was quenched with H_2O (15 mL). The aqueous layer was then extracted with EtOAc (3 x 20 mL). The combined organic layers were dried over Na_2SO_4 and then concentrated under vacuum. The crude material was purified with flash column chromatography with 90 : 10 hexanes : EtOAc to give product **24a** (67 mg, 91% yield) as colorless oil.

^{1}H NMR (500 MHz, $CDCl_3$): δ (ppm) = 7.51-7.49 (dd, J = 1.5 Hz, J = 1.2 Hz, 2H), 7.36-7.33 (ddd, J = 1.9 Hz, J = 1.4 Hz, J = 1.5 Hz, 2H), 7.26 – 7.22 (m, 1H), 5.90 (dddd, J = 17.0, 10.1, 7.8, 6.9 Hz, 1H), 5.24 – 5.14 (m, 2H), 3.70 (s, 3H), 2.70 (ddd, J = 15.0, 9.1, 3.6 Hz, 1H), 2.56 (dd, J = 13.8, 7.8 Hz, 1H), 2.52 – 2.43 (m, 2H), 2.20 (ddd, J = 13.4, 8.6, 3.6 Hz, 1H), 2.06 (s, 1H), 1.92 (ddd, J = 13.4, 9.1, 5.6 Hz, 1H). ^{13}C NMR (100 MHz, $CDCl_3$): δ (ppm) = 155.66,

136.28, 133.76, 128.26, 127.89, 127.01, 118.92, 118.61, 83.65, 60.09, 43.71, 34.28, 29.09. IR (cm-1): 3413, 3074, 2937, 2851, 1639, 1493, 1211, 1066, 913, 760, 731. HRMS $(M + Na)^+$ = 253.1199 calculated for $C_{15}H_{18}NaO_2$; experimental = 253.1210.

(±)-2-methoxy-1-octyl-3-phenylcyclopent-2-en-1-ol (24b)

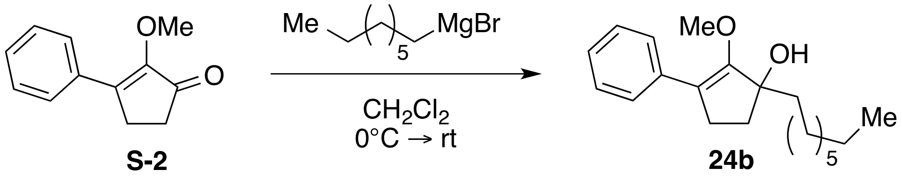

Ketone **S-2** (20 mg, 0.106 mmol) was dissolved in CH_2Cl_2 (0.5 mL) and cooled to 0ºC. Octylmagnesium bromide (106 μL, 2 M solution in Et_2O) was then added dropwise, and the mixture was allowed to warm to room temperature. After stirring for 1 hour, the reaction was quenched with H_2O (15 mL). The aqueous layer was then extracted with EtOAc (3 x 20 mL). The combined organic layers were dried over Na_2SO_4 and then concentrated under vacuum. The crude material was purified with flash column chromatography with 90 : 10 hexanes : EtOAc to give product **24b** (25 mg, 78% yield) as colorless oil.

^1H NMR (500 MHz, $CDCl_3$): δ (ppm) = 7.55 – 7.48 (m, 2H), 7.34 (t, J = 7.8 Hz, 2H), 7.23 (t, J = 7.4 Hz, 1H), 3.70 (s, 3H), 2.72 (ddd, J = 15.0, 9.1, 3.8 Hz, 1H), 2.49 (ddd, J = 15.0, 8.7, 5.4 Hz, 1H), 2.18 (ddd, J = 13.6, 8.7, 3.8 Hz, 1H), 1.94 (ddd, J = 13.9, 9.1, 5.3 Hz, 1H), 1.78 (ddd, J = 13.2, 11.2, 4.7 Hz, 1H), 1.70 (ddd, J = 12.8, 11.3, 3.9 Hz, 1H), 1.31 (ddd, J = 18.0, 8.8, 4.3 Hz, 12H), 0.89 (t, J = 6.9 Hz, 3H). ^{13}C NMR (125 MHz, $CDCl_3$): δ (ppm) = 156.04, 136.41, 128.23, 127.84, 126.89, 118.20, 84.76, 59.96, 39.16, 34.42, 32.09, 30.33, 29.78, 29.50, 29.14, 24.35, 22.87, 14.31. IR (cm-1): 3382, 3055, 3023, 2924, 2852, 1639, 1493, 1459,1444, 1260,

1068, 1016, 798, 760. HRMS $(M^*)^+$ = 302.2245 calculated for $C_{20}H_{30}O_2$; experimental = 302.2240.

(±)-*1-isopropyl-2-methoxy-3-phenylcyclopent-2-enol* (24c)

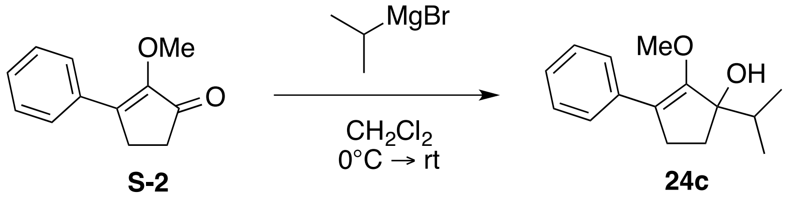

Ketone **S-2** (37 mg, 0.198 mmol) was dissolved in CH_2Cl_2 (1.0 mL) and cooled to 0ºC. Isopropylmagnesium bromide (492 µL, 2 M solution in THF) was then added dropwise, and the mixture was allowed to warm to room temperature. After stirring for 1 hour, the reaction was quenched with H_2O (15 mL). The aqueous layer was then extracted with EtOAc (3 x 20 mL). The combined organic layers were dried over Na_2SO_4 and then concentrated under vacuum. The crude material was purified with flash column chromatography with 90 : 10 hexanes : EtOAc to give product **24c** (28 mg, 61% yield) as colorless oil.

^{1}H NMR (400 MHz, $CDCl_3$): δ (ppm) = δ 7.49 (d, J = 7.7 Hz, 2H), 7.34 (t, J = 7.7 Hz, 2H), 7.23 (t, J = 7.4 Hz, 1H), 3.68 (s, 3H), 2.76 (ddd, J = 15.2, 9.3, 4.1 Hz, 1H), 2.44 (ddd, J = 15.0, 9.0, 4.8 Hz, 1H), 2.18 (ddd, J = 13.5, 9.0, 4.0 Hz, 1H), 2.11 (p, J = 6.8 Hz, 1H), 1.90 (s, 1H), 1.76 (ddd, J = 14.0, 9.3, 4.8 Hz, 1H), 1.05 (d, J = 6.9 Hz, 3H), 0.92 (d, J = 7.0 Hz, 3H). ^{13}C NMR (100 MHz, $CDCl_3$): δ (ppm) = 155.66, 136.56, 128.21, 127.94, 126.90, 118.49, 87.89, 60.17, 34.82, 29.74, 29.51, 18.33, 16.55. IR (cm-1): 3449, 3055, 3023, 2958, 2936, 2872, 2853, 1642, 1598, 1493, 1466, 1444, 1215, 1036, 1014, 798, 760, 694. HRMS $(M + Na)^+$ = 255.1356 calculated for $C_{15}H_{20}NaO_2$; experimental = 255.1350.

(±)-*1-(isobutyl)-2-methoxy-3-phenylcyclopent-2-enol* (**24d**)

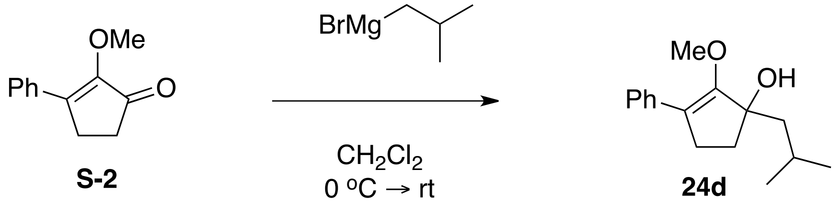

Crude 2-methoxy-3-phenylcyclopent-2-enone (100 mg, 0.532 mmol) was dissolved in CH_2Cl_2 (3 mL) and cooled to 0ºC. Isobutylmagnesium bromide (530 μL, 2 M solution in Et_2O) was then added dropwise, and the mixture was allowed to warm to room temperature. After stirring for 3.5 hours, the reaction was quenched with H_2O (15 mL). The aqueous layer was then extracted with EtOAc (3 x 20 mL). The combined organic layers were dried over Na_2SO_4 and then concentrated under vacuum. The crude material was purified with flash column chromatography with 92.5 : 7.5 hexanes : EtOAc to give product **24d** (93 mg, 71% yield) as a yellow oil.

^{1}H NMR (500 MHz, $CDCl_3$): δ (ppm) = 7.50 (d, J = 6.8 Hz, 2H), 7.34 (t, J = 7.7 Hz, 2H), 7.23 (t, J = 7.4 Hz, 1H), 3.69 (s, 3H), 2.75 (ddd, J = 15.1, 9.0, 3.9 Hz, 1H), 2.50 (ddd, J = 15.0, 8.6, 5.2 Hz, 1H), 2.23 (ddd, J = 13.5, 8.7, 3.9 Hz, 1H), 1.97 (ddd, J = 13.9, 9.0, 5.2 Hz, 1H), 1.90 – 1.79 (m, 2H), 1.76 (dd, J = 13.9, 5.6 Hz, 1H), 1.61 (dd, J = 13.9, 6.6 Hz, 1H), 1.03 (d, J = 6.7 Hz, 3H), 1.00 (d, J = 6.7 Hz, 3H). ^{13}C NMR (125 MHz, $CDCl_3$): δ (ppm) = 156.56, 136.54, 128.22, 127.87, 126.87, 117.54, 84.77, 60.06, 47.31, 34.68, 29.37, 25.04, 24.94, 24.26. IR (cm^{-1}): 3422, 3055, 2952, 2867, 1639, 1599, 1494, 1461, 1445, 1385, 1214, 1035, 760, 695. HRMS (M + Na)$^+$ = 269.1512 calculated for $C_{16}H_{22}NaO_2$; experimental = 269.1524.

(±)-*2-methoxy-3-phenylcyclopent-2-enol* (24e)

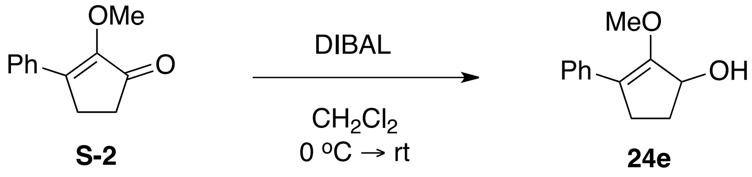

Ketone **S-2** (35 mg, 0.186 mmol) was dissolved in CH_2Cl_2 (1.0 mL) and cooled to 0ºC. DIBAL (279 μL, 1 M solution in toluene) was then added dropwise, and the mixture was allowed to warm to room temperature. After stirring for 1 hour, the reaction was quenched with H_2O (15 mL). The aqueous layer was then extracted with EtOAc (3 x 20 mL). The combined organic layers were dried over Na_2SO_4 and then concentrated under vacuum. The crude material was purified with flash column chromatography with 90 : 10 hexanes : EtOAc to give product **24e** (27 mg, 76% yield) as colorless oil.

^{1}H NMR (400 MHz, $CDCl_3$): δ (ppm) = 7.67 (dd, J = 8.2, 1.4 Hz, 2H), 7.34 (t, J = 7.8 Hz, 2H), 7.21 (t, J = 7.4 Hz, 1H), 5.01 (d, J = 7.6 Hz, 1H), 3.88 (s, 3H), 3.01 – 2.73 (m, 1H), 2.61 (ddd, J = 15.0, 8.9, 3.6 Hz, 1H), 2.50 – 2.21 (m, 1H), 1.9-1.81 (m, 2H). ^{13}C NMR (100 MHz, $CDCl_3$): δ (ppm) = 154.62, 135.69, 128.25, 127.19, 126.57, 116.10, 74.08, 56.80, 30.67, 28.50. IR (cm-1): 3327, 3052, 2922, 2850, 1737, 1640, 1597, 1353, 1234, 1154, 1123, 1038, 1005, 797, 758, 692. HRMS $(M + Na)^+$ = 213.0886 calculated for $C_{12}H_{14}NaO_2$; experimental = 213.0894.

(±)-*3-(2-methoxy-3-(4-methoxyphenyl)-1-methylcyclopent-2-enyl)-1H-indole* (26a)

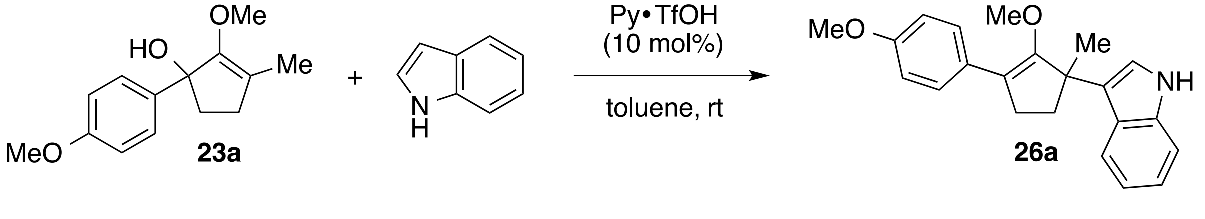

Compound **23a** (50 mg, 0.214 mmol) was dissolved in toluene (1.0 mL). Indole (50 mg, 0.427 mmol) and then pyridinium triflate (5 mg, 0.021 mmol) were added. Upon stirring at room temperature for 1 hour, the reaction mixture was concentrated under vacuum and then directly purified with flash column chromatography with 90 : 10 hexanes : Et_2O to give product **26a** (64 mg, 90% yield) as light green solid.

^{1}H NMR (500 MHz, $CDCl_3$): δ (ppm) = 7.95 (s, 1H), 7.80 (dd, J = 8.0, 1.2 Hz, 1H), 7.62 (d, J = 8.8 Hz, 2H), 7.39 – 7.33 (m, 1H), 7.21 (ddd, J = 8.3, 7.1, 1.2 Hz, 1H), 7.13 – 7.04 (m, 2H), 6.97 (d, J = 8.8 Hz, 2H), 3.88 (s, 3H), 3.46 (s, 3H), 2.94 – 2.74 (m, 2H), 2.54 (ddd, J = 12.8, 9.1, 7.0 Hz, 1H), 2.07 (ddd, J = 12.6, 8.7, 3.8 Hz, 1H), 1.77 (s, 3H). ^{13}C NMR (125 MHz, $CDCl_3$): δ (ppm) = 158.75, 158.03, 137.23, 129.75, 128.68, 126.41, 123.47, 121.90, 120.83, 120.77, 119.35, 115.33, 113.71, 111.38, 58.99, 55.46, 48.13, 37.48, 29.95, 25.19. IR (cm^{-1}): 3411, 3056, 2958, 2932, 2837, 1606, 1509, 1455, 1243, 1177, 1098, 1030, 831, 796, 733. HRMS $(M+Na)^+$ = 356.1621 calculated for $C_{22}H_{23}NNaO_2$; experimental = 356.1622.

(±)-*3-(2-methoxy-1-methyl-3-p-tolylcyclopent-2-enyl)-1H-indole* (26b)

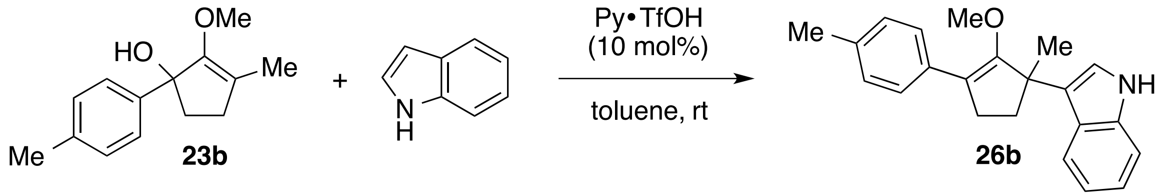

Compound **23b** (79 mg, 0.366 mmol) was dissolved in toluene (1.8 mL). Indole (85 mg, 0.732 mmol) and then pyridinium triflate (8 mg, 0.036 mmol) were added. Upon stirring at room temperature for 1 hour, the reaction mixture was concentrated under vacuum and then directly purified with flash column chromatography with 90 : 10 hexanes : Et_2O to give product **26b** (98 mg, 84% yield) as white solid.

^{1}H NMR (400 MHz, $CDCl_3$): δ (ppm) = 7.96 (s, 1H), 7.80 (dd, J = 8.0, 1.2 Hz, 1H), 7.56 (d, J = 8.1 Hz, 2H), 7.36 (d, J = 8.3 Hz, 1H), 7.27 – 7.15 (m, 3H), 7.16 – 7.08 (m, 1H), 7.07 (d, J = 2.2 Hz, 1H), 3.46 (s, 3H), 2.89 (ddd, J = 15.3, 8.6, 7.0 Hz, 1H), 2.79 (ddd, J = 14.7, 9.2, 3.7 Hz, 1H), 2.60 – 2.45 (m, 1H), 2.40 (s, 3H), 2.06 (ddd, J = 12.6, 8.6, 3.7 Hz, 1H), 1.76 (s, 3H). ^{13}C NMR (100 MHz, $CDCl_3$): δ (ppm) = 159.53, 137.24, 135.87, 134.25, 128.95, 127.50, 126.42, 123.47, 121.92, 120.85, 120.75, 119.37, 115.48, 111.37, 59.11, 48.24, 37.52, 30.02, 25.11, 21.40. IR (cm^{-1}): 3413, 2959, 2930, 2846, 1665, 1631, 1455, 1335, 1127, 816, 735, 531. HRMS $(M + Na)^+$ = 340.1672 calculated for $C_{22}H_{23}NNaO$; experimental = 340.1669.

(±)-*3-(3-(4-fluorophenyl)-2-methoxy-1-methylcyclopent-2-enyl)-1H-indole* (26c)

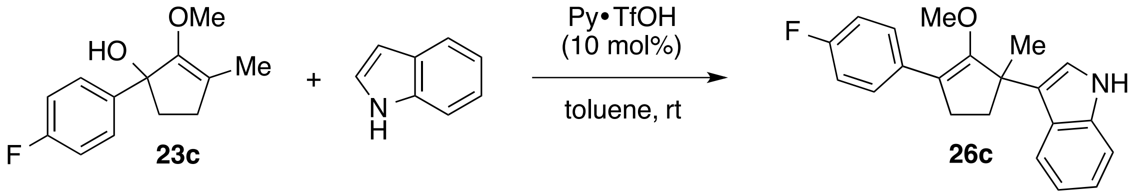

Compound **23c** (50 mg, 0.225 mmol) was dissolved in toluene (1.1 mL). Indole (53 mg, 0.450 mmol) and then pyridinium triflate (5 mg, 0.023 mmol) were added. Upon stirring at room temperature for 1 hour, the reaction mixture was concentrated under vacuum and then directly purified with flash column chromatography with 90 : 10 hexanes : Et_2O to give product **26c** (62 mg, 88% yield) as light green solid.

^{1}H NMR (500 MHz, $CDCl_3$): δ (ppm) = 7.94 (s, 1H), 7.78 (d, J = 8.1 Hz, 1H), 7.70 – 7.59 (m, 2H), 7.37 (d, J = 8.1 Hz, 1H), 7.22 (t, J = 7.5 Hz, 1H), 7.14 – 7.03 (m, 4H), 3.45 (s, 3H), 2.88 (dt, J = 15.3, 8.0 Hz, 1H), 2.78 (td, J = 11.0, 10.1, 4.7 Hz, 1H), 2.55 (dt, J = 15.8, 8.4 Hz, 1H), 2.13 – 1.98 (m, 1H), 1.77 (s, 3H). ^{13}C NMR (125 MHz, $CDCl_3$): δ (ppm) = 162.28, 160.33, 159.76 (d, J = 1.7 Hz), 137.24, 133.19, 133.17, 129.04, 128.98, 126.36, 123.30, 122.02,

120.74, 120.72, 119.46, 115.13, 114.97, 114.54, 111.43, 58.84, 48.13, 37.69, 29.92, 25.13. IR (cm^{-1}): 3411, 3055, 2960, 2927, 2848, 1633, 1506, 1262, 1220, 1092, 1012, 835, 805, 737. HRMS $(M + Na)^+$ = 344.1421 calculated for $C_{21}H_{20}FNNaO$; experimental =344.1419.

(±)-*3-(3-(4-chlorophenyl)-2-methoxy-1-methylcyclopent-2-enyl)-1H-indole* (**26d**)

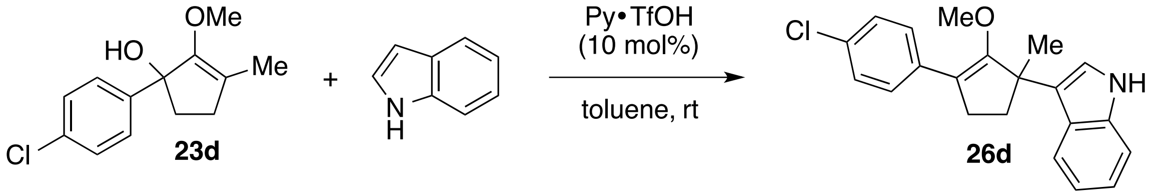

Compound **23d** (50 mg, 0.209 mmol) was dissolved in toluene (1.1 mL). Indole (49 mg, 0.418 mmol) and then pyridinium triflate (5 mg, 0.021 mmol) were added. Upon stirring at room temperature for 1 hour, the reaction mixture was concentrated under vacuum and then directly purified with flash column chromatography with 90 : 10 hexanes : Et_2O to give product **26d** (64 mg, 90% yield) as light green solid.

^{1}H NMR (400 MHz, $CDCl_3$): δ (ppm) = 7.95 (s, 1H), 7.75 (d, J = 8.0 Hz, 1H), 7.59 (d, J = 8.2 Hz, 2H), 7.36 (t, J = 8.9 Hz, 3H), 7.21 (t, J = 7.6 Hz, 1H), 7.16 – 6.99 (m, 2H), 3.45 (s, 3H), 2.86 (dt, J = 15.4, 7.9 Hz, 1H), 2.80 – 2.69 (m, 1H), 2.62 – 2.46 (m, 1H), 2.05 (ddd, J = 12.5, 8.5, 3.5 Hz, 1H), 1.76 (s, 3H). ^{13}C NMR (100 MHz, $CDCl_3$): δ (ppm) = 160.68, 137.22, 135.55, 131.58, 128.72, 128.35, 126.31, 123.17, 122.06, 120.70, 120.68, 119.50, 114.34, 111.43, 58.78, 48.17, 37.80, 29.63, 25.06. IR (cm^{-1}): 3411, 3057, 2961, 2937, 2845, 1629, 1544, 1489, 1260, 1091, 906, 829, 765, 728, 530. HRMS $(M + Na)^+$ = 360.1126 calculated for $C_{21}H_{20}ClNNaO$; experimental =360.1129.

(±)-*3-(3-(4-fluorophenyl)-2-methoxy-1-methylcyclopent-2-enyl)-1H-indole* (26e)

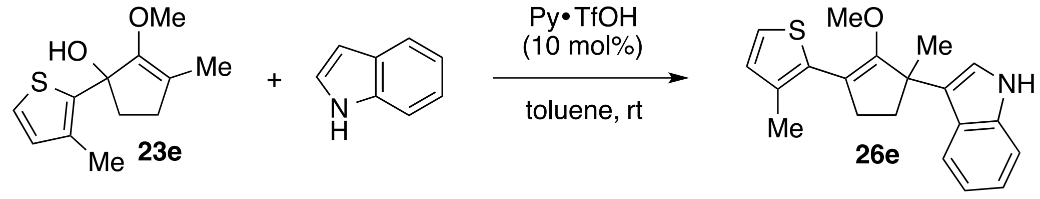

Compound **23e** (50 mg, 0.223 mmol) was dissolved in toluene (1.1 mL). Indole (53 mg, 0.446 mmol) and then pyridinium triflate (5 mg, 0.022 mmol) were added. Upon stirring at room temperature for 1 hour, the reaction mixture was concentrated under vacuum and then directly purified with flash column chromatography with 90 : 10 hexanes : Et_2O to give product **26e** (67 mg, 93% yield) as white solid.

^{1}H NMR (500 MHz, $CDCl_3$): δ (ppm) = 7.93 (bs, 1H), 7.83 (d, J = 8.0 Hz, 1H), 7.37 (d, J = 8.0 Hz, 1H), 7.23 – 7.16 (m, 2H), 7.11 (dd, J = 8.0, 8.0 Hz, 1H), 7.08 (d, J = 2.1 Hz, 1H), 6.84 (d, J = 5.1 Hz, 1H), 3.42 (s, 3H), 2.84 – 2.68 (m, 2H), 2.55 (dt, J = 13.0, 8.0 Hz, 1H), 2.27 (s, 3H), 2.05 (ddd, J = 13.0, 8.0, 3.8 Hz, 1H), 1.71 (s, 3H). ^{13}C NMR (125 MHz, $CDCl_3$): δ (ppm) = 160.86, 137.25, 134.80, 134.55, 129.88, 126.30, 123.55, 122.98, 121.95, 120.93, 120.72, 119.26, 111.46, 105.94, 58.73, 48.31, 37.21, 33.50, 25.23, 15.06. IR (cm^{-1}): 3350, 2958, 2923, 2853, 1456, 1262, 1104, 1083, 1014, 798, 737, 712. HRMS $(M + H)^+$ = 324.1417 calculated for $C_{20}H_{22}NOS$; experimental = 324.1414.

(±)-*3-(2-methoxy-1-methyl-3-(naphthalen-1-yl)cyclopent-2-enyl)-1H-indole* (26f)

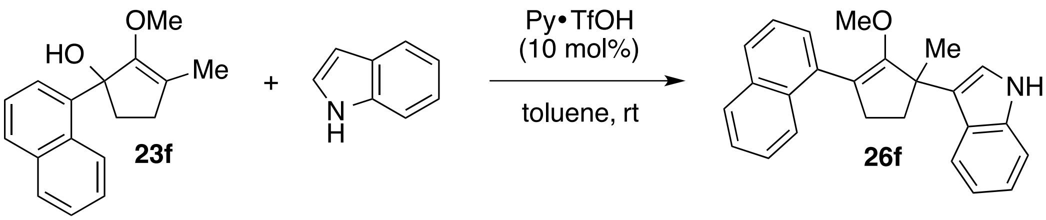

Compound **23f** (60 mg, 0.236 mmol) was dissolved in toluene (1.2 mL). Indole (55 mg, 0.472 mmol) and then pyridinium triflate (5 mg, 0.024 mmol) were added. Upon stirring at room temperature for 1 hour, the reaction mixture was concentrated under vacuum and then directly purified with flash column chromatography with 85 : 15 hexanes : Et_2O to give product **26f** (26 mg, 31% yield) as white solid.

^{1}H NMR (500 MHz, $CDCl_3$): δ (ppm) = 8.15 (d, J = 8.2 Hz, 1H), 8.00 (d, J = 8.0 Hz, 2H), 7.87 (d, J = 7.3 Hz, 1H), 7.78 (d, J = 8.1 Hz, 1H), 7.57 – 7.45 (m, 4H), 7.41 (d, J = 8.1 Hz, 1H), 7.22 (t, J = 7.2 Hz, 1H), 7.20 – 7.13 (m, 2H), 3.16 (s, 3H), 2.88 (ddd, J = 15.0, 9.0, 6.5 Hz, 1H), 2.78 (ddd, J = 15.0, 9.0, 4.0 Hz, 1H), 2.67 (ddd, J = 12.7, 9.0, 6.5 Hz, 1H), 2.15 (ddd, J = 12.7, 9.0, 4.0 Hz, 1H), 1.81 (s, 3H). ^{13}C NMR (125 MHz, $CDCl_3$): δ (ppm) = 159.60, 137.33, 137.17, 133.75, 132.74, 128.54, 127.12, 127.00, 126.49, 126.45, 126.23, 125.92, 125.48, 123.30, 121.95, 121.01, 120.80, 119.29, 111.54, 108.81, 59.08, 48.94, 37.46, 34.92, 25.49. IR (cm^{-1}): 2961, 2922, 1268, 1085, 1012, 792. HRMS $(M + H)^+$ = 354.1852 calculated for $C_{25}H_{24}NO$; experimental = 354.1846.

(±)-*3-(1-allyl-2-methoxy-3-phenylcyclopent-2-enyl)-1H-indole* (**26g**)

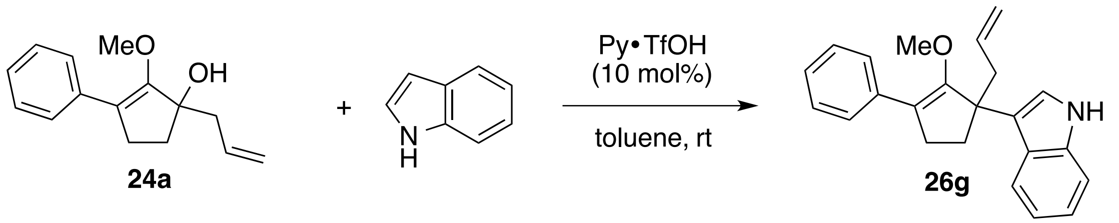

Compound **24a** (50 mg, 0.220 mmol) was dissolved in toluene (1.0 mL). Indole (51 mg, 0.435 mmol) and then pyridinium triflate (5 mg, 0.022 mmol) were added. Upon stirring at room temperature for 1 hour, the reaction mixture was concentrated under vacuum and then directly

purified with flash column chromatography with 85 : 15 hexanes : Et_2O to give product **26g** (72 mg, 80% yield) as white solid.

^{1}H NMR (400 MHz, $CDCl_3$): δ (ppm) = 7.99 (s, 1H), 7.83 (dd, J = 8.0, 1.2 Hz, 1H), 7.61 (d, J = 6.9 Hz, 1H), 7.40 (t, J = 7.8 Hz, 3H), 7.32 – 7.18 (m, 2H), 7.18 – 7.07 (m, 2H), 6.05 (dddd, J = 16.6, 10.2, 8.0, 6.2 Hz, 1H), 5.27 (dd, J = 17.0, 1.9 Hz, 1H), 5.16 (dd, J = 10.2, 1.2 Hz, 1H), 3.44 (s, 3H), 2.99 (dd, J = 13.6, 6.3 Hz, 1H), 2.92 – 2.79 (m, 3H), 2.48 (ddd, J = 13.3, 9.3, 6.5 Hz, 1H), 2.29 (ddd, J = 13.3, 8.6, 4.8 Hz, 1H). ^{13}C NMR (100 MHz, $CDCl_3$): δ (ppm) 157.83, 137.25, 137.14, 135.66, 128.21, 127.94, 126.50, 126.39, 123.08, 122.02, 120.86, 120.80, 119.45, 117.54, 116.72, 111.41, 59.49, 52.22, 42.54, 33.21, 31.31, 29.92. IR (cm^{-1}): 3414, 3072, 2921, 2850, 1736, 1638, 1491, 1260, 1013, 800, 763, 661. HRMS $(M + H)^+$ = 330.1852 calculated for $C_{23}H_{24}NO$; experimental = 330.1843.

(±)-*3-(2-methoxy-1-octyl-3-phenylcyclopent-2-enyl)-1H-indole* (**26h**)

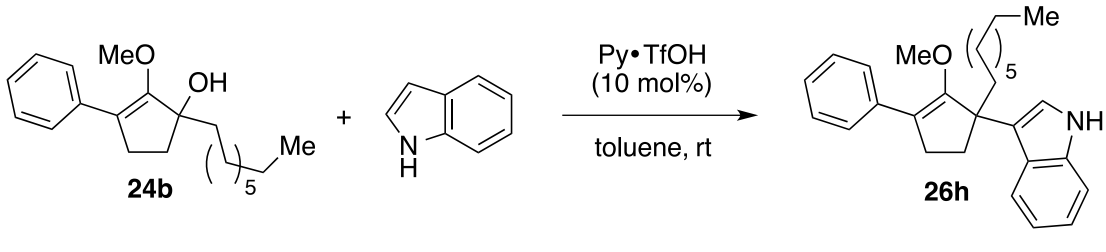

Compound **24b** (23 mg, 0.076 mmol) was dissolved in toluene (0.4 mL). Indole (18 mg, 0.152 mmol) and then pyridinium triflate (2 mg, 0.009 mmol) were added. Upon stirring at room temperature for 18 hours, the reaction mixture was concentrated under vacuum and then directly purified with flash column chromatography with 85 : 15 hexanes : Et_2O to give product **26h** (25 mg, 80% yield) as white solid.

^{1}H NMR (500 MHz, $CDCl_3$): δ (ppm) = 7.95 (s, 1H), 7.79 (d, J = 8.0 Hz, 1H), 7.58 (d, J = 7.2 Hz, 2H), 7.40 – 7.33 (m, 3H), 7.22 (dd, J = 7.4, 7.4 Hz, 1H), 7.17 (dd, J = 7.2, 7.2 Hz, 1H),

7.12 (d, J = 2.4 Hz, 1H), 7.06 (dd, J = 7.5, 7.5 Hz, 1H), 3.38 (s, 3H), 2.81 (qdd, J = 15.0, 9.3, 5.3 Hz, 2H), 2.45 (ddd, J = 13.4, 9.6, 6.2 Hz, 1H), 2.18 (ddd, J = 13.5, 9.1, 4.5 Hz, 1H), 2.10 (ddd, J = 9.6, 6.6, 3.0 Hz, 2H), 1.54 – 1.29 (m, 12H), 0.89 (t, J = 7.0 Hz, 3H). ^{13}C NMR (125 MHz, $CDCl_3$): δ (ppm) = 158.26, 137.37, 137.18, 128.21, 127.85, 126.62, 126.25, 123.88, 121.93, 120.96, 120.61, 119.38, 116.32, 111.34, 59.23, 52.30, 38.48, 33.99, 32.13, 31.37, 30.70, 29.92, 29.64, 24.60, 22.90, 14.34. IR (cm^{-1}): 3417, 3054, 2955, 2921, 2851, 1492, 1258, 1090, 1013, 865, 795, 740, 698. HRMS $(M + Na)^+$ = 424.2611 calculated for $C_{28}H_{35}NNaO$; experimental = 424.2613.

(±)-*3-(1-isopropyl-2-methoxy-3-phenylcyclopent-2-enyl)-1H-indole* (**26i**)

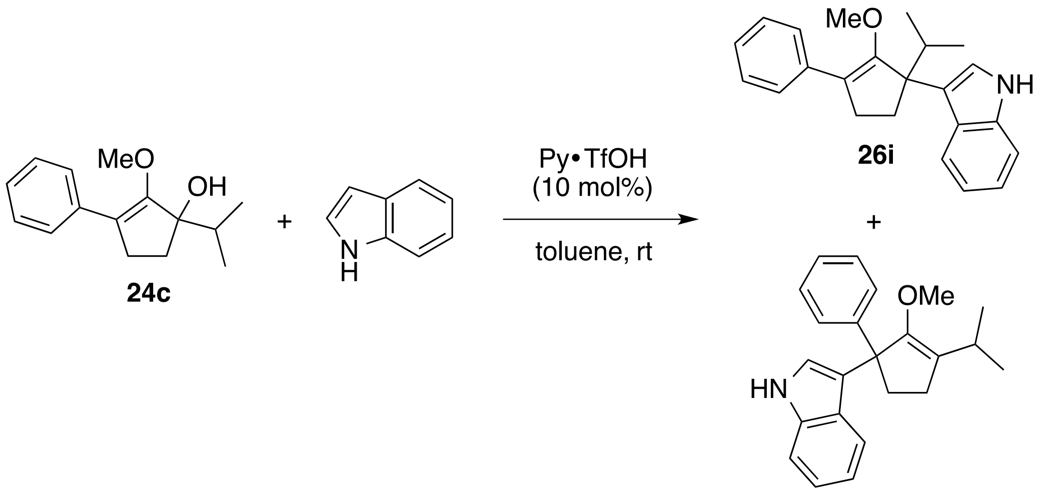

Compound **24c** (20 mg, 0.086 mmol) was dissolved in toluene (0.4 mL). Indole (20 mg, 0.173 mmol) and then pyridinium triflate (2 mg, 0.009 mmol) were added. Upon stirring at room temperature for 26 hours, the reaction mixture was concentrated under vacuum and then directly purified with flash column chromatography with 85 : 15 hexanes : Et_2O to give product **26i** as an inseparable mixture of regioisomers (13 mg, 46% yield) as white solid.

^{1}H NMR (500 MHz, $CDCl_3$, * denotes the minor regioisomer): δ (ppm) = 7.96 (bs, 1H, 1H*), 7.91 (d, J = 8.0 Hz, 1H), 7.47 (d, J = 8.4 Hz, 1H, 1H*), 7.41 (d, J = 8.1 Hz, 1H, 1H*), 7.35 (dd, J = 10.5, 8.1 Hz, 2H, 2H*), 7.33 – 7.28 (m, 2H, 2H*), 7.24 – 7.14 (m, 3H, 2H*), 7.11 – 7.10 (m, 1H, 1H*), 6.98 (dd, J = 7.6 Hz, 1H), 3.32 (s, 3H*), 3.23 (s, 3H), 3.03 (p, J = 6.9 Hz, 1H), 2.97 – 2.88 (m, 1H), 2.80 (p, J = 6.8 Hz, 1H*), 2.74 – 2.65 (m, 1H, 1H*), 2.61 (ddd, J = 13.0, 8.2, 4.9 Hz, 1H), 2.43 – 2.22 (m, 2H, 2H*), 1.13 (dd, J = 9.6, 7.3 Hz, 3H, 3H*), 1.07 (d, J = 6.6 Hz, 3H*), 0.98 (d, J = 6.5 Hz, 3H). ^{13}C NMR (125 MHz, $CDCl_3$, * denotes the minor regioisomer): δ (ppm) = 159.10*, 155.04, 147.34, 137.73*, 137.17, 137.13*, 128.07, 128.06*, 128.03*, 127.98, 127.90, 126.96, 126.79*, 126.21*, 126.00, 122.77, 122.13*, 121.90*, 121.86, 121.77, 121.75*, 121.70, 121.53*, 119.17*, 119.11, 115.42* , 111.27*, 111.21, 61.12, 59.89*, 56.98, 56.01*, 38.19, 32.93, 32.06*, 29.92, 28.03*, 25.99*, 24.89, 21.46*, 21.40, 18.67*, 18.49. HRMS $(M + H)^+$ = 354.1828 calculated for $C_{23}H_{25}NNaO$; experimental =354.1834.

(±)-3-(3-isobutyl-2-methoxy-1-phenylcyclopent-2-en-1-yl)-1H-indole (**26j**)

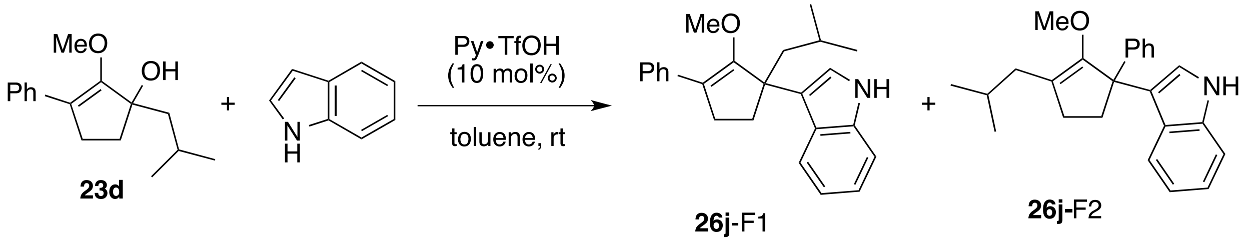

Compound **23d** (93 mg, 0.378 mmol) was dissolved in toluene (1.9 mL). Indole (88 mg, 0.756 mmol) and then pyridinium triflate (8.7 mg, 0.038 mmol) were added. Upon stirring at room temperature for 22 hours, the reaction mixture was concentrated under vacuum and then directly purified with flash column chromatography with 75 : 25 hexanes : CH_2Cl_2 to give product **26j** as a 1:1 mixture of regioisomers (85 mg, 65 % yield) as brown oil.

^{1}H NMR (500 MHz, $CDCl_3$, **26j**-F1): δ (ppm) = 7.93 (bs, 1H), 7.85 (d, J = 8.0 Hz, 1H), 7.57 (d, J = 7.4 Hz, 2H), 7.38 – 7.34 (m, 3H), 7.27 – 7.15 (m, 2H), 7.10 (d, J = 2.6 Hz, 1H), 7.09 (dd, J = 7.3, 0.9 Hz, 1H), 3.35 (s, 3H), 2.92 (ddd, J = 14.5, 9.5, 4.7 Hz, 1H), 2.79 (ddd, J = 14.8, 9.3, 5.5 Hz, 1H), 2.52 (ddd, J = 13.4, 9.5, 5.4 Hz, 1H), 2.25 (ddd, J = 13.7, 9.3, 4.7 Hz, 1H), 2.17 (dd, J = 13.7, 4.3 Hz, 1H), 2.02 (dd, J = 13.7, 6.7 Hz, 1H), 1.89 (m, 1H), 1.04 (d, J = 2.3 Hz, 3H), 1.03 (d, J = 2.2 Hz, 3H). ^{13}C NMR (125 MHz, $CDCl_3$): δ (ppm) = 158.60, 137.58, 137.16, 128.17, 127.92, 126.61, 126.22, 123.80, 121.87, 121.19, 120.52, 119.31, 115.45, 111.31, 59.34, 52.43, 46.88, 34.19, 31.70, 25.90, 24.95, 24.62. IR (cm^{-1}): 3416, 3055, 2952, 2866, 1637, 1598, 1456, 1338, 1208, 1099, 1065, 906, 801, 763, 729, 697, 647. HRMS $(M + Na)^+$ = 368.1985 calculated for $C_{24}H_{27}NNaO$; experimental = 368.1992.

^{1}H NMR (500 MHz, $CDCl_3$, **26j**-F2): δ (ppm) = 7.95 (bs, 1H), 7.44 – 7.38 (m, 2H), 7.36 (dd, J = 8.1, 1.0 Hz, 1H), 7.34 – 7.24 (m, 3H), 7.24 – 7.17 (m, 1H), 7.16 (ddd, J = 8.2, 7.0, 1.1 Hz, 1H), 7.12 (d, J = 2.4 Hz, 1H), 6.98 (ddd, J = 8.1, 7.1, 1.0 Hz, 1H), 3.27 (s, 3H), 2.72 (ddd, J = 13.0, 8.8, 5.8 Hz, 1H), 2.64 (ddd, J = 13.1, 8.4, 5.0 Hz, 1H), 2.46 – 2.36 (m, 1H), 2.32 (ddd, J = 15.2, 8.9, 5.0 Hz, 1H), 2.22 – 2.11 (m, 2H), 1.88 (m, 1H), 0.99 (t, J = 6.5 Hz, 6H).

^{13}C NMR (125 MHz, $CDCl_3$): δ (ppm) = 157.39, 147.54, 137.13, 128.02, 128.00, 126.93, 125.98, 122.81, 122.25, 121.88, 121.76, 120.50, 119.11, 111.22, 60.71, 56.26, 38.68, 36.93, 30.10, 27.07, 23.06.

(±)-*3-(2-methoxy-3-phenylcyclopent-2-enyl)-1H-indole* (26k)

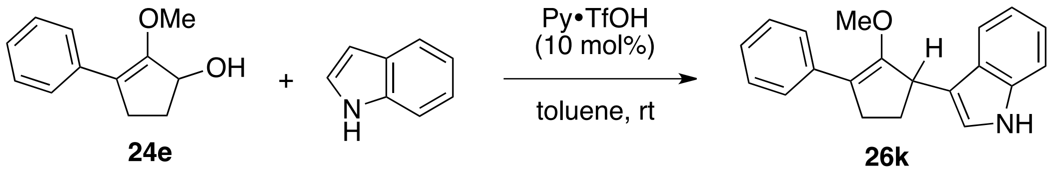

Compound **24e** (27 mg, 0.143 mmol) was dissolved in toluene (0.7 mL). Indole (34 mg, 0.286 mmol) and then pyridinium triflate (3 mg, 0.014 mmol) were added. Upon stirring at room temperature for 3 hours, the reaction mixture was concentrated under vacuum and then directly purified with flash column chromatography with 85 : 15 hexanes : Et_2O to give product **26k** (41 mg, 99% yield) as white solid.

^{1}H NMR (400 MHz, $CDCl_3$): δ (ppm) = 7.97 (s, 1H), 7.76 (d, J = 7.1 Hz, 2H), 7.69 (d, J = 7.9 Hz, 1H), 7.38 (t, J = 7.8 Hz, 3H), 7.27 – 7.11 (m, 3H), 7.06 (d, J = 2.4 Hz, 1H), 4.53 (d, J = 9.2 Hz, 1H), 3.65 (s, 3H), 2.91 – 2.65 (m, 2H), 2.49 (dq, J = 12.5, 8.7 Hz, 1H), 2.10 – 1.90 (m, 1H).^{13}C NMR (125 MHz, $CDCl_3$): δ (ppm) = 156.18, 136.86, 136.64, 128.26, 126.91, 126.76, 125.71, 122.28, 121.59, 119.64, 119.13, 118.24, 113.69, 111.40, 56.67, 39.88, 30.10, 29.74. IR (cm^{-1}): 3416, 3053, 2957, 2923, 2852, 1637, 1456, 1230, 1008, 796, 764, 695. HRMS $(M + H)^+$ = 290.1539 calculated for $C_{20}H_{20}NO$; experimental = 290.1542.

***(±)-3-(3-isopropyl-2-methoxy-1-methylcyclopent-2-en-1-yl)-1H-indole* (26l)**

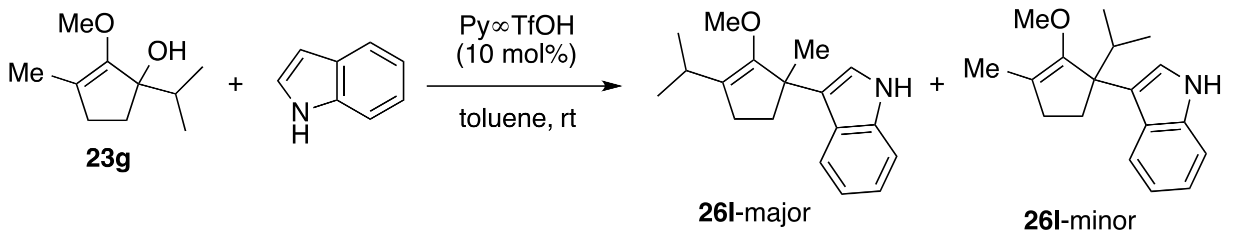

23g **26l**-major **26l**-minor

Compound **23g** (100 mg, 0.588 mmol) was dissolved in toluene (3.0 mL). Indole (138 mg, 1.176 mmol) and then pyridinium triflate (13 mg, 0.059 mmol) was added. Upon stirring at room temperature for 45 minutes, the reaction mixture was concentrated under vacuum and then directly purified with flash column chromatography with 90 : 10 hexanes : Et_2O to give product **26l-**major (87 mg, 55% yield) as a purple solid and **26l-**minor (39 mg, 25% yield) as brown oil.

^{1}H NMR (400 MHz, $CDCl_3$, **26l-**major): δ (ppm) = 7.91 (s, 1H), 7.76 (d, J = 8.0 Hz, 1H), 7.33 (d, J = 8.1 Hz, 1H), 7.20 (t, J = 7.6 Hz, 1H), 7.12 (t, J = 7.5 Hz, 1H), 6.97 (d, J = 2.5 Hz, 1H), 3.47 (s, 3H), 3.02 (p, J = 6.9 Hz, 1H), 2.54 – 2.29 (m, 3H), 2.03 – 1.87 (m, 1H), 1.65 (s, 3H), 1.20 (d, J = 6.9 Hz, 3H), 1.13 (d, J = 6.9 Hz, 3H). ^{13}C NMR (100 MHz, CDCl3): δ (ppm) = 155.48, 137.24, 126.55, 125.38, 123.66, 121.70, 120.87, 119.04, 111.37, 60.40, 46.99, 37.33, 25.88, 25.67, 24.49, 21.43, 21.36. IR (cm^{-1}): 3412, 3057, 2958, 2867, 2846, 1670, 1457, 1334, 1259, 1127, 1099, 800, 766. HRMS $(M+H)^+$ = 269.178 calculated for $C_{18}H_{24}NO$; experimental =269.1780.

^{1}H NMR (500 MHz, $CDCl_3$, **26l-**minor): δ (ppm) = 7.89 (s, 1H), 7.86 (dd, J = 8.0, 1.2 Hz, 1H), 7.33 (d, J = 8.1 Hz, 1H), 7.18 (t, J = 6.9 Hz, 1H), 7.13 – 7.05 (m, 2H), 3.59 (s, 3H), 2.64 (hept, J = 6.8 Hz, 1H), 2.49 – 2.37 (m, 1H), 2.33 – 2.19 (m, 2H), 2.14 (ddd, J = 13.5, 9.7, 6.0 Hz, 1H), 1.79 (s, 3H), 0.97 (d, J = 6.9 Hz, 3H), 0.91 (d, J = 6.9 Hz, 3H). ^{13}C NMR (125 MHz, CDCl3): δ (ppm) = 155.94, 137.11, 126.81, 122.59, 121.79, 121.54, 121.37, 118.94, 111.66, 111.21, 59.73, 56.02, 33.71, 32.77, 28.62, 18.60, 18.49, 13.65.

(±)-3-(3-isobutyl-2-methoxy-1-methylcyclopent-2-en-1-yl)-1H-indole (**26m**)

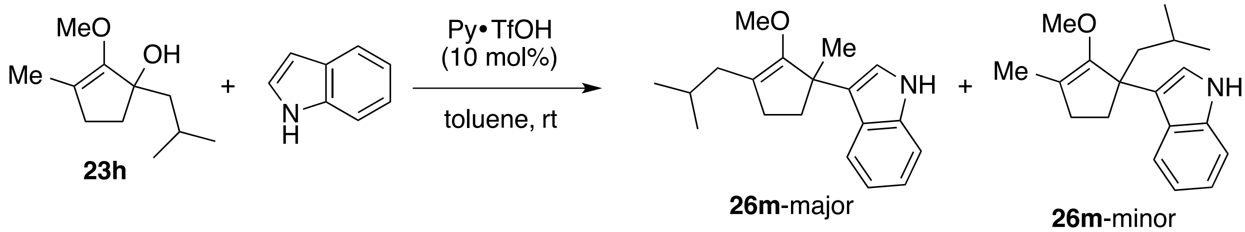

Compound **23h** (100 mg, 0.543 mmol) was dissolved in toluene (1.4 mL). Indole (128 mg, 1.086 mmol) and then pyridinium triflate (12.4 mg, 0.054 mmol) were added. Upon stirring at room temperature for 1 hour, the reaction mixture was concentrated under vacuum and then

directly purified with flash column chromatography with 75 : 25 hexanes : CH_2Cl_2 to give product **26m** as an mixture of regioisomers (121 mg, 78 % yield) as colorless oil.

^{1}H NMR (500 MHz, $CDCl_3$, **26m**-major): δ (ppm) = 7.90 (bs, 1H), 7.76 (d, J = 8.0 Hz, 1H), 7.34 (d, J = 8.1 Hz, 1H), 7.18 (dd, J = 7.3, 7.3 Hz, 1H), 7.09 (dd, J = 7.2, 7.2 Hz, 1H), 7.02 (d, J = 2.4 Hz, 1H), 3.47 (s, 3H), 2.45 (ddd, J = 12.4, 9.0, 6.5 Hz, 1H), 2.40 – 2.32 (m, 2H), 2.19 – 2.08 (m, 2H), 1.98 – 1.83 (m, 2H), 1.63 (s, 3H), 1.00 (d, J = 6.6 Hz, 3H), 0.98 (d, J = 6.6 Hz, 3H). ^{13}C NMR (125 MHz, $CDCl_3$): δ (ppm) = 157.98, 137.24, 126.55, 123.77, 121.76, 120.94, 120.89, 119.08, 118.06, 111.34, 59.98, 47.18, 37.60, 36.91, 29.70, 27.05, 25.82, 23.15, 22.97. IR (cm^{-1}): 3411, 2954, 2868, 1670, 1457, 1417, 1335, 1261, 1242, 1174, 1099, 1012, 909, 802, 736. HRMS $(M + Na)^+$ = 306.1828 calculated for $C_{19}H_{25}NNaO$; experimental = 306.1839.

^{1}H NMR (500 MHz, $CDCl_3$, **26m**-minor): δ (ppm) = 7.87 (bs, 1H), 7.77 (d, J = 8.0 Hz, 1H), 7.33 (d, J = 8.1 Hz, 1H), 7.16 (dd, J = 7.5, 7.5 Hz, 1H), 7.07 (dd, J = 7.5, 7.5 Hz, 1H), 7.04 (d, J = 2.4 Hz, 1H), 3.55 (s, 3H), 2.46 – 2.27 (m, 3H), 2.17 – 2.08 (m, 1H), 2.05 (dd, J = 13.6, 4.3 Hz, 1H), 1.80 (s, 3H), 1.77 – 1.69 (m, 1H), 0.94 (d, J = 3.0 Hz, 3H), 0.93 (d, J = 2.9 Hz, 3H). ^{13}C NMR (125 MHz, $CDCl_3$): δ (ppm) = 155.82, 137.17, 126.72, 124.13, 121.67, 121.34, 120.45, 119.05, 111.97, 111.22, 59.57, 51.55, 46.77, 34.44, 33.62, 25.78, 24.99, 24.50, 13.63.

(±)-*2-(2-methoxy-1-methyl-3-phenylcyclopent-2-en-1-yl)-1H-pyrrole* (27a)

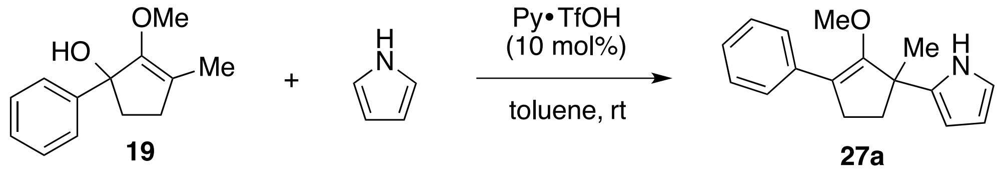

Compound **19** (50 mg, 0.245 mmol) was dissolved in toluene (1.2 mL). Pyrrole (34 µL, 0.490 mmol) and then pyridinium triflate (6 mg, 0.025 mmol) were added. Upon stirring at room temperature for 1.5 hours, the reaction mixture was then directly purified with flash column

chromatography with 100% hexanes → 90 : 10 hexanes : Et_2O to give product **27a** (28 mg, 45% yield) as yellow oil.

^{1}H NMR (500 MHz, $CDCl_3$): δ = 8.32 (bs, 1H), 7.56 (d, J = 9.7 Hz, 2H), 7.34 (t, J = 7.6 Hz, 2H), 7.22 (t, J = 7.2 Hz, 1H), 6.73-6.72 (m, 1H), 6.18-6.16 (m, 1H), 6.06-6.04 (m, 1H), 3.48 (s, 3H), 2.78-2.68 (m, 2H), 2.32-2.26 (m, 1H), 2.07 (ddd, J = 12.7, 8.5, 5.7 Hz, 1H), 1.61 (s, 3H). ^{13}C NMR (125 MHz, $CDCl_3$): δ = 158.63, 138.23, 136.30, 128.08, 127.39, 126.37, 116.59, 116.29, 108.04, 103.37, 59.00, 48.14, 37.30, 29.61, 24.78. IR (neat): cm^{-1}; 3442, 2963, 2935, 2846, 1634, 1599, 1493, 1444, 1417, 1341, 1325, 1308, 1272, 1239, 1211, 1173, 1102, 1077, 1032, 988, 787, 763, 719, 697, 536. HRMS $(M + H)^+$ = 254.1539 calculated for $C_{17}H_{20}NO$; experimental = 254.1529.

(±)-*2-(2-methoxy-1-methyl-3-phenylcyclopent-2-en-1-yl)-3,5-dimethyl-1H-pyrrole* (27b)

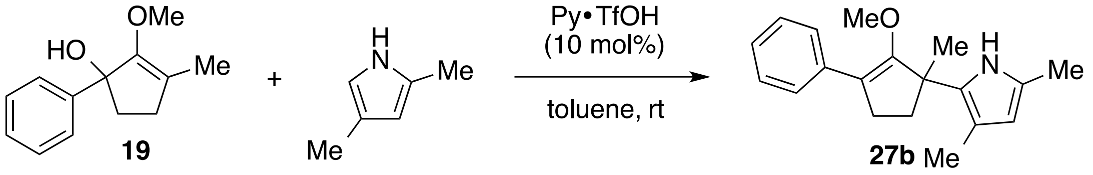

Compound **19** (50 mg, 0.245 mmol) was dissolved in toluene (1.2 mL). 2,4-Dimethylpyrrole (50 μL, 0.490 mmol) and then pyridinium triflate (6 mg, 0.025 mmol) were added. Upon stirring at room temperature for 30 minutes, the reaction mixture was then directly purified with flash column chromatography with 100% hexanes → 95 : 5 hexanes : Et_2O → 90 : 10 hexanes : Et_2O → 80 : 20 hexanes : Et_2O to give product **27b** (57 mg, 83% yield) as yellow oil.

^{1}H NMR (500 MHz, $CDCl_3$): δ = 8.01 (bs, 1H), 7.56-7.54 (m, 2H), 7.37-7.33 (m, 2H), 7.24-7.20 (m, 1H), 5.67 (d, J = 2.9 Hz, 1H), 3.56 (s, 3H), 2.79 (ddd, J = 14.8, 8.8, 6.8 Hz, 1H),

2.61 (ddd, J = 14.8, 9.0, 3.8 Hz, 1H), 2.36 (ddd, J = 12.8, 9.2, 6.8 Hz, 1H), 2.22 (s, 3H), 2.14-2.09 (m, 1H), 2.11 (s, 3H), 1.60 (s, 3H). ^{13}C NMR (125 MHz, $CDCl_3$): δ = 158.66, 136.55, 131.20, 128.56, 127.38, 126.27, 123.78, 116.40, 113.28, 109.51, 59.48, 48.73, 37.08, 29.77, 24.93, 12.95, 12.40. IR (cm^{-1}): 3442, 2963, 2935, 2846, 1634, 1599, 1493, 1444, 1417, 1341, 1325, 1308, 1272, 1239, 1211, 1173, 1102, 1077, 1032, 988, 787, 763, 719, 697, 536. HRMS $(M + H)^+$ = 282.1852 calculated for $C_{19}H_{24}NO$; experimental = 282.1850.

(±)-*1-(2-methoxy-1-methyl-3-phenylcyclopent-2-en-1-yl)azulene* (27c)

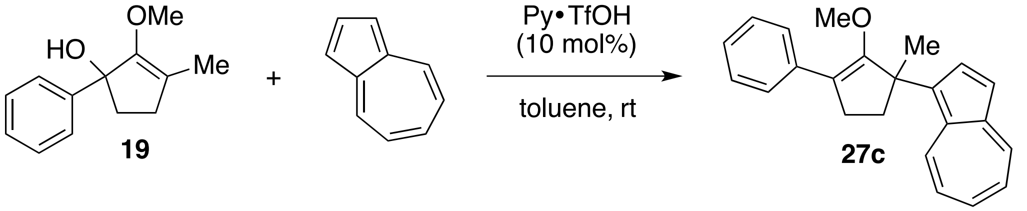

Compound **19** (20 mg, 0.098 mmol) was dissolved in toluene (0.6 mL). Azulene (14 mg, 0.108 mmol) and then pyridinium triflate (2 mg, 0.010 mmol) were added. Upon stirring at room temperature for 30 minutes, the reaction mixture was then directly purified with flash column chromatography with 100% hexanes → 99.75 : 0.25 hexanes : Et_2O to give product **27c** (19 mg, 62% yield) as a blue oil.

^{1}H NMR (500 MHz, $CDCl_3$): δ = 8.70 (d, J = 9.9 Hz, 1H), 8.30 (d, J = 9.7 Hz, 1H), 7.96 (d, J = 3.9 Hz, 1H), 7.69-7.63 (m, 2H), 7.55 (t, J = 9.8 Hz, 1H), 7.41-7.37 (m, 2H), 7.34 (d, J = 4.0 Hz, 1H), 7.25-7.21 (m, 1H), 7.08 (ddd, J = 9.7, 9.7, 7.3 Hz, 2H), 3.31 (s, 3H), 2.95 (dt, J = 14.7, 8.3 Hz, 1H), 2.79 (ddd, J = 14.7, 9.3, 2.6 Hz, 1H), 2.57 (dt, J = 12.9, 8.9 Hz, 1H), 2.16 (ddd, J = 12.9, 8.5, 2.6 Hz, 1H), 1.92 (s, 3H). ^{13}C NMR (125 MHz, $CDCl_3$): δ = 160.67, 141.54, 137.45, 137.06, 136.78, 135.76, 135.66, 135.55, 135.04, 128.08, 127.21, 125.86, 122.37, 121.83, 116.28, 114.00, 54.30, 49.75, 40.37, 29.49, 26.36. IR (neat): cm^{-1}; 3085, 3064, 3048, 3024, 2962,

2930, 2871, 2845, 1735, 1628, 1598, 1573, 1493, 1454, 1444, 1397, 1371, 1353, 1342, 1326, 1273, 1250, 1219, 1170, 1130, 1103, 1078, 1063, 1051, 1032, 1004, 760, 743, 695. HRMS (M + Na)$^+$ = 337.1563 calculated for $C_{23}H_{22}NaO$; experimental = 337.1575.

(±)-*5-(2-methoxy-1-methyl-3-phenylcyclopent-2-en-1-yl)furan-2(5H)-one* (27d)

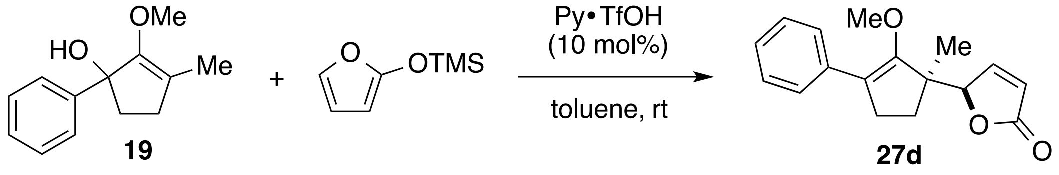

Compound **19** (50 mg, 0.245 mmol) was dissolved in toluene (1.2 mL). 2-(Trimethylsiloxy)furan (82 μL, 0.490 mmol) and then pyridinium triflate (6 mg, 0.025 mmol) were added. Upon stirring at room temperature for 72 hours, the reaction mixture was then directly purified with flash column chromatography with 100% hexanes → 90 : 10 hexanes : Et_2O → 80 : 20 hexanes : Et_2O to give product **27d** as a single diastereomer (41 mg, 62% yield) as a pale yellow crystal.

^{1}H NMR (400 MHz, $CDCl_3$): δ (ppm) = 7.48 (dd, J = 5.8, 1.4 Hz, 1H), 7.40-7.31 (m, 4H), 7.25-7.21 (m, 1H), 6.17 (dd, J = 5.7, 2.1 Hz, 1H), 5.09 (t, J = 1.7 Hz, 1H), 3.53 (s, 3H), 2.66-2.58 (m, 1H), 2.45 (ddd, J = 15.0, 8.9, 6.0 Hz, 1H), 1.77 (ddd, J = 13.3, 8.9, 4.2 Hz, 1H), 1.65-1.58 (m, 1H), 1.43 (s, 3H). ^{13}C NMR (100 MHz, $CDCl_3$): δ = 173.32, 155.40, 155.11, 136.11, 128.08, 127.92, 126.84, 122.21, 117.70, 88.39, 60.11, 52.85, 31.13, 28.55, 22.91. IR (neat): cm^{-1}; 3056, 3023, 2966, 2937, 2851, 1787, 1753, 1644, 1599, 1494, 1445, 1374, 1312, 1273, 1256, 1216, 1161, 1116, 1091, 1063, 1042, 991, 893, 822, 796, 764, 701, 540. HRMS (M + H)$^+$ = 271.1329 calculated for $C_{17}H_{19}O_3$; experimental = 271.1322.

(±)-*(2,3-dimethoxy-3-methylcyclopent-1-en-1-yl)benzene* (27e)

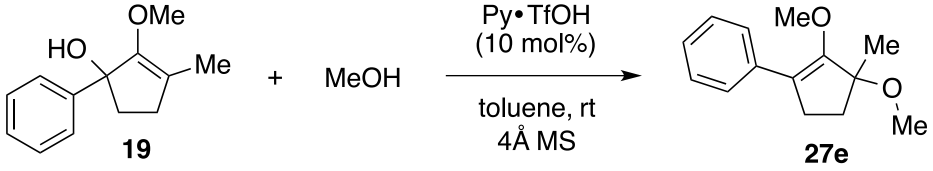

Compound **19** (100 mg, 0.490 mmol) was dissolved in toluene (2.4 mL). 4Å molecular sieves (118 mg), methanol (40 μL, 0.980 mmol), and then pyridinium triflate (12 mg, 0.050 mmol) were added. Upon stirring at room temperature for 23 hours, the reaction mixture was then directly purified with flash column chromatography (buffered with 2% TEA) with 100% hexanes → 85 : 15 hexanes : Et_2O to give product **27e** (74 mg, 69% yield) as colorless oil.

^{1}H NMR (400 MHz, $CDCl_3$): δ (ppm) = 7.61-7.58 (m, 2H), 7.38-7.34 (m, 2H), 7.27-7.23 (m, 1H), 3.77 (s, 3H), 3.30 (s, 3H), 2.68 (ddd, J = 15.1, 8.9, 5.1 Hz, 1H), 2.59 (ddd, J = 15.1, 8.9, 4.4 Hz, 1H), 2.20 (ddd, J = 13.6, 9.0, 4.4 Hz, 1H), 1.91 (ddd, J = 14.0, 8.9, 5.1 Hz, 1H), 1.52 (s, 3H). ^{13}C NMR (125 MHz, $CDCl_3$): δ (ppm) = 153.71, 135.96, 128.08, 127.43, 126.71, 119.66, 86.77, 58.46, 50.55, 31.34, 28.92, 24.90. IR (cm^{-1}): 2968, 2934, 2850, 1638, 1599, 1493, 1444, 1370, 1345, 1328, 1308, 1279, 1250, 1218, 1189, 1154, 1125, 1089, 1064, 1031, 988, 911, 876, 856, 760, 719, 693, 653, 596, 534. HRMS $(M + Na)^+$ = 241.1199 calculated for $C_{14}H_{18}NaO_2$; experimental = 241.1211.

(±)-*(3-((2-methoxy-1-methyl-3-phenylcyclopent-2-en-1-yl)oxy)propyl)benzene* (27f)

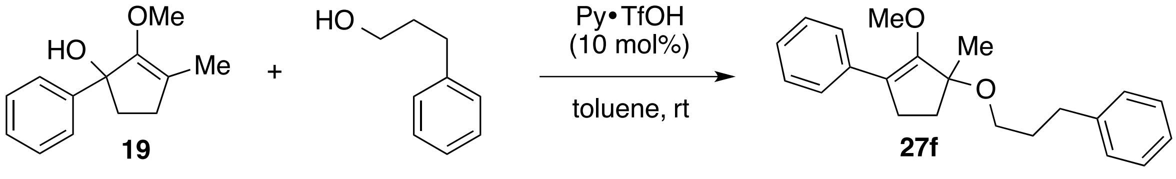

Compound **19** (50 mg, 0.245 mmol) was dissolved in toluene (1.2 mL). 3-Phenyl-1-propanol (67 μL, 0.490 mmol) and then pyridinium triflate (6 mg, 0.025 mmol) were added.

Upon stirring at room temperature for 1 hour, the reaction mixture was then directly purified with flash column chromatography (buffered with 2% TEA) with 100% hexanes → 98 : 2 hexanes : Et_2O → 95 : 5 hexanes : Et_2O → 90 : 10 hexanes : Et_2O to give product **27f** (31 mg, 39% yield) as colorless oil.

^1H NMR (500 MHz, $CDCl_3$): δ = 7.57 (d, J = 7.9 Hz, 2H), 7.34 (t, J = 7.7 Hz, 2H), 7.29-7.16 (m, 6H), 3.75 (s, 3H), 3.43 (t, J = 6.6 Hz, 2H), 2.71 (t, J = 7.8 Hz, 2H), 2.62 (ddd, J = 15.0, 8.8, 5.4 Hz, 1H), 2.54 (ddd, J = 15.1, 8.9, 4.4 Hz, 1H), 2.18-2.12 (m, 1H), 1.95-1.87 (m, 3H), 1.51 (s, 3H). ^{13}C NMR (125 MHz, $CDCl_3$): δ =154.48, 142.20, 136.02, 128.44, 128.26, 128.05, 127.37, 126.59, 125.69, 119.09, 86.25, 62.02, 58.45, 32.57, 32.30, 31.96, 28.79, 25.13. IR (neat): cm^{-1}; 3084, 3058, 3025, 2931, 2853, 1707, 1638, 1600, 1494, 1453, 1445, 1369, 1344, 1329, 1308, 1279, 1252, 1217, 1187, 1121, 1087, 1065, 1030, 988, 911, 862, 797, 761, 746, 695, 581, 543, 493. HRMS $(M + Na)^+$ = 345.1825 calculated for $C_{22}H_{26}NaO_2$; experimental = 345.1835

(±)-*(2-methoxy-1-methyl-3-phenylcyclopent-2-en-1-yl)(phenyl)sulfane* (27g)

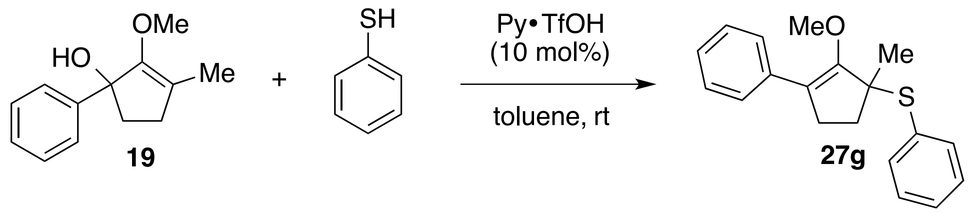

Compound **19** (50 mg, 0.245 mmol) was dissolved in toluene (1.2 mL). Thiophenol (50 μL, 0.490 mmol) and then pyridinium triflate (6 mg, 0.025 mmol) were added. Upon stirring at room temperature for 1.5 hours, the reaction mixture was then directly purified with flash column chromatography with 100% hexanes → 90 : 10 hexanes : Et_2O → 80 : 20 hexanes : Et_2O to give product **27g** (57 mg, 78% yield) as colorless oil.

^{1}H NMR (400 MHz, $CDCl_3$): δ = 7.62-7.59 (2H, m), 7.38-7.19 (8H, m), 3.73 (3H, s), 2.36-2.26 (2H, m), 2.08-2.01 (1H, m), 1.86-1.78 (1H, m), 1.59 (3H, s). ^{13}C NMR (100 MHz, $CDCl_3$): δ = 155.42, 136.91, 136.17, 132.93, 128.64, 128.32, 127.97, 127.53, 126.59, 119.37, 62.55, 60.08, 36.52, 30.11, 25.98. IR (neat): cm^{-1}; 3054, 3020, 3962, 2924, 2851, 1633, 1598, 1582, 1275, 1249, 1210, 1173, 1157, 1104, 989, 915, 830, 803, 761, 749, 692, 655, 618, 560, 536, 499, 478. HRMS $(M + Na)^+$ = 319.1127 calculated for $C_{19}H_{20}NaOS$; experimental = 319.1140.

(±)-*2-(2-methoxy-3-methyl-3,4,5,6-tetrahydro-[1,1'-biphenyl]-3-yl)-1H-pyrrole* (28a)

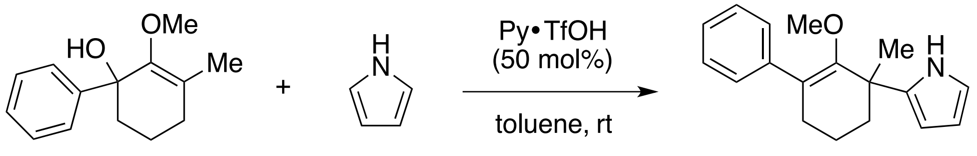

20 **28a**

Compound **20** (50 mg, 0.229 mmol) was dissolved in toluene (1.2 mL). Pyrrole (32 µL, 0.458 mmol) and then pyridinium triflate (25 mg, 0.115 mmol) were added. Upon stirring at room temperature for 18 hours, the reaction mixture was then directly purified with flash column chromatography with 100% hexanes → 90 : 10 hexanes : Et_2O → 80 : 20 hexanes : Et_2O to give product **28a** (55 mg, 89% yield) as colorless oil.

^{1}H NMR (500 MHz, $CDCl_3$): δ = 8.68 (bs, 1H), 7.39-7.36 (m, 2H), 7.34-7.31 (m, 2H), 7.24-7.20 (m, 1H), 6.72 (ddd, J = 2.6, 2.6, 1.5 Hz, 1H), 6.17 (dd, J = 6.0, 2.7 Hz, 1H), 6.03-6.02 (m, 1H), 3.17 (s, 3H), 2.56-2.49 (m, 2H), 2.34-2.28 (m, 1H), 2.23-2.18 (m, 1H), 1.89-1.71 (m, 3H), 1.53 (s, 3H). ^{13}C NMR (125 MHz, $CDCl_3$): δ = 156.11, 140.96, 138.85, 128.26, 128.15, 126.42, 120.79, 116.18, 107.68, 102.95, 60.85, 40.34, 38.25, 31.56, 27.20, 19.92. IR (cm^{-1}): 3443, 2932, 2865, 2834, 1644, 1598, 1554, 1492, 1442, 1416, 1368, 1289, 1271, 1245, 1229,

1193, 1142, 1122, 1078, 1034, 992, 950, 884, 832, 786, 762, 716, 698, 634, 609, 548, 502, 464. HRMS $(M + H)^+$ = 268.1696 calculated for $C_{18}H_{22}NO$; experimental = 268.1702.

(±)-*2-(2-methoxy-3-methyl-3,4,5,6-tetrahydro-[1,1'-biphenyl]-3-yl)azulene* (28c)

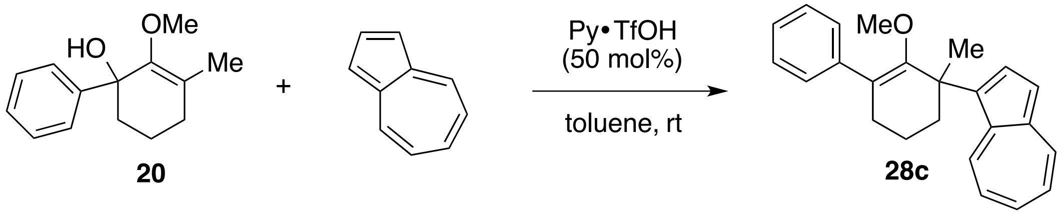

Compound **20** (20 mg, 0.092 mmol) was dissolved in toluene (0.46 mL). Azulene (13 mg, 0.101 mmol) and then pyridinium triflate (10 mg, 0.046 mmol) were added. Upon stirring at room temperature for 39 hours, the reaction mixture was then directly purified with flash column chromatography with 100% hexanes → 99.5 : 0.5 hexanes : Et_2O to give product **28c** (12 mg, 39% yield) as blue oil.

^{1}H NMR (500 MHz, $CDCl_3$): δ = 8.87 (d, J = 9.9 Hz, 1H), 8.29 (d, J = 9.4 Hz, 1H), 7.99 (d, J = 3.9 Hz, 1H), 7.56 (t, J = 9.8 Hz, 1H), 7.49 (d, J = 8.0 Hz, 2H), 7.36-7.33 (m, 3H), 7.25-7.21 (m, 1H), 7.10 (dt, J = 13.0, 9.9 Hz, 2H), 3.04 (s, 3H), 2.74-2.69 (m, 1H), 2.66-2.59 (m, 1H), 2.45-2.39 (m, 1H), 1.96-1.87 (m, 2H), 1.89 (s, 3H), 1.84-1.78 (m, 1H). ^{13}C NMR (125 MHz, $CDCl_3$): δ = 158.94, 141.49, 141.37, 137.29, 136.92, 136.74, 136.53, 135.14, 134.31, 128.38, 128.23, 126.31, 122.23, 121.24, 118.27, 116.41, 61.10, 42.63, 40.88, 31.69, 26.56, 20.54. IR (neat): cm^{-1}; 2967, 2933, 2863, 2834, 1643, 1571, 1455, 1442, 1396, 1136, 1025, 1009, 999, 762, 742, 698. HRMS $(M + H)^+$ = 329.1900 calculated for $C_{24}H_{25}O$; experimental = 329.1911.

(±)-*6-methoxy-5-methyl-5-(3-phenylpropoxy)-2,3,4,5-tetrahydro-1,1'-biphenyl* (28f)

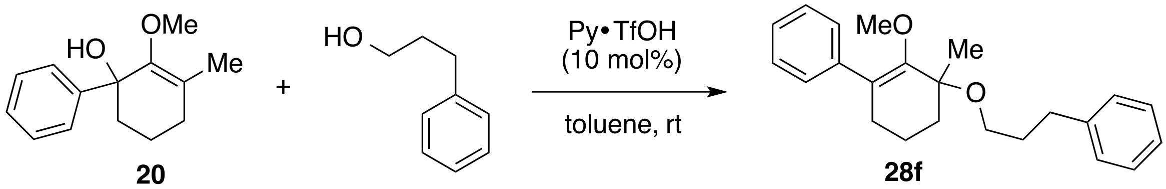

Compound **20** (50 mg, 0.229 mmol) was dissolved in toluene (1.2 mL). 3-Phenyl-1-propanol (62 μL, 0.458 mmol) and then pyridinium triflate (5 mg, 0.023 mmol) were added. Upon stirring at room temperature for 4 hours, the reaction mixture was then directly purified with flash column chromatography (buffered with 2% TEA) with 100% hexanes → 98 : 2 hexanes : Et_2O → 95 : 5 hexanes : Et_2O → 90 : 10 hexanes : Et_2O to give product **28f** (72 mg, 94% yield) as colorless oil.

^{1}H NMR (500 MHz, $CDCl_3$): δ = 7.36-7.17 (m, 10H), 3.53-3.47 (m, 2H), 3.28 (s, 3H), 2.76-2.72 (m, 2H), 2.38 (t, J = 5.9 Hz, 1H), 2.06 (ddd, J = 13.4, 7.8, 3.0 Hz, 1H), 1.96-1.85 (m, 3H), 1.69-1.62 (m, 1H), 1.61-1.55 (m, 1H), 1.42 (s, 3H). ^{13}C NMR (125 MHz, $CDCl_3$): δ = 154.00, 142.39 140.54, 128.51, 128.32, 128.25, 120.08, 126.54, 125.63, 123.46, 75.94, 61.62, 60.76, 35.07, 32.61, 32.23, 31.43, 23.75, 19.70. IR (neat): cm^{-1}; 3082, 3058, 3025, 2932, 2862, 2833, 1645, 1600, 1493, 1475, 1453, 1443, 1385, 1365, 1339, 1299, 1275, 1237, 1191, 1156, 1131, 1104, 1071, 1043, 1011, 990, 961, 930, 909, 881, 858, 805, 759, 747, 696, 666, 653, 633, 620, 599, 577, 541, 494. HRMS $(M + Na)^+$ = 359.1982 calculated for $C_{23}H_{28}NaO_2$; experimental = 359.1987.

(±)-*(2-methoxy-3-methyl-3,4,5,6-tetrahydro-[1,1'-biphenyl]-3-yl)(phenyl)sulfane* (28g)

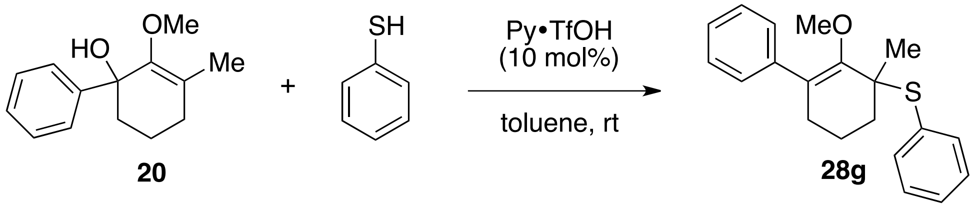

Compound **20** (50 mg, 0.229 mmol) was dissolved in toluene (1.2 mL). Thiophenol (47 μL, 0.458 mmol) and then pyridinium triflate (5 mg, 0.023 mmol) were added. Upon stirring at room temperature for 20 hours, the reaction mixture was then directly purified with flash column chromatography (buffered with 2% TEA) with 100% hexanes → 98 : 2 hexanes : Et_2O → 95 : 5 hexanes : Et_2O → 90 : 10 hexanes : Et_2O → 80 : 20 hexanes : Et_2O to give product **28g** (66 mg, 93% yield) as colorless oil.

^{1}H NMR (500 MHz, $CDCl_3$): δ = 7.63-7.61 (m, 2H), 7.39-7.32 (m, 7H), 7.26-7.22 (m, 1H), 3.30 (s, 3H), 2.43-2.29 (m, 2H), 2.09-2.02 (m, 1H), 2.01-1.95 (m, 1H), 1.72-1.66 (m, 2H), 1.46 (s, 3H). ^{13}C NMR (125 MHz, $CDCl_3$): δ = 154.21, 140.51, 137.36, 132.67, 129.07, 128.61, 128.45, 128.32, 128.16, 127.53, 127.16, 126.63, 122.88, 61.40, 54.39, 37.45, 31.33, 26.34, 19.49. IR (neat): cm^{-1}; 3054, 3019, 2930, 2861, 2833, 1637, 1598, 1491, 1475, 1438, 1368, 1335, 1287, 1265, 1236, 1194, 1168, 1144, 1126, 1084, 1068, 1039, 1016, 1001, 990, 956, 922, 866, 849, 802, 748, 692, 645, 633, 609, 585, 566, 554, 524, 480. HRMS $(M + Na)^+$ = 333.1284 calculated for $C_{20}H_{22}NaOS$; experimental = 333.1273.

(±)-*2-(1H-indol-3-yl)-2-methyl-5-phenylcyclopentanone* (29)

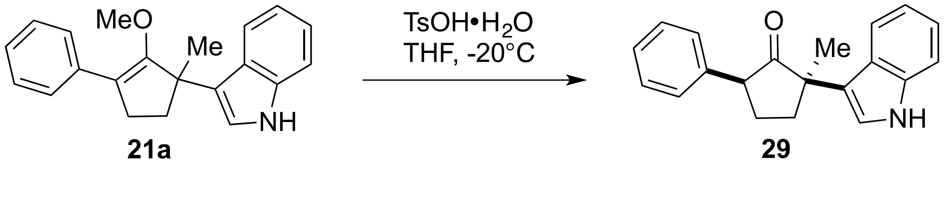

Compound **21a** (50 mg, 0.165 mmol) was dissolved in THF (1.7 mL). After cooling the solution to -20°C, TsOH•H_2O (63 mg, 0.320) was added, and the reaction mixture was stirred at this temperature for 72 hours until the completion of reaction, as monitored by TLC. The reaction mixture was neutralized with aqueous sodium bicarbonate (10 mL) and extracted with EtOAc (3 x 10 mL). The combined organic layers were then washed with water, followed by brine, and concentrated in *vacuo*. The crude residue was further purified by flash column chromatography with 90:10 hexanes : EtOAc to afford product **29** as a 5.5:1 mixture of diastereoisomers (35 mg, 73% yield) as a white solid.

Major diastereomer: ^{1}H NMR (500 MHz, $CDCl_3$): δ (ppm) = 8.00 (bs, 1H), 7.85 (d, J = 8.0 Hz, 1H), 7.37 (d, J = 8.0 Hz, 1H), 7.27 – 7.18 (m, 5H), 7.14 (d, J = 7.1 Hz, 1H), 7.12 – 7.08 (m, 2H), 6.77 (d, J = 2.5 Hz, 1H), 3.49 (dd, J = 10.9, 8.4 Hz, 1H), 2.99 – 2.92 (m, 1H), 2.43 – 2.35 (m, 1H), 2.14 – 2.03 (m, 2H), 1.64 (s, 3H). ^{13}C NMR (125 MHz, $CDCl_3$): δ (ppm) = 218.12, 139.08, 137.57, 128.67, 128.38, 126.93, 125.50, 122.43, 122.40, 120.94, 119.71, 115.94, 111.70, 55.09, 50.17, 35.88, 28.12, 24.86. IR (cm^{-1}): 3334, 2959, 2922, 2853, 1722, 1457, 1259, 1089, 1022, 798, 742. HRMS $(M + H)^+$ = 290.1545 calculated for $C_{20}H_{20}NO$; experimental = 290.1539.

(±)-*3-(2-methoxy-1-methyl-3-phenylcyclopentyl)-1H-indole* (30)

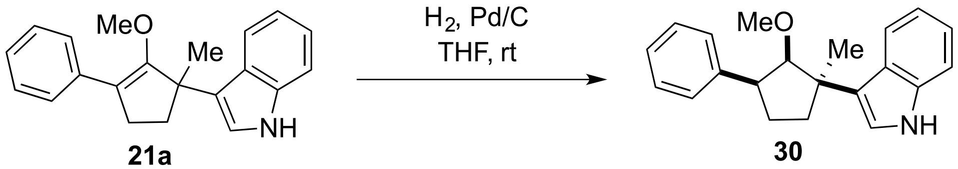

21a **30**

Compound **21a** (50 mg, 0.165 mmol) was dissolved in THF (1.7 mL), and Pd/C (100 mg) was then added. The reaction mixture was stirred at room temperature under a hydrogen balloon for 24 hours until the completion of reaction, as monitored by TLC. The suspension was then

filtered through a pad of celite and washed with EtOAc. After concentrating the filtrate in *vacuo*, the crude residue was further purified by flash column chromatography with 85:15 hexanes : EtOAc to afford product **30** as a 2.3:1 mixture of diastereoisomers (33 mg, 64% yield) as a white solid.

Major diastereomer: ^{1}H NMR (500 MHz, $CDCl_3$): δ (ppm) = 7.96 (bs, 1H), 7.80 (d, J = 8.0 Hz, 1H), 7.44 (d, J = 7.3 Hz, 2H), 7.37 – 7.30 (m, 3H), 7.23 (dd, J = 7.3, 7.3 Hz, 1H), 7.20 – 7.15 (m, 1H), 7.15 – 7.06 (m, 1H), 6.99 (d, J = 2.4 Hz, 1H), 3.91 (d, J = 4.2 Hz, 1H), 3.73 (td, J = 9.5, 4.2 Hz, 1H), 2.71 – 2.58 (m, 1H), 2.50 (s, 3H), 2.38 – 2.23 (m, 2H), 1.98 (ddd, J = 12.6, 8.0, 4.9 Hz, 1H), 1.56 (s, 3H). ^{13}C NMR (125 MHz, $CDCl_3$): δ (ppm) = 141.72, 137.23, 129.39, 128.08, 126.78, 126.23, 122.83, 121.63, 121.20, 121.11, 118.92, 111.42, 92.89, 59.84, 48.81, 48.67, 36.10, 28.13, 27.88. IR (cm^{-1}): 3290, 2960, 2925, 1455, 1261, 1092, 1014, 802, 738, 698. HRMS $(M + H)^+$ = 328.1672 calculated for $C_{21}H_{23}NNaO$; experimental = 328.1670.

(±)-*1-(3-(2-methoxy-1-methyl-3-phenylcyclopent-2-enyl)-1H-indol-1-yl)ethanone* (31)

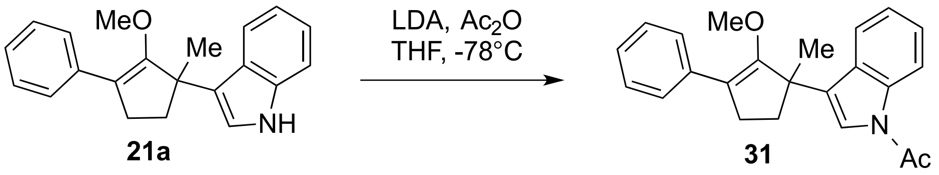

A solution of diisopropylamine (0.7 mL, 4.95 mmol) in THF (8 mL) was cooled to 0°C. *n*-BuLi (1.98 mL, 2.5M in hexane) was then added dropwise. After stirring for 30 minutes, a solution of compound **21a** (500 mg, 1.65 mmol) in THF (8 mL) was added dropwise. Upon further stirring for 60 minutes, acetic anhydride (0.47 mL, 4.95 mmol) was added. The reaction was continued for another 60 minutes until the completion of reaction, as monitored by TLC. The reaction was then quenched with saturated aqueous solution of NH_4Cl (10 mL), and the mixture was partitioned between EtOAc/H_2O (50 mL, 1:1). The aqueous layer was extracted

with EtOAc (3 x 50 mL). The combined organic layers were then washed thoroughly with water, followed by brine, dried over Na_2SO_4, and concentrated in *vacuo*. The crude material was further purified by flash column chromatography with 85:15 hexanes : EtOAc to afford compound **31** (500 mg, 88%) as white solid.

^{1}H NMR (400 MHz, $CDCl_3$): δ (ppm) = 8.50 (d, J = 7.6 Hz, 1H), 7.76 (d, J = 7.8 Hz, 1H), 7.62 (d, J = 7.7 Hz, 2H), 7.41 (d, J = 7.5 Hz, 2H), 7.38 (d, J = 7.9 Hz, 1H), 7.33 (d, J = 10.2 Hz, 1H), 7.27 (dd, J = 7.5, 7.5 Hz, 2H), 3.49 (s, 3H), 3.02 – 2.90 (m, 1H), 2.80 (ddd, J = 14.9, 9.4, 3.2 Hz, 1H), 2.67 (s, 3H), 2.51 (ddd, J = 13.0, 9.3, 7.5 Hz, 1H), 2.11 – 1.99 (m, 1H), 1.76 (s, 3H). ^{13}C NMR (100 MHz, $CDCl_3$): δ (ppm) = 168.63, 158.94, 136.83, 136.79, 129.62, 129.26, 128.30, 127.72, 126.57, 125.16, 123.56, 121.47, 120.85, 116.93, 116.04, 59.55, 48.37, 36.38, 30.29, 24.63, 24.32. IR (cm^{-1}): 2962, 2936, 1698, 1633, 1492, 1449, 1379, 1343, 1228, 994, 933, 909, 731, 697. HRMS $(M + H)^+$ = 346.1802 calculated for $C_{23}H_{24}NO_2$; experimental = 346.1800.

(±)-*1-(3-(1-methoxy-2-methyl-5-phenyl-6-oxabicyclo[3.1.0]hexan-2-yl)-1H-indol-1-yl)ethanone* (**32**)

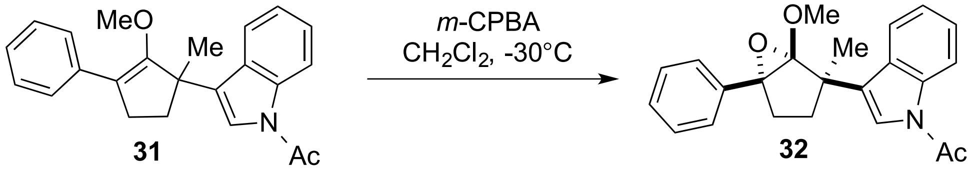

Compound **31** (40 mg, 0.116 mmol) was dissolved in dichloromethane (1.3 mL), and the solution was then cooled to -30°C. *Meta*-chloroperoxybenzoic acid (31 mg, 0.139 mmol, 77% purity) was then added dropwise as a solution in dichloromethane (1 mL). The reaction mixture was slowly warmed to room temperature and then stirred for 2 hours until the completion of reaction, as monitored by TLC. The reaction mixture was neutralized with aqueous sodium

bicarbonate (5 mL) and extracted with dichloromethane (3 x 5 mL). The combined organic layers were then washed with water, followed by brine, and then concentrated in *vacuo*. The crude residue was further purified by flash column chromatography with 90:10 hexanes : EtOAc to afford product **32** as a 5:1 mixture diastereomers (35 mg, 83% yield) as a white solid.

Major diastereomer: ^{1}H NMR (500 MHz, $CDCl_3$): δ (ppm) = 8.53 (d, J = 8.1 Hz, 1H), 7.71 (d, J = 7.9 Hz, 1H), 7.48 (d, J = 6.9 Hz, 3H), 7.42 (t, J = 7.5 Hz, 2H), 7.40 – 7.34 (m, 2H), 7.32 – 7.28 (m, 1H), 3.48 (s, 3H), 2.57 (s, 3H), 2.23 – 2.10 (m, 2H), 2.08 – 1.98 (m, 1H), 1.82 (s, 3H), 1.81 – 1.71 (m, 1H). ^{13}C NMR (125 MHz, $CDCl_3$): δ (ppm) = 168.73, 136.67, 136.08, 129.28, 128.58, 127.99, 126.54, 125.15, 123.44, 122.79, 120.90, 117.19, 97.57, 73.79, 54.91, 45.51, 33.74, 29.71, 24.33, 21.71. IR (cm^{-1}): 2965, 2928, 1705, 1449, 1391, 1229, 1199, 1073, 1023, 1009, 907, 730, 697. HRMS $(M + H)^+$ = 362.1751 calculated for $C_{23}H_{24}NO_3$; experimental = 362.1756.

(±)-*methyl 2-(1-acetyl-1H-indol-3-yl)-2-methyl-5-oxo-5-phenylpentanoate* (33)

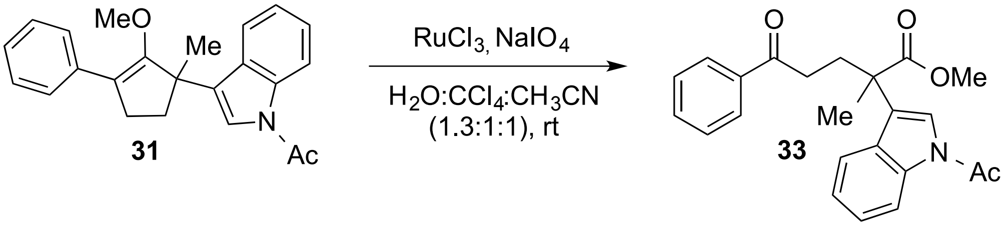

Compound **31** (50 mg, 0.145 mmol) was dissolved in a mixture of $H_2O/CCl_4/CH_3CN$ (1.3:1:1, 0.05 M). $NaIO_4$ (124 mg, 0.579 mmol) was then added, followed by $RuCl_3$ (1.5 mg, 0.0073 mmol). The reaction mixture was stirred at room temperature for 1.5 hours until the completion of reaction, as monitored by TLC. The reaction mixture was quenched with saturated aqueous $Na_2S_2O_3$ (5mL), stirred for 15 min, and then extracted with dichloromethane (3 x 5 mL). The combined organic layers were then washed with water, followed by brine, and

then concentrated in *vacuo*. The crude residue was further purified by flash column chromatography with 80:20 hexanes : EtOAc to afford product **33** (40 mg, 73% yield) as a white solid.

^{1}H NMR (400 MHz, $CDCl_3$): δ (ppm) = 8.47 (d, J = 7.6 Hz, 1H), 7.87 (d, J = 7.3 Hz, 2H), 7.54 (d, J = 7.1 Hz, 2H), 7.42 (dd, J = 7.7, 7.7 Hz, 2H), 7.39 – 7.33 (m, 2H), 7.26 (dd, J = 7.4, 7.4 Hz, 1H), 3.68 (s, 3H), 3.05 – 2.89 (m, 2H), 2.66 (s, 3H), 2.61 (dt, J = 9.2, 6.2 Hz, 2H), 1.72 (s, 3H). ^{13}C NMR (125 MHz, $CDCl_3$): δ (ppm) = 199.55, 175.89, 168.61, 136.79, 136.51, 133.32, 128.76, 128.73, 128.20, 125.52, 125.34, 123.85, 122.19, 120.09, 116.9, 52.66, 45.42, 34.21, 32.11, 24.34, 23.15. IR (cm^{-1}): 2949, 2926, 1728, 1706, 1685, 1452, 1380, 1332, 1295, 1230, 1105, 746. HRMS $(M + Na)^+$ = 400.1519 calculated for $C_{23}H_{23}NNaO_4$; experimental = 400.1519.

X-RAY CRYSTAL DATA

(±)-*5-methoxy-3-(2-methoxy-1-methyl-3-phenylcyclopent-2-enyl)-1H-indole* (21c)

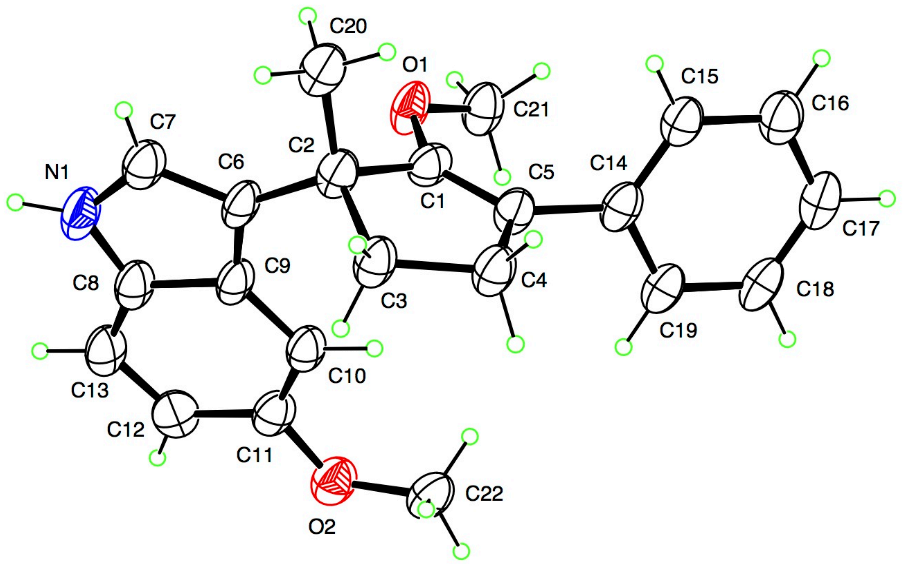

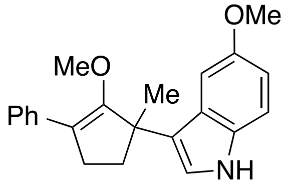

21c

Crystal data

$C_{22}H_{23}NO_2$	$F(000) = 712$
$M_r = 333.41$	$D_x = 1.278$ Mg m^{-3}
Monoclinic, $P2_1/c$	Cu $K\alpha$ radiation, $\lambda = 1.54184$ Å
Hall symbol: -P 2ybc	Cell parameters from 3184 reflections
$a = 14.0542$ (10) Å	$\theta = 3.2$–61.2°
$b = 15.9871$ (11) Å	$\mu = 0.64$ mm^{-1}
$c = 7.7187$ (4) Å	$T = 90$ K
$\beta = 91.974$ (4)°	Needle, colourless
$V = 1733.25$ (19) Å^3	0.37 × 0.05 × 0.02 mm
$Z = 4$	

Data collection

Bruker Kappa APEX-II DUO diffractometer	2537 independent reflections
Radiation source: IμS microfocus	1965 reflections with $I > 2\sigma(I)$
QUAZAR multilayer optics	$R_{int} = 0.082$
ϕ and ω scans	$\theta_{max} = 61.2°$, $\theta_{min} = 3.2°$
Absorption correction: multi-scan TWINABS (Sheldrick, 2004)	$h = 0\rightarrow15$
$T_{min} = 0.618$, $T_{max} = 0.987$	$k = -17\rightarrow0$
13157 measured reflections	$l = -8\rightarrow8$

Refinement

Refinement on F^2	Primary atom site location: structure-invariant direct methods
Least-squares matrix: full	Secondary atom site location: difference Fourier map
$R[F^2 > 2\sigma(F^2)] = 0.072$	Hydrogen site location: inferred from neighbouring sites
$wR(F^2) = 0.210$	H atoms treated by a mixture of independent and constrained refinement
$S = 1.05$	$w = 1/[\sigma^2(F_o^2) + (0.1411P)^2 + 0.3725P]$ where $P = (F_o^2 + 2F_c^2)/3$
2537 reflections	$(\Delta/\sigma)_{max} < 0.001$
232 parameters	$\Delta\rangle_{max} = 0.33$ e Å^{-3}
0 restraints	$\Delta\rangle_{min} = -0.30$ e Å^{-3}

Refinement. The crystal was a nonmerohedral twin by twofold rotation about reciprocal 0 0 1. Refinement of F^2 against ALL reflections in a TWIN4 file prepared by TWINABS. When refined vs. a TWIN5 file, the BASF parameter refined to 0.279 (5).

Fractional atomic coordinates and isotropic or equivalent isotropic displacement parameters (Å^2)

	x	*y*	*z*	U_{iso}*/U_{eq}	
O1	0.80629 (17)	0.60700 (14)	0.1714 (2)	0.0396 (6)	
O2	0.43063 (16)	0.51840 (15)	0.2137 (3)	0.0432 (6)	
N1	0.6418 (2)	0.75114 (19)	0.5822 (4)	0.0433 (8)	
H1N	0.628 (3)	0.798 (3)	0.636 (5)	0.052*	
C1	0.8095 (2)	0.5447 (2)	0.2939 (4)	0.0357 (8)	
C2	0.8064 (2)	0.5774 (2)	0.4780 (4)	0.0370 (8)	
C3	0.7893 (2)	0.4943 (2)	0.5787 (4)	0.0385 (8)	
H3A	0.8305	0.4921	0.6853	0.046*	
H3B	0.7220	0.4903	0.6120	0.046*	
C4	0.8138 (3)	0.4225 (2)	0.4564 (4)	0.0397 (8)	
H4A	0.8760	0.3974	0.4904	0.048*	
H4B	0.7643	0.3784	0.4574	0.048*	
C5	0.8174 (2)	0.4625 (2)	0.2801 (4)	0.0346 (8)	
C6	0.7261 (2)	0.63932 (19)	0.5008 (4)	0.0360 (8)	
C7	0.7296 (3)	0.7139 (2)	0.5890 (4)	0.0395 (8)	
H7	0.7848	0.7363	0.6463	0.047*	
C8	0.5787 (3)	0.7014 (2)	0.4894 (4)	0.0389 (8)	
C9	0.6300 (2)	0.63013 (19)	0.4369 (4)	0.0356 (8)	
C10	0.5815 (2)	0.5667 (2)	0.3414 (4)	0.0365 (8)	
H10	0.6143	0.5182	0.3045	0.044*	
C11	0.4862 (2)	0.5768 (2)	0.3034 (4)	0.0373 (8)	
C12	0.4364 (3)	0.6486 (2)	0.3550 (4)	0.0410 (9)	
H12	0.3705	0.6539	0.3256	0.049*	
C13	0.4825 (3)	0.7113 (2)	0.4478 (4)	0.0429 (9)	
H13	0.4492	0.7599	0.4824	0.051*	
C14	0.8377 (2)	0.4080 (2)	0.1271 (4)	0.0357 (8)	
C15	0.9285 (2)	0.4048 (2)	0.0629 (4)	0.0385 (8)	
H15	0.9772	0.4390	0.1137	0.046*	
C16	0.9497 (3)	0.3527 (2)	-0.0738 (4)	0.0446 (9)	
H16	1.0121	0.3518	-0.1171	0.054*	
C17	0.8790 (3)	0.3018 (2)	-0.1473 (4)	0.0442 (9)	
H17	0.8930	0.2654	-0.2403	0.053*	
C18	0.7883 (3)	0.3044 (2)	-0.0848 (4)	0.0414 (9)	
H18	0.7397	0.2702	-0.1358	0.050*	
C19	0.7676 (3)	0.3568 (2)	0.0526 (4)	0.0392 (8)	

H19	0.7052	0.3576	0.0958	0.047*	
C20	0.9036 (2)	0.6158 (2)	0.5286 (4)	0.0440 (9)	
H20A	0.9536	0.5738	0.5151	0.066*	
H20B	0.9035	0.6344	0.6496	0.066*	
H20C	0.9157	0.6638	0.4534	0.066*	
C21	0.7936 (3)	0.5828 (2)	-0.0057 (4)	0.0410 (9)	
H21A	0.8522	0.5568	-0.0451	0.062*	
H21B	0.7788	0.6322	-0.0766	0.062*	
H21C	0.7411	0.5426	-0.0172	0.062*	
C22	0.4733 (3)	0.4386 (2)	0.1842 (4)	0.0426 (9)	
H22A	0.5245	0.4447	0.1022	0.064*	
H22B	0.4250	0.4000	0.1362	0.064*	
H22C	0.4995	0.4163	0.2941	0.064*	

Atomic displacement parameters (Å^2)

	U^{11}	U^{22}	U^{33}	U^{12}	U^{13}	U^{23}
O1	0.0661 (16)	0.0202 (13)	0.0327 (12)	0.0037 (11)	0.0037 (9)	0.0014 (8)
O2	0.0529 (15)	0.0334 (14)	0.0434 (13)	0.0002 (11)	0.0014 (10)	0.0018 (10)
N1	0.068 (2)	0.0223 (16)	0.0403 (16)	0.0018 (15)	0.0084 (13)	-0.0026 (12)
C1	0.0451 (19)	0.024 (2)	0.0385 (18)	-0.0023 (14)	0.0042 (13)	0.0047 (13)
C2	0.052 (2)	0.028 (2)	0.0309 (16)	-0.0008 (15)	0.0025 (13)	-0.0007 (12)
C3	0.057 (2)	0.026 (2)	0.0324 (17)	0.0014 (16)	0.0039 (13)	0.0016 (13)
C4	0.058 (2)	0.027 (2)	0.0346 (17)	0.0004 (16)	0.0035 (14)	0.0006 (13)
C5	0.0458 (19)	0.028 (2)	0.0305 (16)	-0.0008 (14)	0.0022 (12)	-0.0026 (12)
C6	0.058 (2)	0.0172 (18)	0.0328 (16)	-0.0013 (15)	0.0056 (13)	0.0008 (12)
C7	0.057 (2)	0.0261 (19)	0.0357 (17)	-0.0005 (16)	0.0057 (13)	0.0021 (13)
C8	0.060 (2)	0.0225 (19)	0.0343 (17)	0.0035 (16)	0.0087 (14)	0.0034 (13)
C9	0.056 (2)	0.0182 (18)	0.0329 (17)	0.0007 (14)	0.0081 (13)	0.0022 (12)
C10	0.053 (2)	0.0236 (19)	0.0331 (17)	0.0014 (15)	0.0076 (13)	0.0020 (12)
C11	0.050 (2)	0.027 (2)	0.0351 (17)	-0.0005 (15)	0.0013 (13)	0.0039 (13)

C12	0.051 (2)	0.034 (2)	0.0378 (18)	0.0058 (16)	0.0059 (14)	0.0115 (14)
C13	0.066 (2)	0.0235 (19)	0.0401 (18)	0.0076 (17)	0.0125 (15)	0.0070 (13)
C14	0.052 (2)	0.0219 (19)	0.0328 (16)	0.0021 (15)	-0.0026 (13)	0.0038 (12)
C15	0.049 (2)	0.027 (2)	0.0391 (18)	0.0014 (15)	0.0009 (14)	-0.0008 (13)
C16	0.060 (2)	0.033 (2)	0.0408 (19)	0.0038 (17)	0.0069 (15)	0.0006 (14)
C17	0.068 (2)	0.029 (2)	0.0352 (17)	0.0066 (17)	-0.0013 (15)	-0.0035 (14)
C18	0.059 (2)	0.022 (2)	0.0433 (18)	0.0017 (16)	-0.0088 (15)	0.0000 (14)
C19	0.051 (2)	0.0238 (19)	0.0426 (18)	0.0014 (15)	-0.0025 (14)	0.0008 (13)
C20	0.057 (2)	0.034 (2)	0.0415 (18)	-0.0024 (17)	0.0009 (15)	-0.0033 (14)
C21	0.061 (2)	0.029 (2)	0.0327 (18)	0.0053 (16)	0.0017 (14)	0.0021 (13)
C22	0.054 (2)	0.030 (2)	0.0445 (19)	-0.0034 (16)	-0.0001 (14)	0.0036 (14)

Geometric parameters (Å, º)

O1—C1	1.373 (4)	C10—H10	0.9500
O1—C21	1.426 (4)	C11—C12	1.409 (5)
O2—C11	1.387 (4)	C12—C13	1.381 (5)
O2—C22	1.432 (4)	C12—H12	0.9500
N1—C7	1.369 (5)	C13—H13	0.9500
N1—C8	1.375 (5)	C14—C15	1.386 (5)
N1—H1N	0.87 (4)	C14—C19	1.390 (5)
C1—C5	1.323 (5)	C15—C16	1.384 (5)
C1—C2	1.516 (4)	C15—H15	0.9500
C2—C6	1.516 (5)	C16—C17	1.390 (5)
C2—C20	1.536 (5)	C16—H16	0.9500
C2—C3	1.561 (5)	C17—C18	1.379 (5)
C3—C4	1.533 (5)	C17—H17	0.9500
C3—H3A	0.9900	C18—C19	1.390 (5)
C3—H3B	0.9900	C18—H18	0.9500
C4—C5	1.506 (5)	C19—H19	0.9500
C4—H4A	0.9900	C20—H20A	0.9800
C4—H4B	0.9900	C20—H20B	0.9800
C5—C14	1.503 (4)	C20—H20C	0.9800
C6—C7	1.372 (5)	C21—H21A	0.9800

C6—C9	1.430 (5)	C21—H21B	0.9800
C7—H7	0.9500	C21—H21C	0.9800
C8—C13	1.387 (5)	C22—H22A	0.9800
C8—C9	1.415 (5)	C22—H22B	0.9800
C9—C10	1.415 (5)	C22—H22C	0.9800
C10—C11	1.371 (5)		
C1—O1—C21	117.6 (3)	C10—C11—C12	121.8 (3)
C11—O2—C22	116.6 (3)	O2—C11—C12	114.4 (3)
C7—N1—C8	109.4 (3)	C13—C12—C11	120.6 (3)
C7—N1—H1N	124 (3)	C13—C12—H12	119.7
C8—N1—H1N	126 (3)	C11—C12—H12	119.7
C5—C1—O1	131.8 (3)	C12—C13—C8	118.3 (3)
C5—C1—C2	115.0 (3)	C12—C13—H13	120.9
O1—C1—C2	113.2 (3)	C8—C13—H13	120.9
C6—C2—C1	112.3 (3)	C15—C14—C19	118.5 (3)
C6—C2—C20	111.5 (3)	C15—C14—C5	120.3 (3)
C1—C2—C20	108.8 (3)	C19—C14—C5	121.1 (3)
C6—C2—C3	111.7 (3)	C16—C15—C14	121.3 (3)
C1—C2—C3	100.6 (3)	C16—C15—H15	119.3
C20—C2—C3	111.4 (3)	C14—C15—H15	119.3
C4—C3—C2	106.7 (3)	C15—C16—C17	119.6 (3)
C4—C3—H3A	110.4	C15—C16—H16	120.2
C2—C3—H3A	110.4	C17—C16—H16	120.2
C4—C3—H3B	110.4	C18—C17—C16	119.7 (3)
C2—C3—H3B	110.4	C18—C17—H17	120.1
H3A—C3—H3B	108.6	C16—C17—H17	120.1
C5—C4—C3	104.7 (3)	C17—C18—C19	120.3 (3)
C5—C4—H4A	110.8	C17—C18—H18	119.8
C3—C4—H4A	110.8	C19—C18—H18	119.8
C5—C4—H4B	110.8	C14—C19—C18	120.5 (3)
C3—C4—H4B	110.8	C14—C19—H19	119.8
H4A—C4—H4B	108.9	C18—C19—H19	119.8
C1—C5—C14	131.1 (3)	C2—C20—H20A	109.5
C1—C5—C4	110.1 (3)	C2—C20—H20B	109.5
C14—C5—C4	118.5 (3)	H20A—C20—H20B	109.5
C7—C6—C9	106.1 (3)	C2—C20—H20C	109.5
C7—C6—C2	127.7 (3)	H20A—C20—H20C	109.5
C9—C6—C2	126.2 (3)	H20B—C20—H20C	109.5
N1—C7—C6	110.0 (3)	O1—C21—H21A	109.5
N1—C7—H7	125.0	O1—C21—H21B	109.5

C6—C7—H7	125.0	H21A—C21—H21B	109.5
N1—C8—C13	131.4 (3)	O1—C21—H21C	109.5
N1—C8—C9	106.8 (3)	H21A—C21—H21C	109.5
C13—C8—C9	121.8 (3)	H21B—C21—H21C	109.5
C10—C9—C8	119.0 (3)	O2—C22—H22A	109.5
C10—C9—C6	133.3 (3)	O2—C22—H22B	109.5
C8—C9—C6	107.7 (3)	H22A—C22—H22B	109.5
C11—C10—C9	118.4 (3)	O2—C22—H22C	109.5
C11—C10—H10	120.8	H22A—C22—H22C	109.5
C9—C10—H10	120.8	H22B—C22—H22C	109.5
C10—C11—O2	123.8 (3)		
C21—O1—C1—C5	11.8 (5)	C13—C8—C9—C10	-0.9 (4)
C21—O1—C1—C2	-170.3 (3)	N1—C8—C9—C6	-0.3 (3)
C5—C1—C2—C6	-132.1 (3)	C13—C8—C9—C6	-179.8 (3)
O1—C1—C2—C6	49.6 (4)	C7—C6—C9—C10	-178.2 (3)
C5—C1—C2—C20	103.9 (3)	C2—C6—C9—C10	0.4 (5)
O1—C1—C2—C20	-74.4 (3)	C7—C6—C9—C8	0.4 (3)
C5—C1—C2—C3	-13.2 (4)	C2—C6—C9—C8	179.1 (3)
O1—C1—C2—C3	168.5 (3)	C8—C9—C10—C11	0.0 (4)
C6—C2—C3—C4	135.8 (3)	C6—C9—C10—C11	178.5 (3)
C1—C2—C3—C4	16.4 (3)	C9—C10—C11—O2	-178.8 (3)
C20—C2—C3—C4	-98.7 (3)	C9—C10—C11—C12	0.8 (4)
C2—C3—C4—C5	-14.9 (3)	C22—O2—C11—C10	9.9 (4)
O1—C1—C5—C14	8.6 (6)	C22—O2—C11—C12	-169.6 (2)
C2—C1—C5—C14	-169.3 (3)	C10—C11—C12—C13	-0.6 (5)

O1—C1—C5—C4	-177.9 (3)	O2—C11—C12—C13	179.0 (3)
C2—C1—C5—C4	4.2 (4)	C11—C12—C13—C8	-0.3 (4)
C3—C4—C5—C1	7.1 (4)	N1—C8—C13—C12	-178.3 (3)
C3—C4—C5—C14	-178.5 (3)	C9—C8—C13—C12	1.1 (4)
C1—C2—C6—C7	-136.4 (3)	C1—C5—C14—C15	73.5 (5)
C20—C2—C6—C7	-13.9 (4)	C4—C5—C14—C15	-99.6 (4)
C3—C2—C6—C7	111.4 (4)	C1—C5—C14—C19	-109.8 (4)
C1—C2—C6—C9	45.2 (4)	C4—C5—C14—C19	77.1 (4)
C20—C2—C6—C9	167.7 (3)	C19—C14—C15—C16	0.8 (5)
C3—C2—C6—C9	-66.9 (4)	C5—C14—C15—C16	177.6 (3)
C8—N1—C7—C6	0.2 (4)	C14—C15—C16—C17	-0.7 (5)
C9—C6—C7—N1	-0.3 (3)	C15—C16—C17—C18	0.7 (5)
C2—C6—C7—N1	-179.0 (3)	C16—C17—C18—C19	-0.8 (5)
C7—N1—C8—C13	179.5 (3)	C15—C14—C19—C18	-0.8 (5)
C7—N1—C8—C9	0.1 (3)	C5—C14—C19—C18	-177.6 (3)
N1—C8—C9—C10	178.6 (3)	C17—C18—C19—C14	0.8 (5)

(±)-*5-methoxy-3-(2-methoxy-1-methyl-3-phenylcyclohex-2-enyl)-1H-indole* (22b)

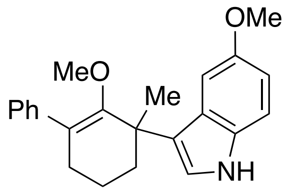

22b

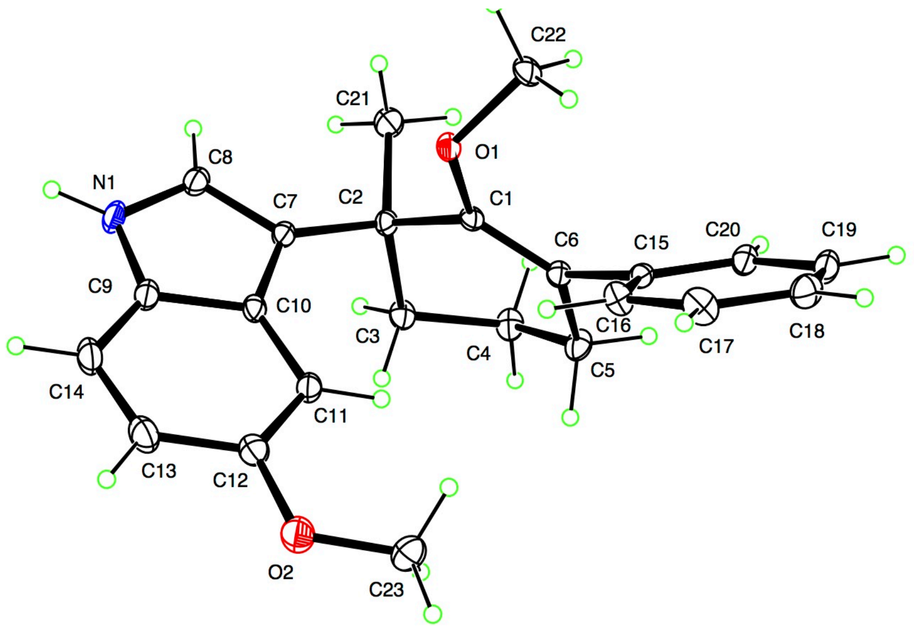

Crystal data

$C_{23}H_{25}NO_2$	F(000) = 1488
M_r = 347.44	D_x = 1.253 Mg m^{-3}
Orthorhombic, *Pbca*	Mo $K\alpha$ radiation, λ = 0.71073 Å
Hall symbol: -P 2ac 2ab	Cell parameters from 9950 reflections
a = 7.5352 (3) Å	θ = 2.5–30.5°
b = 16.1007 (6) Å	μ = 0.08 mm^{-1}
c = 30.3669 (12) Å	T = 90 K
V = 3684.2 (2) Å^3	Needle, colourless
Z = 8	0.44 × 0.16 × 0.09 mm

Data collection

Bruker Kappa APEX-II DUO diffractometer	5627 independent reflections
Radiation source: fine-focus sealed tube	4736 reflections with $I > 2\sigma(I)$
TRIUMPH curved graphite	R_{int} = 0.043
ϕ and ω scans	θ_{max} = 30.5°, θ_{min} = 2.5°
Absorption correction: multi-scan *SADABS* (Sheldrick, 2004)	h = -10 – 9
T_{min} = 0.900, T_{max} = 0.993	k = -22 – 22
50544 measured reflections	l = -41 – 43

Refinement

Refinement on F^2	Primary atom site location: structure-invariant direct methods
Least-squares matrix: full	Secondary atom site location: difference Fourier map
$R[F^2 > 2\sigma(F^2)] = 0.043$	Hydrogen site location: inferred from neighbouring sites
$wR(F^2) = 0.114$	H atoms treated by a mixture of independent and constrained refinement
$S = 1.06$	$w = 1/[\sigma^2(F_o^2) + (0.0562P)^2 + 1.5053P]$ where $P = (F_o^2 + 2F_c^2)/3$
5627 reflections	$(\Delta/\sigma)_{max} = 0.001$
241 parameters	$\Delta\rangle_{max} = 0.45$ e Å^{-3}
0 restraints	$\Delta\rangle_{min} = -0.21$ e Å^{-3}

Fractional atomic coordinates and isotropic or equivalent isotropic displacement parameters (Å^2)

	x	*y*	*z*	U_{iso}*/U_{eq}	
O1	0.14716 (9)	0.61972 (5)	0.66120 (2)	0.01365 (15)	
O2	0.55810 (12)	0.89891 (5)	0.62757 (3)	0.02121 (18)	
N1	0.64117 (12)	0.64350 (6)	0.74545 (3)	0.01605 (18)	
H1N	0.682 (2)	0.6398 (9)	0.7727 (5)	0.019*	
C1	0.25741 (12)	0.59221 (6)	0.62789 (3)	0.01040 (17)	
C2	0.42193 (12)	0.55042 (6)	0.64702 (3)	0.01118 (17)	
C3	0.56127 (13)	0.53718 (6)	0.61032 (3)	0.01336 (18)	
H3A	0.6209	0.5907	0.6040	0.016*	
H3B	0.6525	0.4976	0.6208	0.016*	
C4	0.47885 (14)	0.50382 (7)	0.56802 (3)	0.01488 (19)	
H4A	0.5731	0.4935	0.5460	0.018*	
H4B	0.4180	0.4505	0.5741	0.018*	
C5	0.34635 (13)	0.56636 (7)	0.54988 (3)	0.01506 (19)	
H5A	0.4122	0.6129	0.5362	0.018*	

H5B	0.2751	0.5393	0.5265	0.018*	
C6	0.22179 (12)	0.60093 (6)	0.58464 (3)	0.01096 (17)	
C7	0.50345 (12)	0.60453 (6)	0.68264 (3)	0.01141 (18)	
C8	0.56507 (13)	0.57853 (7)	0.72313 (3)	0.01464 (19)	
H8	0.5561	0.5234	0.7340	0.018*	
C9	0.62790 (13)	0.71391 (7)	0.72013 (3)	0.01433 (19)	
C10	0.54284 (12)	0.69191 (6)	0.68019 (3)	0.01195 (18)	
C11	0.51621 (13)	0.75333 (6)	0.64762 (3)	0.01338 (18)	
H11	0.4607	0.7402	0.6204	0.016*	
C12	0.57335 (14)	0.83315 (7)	0.65658 (3)	0.0163 (2)	
C13	0.65557 (15)	0.85429 (7)	0.69694 (4)	0.0197 (2)	
H13	0.6914	0.9100	0.7021	0.024*	
C14	0.68442 (14)	0.79498 (7)	0.72894 (3)	0.0187 (2)	
H14	0.7406	0.8087	0.7560	0.022*	
C15	0.06761 (12)	0.64799 (6)	0.56679 (3)	0.01131 (18)	
C16	0.03336 (14)	0.72979 (6)	0.57977 (3)	0.01550 (19)	
H16	0.1056	0.7550	0.6016	0.019*	
C17	-0.10530 (15)	0.77471 (7)	0.56101 (4)	0.0191 (2)	
H17	-0.1270	0.8302	0.5702	0.023*	
C18	-0.21232 (14)	0.73882 (7)	0.52884 (4)	0.0181 (2)	
H18	-0.3055	0.7699	0.5157	0.022*	
C19	-0.18187 (14)	0.65731 (7)	0.51613 (3)	0.0163 (2)	
H19	-0.2560	0.6320	0.4947	0.020*	
C20	-0.04299 (13)	0.61248 (6)	0.53477 (3)	0.01383 (19)	
H20	-0.0227	0.5568	0.5256	0.017*	
C21	0.36532 (14)	0.46621 (7)	0.66690 (3)	0.0170 (2)	

H21A	0.4691	0.4386	0.6797	0.026*	
H21B	0.3146	0.4311	0.6437	0.026*	
H21C	0.2764	0.4755	0.6899	0.026*	
C22	-0.03964 (13)	0.60171 (7)	0.65837 (3)	0.0164 (2)	
H22A	-0.1008	0.6480	0.6439	0.025*	
H22B	-0.0880	0.5941	0.6881	0.025*	
H22C	-0.0574	0.5508	0.6413	0.025*	
C23	0.47364 (16)	0.88257 (7)	0.58672 (4)	0.0195 (2)	
H23A	0.5427	0.8414	0.5703	0.029*	
H23B	0.4659	0.9341	0.5696	0.029*	
H23C	0.3540	0.8610	0.5921	0.029*	

Atomic displacement parameters (Å^2)

	U^{11}	U^{22}	U^{33}	U^{12}	U^{13}	U^{23}
O1	0.0077 (3)	0.0234 (4)	0.0098 (3)	0.0002 (3)	0.0013 (2)	-0.0036 (3)
O2	0.0264 (4)	0.0154 (4)	0.0218 (4)	-0.0044 (3)	-0.0054 (3)	0.0026 (3)
N1	0.0149 (4)	0.0244 (5)	0.0089 (4)	0.0007 (3)	-0.0029 (3)	0.0003 (3)
C1	0.0080 (4)	0.0127 (4)	0.0105 (4)	0.0001 (3)	0.0010 (3)	-0.0010 (3)
C2	0.0090 (4)	0.0141 (4)	0.0105 (4)	0.0009 (3)	-0.0011 (3)	0.0005 (3)
C3	0.0099 (4)	0.0169 (5)	0.0133 (4)	0.0025 (3)	0.0004 (3)	-0.0024 (3)
C4	0.0130 (4)	0.0180 (5)	0.0136 (4)	0.0036 (4)	0.0008 (3)	-0.0044 (4)
C5	0.0133 (4)	0.0216 (5)	0.0104 (4)	0.0037 (4)	0.0010 (3)	-0.0020 (3)
C6	0.0096 (4)	0.0125 (4)	0.0107 (4)	0.0009 (3)	0.0003 (3)	-0.0009 (3)
C7	0.0082 (4)	0.0166 (4)	0.0094 (4)	0.0012 (3)	0.0001 (3)	0.0004 (3)
C8	0.0122 (4)	0.0203 (5)	0.0115 (4)	0.0013 (4)	-0.0003 (3)	0.0018 (3)
C9	0.0112 (4)	0.0213 (5)	0.0105 (4)	0.0008 (4)	-0.0008 (3)	-0.0017 (3)
C10	0.0085 (4)	0.0170 (5)	0.0103 (4)	0.0003 (3)	-0.0002 (3)	-0.0014 (3)

C11	0.0122 (4)	0.0162 (4)	0.0117 (4)	-0.0001 (3)	-0.0017 (3)	-0.0004 (3)
C12	0.0148 (4)	0.0169 (5)	0.0173 (5)	-0.0011 (4)	-0.0011 (4)	0.0008 (4)
C13	0.0185 (5)	0.0200 (5)	0.0207 (5)	-0.0045 (4)	-0.0026 (4)	-0.0042 (4)
C14	0.0161 (5)	0.0250 (5)	0.0151 (5)	-0.0026 (4)	-0.0034 (4)	-0.0054 (4)
C15	0.0104 (4)	0.0136 (4)	0.0099 (4)	0.0008 (3)	0.0006 (3)	0.0017 (3)
C16	0.0132 (4)	0.0146 (5)	0.0186 (5)	0.0001 (4)	-0.0020 (4)	-0.0016 (4)
C17	0.0169 (5)	0.0140 (5)	0.0264 (5)	0.0026 (4)	-0.0010 (4)	-0.0001 (4)
C18	0.0148 (5)	0.0211 (5)	0.0185 (5)	0.0047 (4)	-0.0010 (4)	0.0046 (4)
C19	0.0142 (4)	0.0233 (5)	0.0114 (4)	0.0018 (4)	-0.0026 (3)	0.0003 (4)
C20	0.0139 (4)	0.0163 (5)	0.0113 (4)	0.0011 (4)	-0.0011 (3)	-0.0006 (3)
C21	0.0169 (5)	0.0165 (5)	0.0176 (5)	-0.0011 (4)	-0.0022 (4)	0.0032 (4)
C22	0.0081 (4)	0.0257 (5)	0.0154 (4)	-0.0012 (4)	0.0019 (3)	-0.0024 (4)
C23	0.0224 (5)	0.0181 (5)	0.0180 (5)	0.0017 (4)	-0.0011 (4)	0.0030 (4)

Geometric parameters (Å, º)

O1—C1	1.3818 (11)	C10—C11	1.4129 (14)
O1—C22	1.4397 (12)	C11—C12	1.3824 (15)
O2—C12	1.3821 (13)	C11—H11	0.9500
O2—C23	1.4189 (13)	C12—C13	1.4148 (15)
N1—C8	1.3721 (14)	C13—C14	1.3798 (16)
N1—C9	1.3735 (14)	C13—H13	0.9500
N1—H1N	0.886 (15)	C14—H14	0.9500
C1—C6	1.3479 (13)	C15—C16	1.3987 (14)
C1—C2	1.5254 (13)	C15—C20	1.4025 (13)
C2—C7	1.5188 (13)	C16—C17	1.3925 (15)
C2—C21	1.5443 (14)	C16—H16	0.9500
C2—C3	1.5458 (13)	C17—C18	1.3924 (16)
C3—C4	1.5245 (14)	C17—H17	0.9500
C3—H3A	0.9900	C18—C19	1.3870 (16)

C3—H3B	0.9900	C18—H18	0.9500
C4—C5	1.5212 (14)	C19—C20	1.3915 (14)
C4—H4A	0.9900	C19—H19	0.9500
C4—H4B	0.9900	C20—H20	0.9500
C5—C6	1.5182 (13)	C21—H21A	0.9800
C5—H5A	0.9900	C21—H21B	0.9800
C5—H5B	0.9900	C21—H21C	0.9800
C6—C15	1.4893 (13)	C22—H22A	0.9800
C7—C8	1.3793 (13)	C22—H22B	0.9800
C7—C10	1.4398 (14)	C22—H22C	0.9800
C8—H8	0.9500	C23—H23A	0.9800
C9—C14	1.3988 (15)	C23—H23B	0.9800
C9—C10	1.4168 (13)	C23—H23C	0.9800
C1—O1—C22	118.66 (8)	C12—C11—H11	121.0
C12—O2—C23	116.91 (8)	C10—C11—H11	121.0
C8—N1—C9	108.80 (8)	O2—C12—C11	124.11 (9)
C8—N1—H1N	123.8 (9)	O2—C12—C13	113.84 (9)
C9—N1—H1N	127.2 (9)	C11—C12—C13	122.04 (10)
C6—C1—O1	124.07 (9)	C14—C13—C12	120.84 (10)
C6—C1—C2	125.36 (8)	C14—C13—H13	119.6
O1—C1—C2	110.57 (7)	C12—C13—H13	119.6
C7—C2—C1	110.29 (8)	C13—C14—C9	117.58 (9)
C7—C2—C21	109.68 (8)	C13—C14—H14	121.2
C1—C2—C21	108.16 (8)	C9—C14—H14	121.2
C7—C2—C3	108.53 (8)	C16—C15—C20	118.01 (9)
C1—C2—C3	109.78 (8)	C16—C15—C6	121.37 (9)
C21—C2—C3	110.39 (8)	C20—C15—C6	120.56 (9)
C4—C3—C2	112.29 (8)	C17—C16—C15	120.82 (10)
C4—C3—H3A	109.1	C17—C16—H16	119.6
C2—C3—H3A	109.1	C15—C16—H16	119.6
C4—C3—H3B	109.1	C18—C17—C16	120.40 (10)
C2—C3—H3B	109.1	C18—C17—H17	119.8
H3A—C3—H3B	107.9	C16—C17—H17	119.8
C5—C4—C3	109.83 (8)	C19—C18—C17	119.47 (10)
C5—C4—H4A	109.7	C19—C18—H18	120.3
C3—C4—H4A	109.7	C17—C18—H18	120.3
C5—C4—H4B	109.7	C18—C19—C20	120.14 (10)
C3—C4—H4B	109.7	C18—C19—H19	119.9
H4A—C4—H4B	108.2	C20—C19—H19	119.9
C6—C5—C4	113.37 (8)	C19—C20—C15	121.15 (10)
C6—C5—H5A	108.9	C19—C20—H20	119.4

C4—C5—H5A	108.9	C15—C20—H20	119.4
C6—C5—H5B	108.9	C2—C21—H21A	109.5
C4—C5—H5B	108.9	C2—C21—H21B	109.5
H5A—C5—H5B	107.7	H21A—C21—H21B	109.5
C1—C6—C15	124.26 (9)	C2—C21—H21C	109.5
C1—C6—C5	121.08 (9)	H21A—C21—H21C	109.5
C15—C6—C5	114.57 (8)	H21B—C21—H21C	109.5
C8—C7—C10	105.86 (9)	O1—C22—H22A	109.5
C8—C7—C2	126.65 (9)	O1—C22—H22B	109.5
C10—C7—C2	127.38 (8)	H22A—C22—H22B	109.5
N1—C8—C7	110.47 (9)	O1—C22—H22C	109.5
N1—C8—H8	124.8	H22A—C22—H22C	109.5
C7—C8—H8	124.8	H22B—C22—H22C	109.5
N1—C9—C14	129.86 (9)	O2—C23—H23A	109.5
N1—C9—C10	107.81 (9)	O2—C23—H23B	109.5
C14—C9—C10	122.33 (9)	H23A—C23—H23B	109.5
C11—C10—C9	119.24 (9)	O2—C23—H23C	109.5
C11—C10—C7	133.70 (9)	H23A—C23—H23C	109.5
C9—C10—C7	107.05 (9)	H23B—C23—H23C	109.5
C12—C11—C10	117.95 (9)		
C22—O1—C1—C6	46.49 (14)	C14—C9—C10—C11	1.06 (15)
C22—O1—C1—C2	-132.97 (9)	N1—C9—C10—C7	0.51 (11)
C6—C1—C2—C7	132.23 (10)	C14—C9—C10—C7	-179.82 (9)
O1—C1—C2—C7	-48.32 (10)	C8—C7—C10—C11	179.03 (11)
C6—C1—C2—C21	-107.82 (11)	C2—C7—C10—C11	2.65 (18)
O1—C1—C2—C21	71.63 (10)	C8—C7—C10—C9	0.09 (11)
C6—C1—C2—C3	12.69 (13)	C2—C7—C10—C9	-176.28 (9)
O1—C1—C2—C3	-167.86 (8)	C9—C10—C11—C12	-0.71 (14)
C7—C2—C3—C4	-164.76 (8)	C7—C10—C11—C12	-179.54 (10)
C1—C2—C3—C4	-44.15 (11)	C23—O2—C12—C11	1.82 (15)
C21—C2—C3—C4	75.00 (10)	C23—O2—C12—C13	-178.79 (10)
C2—C3—C4—C5	62.29 (11)	C10—C11—C12—O2	179.07 (9)
C3—C4—C5—C6	-46.20 (12)	C10—C11—C12—C13	-0.28 (16)
O1—C1—C6—C15	5.83 (16)	O2—C12—C13—	-178.42 (10)

		C14	
C2—C1—C6—C15	-174.79 (9)	C11—C12—C13—C14	0.98 (17)
O1—C1—C6—C5	-177.81 (9)	C12—C13—C14—C9	-0.63 (16)
C2—C1—C6—C5	1.57 (15)	N1—C9—C14—C13	179.21 (11)
C4—C5—C6—C1	15.60 (14)	C10—C9—C14—C13	-0.38 (16)
C4—C5—C6—C15	-167.70 (9)	C1—C6—C15—C16	52.17 (14)
C1—C2—C7—C8	135.83 (10)	C5—C6—C15—C16	-124.40 (10)
C21—C2—C7—C8	16.81 (13)	C1—C6—C15—C20	-130.82 (11)
C3—C2—C7—C8	-103.87 (11)	C5—C6—C15—C20	52.61 (12)
C1—C2—C7—C10	-48.51 (13)	C20—C15—C16—C17	-0.71 (15)
C21—C2—C7—C10	-167.54 (9)	C6—C15—C16—C17	176.38 (10)
C3—C2—C7—C10	71.79 (12)	C15—C16—C17—C18	-0.13 (17)
C9—N1—C8—C7	1.03 (12)	C16—C17—C18—C19	1.17 (17)
C10—C7—C8—N1	-0.68 (11)	C17—C18—C19—C20	-1.36 (16)
C2—C7—C8—N1	175.73 (9)	C18—C19—C20—C15	0.52 (16)
C8—N1—C9—C14	179.43 (11)	C16—C15—C20—C19	0.52 (15)
C8—N1—C9—C10	-0.93 (11)	C6—C15—C20—C19	-176.59 (9)
N1—C9—C10—C11	-178.60 (9)		

Hydrogen-bond geometry (Å, º)

D—H···*A*	*D*—H	H···*A*	*D*···*A*	*D*—H···*A*
N1—H1*N*···O1^{i}	0.886 (15)	2.050 (15)	2.8607 (11)	151.6 (13)

Symmetry code: (i) *x*+1/2, *y*, -*z*+3/2.

(±)-*5-methoxy-3-(2-methoxy-1-methyl-3-phenylcyclohex-2-enyl)-1H-indole* (27d)

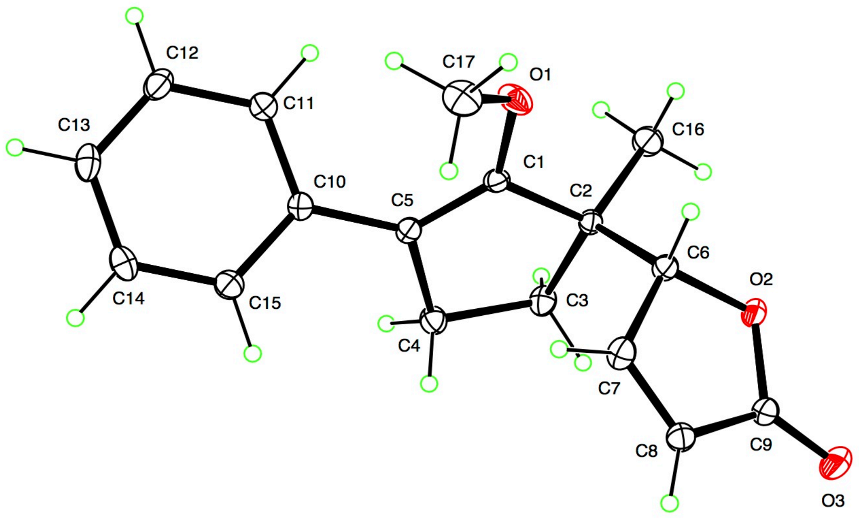

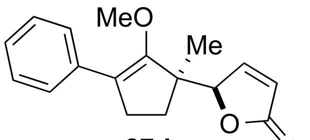

27d

Crystal data

$C_{17}H_{18}O_3$	$Z = 2$
$M_r = 270.31$	$F(000) = 288$
Triclinic, $P\bar{1}$	$D_x = 1.308$ Mg m^{-3}
Hall symbol: -P 1	Mo $K\alpha$ radiation, $\lambda = 0.71073$ Å
$a = 6.2616$ (10) Å	Cell parameters from 7899 reflections
$b = 9.3550$ (15) Å	$\theta = 2.7–32.5°$
$c = 11.752$ (2) Å	$\mu = 0.09$ mm^{-1}
$\alpha = 87.526$ (10)°	$T = 90$ K
$\beta = 88.241$ (10)°	Lath, colorless
$\gamma = 86.453$ (9)°	0.32 × 0.15 × 0.04 mm
$V = 686.18$ (19) Å^3	

Data collection

Bruker Kappa APEX-II DUO diffractometer	4973 independent reflections
Radiation source: fine-focus sealed tube	3715 reflections with $I > 2\sigma(I)$
TRIUMPH curved graphite	$R_{int} = 0.032$
ϕ and ω scans	$\theta_{max} = 32.6°$, $\theta_{min} = 1.7°$
Absorption correction: multi-scan *SADABS* (Sheldrick, 2004)	$h = -9 – 9$
$T_{min} = 0.921$, $T_{max} = 0.997$	$k = -14 – 14$
23967 measured reflections	$l = -17 – 17$

Refinement

Refinement on F^2	Primary atom site location: structure-invariant direct methods
Least-squares matrix: full	Secondary atom site location: difference Fourier map
$R[F^2 > 2\sigma(F^2)] = 0.048$	Hydrogen site location: inferred from neighbouring sites
$wR(F^2) = 0.128$	H-atom parameters constrained
$S = 1.02$	$w = 1/[\sigma^2(F_o^2) + (0.0642P)^2 + 0.2257P]$ where $P = (F_o^2 + 2F_c^2)/3$
4973 reflections	$(\Delta/\sigma)_{max} < 0.001$
183 parameters	$\Delta\rangle_{max} = 0.49$ e Å^{-3}
0 restraints	$\Delta\rangle_{min} = -0.30$ e Å^{-3}

Fractional atomic coordinates and isotropic or equivalent isotropic displacement parameters (Å^2)

	x	*y*	*z*	$U_{iso}*/U_{eq}$	
O1	0.93583 (13)	0.29814 (10)	0.22438 (7)	0.01898 (19)	
O2	0.49090 (13)	0.27005 (9)	0.50999 (7)	0.01499 (17)	
O3	0.18233 (14)	0.32718 (10)	0.60465 (8)	0.0221 (2)	
C1	0.75462 (16)	0.22423 (11)	0.22098 (9)	0.01128 (19)	
C2	0.66298 (17)	0.17469 (11)	0.33569 (9)	0.01084 (19)	
C3	0.47526 (18)	0.08789 (12)	0.30187 (9)	0.0145 (2)	
H3A	0.3458	0.1131	0.3490	0.017*	
H3B	0.5120	-0.0161	0.3130	0.017*	
C4	0.43520 (18)	0.12663 (12)	0.17510 (9)	0.0143 (2)	
H4A	0.4096	0.0400	0.1332	0.017*	
H4B	0.3104	0.1962	0.1667	0.017*	
C5	0.63918 (17)	0.19228 (11)	0.13211 (9)	0.01152 (19)	
C6	0.58990 (17)	0.30905 (12)	0.40106 (9)	0.0126 (2)	
H6	0.7180	0.3641	0.4147	0.015*	

C7	0.42456 (19)	0.40745 (12)	0.34482 (10)	0.0149 (2)	
H7	0.4393	0.4508	0.2706	0.018*	
C8	0.25370 (18)	0.42554 (12)	0.41373 (10)	0.0153 (2)	
H8	0.1279	0.4840	0.3975	0.018*	
C9	0.29316 (18)	0.33946 (12)	0.51931 (10)	0.0143 (2)	
C10	0.68822 (17)	0.21143 (11)	0.00925 (9)	0.0119 (2)	
C11	0.89458 (18)	0.18071 (12)	-0.03579 (10)	0.0148 (2)	
H11	1.0073	0.1519	0.0141	0.018*	
C12	0.93669 (19)	0.19180 (13)	-0.15255 (10)	0.0173 (2)	
H12	1.0777	0.1708	-0.1818	0.021*	
C13	0.7735 (2)	0.23343 (12)	-0.22672 (10)	0.0171 (2)	
H13	0.8028	0.2414	-0.3065	0.021*	
C14	0.56732 (19)	0.26327 (12)	-0.18344 (10)	0.0165 (2)	
H14	0.4551	0.2914	-0.2338	0.020*	
C15	0.52489 (19)	0.25210 (12)	-0.06682 (10)	0.0153 (2)	
H15	0.3832	0.2723	-0.0381	0.018*	
C16	0.82849 (19)	0.08570 (13)	0.40663 (10)	0.0177 (2)	
H16A	0.8852	0.0037	0.3635	0.027*	
H16B	0.7604	0.0512	0.4779	0.027*	
H16C	0.9457	0.1453	0.4238	0.027*	
C17	0.9495 (2)	0.42317 (13)	0.15092 (11)	0.0207 (2)	
H17A	1.0199	0.3968	0.0785	0.031*	
H17B	1.0327	0.4930	0.1872	0.031*	
H17C	0.8051	0.4654	0.1367	0.031*	

Atomic displacement parameters (Å^2)

	U^{11}	U^{22}	U^{33}	U^{12}	U^{13}	U^{23}
O1	0.0150 (4)	0.0249 (4)	0.0176 (4)	-0.0084 (3)	-0.0043 (3)	0.0064 (3)
O2	0.0152 (4)	0.0195 (4)	0.0098 (4)	0.0025 (3)	0.0008 (3)	-0.0008

						(3)
O3	0.0201 (4)	0.0301 (5)	0.0155 (4)	0.0019 (4)	0.0050 (3)	-0.0003 (3)
C1	0.0097 (4)	0.0125 (5)	0.0115 (4)	-0.0005 (3)	0.0007 (4)	0.0006 (4)
C2	0.0115 (4)	0.0112 (4)	0.0098 (4)	-0.0002 (3)	-0.0001 (3)	-0.0004 (3)
C3	0.0168 (5)	0.0139 (5)	0.0129 (5)	-0.0042 (4)	0.0012 (4)	-0.0006 (4)
C4	0.0145 (5)	0.0160 (5)	0.0128 (5)	-0.0033 (4)	-0.0009 (4)	-0.0002 (4)
C5	0.0122 (5)	0.0112 (5)	0.0108 (4)	0.0004 (4)	0.0006 (4)	0.0000 (3)
C6	0.0121 (5)	0.0142 (5)	0.0114 (5)	-0.0014 (4)	0.0009 (4)	-0.0011 (4)
C7	0.0190 (5)	0.0123 (5)	0.0130 (5)	0.0006 (4)	0.0010 (4)	0.0006 (4)
C8	0.0155 (5)	0.0162 (5)	0.0139 (5)	0.0025 (4)	-0.0008 (4)	-0.0014 (4)
C9	0.0138 (5)	0.0159 (5)	0.0134 (5)	-0.0002 (4)	0.0002 (4)	-0.0035 (4)
C10	0.0139 (5)	0.0107 (4)	0.0110 (5)	-0.0006 (4)	-0.0004 (4)	-0.0004 (4)
C11	0.0137 (5)	0.0174 (5)	0.0134 (5)	-0.0017 (4)	-0.0009 (4)	-0.0012 (4)
C12	0.0169 (5)	0.0208 (6)	0.0147 (5)	-0.0046 (4)	0.0045 (4)	-0.0031 (4)
C13	0.0245 (6)	0.0170 (5)	0.0103 (5)	-0.0069 (4)	0.0008 (4)	0.0002 (4)
C14	0.0209 (6)	0.0154 (5)	0.0131 (5)	-0.0016 (4)	-0.0048 (4)	0.0019 (4)
C15	0.0152 (5)	0.0160 (5)	0.0144 (5)	0.0008 (4)	-0.0009 (4)	0.0004 (4)
C16	0.0177 (5)	0.0192 (5)	0.0154 (5)	0.0042 (4)	-0.0010 (4)	0.0023 (4)
C17	0.0208 (6)	0.0167 (5)	0.0251 (6)	-0.0069 (4)	-0.0012 (5)	0.0013 (5)

Geometric parameters (Å, º)

O1—C1	1.3672 (13)	C7—H7	0.9500
O1—C17	1.4288 (15)	C8—C9	1.4687 (16)
O2—C9	1.3657 (14)	C8—H8	0.9500
O2—C6	1.4474 (13)	C10—C11	1.3992 (16)

O3—C9	1.2074 (14)	C10—C15	1.4031 (15)
C1—C5	1.3426 (15)	C11—C12	1.3900 (16)
C1—C2	1.5142 (15)	C11—H11	0.9500
C2—C16	1.5325 (16)	C12—C13	1.3898 (17)
C2—C6	1.5395 (15)	C12—H12	0.9500
C2—C3	1.5407 (15)	C13—C14	1.3896 (17)
C3—C4	1.5424 (16)	C13—H13	0.9500
C3—H3A	0.9900	C14—C15	1.3888 (16)
C3—H3B	0.9900	C14—H14	0.9500
C4—C5	1.5148 (15)	C15—H15	0.9500
C4—H4A	0.9900	C16—H16A	0.9800
C4—H4B	0.9900	C16—H16B	0.9800
C5—C10	1.4725 (15)	C16—H16C	0.9800
C6—C7	1.4925 (16)	C17—H17A	0.9800
C6—H6	1.0000	C17—H17B	0.9800
C7—C8	1.3287 (16)	C17—H17C	0.9800
C1—O1—C17	117.54 (9)	C7—C8—H8	125.9
C9—O2—C6	109.42 (8)	C9—C8—H8	125.9
C5—C1—O1	130.39 (10)	O3—C9—O2	121.54 (11)
C5—C1—C2	114.14 (9)	O3—C9—C8	129.94 (11)
O1—C1—C2	115.43 (9)	O2—C9—C8	108.51 (9)
C1—C2—C16	112.23 (9)	C11—C10—C15	118.12 (10)
C1—C2—C6	107.72 (9)	C11—C10—C5	121.29 (10)
C16—C2—C6	108.42 (9)	C15—C10—C5	120.45 (10)
C1—C2—C3	102.29 (8)	C12—C11—C10	120.84 (10)
C16—C2—C3	113.01 (9)	C12—C11—H11	119.6
C6—C2—C3	113.01 (9)	C10—C11—H11	119.6
C2—C3—C4	106.67 (9)	C13—C12—C11	120.34 (11)
C2—C3—H3A	110.4	C13—C12—H12	119.8
C4—C3—H3A	110.4	C11—C12—H12	119.8
C2—C3—H3B	110.4	C14—C13—C12	119.55 (10)
C4—C3—H3B	110.4	C14—C13—H13	120.2
H3A—C3—H3B	108.6	C12—C13—H13	120.2
C5—C4—C3	104.17 (9)	C15—C14—C13	120.19 (11)
C5—C4—H4A	110.9	C15—C14—H14	119.9
C3—C4—H4A	110.9	C13—C14—H14	119.9
C5—C4—H4B	110.9	C14—C15—C10	120.95 (11)
C3—C4—H4B	110.9	C14—C15—H15	119.5
H4A—C4—H4B	108.9	C10—C15—H15	119.5
C1—C5—C10	129.27 (10)	C2—C16—H16A	109.5
C1—C5—C4	109.57 (9)	C2—C16—H16B	109.5

C10—C5—C4	121.16 (9)	H16A—C16—H16B	109.5
O2—C6—C7	103.90 (9)	C2—C16—H16C	109.5
O2—C6—C2	110.91 (9)	H16A—C16—H16C	109.5
C7—C6—C2	115.68 (9)	H16B—C16—H16C	109.5
O2—C6—H6	108.7	O1—C17—H17A	109.5
C7—C6—H6	108.7	O1—C17—H17B	109.5
C2—C6—H6	108.7	H17A—C17—H17B	109.5
C8—C7—C6	110.04 (10)	O1—C17—H17C	109.5
C8—C7—H7	125.0	H17A—C17—H17C	109.5
C6—C7—H7	125.0	H17B—C17—H17C	109.5
C7—C8—C9	108.11 (10)		
C17—O1—C1—C5	40.44 (17)	C1—C2—C6—C7	59.34 (12)
C17—O1—C1—C2	-136.92 (10)	C16—C2—C6—C7	-178.98 (9)
C5—C1—C2—C16	127.56 (11)	C3—C2—C6—C7	-52.89 (12)
O1—C1—C2—C16	-54.64 (12)	O2—C6—C7—C8	1.29 (12)
C5—C1—C2—C6	-113.16 (10)	C2—C6—C7—C8	123.09 (11)
O1—C1—C2—C6	64.64 (11)	C6—C7—C8—C9	-0.62 (13)
C5—C1—C2—C3	6.16 (12)	C6—O2—C9—O3	-177.68 (10)
O1—C1—C2—C3	-176.05 (9)	C6—O2—C9—C8	1.18 (12)
C1—C2—C3—C4	-14.75 (11)	C7—C8—C9—O3	178.40 (12)
C16—C2—C3—C4	-135.63 (10)	C7—C8—C9—O2	-0.34 (13)
C6—C2—C3—C4	100.78 (10)	C1—C5—C10—C11	41.28 (17)
C2—C3—C4—C5	17.94 (11)	C4—C5—C10—C11	-137.88 (11)
O1—C1—C5—C10	8.7 (2)	C1—C5—C10—C15	-142.99 (12)
C2—C1—C5—C10	-173.87 (10)	C4—C5—C10—C15	37.85 (15)
O1—C1—C5—C4	-172.01 (11)	C15—C10—C11—C12	0.70 (16)
C2—C1—C5—C4	5.37 (13)	C5—C10—C11—C12	176.53 (10)
C3—C4—C5—C1	-14.54 (12)	C10—C11—C12—C13	-0.16 (17)
C3—C4—C5—C10	164.78 (9)	C11—C12—C13—C14	-0.33 (17)
C9—O2—C6—C7	-1.49 (11)	C12—C13—C14—C15	0.26 (17)
C9—O2—C6—C2	-126.41 (9)	C13—C14—C15—C10	0.30 (17)
C1—C2—C6—O2	177.32 (8)	C11—C10—C15—C14	-0.78 (16)
C16—C2—C6—O2	-61.00 (11)	C5—C10—C15—C14	-176.64 (10)

C3—C2—C6—O2	65.08 (11)		

(±)-*2-(1H-indol-3-yl)-2-methyl-5-phenylcyclopentanone* (29)

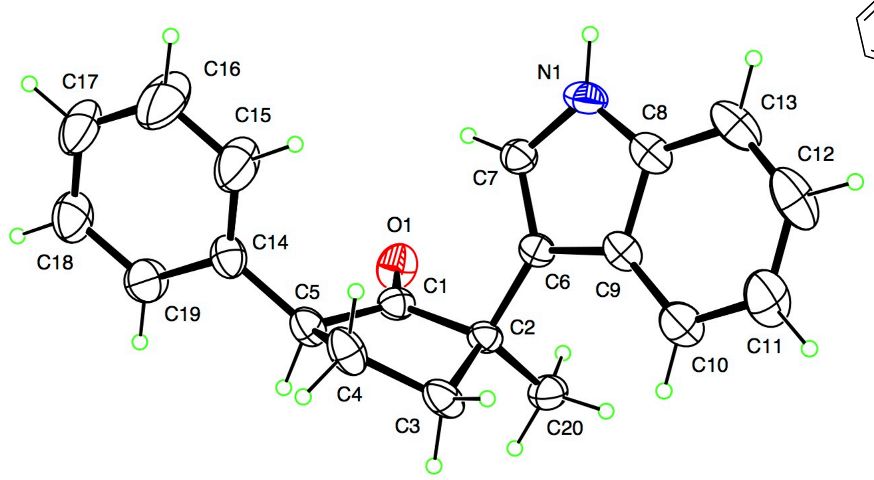

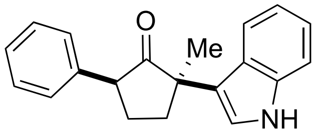

29

Crystal data

$C_{20}H_{19}NO$	D_x = 1.204 Mg m^{-3}
M_r = 306.60	Mo *K*α radiation, λ = 0.71073 Å
Trigonal, $R\bar{3}$	Cell parameters from 3430 reflections
Hall symbol: -R 3	θ = 2.4–26.4°
a = 25.0795 (18) Å	μ = 0.07 mm^{-1}
c = 13.9752 (13) Å	*T* = 135 K
V = 7612.5 (10) Å^3	Fragment, colourless
Z = 18	0.30 × 0.21 × 0.09 mm
F(000) = 2952	

Data collection

Bruker Kappa APEX-II DUO diffractometer	3678 independent reflections
Radiation source: fine-focus sealed tube	2685 reflections with $I > 2\sigma(I)$
TRIUMPH curved graphite	R_{int} = 0.033
ϕ and ω scans	θ_{max} = 27.1°, θ_{min} = 1.6°
Absorption correction: multi-scan *SADABS* (Sheldrick, 2004)	*h* = -31→20
T_{min} = 0.879, T_{max} = 0.993	*k* = -18→31

12232 measured reflections	$l = -15 \rightarrow 17$

Refinement

Refinement on F^2	Primary atom site location: structure-invariant direct methods
Least-squares matrix: full	Secondary atom site location: difference Fourier map
$R[F^2 > 2\sigma(F^2)] = 0.048$	Hydrogen site location: inferred from neighbouring sites
$wR(F^2) = 0.126$	H atoms treated by a mixture of independent and constrained refinement
$S = 1.07$	$w = 1/[\sigma^2(F_o^2) + (0.0571P)^2 + 4.8033P]$ where $P = (F_o^2 + 2F_c^2)/3$
3678 reflections	$(\Delta/\sigma)_{max} < 0.001$
203 parameters	$\Delta\rangle_{max} = 0.34$ e Å^{-3}
0 restraints	$\Delta\rangle_{min} = -0.32$ e Å^{-3}

Fractional atomic coordinates and isotropic or equivalent isotropic displacement parameters (Å^2)

	x	*y*	*z*	U_{iso}*/U_{eq}	
O1	0.13148 (5)	0.75077 (5)	0.75722 (7)	0.0357 (3)	
N1	0.05284 (6)	0.54941 (6)	0.71803 (9)	0.0321 (3)	
H1N	0.0617 (8)	0.5341 (8)	0.6669 (12)	0.038*	
C1	0.12714 (7)	0.72767 (6)	0.83534 (10)	0.0255 (3)	
C2	0.06738 (6)	0.67531 (6)	0.87654 (9)	0.0238 (3)	
C3	0.08496 (7)	0.67388 (7)	0.98231 (10)	0.0322 (4)	
H3A	0.0775	0.7023	1.0217	0.039*	
H3B	0.0608	0.6318	1.0090	0.039*	
C4	0.15323 (7)	0.69441 (7)	0.97971 (11)	0.0356 (4)	
H4A	0.1599	0.6606	0.9582	0.043*	
H4B	0.1724	0.7088	1.0434	0.043*	
C5	0.17933 (7)	0.74747 (7)	0.90695 (11)	0.0298 (3)	
H5	0.1825	0.7839	0.9414	0.036*	
C6	0.05331 (6)	0.61658 (6)	0.82517 (9)	0.0232 (3)	
C7	0.08105 (7)	0.61003 (7)	0.74526 (10)	0.0271 (3)	
H7	0.1150	0.6428	0.7132	0.032*	
C8	0.00532 (7)	0.51525 (7)	0.77986 (10)	0.0298 (3)	

C9	0.00376 (7)	0.55580 (6)	0.84897 (9)	0.0264 (3)	
C10	-0.04299 (8)	0.53159 (7)	0.91799 (11)	0.0373 (4)	
H10	-0.0450	0.5575	0.9658	0.045*	
C11	-0.08609 (9)	0.46969 (8)	0.91572 (13)	0.0487 (5)	
H11	-0.1182	0.4531	0.9620	0.058*	
C12	-0.08331 (9)	0.43075 (8)	0.84605 (12)	0.0487 (5)	
H12	-0.1138	0.3883	0.8458	0.058*	
C13	-0.03781 (8)	0.45254 (7)	0.77874 (12)	0.0393 (4)	
H13	-0.0356	0.4259	0.7327	0.047*	
C14	0.24191 (7)	0.76832 (7)	0.86514 (11)	0.0315 (4)	
C15	0.25418 (9)	0.72863 (9)	0.8152 (2)	0.0789 (9)	
H15	0.2225	0.6871	0.8065	0.095*	
C16	0.31172 (10)	0.74794 (11)	0.7773 (3)	0.0972 (11)	
H16	0.3190	0.7195	0.7430	0.117*	
C17	0.35865 (8)	0.80755 (9)	0.78824 (16)	0.0535 (5)	
H17	0.3986	0.8203	0.7635	0.064*	
C18	0.34667 (8)	0.84780 (8)	0.83529 (14)	0.0455 (4)	
H18	0.3782	0.8895	0.8424	0.055*	
C19	0.28847 (8)	0.82830 (7)	0.87306 (13)	0.0421 (4)	
H19	0.2808	0.8572	0.9051	0.050*	
C20	0.01443 (7)	0.68826 (7)	0.86432 (11)	0.0324 (4)	
H20A	0.0095	0.6944	0.7963	0.049*	
H20B	0.0235	0.7254	0.9002	0.049*	
H20C	-0.0237	0.6533	0.8886	0.049*	

Atomic displacement parameters (Å^2)

	U^{11}	U^{22}	U^{33}	U^{12}	U^{13}	U^{23}
O1	0.0348 (6)	0.0358 (6)	0.0313 (6)	0.0136 (5)	0.0056 (5)	0.0138 (5)
N1	0.0391 (8)	0.0363 (7)	0.0298 (7)	0.0256 (6)	-0.0035 (6)	-0.0096 (6)
C1	0.0300 (8)	0.0208 (7)	0.0263 (7)	0.0130 (6)	0.0039 (6)	0.0008 (6)
C2	0.0272 (7)	0.0221 (7)	0.0191 (7)	0.0101 (6)	0.0030 (5)	0.0002 (5)
C3	0.0419 (9)	0.0251 (8)	0.0196 (7)	0.0092 (7)	0.0007 (6)	-0.0007 (6)

C4	0.0405 (9)	0.0298 (8)	0.0268 (8)	0.0103 (7)	-0.0093 (7)	0.0007 (6)
C5	0.0314 (8)	0.0229 (7)	0.0297 (8)	0.0094 (6)	-0.0025 (6)	-0.0024 (6)
C6	0.0270 (7)	0.0241 (7)	0.0197 (6)	0.0136 (6)	-0.0014 (5)	0.0002 (5)
C7	0.0276 (8)	0.0297 (8)	0.0273 (7)	0.0168 (7)	-0.0026 (6)	-0.0032 (6)
C8	0.0398 (9)	0.0268 (8)	0.0285 (7)	0.0210 (7)	-0.0103 (6)	-0.0022 (6)
C9	0.0346 (8)	0.0229 (7)	0.0212 (7)	0.0141 (6)	-0.0052 (6)	0.0015 (5)
C10	0.0466 (10)	0.0289 (8)	0.0262 (8)	0.0112 (8)	0.0021 (7)	0.0036 (6)
C11	0.0534 (11)	0.0336 (9)	0.0356 (9)	0.0042 (8)	0.0028 (8)	0.0120 (7)
C12	0.0664 (13)	0.0211 (8)	0.0406 (10)	0.0083 (8)	-0.0164 (9)	0.0060 (7)
C13	0.0622 (11)	0.0239 (8)	0.0355 (9)	0.0242 (8)	-0.0177 (8)	-0.0038 (7)
C14	0.0299 (8)	0.0253 (8)	0.0374 (8)	0.0125 (7)	-0.0052 (6)	-0.0004 (6)
C15	0.0368 (11)	0.0337 (11)	0.155 (3)	0.0092 (9)	0.0163 (13)	-0.0280 (13)
C16	0.0462 (13)	0.0457 (13)	0.198 (3)	0.0219 (11)	0.0274 (16)	-0.0310 (17)
C17	0.0291 (9)	0.0448 (11)	0.0901 (16)	0.0211 (9)	0.0039 (9)	0.0035 (10)
C18	0.0356 (9)	0.0307 (9)	0.0589 (11)	0.0081 (8)	0.0036 (8)	0.0048 (8)
C19	0.0406 (10)	0.0270 (8)	0.0515 (10)	0.0116 (8)	0.0089 (8)	0.0004 (7)
C20	0.0325 (8)	0.0286 (8)	0.0367 (8)	0.0156 (7)	0.0062 (7)	-0.0020 (6)

Geometric parameters (Å, º)

O1—C1	1.2151 (17)	C9—C10	1.401 (2)
N1—C8	1.371 (2)	C10—C11	1.379 (2)
N1—C7	1.3714 (19)	C10—H10	0.9500
N1—H1N	0.891 (17)	C11—C12	1.405 (3)
C1—C5	1.520 (2)	C11—H11	0.9500
C1—C2	1.5277 (19)	C12—C13	1.364 (3)
C2—C6	1.5132 (19)	C12—H12	0.9500
C2—C20	1.526 (2)	C13—H13	0.9500

C2—C3	1.5481 (19)	C14—C15	1.371 (3)
C3—C4	1.522 (2)	C14—C19	1.372 (2)
C3—H3A	0.9900	C15—C16	1.378 (3)
C3—H3B	0.9900	C15—H15	0.9500
C4—C5	1.537 (2)	C16—C17	1.373 (3)
C4—H4A	0.9900	C16—H16	0.9500
C4—H4B	0.9900	C17—C18	1.358 (3)
C5—C14	1.503 (2)	C17—H17	0.9500
C5—H5	1.0000	C18—C19	1.391 (2)
C6—C7	1.3684 (19)	C18—H18	0.9500
C6—C9	1.444 (2)	C19—H19	0.9500
C7—H7	0.9500	C20—H20A	0.9800
C8—C13	1.394 (2)	C20—H20B	0.9800
C8—C9	1.417 (2)	C20—H20C	0.9800
C8—N1—C7	108.85 (12)	C10—C9—C8	118.40 (14)
C8—N1—H1N	124.2 (11)	C10—C9—C6	135.11 (14)
C7—N1—H1N	126.8 (11)	C8—C9—C6	106.42 (13)
O1—C1—C5	125.34 (13)	C11—C10—C9	119.17 (16)
O1—C1—C2	124.33 (13)	C11—C10—H10	120.4
C5—C1—C2	110.31 (12)	C9—C10—H10	120.4
C6—C2—C20	110.48 (12)	C10—C11—C12	121.00 (17)
C6—C2—C1	108.00 (11)	C10—C11—H11	119.5
C20—C2—C1	111.04 (12)	C12—C11—H11	119.5
C6—C2—C3	111.15 (11)	C13—C12—C11	121.42 (16)
C20—C2—C3	113.58 (12)	C13—C12—H12	119.3
C1—C2—C3	102.20 (11)	C11—C12—H12	119.3
C4—C3—C2	104.75 (12)	C12—C13—C8	117.78 (16)
C4—C3—H3A	110.8	C12—C13—H13	121.1
C2—C3—H3A	110.8	C8—C13—H13	121.1
C4—C3—H3B	110.8	C15—C14—C19	117.40 (16)
C2—C3—H3B	110.8	C15—C14—C5	121.34 (15)
H3A—C3—H3B	108.9	C19—C14—C5	121.23 (14)
C3—C4—C5	103.22 (12)	C14—C15—C16	121.10 (18)
C3—C4—H4A	111.1	C14—C15—H15	119.4
C5—C4—H4A	111.1	C16—C15—H15	119.4
C3—C4—H4B	111.1	C17—C16—C15	121.1 (2)
C5—C4—H4B	111.1	C17—C16—H16	119.4
H4A—C4—H4B	109.1	C15—C16—H16	119.4
C14—C5—C1	115.89 (12)	C18—C17—C16	118.41 (18)
C14—C5—C4	118.49 (13)	C18—C17—H17	120.8
C1—C5—C4	103.20 (12)	C16—C17—H17	120.8
C14—C5—H5	106.1	C17—C18—C19	120.37 (17)
C1—C5—H5	106.1	C17—C18—H18	119.8

C4—C5—H5	106.1	C19—C18—H18	119.8
C7—C6—C9	106.39 (12)	C14—C19—C18	121.54 (16)
C7—C6—C2	128.24 (13)	C14—C19—H19	119.2
C9—C6—C2	125.25 (12)	C18—C19—H19	119.2
C6—C7—N1	110.37 (13)	C2—C20—H20A	109.5
C6—C7—H7	124.8	C2—C20—H20B	109.5
N1—C7—H7	124.8	H20A—C20—H20B	109.5
N1—C8—C13	129.77 (15)	C2—C20—H20C	109.5
N1—C8—C9	107.96 (13)	H20A—C20—H20C	109.5
C13—C8—C9	122.21 (15)	H20B—C20—H20C	109.5
O1—C1—C2—C6	74.14 (17)	N1—C8—C9—C10	177.37 (13)
C5—C1—C2—C6	-107.39 (13)	C13—C8—C9—C10	-0.2 (2)
O1—C1—C2—C20	-47.13 (18)	N1—C8—C9—C6	-0.05 (15)
C5—C1—C2—C20	131.34 (12)	C13—C8—C9—C6	-177.61 (13)
O1—C1—C2—C3	-168.58 (14)	C7—C6—C9—C10	-176.34 (16)
C5—C1—C2—C3	9.90 (15)	C2—C6—C9—C10	-0.1 (3)
C6—C2—C3—C4	83.72 (14)	C7—C6—C9—C8	0.45 (15)
C20—C2—C3—C4	-150.95 (12)	C2—C6—C9—C8	176.72 (12)
C1—C2—C3—C4	-31.28 (14)	C8—C9—C10—C11	-0.8 (2)
C2—C3—C4—C5	41.31 (14)	C6—C9—C10—C11	175.71 (17)
O1—C1—C5—C14	-35.6 (2)	C9—C10—C11—C12	0.7 (3)
C2—C1—C5—C14	145.97 (12)	C10—C11—C12—C13	0.5 (3)
O1—C1—C5—C4	-166.76 (14)	C11—C12—C13—C8	-1.4 (3)
C2—C1—C5—C4	14.79 (15)	N1—C8—C13—C12	-175.71 (15)
C3—C4—C5—C14	-163.57 (13)	C9—C8—C13—C12	1.3 (2)
C3—C4—C5—C1	-33.95 (15)	C1—C5—C14—C15	-64.5 (2)

C20—C2—C6—C7	111.22 (16)	C4—C5—C14—C15	59.0 (2)
C1—C2—C6—C7	-10.40 (19)	C1—C5—C14—C19	113.49 (17)
C3—C2—C6—C7	-121.74 (15)	C4—C5—C14—C19	-122.99 (17)
C20—C2—C6—C9	-64.22 (17)	C19—C14—C15—C16	2.1 (4)
C1—C2—C6—C9	174.17 (13)	C5—C14—C15—C16	-179.8 (3)
C3—C2—C6—C9	62.82 (18)	C14—C15—C16—C17	0.0 (5)
C9—C6—C7—N1	-0.69 (16)	C15—C16—C17—C18	-1.8 (4)
C2—C6—C7—N1	-176.81 (13)	C16—C17—C18—C19	1.4 (3)
C8—N1—C7—C6	0.68 (16)	C15—C14—C19—C18	-2.5 (3)
C7—N1—C8—C13	176.94 (15)	C5—C14—C19—C18	179.42 (16)
C7—N1—C8—C9	-0.37 (16)	C17—C18—C19—C14	0.7 (3)

Hydrogen-bond geometry (Å, º)

D—H···*A*	*D*—H	H···*A*	*D*···*A*	*D*—H···*A*
N1—H1*N*···O1^{i}	0.891 (17)	2.033 (18)	2.8570 (16)	153.4 (15)

Symmetry code: (i) *x*-*y*+2/3, *x*+1/3, -*z*+4/3.

(±)-*3-((1S,2R,3S)-2-methoxy-1-methyl-3-phenylcyclopentyl)-1H-indole* (**30**)

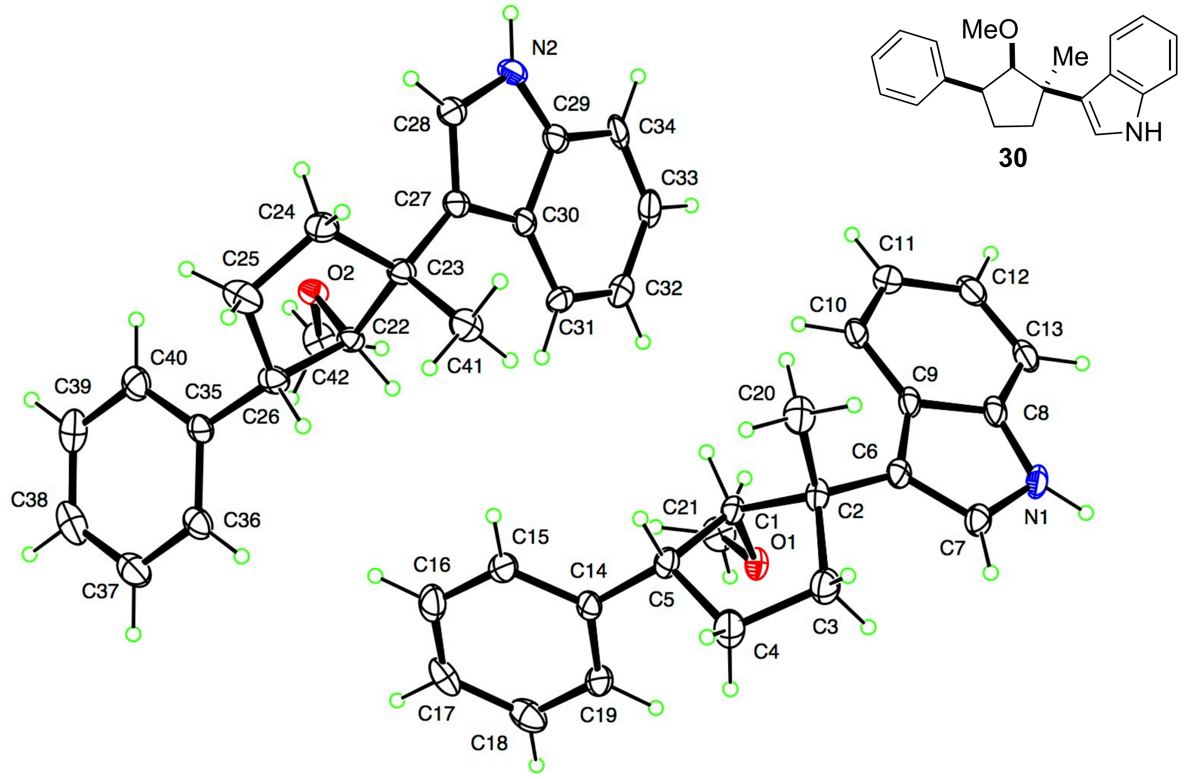

30

Crystal data

$C_{21}H_{23}NO$	$Z = 4$
$M_r = 305.40$	$F(000) = 656$
Triclinic, $P\bar{1}$	$D_x = 1.212$ Mg m^{-3}
Hall symbol: -P 1	Mo $K\alpha$ radiation, $\lambda = 0.71073$ Å
$a = 12.0196$ (11) Å	Cell parameters from 6801 reflections
$b = 12.4522$ (11) Å	$\theta = 2.9$–26.3°
$c = 14.0202$ (14) Å	$\mu = 0.07$ mm^{-1}
$\alpha = 94.894$ (4)°	$T = 90$ K
$\beta = 114.290$ (4)°	Needle, colourless
$\gamma = 113.544$ (4)°	0.18 × 0.12 × 0.10 mm
$V = 1673.4$ (3) Å^3	

Data collection

Bruker Kappa APEX-II DUO diffractometer	20352 independent reflections
Radiation source: fine-focus sealed tube	14935 reflections with $I > 2\sigma(I)$
TRIUMPH curved graphite	$R_{int} = 0.051$

ϕ and ω scans	θ_{max} = 26.5°, θ_{min} = 1.7°
Absorption correction: multi-scan *SADABS* (Sheldrick, 2004)	*h* = -15 – 15
T_{min} = 0.940, T_{max} = 0.993	*k* = -15 – 15
20648 measured reflections	*l* = -17 – 17

Refinement

Refinement on F^2	Primary atom site location: structure-invariant direct methods
Least-squares matrix: full	Secondary atom site location: difference Fourier map
$R[F^2 > 2\sigma(F^2)]$ = 0.074	Hydrogen site location: inferred from neighbouring sites
$wR(F^2)$ = 0.217	H atoms treated by a mixture of independent and constrained refinement
S = 1.03	$w = 1/[\sigma^2(F_o^2) + (0.1362P)^2 + 0.2759P]$ where $P = (F_o^2 + 2F_c^2)/3$
20352 reflections	$(\Delta/\sigma)_{max}$ = 0.001
426 parameters	$\Delta\rangle_{max}$ = 0.65 e Å^{-3}
0 restraints	$\Delta\rangle_{min}$ = -0.41 e Å^{-3}

Refinement. Refinement of F^2 against ALL reflections, using a HKLF 5 file prepared by ROTAX. The crystal is twinned by twofold rotation about reciprocal 0 1 0. The BASF parameter refined to 0.224 (2).

Fractional atomic coordinates and isotropic or equivalent isotropic displacement parameters (Å^2)

	x	*y*	*z*	U_{iso}*/U_{eq}	
O1	0.61829 (11)	0.17554 (10)	0.37206 (9)	0.0170 (3)	
N1	0.48670 (16)	0.06857 (14)	0.61515 (12)	0.0211 (3)	
H1N	0.4608 (19)	-0.0058 (17)	0.6293 (15)	0.025*	
C1	0.56680 (17)	0.26201 (15)	0.36260 (13)	0.0156 (4)	
H1	0.6438	0.3471	0.4076	0.019*	
C2	0.45279 (18)	0.22411 (15)	0.39711 (14)	0.0184 (4)	
C3	0.32442 (18)	0.11983 (16)	0.29429 (15)	0.0247 (4)	

H3A	0.3146	0.0389	0.3028	0.030*	
H3B	0.2389	0.1224	0.2835	0.030*	
C4	0.34723 (18)	0.13978 (17)	0.19592 (15)	0.0268 (4)	
H4A	0.2729	0.1527	0.1417	0.032*	
H4B	0.3463	0.0674	0.1597	0.032*	
C5	0.49032 (17)	0.25470 (15)	0.24100 (14)	0.0181 (4)	
H5	0.4742	0.3275	0.2388	0.022*	
C6	0.49358 (17)	0.18467 (15)	0.49983 (14)	0.0171 (4)	
C7	0.42118 (18)	0.07348 (15)	0.50993 (15)	0.0204 (4)	
H7	0.3367	0.0081	0.4520	0.024*	
C8	0.60802 (19)	0.17693 (15)	0.67508 (15)	0.0192 (4)	
C9	0.61530 (18)	0.25386 (15)	0.60647 (14)	0.0178 (4)	
C10	0.72979 (18)	0.37149 (15)	0.65151 (14)	0.0200 (4)	
H10	0.7372	0.4260	0.6083	0.024*	
C11	0.8320 (2)	0.40783 (16)	0.75923 (15)	0.0233 (4)	
H11	0.9104	0.4873	0.7892	0.028*	
C12	0.8222 (2)	0.32960 (16)	0.82523 (15)	0.0244 (4)	
H12	0.8937	0.3570	0.8992	0.029*	
C13	0.7104 (2)	0.21381 (16)	0.78398 (14)	0.0231 (4)	
H13	0.7032	0.1606	0.8284	0.028*	
C14	0.56763 (17)	0.25727 (15)	0.17868 (13)	0.0171 (4)	
C15	0.62876 (18)	0.36394 (15)	0.15253 (13)	0.0205 (4)	
H15	0.6184	0.4328	0.1727	0.025*	
C16	0.7040 (2)	0.37153 (17)	0.09798 (15)	0.0271 (4)	
H16	0.7452	0.4454	0.0821	0.033*	
C17	0.7191 (2)	0.27232 (18)	0.06665 (14)	0.0290 (4)	
H17	0.7700	0.2773	0.0288	0.035*	
C18	0.6589 (2)	0.16473	0.09105	0.0283 (4)	

		(18)	(15)		
H18	0.6690	0.0960	0.0700	0.034*	
C19	0.58385 (19)	0.15787 (16)	0.14636 (14)	0.0221 (4)	
H19	0.5430	0.0840	0.1623	0.027*	
C20	0.42630 (19)	0.33308 (16)	0.41746 (15)	0.0248 (4)	
H20A	0.5122	0.4026	0.4764	0.037*	
H20B	0.3536	0.3089	0.4387	0.037*	
H20C	0.3963	0.3569	0.3502	0.037*	
C21	0.76476 (18)	0.23114 (17)	0.41818 (15)	0.0240 (4)	
H21A	0.8091	0.2770	0.4955	0.036*	
H21B	0.7943	0.2874	0.3783	0.036*	
H21C	0.7921	0.1675	0.4126	0.036*	
O2	0.92748 (11)	0.82071 (10)	0.27949 (9)	0.0168 (3)	
N2	0.91489 (15)	0.93003 (13)	0.58410 (12)	0.0197 (3)	
H2N	0.9677 (19)	1.0001 (16)	0.6409 (16)	0.024*	
C22	0.78129 (16)	0.73850 (15)	0.22465 (13)	0.0149 (4)	
H22	0.7625	0.6523	0.2225	0.018*	
C23	0.71312 (17)	0.77953 (14)	0.28288 (14)	0.0166 (4)	
C24	0.69970 (19)	0.88508 (15)	0.23735 (14)	0.0214 (4)	
H24A	0.6145	0.8862	0.2294	0.026*	
H24B	0.7809	0.9651	0.2878	0.026*	
C25	0.6928 (2)	0.86245 (17)	0.12605 (14)	0.0273 (4)	
H25A	0.7677	0.9343	0.1251	0.033*	
H25B	0.6022	0.8488	0.0675	0.033*	
C26	0.71035 (18)	0.74762 (15)	0.10734 (13)	0.0189 (4)	
H26	0.6151	0.6751	0.0667	0.023*	
C27	0.79869 (17)	0.81770 (15)	0.40620 (14)	0.0165 (4)	
C28	0.84417 (17)	0.92655 (15)	0.47728 (14)	0.0186 (4)	
H28	0.8288	0.9918	0.4555	0.022*	
C29	0.92118	0.82249	0.58455	0.0187 (4)	

	(17)	(15)	(14)		
C30	0.84683 (17)	0.74793 (15)	0.47409 (13)	0.0167 (4)	
C31	0.83839 (18)	0.63131 (15)	0.45428 (14)	0.0192 (4)	
H31	0.7880	0.5781	0.3815	0.023*	
C32	0.90383 (18)	0.59527 (16)	0.54132 (15)	0.0235 (4)	
H32	0.8999	0.5175	0.5277	0.028*	
C33	0.97605 (19)	0.67088 (16)	0.64955 (15)	0.0251 (4)	
H33	1.0191	0.6430	0.7080	0.030*	
C34	0.98566 (18)	0.78497 (16)	0.67267 (14)	0.0222 (4)	
H34	1.0345	0.8363	0.7460	0.027*	
C35	0.78342 (18)	0.74471 (15)	0.04207 (13)	0.0174 (4)	
C36	0.72642 (19)	0.63856 (16)	-0.04105 (14)	0.0209 (4)	
H36	0.6418	0.5702	-0.0563	0.025*	
C37	0.7902 (2)	0.62998 (18)	-0.10230 (15)	0.0277 (4)	
H37	0.7491	0.5567	-0.1590	0.033*	
C38	0.9143 (2)	0.72895 (19)	-0.08030 (16)	0.0317 (5)	
H38	0.9588	0.7235	-0.1216	0.038*	
C39	0.9727 (2)	0.83516 (18)	0.00178 (16)	0.0294 (5)	
H39	1.0575	0.9031	0.0169	0.035*	
C40	0.90812 (19)	0.84353 (16)	0.06269 (14)	0.0235 (4)	
H40	0.9493	0.9173	0.1189	0.028*	
C41	0.56731 (17)	0.67340 (15)	0.24616 (14)	0.0218 (4)	
H41A	0.5245	0.6990	0.2832	0.033*	
H41B	0.5752	0.6019	0.2653	0.033*	
H41C	0.5095	0.6518	0.1666	0.033*	
C42	1.00849 (18)	0.76069 (17)	0.28559 (15)	0.0238 (4)	
H42A	0.9770	0.7134	0.2116	0.036*	
H42B	0.9975	0.7053	0.3306	0.036*	
H42C	1.1066	0.8222	0.3188	0.036*	

Atomic displacement parameters (Å^2)

	U^{11}	U^{22}	U^{33}	U^{12}	U^{13}	U^{23}
O1	0.0174 (6)	0.0200 (6)	0.0204 (6)	0.0112 (5)	0.0116 (5)	0.0120 (5)
N1	0.0274 (9)	0.0185 (8)	0.0293 (9)	0.0121 (7)	0.0216 (8)	0.0143 (7)
C1	0.0173 (9)	0.0152 (8)	0.0190 (9)	0.0091 (7)	0.0111 (8)	0.0085 (7)
C2	0.0190 (9)	0.0200 (9)	0.0223 (9)	0.0098 (8)	0.0138 (8)	0.0113 (8)
C3	0.0218 (10)	0.0255 (10)	0.0256 (10)	0.0081 (8)	0.0133 (9)	0.0095 (8)
C4	0.0193 (10)	0.0306 (10)	0.0208 (10)	0.0058 (9)	0.0071 (8)	0.0096 (8)
C5	0.0198 (9)	0.0173 (9)	0.0186 (9)	0.0091 (8)	0.0096 (8)	0.0094 (7)
C6	0.0192 (9)	0.0178 (9)	0.0246 (10)	0.0111 (8)	0.0168 (8)	0.0088 (7)
C7	0.0206 (9)	0.0202 (9)	0.0263 (10)	0.0097 (8)	0.0161 (8)	0.0092 (8)
C8	0.0270 (10)	0.0209 (9)	0.0258 (10)	0.0156 (8)	0.0215 (9)	0.0118 (8)
C9	0.0252 (10)	0.0189 (9)	0.0242 (9)	0.0146 (8)	0.0197 (8)	0.0123 (8)
C10	0.0271 (10)	0.0208 (9)	0.0224 (9)	0.0127 (8)	0.0191 (8)	0.0098 (8)
C11	0.0285 (10)	0.0197 (9)	0.0241 (10)	0.0105 (8)	0.0159 (9)	0.0057 (8)
C12	0.0353 (11)	0.0284 (10)	0.0183 (9)	0.0186 (9)	0.0168 (9)	0.0083 (8)
C13	0.0381 (11)	0.0269 (10)	0.0227 (10)	0.0220 (9)	0.0229 (9)	0.0142 (8)
C14	0.0161 (9)	0.0194 (9)	0.0113 (8)	0.0069 (8)	0.0041 (7)	0.0065 (7)
C15	0.0233 (10)	0.0184 (9)	0.0149 (9)	0.0099 (8)	0.0050 (8)	0.0070 (7)
C16	0.0273 (11)	0.0293 (10)	0.0202 (10)	0.0095 (9)	0.0106 (9)	0.0130 (8)
C17	0.0329 (11)	0.0435 (12)	0.0159 (9)	0.0202 (10)	0.0141 (9)	0.0117 (9)
C18	0.0355 (12)	0.0365 (11)	0.0184 (10)	0.0247 (10)	0.0106 (9)	0.0086 (9)
C19	0.0284 (10)	0.0193 (9)	0.0176 (9)	0.0117 (8)	0.0093 (8)	0.0098 (7)
C20	0.0276 (10)	0.0282 (10)	0.0318 (11)	0.0186 (9)	0.0194 (9)	0.0148 (9)
C21	0.0199	0.0293	0.0264	0.0150 (8)	0.0110 (8)	0.0095 (8)

	(10)	(10)	(10)			
O2	0.0128 (6)	0.0183 (6)	0.0156 (6)	0.0059 (5)	0.0060 (5)	0.0012 (5)
N2	0.0196 (8)	0.0192 (8)	0.0163 (8)	0.0049 (7)	0.0104 (7)	-0.0002 (6)
C22	0.0128 (8)	0.0150 (8)	0.0129 (8)	0.0051 (7)	0.0047 (7)	0.0018 (7)
C23	0.0180 (9)	0.0162 (9)	0.0170 (9)	0.0088 (7)	0.0094 (8)	0.0034 (7)
C24	0.0235 (10)	0.0207 (9)	0.0226 (9)	0.0127 (8)	0.0116 (8)	0.0052 (8)
C25	0.0390 (12)	0.0342 (11)	0.0181 (9)	0.0290 (10)	0.0105 (9)	0.0095 (8)
C26	0.0186 (9)	0.0200 (9)	0.0132 (8)	0.0090 (8)	0.0042 (7)	0.0032 (7)
C27	0.0142 (9)	0.0168 (9)	0.0188 (9)	0.0053 (7)	0.0109 (8)	0.0030 (7)
C28	0.0180 (9)	0.0189 (9)	0.0232 (9)	0.0090 (8)	0.0136 (8)	0.0053 (8)
C29	0.0159 (9)	0.0193 (9)	0.0189 (9)	0.0039 (8)	0.0117 (8)	0.0033 (7)
C30	0.0135 (8)	0.0179 (9)	0.0165 (9)	0.0034 (7)	0.0094 (7)	0.0042 (7)
C31	0.0189 (9)	0.0133 (9)	0.0169 (9)	0.0028 (8)	0.0066 (8)	0.0027 (7)
C32	0.0229 (10)	0.0193 (9)	0.0274 (10)	0.0059 (8)	0.0150 (9)	0.0096 (8)
C33	0.0226 (10)	0.0275 (10)	0.0225 (10)	0.0065 (8)	0.0128 (8)	0.0139 (8)
C34	0.0182 (9)	0.0281 (10)	0.0137 (9)	0.0021 (8)	0.0106 (8)	0.0066 (8)
C35	0.0211 (9)	0.0195 (9)	0.0115 (8)	0.0114 (8)	0.0058 (7)	0.0071 (7)
C36	0.0255 (10)	0.0258 (10)	0.0157 (9)	0.0161 (8)	0.0094 (8)	0.0092 (8)
C37	0.0376 (12)	0.0329 (11)	0.0202 (10)	0.0221 (10)	0.0151 (9)	0.0098 (8)
C38	0.0383 (12)	0.0488 (13)	0.0260 (11)	0.0283 (11)	0.0219 (10)	0.0205 (10)
C39	0.0267 (11)	0.0348 (11)	0.0312 (11)	0.0142 (9)	0.0163 (9)	0.0206 (9)
C40	0.0253 (10)	0.0255 (10)	0.0169 (9)	0.0119 (9)	0.0075 (8)	0.0102 (8)
C41	0.0166 (9)	0.0250 (10)	0.0218 (10)	0.0083 (8)	0.0099 (8)	0.0043 (8)
C42	0.0186 (9)	0.0338 (11)	0.0238 (10)	0.0163 (9)	0.0103 (8)	0.0115 (8)

Geometric parameters (Å, º)

O1—C21	1.418 (2)	O2—C42	1.428 (2)
O1—C1	1.4315 (19)	O2—C22	1.4296 (19)

N1—C8	1.371 (2)	N2—C28	1.371 (2)
N1—C7	1.378 (2)	N2—C29	1.372 (2)
N1—H1N	0.920 (19)	N2—H2N	0.906 (18)
C1—C5	1.542 (2)	C22—C26	1.549 (2)
C1—C2	1.550 (2)	C22—C23	1.553 (2)
C1—H1	1.0000	C22—H22	1.0000
C2—C6	1.513 (2)	C23—C27	1.510 (2)
C2—C20	1.542 (2)	C23—C41	1.541 (2)
C2—C3	1.548 (2)	C23—C24	1.546 (2)
C3—C4	1.534 (2)	C24—C25	1.523 (2)
C3—H3A	0.9900	C24—H24A	0.9900
C3—H3B	0.9900	C24—H24B	0.9900
C4—C5	1.546 (2)	C25—C26	1.543 (2)
C4—H4A	0.9900	C25—H25A	0.9900
C4—H4B	0.9900	C25—H25B	0.9900
C5—C14	1.509 (2)	C26—C35	1.514 (2)
C5—H5	1.0000	C26—H26	1.0000
C6—C7	1.366 (2)	C27—C28	1.363 (2)
C6—C9	1.446 (2)	C27—C30	1.444 (2)
C7—H7	0.9500	C28—H28	0.9500
C8—C13	1.393 (3)	C29—C34	1.395 (2)
C8—C9	1.418 (2)	C29—C30	1.419 (2)
C9—C10	1.398 (2)	C30—C31	1.410 (2)
C10—C11	1.381 (3)	C31—C32	1.377 (2)
C10—H10	0.9500	C31—H31	0.9500
C11—C12	1.404 (2)	C32—C33	1.401 (2)
C11—H11	0.9500	C32—H32	0.9500
C12—C13	1.375 (2)	C33—C34	1.377 (3)
C12—H12	0.9500	C33—H33	0.9500
C13—H13	0.9500	C34—H34	0.9500
C14—C19	1.395 (2)	C35—C36	1.390 (2)
C14—C15	1.401 (2)	C35—C40	1.398 (2)
C15—C16	1.387 (3)	C36—C37	1.389 (2)
C15—H15	0.9500	C36—H36	0.9500
C16—C17	1.380 (3)	C37—C38	1.388 (3)
C16—H16	0.9500	C37—H37	0.9500
C17—C18	1.394 (3)	C38—C39	1.379 (3)
C17—H17	0.9500	C38—H38	0.9500
C18—C19	1.396 (3)	C39—C40	1.392 (3)
C18—H18	0.9500	C39—H39	0.9500
C19—H19	0.9500	C40—H40	0.9500
C20—H20A	0.9800	C41—H41A	0.9800

C20—H20B	0.9800	C41—H41B	0.9800
C20—H20C	0.9800	C41—H41C	0.9800
C21—H21A	0.9800	C42—H42A	0.9800
C21—H21B	0.9800	C42—H42B	0.9800
C21—H21C	0.9800	C42—H42C	0.9800
C21—O1—C1	113.42 (12)	C42—O2—C22	113.38 (12)
C8—N1—C7	108.33 (14)	C28—N2—C29	108.32 (14)
C8—N1—H1N	129.2 (12)	C28—N2—H2N	123.1 (12)
C7—N1—H1N	119.8 (12)	C29—N2—H2N	126.4 (12)
O1—C1—C5	109.63 (13)	O2—C22—C26	110.69 (13)
O1—C1—C2	109.68 (12)	O2—C22—C23	109.69 (13)
C5—C1—C2	104.25 (13)	C26—C22—C23	103.93 (13)
O1—C1—H1	111.0	O2—C22—H22	110.8
C5—C1—H1	111.0	C26—C22—H22	110.8
C2—C1—H1	111.0	C23—C22—H22	110.8
C6—C2—C20	109.10 (14)	C27—C23—C41	109.20 (13)
C6—C2—C3	113.66 (13)	C27—C23—C24	113.69 (13)
C20—C2—C3	110.10 (14)	C41—C23—C24	109.61 (14)
C6—C2—C1	111.86 (14)	C27—C23—C22	112.20 (14)
C20—C2—C1	109.47 (13)	C41—C23—C22	109.40 (13)
C3—C2—C1	102.47 (13)	C24—C23—C22	102.53 (13)
C4—C3—C2	107.22 (13)	C25—C24—C23	107.27 (13)
C4—C3—H3A	110.3	C25—C24—H24A	110.3
C2—C3—H3A	110.3	C23—C24—H24A	110.3
C4—C3—H3B	110.3	C25—C24—H24B	110.3
C2—C3—H3B	110.3	C23—C24—H24B	110.3
H3A—C3—H3B	108.5	H24A—C24—H24B	108.5
C3—C4—C5	106.84 (14)	C24—C25—C26	107.35 (14)
C3—C4—H4A	110.4	C24—C25—H25A	110.2
C5—C4—H4A	110.4	C26—C25—H25A	110.2
C3—C4—H4B	110.4	C24—C25—H25B	110.2
C5—C4—H4B	110.4	C26—C25—H25B	110.2
H4A—C4—H4B	108.6	H25A—C25—H25B	108.5
C14—C5—C1	115.38 (14)	C35—C26—C25	116.55 (14)
C14—C5—C4	115.93 (14)	C35—C26—C22	114.62 (14)
C1—C5—C4	103.16 (13)	C25—C26—C22	103.42 (13)
C14—C5—H5	107.3	C35—C26—H26	107.2
C1—C5—H5	107.3	C25—C26—H26	107.2
C4—C5—H5	107.3	C22—C26—H26	107.2
C7—C6—C9	105.55 (15)	C28—C27—C30	105.43 (15)
C7—C6—C2	126.21 (16)	C28—C27—C23	126.29 (16)

C9—C6—C2	128.24 (14)	C30—C27—C23	128.27 (14)
C6—C7—N1	111.17 (16)	C27—C28—N2	111.48 (16)
C6—C7—H7	124.4	C27—C28—H28	124.3
N1—C7—H7	124.4	N2—C28—H28	124.3
N1—C8—C13	129.58 (16)	N2—C29—C34	129.69 (16)
N1—C8—C9	107.84 (15)	N2—C29—C30	107.65 (15)
C13—C8—C9	122.57 (16)	C34—C29—C30	122.66 (16)
C10—C9—C8	117.94 (16)	C31—C30—C29	117.80 (16)
C10—C9—C6	135.01 (15)	C31—C30—C27	135.12 (16)
C8—C9—C6	107.05 (14)	C29—C30—C27	107.06 (15)
C11—C10—C9	119.56 (16)	C32—C31—C30	119.39 (16)
C11—C10—H10	120.2	C32—C31—H31	120.3
C9—C10—H10	120.2	C30—C31—H31	120.3
C10—C11—C12	121.28 (17)	C31—C32—C33	121.42 (17)
C10—C11—H11	119.4	C31—C32—H32	119.3
C12—C11—H11	119.4	C33—C32—H32	119.3
C13—C12—C11	120.73 (17)	C34—C33—C32	121.08 (17)
C13—C12—H12	119.6	C34—C33—H33	119.5
C11—C12—H12	119.6	C32—C33—H33	119.5
C12—C13—C8	117.92 (16)	C33—C34—C29	117.64 (16)
C12—C13—H13	121.0	C33—C34—H34	121.2
C8—C13—H13	121.0	C29—C34—H34	121.2
C19—C14—C15	117.29 (16)	C36—C35—C40	117.84 (16)
C19—C14—C5	123.01 (15)	C36—C35—C26	119.09 (15)
C15—C14—C5	119.70 (15)	C40—C35—C26	123.06 (15)
C16—C15—C14	121.65 (17)	C37—C36—C35	121.57 (17)
C16—C15—H15	119.2	C37—C36—H36	119.2
C14—C15—H15	119.2	C35—C36—H36	119.2
C17—C16—C15	120.31 (16)	C38—C37—C36	119.73 (17)
C17—C16—H16	119.8	C38—C37—H37	120.1
C15—C16—H16	119.8	C36—C37—H37	120.1
C16—C17—C18	119.37 (17)	C39—C38—C37	119.67 (17)
C16—C17—H17	120.3	C39—C38—H38	120.2
C18—C17—H17	120.3	C37—C38—H38	120.2
C17—C18—C19	120.02 (17)	C38—C39—C40	120.41 (17)
C17—C18—H18	120.0	C38—C39—H39	119.8
C19—C18—H18	120.0	C40—C39—H39	119.8
C14—C19—C18	121.35 (16)	C39—C40—C35	120.78 (17)
C14—C19—H19	119.3	C39—C40—H40	119.6
C18—C19—H19	119.3	C35—C40—H40	119.6
C2—C20—H20A	109.5	C23—C41—H41A	109.5
C2—C20—H20B	109.5	C23—C41—H41B	109.5

H20A—C20—H20B	109.5	H41A—C41—H41B	109.5
C2—C20—H20C	109.5	C23—C41—H41C	109.5
H20A—C20—H20C	109.5	H41A—C41—H41C	109.5
H20B—C20—H20C	109.5	H41B—C41—H41C	109.5
O1—C21—H21A	109.5	O2—C42—H42A	109.5
O1—C21—H21B	109.5	O2—C42—H42B	109.5
H21A—C21—H21B	109.5	H42A—C42—H42B	109.5
O1—C21—H21C	109.5	O2—C42—H42C	109.5
H21A—C21—H21C	109.5	H42A—C42—H42C	109.5
H21B—C21—H21C	109.5	H42B—C42—H42C	109.5
C21—O1—C1—C5	108.16 (15)	C42—O2—C22—C26	-104.05 (15)
C21—O1—C1—C2	-137.95 (14)	C42—O2—C22—C23	141.84 (14)
O1—C1—C2—C6	43.55 (18)	O2—C22—C23—C27	-42.53 (18)
C5—C1—C2—C6	160.86 (13)	C26—C22—C23—C27	-160.91 (13)
O1—C1—C2—C20	164.60 (13)	O2—C22—C23—C41	-163.88 (13)
C5—C1—C2—C20	-78.09 (16)	C26—C22—C23—C41	77.73 (16)
O1—C1—C2—C3	-78.56 (15)	O2—C22—C23—C24	79.84 (15)
C5—C1—C2—C3	38.75 (16)	C26—C22—C23—C24	-38.55 (15)
C6—C2—C3—C4	-145.12 (15)	C27—C23—C24—C25	147.04 (15)
C20—C2—C3—C4	92.14 (17)	C41—C23—C24—C25	-90.45 (17)
C1—C2—C3—C4	-24.24 (17)	C22—C23—C24—C25	25.69 (18)
C2—C3—C4—C5	0.93 (19)	C23—C24—C25—C26	-3.2 (2)
O1—C1—C5—C14	-48.49 (18)	C24—C25—C26—C35	-147.53 (16)
C2—C1—C5—C14	-165.83 (14)	C24—C25—C26—C22	-20.79 (19)
O1—C1—C5—C4	78.96 (15)	O2—C22—C26—C35	47.07 (18)
C2—C1—C5—C4	-38.39 (16)	C23—C22—C26—	164.77 (13)

		C35	
C3—C4—C5—C14	150.06 (16)	O2—C22—C26—C25	-80.87 (16)
C3—C4—C5—C1	22.96 (18)	C23—C22—C26—C25	36.82 (16)
C20—C2—C6—C7	116.64 (18)	C41—C23—C27—C28	-112.43 (18)
C3—C2—C6—C7	-6.6 (2)	C24—C23—C27—C28	10.3 (2)
C1—C2—C6—C7	-122.10 (18)	C22—C23—C27—C28	126.10 (17)
C20—C2—C6—C9	-63.9 (2)	C41—C23—C27—C30	66.0 (2)
C3—C2—C6—C9	172.83 (16)	C24—C23—C27—C30	-171.31 (15)
C1—C2—C6—C9	57.4 (2)	C22—C23—C27—C30	-55.5 (2)
C9—C6—C7—N1	1.2 (2)	C30—C27—C28—N2	-0.97 (19)
C2—C6—C7—N1	-179.25 (15)	C23—C27—C28—N2	177.71 (15)
C8—N1—C7—C6	-2.4 (2)	C29—N2—C28—C27	2.13 (19)
C7—N1—C8—C13	-178.33 (18)	C28—N2—C29—C34	177.76 (17)
C7—N1—C8—C9	2.65 (18)	C28—N2—C29—C30	-2.37 (18)
N1—C8—C9—C10	178.48 (14)	N2—C29—C30—C31	-179.58 (14)
C13—C8—C9—C10	-0.6 (3)	C34—C29—C30—C31	0.3 (2)
N1—C8—C9—C6	-1.93 (18)	N2—C29—C30—C27	1.77 (18)
C13—C8—C9—C6	178.97 (15)	C34—C29—C30—C27	-178.34 (15)
C7—C6—C9—C10	179.95 (18)	C28—C27—C30—C31	-178.81 (18)
C2—C6—C9—C10	0.4 (3)	C23—C27—C30—C31	2.5 (3)
C7—C6—C9—C8	0.47 (19)	C28—C27—C30—C29	-0.51 (18)
C2—C6—C9—C8	-179.09 (15)	C23—C27—C30—C29	-179.15 (15)

C8—C9—C10—C11	1.1 (2)	C29—C30—C31—C32	-1.2 (2)
C6—C9—C10—C11	-178.31 (18)	C27—C30—C31—C32	176.94 (18)
C9—C10—C11—C12	-1.0 (3)	C30—C31—C32—C33	1.5 (3)
C10—C11—C12—C13	0.4 (3)	C31—C32—C33—C34	-0.8 (3)
C11—C12—C13—C8	0.2 (3)	C32—C33—C34—C29	-0.1 (2)
N1—C8—C13—C12	-178.93 (16)	N2—C29—C34—C33	-179.79 (16)
C9—C8—C13—C12	0.0 (3)	C30—C29—C34—C33	0.4 (2)
C1—C5—C14—C19	73.5 (2)	C25—C26—C35—C36	-133.82 (17)
C4—C5—C14—C19	-47.2 (2)	C22—C26—C35—C36	105.21 (18)
C1—C5—C14—C15	-105.43 (18)	C25—C26—C35—C40	47.5 (2)
C4—C5—C14—C15	133.84 (17)	C22—C26—C35—C40	-73.5 (2)
C19—C14—C15—C16	-0.8 (3)	C40—C35—C36—C37	-0.1 (3)
C5—C14—C15—C16	178.23 (16)	C26—C35—C36—C37	-178.88 (16)
C14—C15—C16—C17	0.8 (3)	C35—C36—C37—C38	0.4 (3)
C15—C16—C17—C18	-0.5 (3)	C36—C37—C38—C39	-0.4 (3)
C16—C17—C18—C19	0.2 (3)	C37—C38—C39—C40	0.1 (3)
C15—C14—C19—C18	0.5 (3)	C38—C39—C40—C35	0.1 (3)
C5—C14—C19—C18	-178.46 (16)	C36—C35—C40—C39	-0.1 (2)
C17—C18—C19—C14	-0.2 (3)	C26—C35—C40—C39	178.58 (17)

Hydrogen-bond geometry (Å, º)

D—H···*A*	*D*—H	H···*A*	*D*···*A*	*D*—H···*A*
N1—H1*N*···O1^{i}	0.920 (19)	1.935 (19)	2.8423 (18)	168.6 (17)

N2—H2*N*···O2ii	0.906 (18)	1.996 (18)	2.8616 (18)	159.2 (16)

Symmetry codes: (i) -*x*+1, -*y*, -*z*+1; (ii) -*x*+2, -*y*+2, -*z*+1.

(±)-*1-(3-(1-methoxy-2-methyl-5-phenyl-6-oxabicyclo[3.1.0]hexan-2-yl)-1H-indol-1-yl)ethanone* (32)

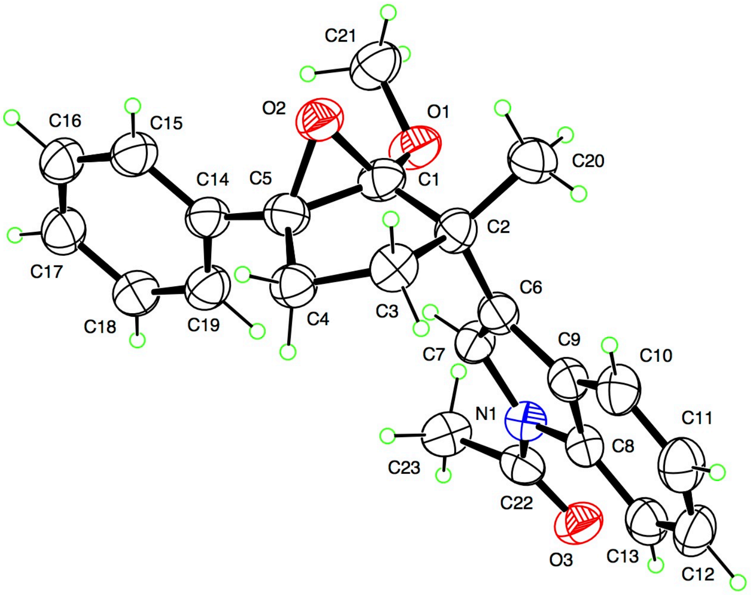

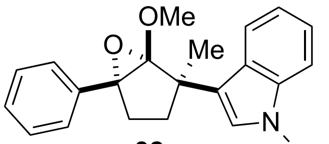

32

Crystal data

$C_{23}H_{23}NO_3$	*F*(000) = 768
M_r = 361.42	D_x = 1.303 Mg m^{-3}
Orthorhombic, $P2_12_12_1$	Cu *K*α radiation, λ = 1.54184 Å
Hall symbol: P 2ac 2ab	Cell parameters from 7072 reflections
a = 7.2261 (3) Å	θ = 4.0–60.9°
b = 13.4245 (5) Å	μ = 0.69 mm^{-1}
c = 18.9990 (9) Å	*T* = 90 K
V = 1843.03 (13) Å^3	Needle, colorless
Z = 4	0.23 × 0.04 × 0.02 mm

Data collection

Bruker Kappa APEX-II DUO diffractometer	2831 independent reflections
Radiation source: IμS microfocus	2333 reflections with *I* > 2σ(*I*)
QUAZAR multilayer optics	R_{int} = 0.073

ϕ and ω scans	θ_{max} = 61.2°, θ_{min} = 4.0°
Absorption correction: multi-scan *SADABS* (Sheldrick, 2004)	h = -7 – 8
T_{min} = 0.837, T_{max} = 0.986	k = -13 – 15
18058 measured reflections	l = -21 – 21

Refinement

Refinement on F^2	Secondary atom site location: difference Fourier map
Least-squares matrix: full	Hydrogen site location: inferred from neighbouring sites
$R[F^2 > 2\sigma(F^2)]$ = 0.052	H-atom parameters constrained
$wR(F^2)$ = 0.122	$w = 1/[\sigma^2(F_o^2) + (0.0357P)^2 + 0.8243P]$ where $P = (F_o^2 + 2F_c^2)/3$
S = 1.07	$(\Delta/\sigma)_{max}$ < 0.001
2831 reflections	$\Delta\rangle_{max}$ = 0.17 e Å^{-3}
247 parameters	$\Delta\rangle_{min}$ = -0.14 e Å^{-3}
0 restraints	Absolute structure: 1174 Friedel pairs (Flack, 1983)
Primary atom site location: structure-invariant direct methods	Flack parameter: 0.1 (4)

Fractional atomic coordinates and isotropic or equivalent isotropic displacement parameters (Å^2)

	x	y	z	U_{iso}*/U_{eq}	
O1	0.4610 (3)	0.46844 (17)	0.62253 (14)	0.0625 (7)	
O2	0.6491 (3)	0.44203 (16)	0.72455 (12)	0.0560 (6)	
O3	0.6346 (4)	0.75636 (17)	0.37777 (12)	0.0588 (7)	
N1	0.6480 (4)	0.70144 (18)	0.49084 (14)	0.0462 (7)	
C1	0.6063 (5)	0.4995 (2)	0.66414 (18)	0.0523 (9)	
C2	0.6150 (5)	0.6120 (2)	0.67643 (17)	0.0505 (9)	
C3	0.7995 (5)	0.6221 (3)	0.7166 (2)	0.0557 (10)	
H3A	0.8570	0.6876	0.7069	0.067*	
H3B	0.7789	0.6160	0.7679	0.067*	

C4	0.9250 (5)	0.5372 (2)	0.69003 (19)	0.0541 (9)	
H4A	0.9993	0.5590	0.6490	0.065*	
H4B	1.0097	0.5143	0.7276	0.065*	
C5	0.7907 (5)	0.4557 (3)	0.6694 (2)	0.0536 (9)	
C6	0.6251 (5)	0.6674 (2)	0.60702 (18)	0.0466 (8)	
C7	0.6317 (5)	0.6274 (2)	0.54184 (18)	0.0496 (8)	
H7	0.6259	0.5581	0.5321	0.060*	
C8	0.6539 (5)	0.7935 (2)	0.52544 (17)	0.0470 (9)	
C9	0.6416 (5)	0.7735 (2)	0.59808 (18)	0.0494 (9)	
C10	0.6484 (5)	0.8539 (2)	0.6448 (2)	0.0589 (10)	
H10	0.6393	0.8431	0.6941	0.071*	
C11	0.6684 (5)	0.9485 (3)	0.6187 (2)	0.0641 (11)	
H11	0.6720	1.0032	0.6504	0.077*	
C12	0.6835 (5)	0.9664 (3)	0.5466 (2)	0.0626 (11)	
H12	0.6991	1.0326	0.5301	0.075*	
C13	0.6762 (5)	0.8896 (2)	0.4994 (2)	0.0552 (9)	
H13	0.6860	0.9016	0.4502	0.066*	
C14	0.8539 (5)	0.3652 (3)	0.6314 (2)	0.0533 (9)	
C15	0.8754 (5)	0.2749 (3)	0.6653 (2)	0.0607 (10)	
H15	0.8473	0.2692	0.7139	0.073*	
C16	0.9383 (5)	0.1924 (3)	0.6281 (2)	0.0626 (10)	
H16	0.9520	0.1302	0.6514	0.075*	
C17	0.9811 (5)	0.2007 (3)	0.5572 (2)	0.0624 (11)	
H17	1.0247	0.1443	0.5319	0.075*	
C18	0.9600 (5)	0.2912 (2)	0.5235 (2)	0.0611 (10)	
H18	0.9886	0.2971	0.4748	0.073*	
C19	0.8976 (5)	0.3729 (3)	0.5603 (2)	0.0571 (10)	
H19	0.8842	0.4350	0.5369	0.069*	
C20	0.4505 (5)	0.6464 (3)	0.7209 (2)	0.0637 (11)	
H20A	0.3352	0.6333	0.6954	0.096*	
H20B	0.4613	0.7180	0.7301	0.096*	
H20C	0.4496	0.6101	0.7656	0.096*	
C21	0.3832 (6)	0.3741 (3)	0.6361 (2)	0.0671 (11)	
H21A	0.4799	0.3231	0.6332	0.101*	
H21B	0.2868	0.3600	0.6012	0.101*	
H21C	0.3287	0.3737	0.6834	0.101*	
C22	0.6482 (5)	0.6864 (3)	0.41727	0.0507 (9)	

			(19)		
C23	0.6673 (5)	0.5806 (2)	0.39377 (18)	0.0594 (10)	
H23A	0.5537	0.5440	0.4049	0.089*	
H23B	0.7722	0.5496	0.4181	0.089*	
H23C	0.6887	0.5787	0.3428	0.089*	

Atomic displacement parameters (Å^2)

	U^{11}	U^{22}	U^{33}	U^{12}	U^{13}	U^{23}
O1	0.0604 (16)	0.0636 (15)	0.0636 (17)	-0.0150 (13)	-0.0133 (14)	0.0187 (14)
O2	0.0569 (15)	0.0570 (14)	0.0542 (15)	-0.0033 (13)	-0.0009 (14)	0.0118 (12)
O3	0.0645 (16)	0.0574 (15)	0.0544 (16)	-0.0052 (13)	-0.0005 (15)	0.0136 (13)
N1	0.0448 (17)	0.0460 (15)	0.0478 (17)	-0.0007 (14)	0.0032 (15)	0.0019 (13)
C1	0.055 (2)	0.057 (2)	0.045 (2)	-0.0056 (18)	-0.0048 (19)	0.0073 (17)
C2	0.052 (2)	0.052 (2)	0.048 (2)	0.0043 (18)	-0.0040 (18)	0.0056 (16)
C3	0.062 (2)	0.053 (2)	0.052 (2)	-0.0026 (18)	-0.0036 (19)	-0.0035 (18)
C4	0.054 (2)	0.057 (2)	0.052 (2)	-0.0030 (17)	-0.0012 (18)	0.0048 (18)
C5	0.049 (2)	0.058 (2)	0.054 (2)	-0.0036 (17)	0.0007 (19)	0.0048 (19)
C6	0.0423 (19)	0.0464 (18)	0.051 (2)	0.0012 (16)	0.0015 (18)	-0.0024 (16)
C7	0.050 (2)	0.0420 (17)	0.057 (2)	0.0020 (16)	-0.0009 (19)	0.0045 (17)
C8	0.041 (2)	0.0458 (19)	0.055 (2)	0.0052 (16)	0.0014 (18)	0.0016 (16)
C9	0.043 (2)	0.050 (2)	0.055 (2)	0.0038 (17)	-0.0008 (18)	0.0004 (17)
C10	0.062 (3)	0.054 (2)	0.060 (2)	0.008 (2)	0.000 (2)	-0.0037 (18)
C11	0.067 (3)	0.054 (2)	0.070 (3)	0.0049 (19)	-0.004 (2)	-0.012 (2)
C12	0.063 (3)	0.0427 (19)	0.082 (3)	0.0044 (17)	-0.007 (2)	0.002 (2)
C13	0.044 (2)	0.057 (2)	0.064 (2)	0.0035	-0.0049	-0.001 (2)

				(16)	(19)	
C14	0.046 (2)	0.054 (2)	0.060 (2)	-0.0047 (17)	-0.002 (2)	0.0109 (19)
C15	0.056 (2)	0.056 (2)	0.070 (3)	-0.006 (2)	-0.003 (2)	0.010 (2)
C16	0.057 (2)	0.046 (2)	0.085 (3)	-0.0026 (17)	-0.003 (2)	0.007 (2)
C17	0.057 (2)	0.047 (2)	0.083 (3)	-0.0018 (18)	-0.002 (2)	-0.003 (2)
C18	0.061 (2)	0.054 (2)	0.068 (3)	-0.0031 (19)	0.002 (2)	0.001 (2)
C19	0.061 (2)	0.047 (2)	0.063 (3)	-0.0034 (18)	-0.001 (2)	0.0031 (19)
C20	0.059 (3)	0.074 (3)	0.058 (3)	0.004 (2)	0.009 (2)	0.009 (2)
C21	0.072 (3)	0.054 (2)	0.076 (3)	-0.010 (2)	-0.010 (2)	0.007 (2)
C22	0.040 (2)	0.062 (2)	0.050 (2)	-0.0041 (18)	0.0023 (18)	0.0013 (19)
C23	0.071 (3)	0.056 (2)	0.052 (2)	-0.0077 (19)	0.002 (2)	-0.0019 (18)

Geometric parameters (Å, º)

O1—C1	1.379 (4)	C10—H10	0.9500
O1—C21	1.410 (4)	C11—C12	1.394 (5)
O2—C1	1.417 (4)	C11—H11	0.9500
O2—C5	1.476 (4)	C12—C13	1.367 (5)
O3—C22	1.206 (4)	C12—H12	0.9500
N1—C7	1.393 (4)	C13—H13	0.9500
N1—C8	1.400 (4)	C14—C15	1.381 (5)
N1—C22	1.412 (4)	C14—C19	1.391 (5)
C1—C5	1.460 (5)	C15—C16	1.389 (5)
C1—C2	1.530 (5)	C15—H15	0.9500
C2—C6	1.516 (4)	C16—C17	1.386 (5)
C2—C20	1.530 (5)	C16—H16	0.9500
C2—C3	1.542 (5)	C17—C18	1.383 (5)
C3—C4	1.541 (5)	C17—H17	0.9500
C3—H3A	0.9900	C18—C19	1.376 (5)
C3—H3B	0.9900	C18—H18	0.9500
C4—C5	1.514 (5)	C19—H19	0.9500
C4—H4A	0.9900	C20—H20A	0.9800
C4—H4B	0.9900	C20—H20B	0.9800
C5—C14	1.485 (5)	C20—H20C	0.9800
C6—C7	1.350 (4)	C21—H21A	0.9800

C6—C9	1.439 (4)	C21—H21B	0.9800
C7—H7	0.9500	C21—H21C	0.9800
C8—C13	1.392 (4)	C22—C23	1.496 (5)
C8—C9	1.409 (4)	C23—H23A	0.9800
C9—C10	1.399 (5)	C23—H23B	0.9800
C10—C11	1.372 (5)	C23—H23C	0.9800
C1—O1—C21	118.1 (3)	C9—C10—H10	120.4
C1—O2—C5	60.6 (2)	C10—C11—C12	121.6 (4)
C7—N1—C8	107.8 (3)	C10—C11—H11	119.2
C7—N1—C22	125.9 (3)	C12—C11—H11	119.2
C8—N1—C22	126.2 (3)	C13—C12—C11	120.8 (4)
O1—C1—O2	117.8 (3)	C13—C12—H12	119.6
O1—C1—C5	127.8 (3)	C11—C12—H12	119.6
O2—C1—C5	61.7 (2)	C12—C13—C8	118.0 (4)
O1—C1—C2	114.7 (3)	C12—C13—H13	121.0
O2—C1—C2	113.9 (3)	C8—C13—H13	121.0
C5—C1—C2	110.5 (3)	C15—C14—C19	119.5 (4)
C6—C2—C20	111.7 (3)	C15—C14—C5	121.7 (3)
C6—C2—C1	110.7 (3)	C19—C14—C5	118.8 (3)
C20—C2—C1	110.5 (3)	C14—C15—C16	120.0 (4)
C6—C2—C3	110.2 (3)	C14—C15—H15	120.0
C20—C2—C3	111.9 (3)	C16—C15—H15	120.0
C1—C2—C3	101.4 (3)	C17—C16—C15	120.2 (3)
C4—C3—C2	106.4 (3)	C17—C16—H16	119.9
C4—C3—H3A	110.5	C15—C16—H16	119.9
C2—C3—H3A	110.5	C18—C17—C16	119.8 (4)
C4—C3—H3B	110.5	C18—C17—H17	120.1
C2—C3—H3B	110.5	C16—C17—H17	120.1
H3A—C3—H3B	108.6	C19—C18—C17	120.0 (4)
C5—C4—C3	104.0 (3)	C19—C18—H18	120.0
C5—C4—H4A	111.0	C17—C18—H18	120.0
C3—C4—H4A	111.0	C18—C19—C14	120.6 (3)
C5—C4—H4B	111.0	C18—C19—H19	119.7
C3—C4—H4B	111.0	C14—C19—H19	119.7
H4A—C4—H4B	109.0	C2—C20—H20A	109.5
C1—C5—O2	57.7 (2)	C2—C20—H20B	109.5
C1—C5—C14	125.2 (3)	H20A—C20—H20B	109.5
O2—C5—C14	117.2 (3)	C2—C20—H20C	109.5
C1—C5—C4	108.1 (3)	H20A—C20—H20C	109.5
O2—C5—C4	110.5 (3)	H20B—C20—H20C	109.5
C14—C5—C4	121.4 (3)	O1—C21—H21A	109.5

C7—C6—C9	106.4 (3)	O1—C21—H21B	109.5
C7—C6—C2	127.2 (3)	H21A—C21—H21B	109.5
C9—C6—C2	126.3 (3)	O1—C21—H21C	109.5
C6—C7—N1	110.9 (3)	H21A—C21—H21C	109.5
C6—C7—H7	124.5	H21B—C21—H21C	109.5
N1—C7—H7	124.5	O3—C22—N1	120.3 (3)
C13—C8—N1	130.9 (3)	O3—C22—C23	124.1 (3)
C13—C8—C9	122.2 (3)	N1—C22—C23	115.6 (3)
N1—C8—C9	106.8 (3)	C22—C23—H23A	109.5
C10—C9—C8	118.2 (3)	C22—C23—H23B	109.5
C10—C9—C6	133.8 (3)	H23A—C23—H23B	109.5
C8—C9—C6	108.0 (3)	C22—C23—H23C	109.5
C11—C10—C9	119.2 (4)	H23A—C23—H23C	109.5
C11—C10—H10	120.4	H23B—C23—H23C	109.5
C21—O1—C1—O2	-9.7 (5)	C8—N1—C7—C6	0.5 (4)
C21—O1—C1—C5	64.6 (5)	C22—N1—C7—C6	-176.2 (3)
C21—O1—C1—C2	-147.9 (3)	C7—N1—C8—C13	177.3 (4)
C5—O2—C1—O1	120.2 (4)	C22—N1—C8—C13	-6.0 (6)
C5—O2—C1—C2	-101.3 (4)	C7—N1—C8—C9	0.3 (4)
O1—C1—C2—C6	-57.1 (4)	C22—N1—C8—C9	177.0 (3)
O2—C1—C2—C6	163.1 (3)	C13—C8—C9—C10	1.3 (5)
C5—C1—C2—C6	95.9 (4)	N1—C8—C9—C10	178.7 (3)
O1—C1—C2—C20	67.2 (4)	C13—C8—C9—C6	-178.2 (3)
O2—C1—C2—C20	-72.6 (4)	N1—C8—C9—C6	-0.9 (4)
C5—C1—C2—C20	-139.8 (3)	C7—C6—C9—C10	-178.2 (4)
O1—C1—C2—C3	-174.1 (3)	C2—C6—C9—C10	-1.8 (7)
O2—C1—C2—C3	46.1 (4)	C7—C6—C9—C8	1.2 (4)
C5—C1—C2—C3	-21.0 (4)	C2—C6—C9—C8	177.7 (3)
C6—C2—C3—C4	-87.0 (3)	C8—C9—C10—C11	-0.6 (6)
C20—C2—C3—C4	148.1 (3)	C6—C9—C10—C11	178.8 (4)
C1—C2—C3—C4	30.3 (3)	C9—C10—C11—C12	-0.5 (6)
C2—C3—C4—C5	-29.0 (4)	C10—C11—C12—C13	0.9 (6)
O1—C1—C5—O2	-104.6 (4)	C11—C12—C13—C8	-0.2 (6)
C2—C1—C5—O2	106.8 (3)	N1—C8—C13—C12	-177.5 (4)
O1—C1—C5—C14	-2.1 (6)	C9—C8—C13—C12	-0.9 (5)
O2—C1—C5—C14	102.5 (4)	C1—C5—C14—C15	-105.5 (5)

C2—C1—C5—C14	-150.7 (3)	O2—C5—C14—C15	-37.4 (5)
O1—C1—C5—C4	152.1 (3)	C4—C5—C14—C15	103.4 (4)
O2—C1—C5—C4	-103.3 (3)	C1—C5—C14—C19	76.5 (5)
C2—C1—C5—C4	3.6 (4)	O2—C5—C14—C19	144.6 (3)
C1—O2—C5—C14	-116.2 (4)	C4—C5—C14—C19	-74.6 (5)
C1—O2—C5—C4	99.0 (3)	C19—C14—C15—C16	-0.7 (6)
C3—C4—C5—C1	15.7 (4)	C5—C14—C15—C16	-178.7 (3)
C3—C4—C5—O2	-45.8 (4)	C14—C15—C16—C17	0.6 (6)
C3—C4—C5—C14	171.1 (3)	C15—C16—C17—C18	-0.3 (6)
C20—C2—C6—C7	-125.5 (4)	C16—C17—C18—C19	0.3 (6)
C1—C2—C6—C7	-1.9 (5)	C17—C18—C19—C14	-0.5 (6)
C3—C2—C6—C7	109.5 (4)	C15—C14—C19—C18	0.7 (6)
C20—C2—C6—C9	58.7 (5)	C5—C14—C19—C18	178.7 (4)
C1—C2—C6—C9	-177.6 (4)	C7—N1—C22—O3	168.6 (3)
C3—C2—C6—C9	-66.2 (4)	C8—N1—C22—O3	-7.6 (6)
C9—C6—C7—N1	-1.1 (4)	C7—N1—C22—C23	-11.8 (5)
C2—C6—C7—N1	-177.5 (3)	C8—N1—C22—C23	172.0 (3)

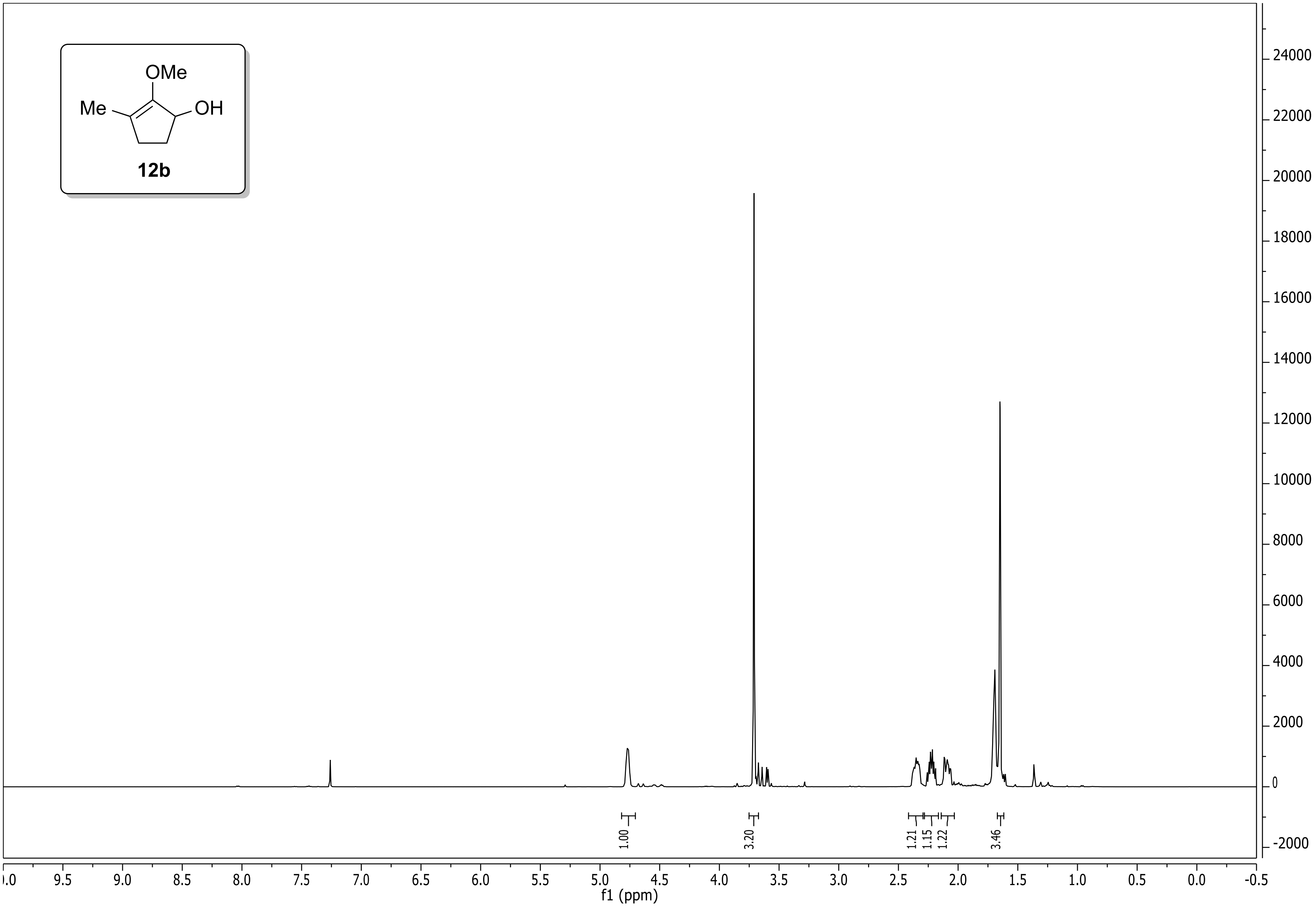
OMe
Me
OH
12b
1.00
3.20
1.21
1.15
1.22
3.46
f1 (ppm)

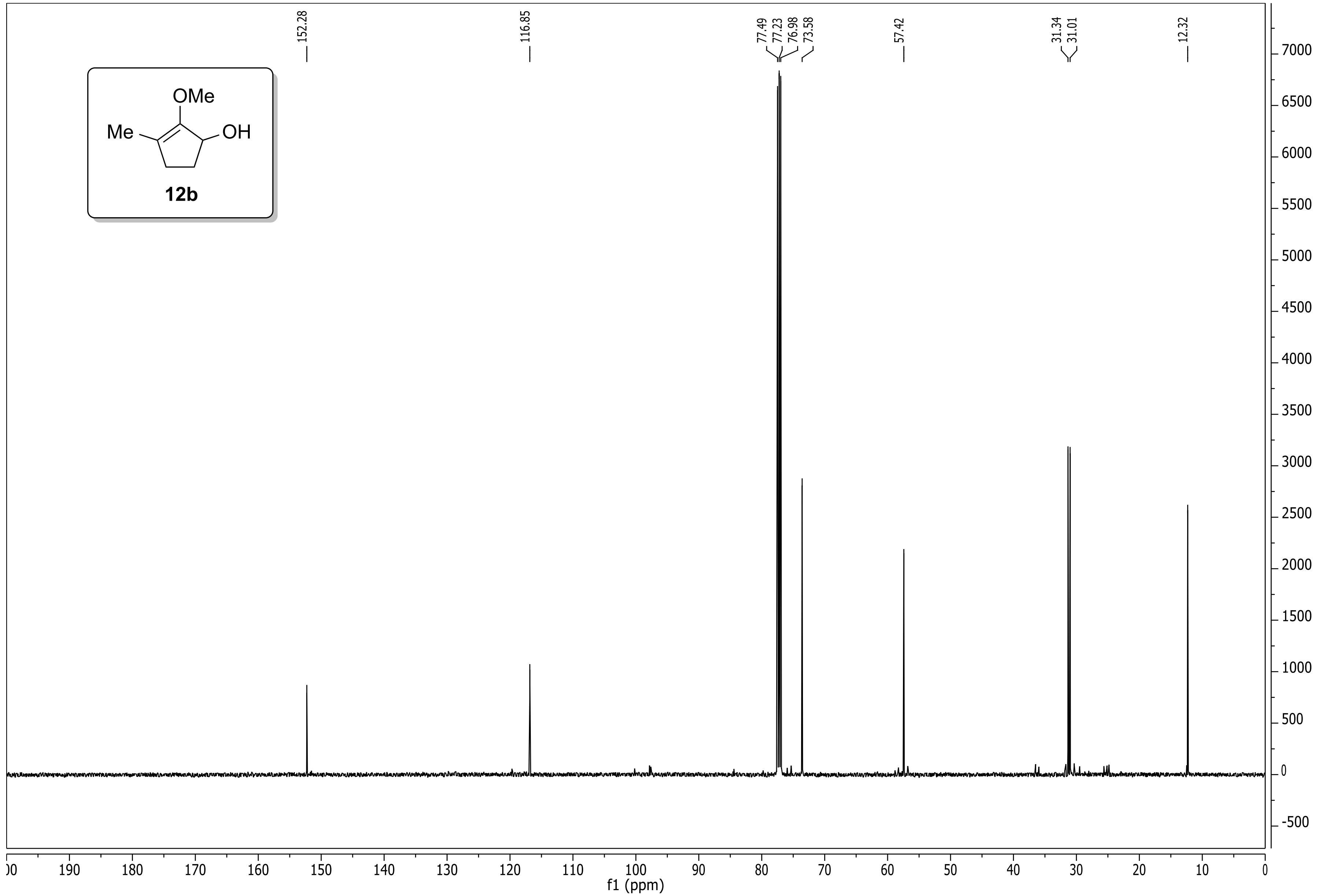
OMe
Me
OH
12b
152.28
116.85
77.49
77.23
76.98
73.58
57.42
31.34
31.01
12.32
f1 (ppm)

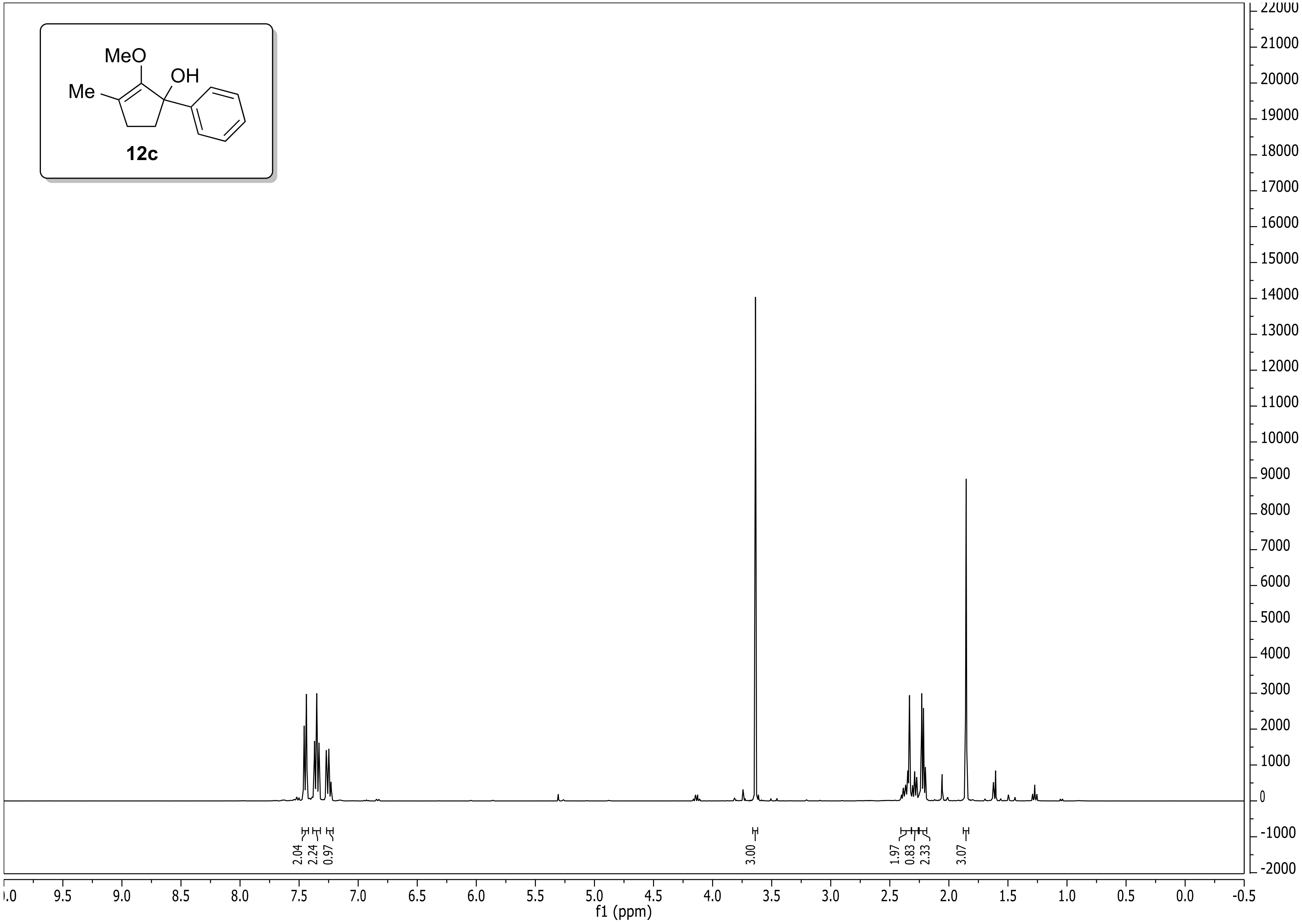
MeO
OH
Me
12c
f1 (ppm)

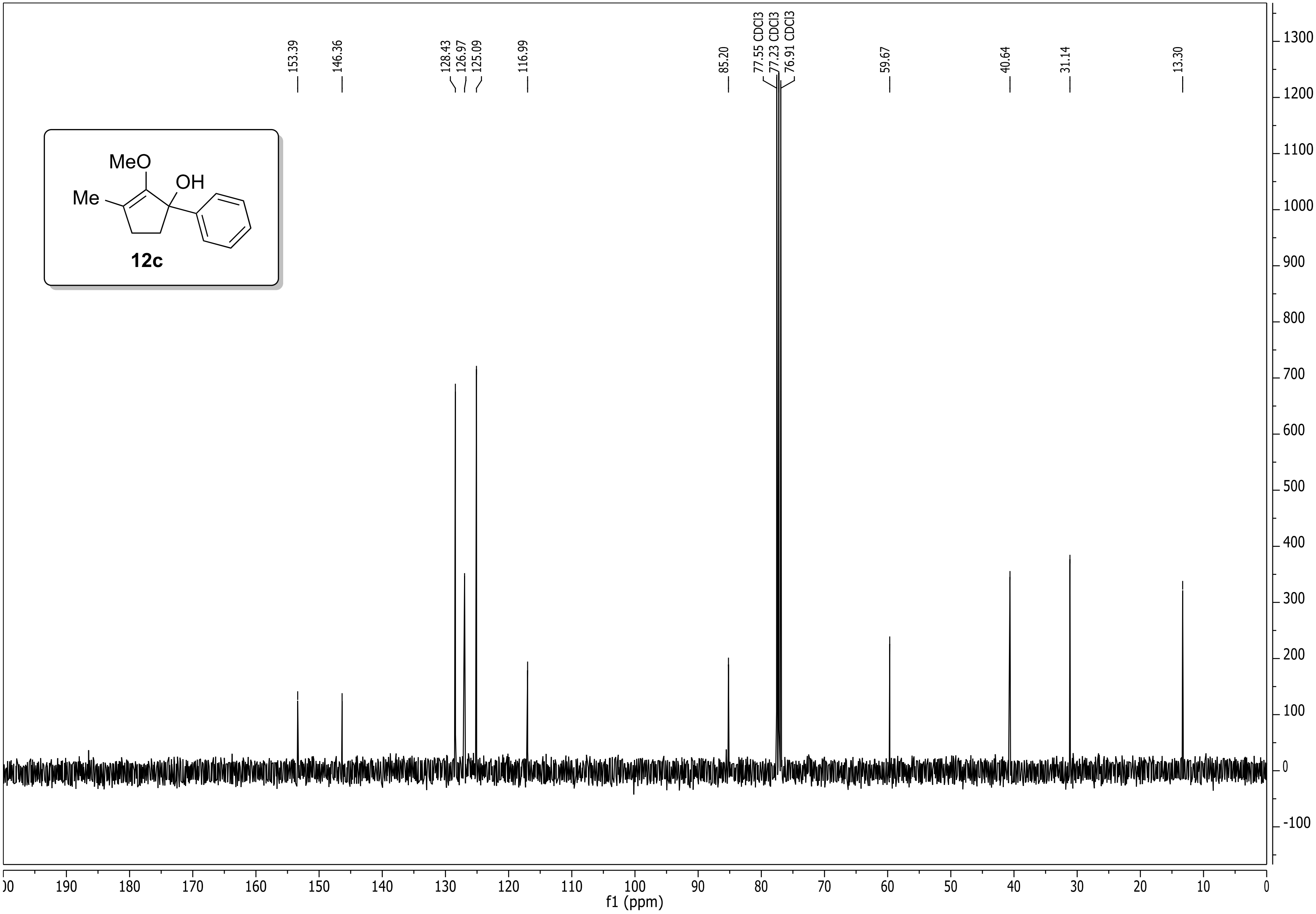
153.39
146.36
128.43
126.97
125.09
116.99
85.20
77.55 CDCl3
77.23 CDCl3
76.91 CDCl3
59.67
40.64
31.14
13.30
MeO
OH
Me
12c
f1 (ppm)

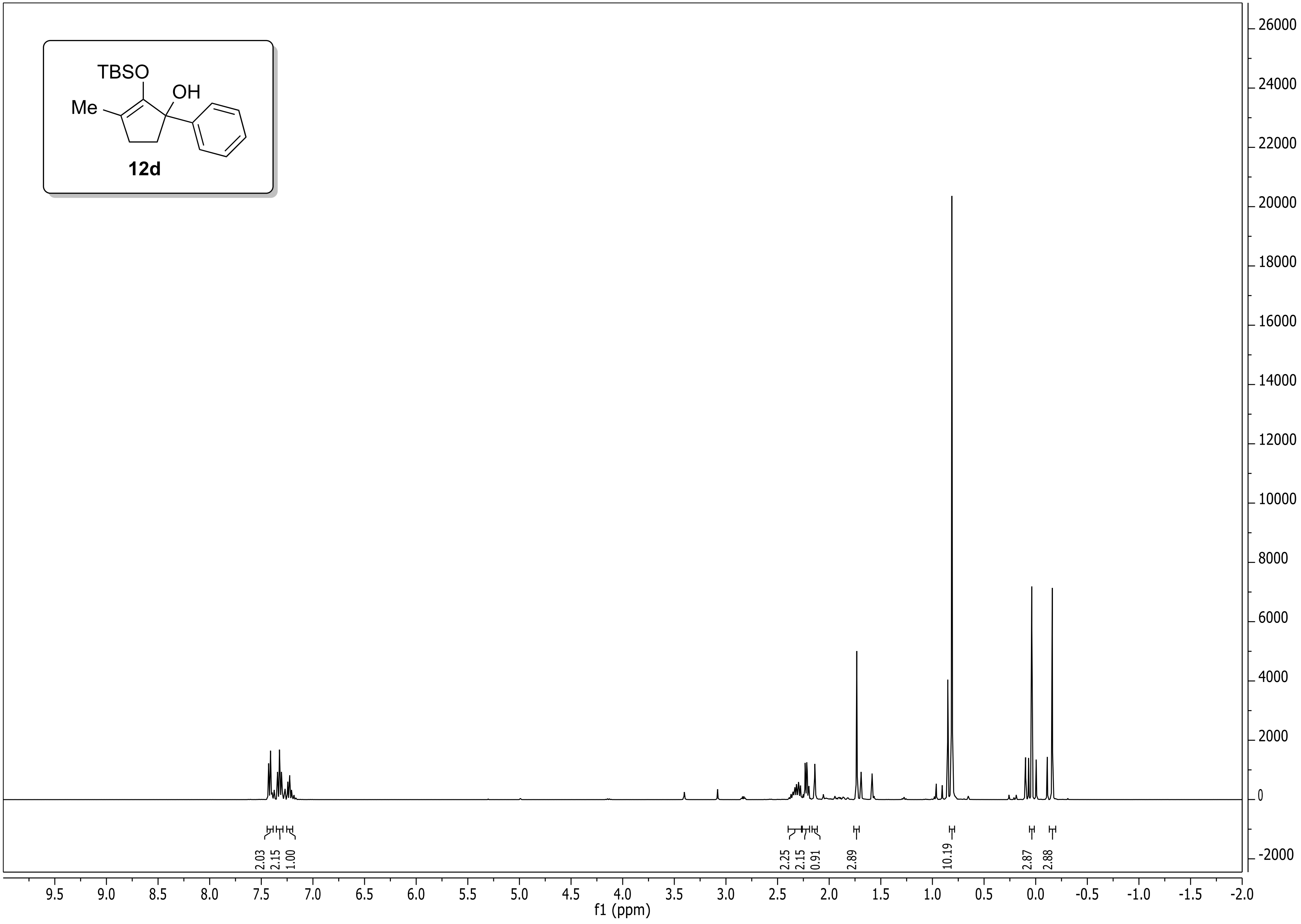

TBSO
OH
Me
12d
f1 (ppm)

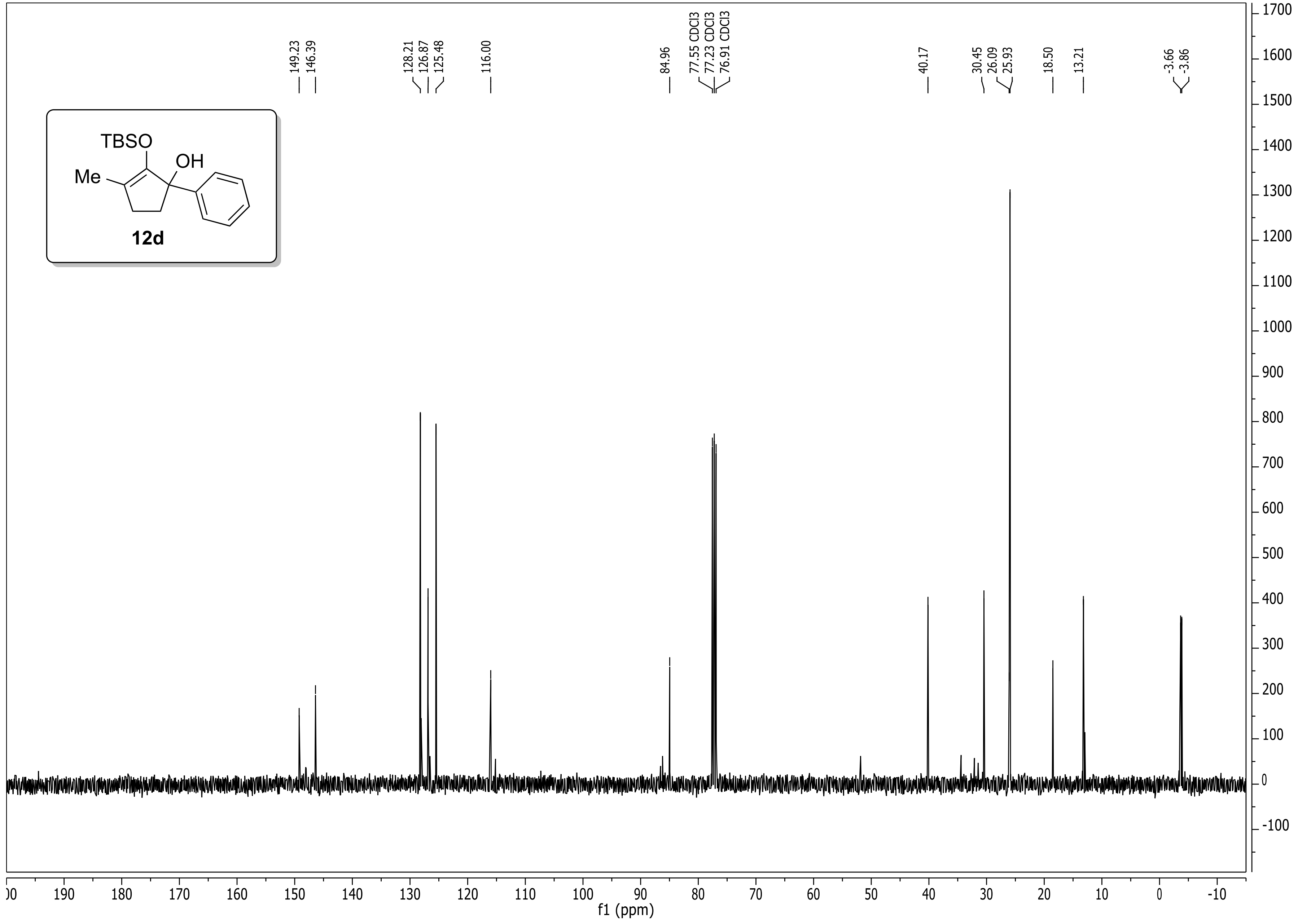
TBSO
OH
Me
12d
149.23
146.39
128.21
126.87
125.48
116.00
84.96
77.55 CDCl3
77.23 CDCl3
76.91 CDCl3
40.17
30.45
26.09
25.93
18.50
13.21
-3.66
-3.86
f1 (ppm)

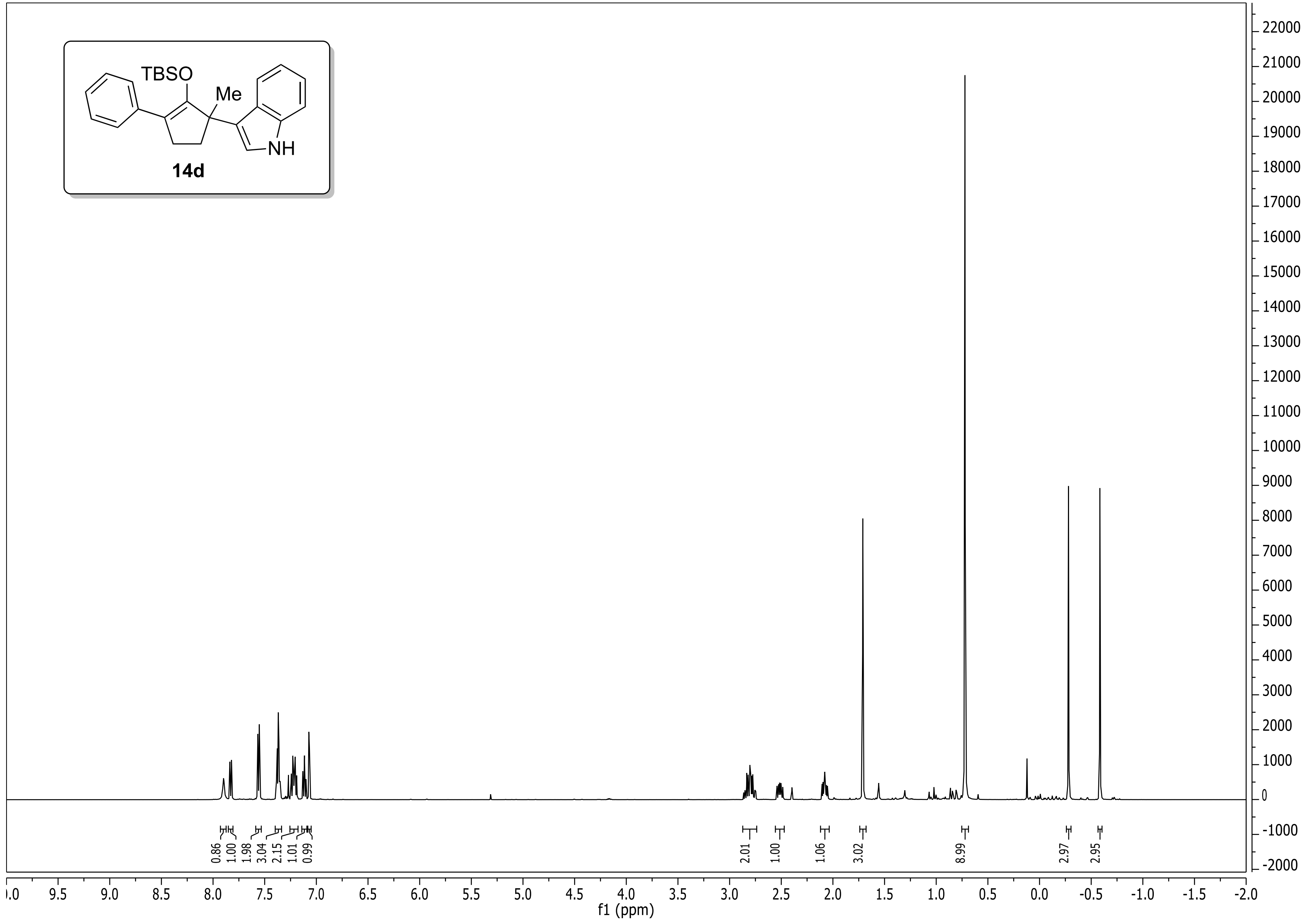

TBSO
Me
NH
14d
0.86
1.00
1.98
3.04
2.15
1.01
0.99
2.01
1.00
1.06
3.02
8.99
2.97
2.95
f1 (ppm)

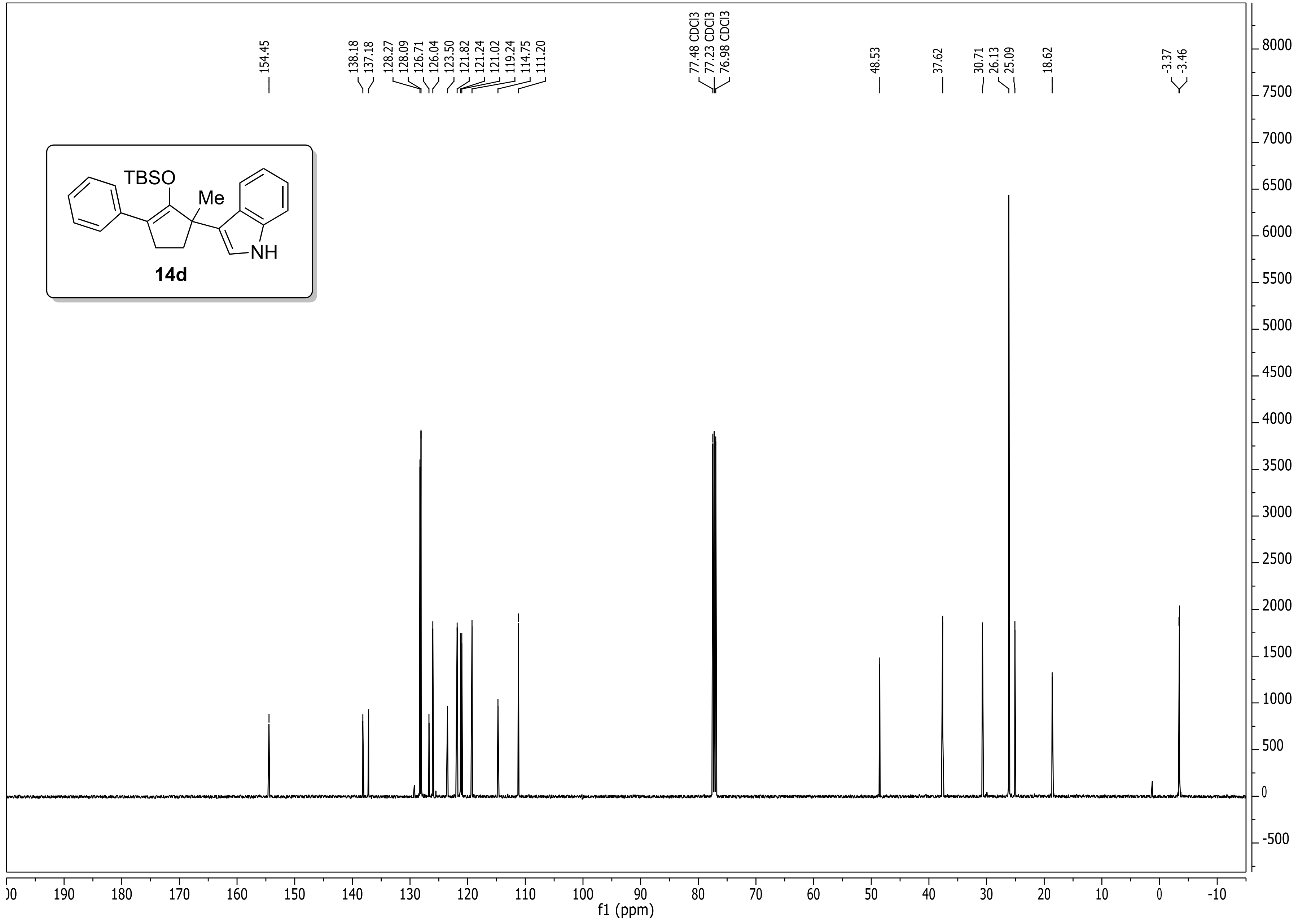
154.45
138.18
137.18
128.27
128.09
126.71
126.04
123.50
121.82
121.24
121.02
119.24
114.75
111.20
77.48 CDCl3
77.23 CDCl3
76.98 CDCl3
48.53
37.62
30.71
26.13
25.09
18.62
-3.37
-3.46
TBSO
Me
NH
14d
f1 (ppm)

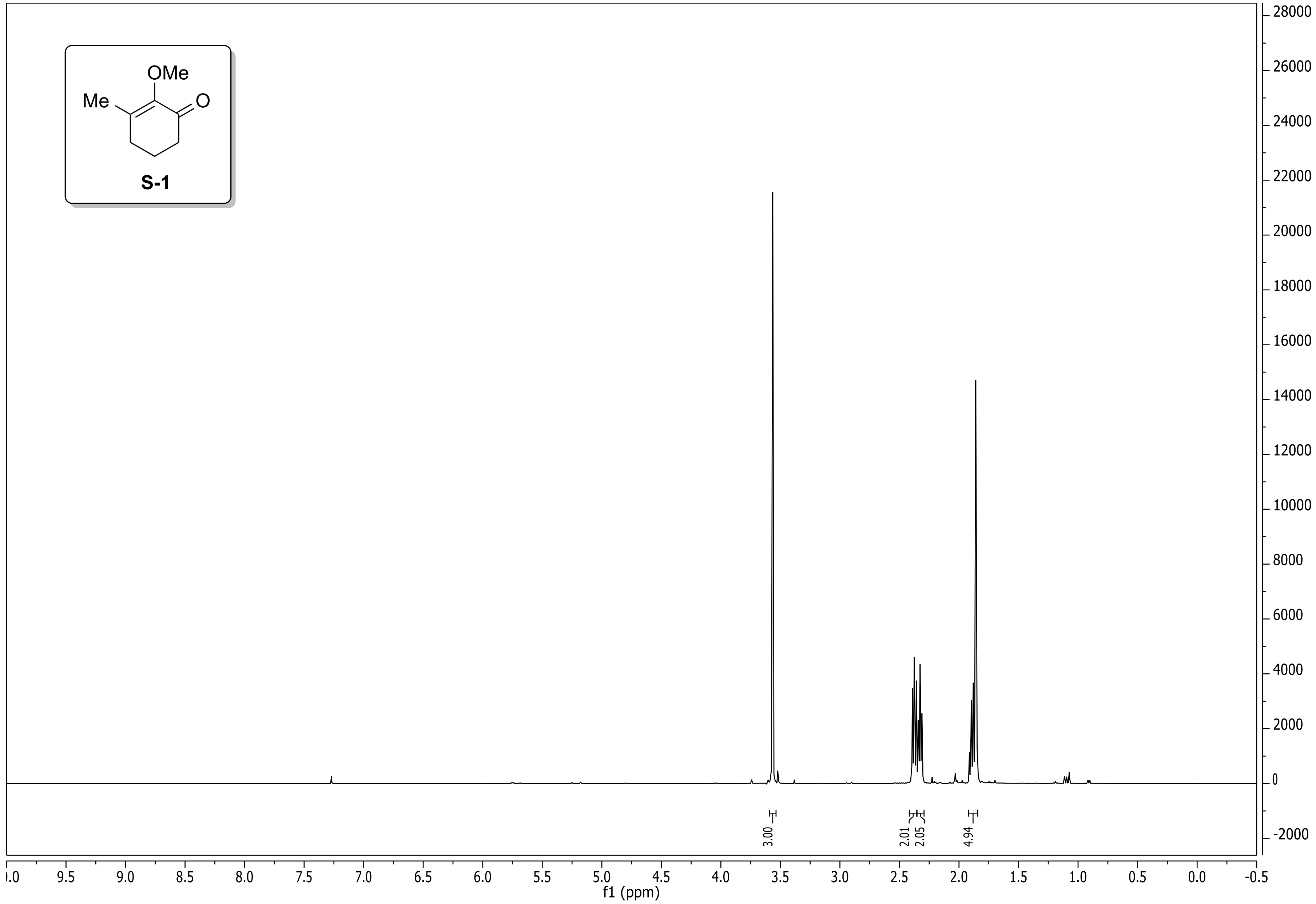
OMe
Me
O
S-1
3.00
2.01
2.05
4.94
f1 (ppm)

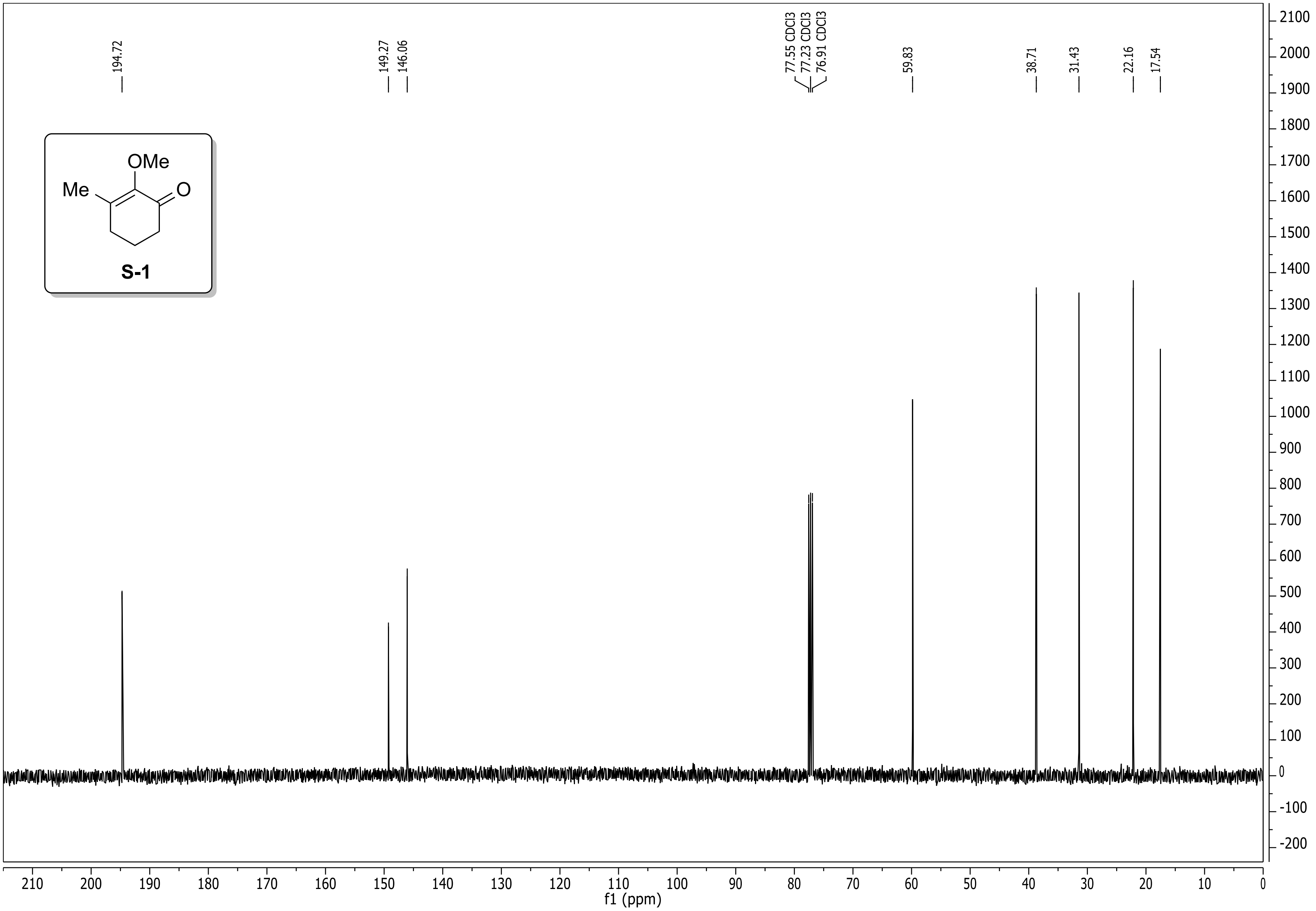
194.72
149.27
146.06
77.55 CDCl3
77.23 CDCl3
76.91 CDCl3
59.83
38.71
31.43
22.16
17.54
OMe
Me
O
S-1
210 200 190 180 170 160 150 140 130 120 110 100 90 80 70 60 50 40 30 20 10 0
f1 (ppm)
2100 2000 1900 1800 1700 1600 1500 1400 1300 1200 1100 1000 900 800 700 600 500 400 300 200 100 0 -100 -200

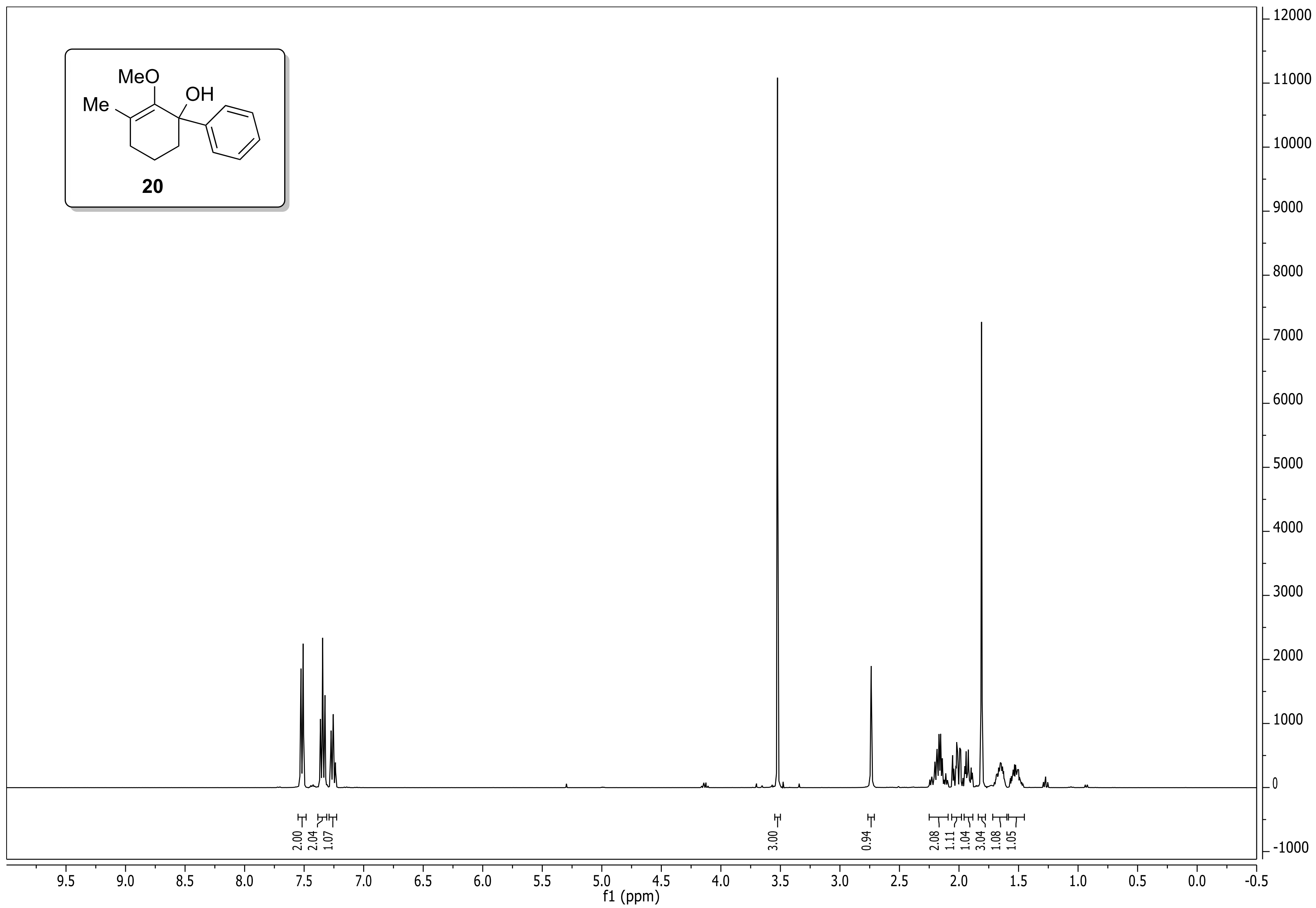
MeO
OH
Me
20
f1 (ppm)

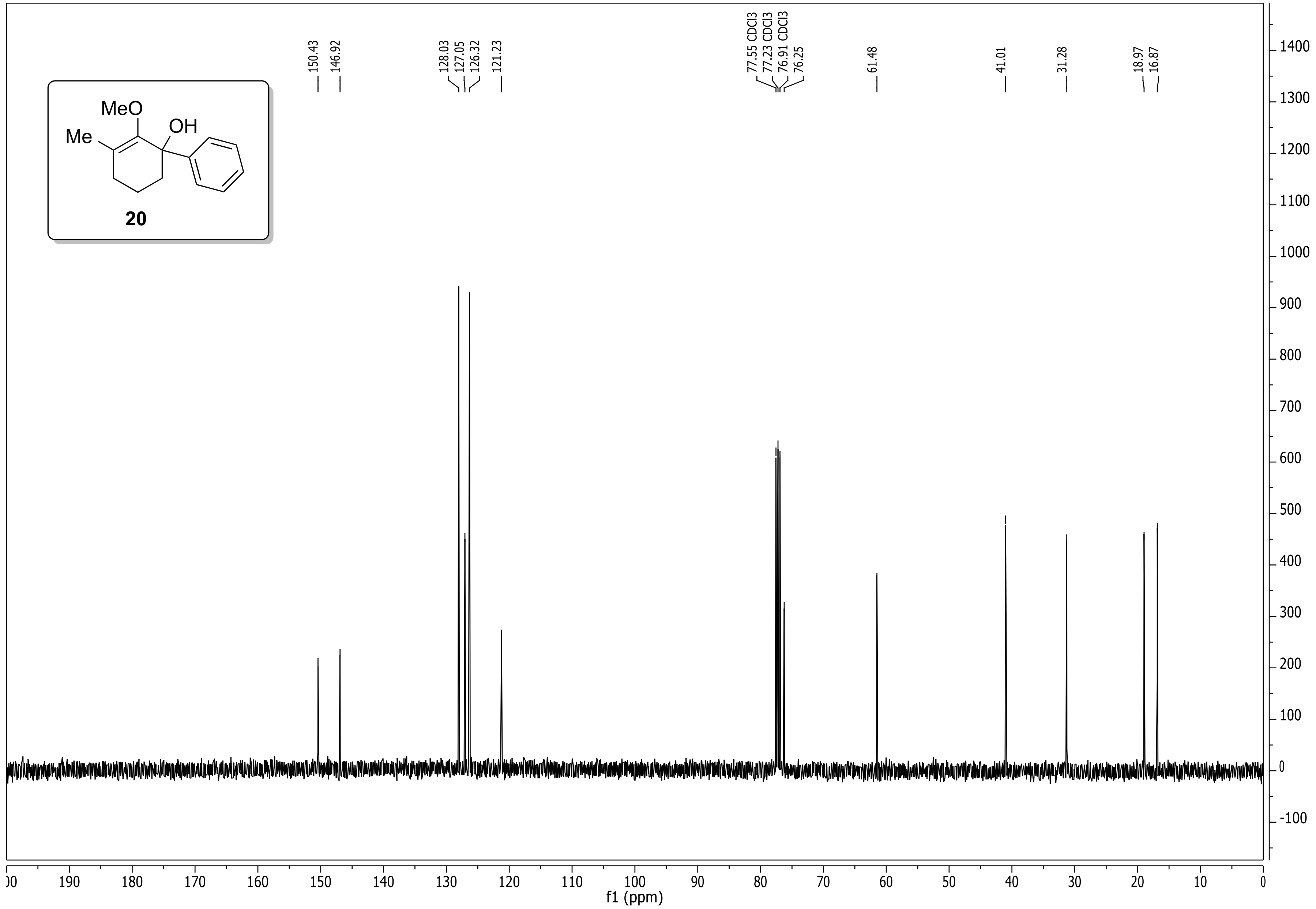
MeO
OH
Me
20
150.43
146.92
128.03
127.05
126.32
121.23
77.55 CDCl3
77.23 CDCl3
76.91 CDCl3
76.25
61.48
41.01
31.28
18.97
16.87
f1 (ppm)

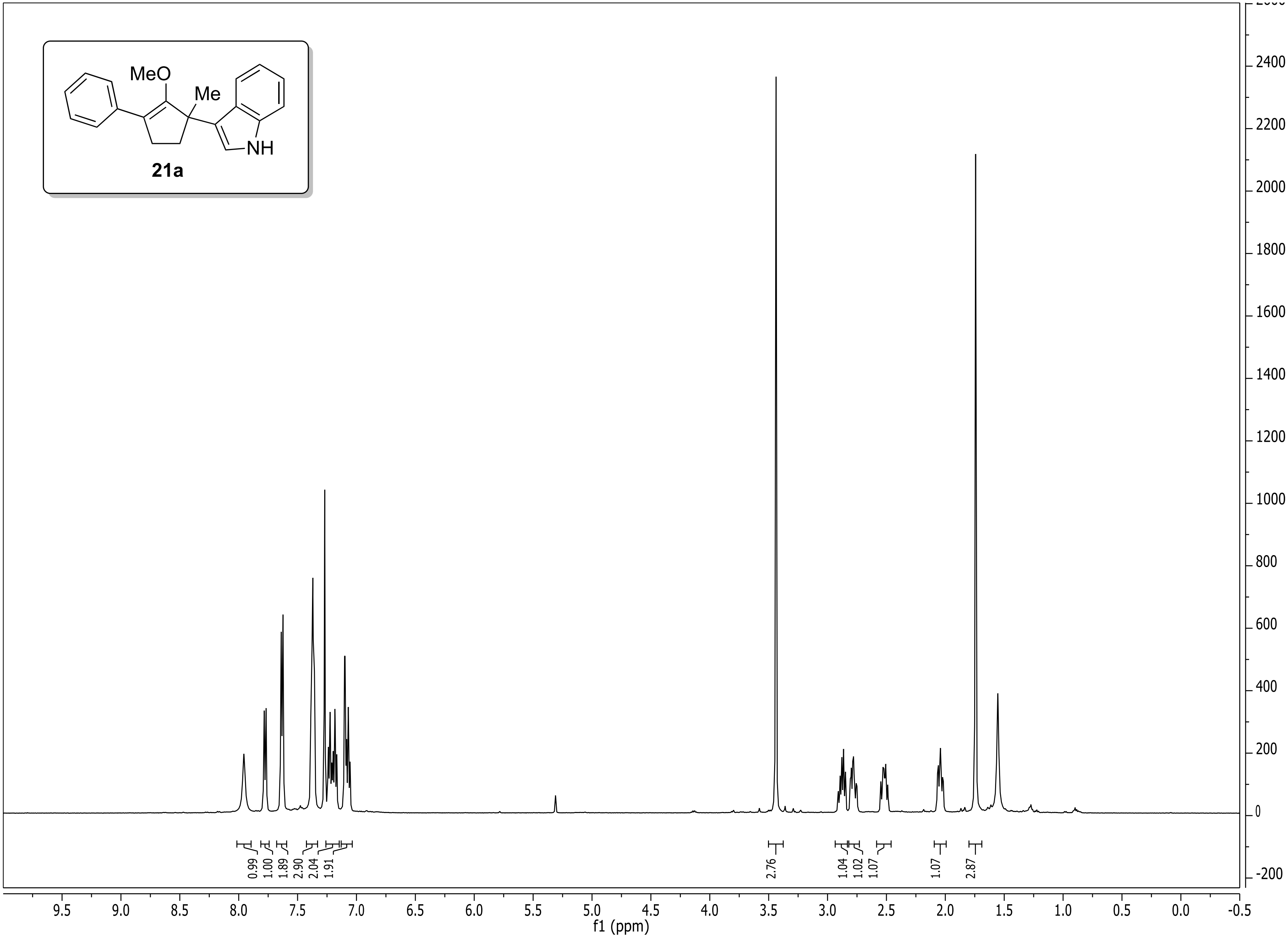

MeO
Me
NH
21a
0.99
1.00
1.89
2.90
2.04
1.91
2.76
1.04
1.02
1.07
1.07
2.87
f1 (ppm)

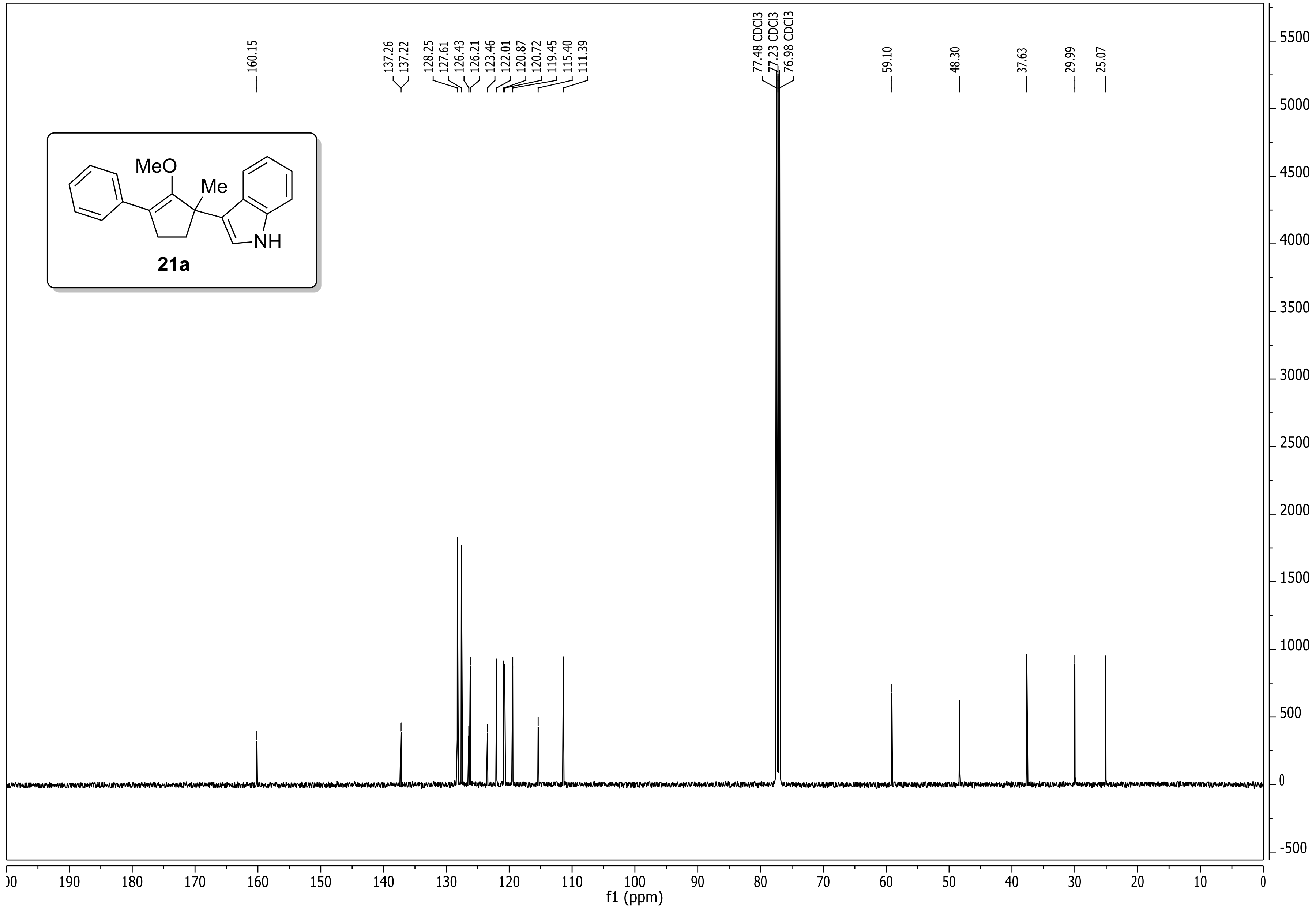
160.15
137.26
137.22
128.25
127.61
126.43
126.21
123.46
122.01
120.87
120.72
119.45
115.40
111.39
77.48 CDCl3
77.23 CDCl3
76.98 CDCl3
59.10
48.30
37.63
29.99
25.07
MeO
Me
NH
21a
f1 (ppm)

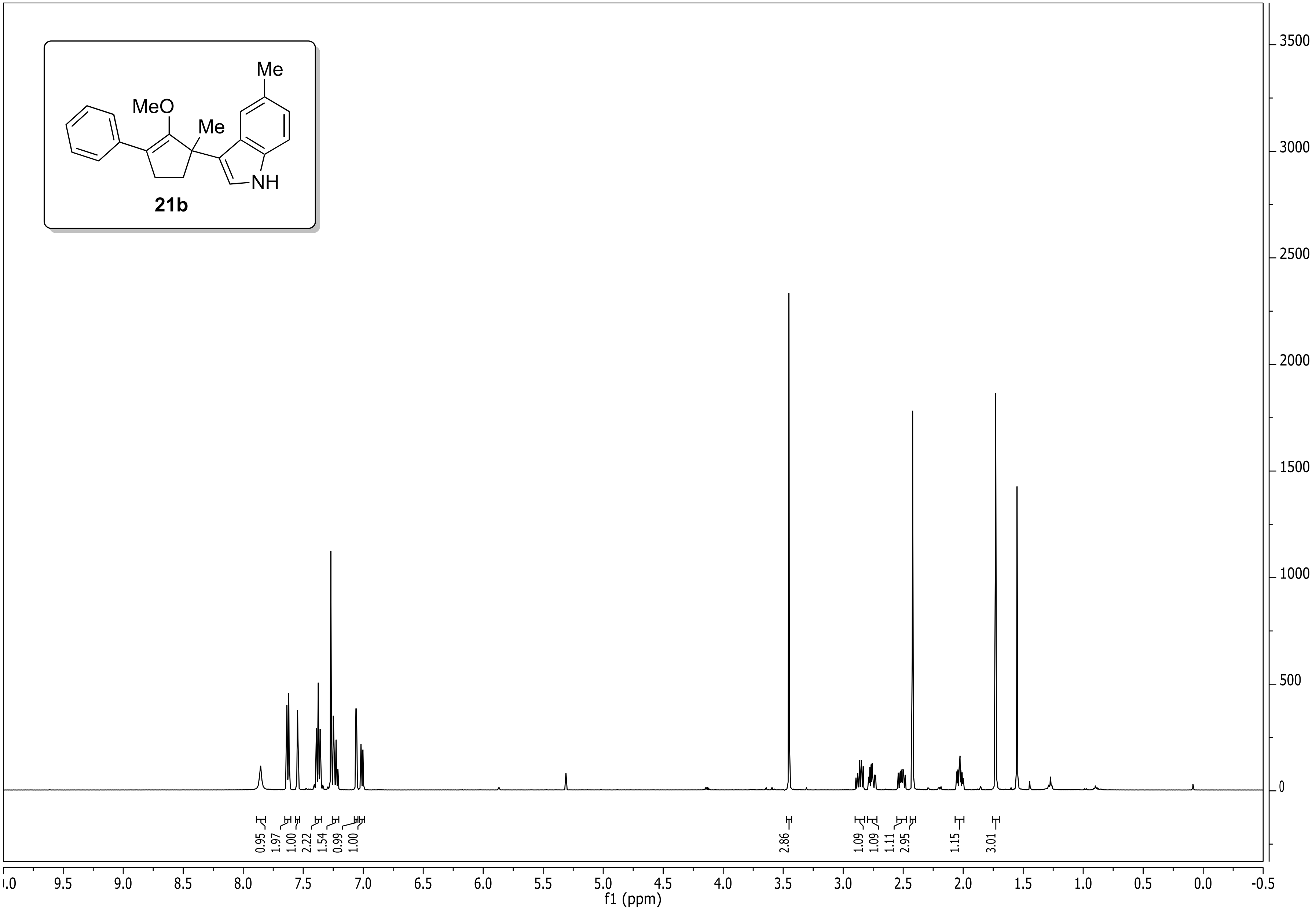
Me
MeO
Me
NH
21b
3500
3000
2500
2000
1500
1000
500
0
0.95
1.97
1.00
2.22
1.54
0.99
1.00
2.86
1.09
1.09
1.11
2.95
1.15
3.01
9.5
9.0
8.5
8.0
7.5
7.0
6.5
6.0
5.5
5.0
4.5
4.0
3.5
3.0
2.5
2.0
1.5
1.0
0.5
0.0
-0.5
f1 (ppm)

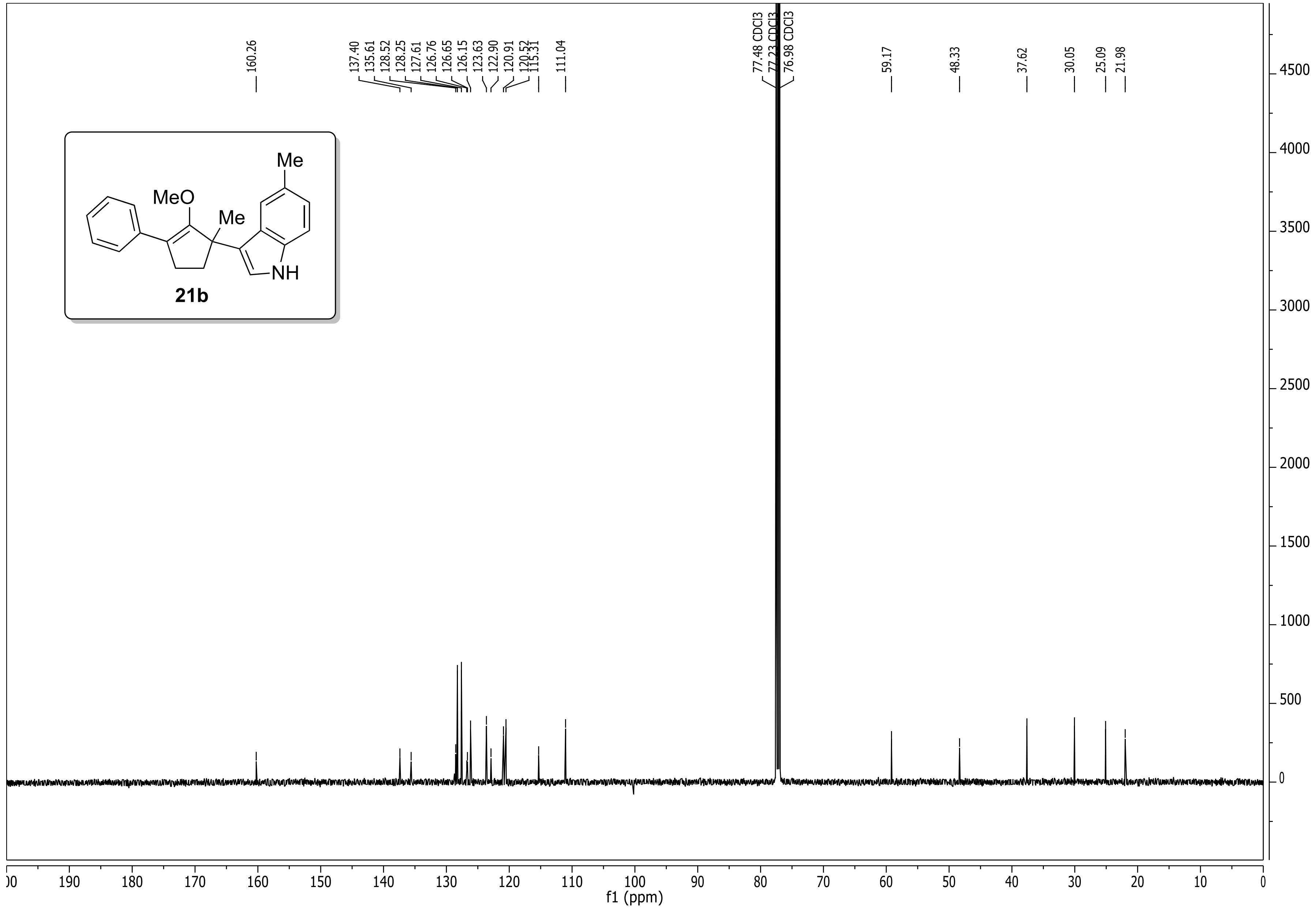
160.26
137.40
135.61
128.52
128.25
127.61
126.76
126.65
126.15
123.63
122.90
120.91
120.52
115.31
111.04
77.48 CDCl3
77.23 CDCl3
76.98 CDCl3
59.17
48.33
37.62
30.05
25.09
21.98
MeO
Me
Me
NH
21b
f1 (ppm)

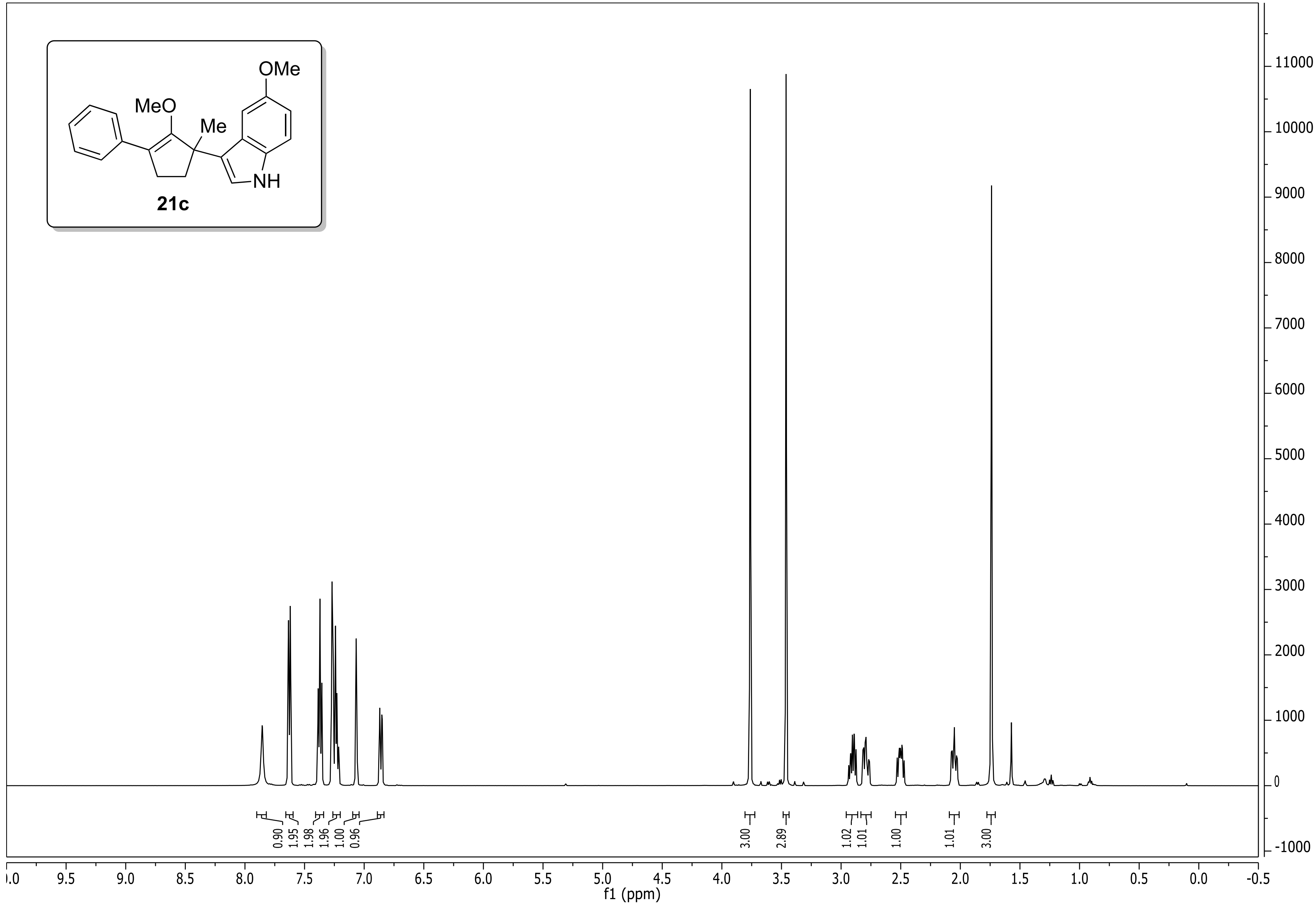
OMe
MeO
Me
NH
21c
0.90 1.95 1.98 1.96 1.00 0.96
3.00 2.89
1.02 1.01 1.00 1.01 3.00
f1 (ppm)

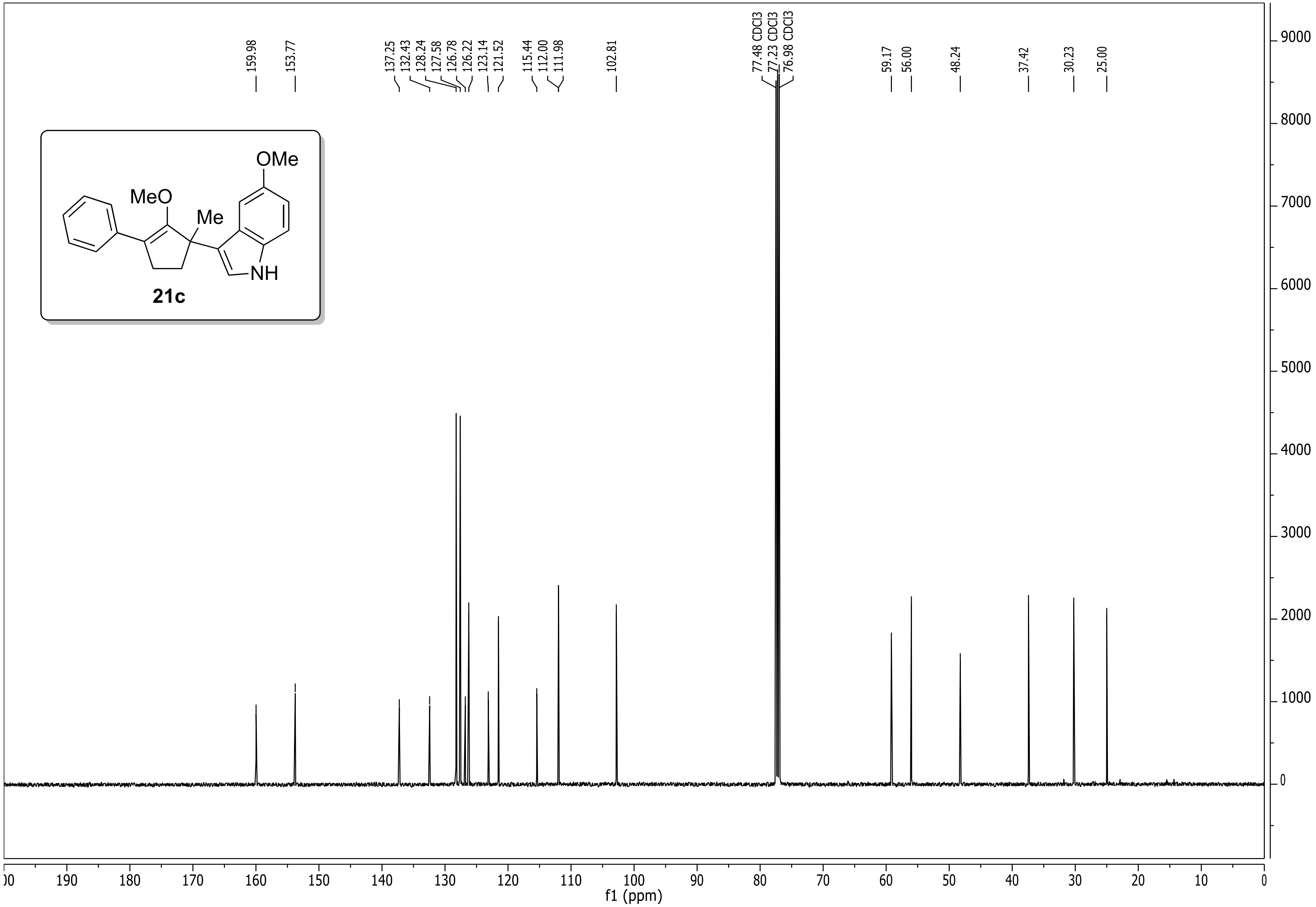
159.98
153.77
137.25
132.43
128.24
127.58
126.78
126.22
123.14
121.52
115.44
112.00
111.98
102.81
77.48 CDCl3
77.23 CDCl3
76.98 CDCl3
59.17
56.00
48.24
37.42
30.23
25.00
OMe
MeO
Me
NH
21c
f1 (ppm)

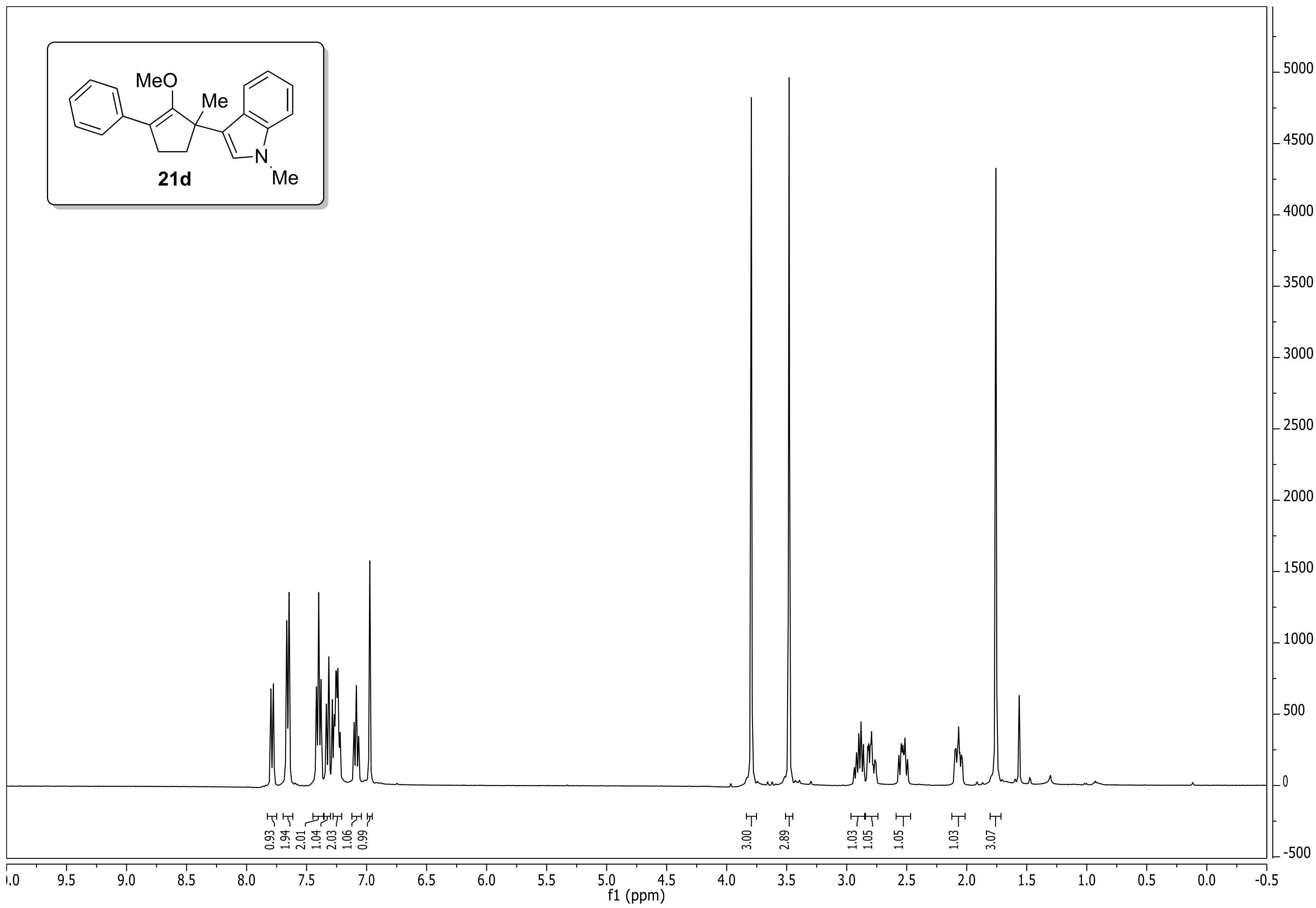

MeO
Me
N
Me
21d
0.93
1.94
2.01
1.04
2.03
1.06
0.99
3.00
2.89
1.03
1.05
1.05
1.03
3.07
f1 (ppm)

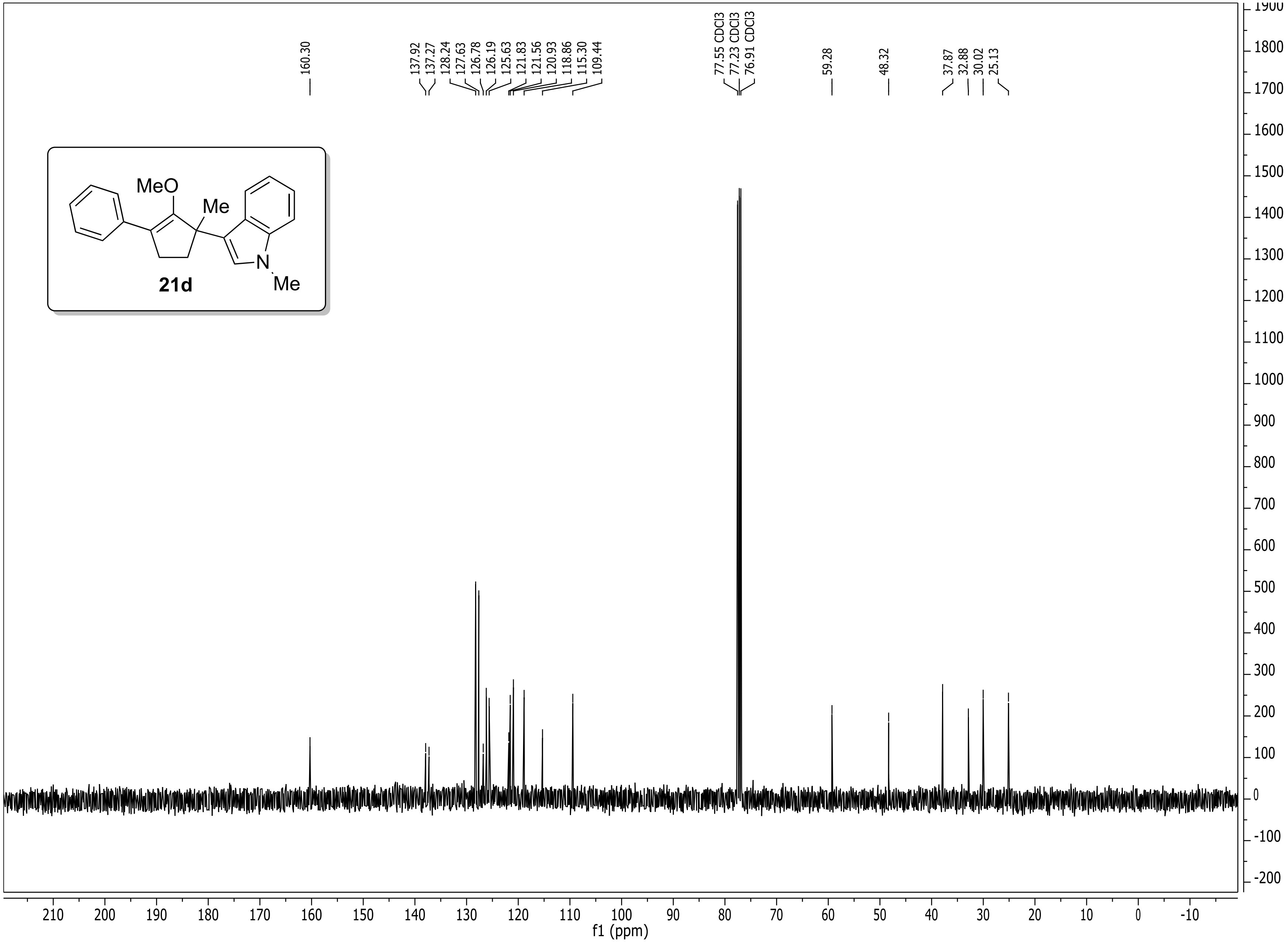

160.30
137.92
137.27
128.24
127.63
126.78
126.19
125.63
121.83
121.56
120.93
118.86
115.30
109.44
77.55 CDCl3
77.23 CDCl3
76.91 CDCl3
59.28
48.32
37.87
32.88
30.02
25.13
MeO
Me
N
Me
21d
f1 (ppm)

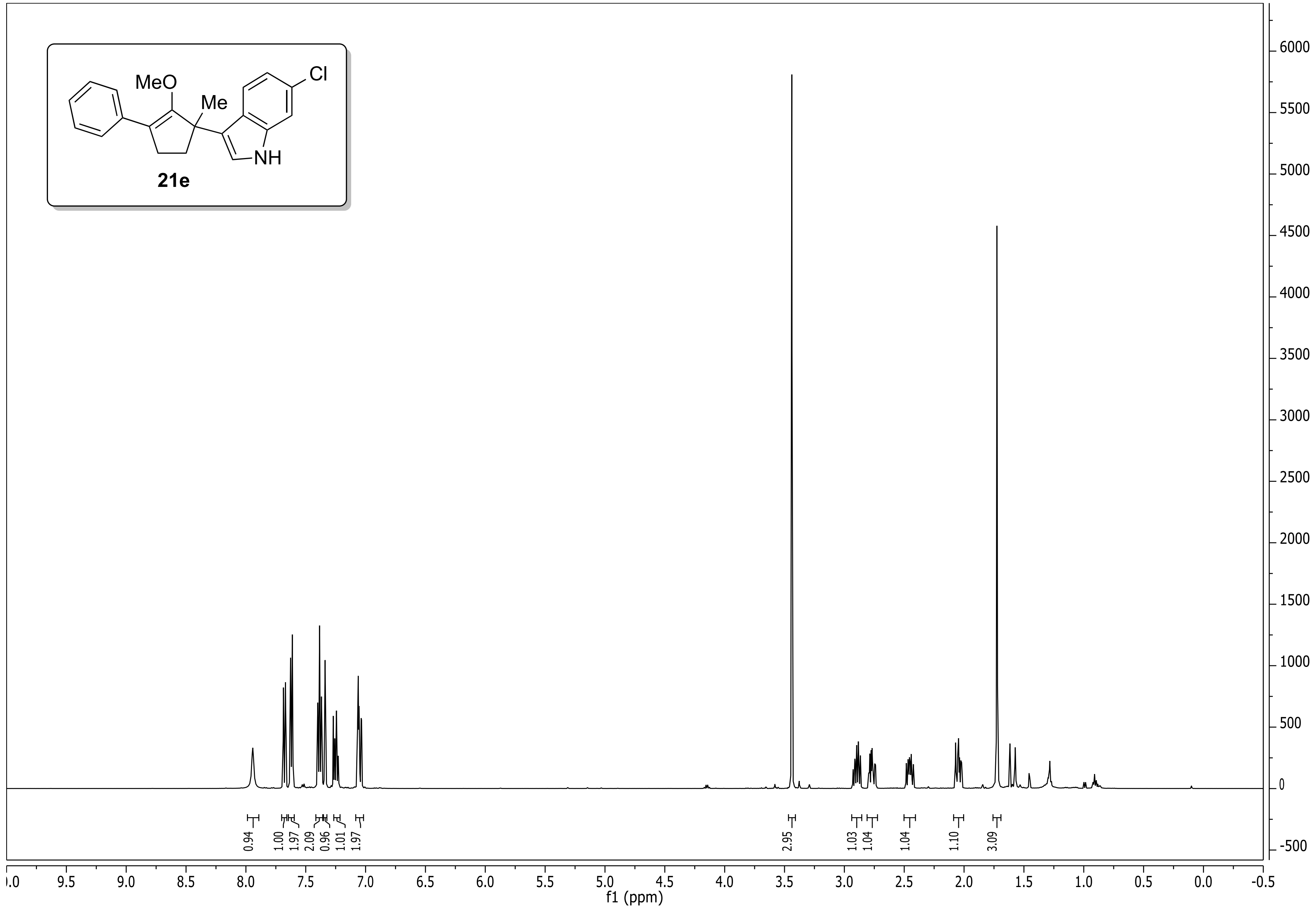

MeO
Me
Cl
NH
21e
0.94
1.00
1.97
2.09
0.96
1.01
1.97
2.95
1.03
1.04
1.04
1.10
3.09
f1 (ppm)

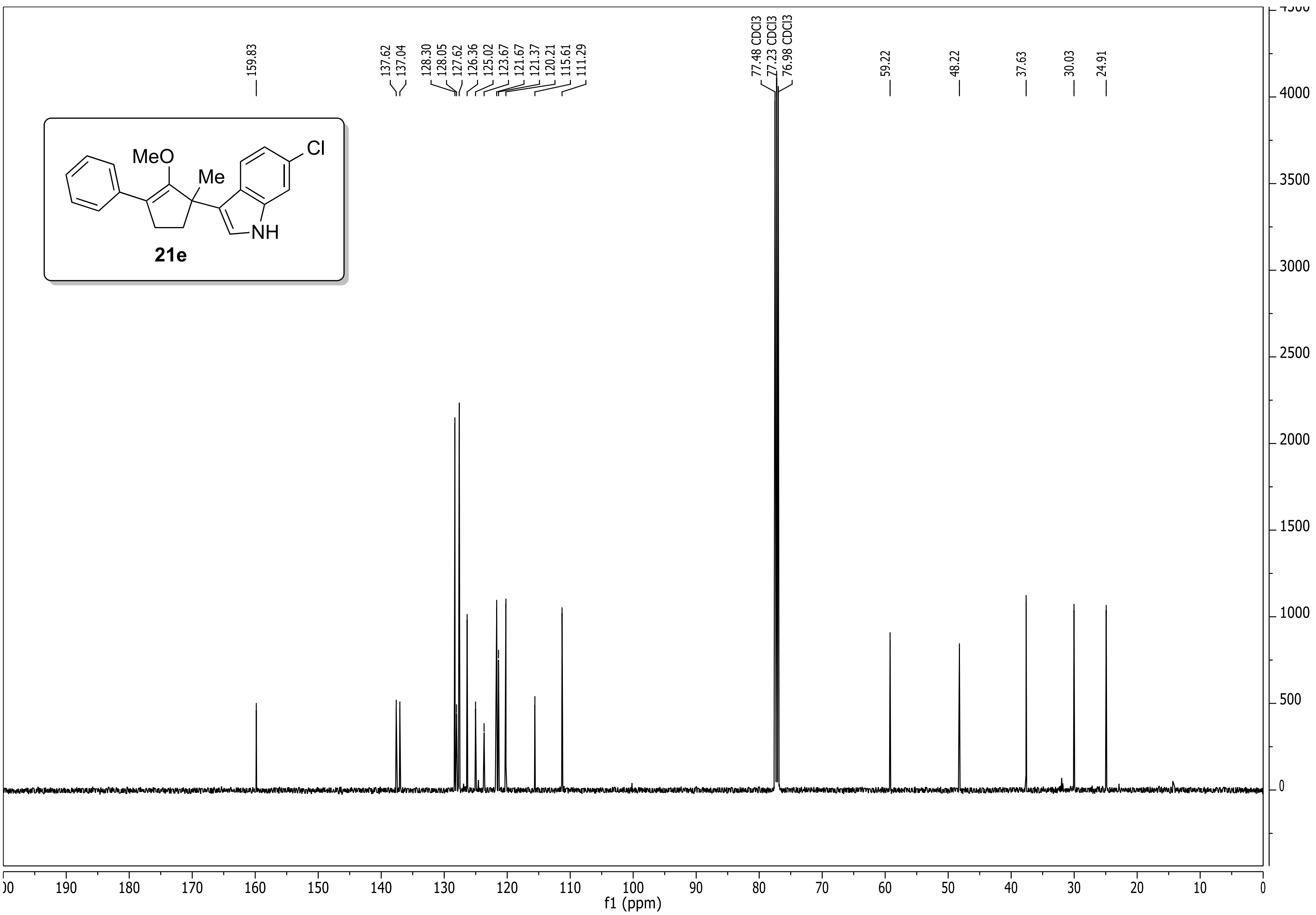
159.83
137.62
137.04
128.30
128.05
127.62
126.36
125.02
123.67
121.67
121.37
120.21
115.61
111.29
77.48 CDCl3
77.23 CDCl3
76.98 CDCl3
59.22
48.22
37.63
30.03
24.91
MeO
Me
Cl
NH
21e
f1 (ppm)

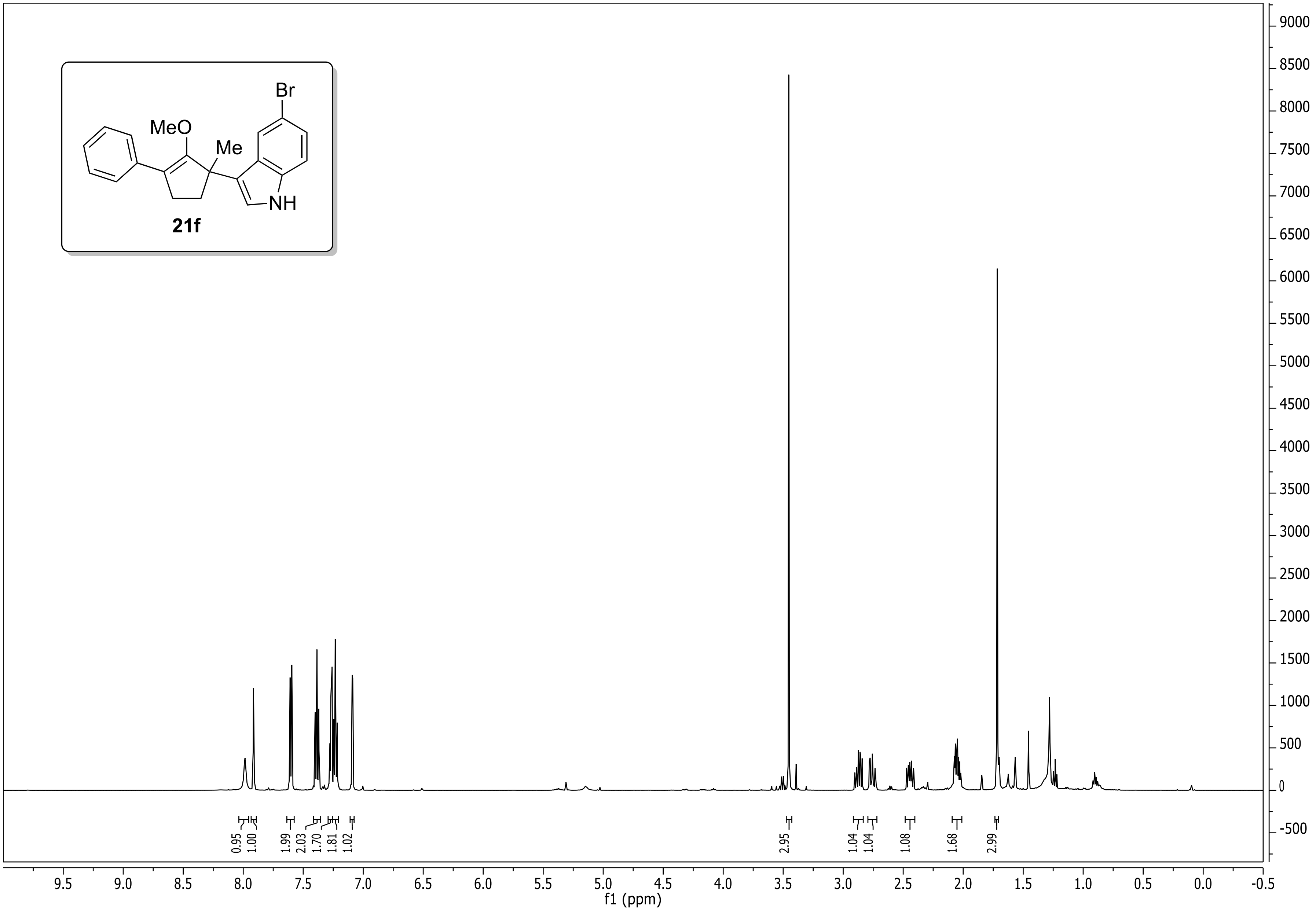
MeO
Me
Br
NH
21f
f1 (ppm)

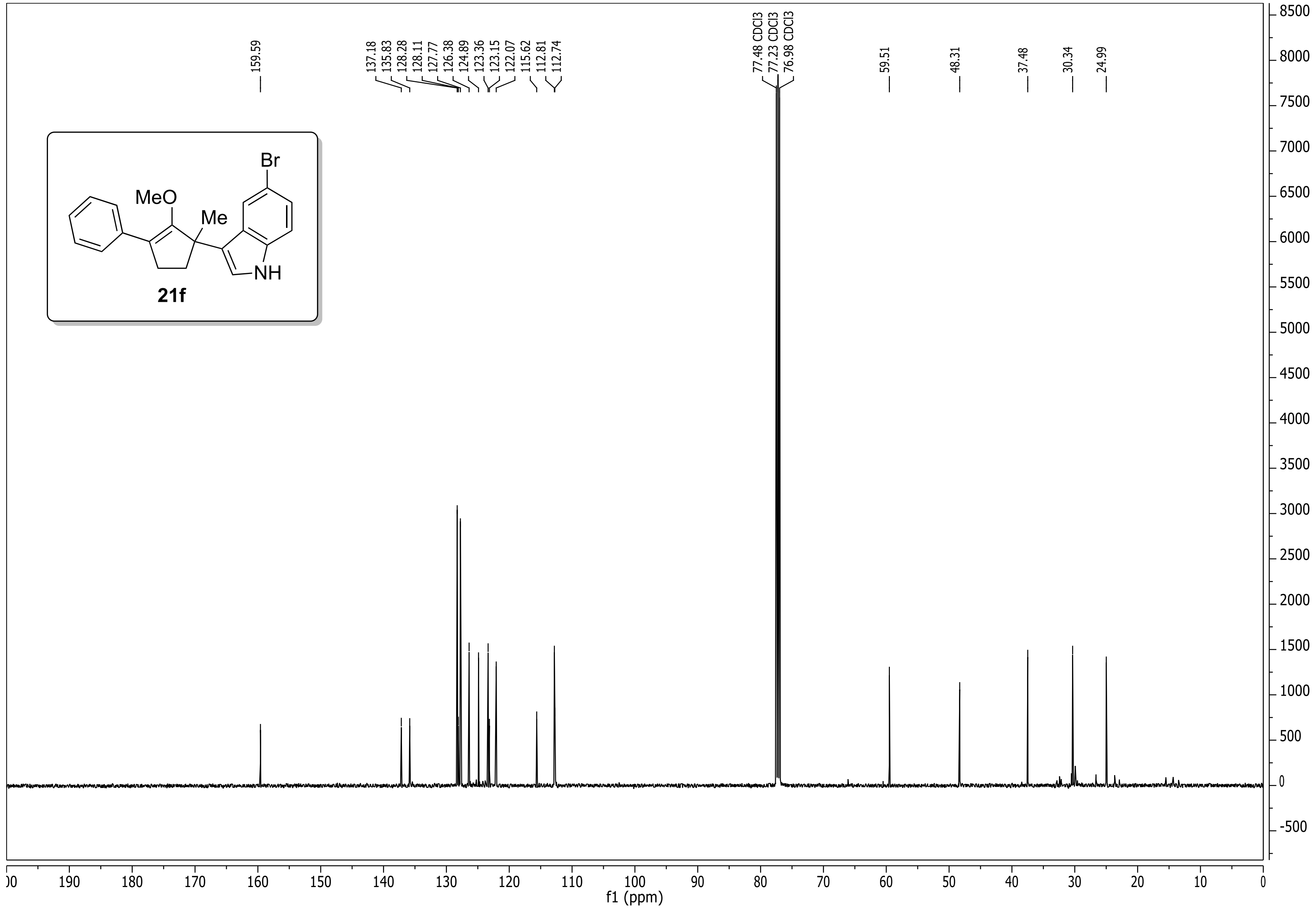

159.59
137.18
135.83
128.28
128.11
127.77
126.38
124.89
123.36
123.15
122.07
115.62
112.81
112.74
77.48 CDCl3
77.23 CDCl3
76.98 CDCl3
59.51
48.31
37.48
30.34
24.99
Br
MeO
Me
NH
21f
f1 (ppm)

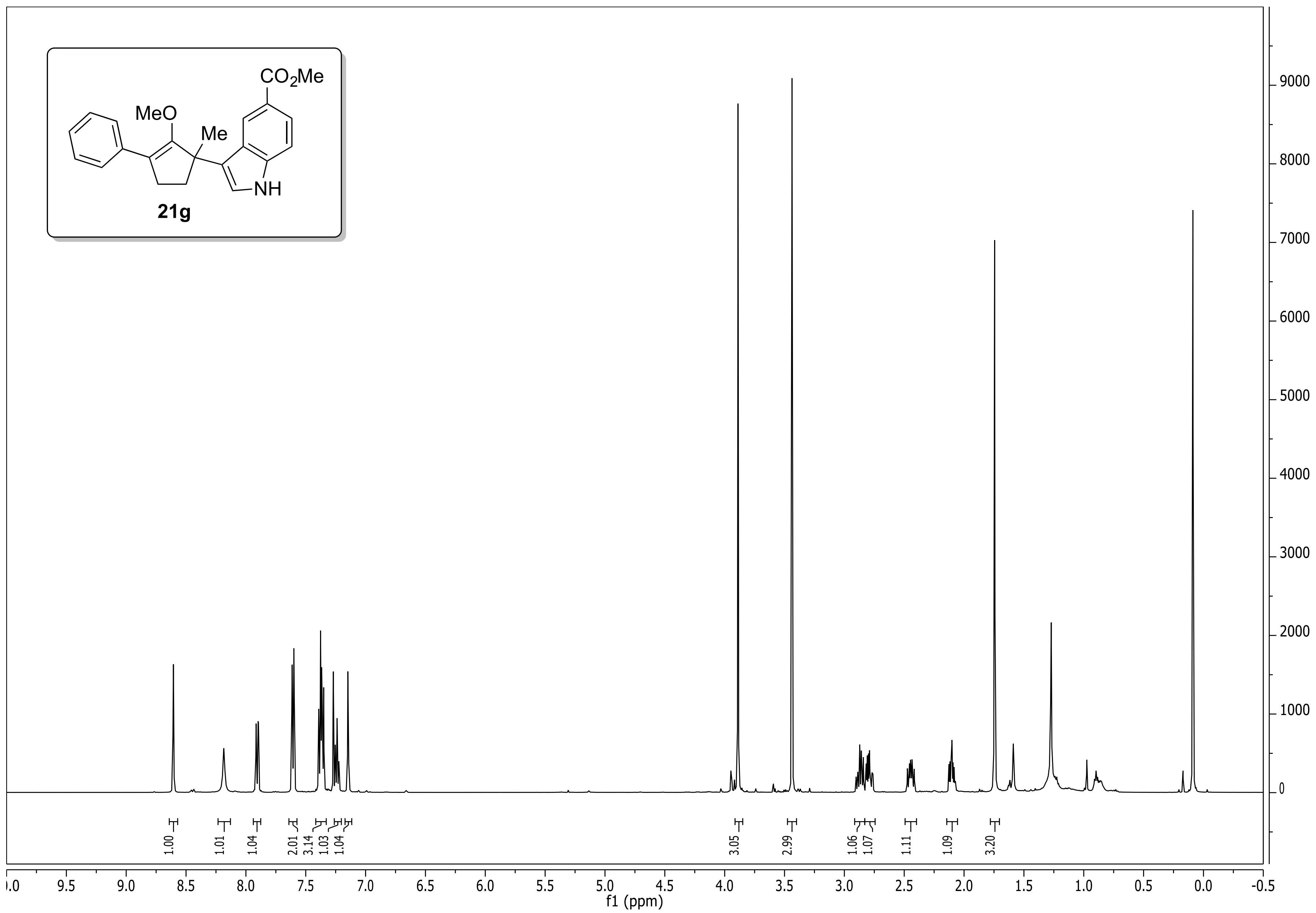
CO2Me
MeO
Me
NH
21g
1.00
1.01
1.04
2.01
3.14
1.03
1.04
3.05
2.99
1.06
1.07
1.11
1.09
3.20
f1 (ppm)

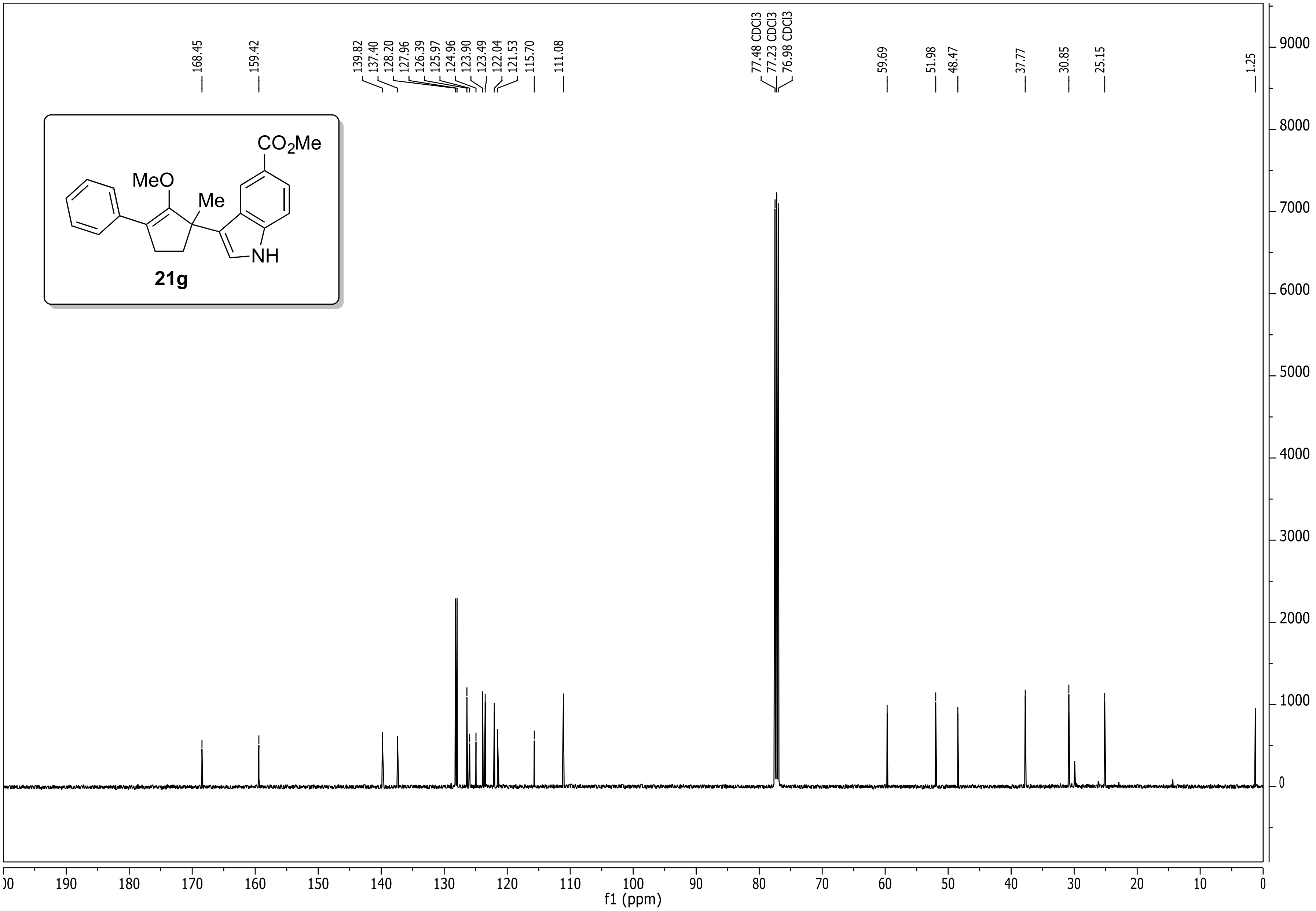
168.45
159.42
139.82
137.40
128.20
127.96
126.39
125.97
124.96
123.90
123.49
122.04
121.53
115.70
111.08
77.48 CDCl3
77.23 CDCl3
76.98 CDCl3
59.69
51.98
48.47
37.77
30.85
25.15
1.25
CO2Me
MeO
Me
NH
21g
f1 (ppm)

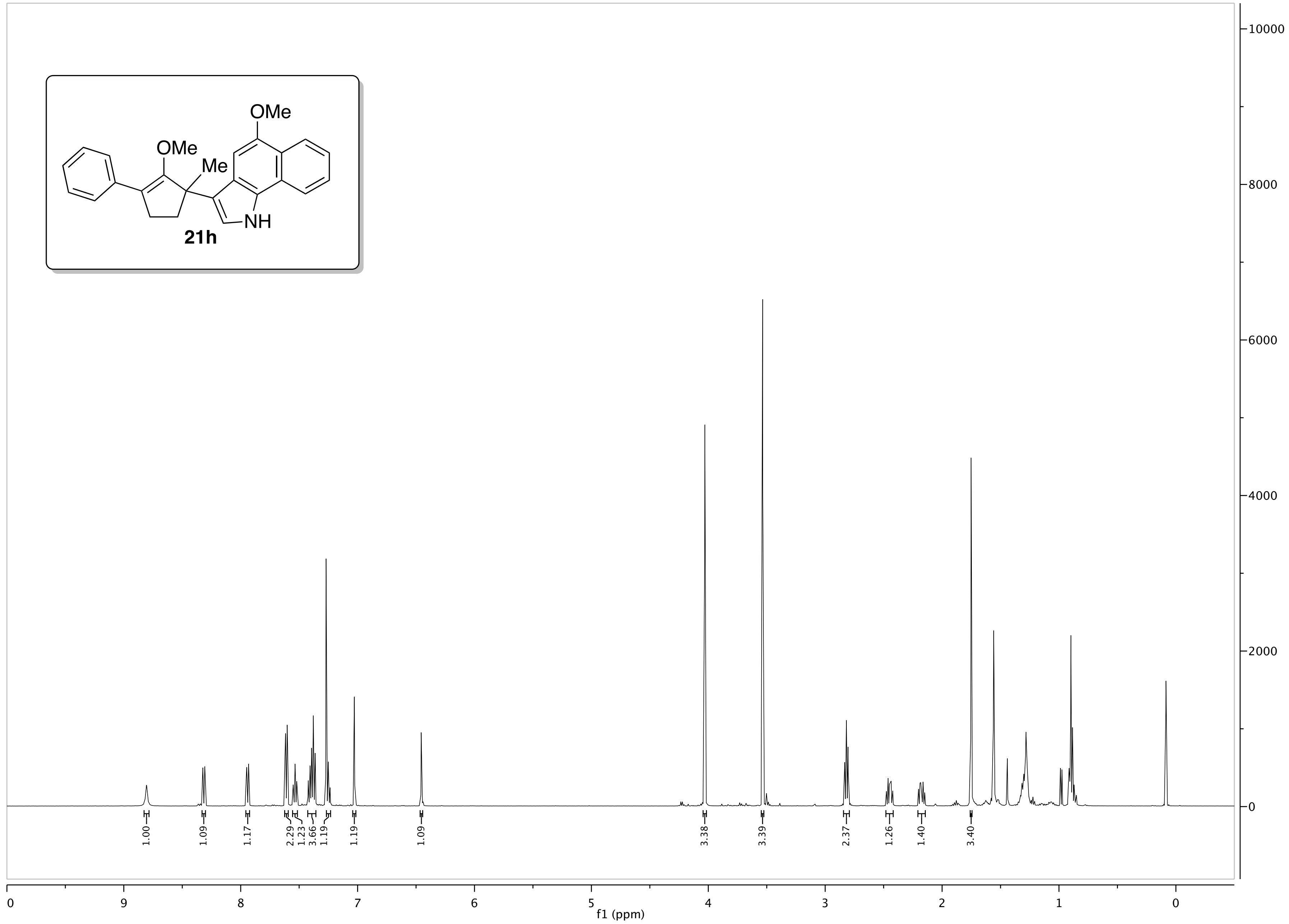
OMe
OMe
Me
NH
21h
1.00
1.09
1.17
2.29
1.23
3.66
1.19
1.19
1.09
3.38
3.39
2.37
1.26
1.40
3.40
f1 (ppm)

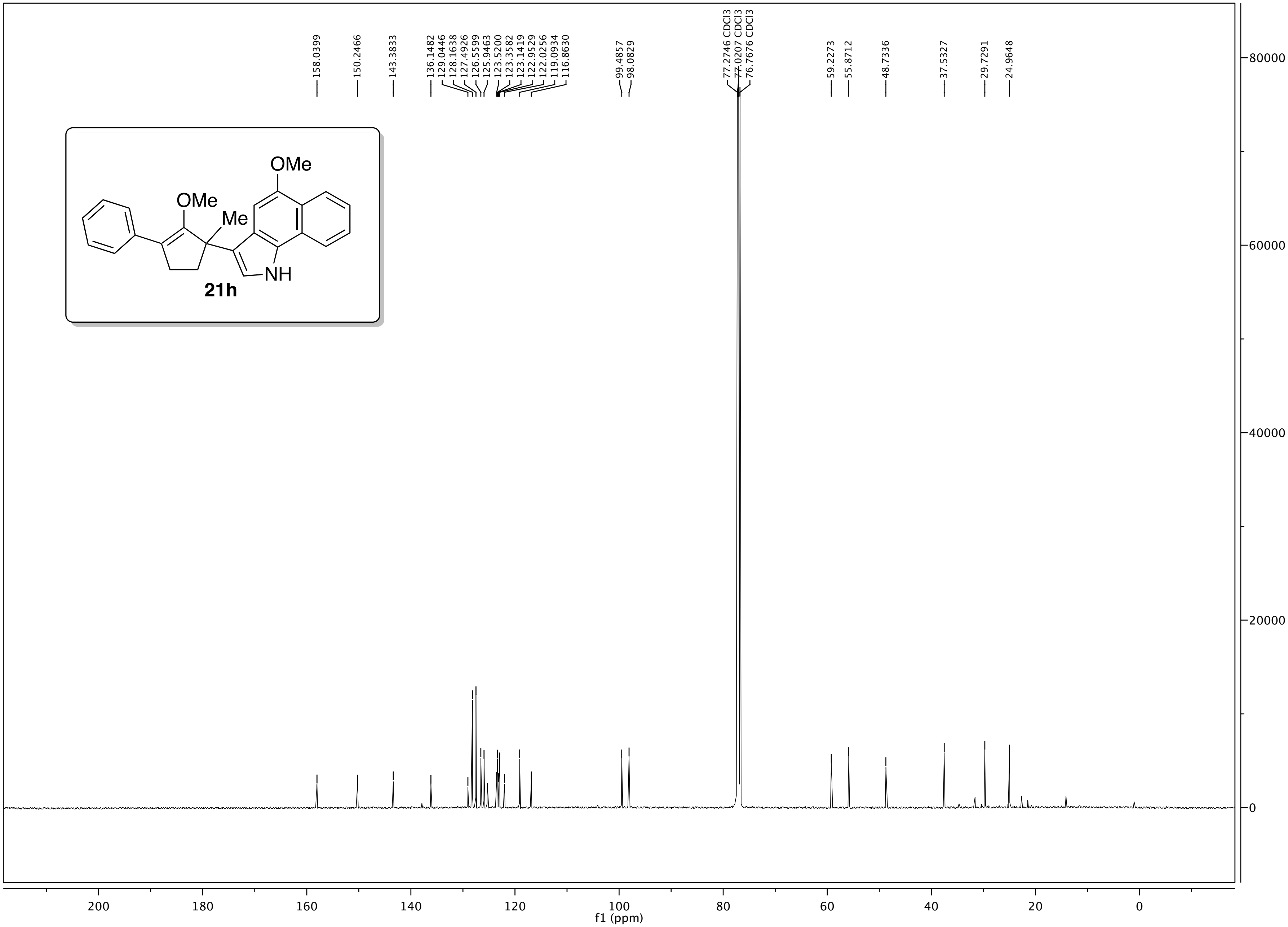
158.0399
150.2466
143.3833
136.1482
129.0446
128.1638
127.4926
126.5599
125.9463
123.5200
123.3582
123.1419
122.9529
122.0256
119.0934
116.8630
99.4857
98.0829
77.2746 CDCl3
77.0207 CDCl3
76.7676 CDCl3
59.2273
55.8712
48.7336
37.5327
29.7291
24.9648
OMe
OMe
Me
NH
21h
80000
60000
40000
20000
0
200
180
160
140
120
100
80
60
40
20
0
f1 (ppm)

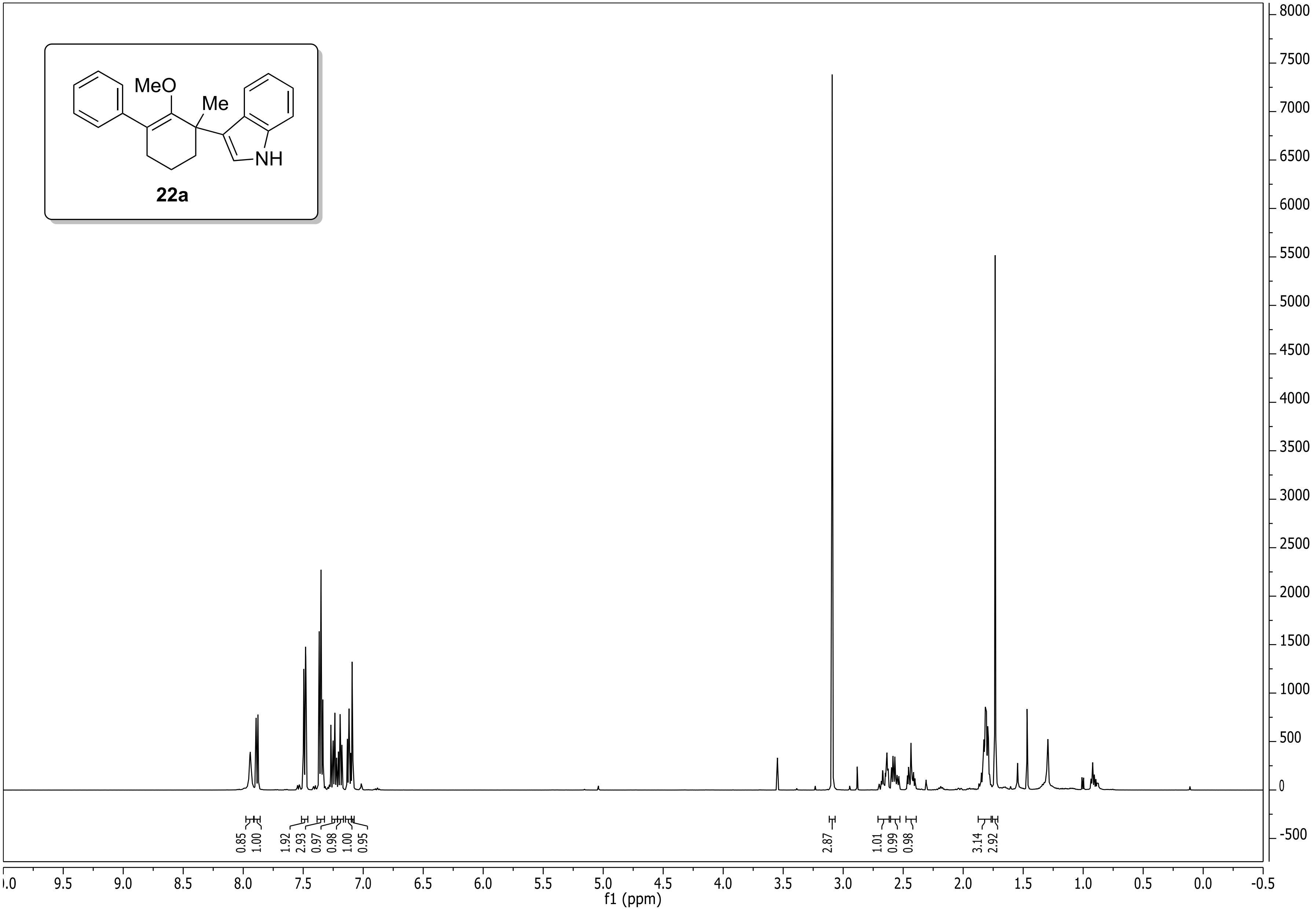
MeO
Me
NH
22a

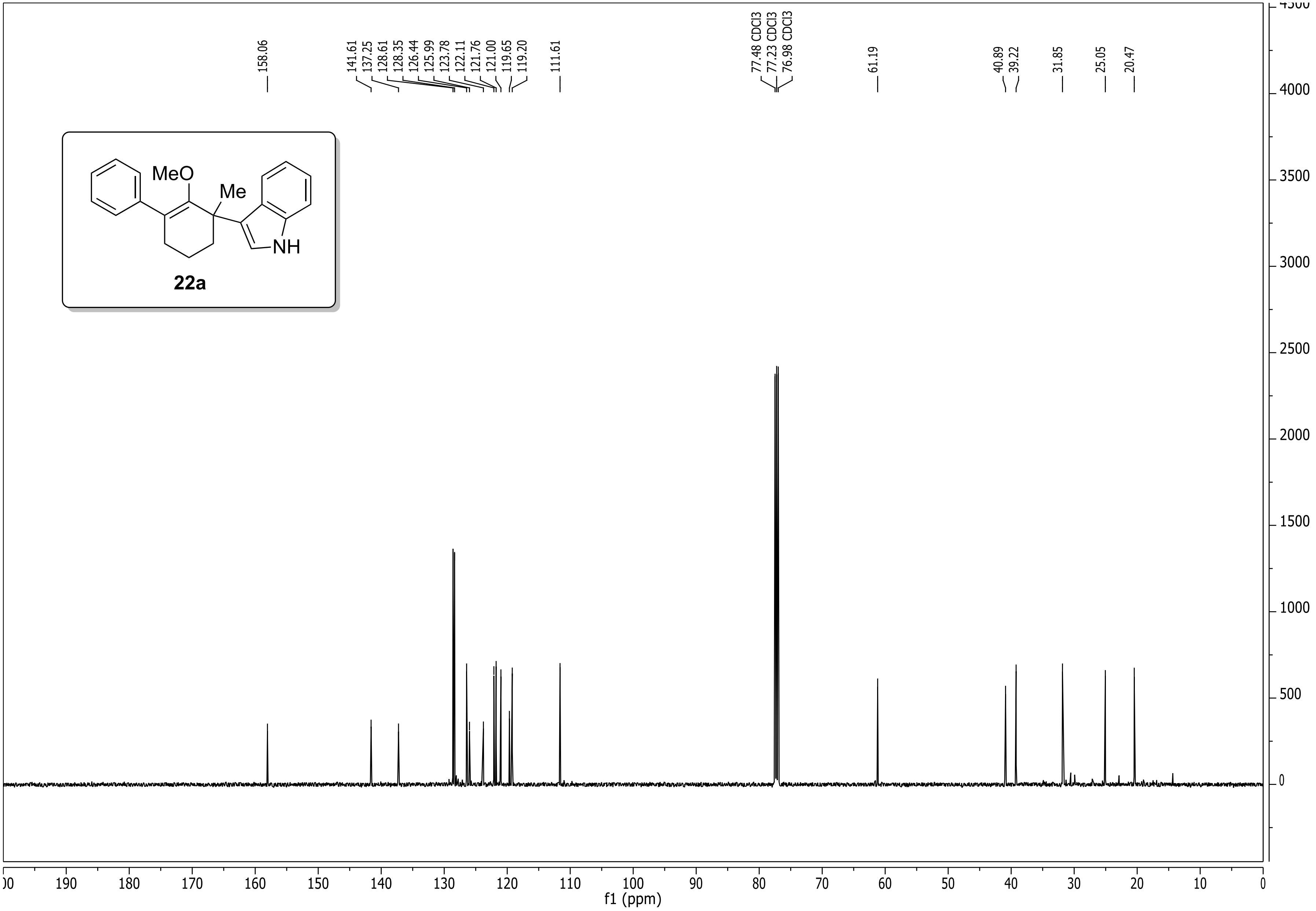

158.06
141.61
137.25
128.61
128.35
126.44
125.99
123.78
122.11
121.76
121.00
119.65
119.20
111.61
77.48 CDCl3
77.23 CDCl3
76.98 CDCl3
61.19
40.89
39.22
31.85
25.05
20.47
MeO
Me
NH
22a
f1 (ppm)

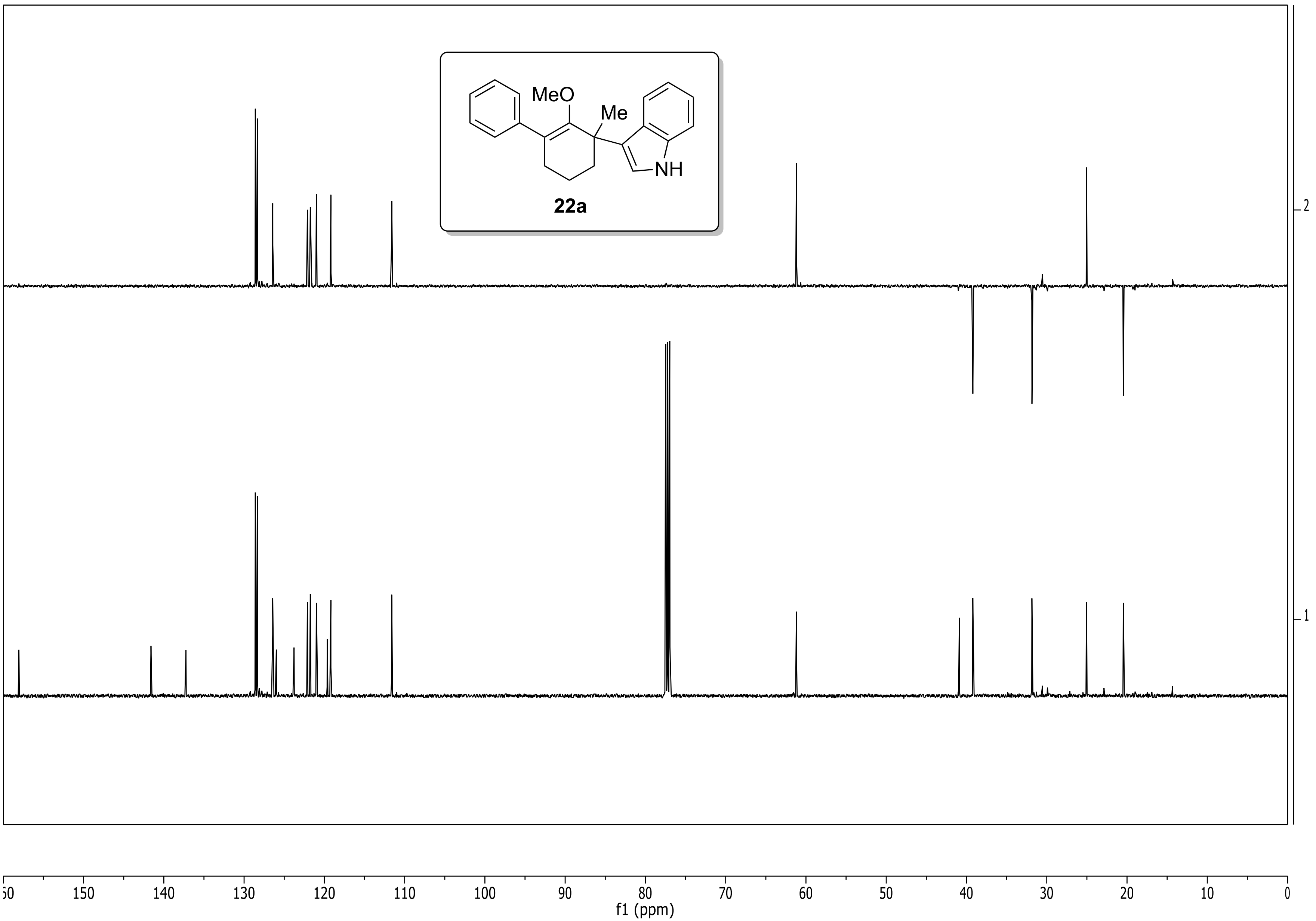
MeO
Me
NH
22a
2
1
150
140
130
120
110
100
90
80
70
60
50
40
30
20
10
0
f1 (ppm)

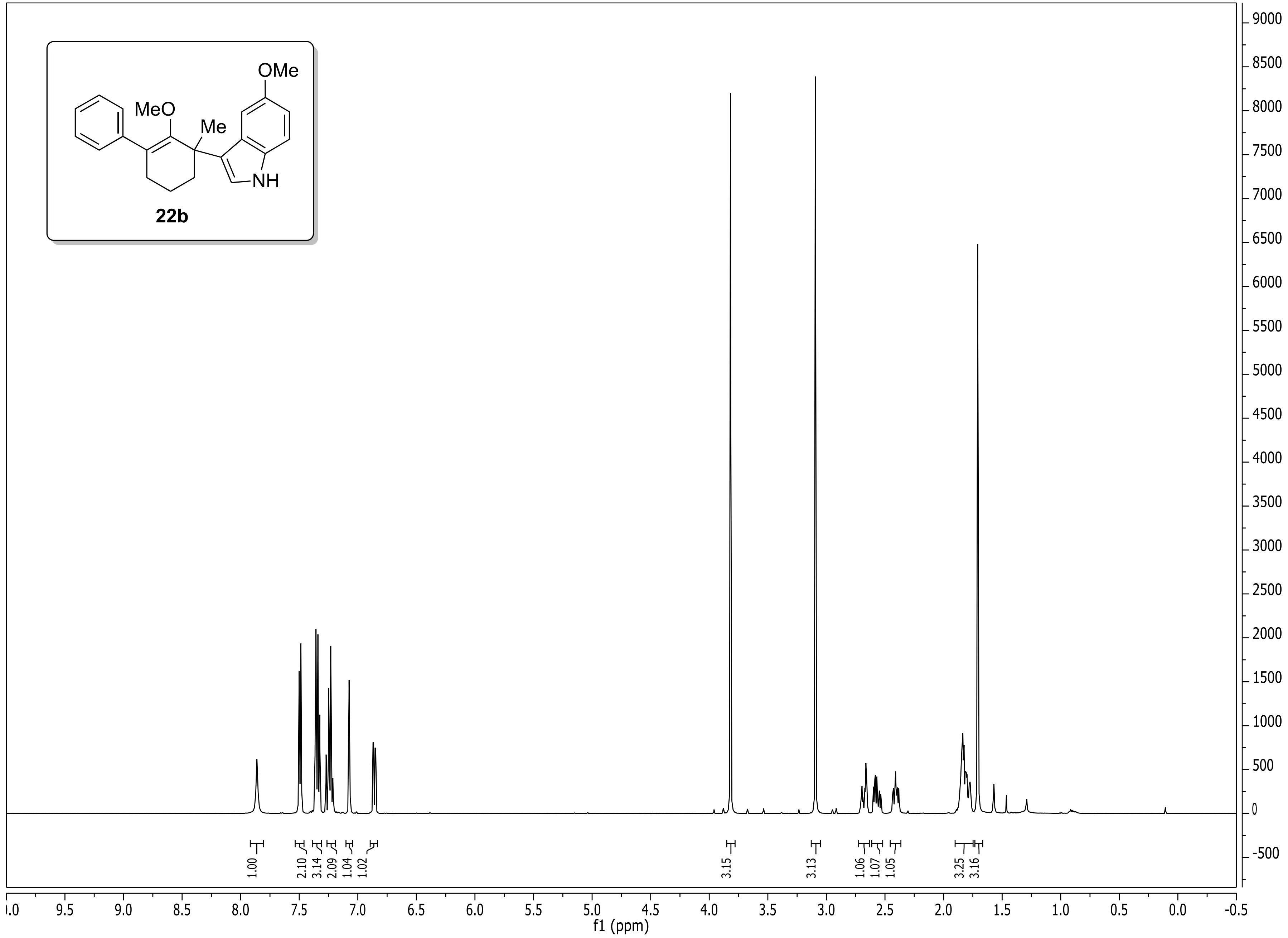
OMe
MeO
Me
NH
22b
1.00
2.10
3.14
2.09
1.04
1.02
3.15
3.13
1.06
1.07
1.05
3.25
3.16
f1 (ppm)

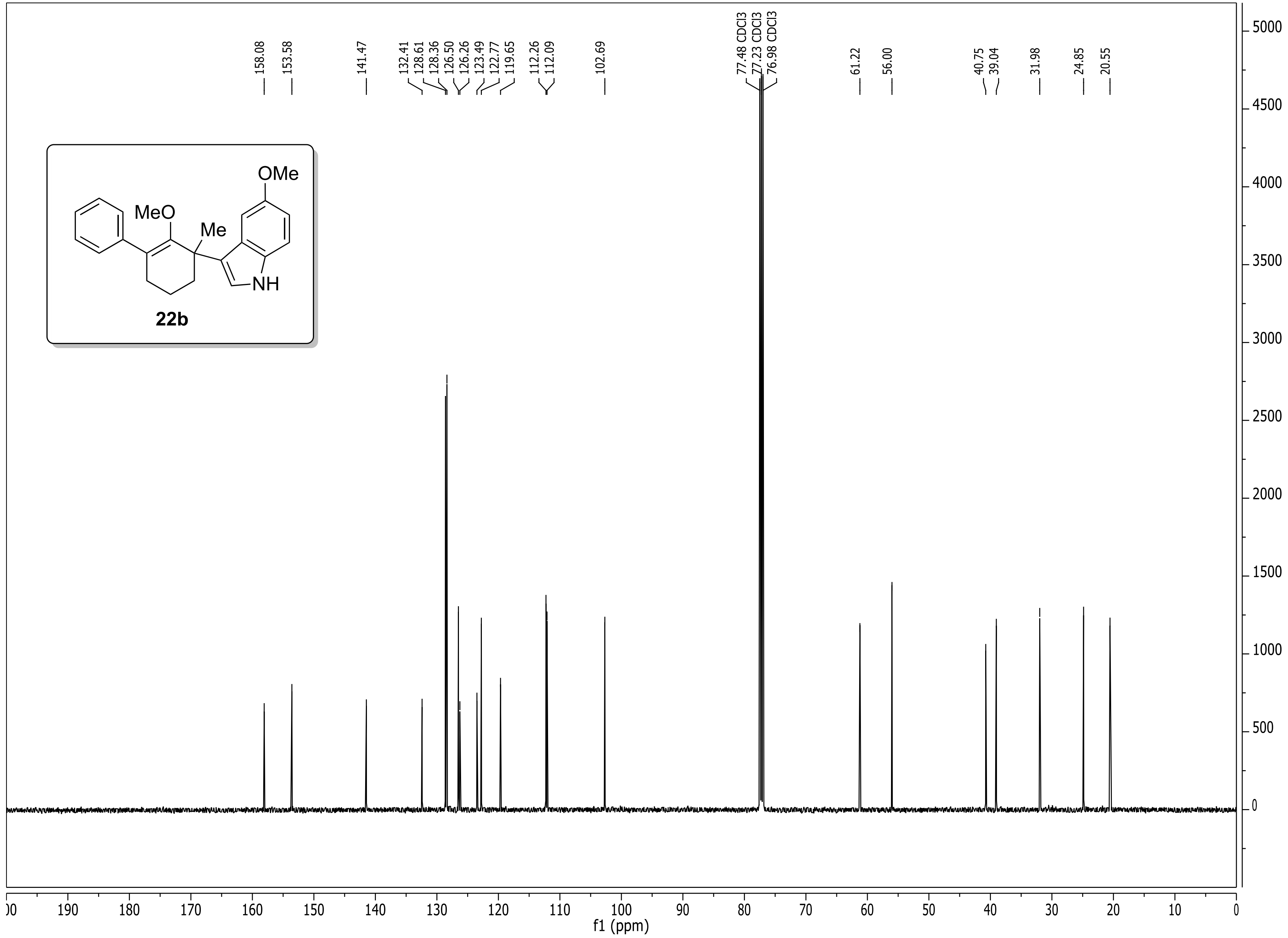
158.08
153.58
141.47
132.41
128.61
128.36
126.50
126.26
123.49
122.77
119.65
112.26
112.09
102.69
77.48 CDCl3
77.23 CDCl3
76.98 CDCl3
61.22
56.00
40.75
39.04
31.98
24.85
20.55
OMe
MeO
Me
NH
22b
f1 (ppm)

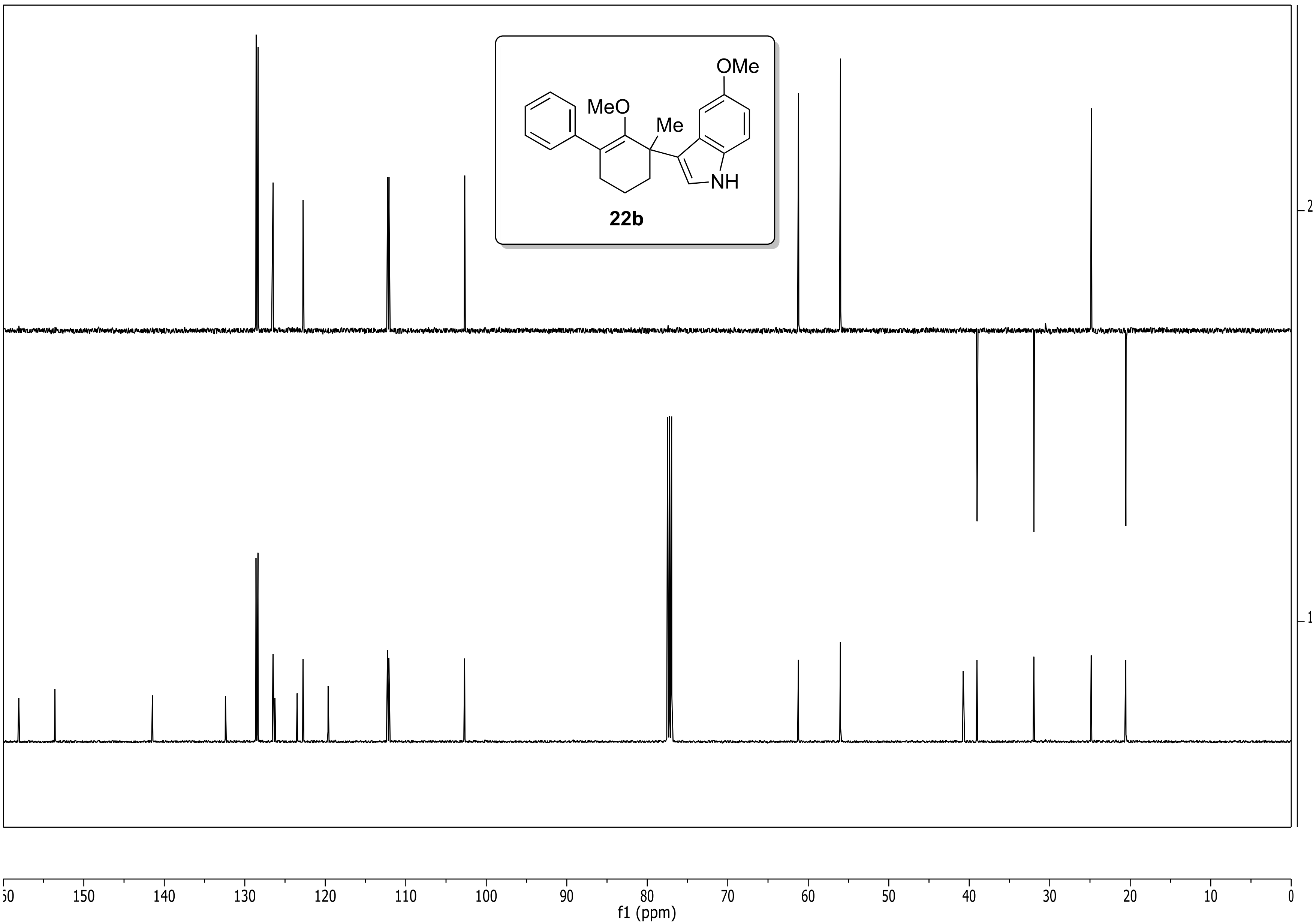

OMe
MeO
Me
NH
22b
2
1
60
150
140
130
120
110
100
90
80
70
60
50
40
30
20
10
0
f1 (ppm)

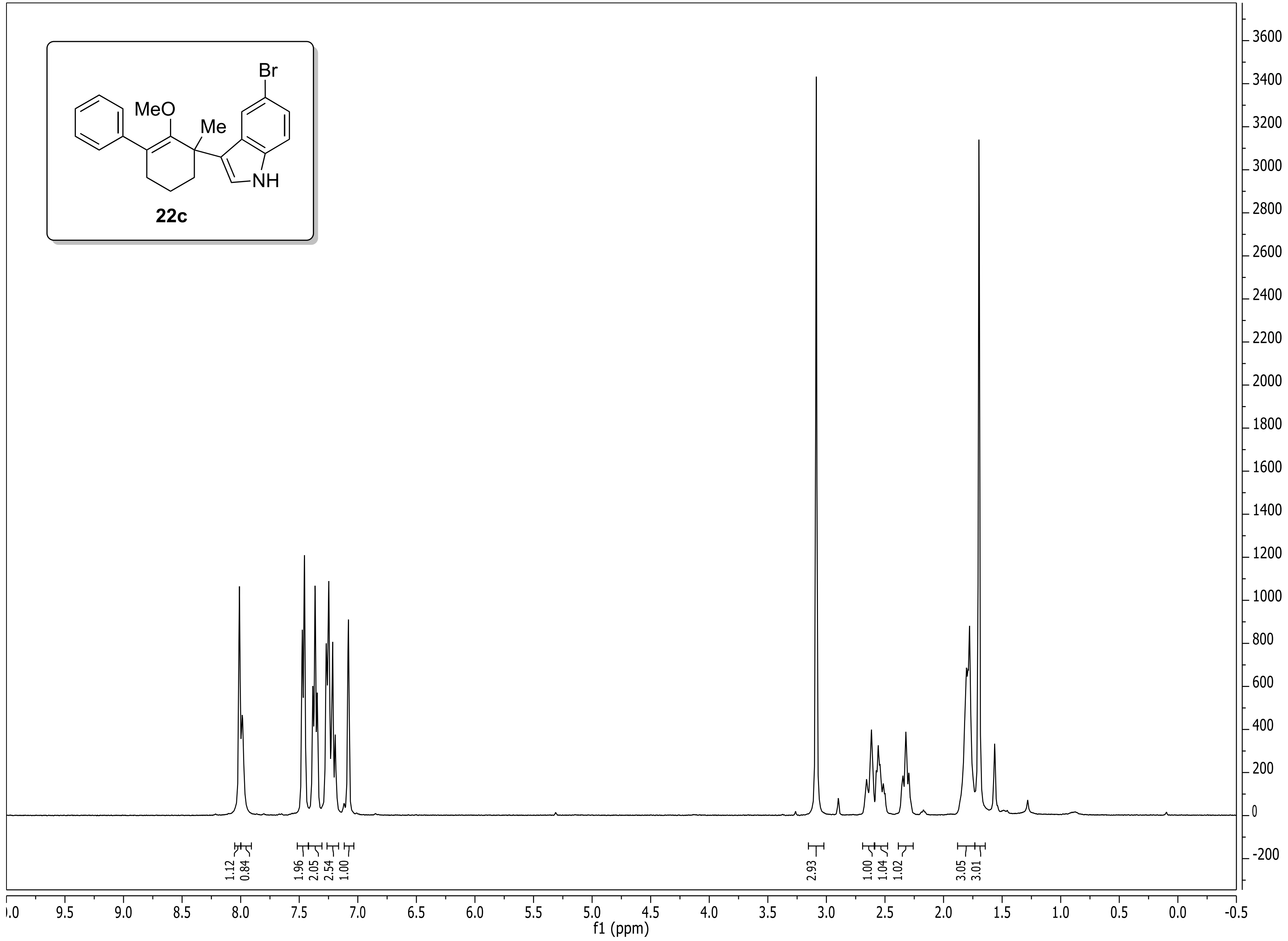

Br
MeO
Me
NH
22c
1.12
0.84
1.96
2.05
2.54
1.00
2.93
1.00
1.04
1.02
3.05
3.01
f1 (ppm)

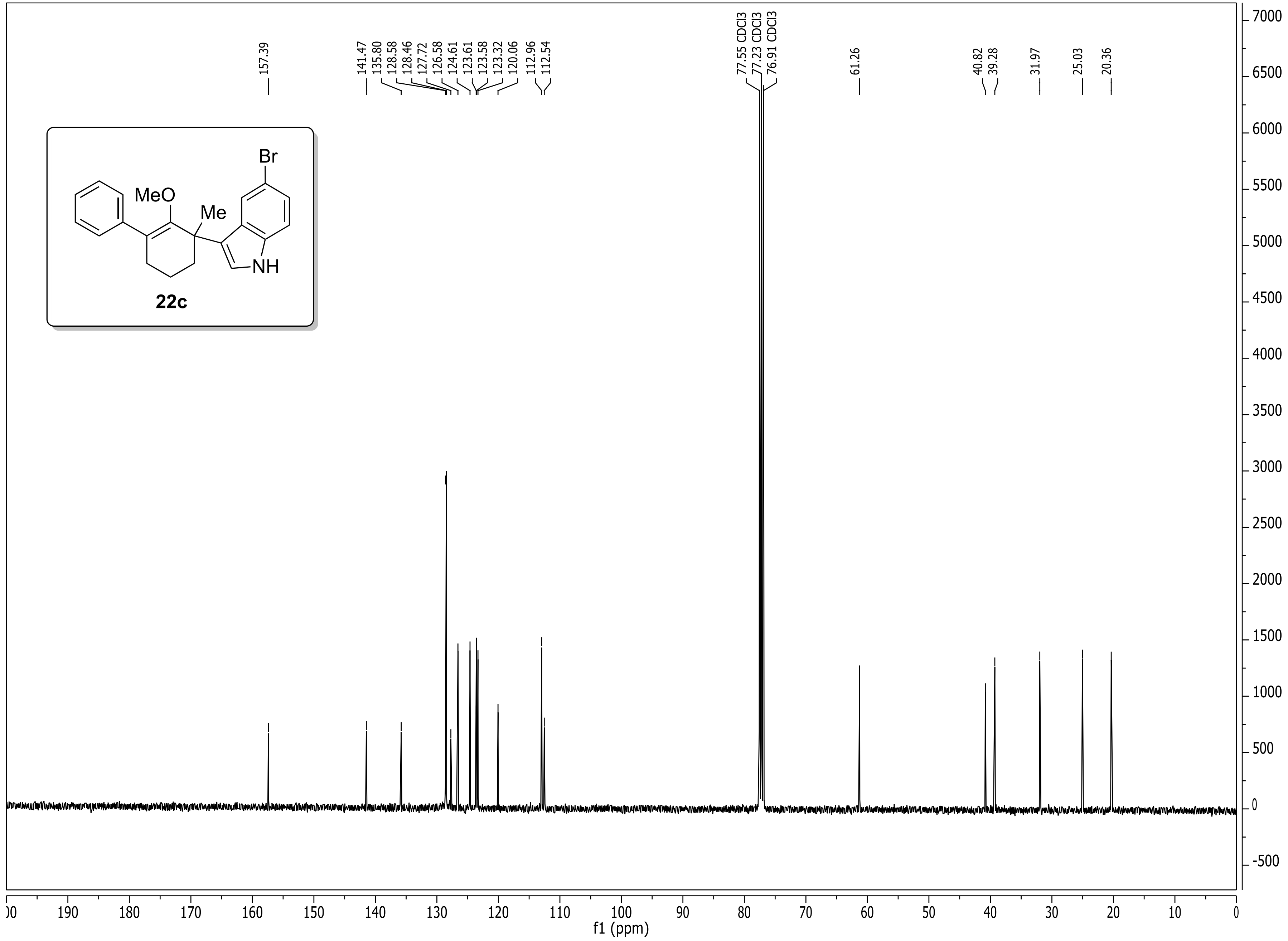
157.39
141.47
135.80
128.58
128.46
127.72
126.58
124.61
123.61
123.58
123.32
120.06
112.96
112.54
77.55 CDCl3
77.23 CDCl3
76.91 CDCl3
61.26
40.82
39.28
31.97
25.03
20.36
Br
MeO
Me
NH
22c
f1 (ppm)

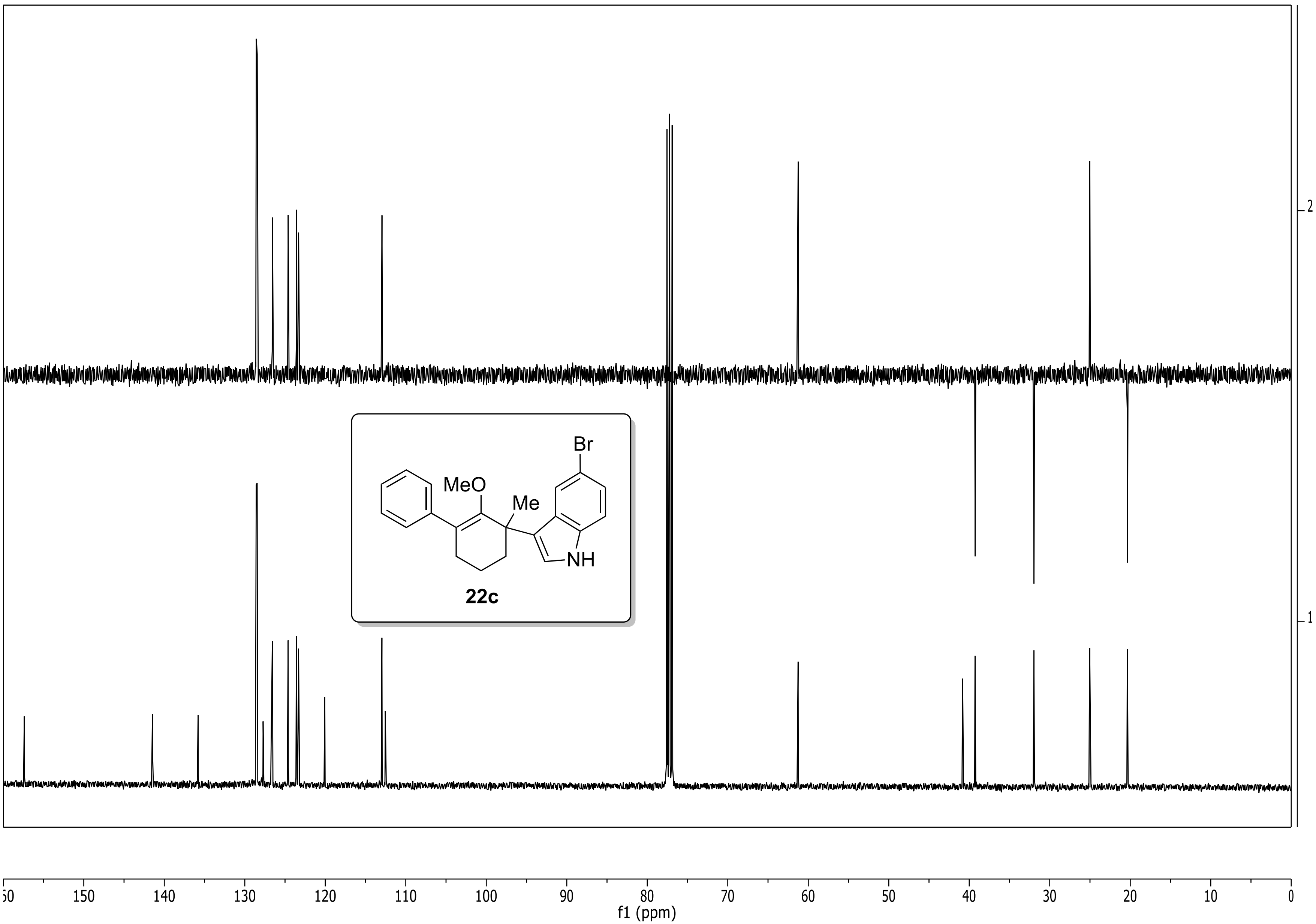
Br
MeO
Me
NH
22c
f1 (ppm)
150 140 130 120 110 100 90 80 70 60 50 40 30 20 10 0
2
1

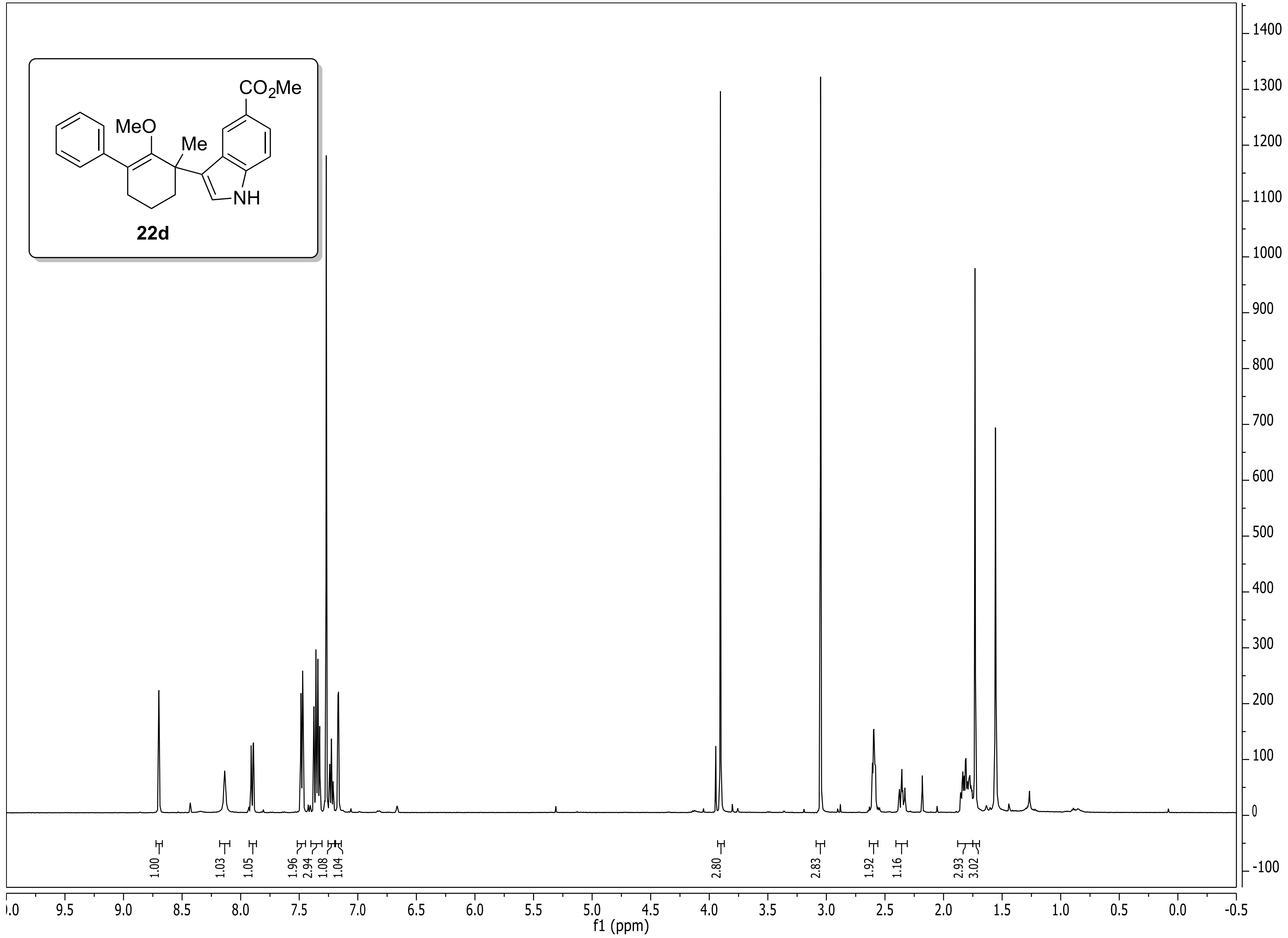
CO2Me
MeO
Me
NH
22d
1.00
1.03
1.05
1.96
2.94
1.08
1.04
2.80
2.83
1.92
1.16
2.93
3.02
f1 (ppm)

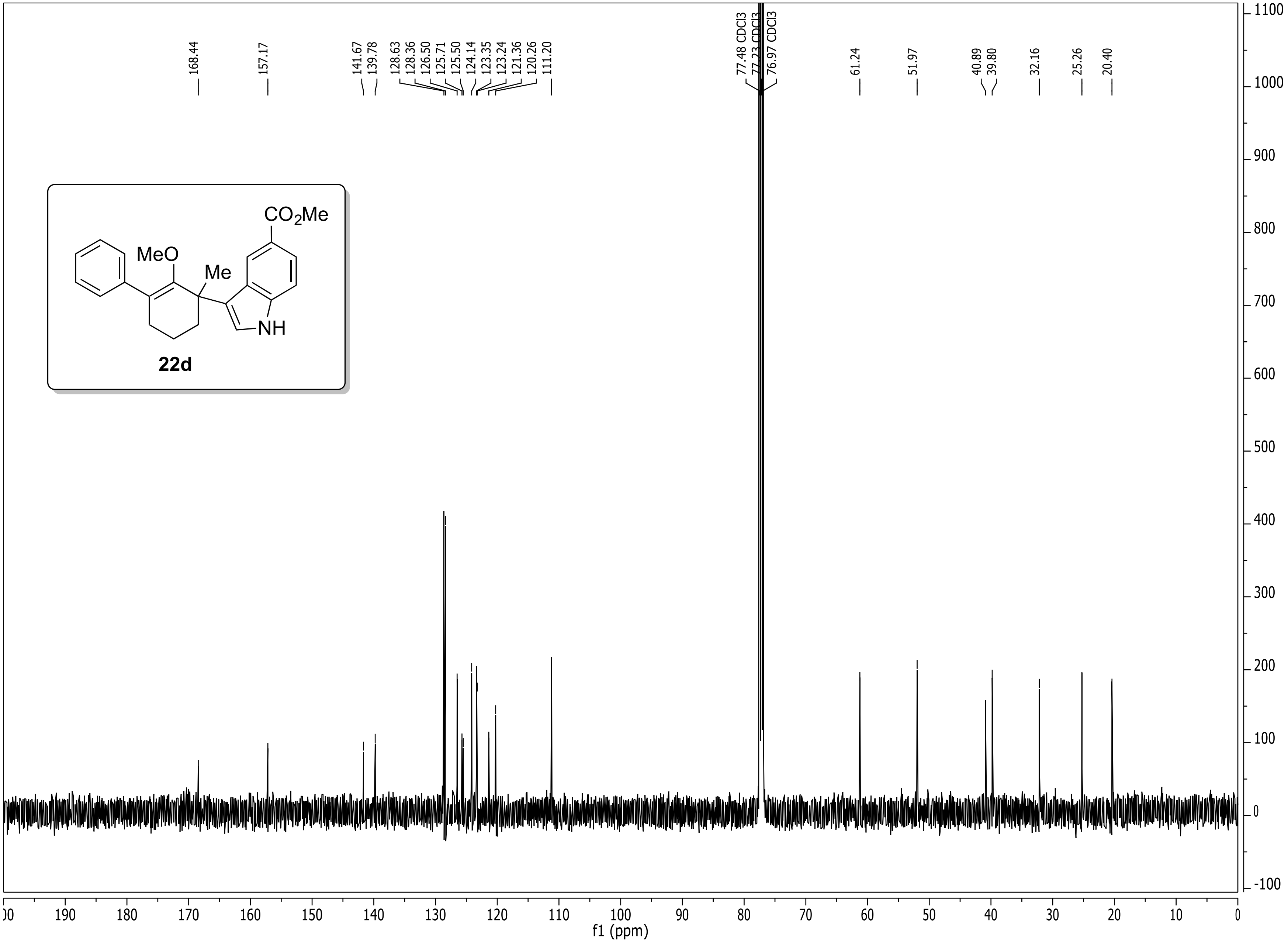
168.44
157.17
141.67
139.78
128.63
128.36
126.50
125.71
125.50
124.14
123.35
123.24
121.36
120.26
111.20
77.48 CDCl3
77.23 CDCl3
76.97 CDCl3
61.24
51.97
40.89
39.80
32.16
25.26
20.40
CO2Me
MeO
Me
NH
22d
f1 (ppm)

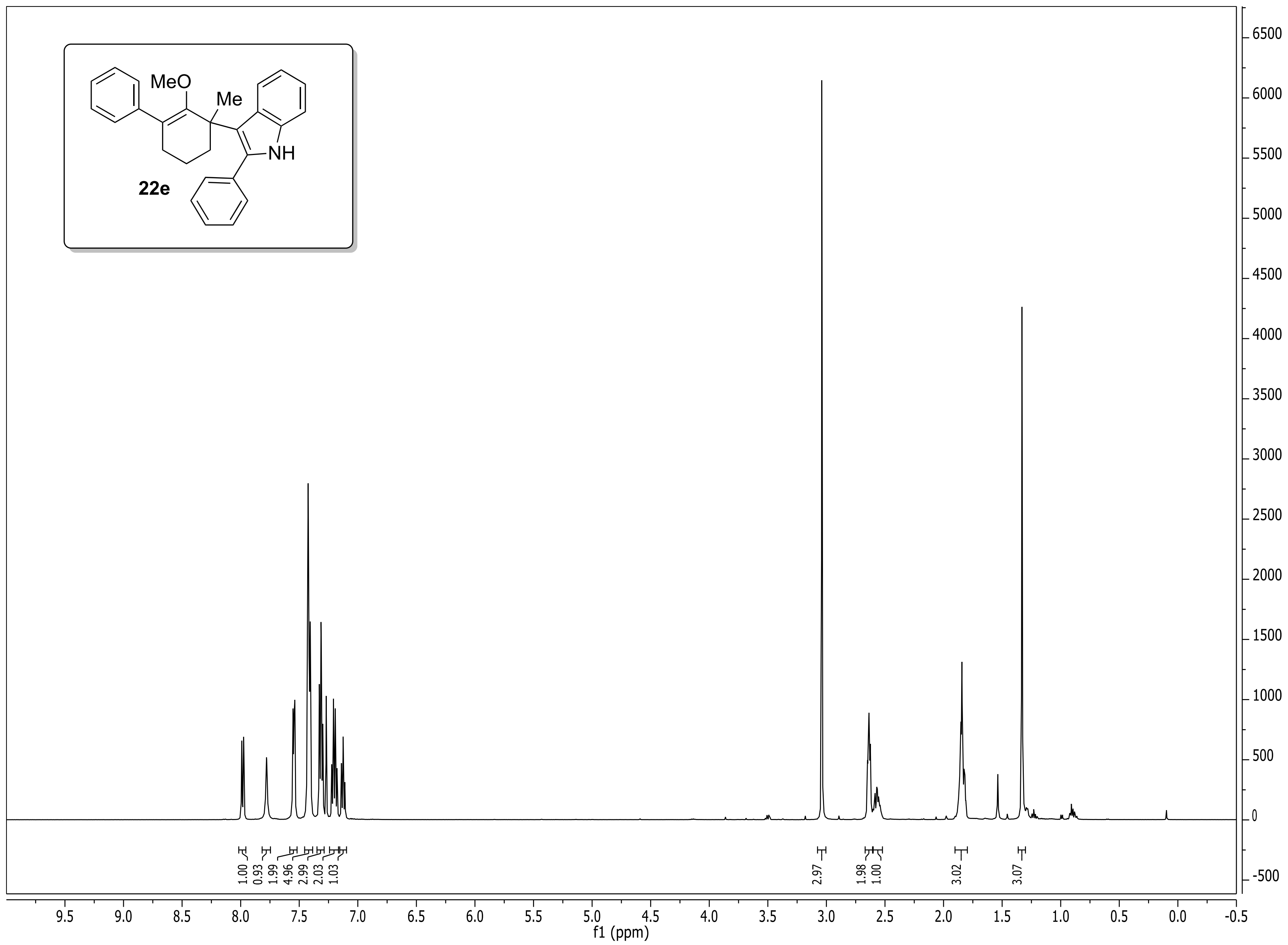
MeO
Me
NH
22e
1.00
0.93
1.99
4.96
2.99
2.03
1.03
2.97
1.98
1.00
3.02
3.07
f1 (ppm)

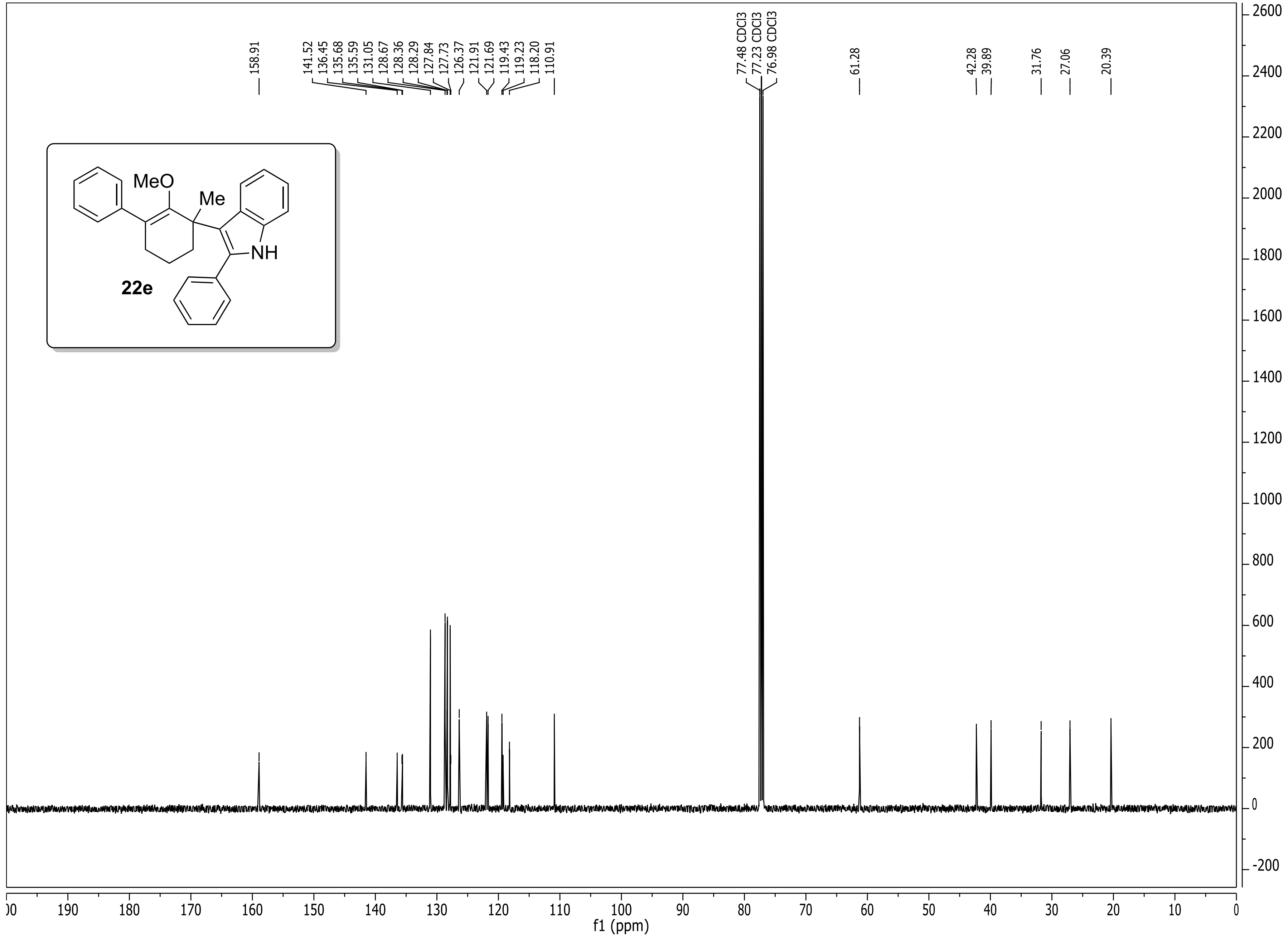
158.91
141.52
136.45
135.68
135.59
131.05
128.67
128.36
128.29
127.84
127.73
126.37
121.91
121.69
119.43
119.23
118.20
110.91
77.48 CDCl3
77.23 CDCl3
76.98 CDCl3
61.28
42.28
39.89
31.76
27.06
20.39
MeO
Me
NH
22e
f1 (ppm)

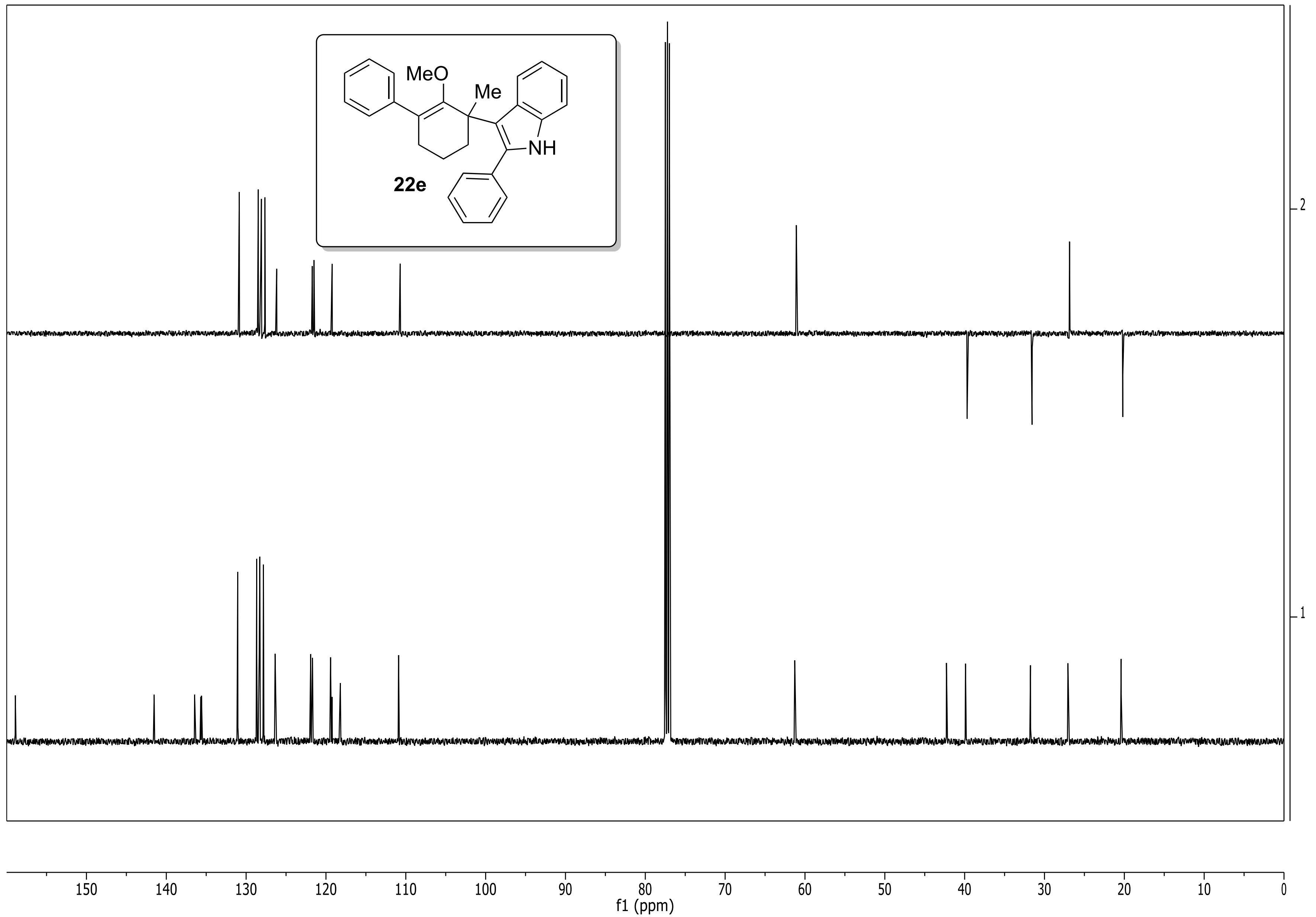
MeO
Me
NH
22e
150
140
130
120
110
100
90
80
70
60
50
40
30
20
10
0
f1 (ppm)
2
1

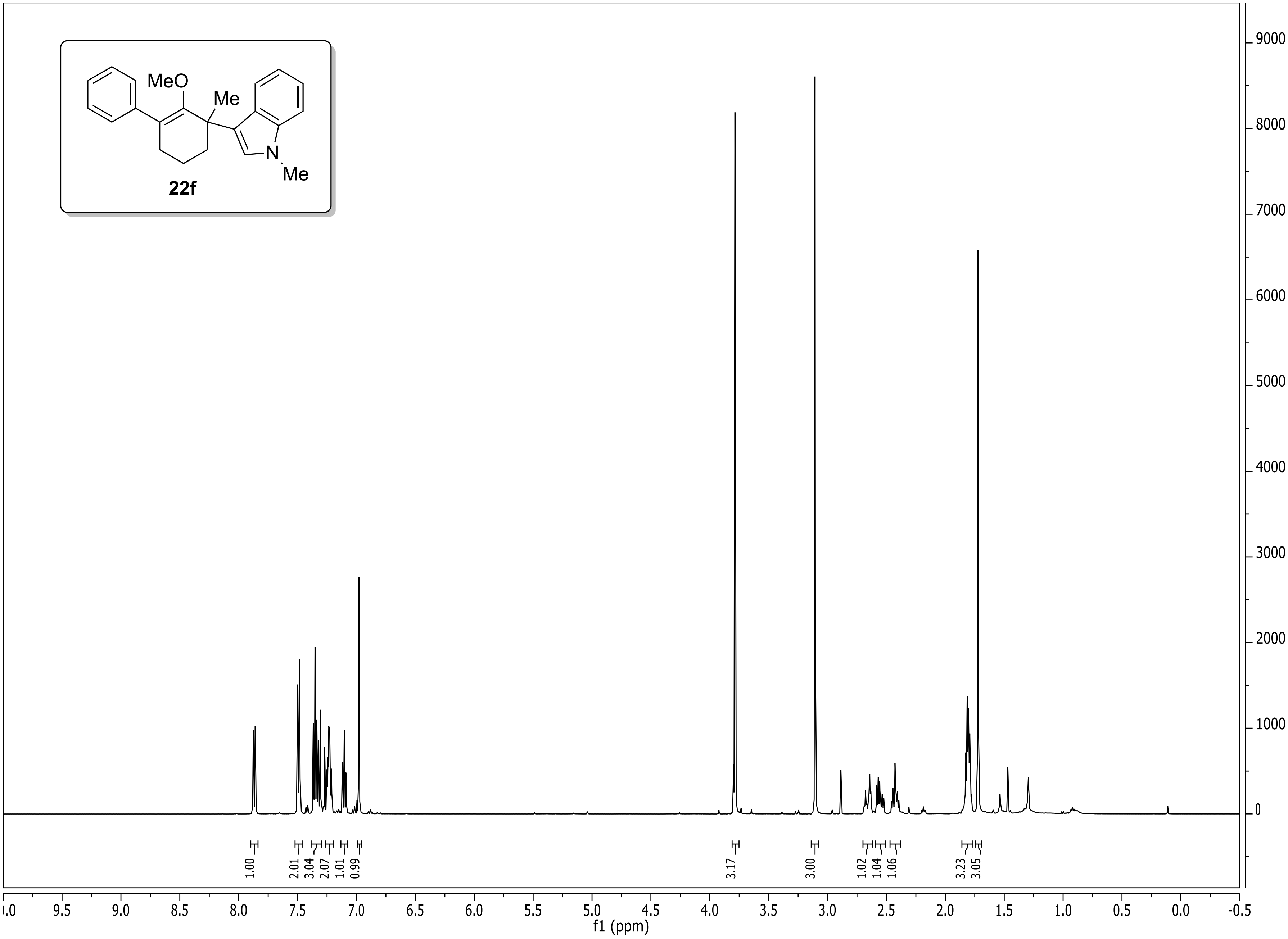
MeO
Me
N
Me
22f
1.00
2.01
3.04
2.07
1.01
0.99
3.17
3.00
1.02
1.04
1.06
3.23
3.05
f1 (ppm)

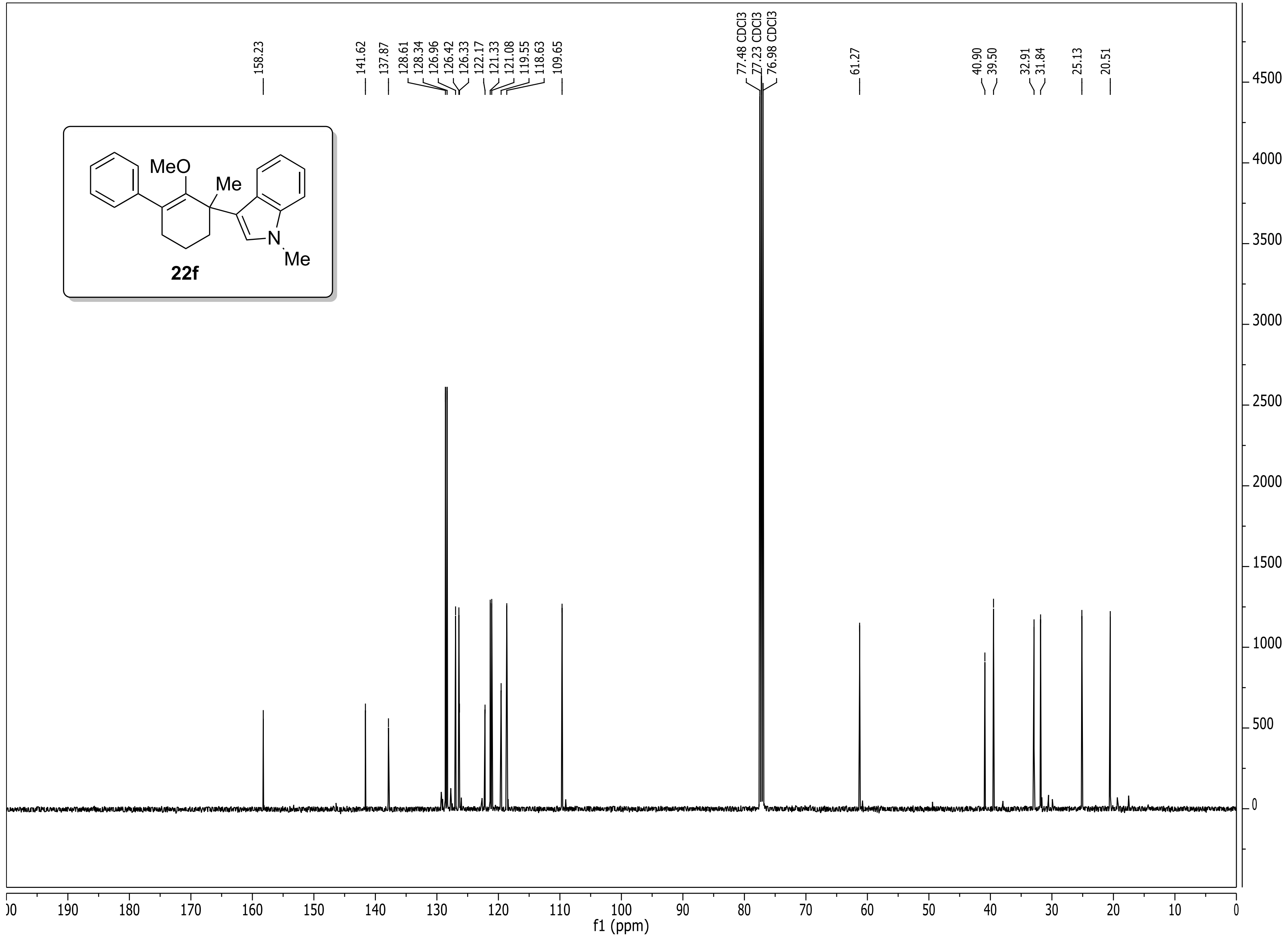

158.23
141.62
137.87
128.61
128.34
126.96
126.42
126.33
122.17
121.33
121.08
119.55
118.63
109.65
77.48 CDCl3
77.23 CDCl3
76.98 CDCl3
61.27
40.90
39.50
32.91
31.84
25.13
20.51
MeO
Me
N
Me
22f
f1 (ppm)

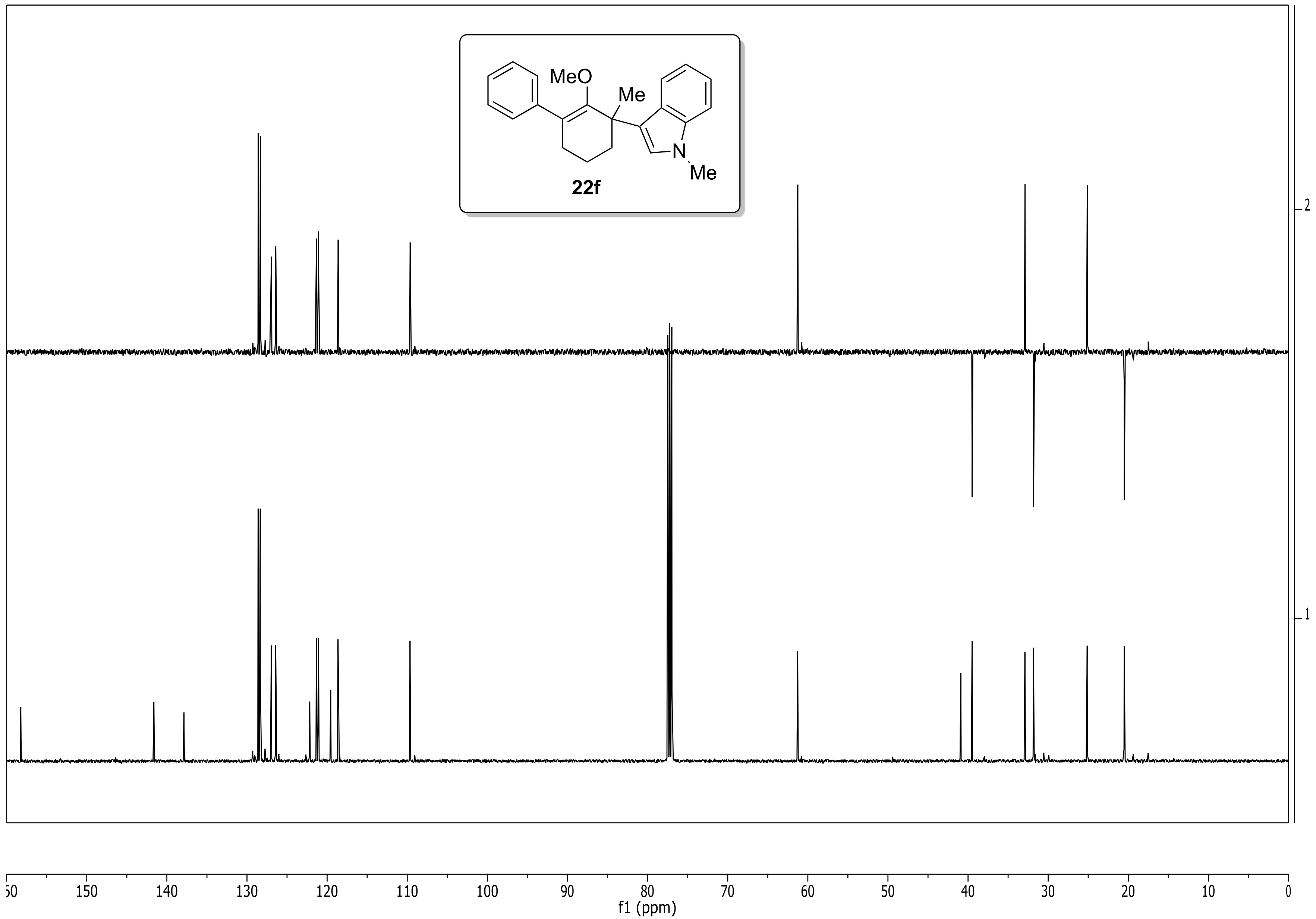
MeO
Me
N
Me
22f
2
1
f1 (ppm)
150
140
130
120
110
100
90
80
70
60
50
40
30
20
10
0

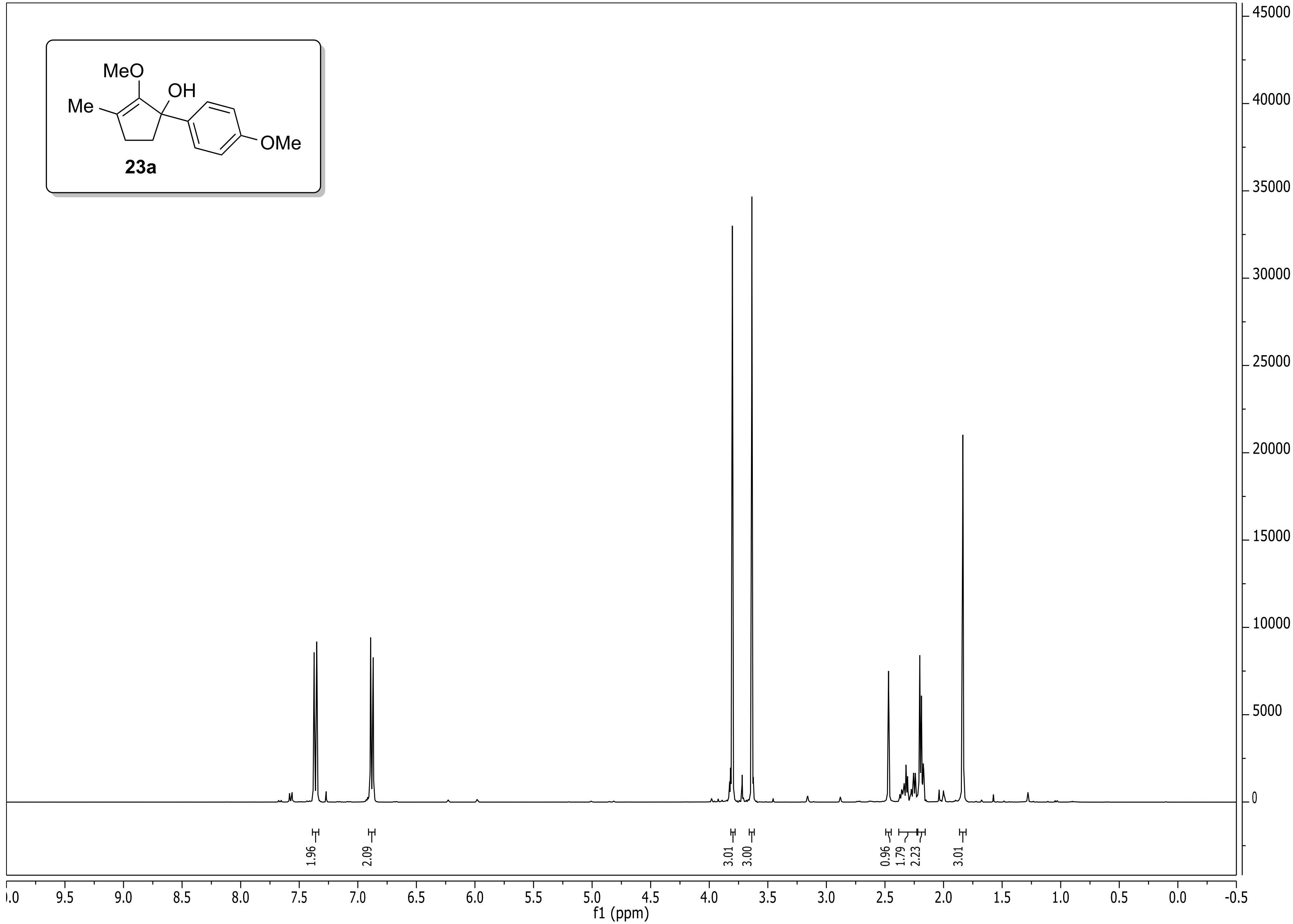
MeO
OH
Me
OMe
23a
1.96
2.09
3.01
3.00
0.96
1.79
2.23
3.01
f1 (ppm)

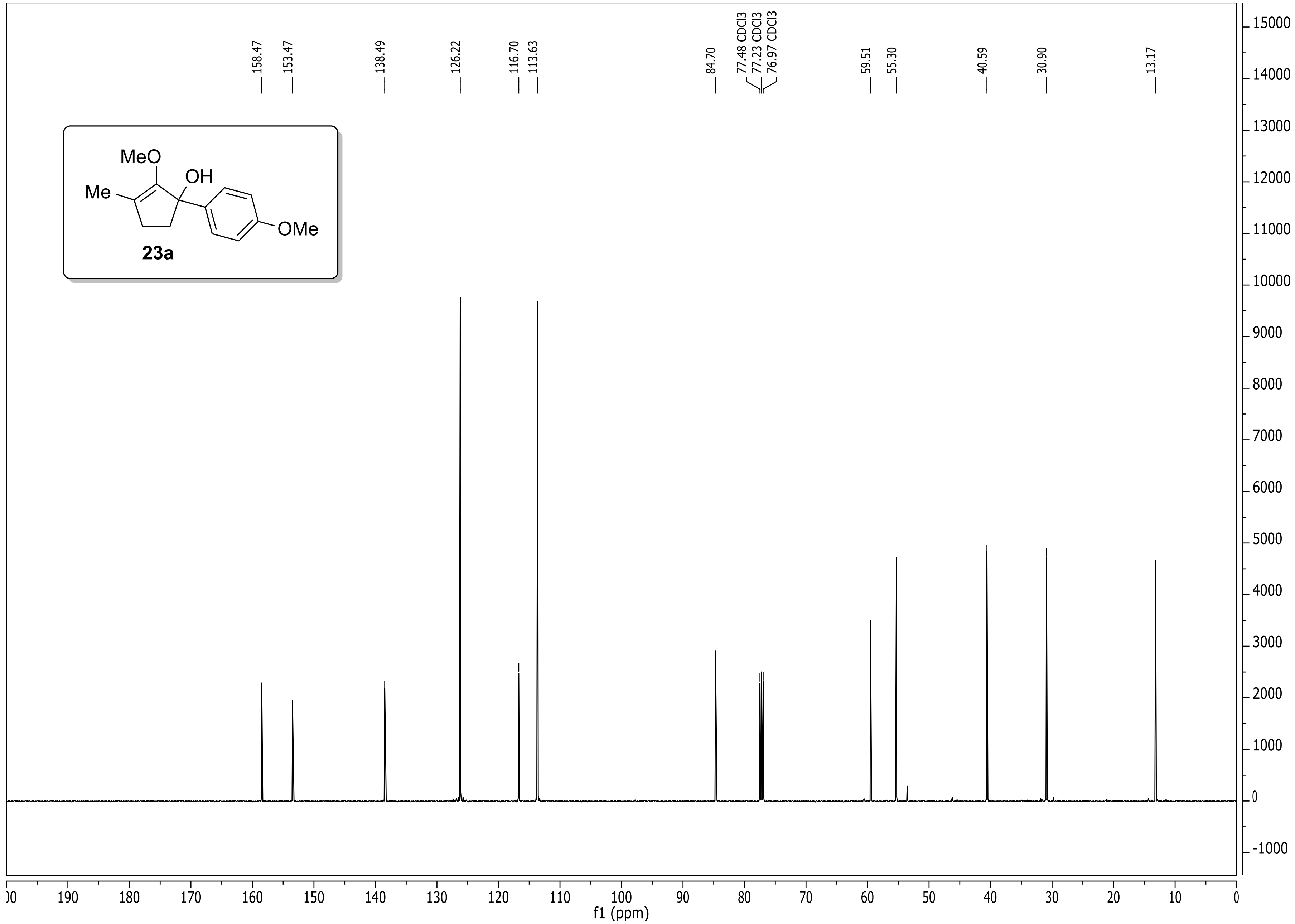

158.47
153.47
138.49
126.22
116.70
113.63
84.70
77.48 CDCl3
77.23 CDCl3
76.97 CDCl3
59.51
55.30
40.59
30.90
13.17
MeO
OH
Me
OMe
23a
f1 (ppm)

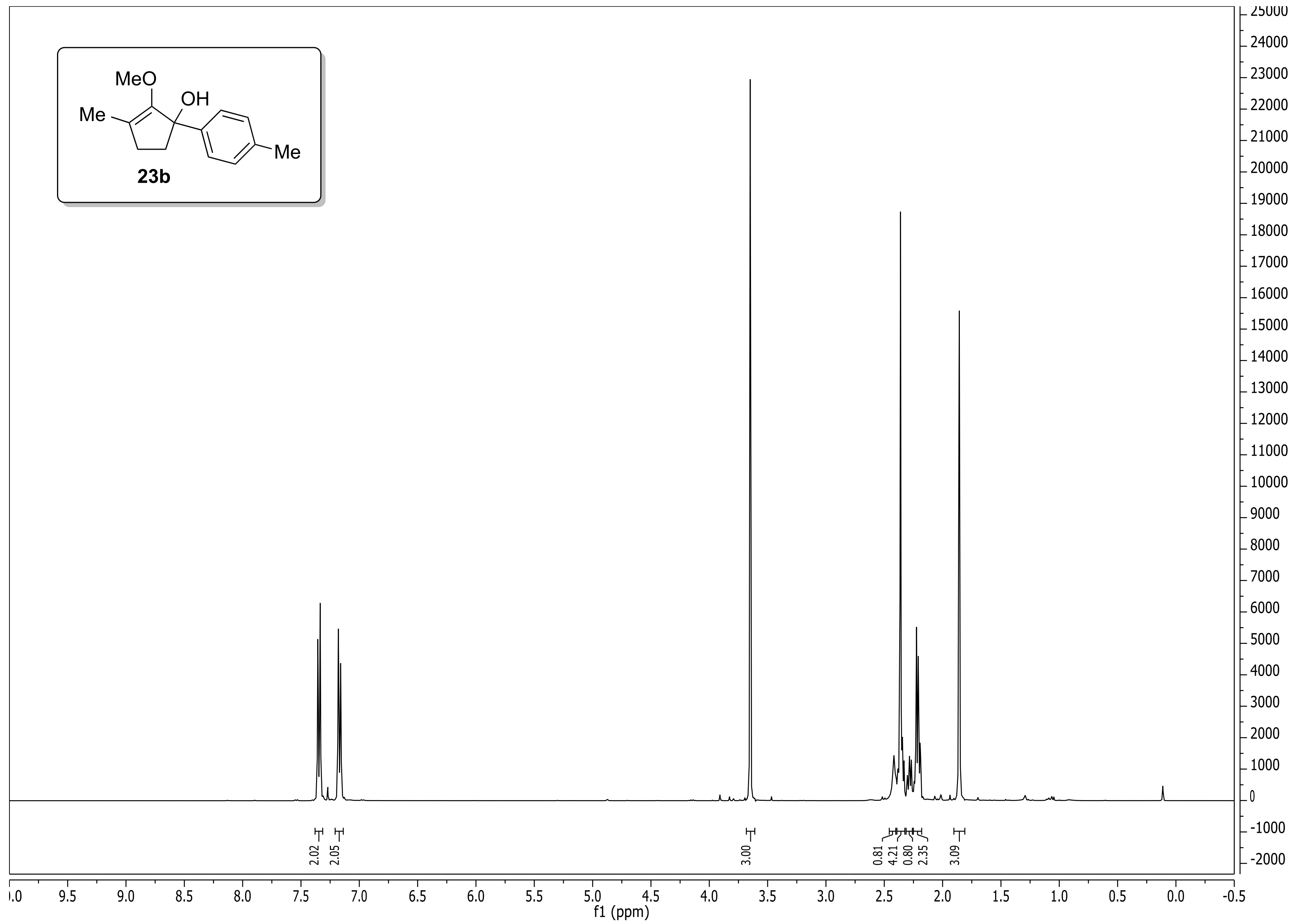
MeO
OH
Me
Me
23b
2.02
2.05
3.00
0.81
4.21
0.80
2.35
3.09
f1 (ppm)

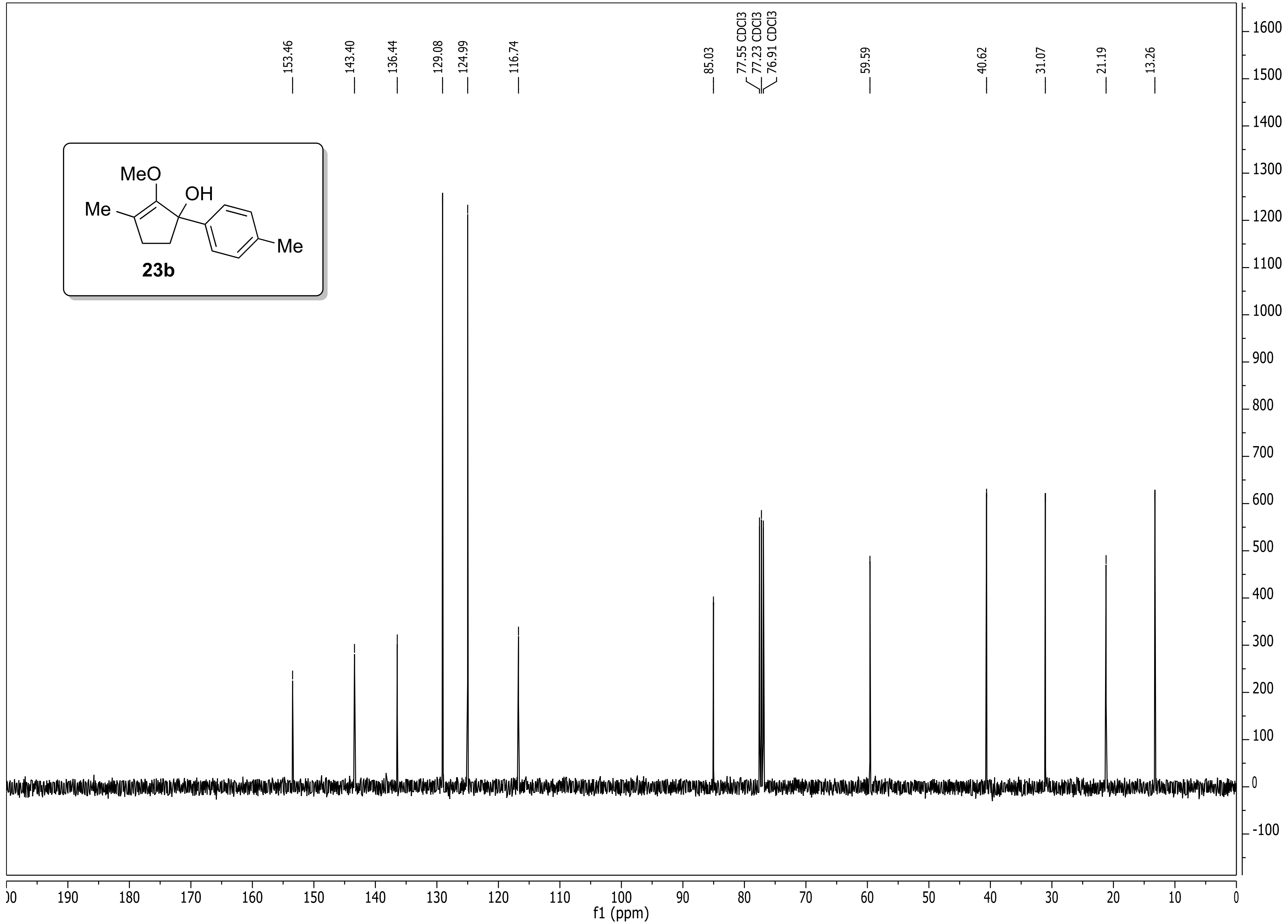
153.46
143.40
136.44
129.08
124.99
116.74
85.03
77.55 CDCl3
77.23 CDCl3
76.91 CDCl3
59.59
40.62
31.07
21.19
13.26
MeO
OH
Me
Me
23b
f1 (ppm)

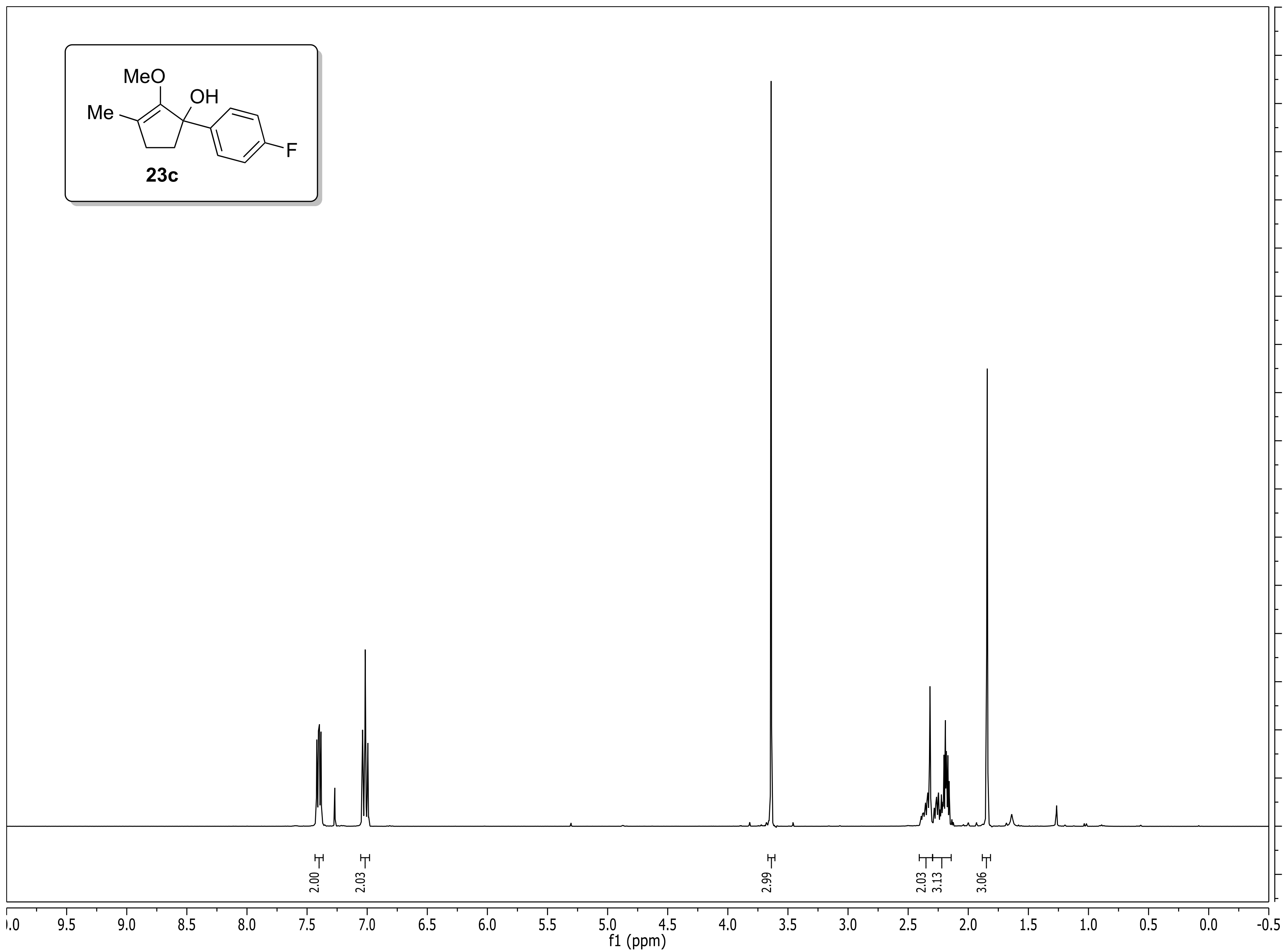
MeO
OH
Me
F
23c
32000
30000
28000
26000
24000
22000
20000
18000
16000
14000
12000
10000
8000
6000
4000
2000
0
-2000
2.00
2.03
2.99
2.03
3.13
3.06
9.5
9.0
8.5
8.0
7.5
7.0
6.5
6.0
5.5
5.0
4.5
4.0
3.5
3.0
2.5
2.0
1.5
1.0
0.5
0.0
-0.5
f1 (ppm)

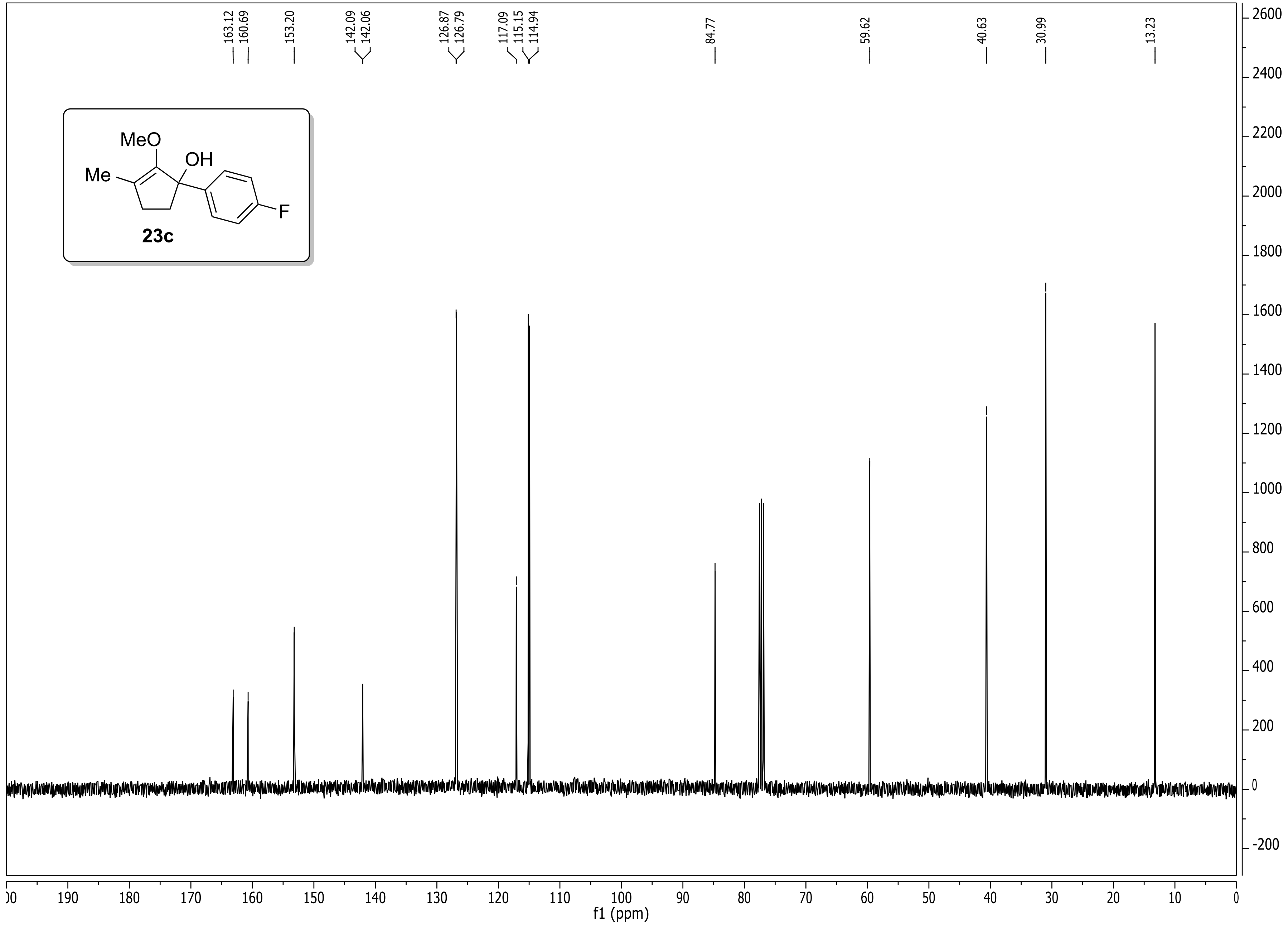

163.12
160.69
153.20
142.09
142.06
126.87
126.79
117.09
115.15
114.94
84.77
59.62
40.63
30.99
13.23
MeO
OH
Me
F
23c
f1 (ppm)

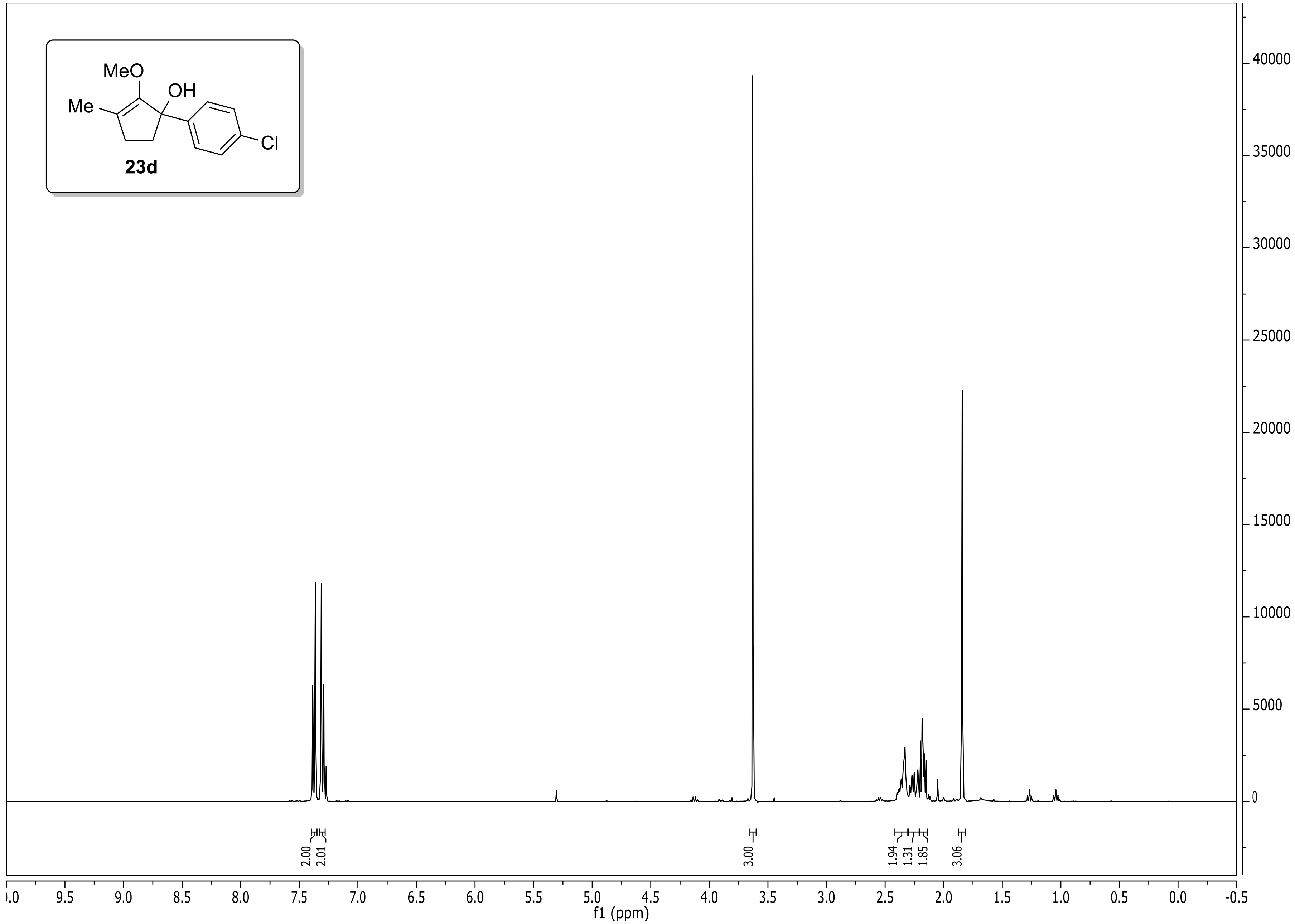
MeO
OH
Me
Cl
23d
2.00
2.01
3.00
1.94
1.31
1.85
3.06
f1 (ppm)

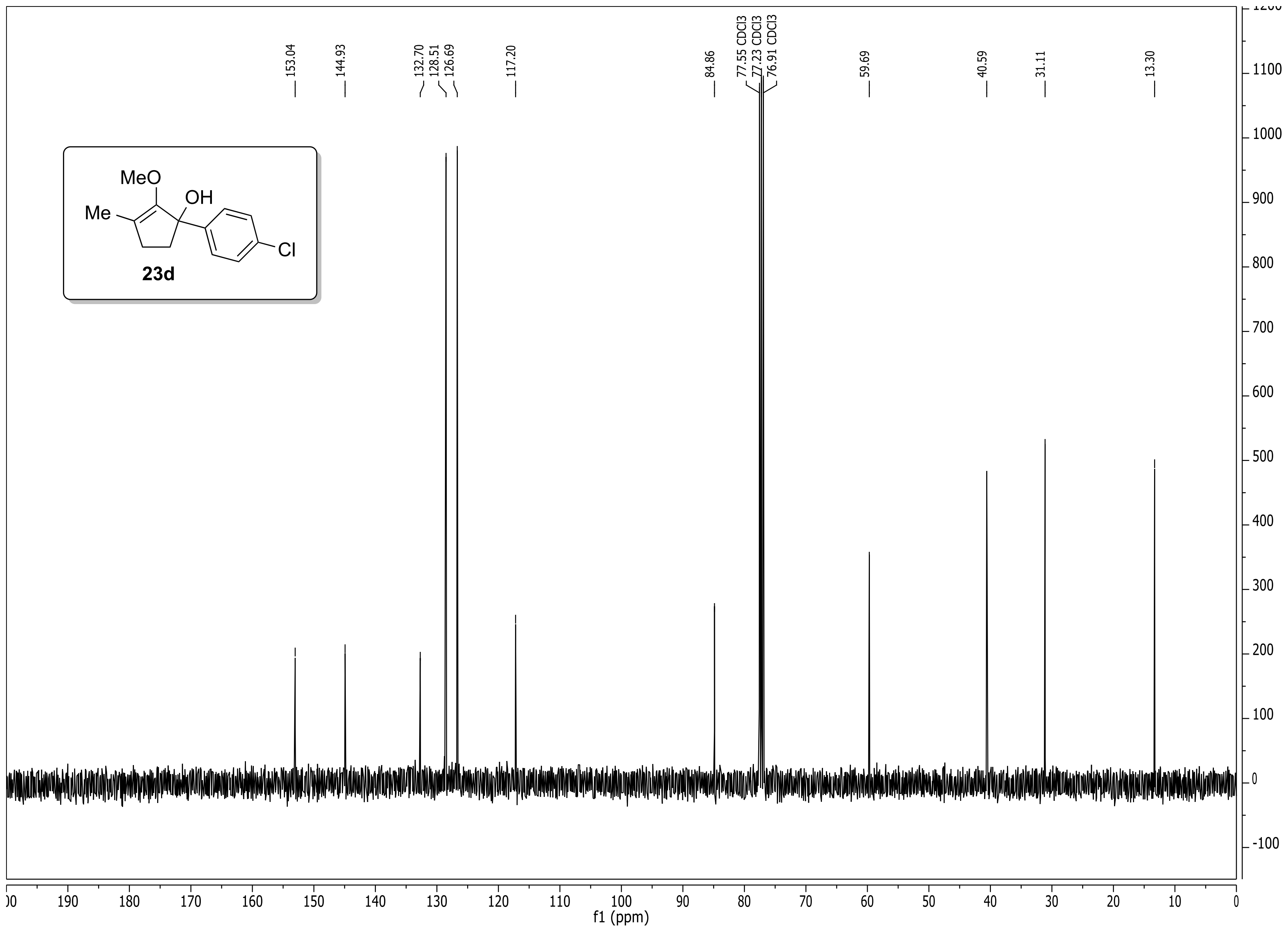
153.04
144.93
132.70
128.51
126.69
117.20
84.86
77.55 CDCl3
77.23 CDCl3
76.91 CDCl3
59.69
40.59
31.11
13.30
MeO
OH
Me
Cl
23d
f1 (ppm)

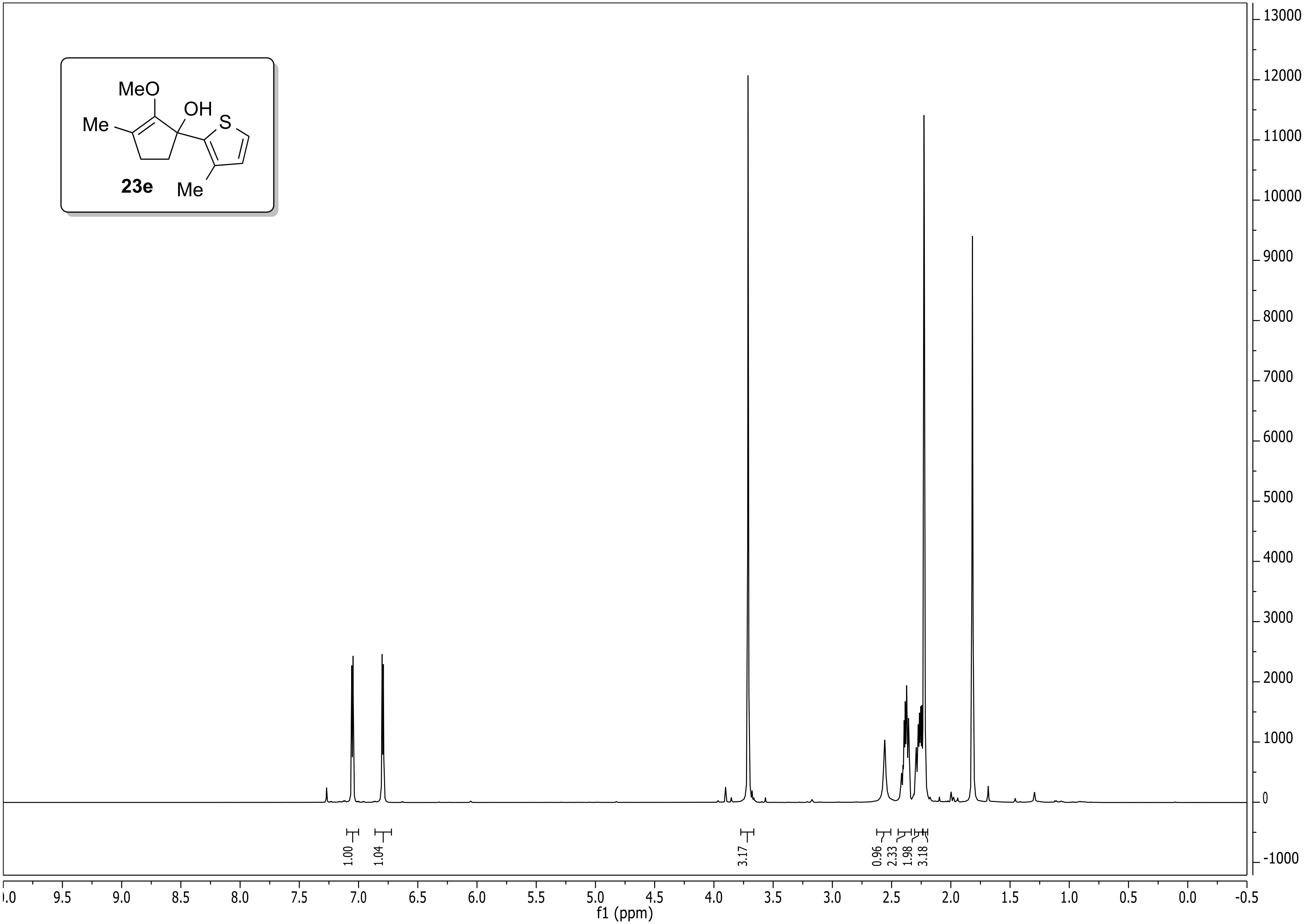

MeO
OH
S
Me
23e
Me
13000
12000
11000
10000
9000
8000
7000
6000
5000
4000
3000
2000
1000
0
-1000
1.00
1.04
3.17
0.96
2.33
1.98
3.18
9.5
9.0
8.5
8.0
7.5
7.0
6.5
6.0
5.5
5.0
4.5
4.0
3.5
3.0
2.5
2.0
1.5
1.0
0.5
0.0
-0.5
f1 (ppm)

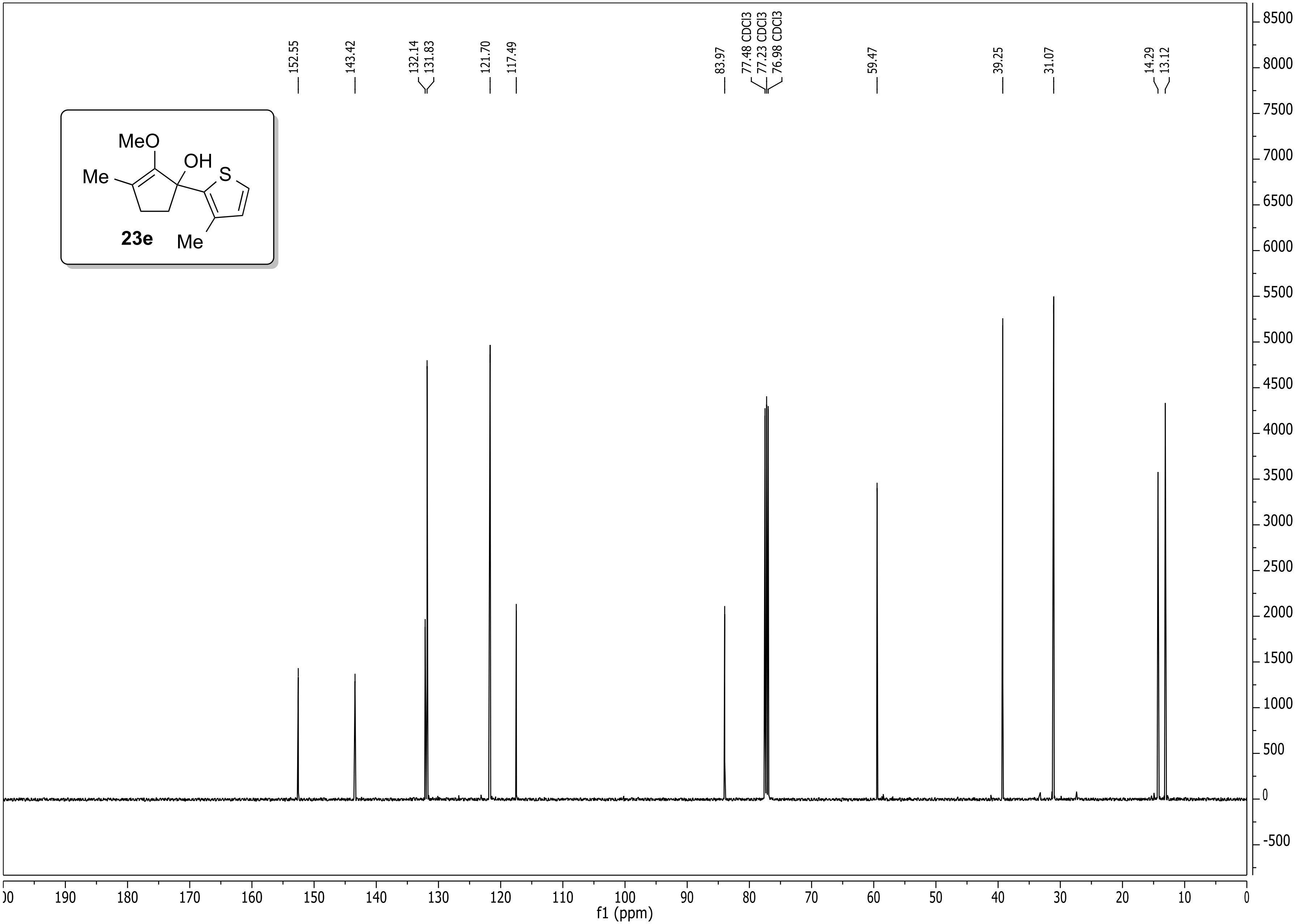
152.55
143.42
132.14
131.83
121.70
117.49
83.97
77.48 CDCl3
77.23 CDCl3
76.98 CDCl3
59.47
39.25
31.07
14.29
13.12
MeO
OH
Me
S
23e
Me
f1 (ppm)

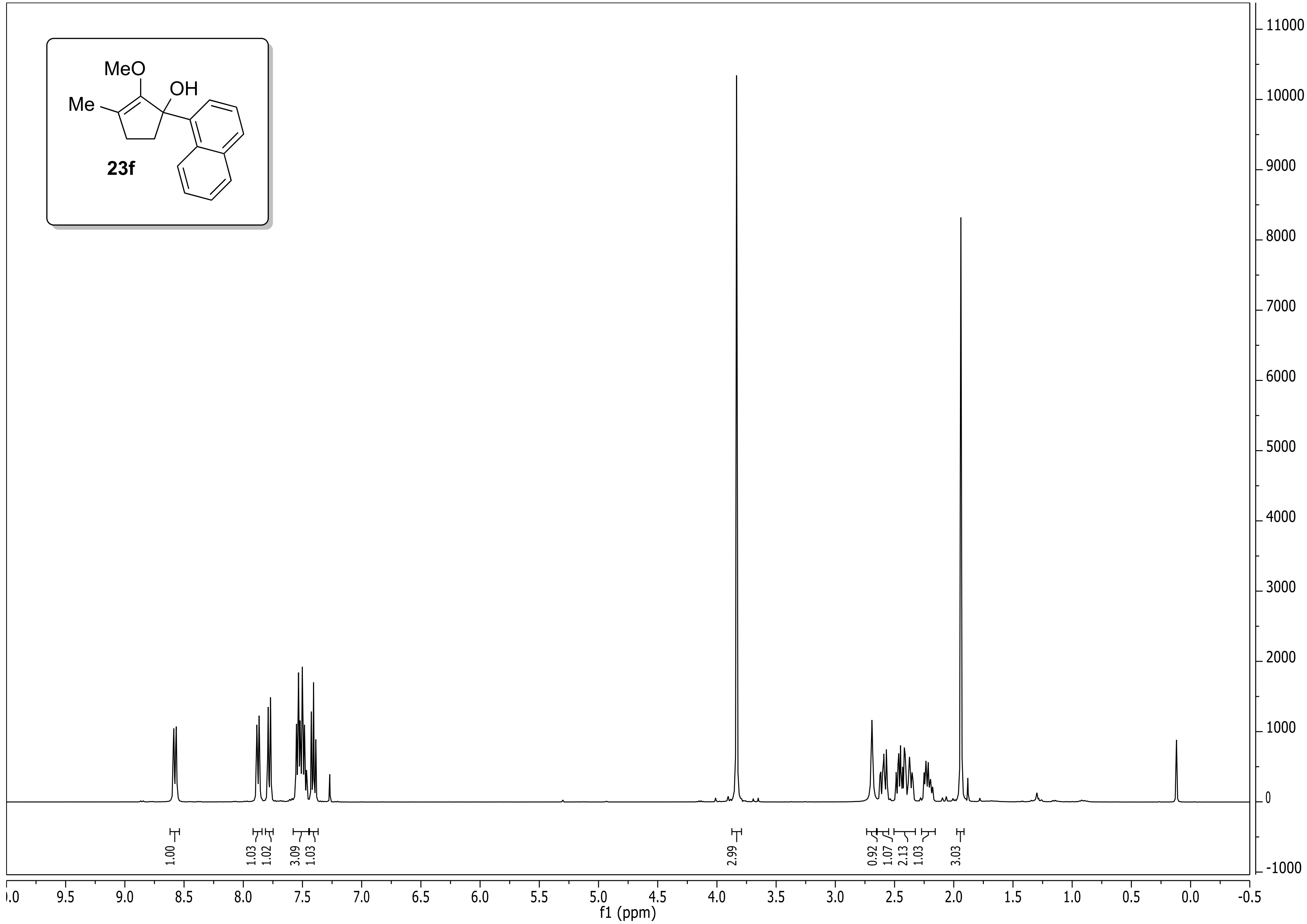
MeO
OH
Me
23f
1.00
1.03
1.02
3.09
1.03
2.99
0.92
1.07
2.13
1.03
3.03
f1 (ppm)

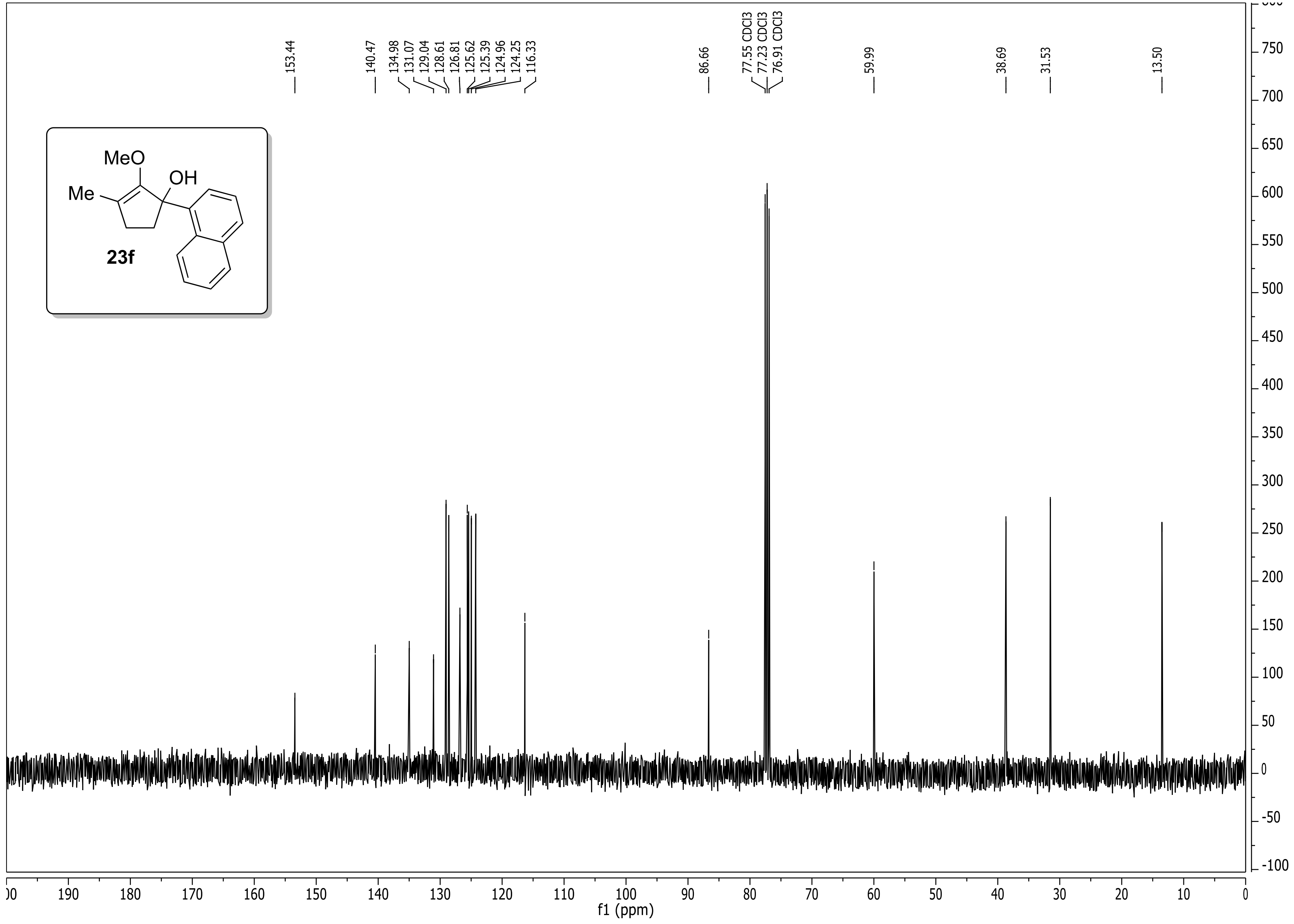

153.44
140.47
134.98
131.07
129.04
128.61
126.81
125.62
125.39
124.96
124.25
116.33
86.66
77.55 CDCl3
77.23 CDCl3
76.91 CDCl3
59.99
38.69
31.53
13.50
MeO
OH
Me
23f
f1 (ppm)

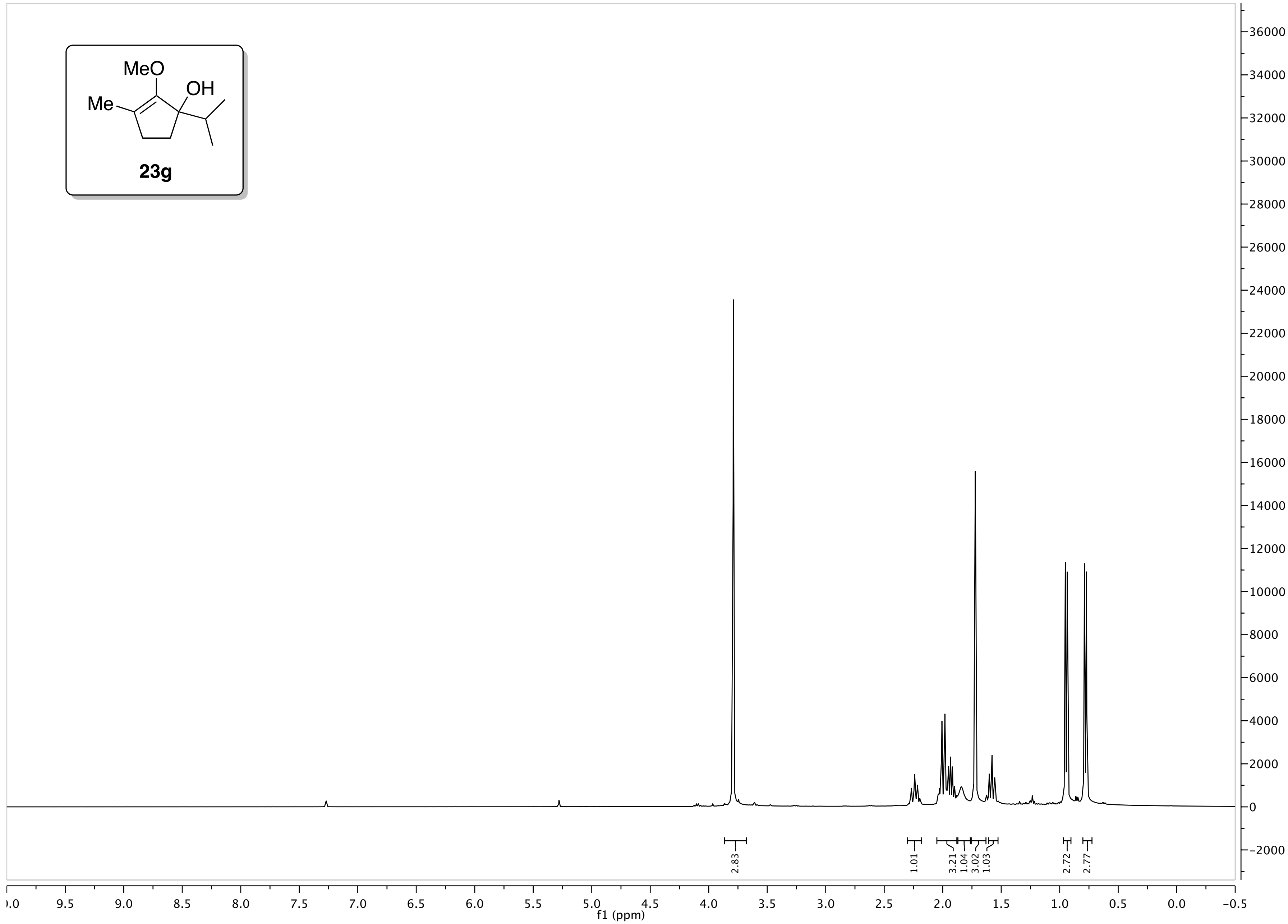
MeO
OH
Me
23g
2.83
1.01
3.21
1.04
3.02
1.03
2.72
2.77
f1 (ppm)

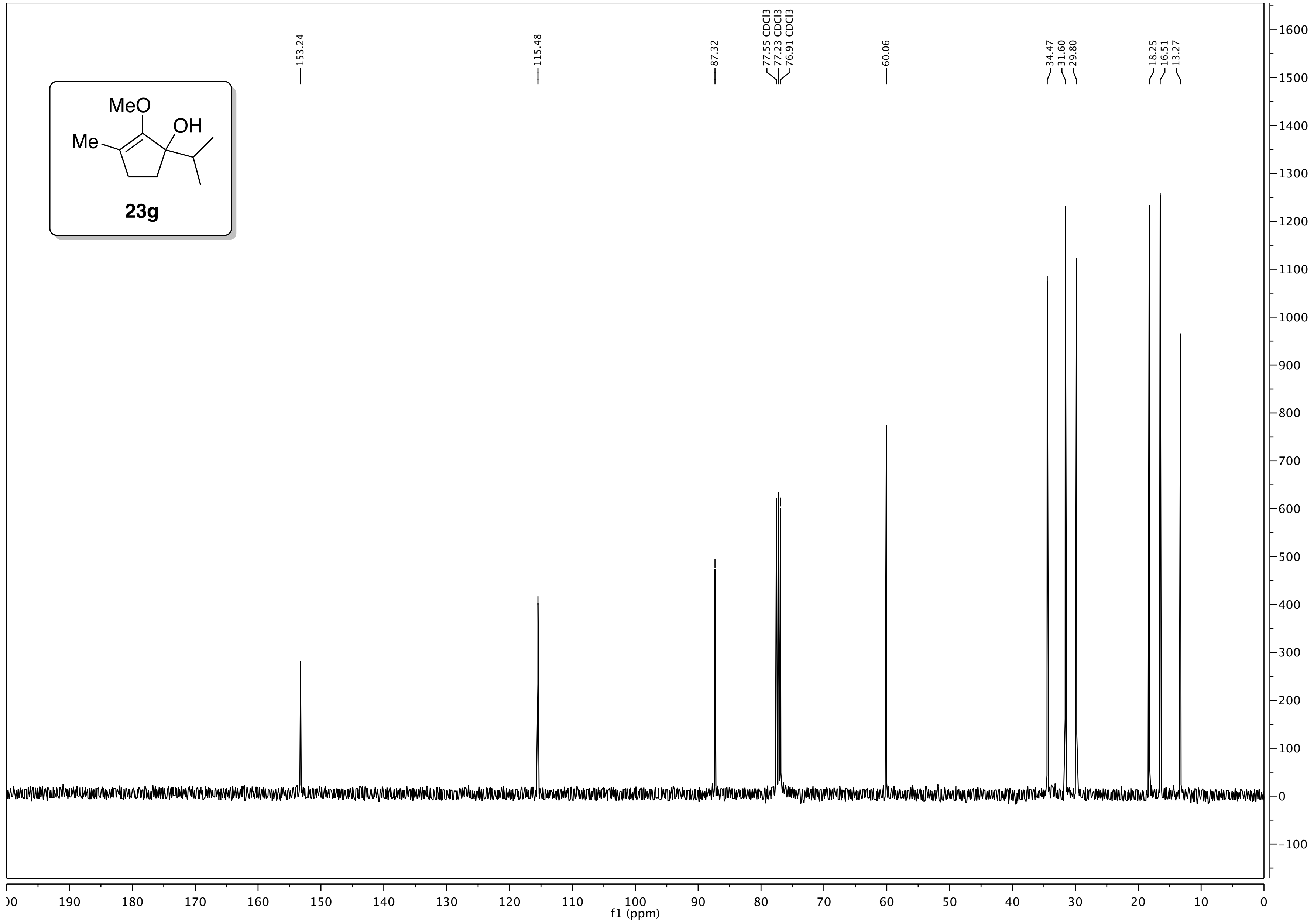
MeO
OH
Me
23g
153.24
115.48
87.32
77.55 CDCl3
77.23 CDCl3
76.91 CDCl3
60.06
34.47
31.60
29.80
18.25
16.51
13.27
f1 (ppm)

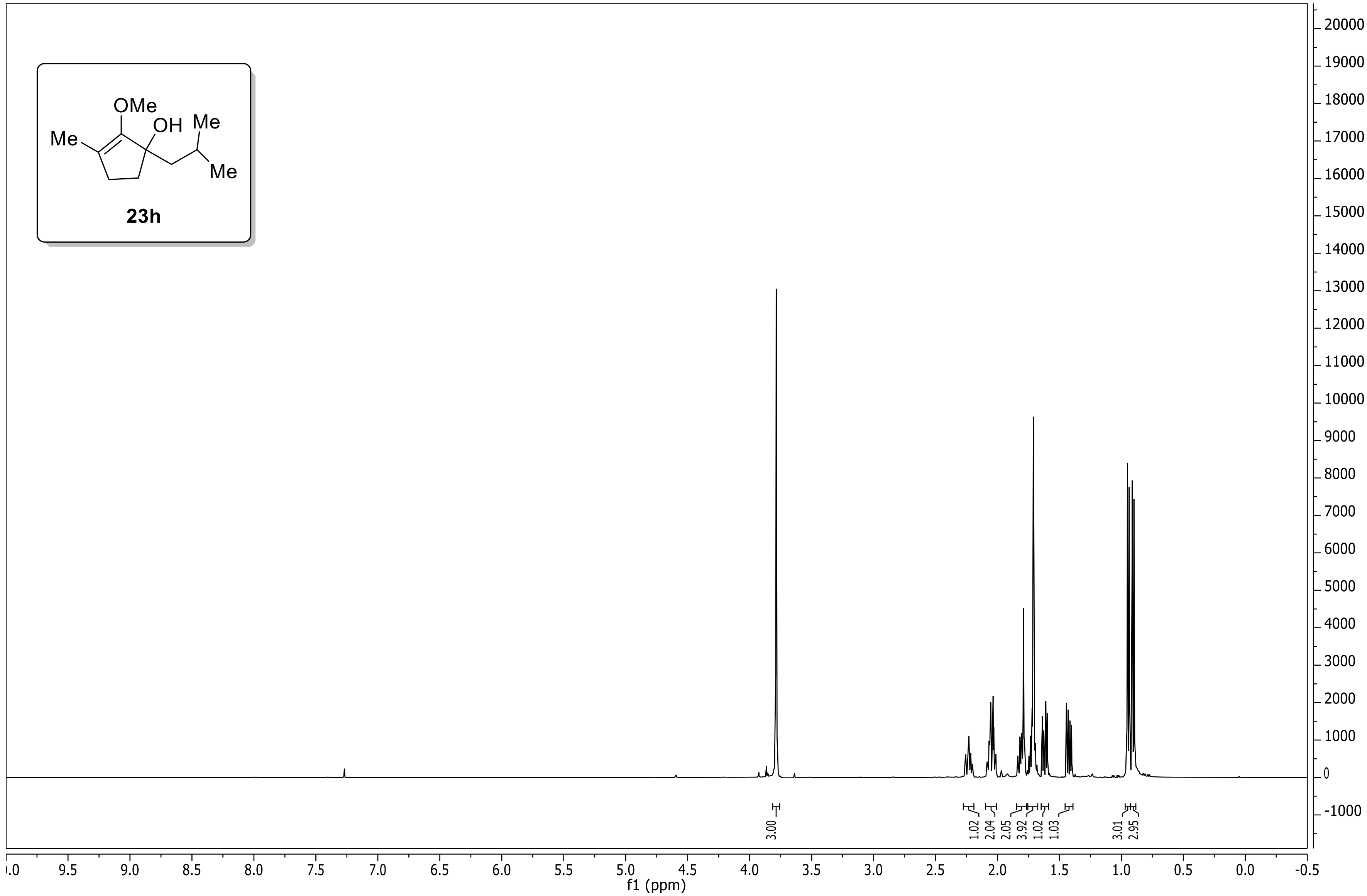

OMe
OH
Me
Me
Me
23h
f1 (ppm)

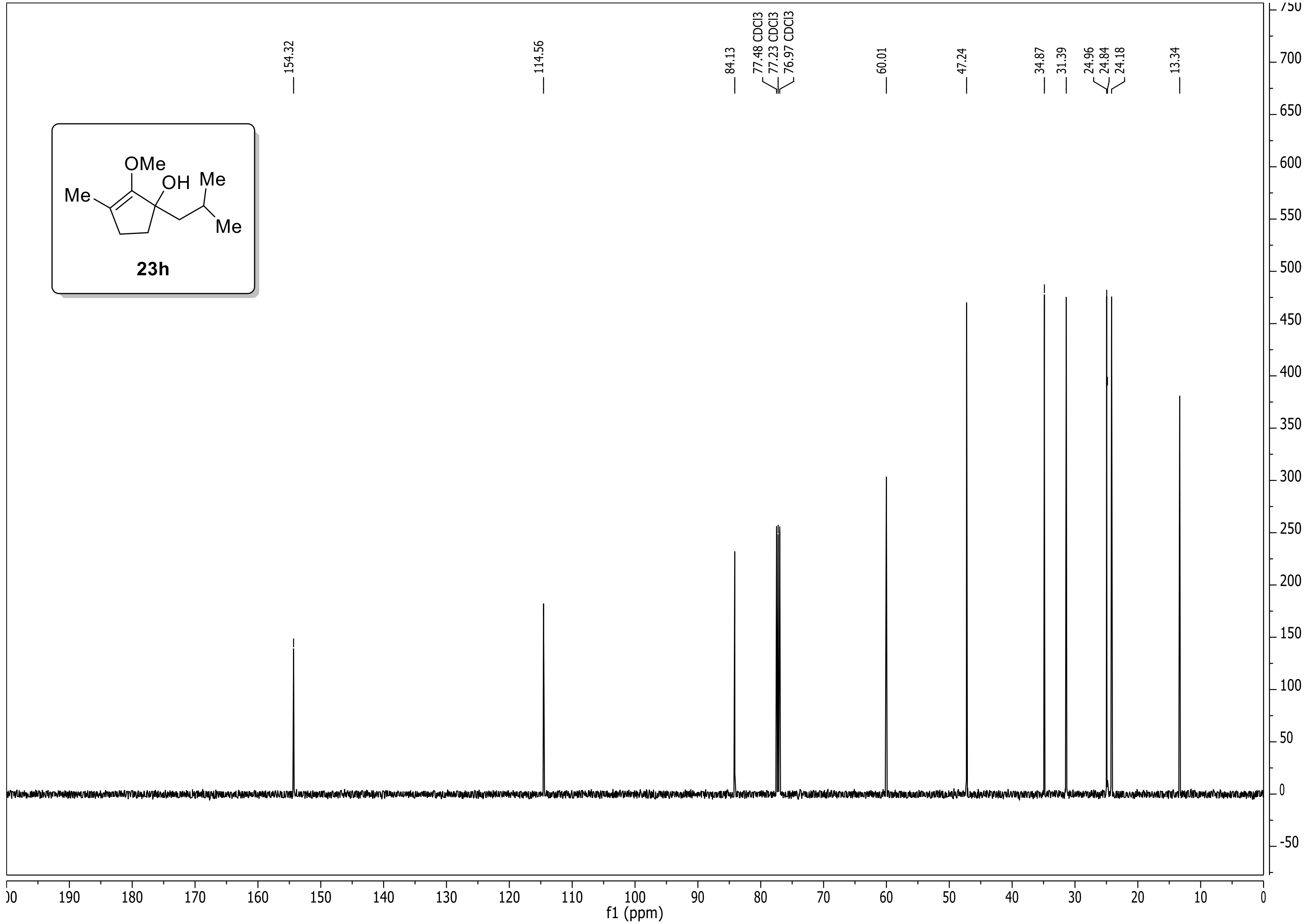
154.32
114.56
84.13
77.48 CDCl3
77.23 CDCl3
76.97 CDCl3
60.01
47.24
34.87
31.39
24.96
24.84
24.18
13.34
OMe
OH
Me
Me
Me
23h
f1 (ppm)

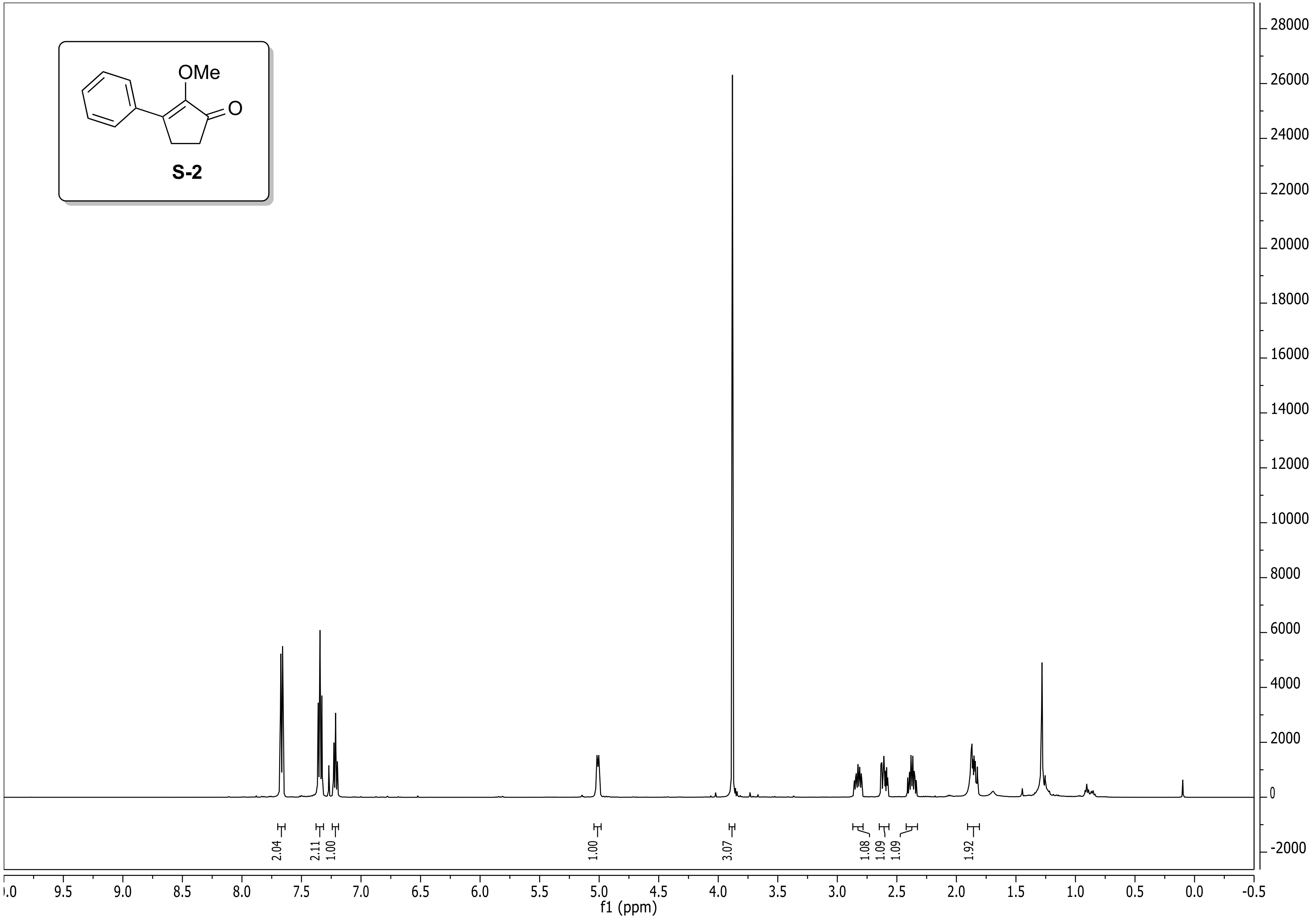
OMe
O
S-2
28000
26000
24000
22000
20000
18000
16000
14000
12000
10000
8000
6000
4000
2000
0
-2000
2.04
2.11
1.00
1.00
3.07
1.08
1.09
1.09
1.92
9.5
9.0
8.5
8.0
7.5
7.0
6.5
6.0
5.5
5.0
4.5
4.0
3.5
3.0
2.5
2.0
1.5
1.0
0.5
0.0
-0.5
f1 (ppm)

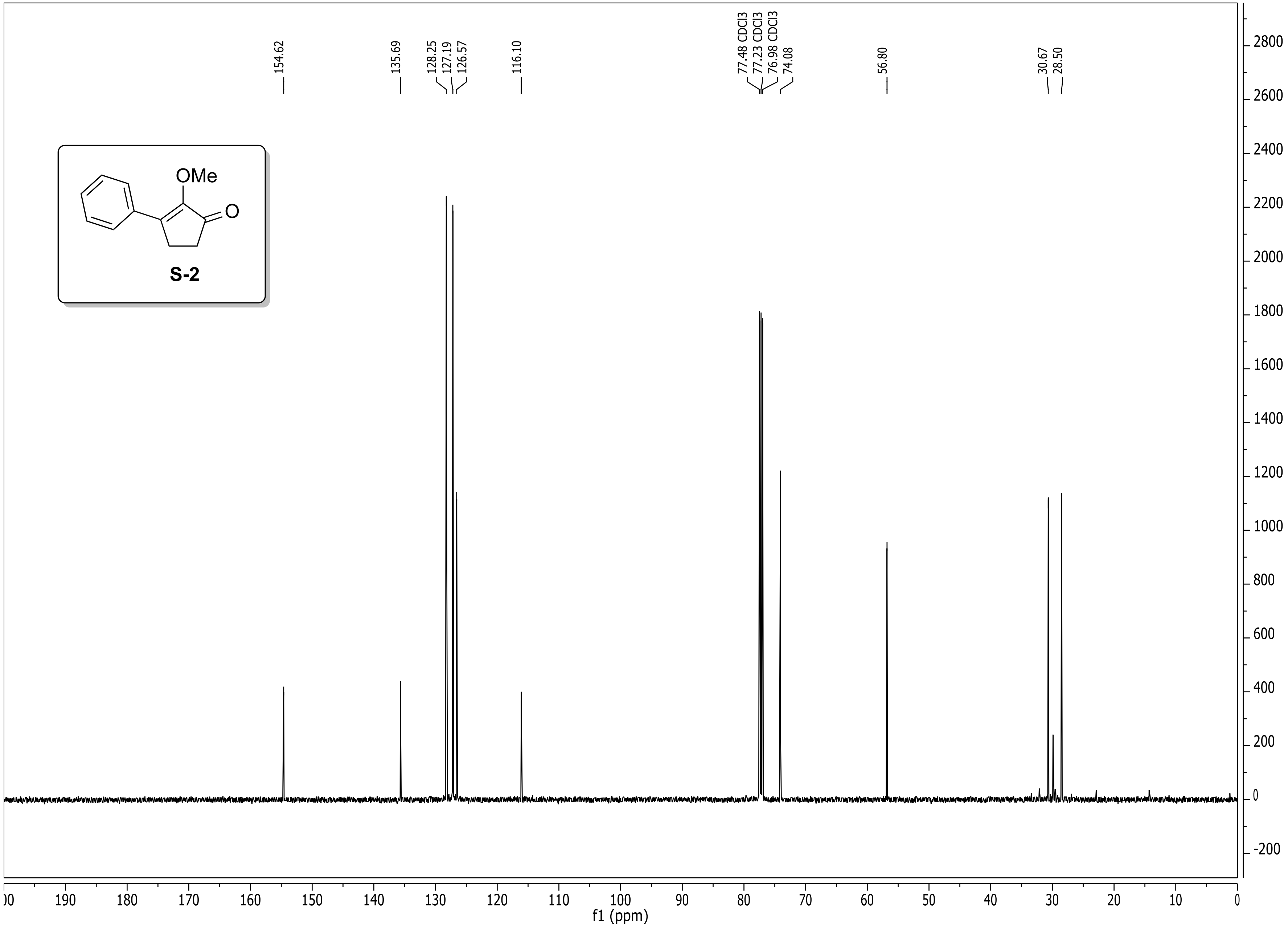
154.62
135.69
128.25
127.19
126.57
116.10
77.48 CDCl3
77.23 CDCl3
76.98 CDCl3
74.08
56.80
30.67
28.50
OMe
O
S-2
f1 (ppm)

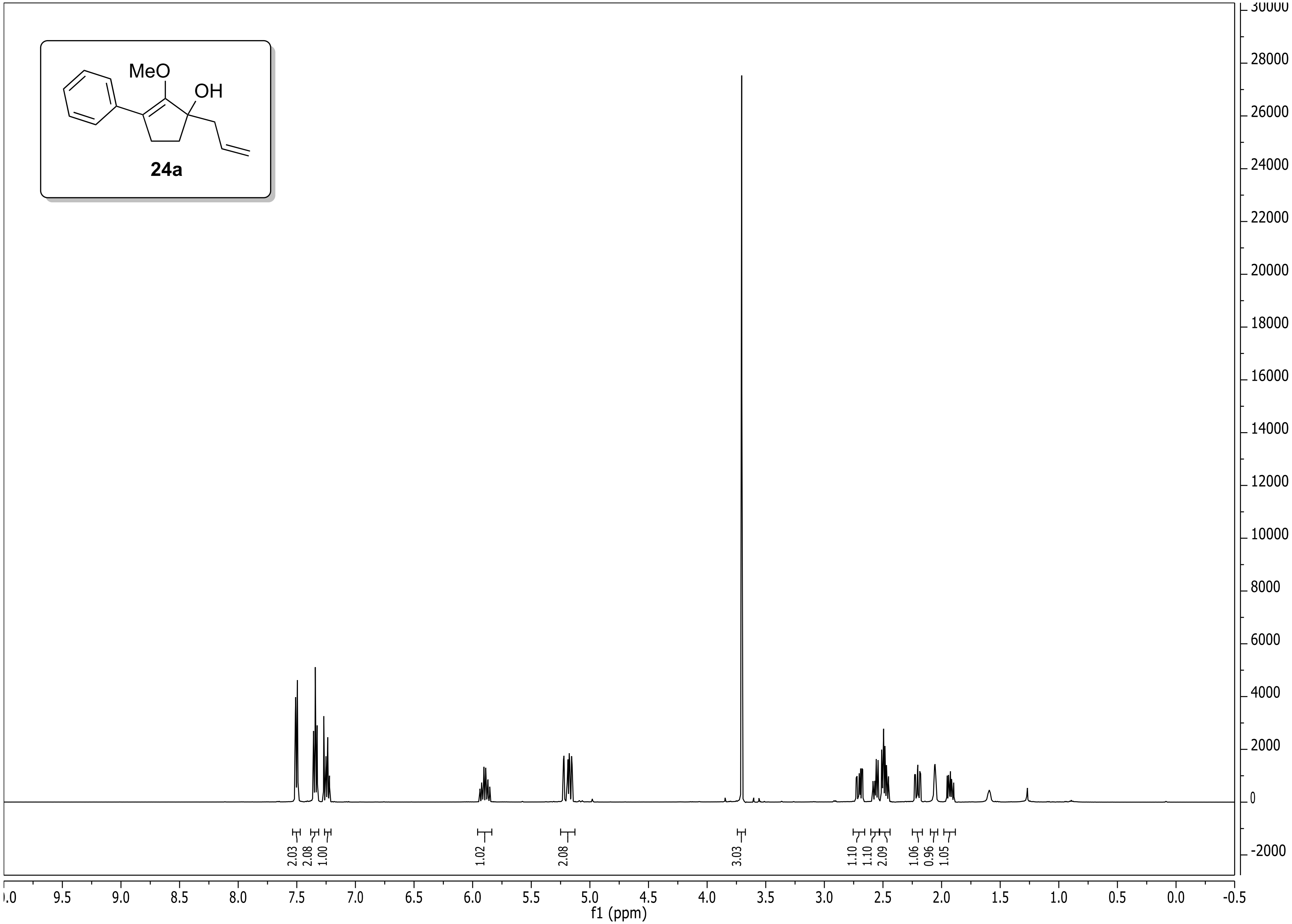
MeO
OH
24a
2.03
2.08
1.00
1.02
2.08
3.03
1.10
1.10
2.09
1.06
0.96
1.05
f1 (ppm)

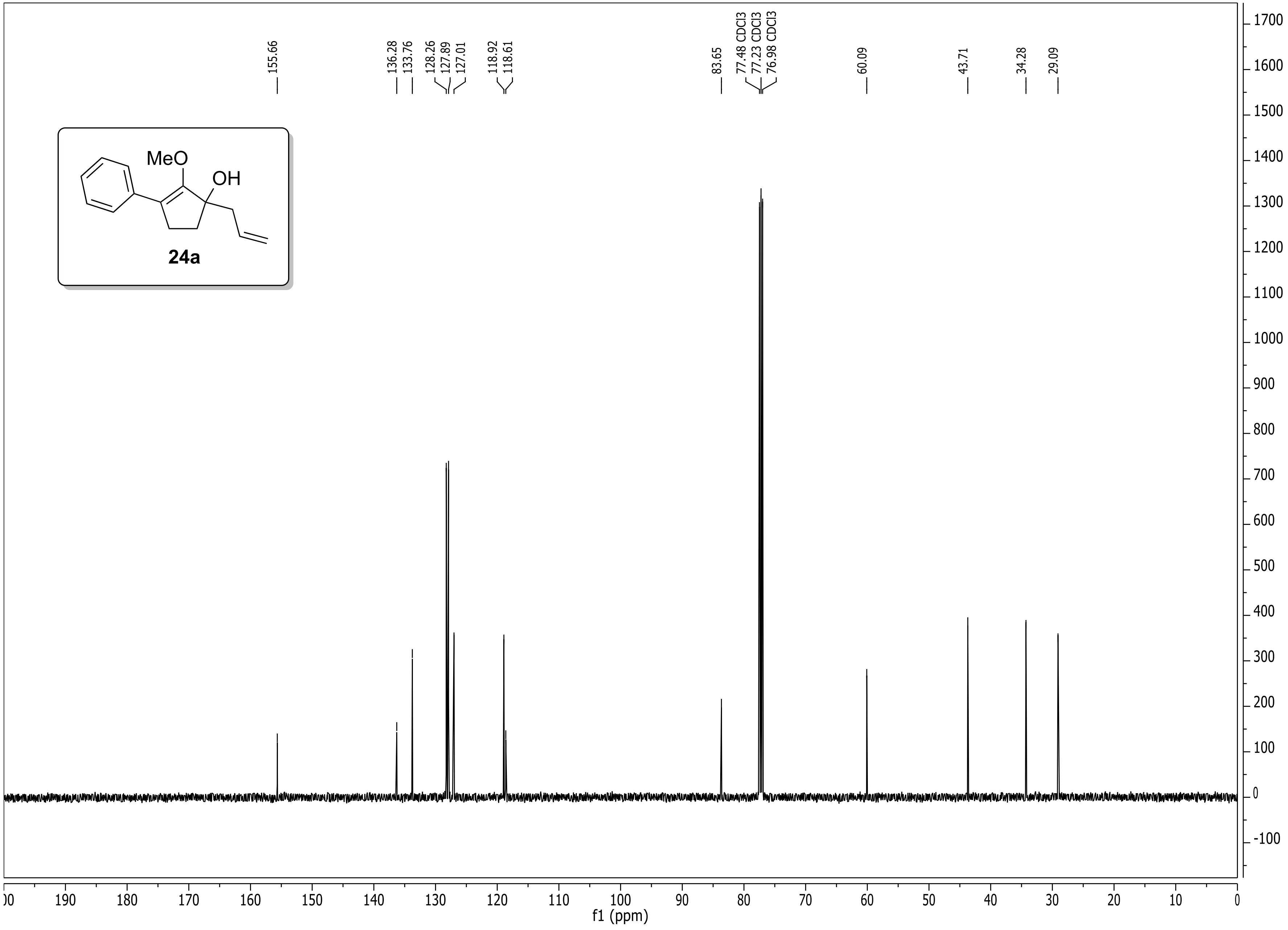
155.66
136.28
133.76
128.26
127.89
127.01
118.92
118.61
83.65
77.48 CDCl3
77.23 CDCl3
76.98 CDCl3
60.09
43.71
34.28
29.09
MeO
OH
24a
f1 (ppm)

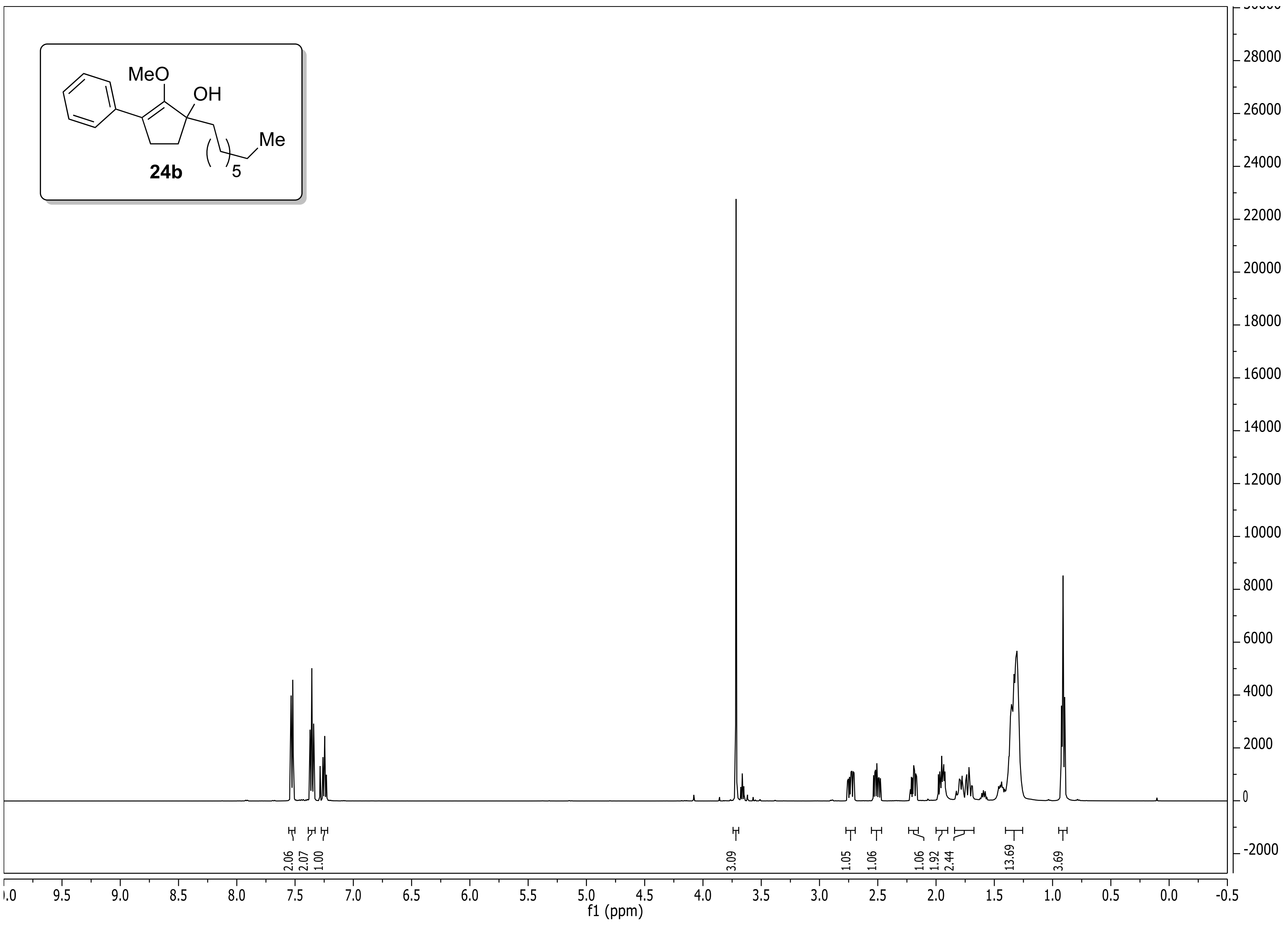
MeO
OH
Me
5
24b
2.06
2.07
1.00
3.09
1.05
1.06
1.06
1.92
2.44
13.69
3.69
f1 (ppm)

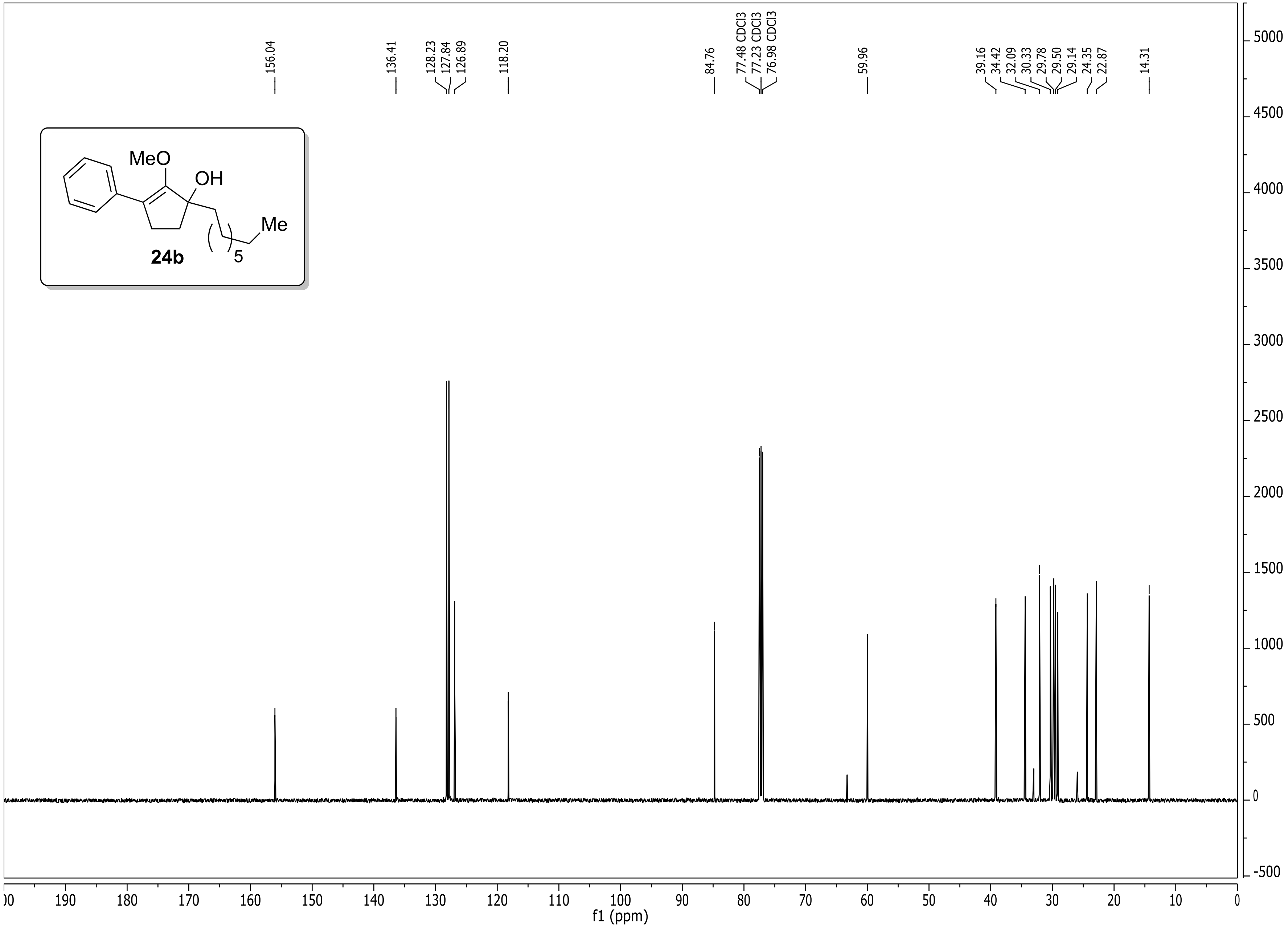

156.04
136.41
128.23
127.84
126.89
118.20
84.76
77.48 CDCl3
77.23 CDCl3
76.98 CDCl3
59.96
39.16
34.42
32.09
30.33
29.78
29.50
29.14
24.35
22.87
14.31
MeO
OH
Me
5
24b
f1 (ppm)

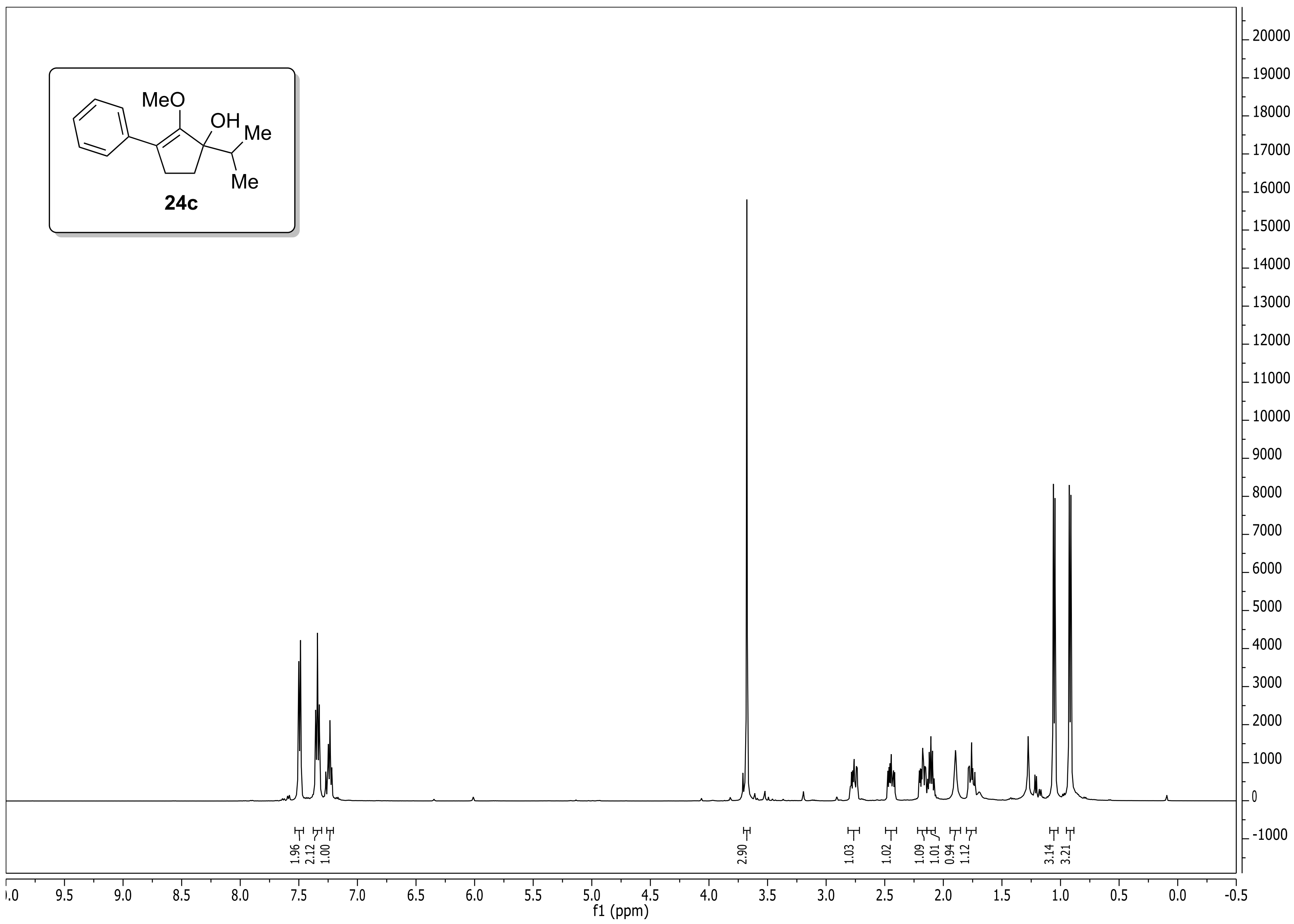
MeO
OH
Me
Me
24c
1.96
2.12
1.00
2.90
1.03
1.02
1.09
1.01
0.94
1.12
3.14
3.21
f1 (ppm)

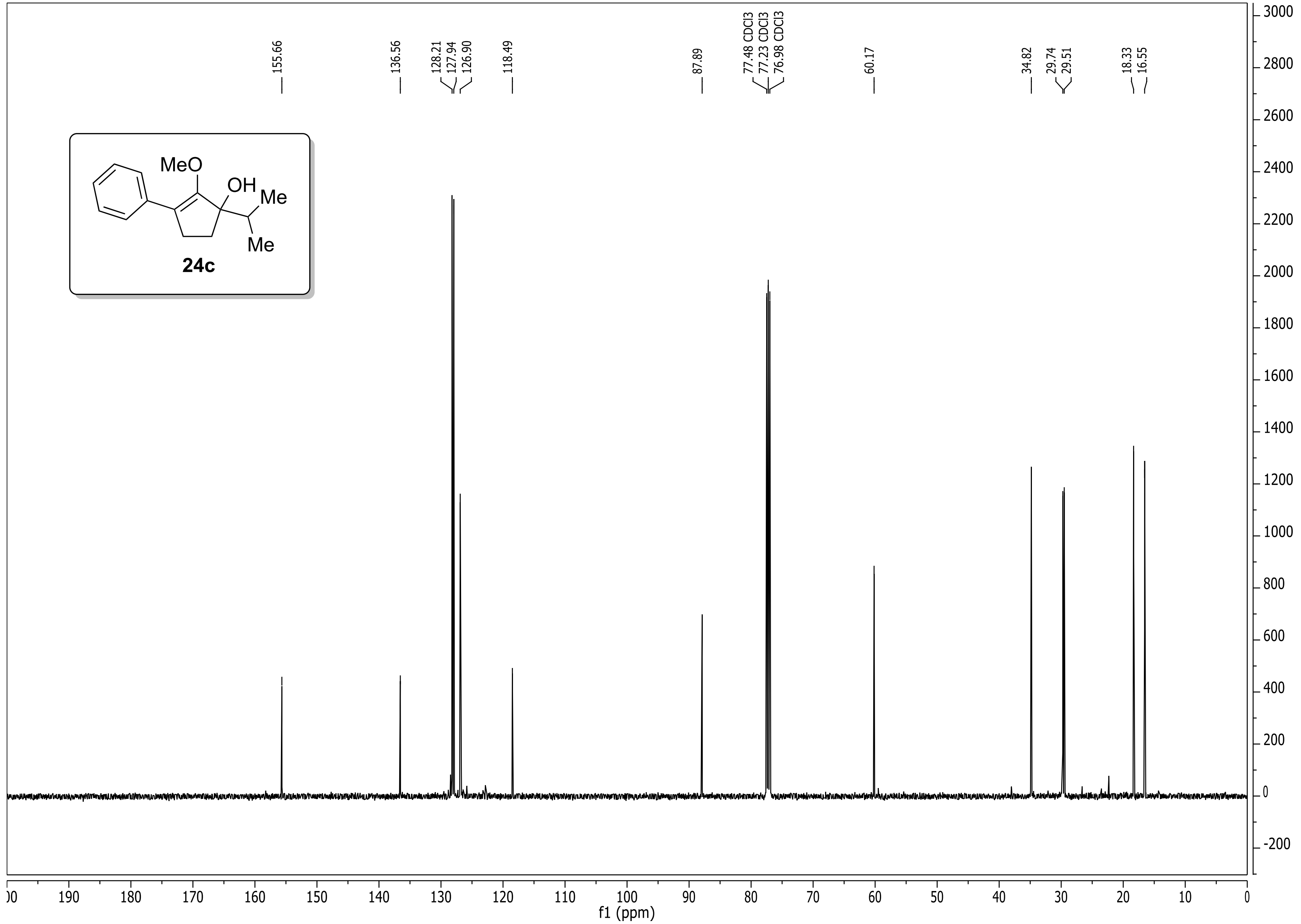
155.66
136.56
128.21
127.94
126.90
118.49
87.89
77.48 CDCl3
77.23 CDCl3
76.98 CDCl3
60.17
34.82
29.74
29.51
18.33
16.55
MeO
OH
Me
Me
24c
f1 (ppm)

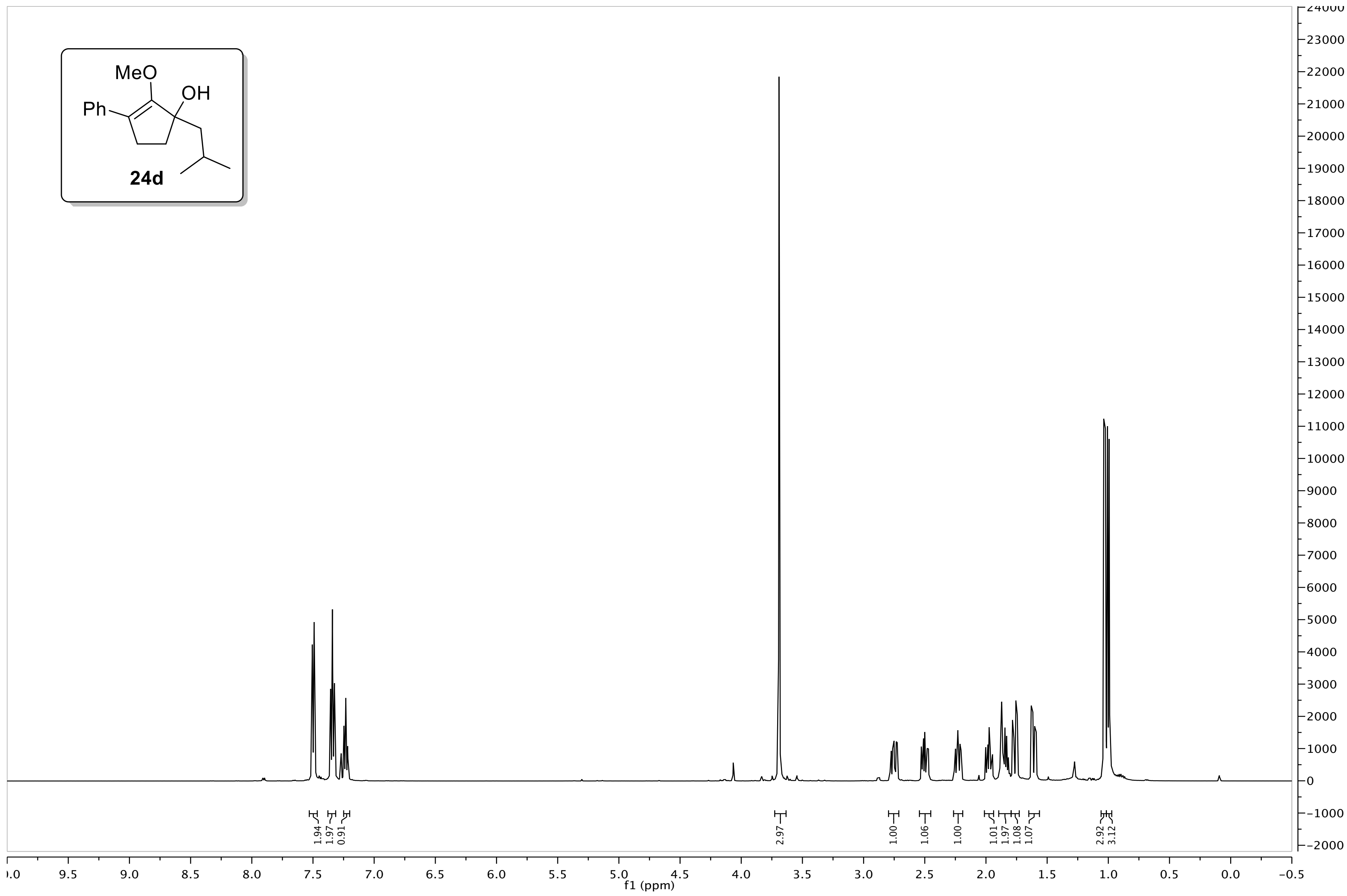
MeO
OH
Ph
24d
f1 (ppm)
1.94
1.97
0.91
2.97
1.00
1.06
1.00
1.01
1.97
1.08
1.07
2.92
3.12

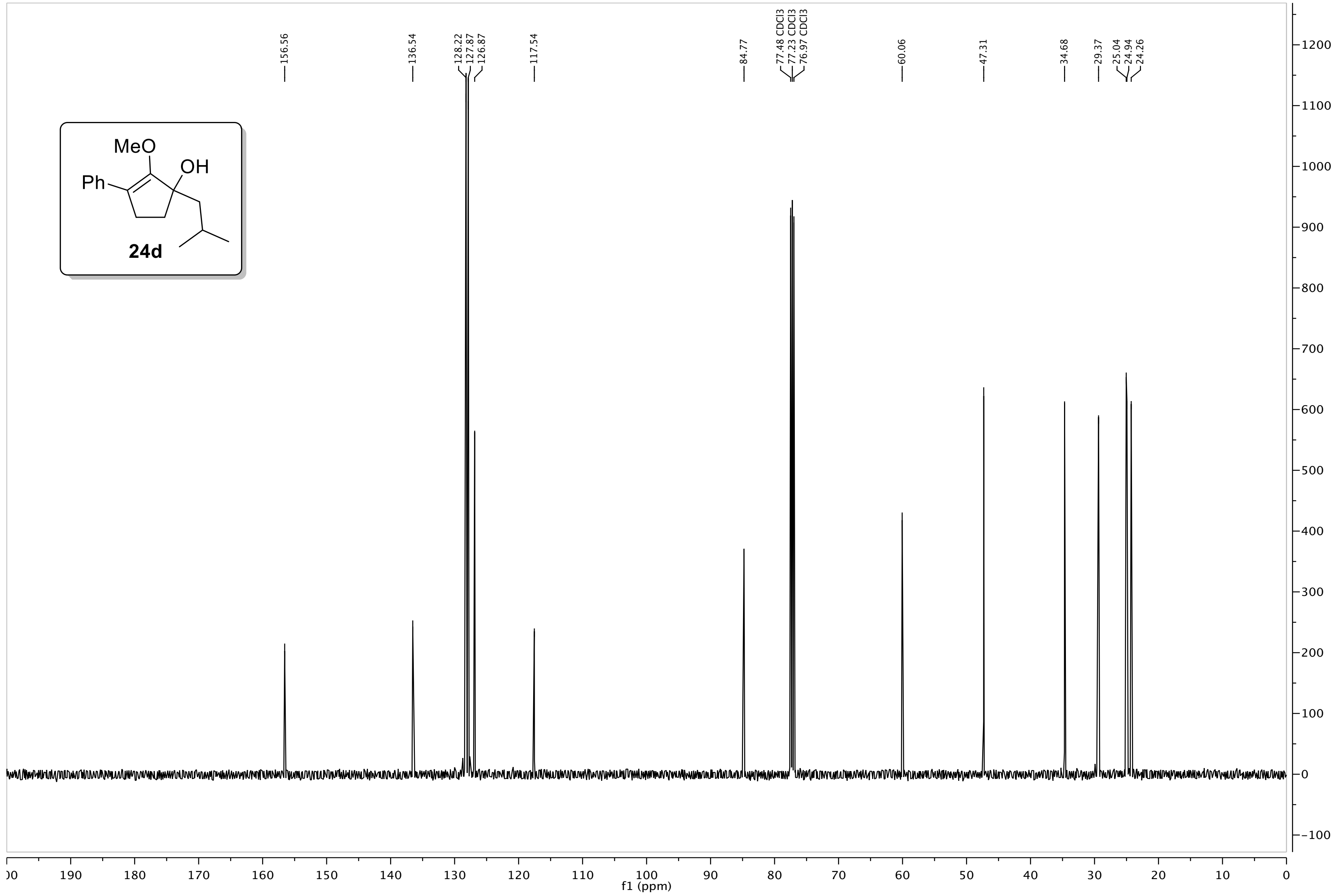

156.56
136.54
128.22
127.87
126.87
117.54
84.77
77.48 CDCl3
77.23 CDCl3
76.97 CDCl3
60.06
47.31
34.68
29.37
25.04
24.94
24.26
MeO
OH
Ph
24d
f1 (ppm)

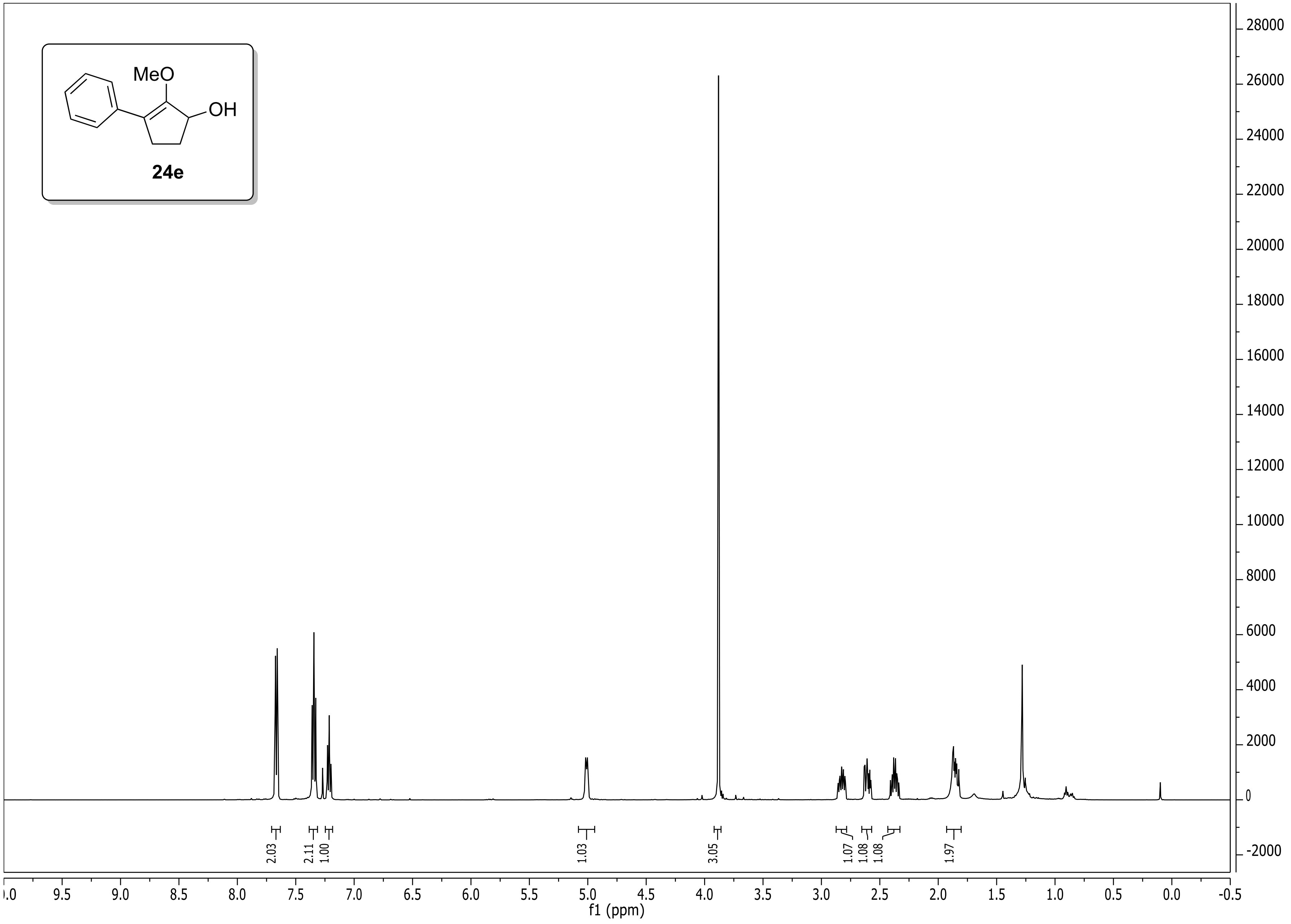

MeO
OH
24e
2.03
2.11
1.00
1.03
3.05
1.07
1.08
1.08
1.97
f1 (ppm)

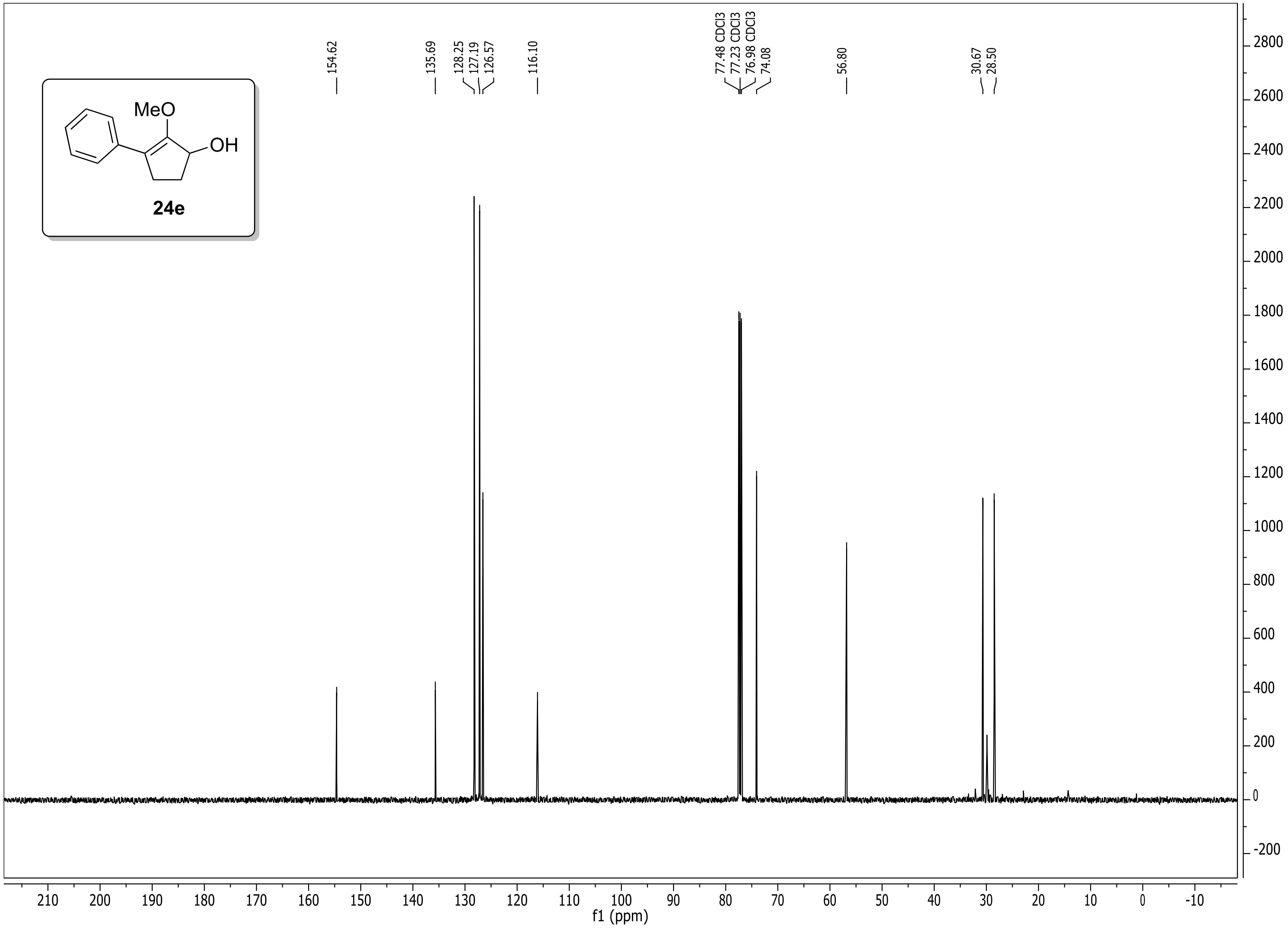
MeO
OH
24e
154.62
135.69
128.25
127.19
126.57
116.10
77.48 CDCl3
77.23 CDCl3
76.98 CDCl3
74.08
56.80
30.67
28.50
f1 (ppm)

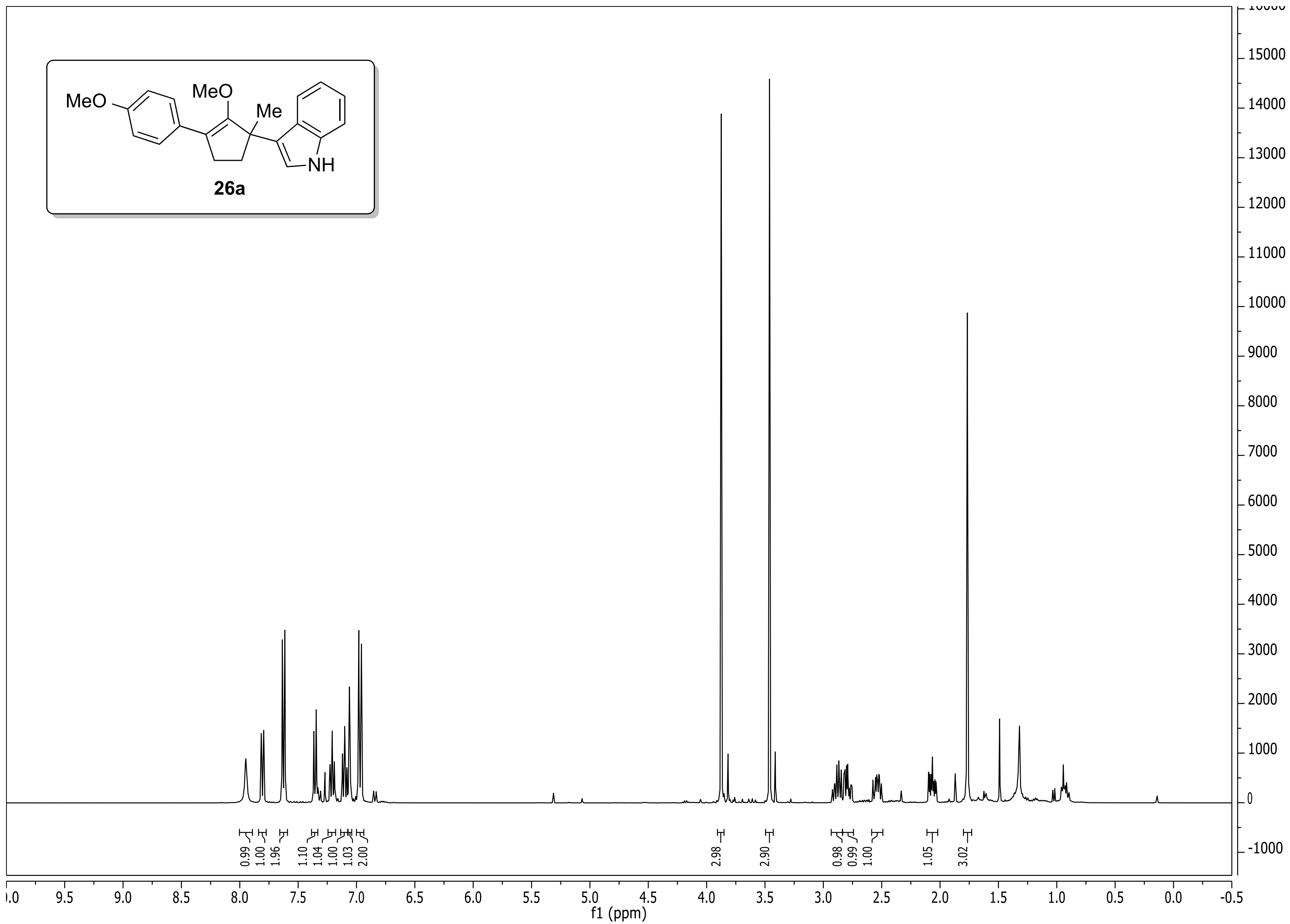
MeO
MeO
Me
NH
26a
f1 (ppm)

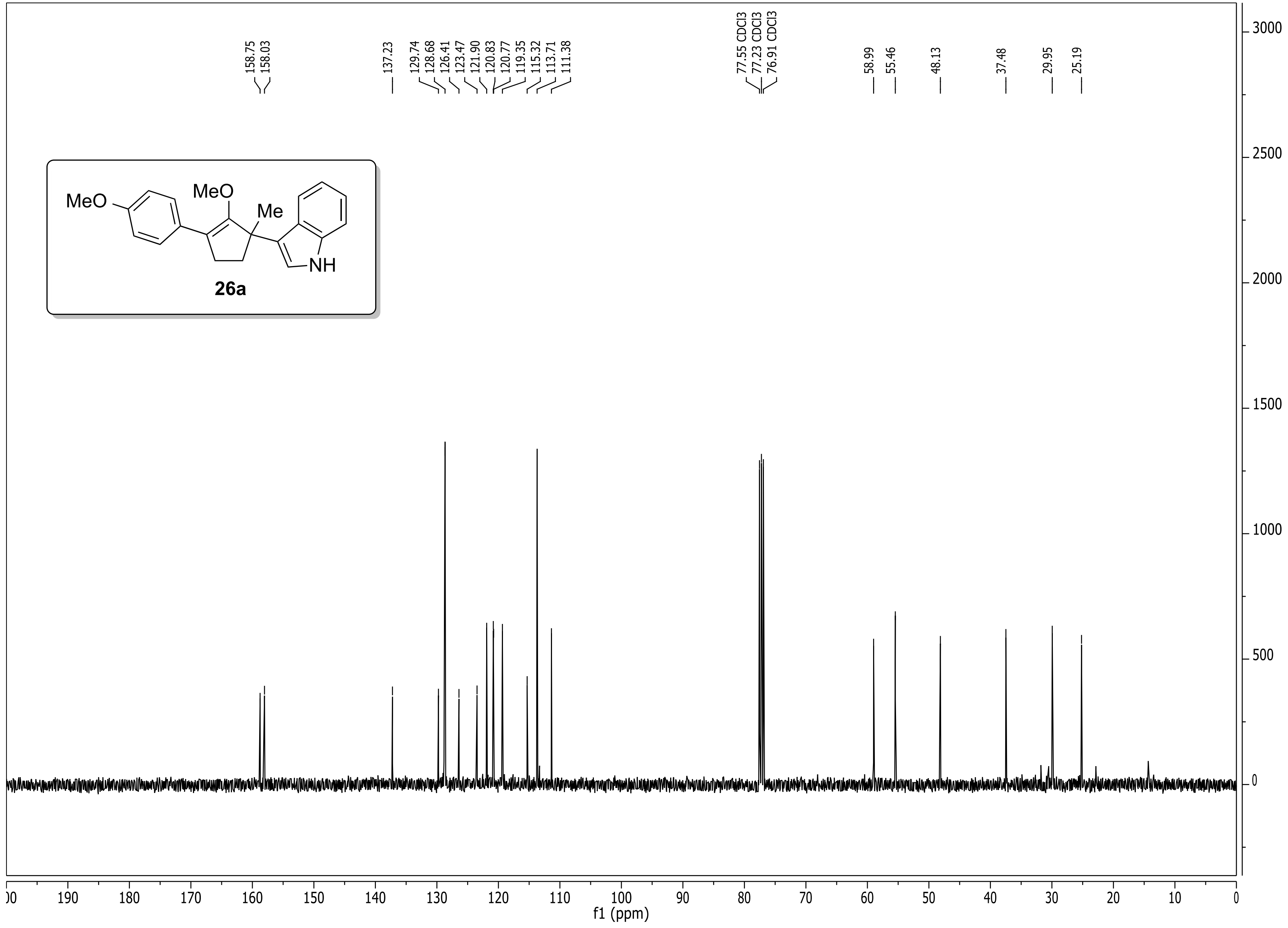
158.75
158.03
137.23
129.74
128.68
126.41
123.47
121.90
120.83
120.77
119.35
115.32
113.71
111.38
77.55 CDCl3
77.23 CDCl3
76.91 CDCl3
58.99
55.46
48.13
37.48
29.95
25.19
MeO
MeO
Me
NH
26a
f1 (ppm)

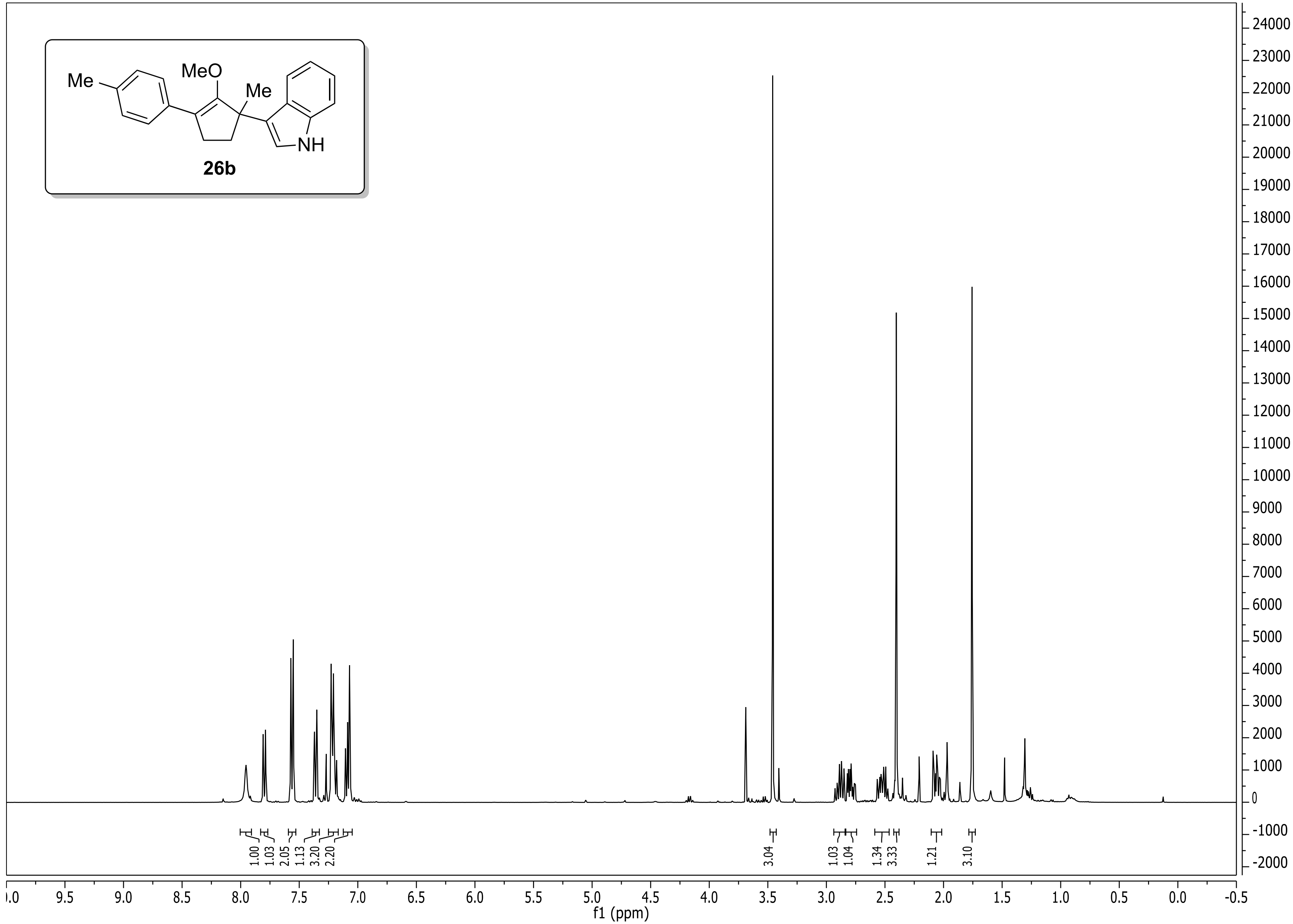
Me
MeO
Me
NH
26b
1.00
1.03
2.05
1.13
3.20
2.20
3.04
1.03
1.04
1.34
3.33
1.21
3.10
f1 (ppm)

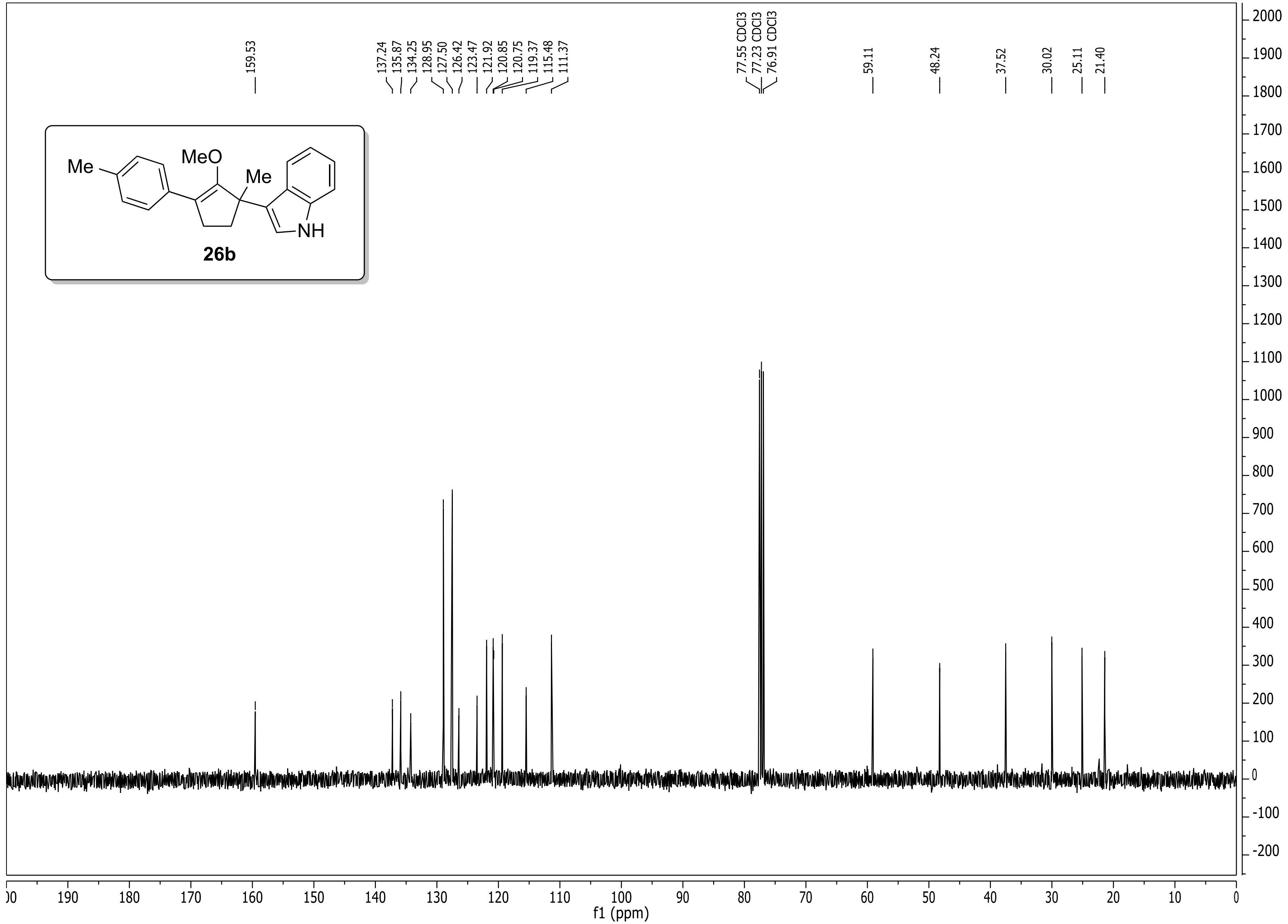
159.53
137.24
135.87
134.25
128.95
127.50
126.42
123.47
121.92
120.85
120.75
119.37
115.48
111.37
77.55 CDCl3
77.23 CDCl3
76.91 CDCl3
59.11
48.24
37.52
30.02
25.11
21.40
Me
MeO
Me
NH
26b
f1 (ppm)

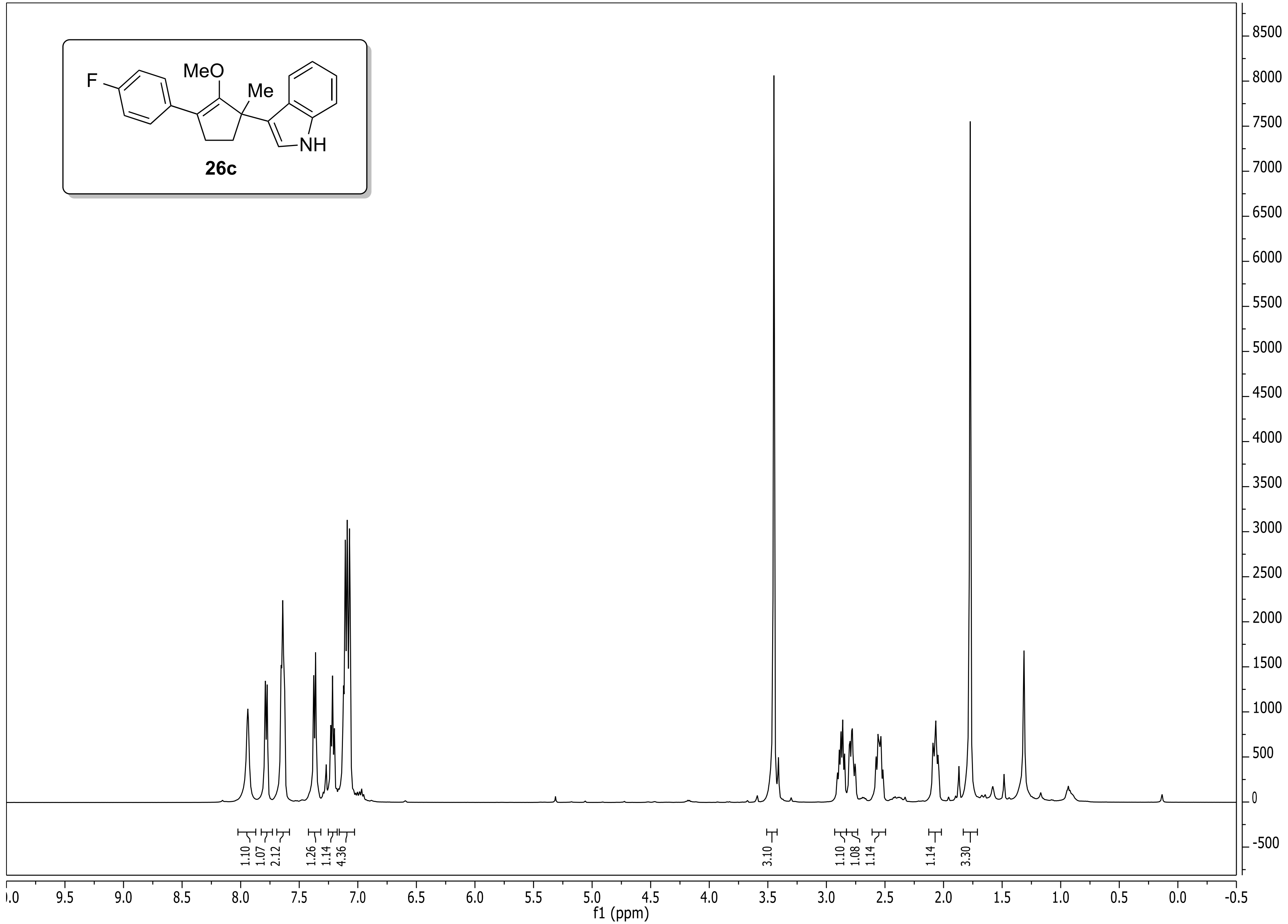
F
MeO
Me
NH
26c
1.10
1.07
2.12
1.26
1.14
4.36
3.10
1.10
1.08
1.14
1.14
3.30
f1 (ppm)

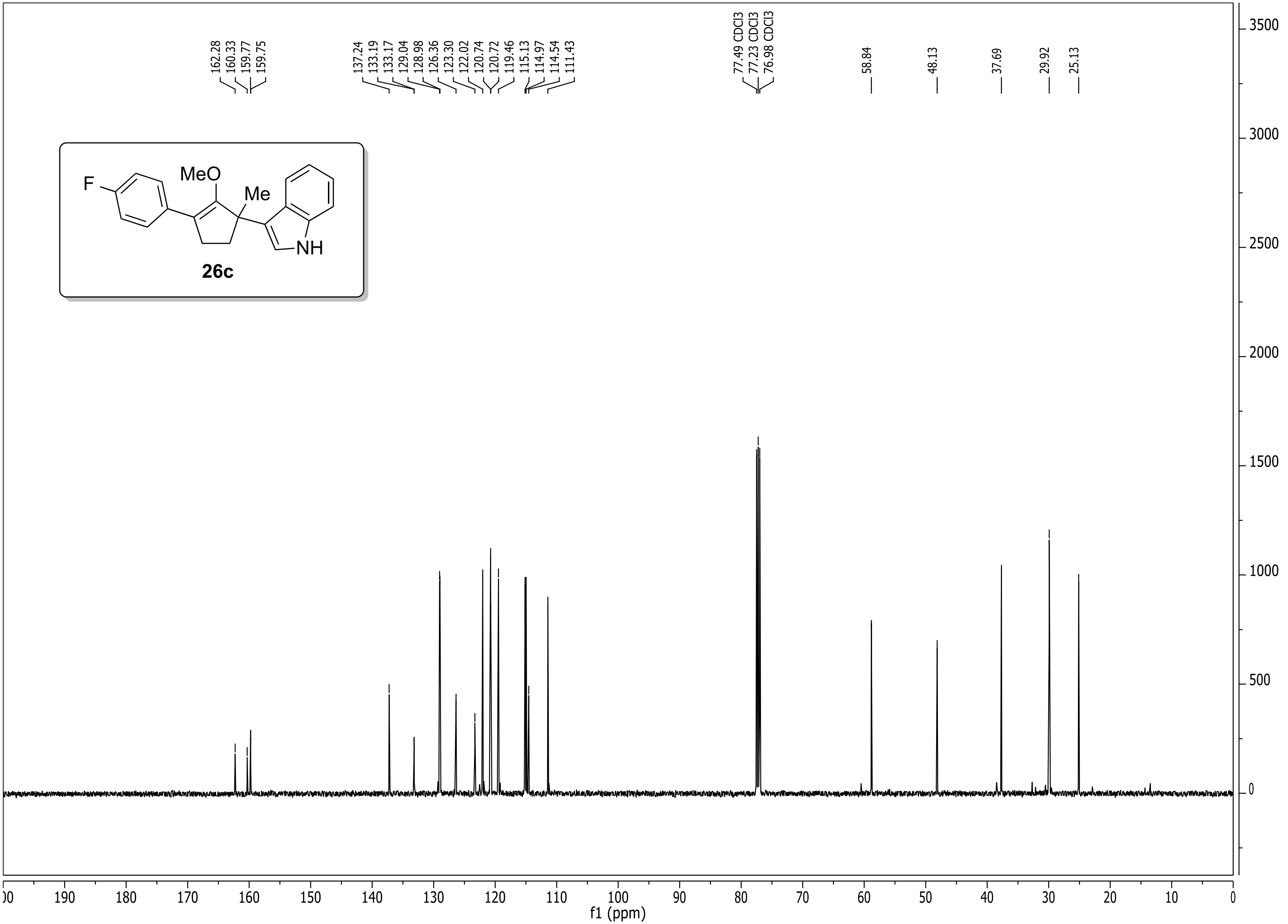
162.28
160.33
159.77
159.75
137.24
133.19
133.17
129.04
128.98
126.36
123.30
122.02
120.74
120.72
119.46
115.13
114.97
114.54
111.43
77.49 CDCl3
77.23 CDCl3
76.98 CDCl3
58.84
48.13
37.69
29.92
25.13
F
MeO
Me
NH
26c
3500
3000
2500
2000
1500
1000
500
0
190
180
170
160
150
140
130
120
110
100
90
80
70
60
50
40
30
20
10
0
f1 (ppm)

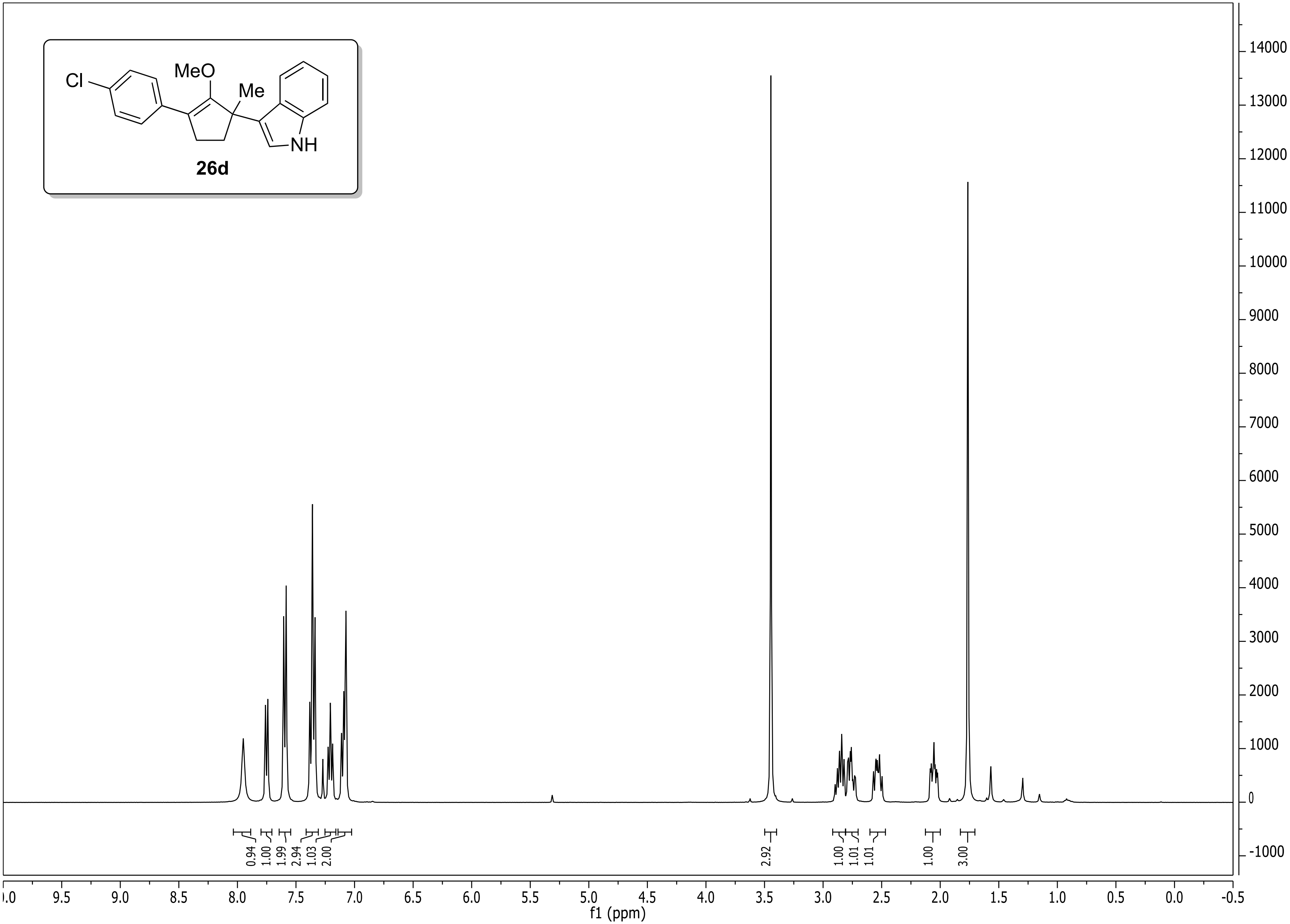
Cl
MeO
Me
NH
26d
0.94
1.00
1.99
2.94
1.03
2.00
2.92
1.00
1.01
1.01
1.00
3.00
f1 (ppm)

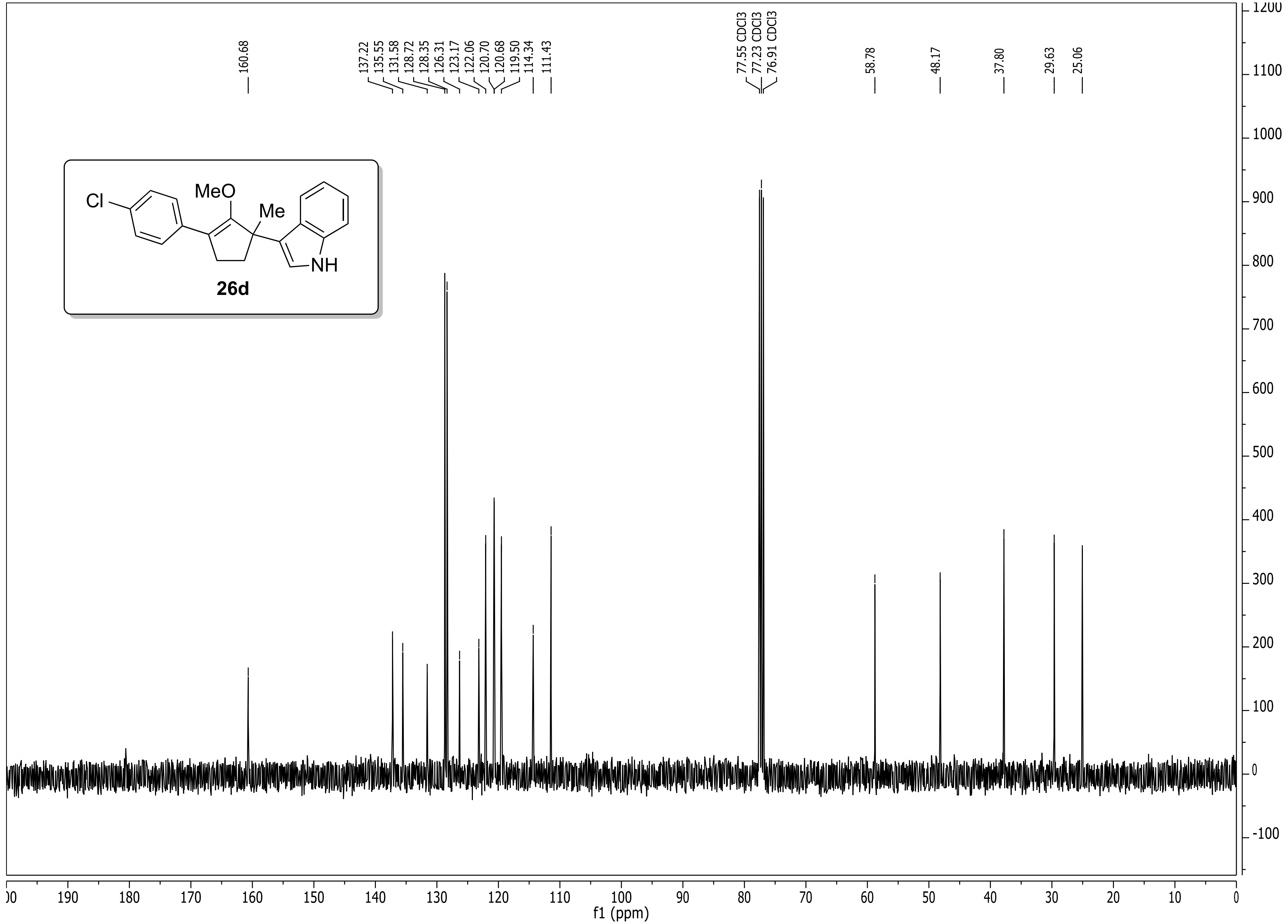

Cl
MeO
Me
NH
26d
160.68
137.22
135.55
131.58
128.72
128.35
126.31
123.17
122.06
120.70
120.68
119.50
114.34
111.43
77.55 CDCl3
77.23 CDCl3
76.91 CDCl3
58.78
48.17
37.80
29.63
25.06
f1 (ppm)

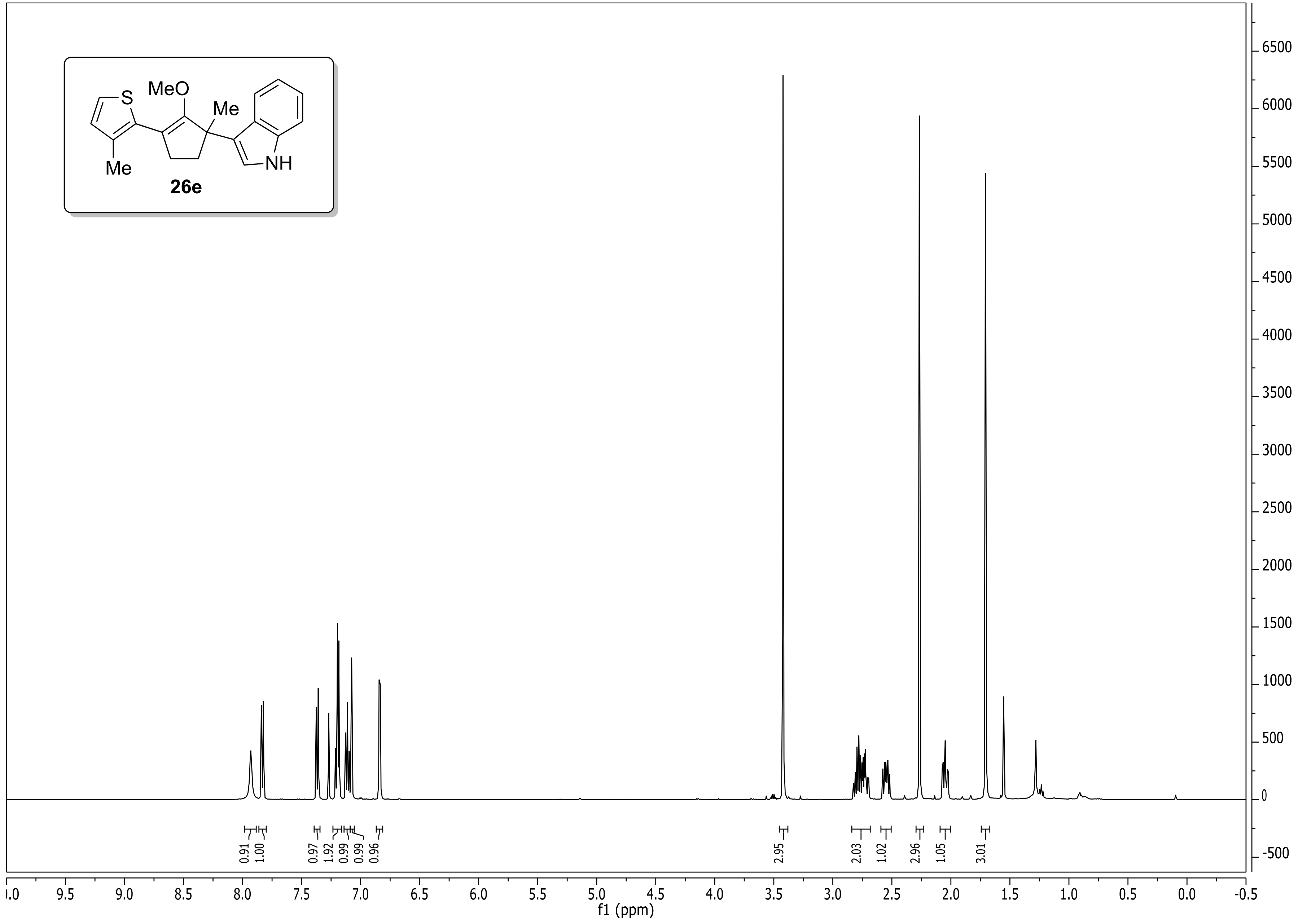

MeO
Me
S
Me
NH
26e
6500
6000
5500
5000
4500
4000
3500
3000
2500
2000
1500
1000
500
0
-500
0.91
1.00
0.97
1.92
0.99
0.99
0.96
2.95
2.03
1.02
2.96
1.05
3.01
9.5
9.0
8.5
8.0
7.5
7.0
6.5
6.0
5.5
5.0
4.5
4.0
3.5
3.0
2.5
2.0
1.5
1.0
0.5
0.0
-0.5
f1 (ppm)

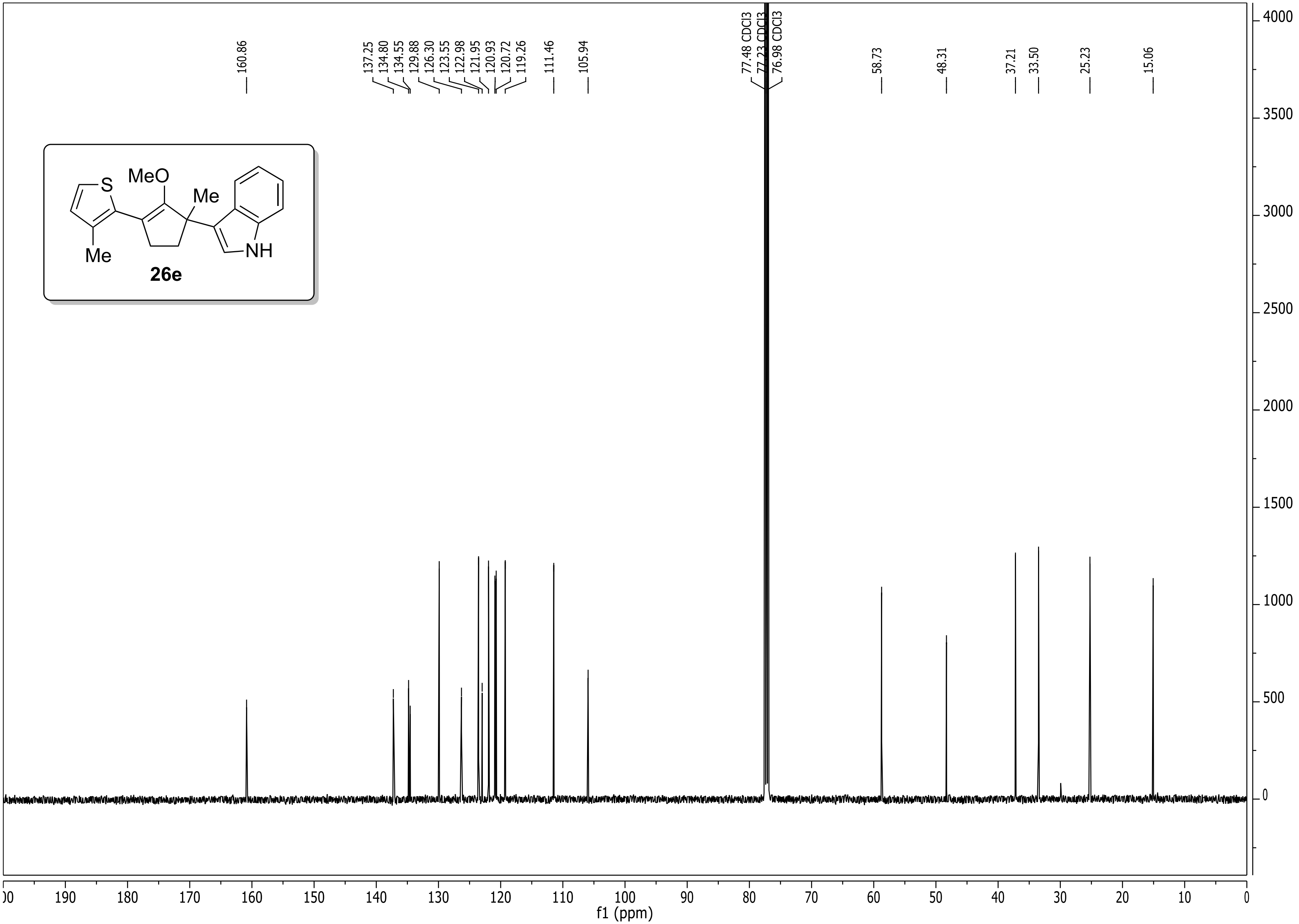
160.86
137.25
134.80
134.55
129.88
126.30
123.55
122.98
121.95
120.93
120.72
119.26
111.46
105.94
77.48 CDCl3
77.23 CDCl3
76.98 CDCl3
58.73
48.31
37.21
33.50
25.23
15.06
S
MeO
Me
Me
NH
26e
f1 (ppm)

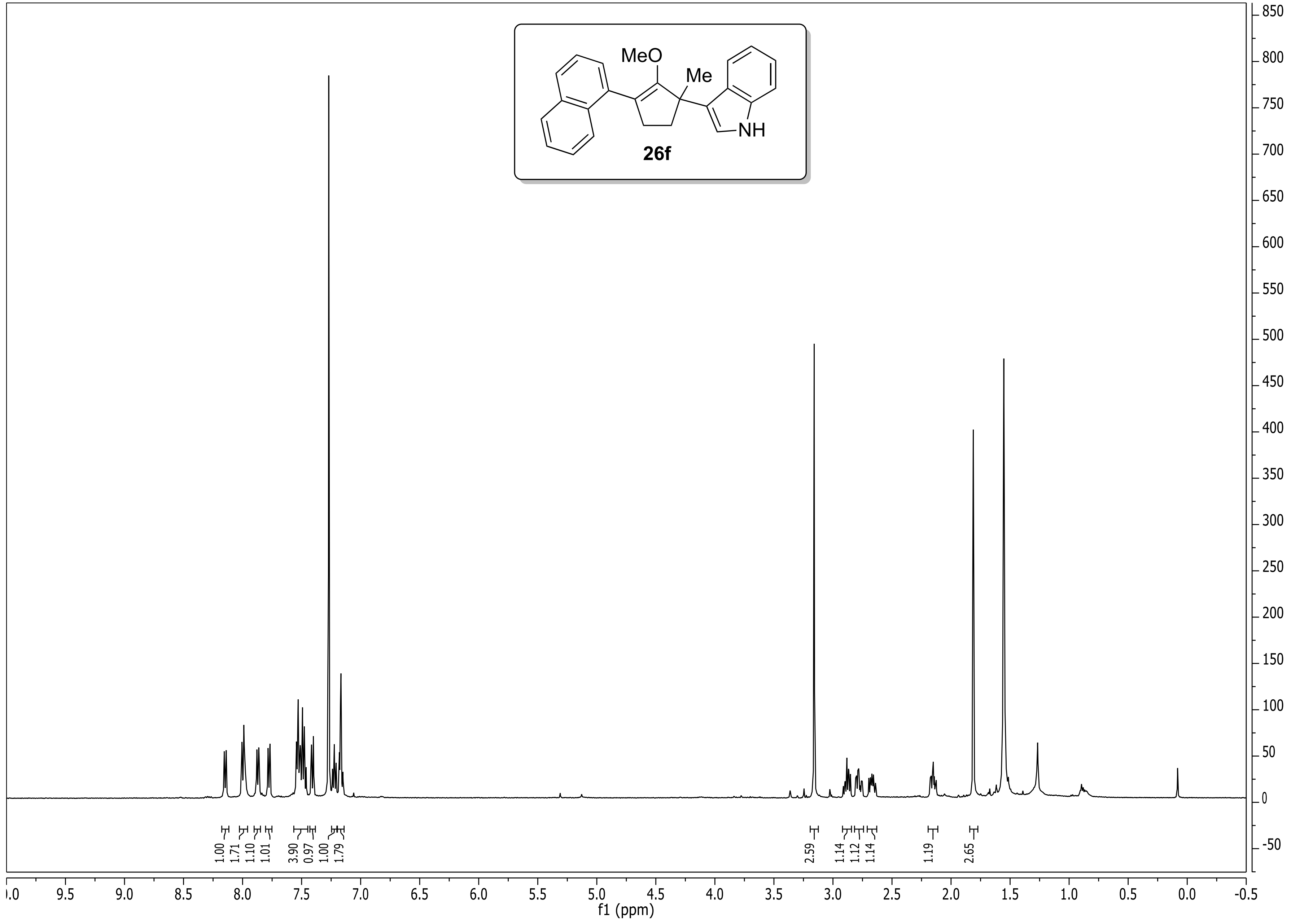
MeO
Me
NH
26f
1.00
1.71
1.10
1.01
3.90
0.97
1.00
1.79
2.59
1.14
1.12
1.14
1.19
2.65
f1 (ppm)

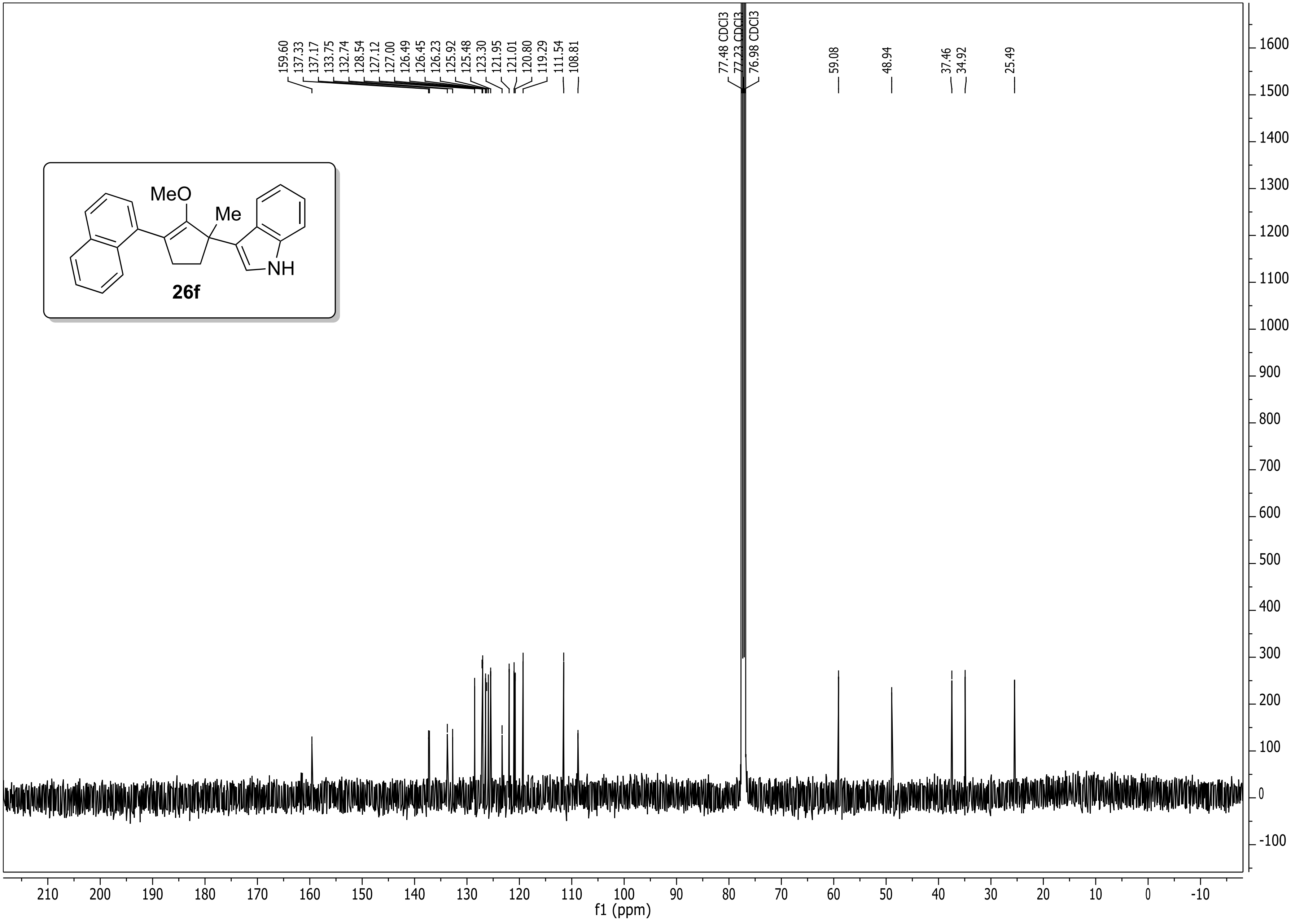
159.60
137.33
137.17
133.75
132.74
128.54
127.12
127.00
126.49
126.45
126.23
125.92
125.48
123.30
121.95
121.01
120.80
119.29
111.54
108.81
77.48 CDCl3
77.23 CDCl3
76.98 CDCl3
59.08
48.94
37.46
34.92
25.49
MeO
Me
NH
26f
f1 (ppm)

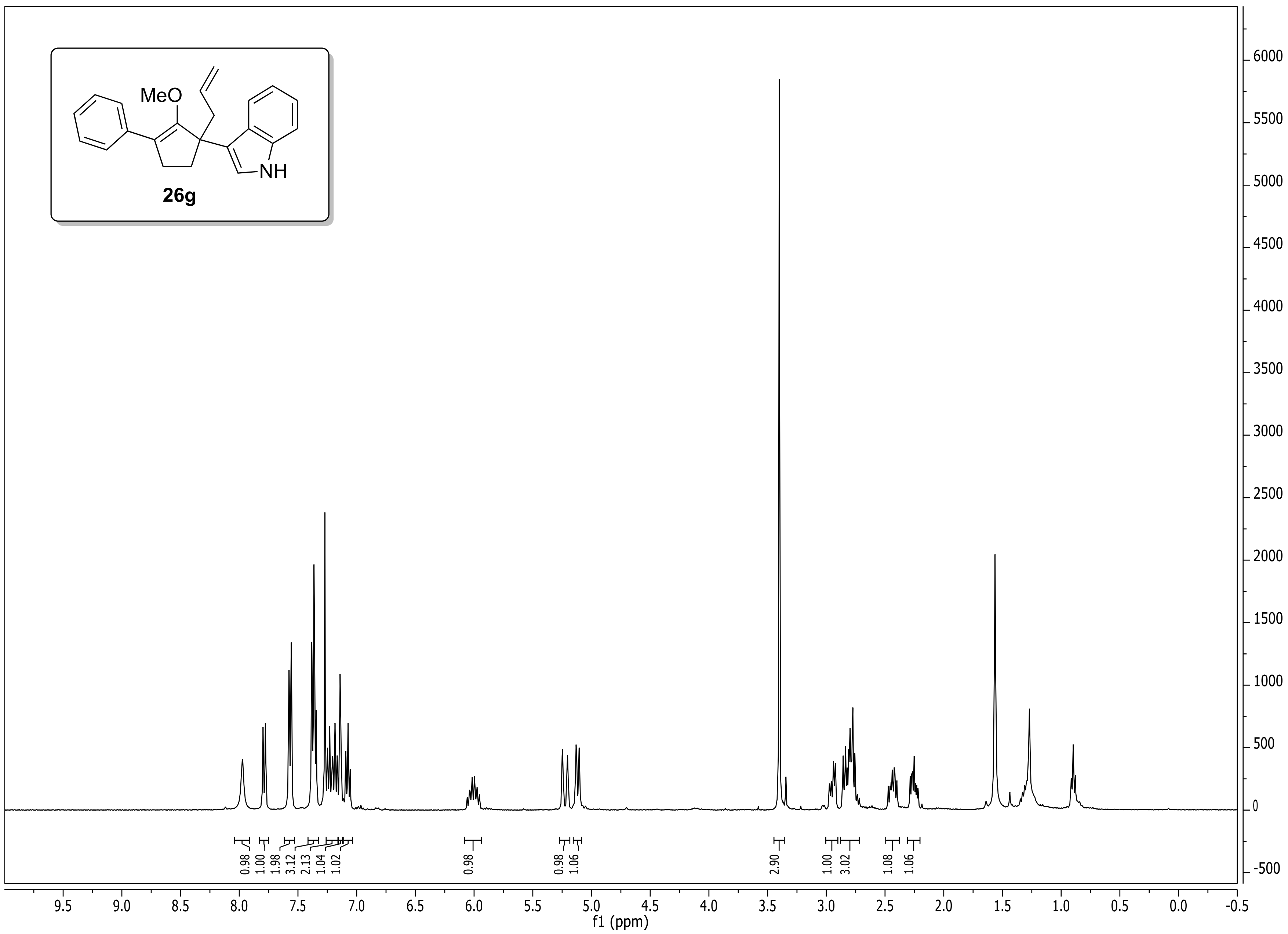
MeO
NH
26g
f1 (ppm)

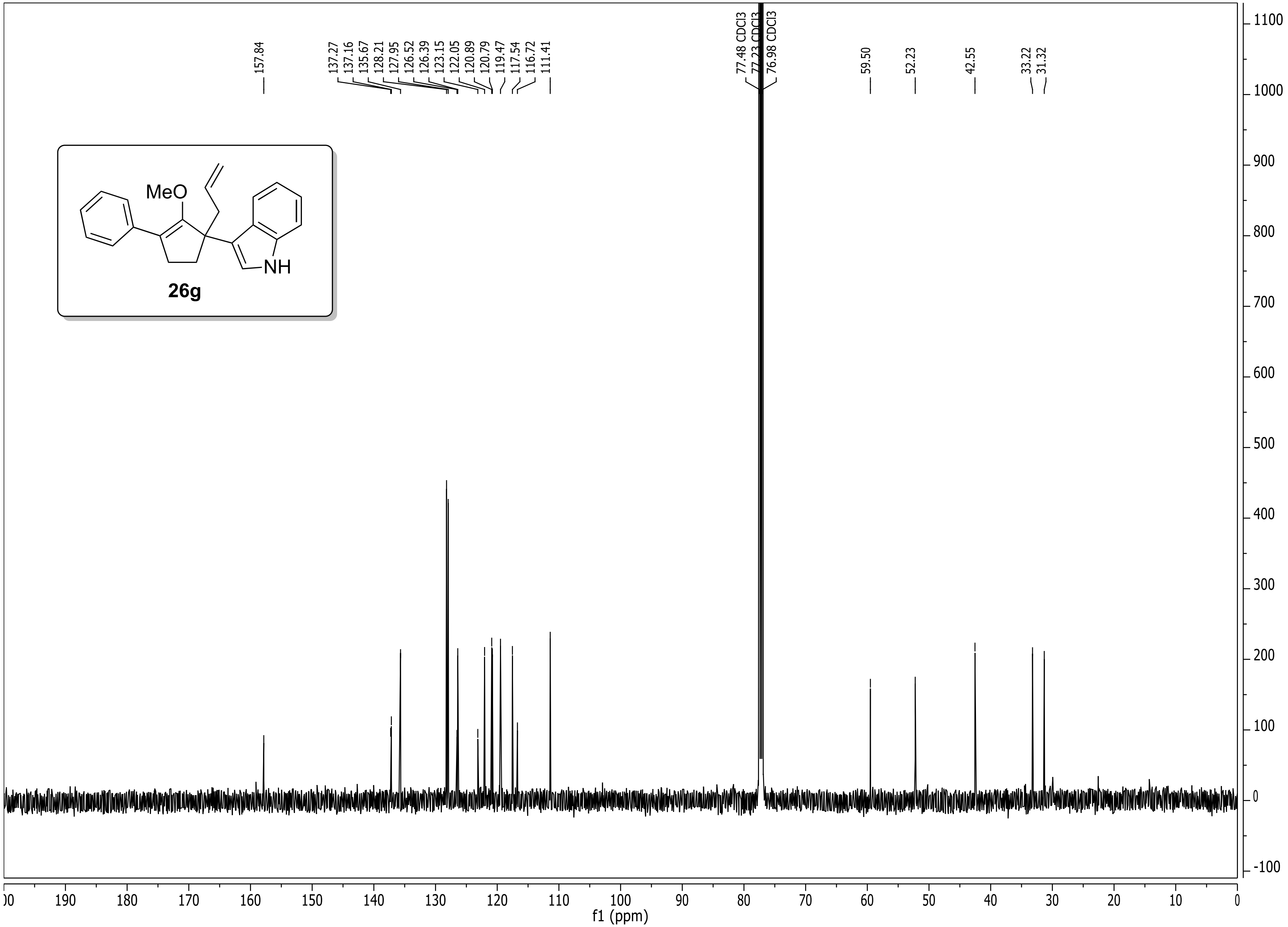
157.84
137.27
137.16
135.67
128.21
127.95
126.52
126.39
123.15
122.05
120.89
120.79
119.47
117.54
116.72
111.41
77.48 CDCl3
77.23 CDCl3
76.98 CDCl3
59.50
52.23
42.55
33.22
31.32
MeO
NH
26g
f1 (ppm)

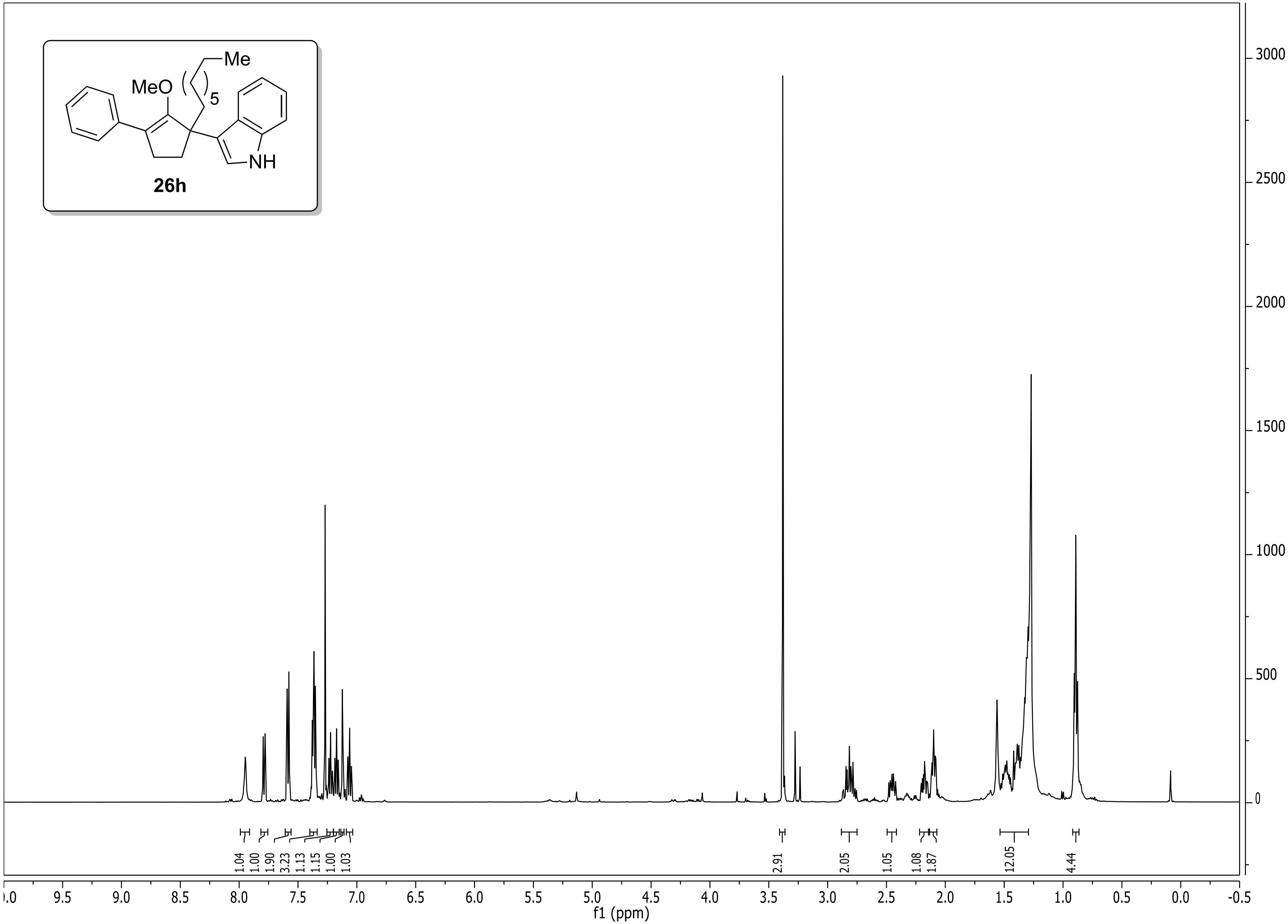
MeO
Me
5
NH
26h
f1 (ppm)

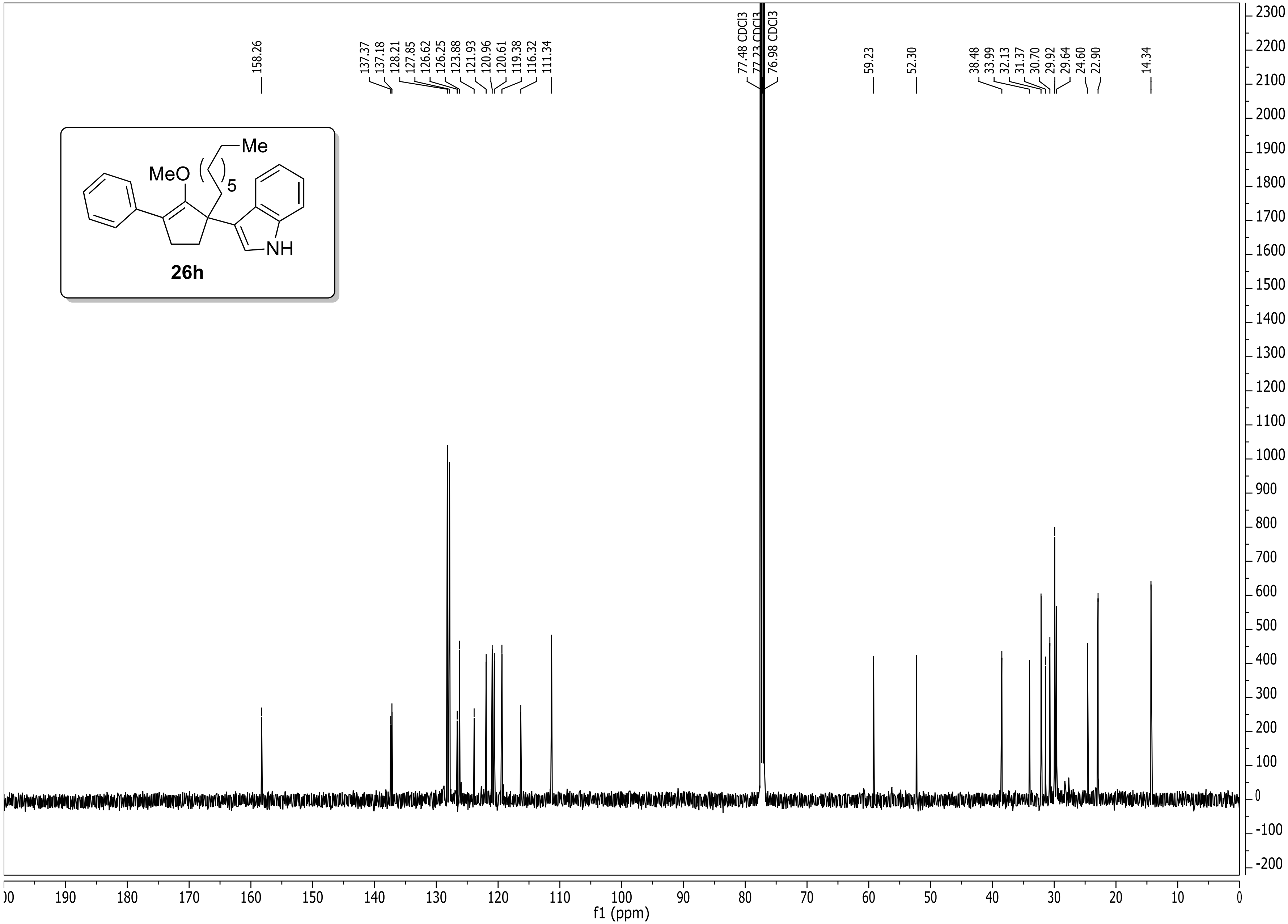

158.26
137.37
137.18
128.21
127.85
126.62
126.25
123.88
121.93
120.96
120.61
119.38
116.32
111.34
77.48 CDCl3
77.23 CDCl3
76.98 CDCl3
59.23
52.30
38.48
33.99
32.13
31.37
30.70
29.92
29.64
24.60
22.90
14.34
MeO
Me
5
NH
26h
f1 (ppm)

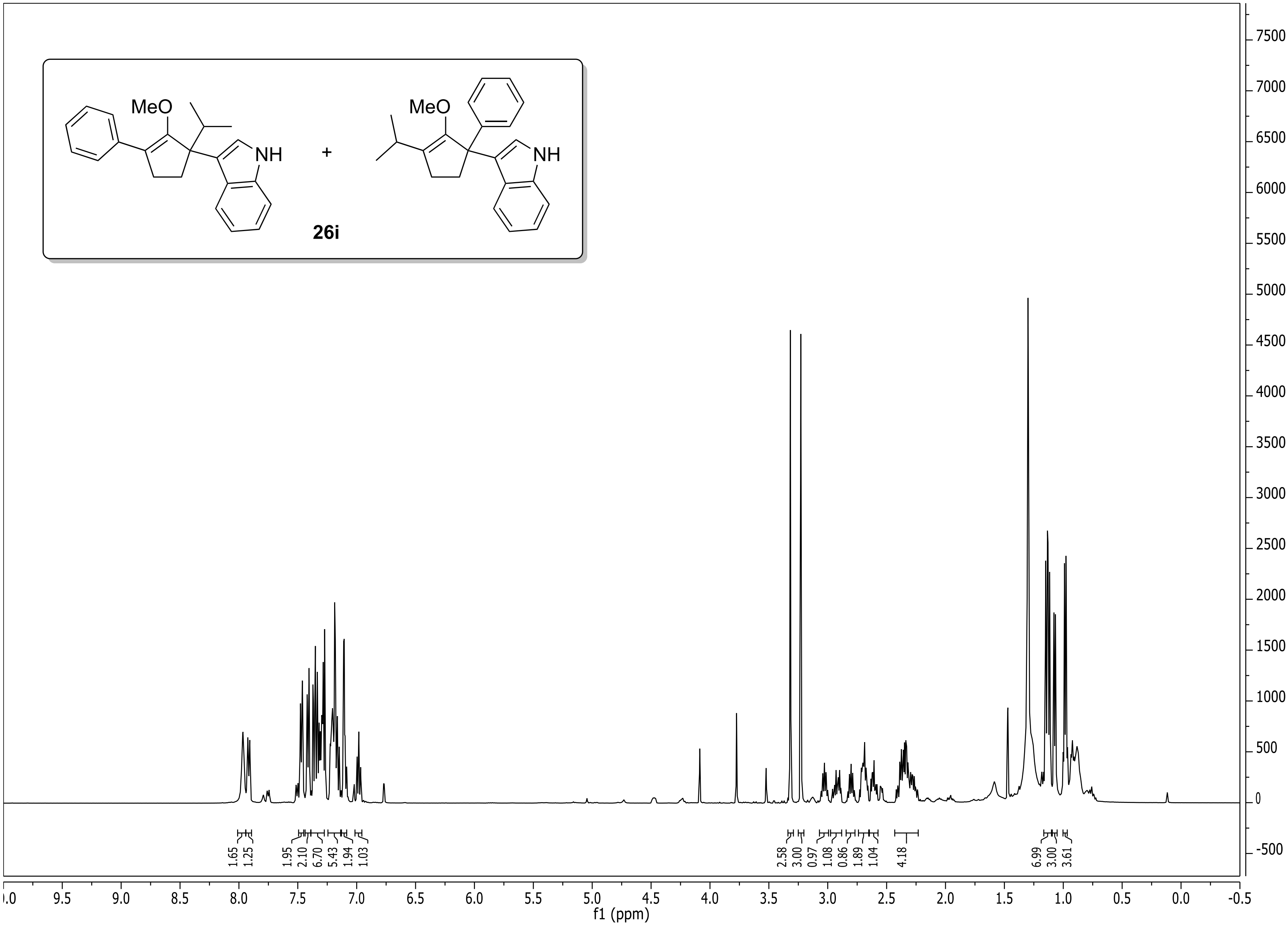
MeO
NH
+
MeO
NH
26i
f1 (ppm)

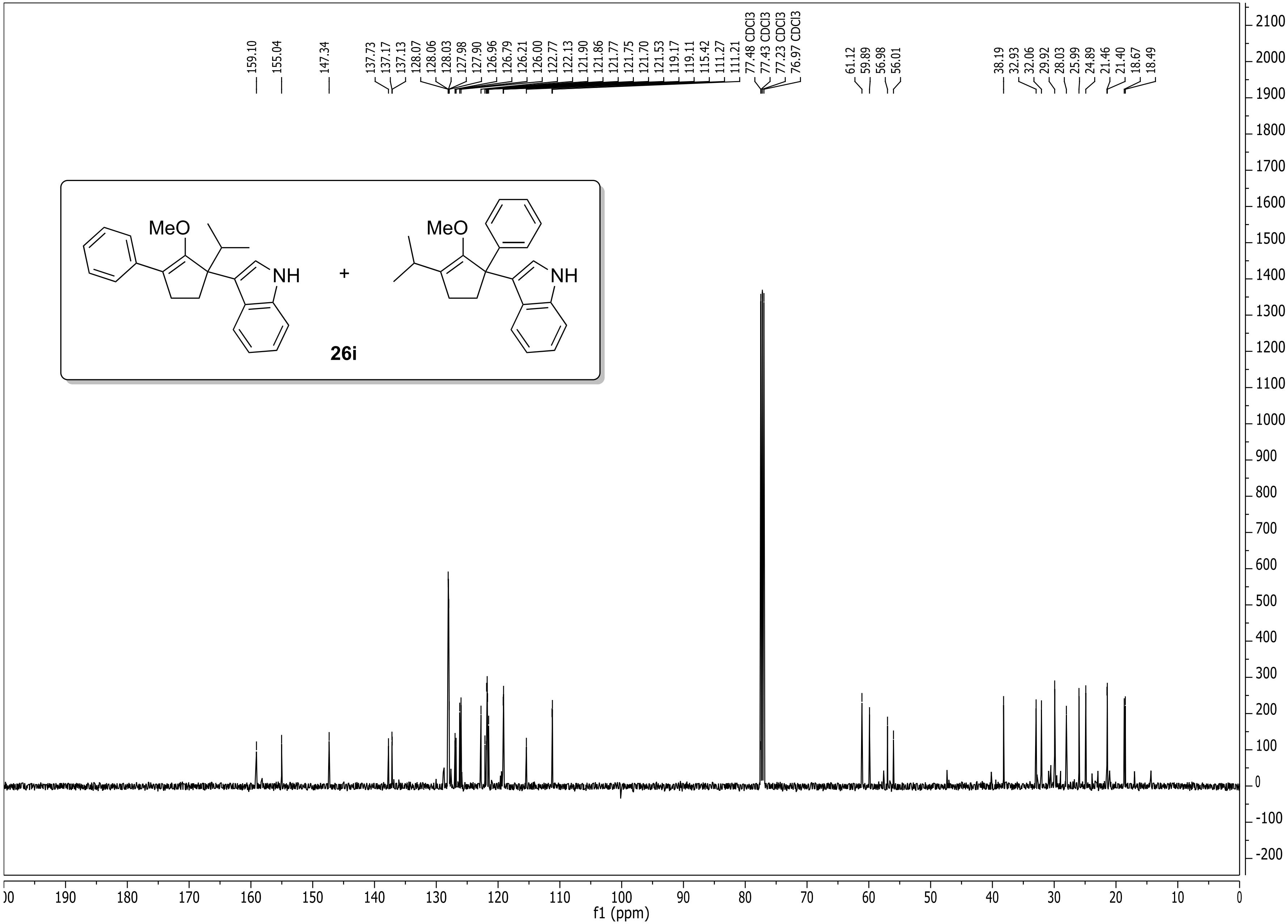
159.10
155.04
147.34
137.73
137.17
137.13
128.07
128.06
128.03
127.98
127.90
126.96
126.79
126.21
126.00
122.77
122.13
121.90
121.86
121.77
121.75
121.70
121.53
119.17
119.11
115.42
111.27
111.21
77.48 CDCl3
77.43 CDCl3
77.23 CDCl3
76.97 CDCl3
61.12
59.89
56.98
56.01
38.19
32.93
32.06
29.92
28.03
25.99
24.89
21.46
21.40
18.67
18.49
MeO
NH
+
26i
f1 (ppm)

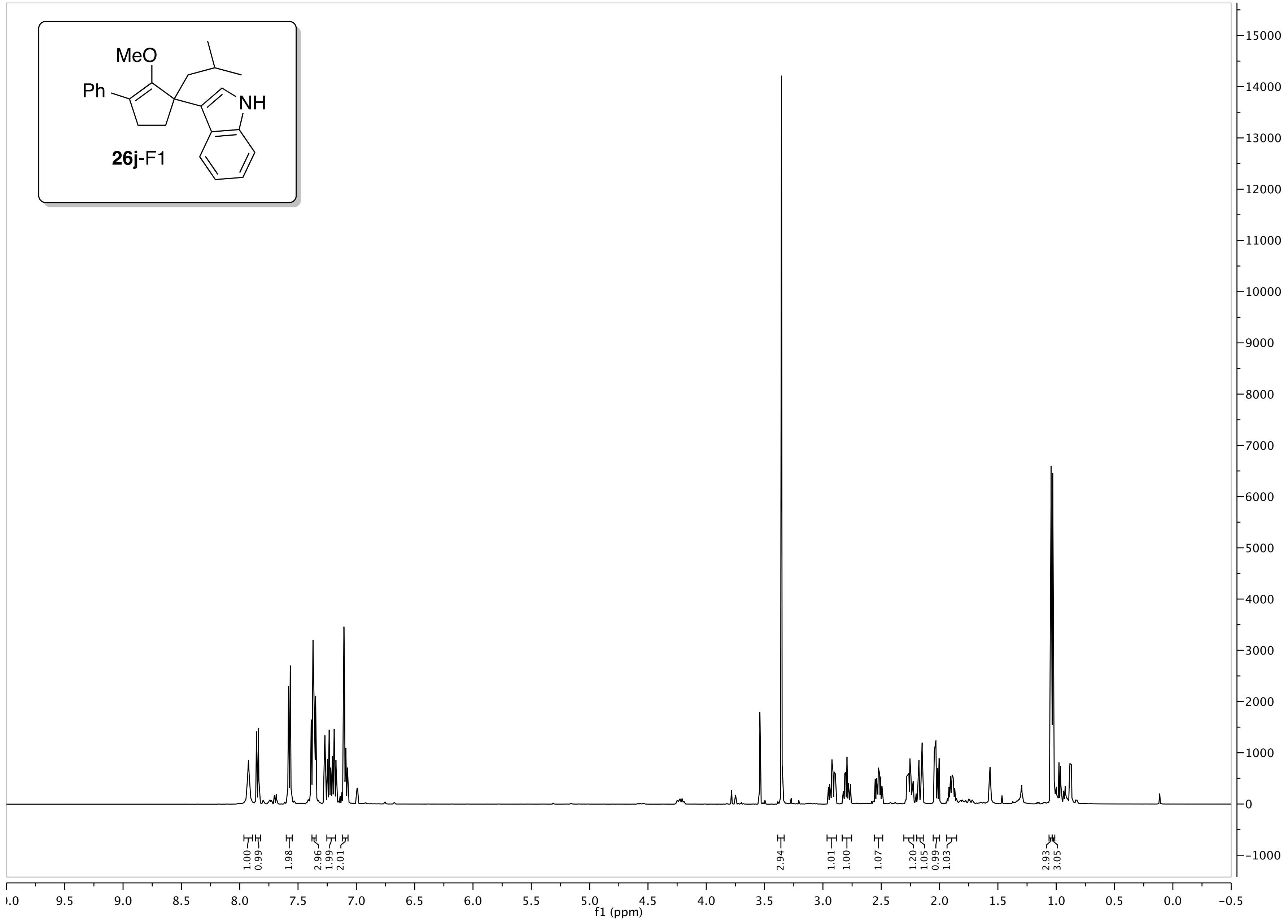

MeO
Ph
NH
26j-F1
f1 (ppm)

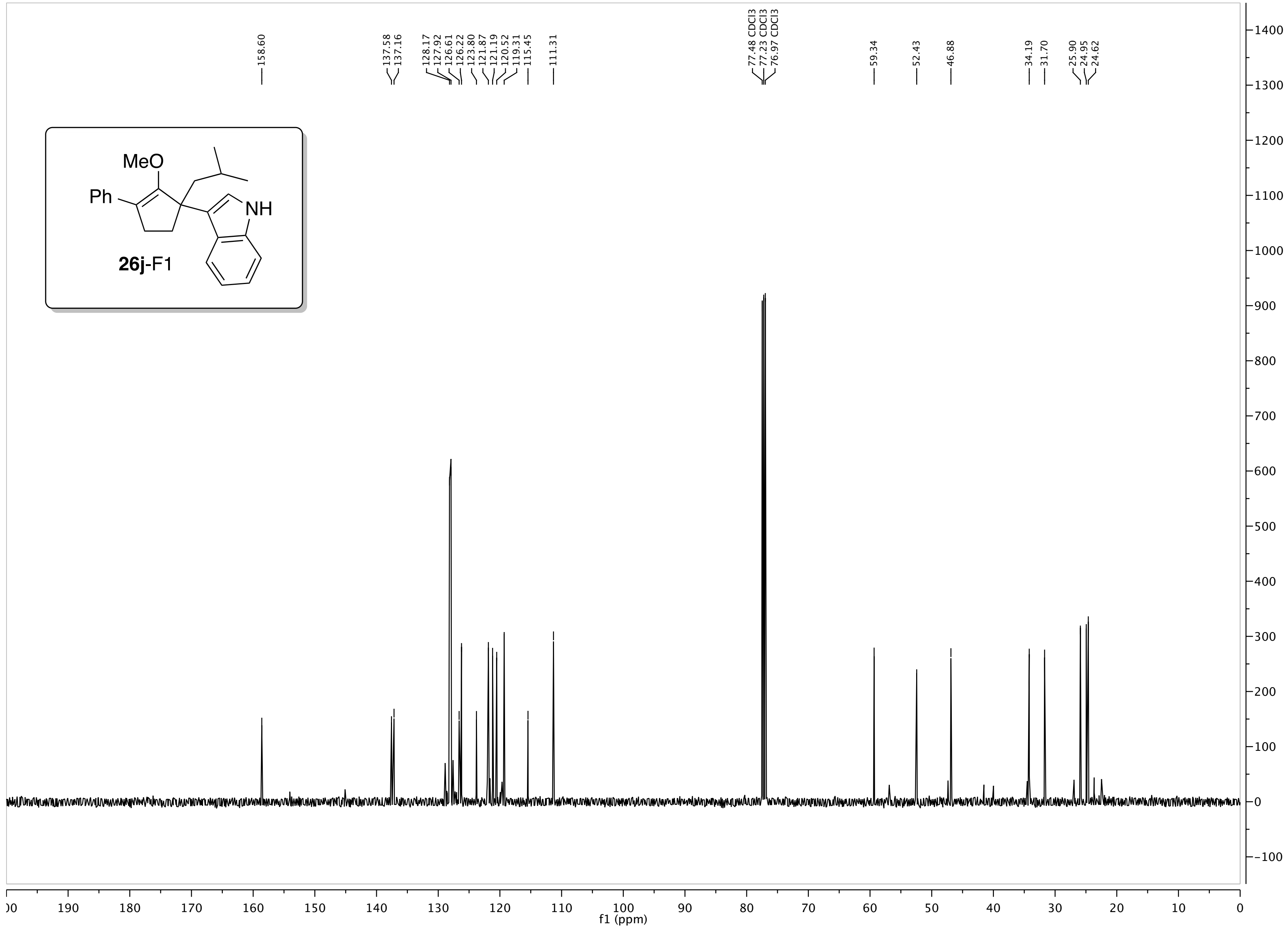

158.60
137.58
137.16
128.17
127.92
126.61
126.22
123.80
121.87
121.19
120.52
119.31
115.45
111.31
77.48 CDCl3
77.23 CDCl3
76.97 CDCl3
59.34
52.43
46.88
34.19
31.70
25.90
24.95
24.62
MeO
Ph
NH
26j-F1
f1 (ppm)

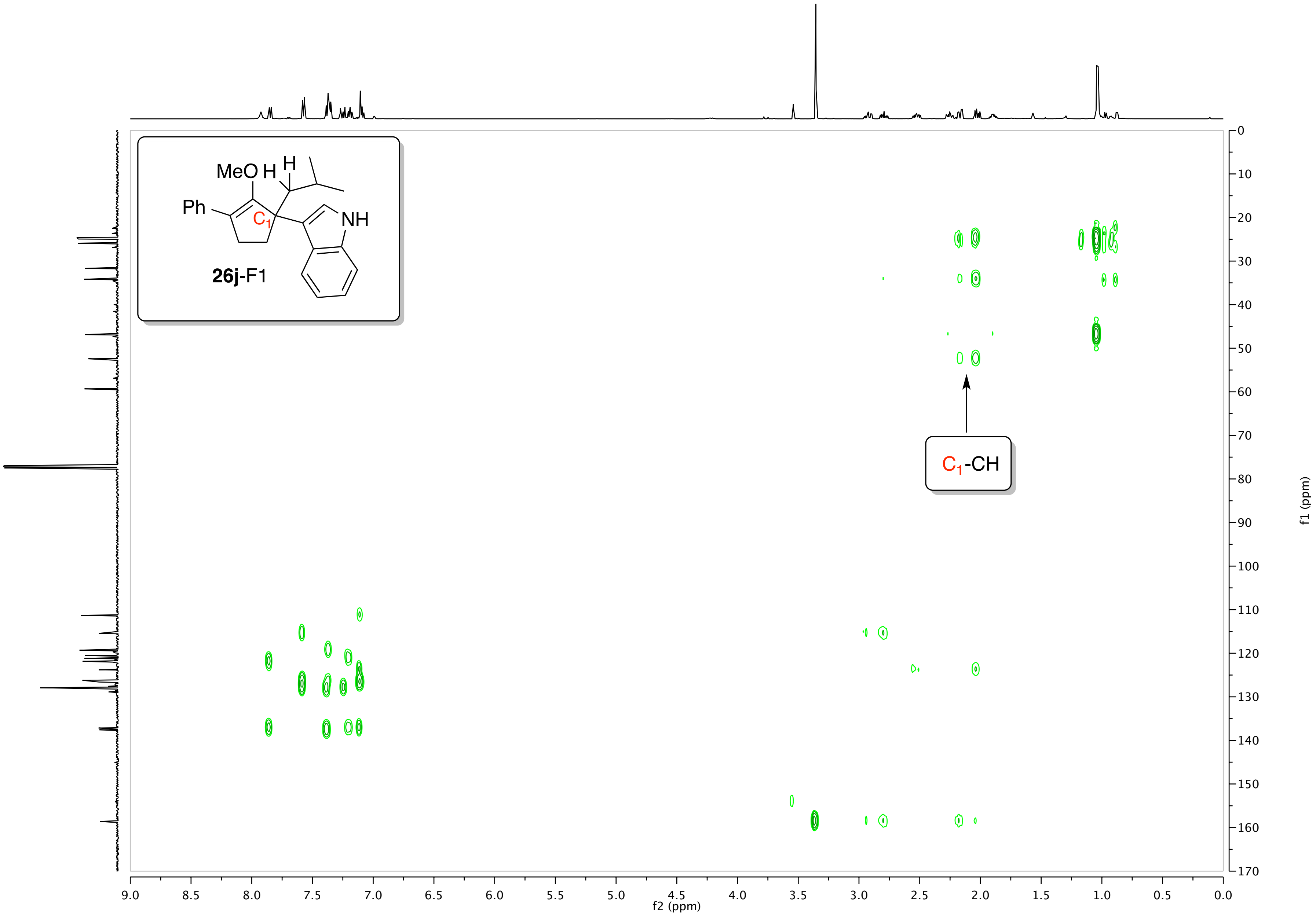
MeO
H
H
Ph
C1
NH
26j-F1
C1-CH
f1 (ppm)
f2 (ppm)

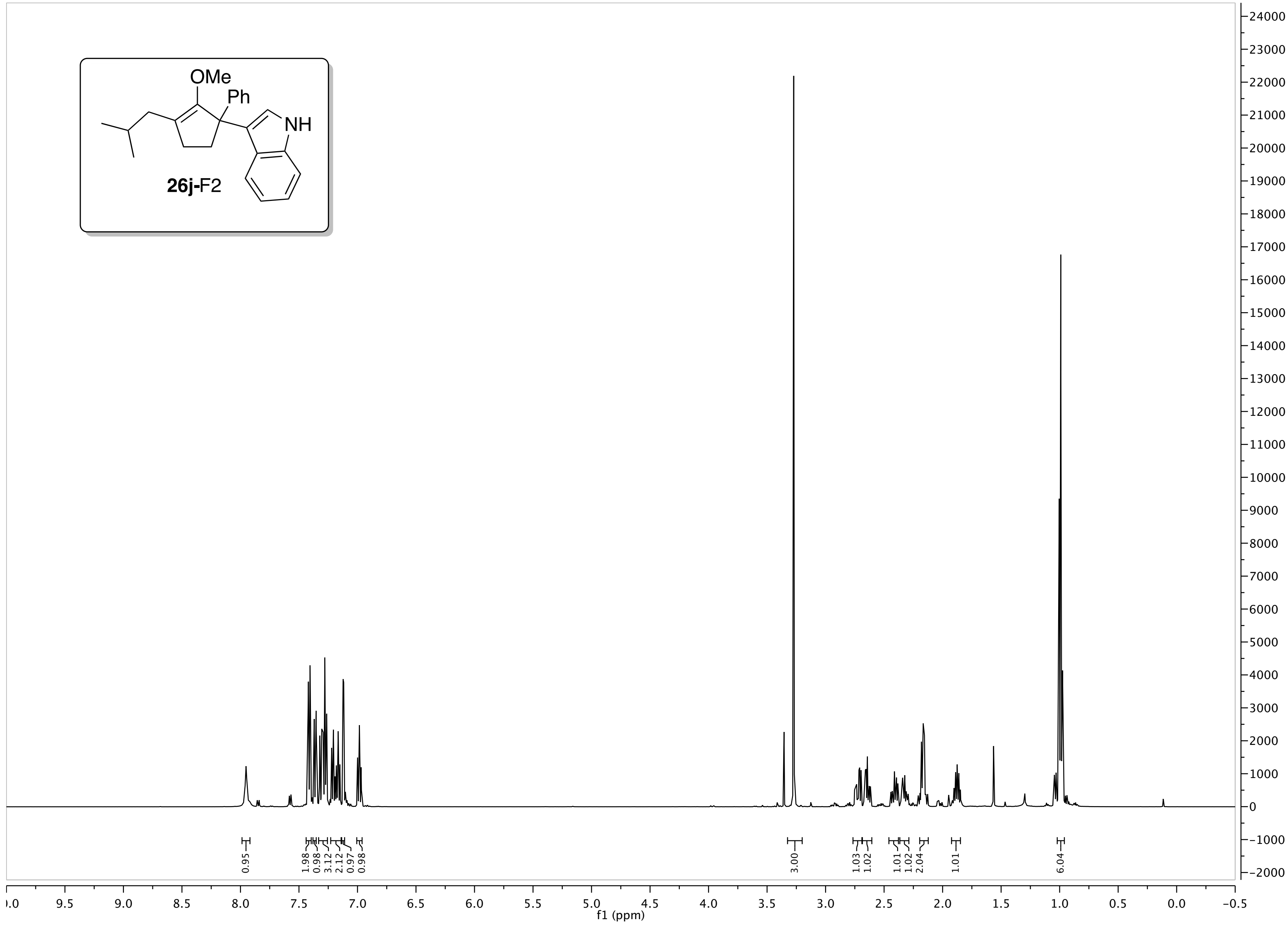
OMe
Ph
NH
26j-F2
f1 (ppm)

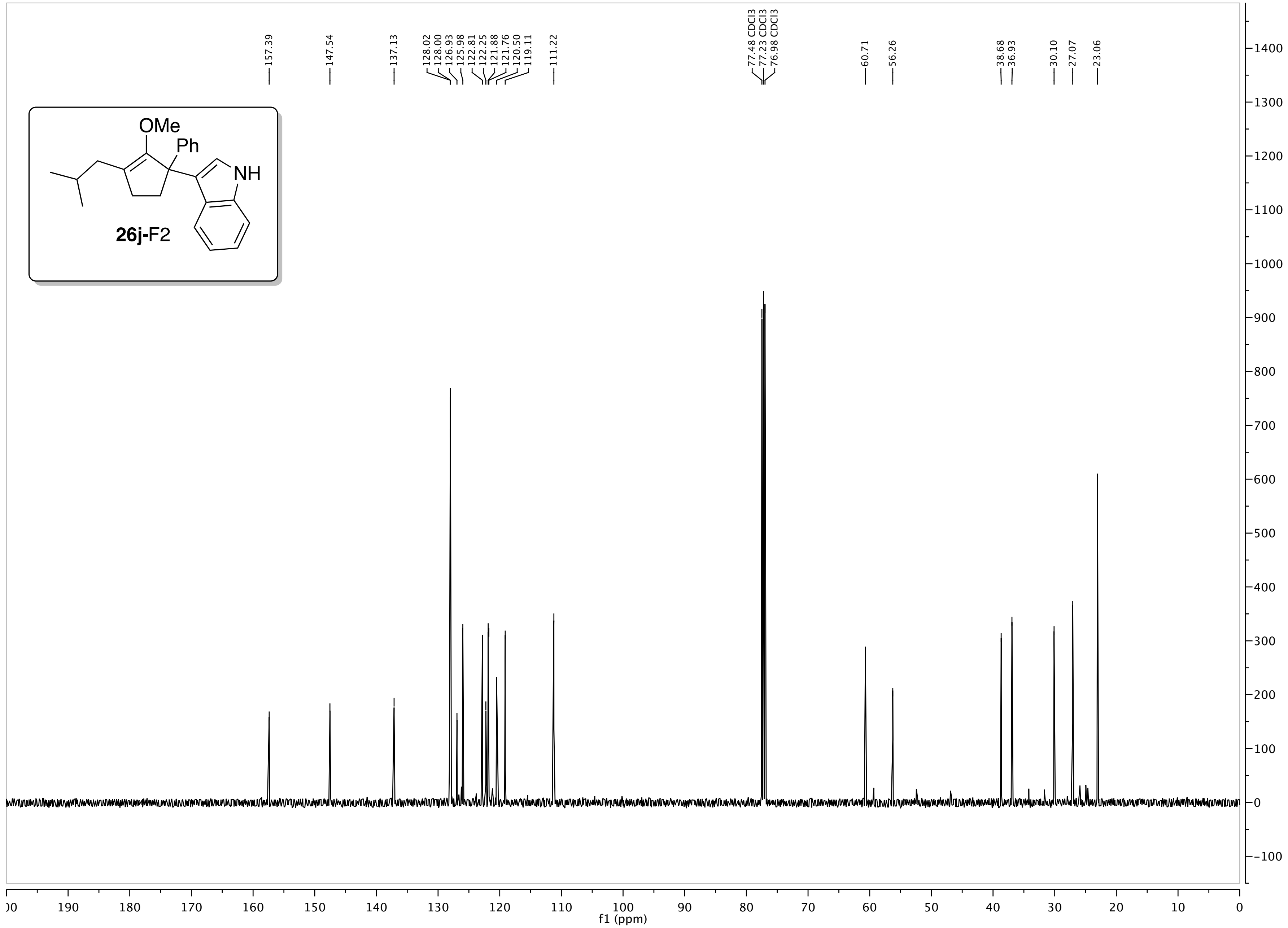

157.39
147.54
137.13
128.02
128.00
126.93
125.98
122.81
122.25
121.88
121.76
120.50
119.11
111.22
77.48 CDCl3
77.23 CDCl3
76.98 CDCl3
60.71
56.26
38.68
36.93
30.10
27.07
23.06
OMe
Ph
NH
26j-F2
f1 (ppm)

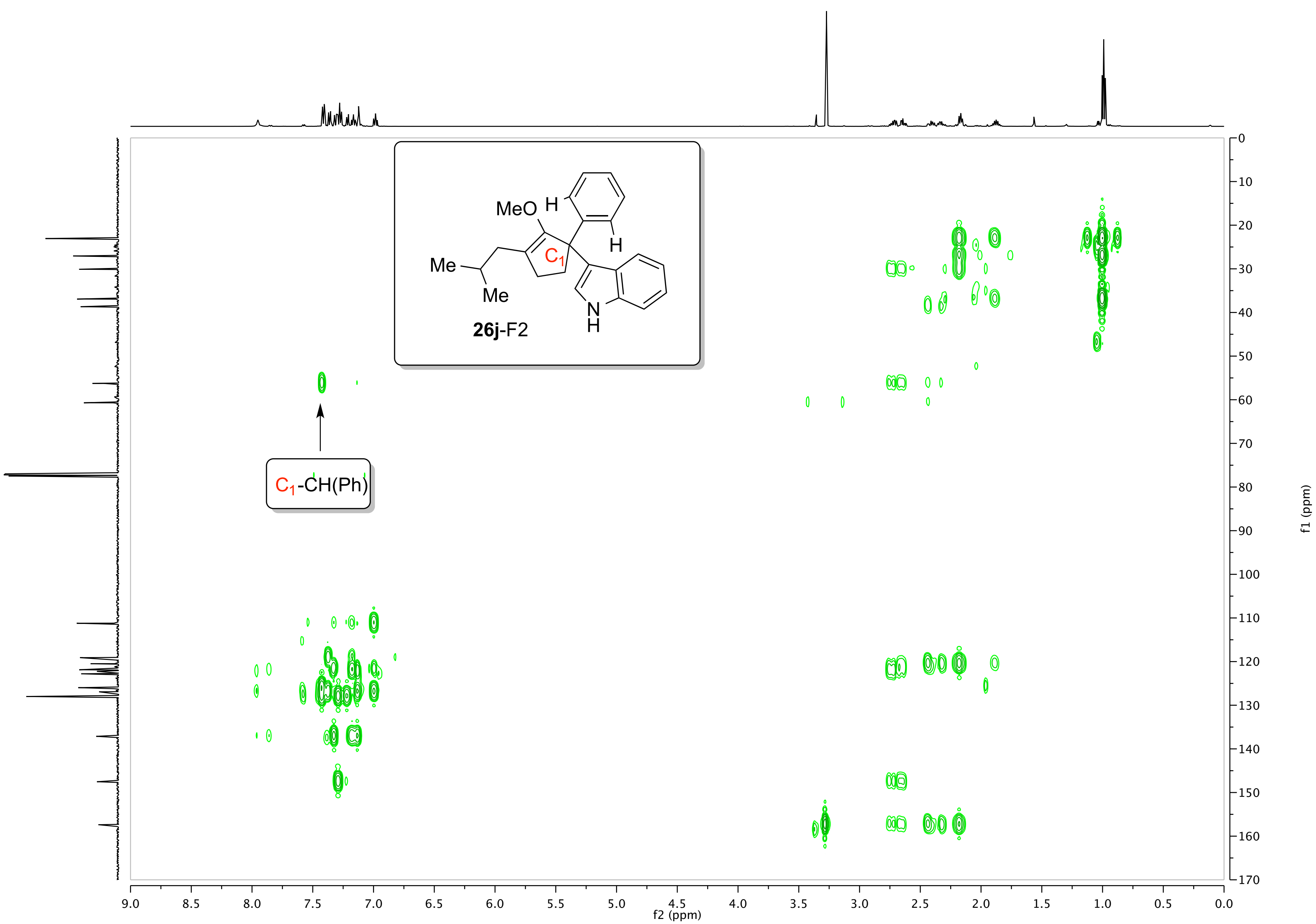
MeO
H
H
Me
Me
C1
N
H
26j-F2
C1-CH(Ph)
f2 (ppm)
f1 (ppm)

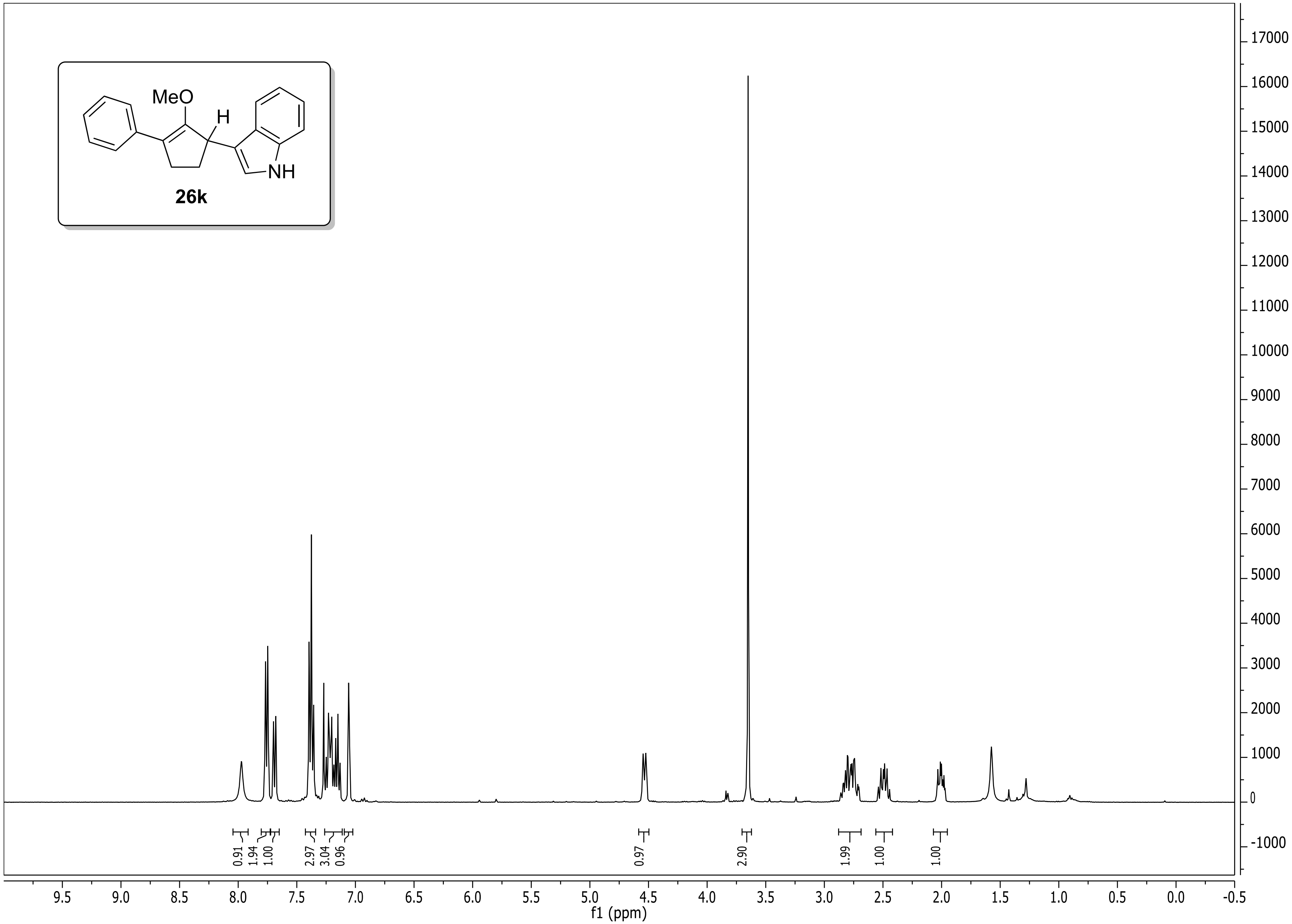

MeO
H
NH
26k
17000
16000
15000
14000
13000
12000
11000
10000
9000
8000
7000
6000
5000
4000
3000
2000
1000
0
-1000
0.91
1.94
1.00
2.97
3.04
0.96
0.97
2.90
1.99
1.00
1.00
9.5 9.0 8.5 8.0 7.5 7.0 6.5 6.0 5.5 5.0 4.5 4.0 3.5 3.0 2.5 2.0 1.5 1.0 0.5 0.0 -0.5
f1 (ppm)

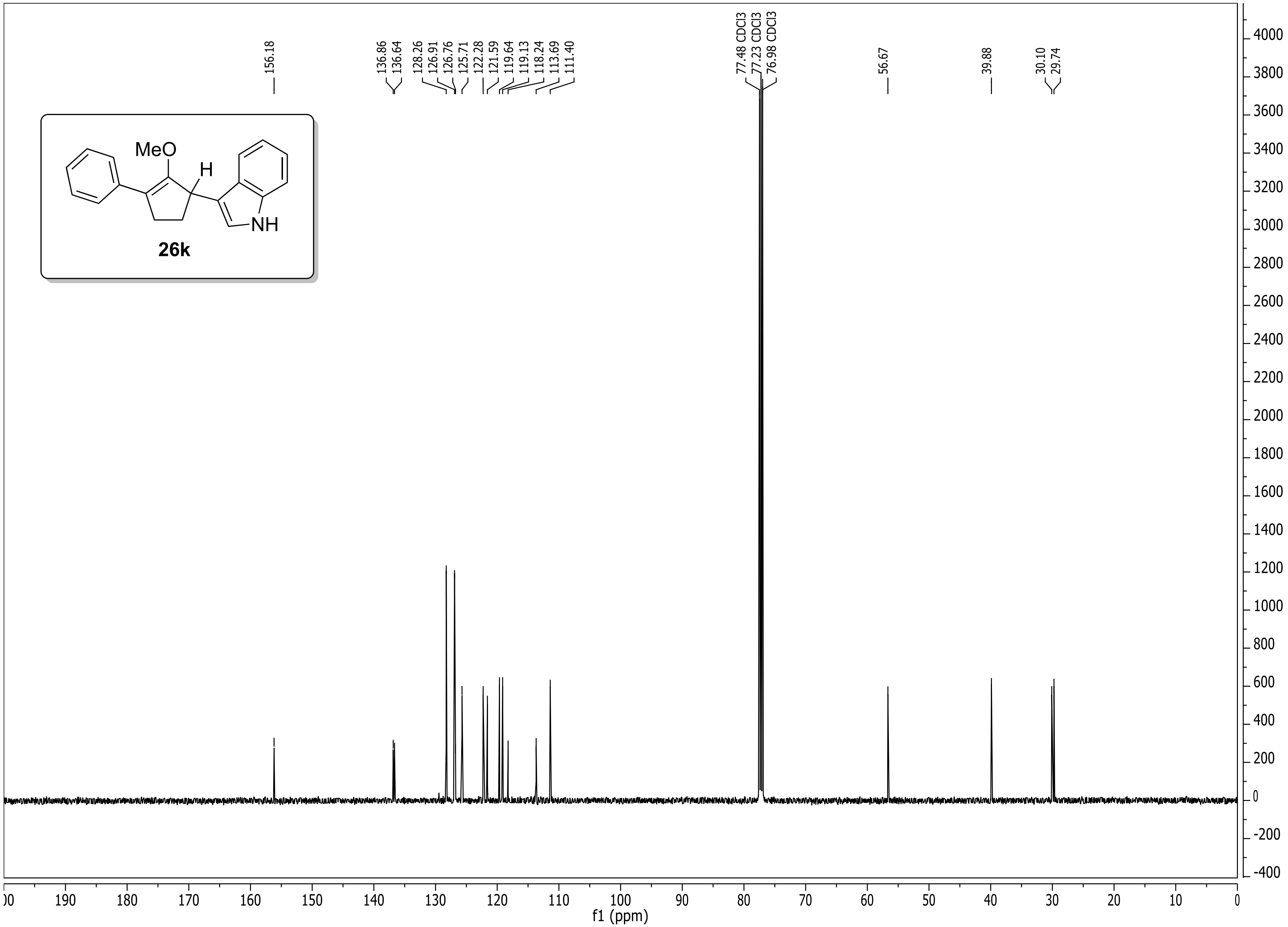
156.18
136.86
136.64
128.26
126.91
126.76
125.71
122.28
121.59
119.64
119.13
118.24
113.69
111.40
77.48 CDCl3
77.23 CDCl3
76.98 CDCl3
56.67
39.88
30.10
29.74
MeO
H
NH
26k
f1 (ppm)

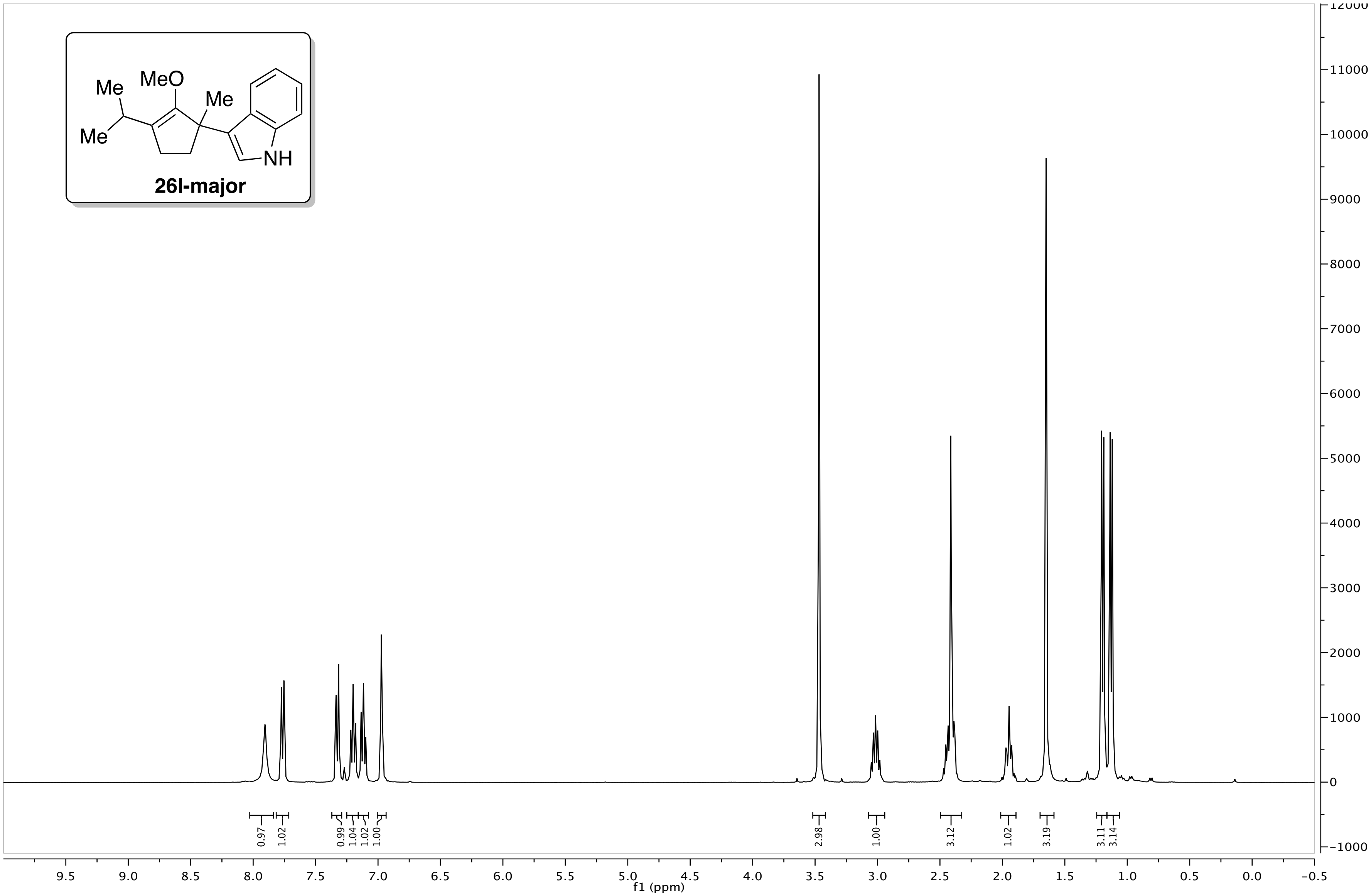
Me
MeO
Me
Me
NH
26l-major
f1 (ppm)

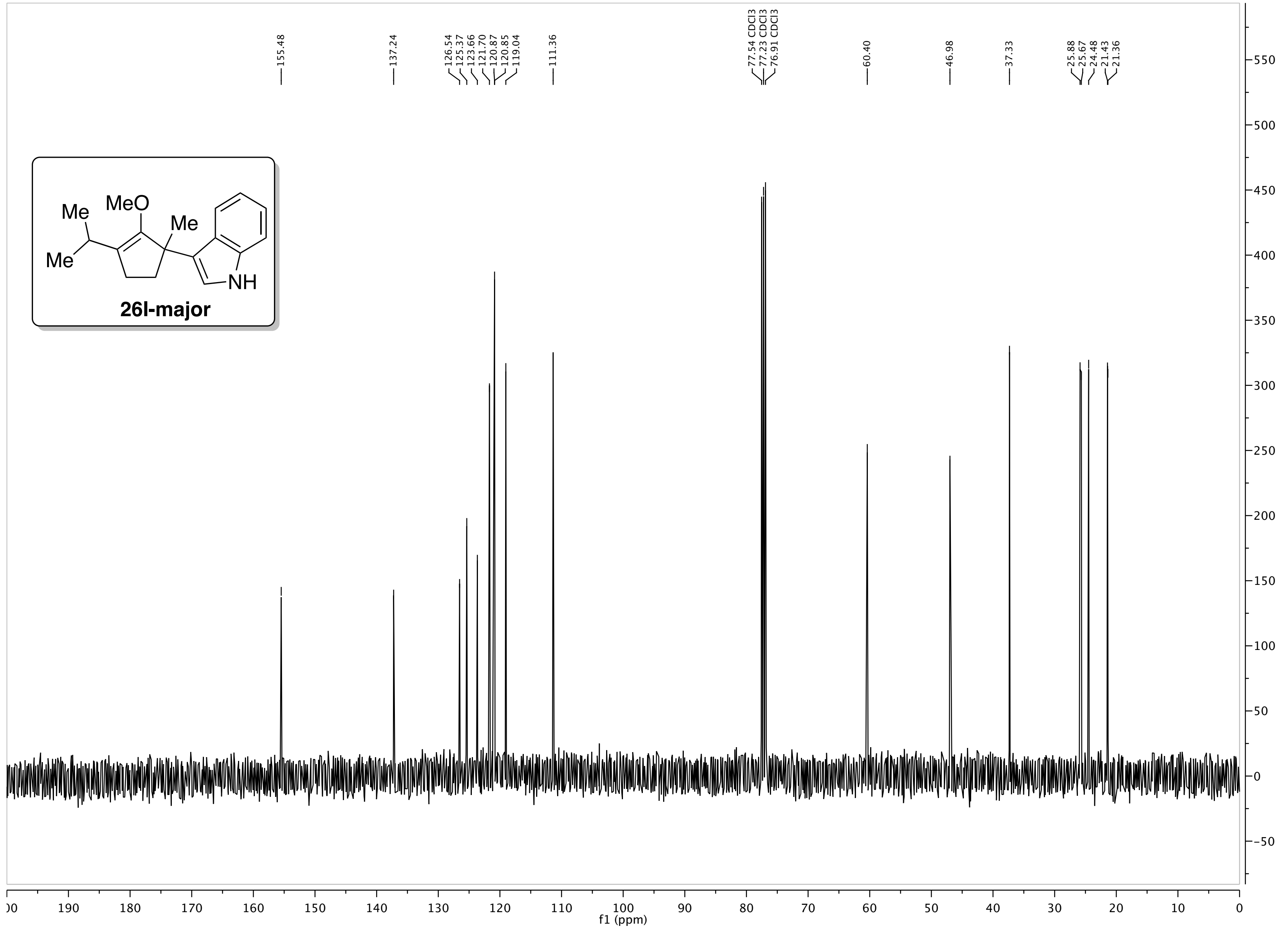

155.48
137.24
126.54
125.37
123.66
121.70
120.87
120.85
119.04
111.36
77.54 CDCl3
77.23 CDCl3
76.91 CDCl3
60.40
46.98
37.33
25.88
25.67
24.48
21.43
21.36
Me
MeO
Me
Me
NH
26l-major
f1 (ppm)

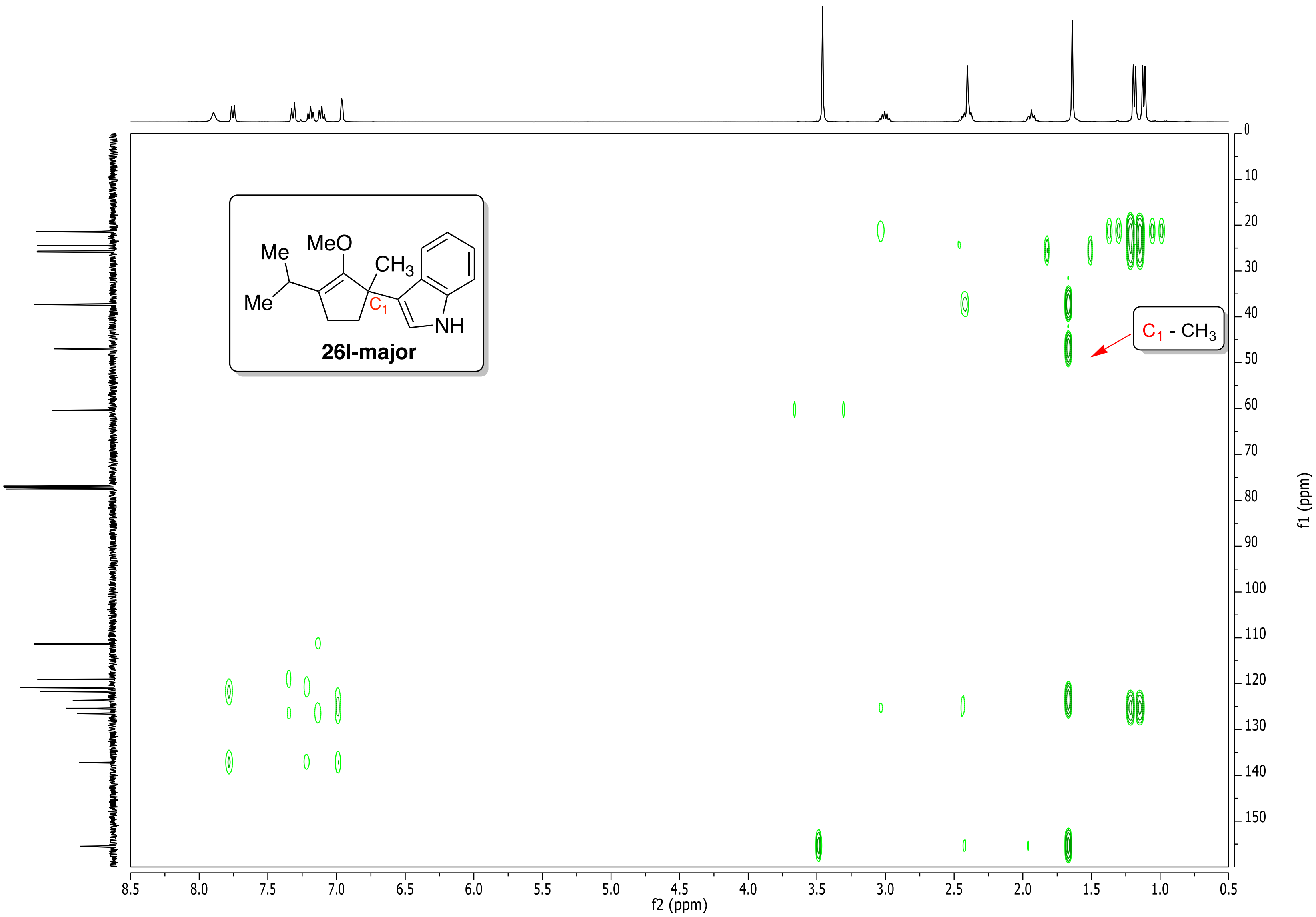
Me
MeO
CH3
Me
C1
NH
26l-major
C1 - CH3
f2 (ppm)
f1 (ppm)

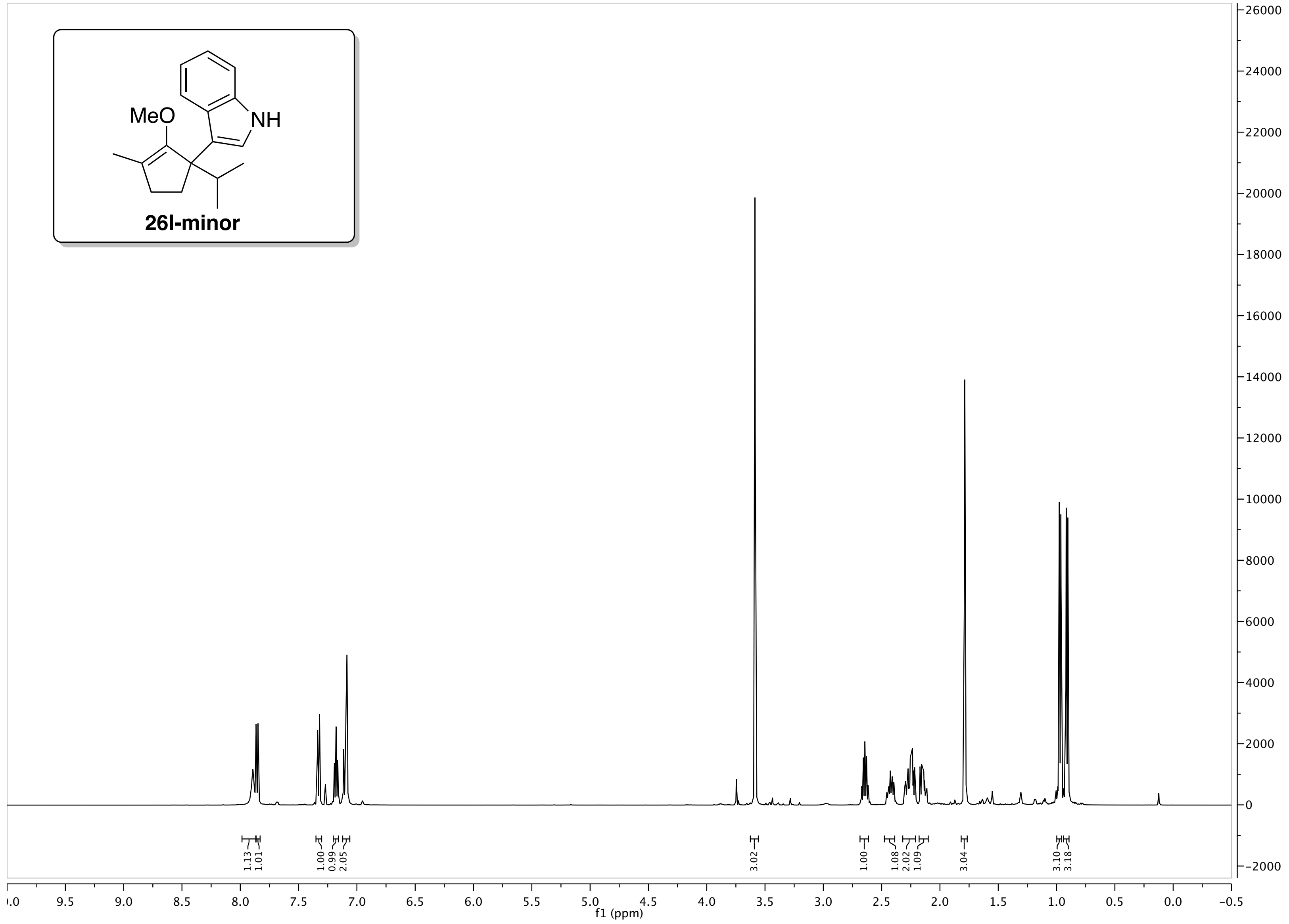
MeO
NH
26l-minor
f1 (ppm)
1.13
1.01
1.00
0.99
2.05
3.02
1.00
1.08
2.02
1.09
3.04
3.10
3.18

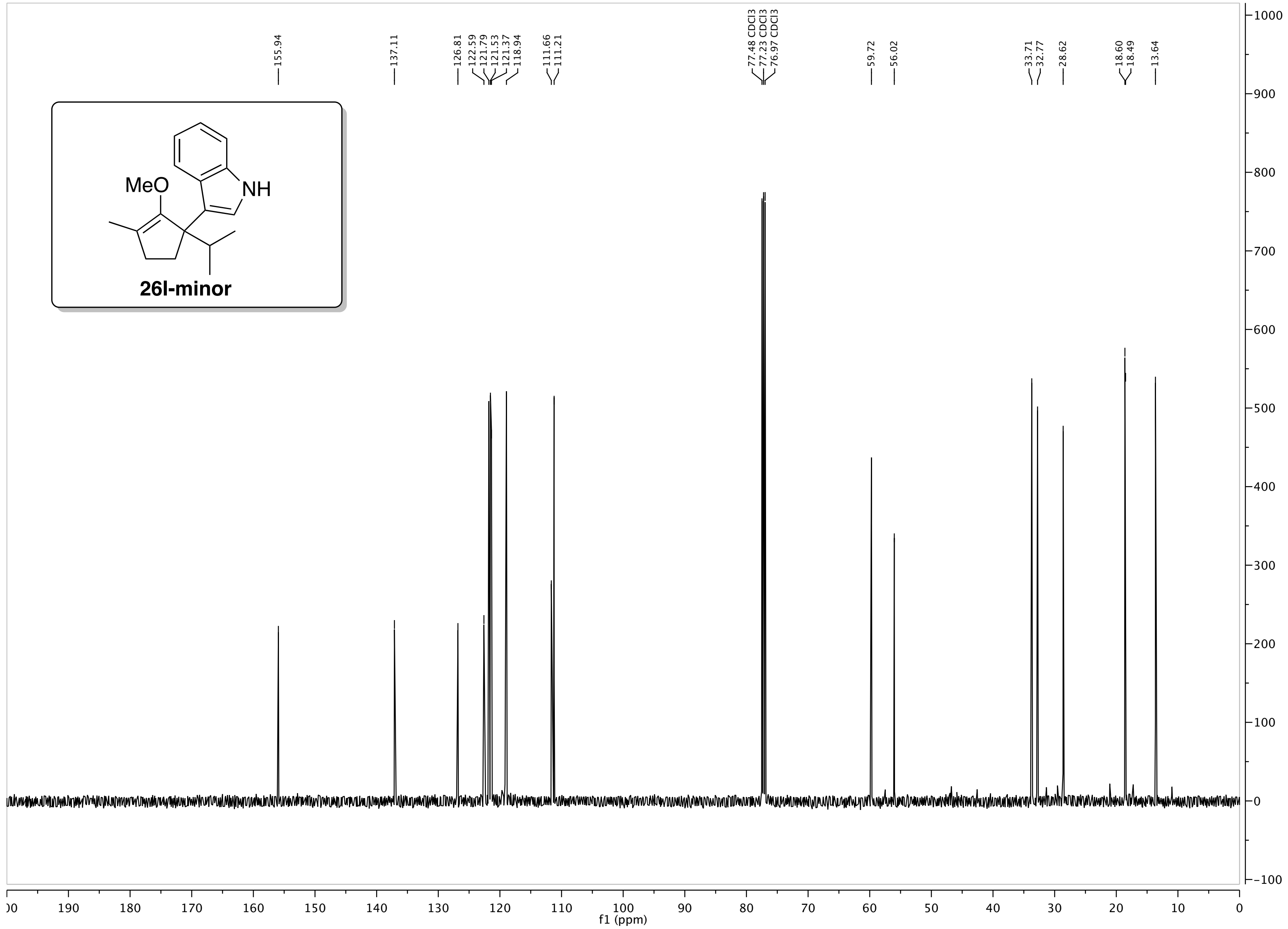
155.94
137.11
126.81
122.59
121.79
121.53
121.37
118.94
111.66
111.21
77.48 CDCl3
77.23 CDCl3
76.97 CDCl3
59.72
56.02
33.71
32.77
28.62
18.60
18.49
13.64
MeO
NH
26l-minor
f1 (ppm)

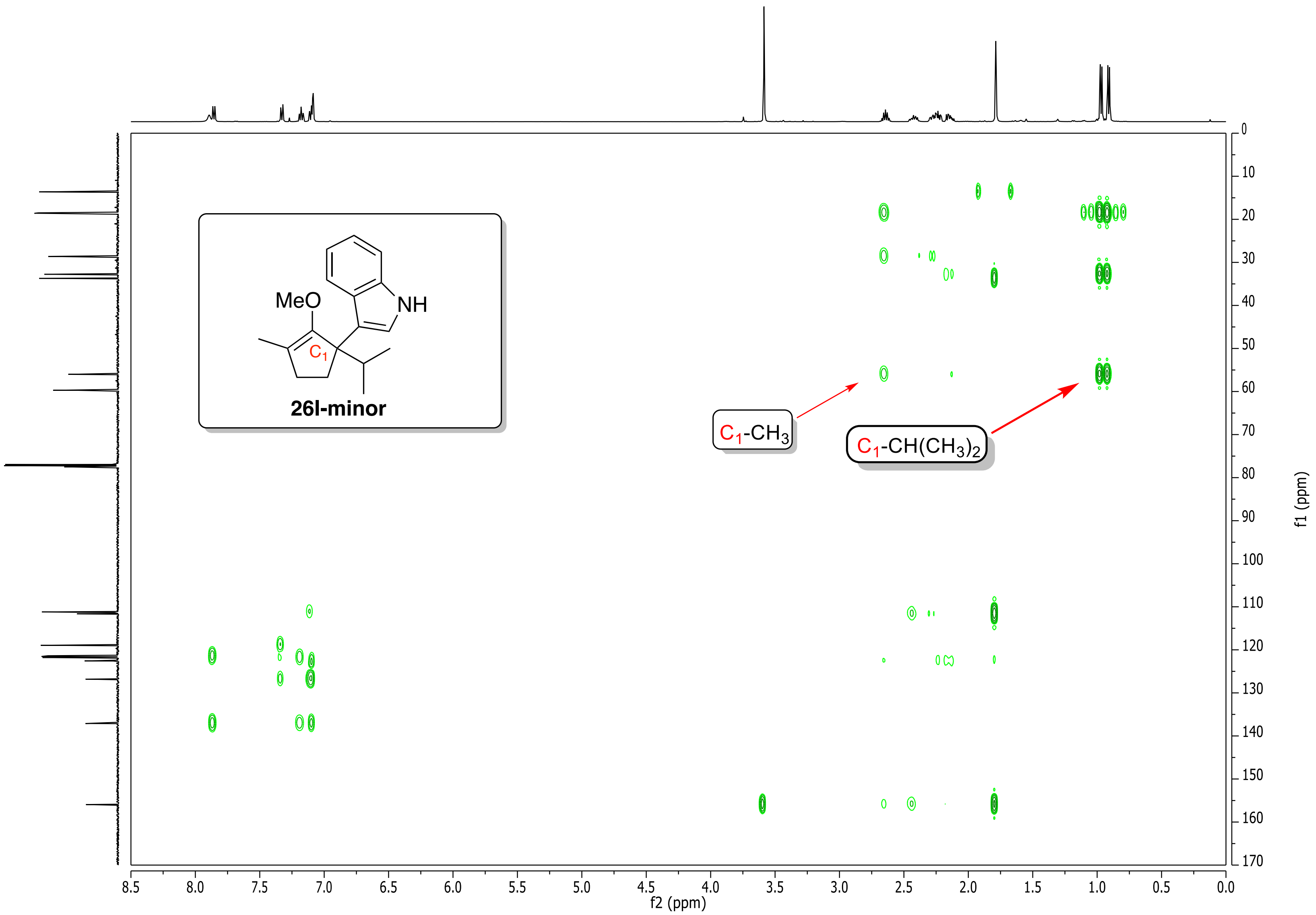
MeO
NH
C1
26l-minor
C1-CH3
C1-CH(CH3)2
f2 (ppm)
f1 (ppm)

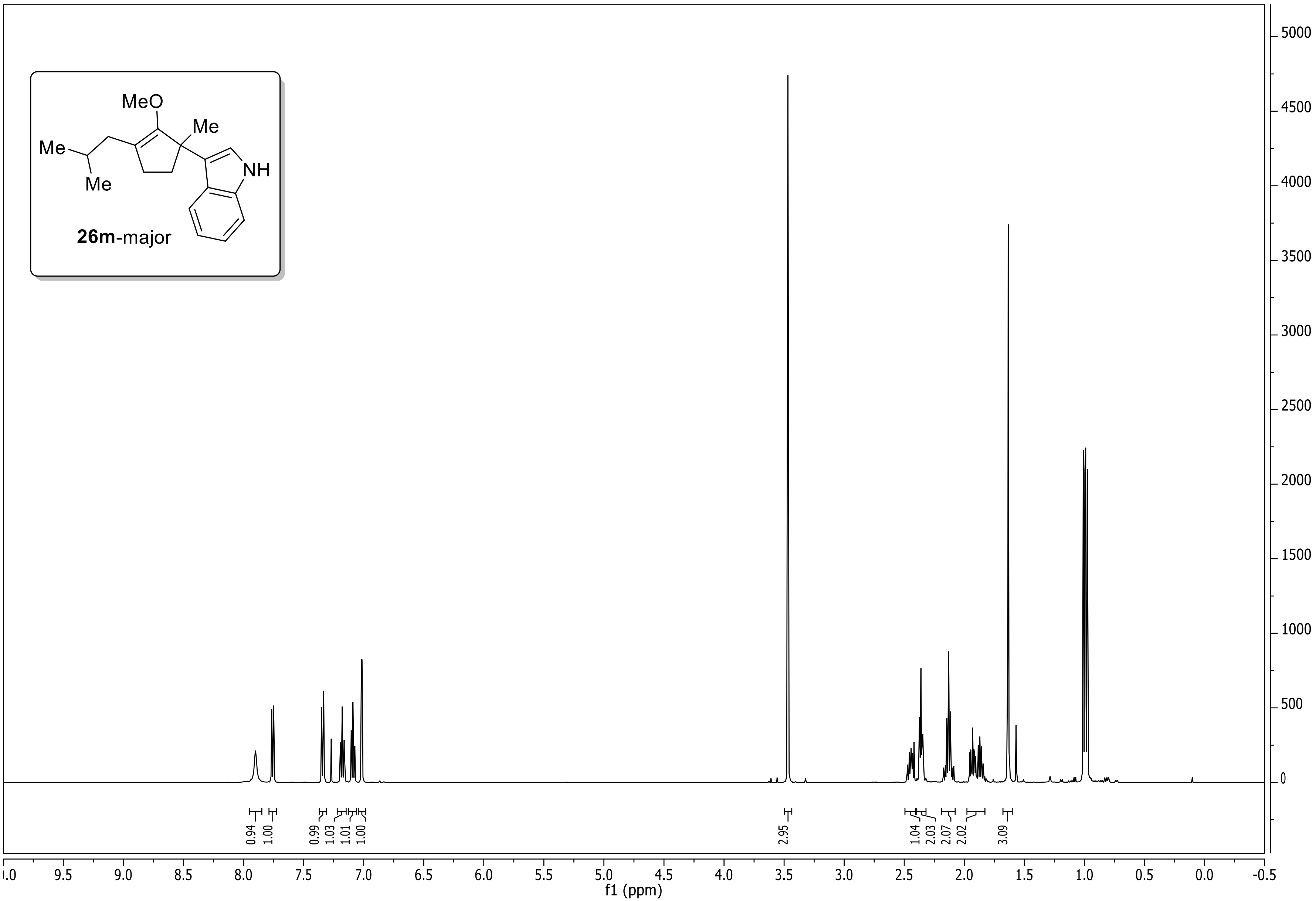
MeO
Me
Me
Me
NH
26m-major
f1 (ppm)

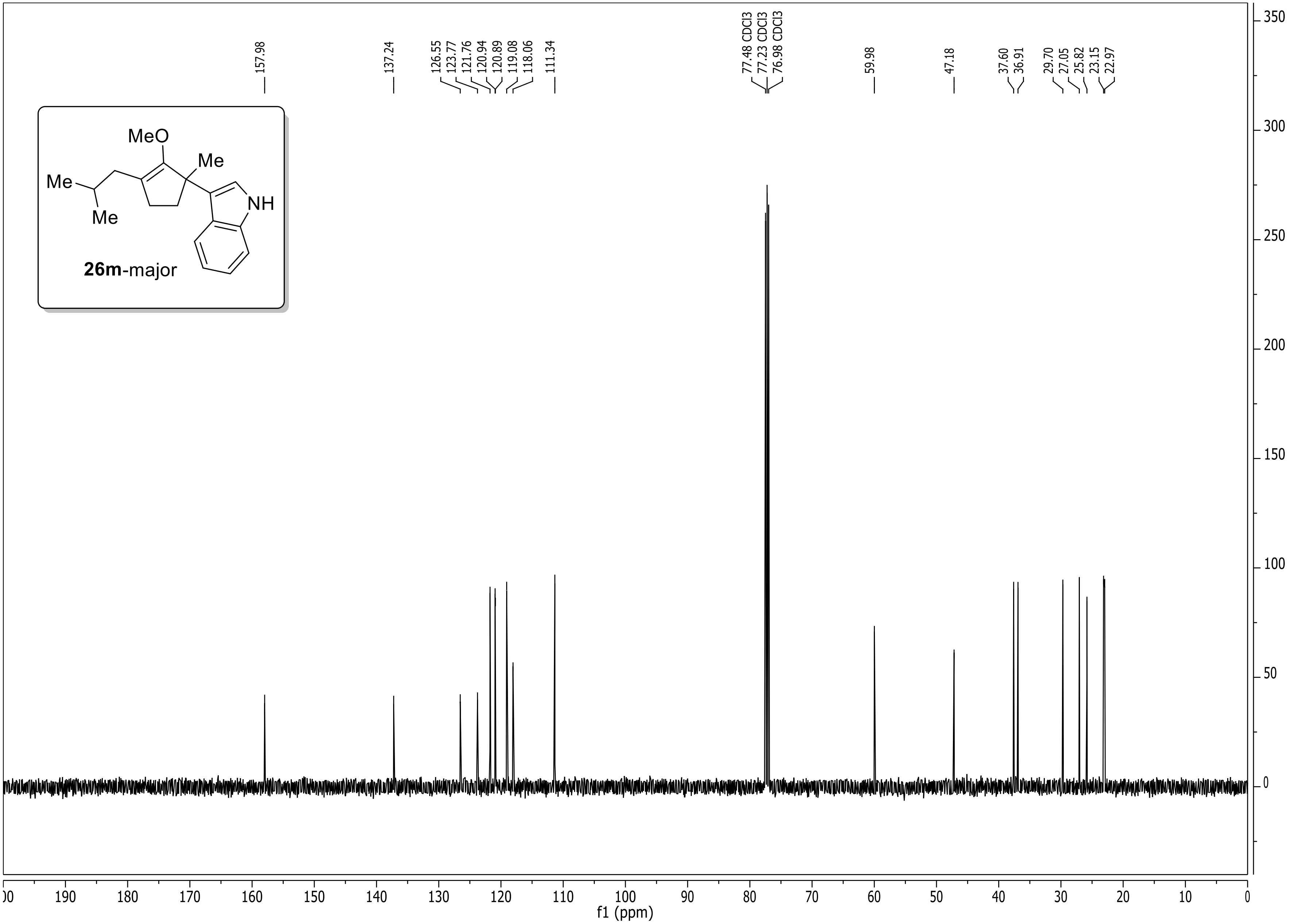

157.98
137.24
126.55
123.77
121.76
120.94
120.89
119.08
118.06
111.34
77.48 CDCl3
77.23 CDCl3
76.98 CDCl3
59.98
47.18
37.60
36.91
29.70
27.05
25.82
23.15
22.97
MeO
Me
Me
Me
NH
26m-major
f1 (ppm)

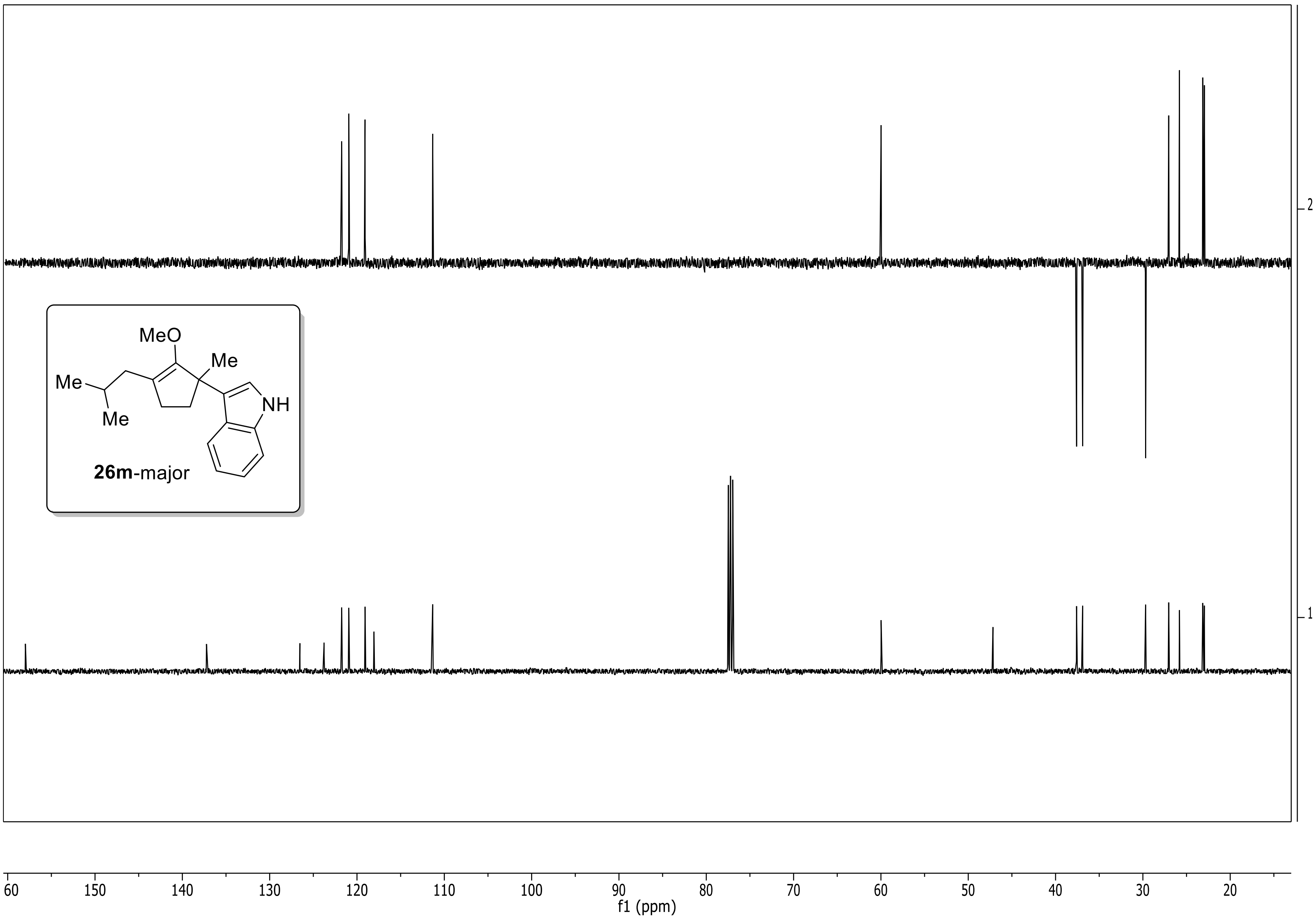
MeO
Me
Me
Me
NH
26m-major
2
1
60
150
140
130
120
110
100
90
80
70
60
50
40
30
20
f1 (ppm)

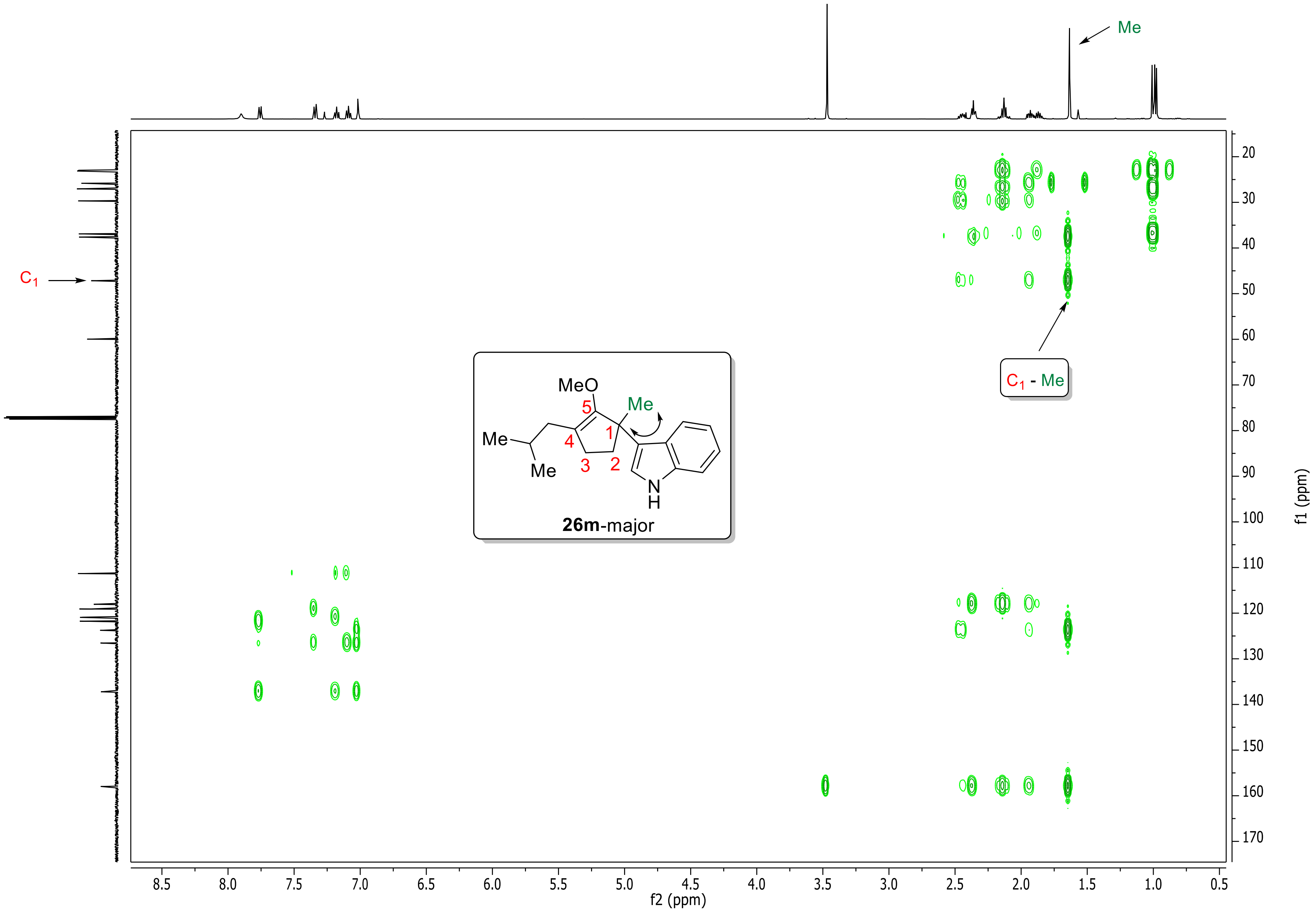
Me
C1
C1 - Me
MeO
Me
Me
Me
N
H
5
1
4
3
2
26m-major
f2 (ppm)
f1 (ppm)

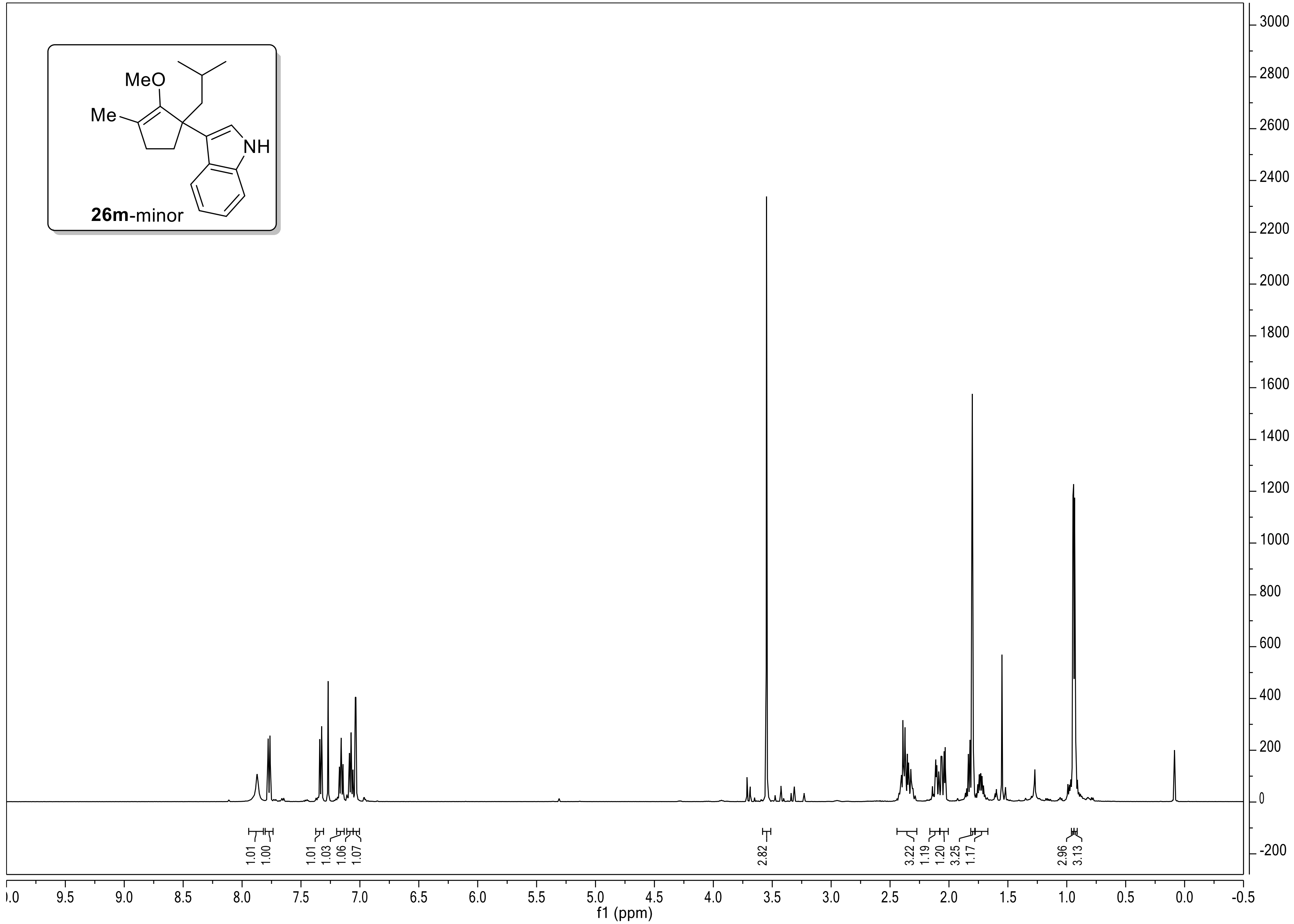

MeO
Me
NH
26m-minor
f1 (ppm)

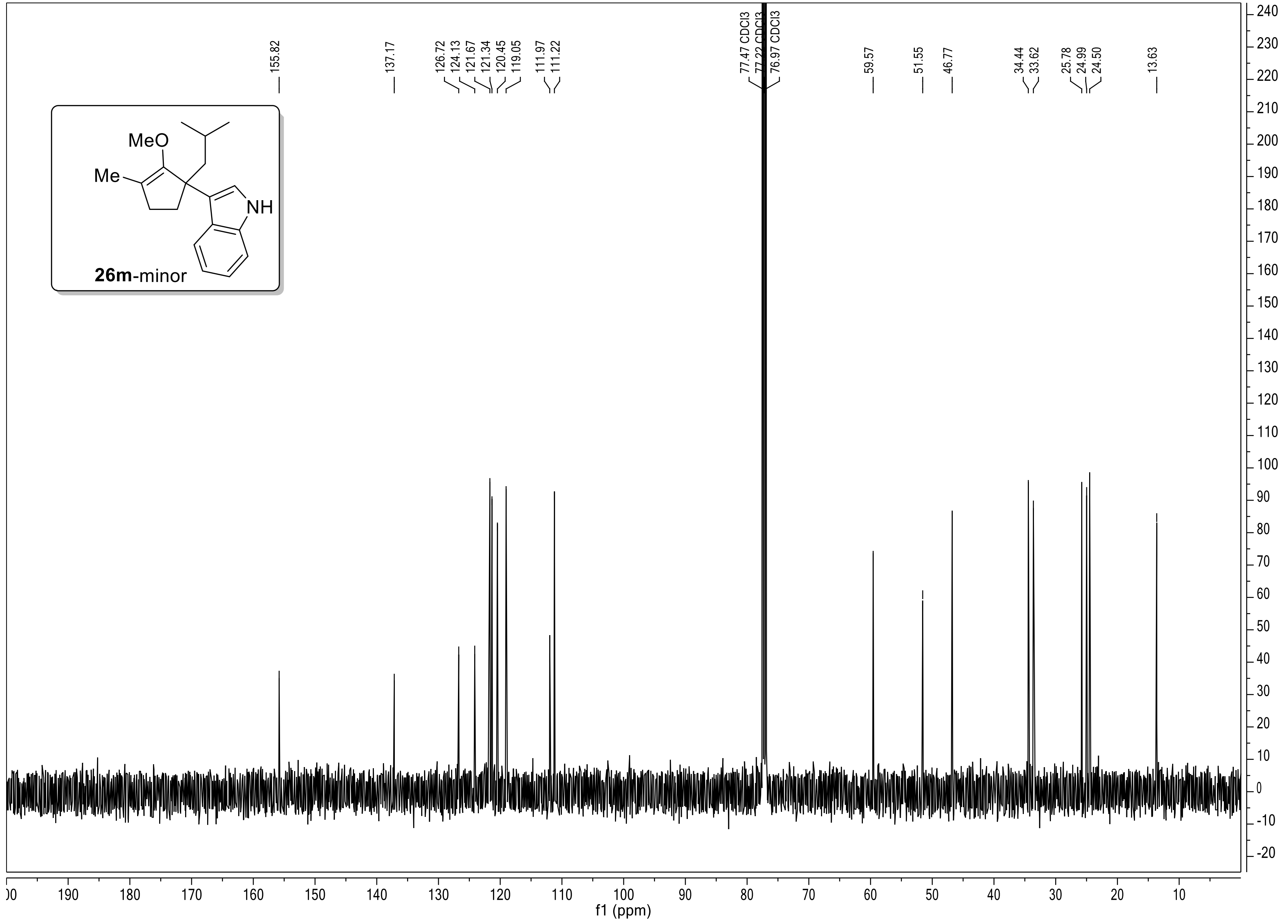
155.82
137.17
126.72
124.13
121.67
121.34
120.45
119.05
111.97
111.22
77.47 CDCl3
77.22 CDCl3
76.97 CDCl3
59.57
51.55
46.77
34.44
33.62
25.78
24.99
24.50
13.63
MeO
Me
NH
26m-minor
f1 (ppm)

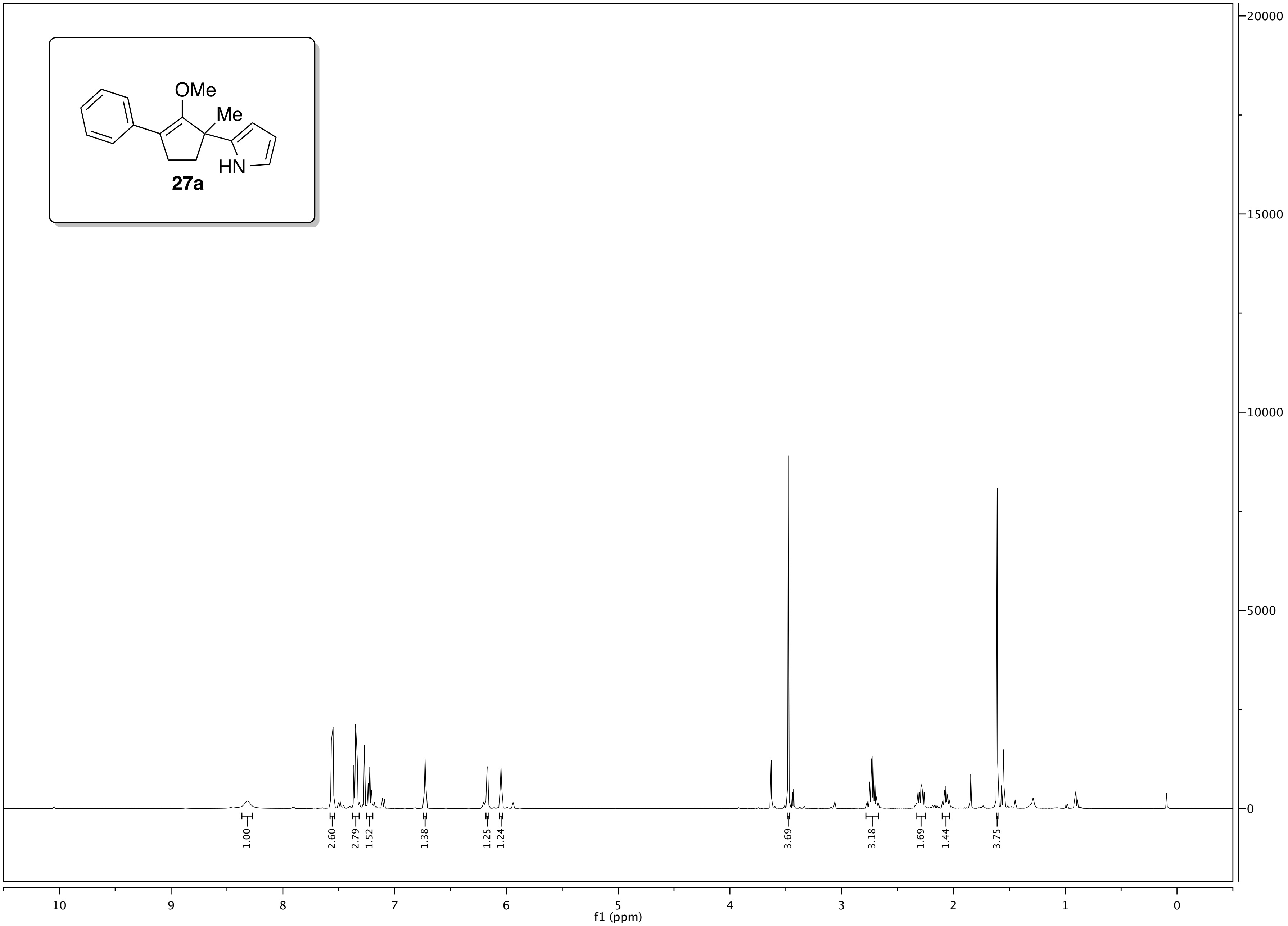

OMe
Me
HN
27a
1.00
2.60
2.79
1.52
1.38
1.25
1.24
3.69
3.18
1.69
1.44
3.75
20000
15000
10000
5000
0
10
9
8
7
6
5
4
3
2
1
0
f1 (ppm)

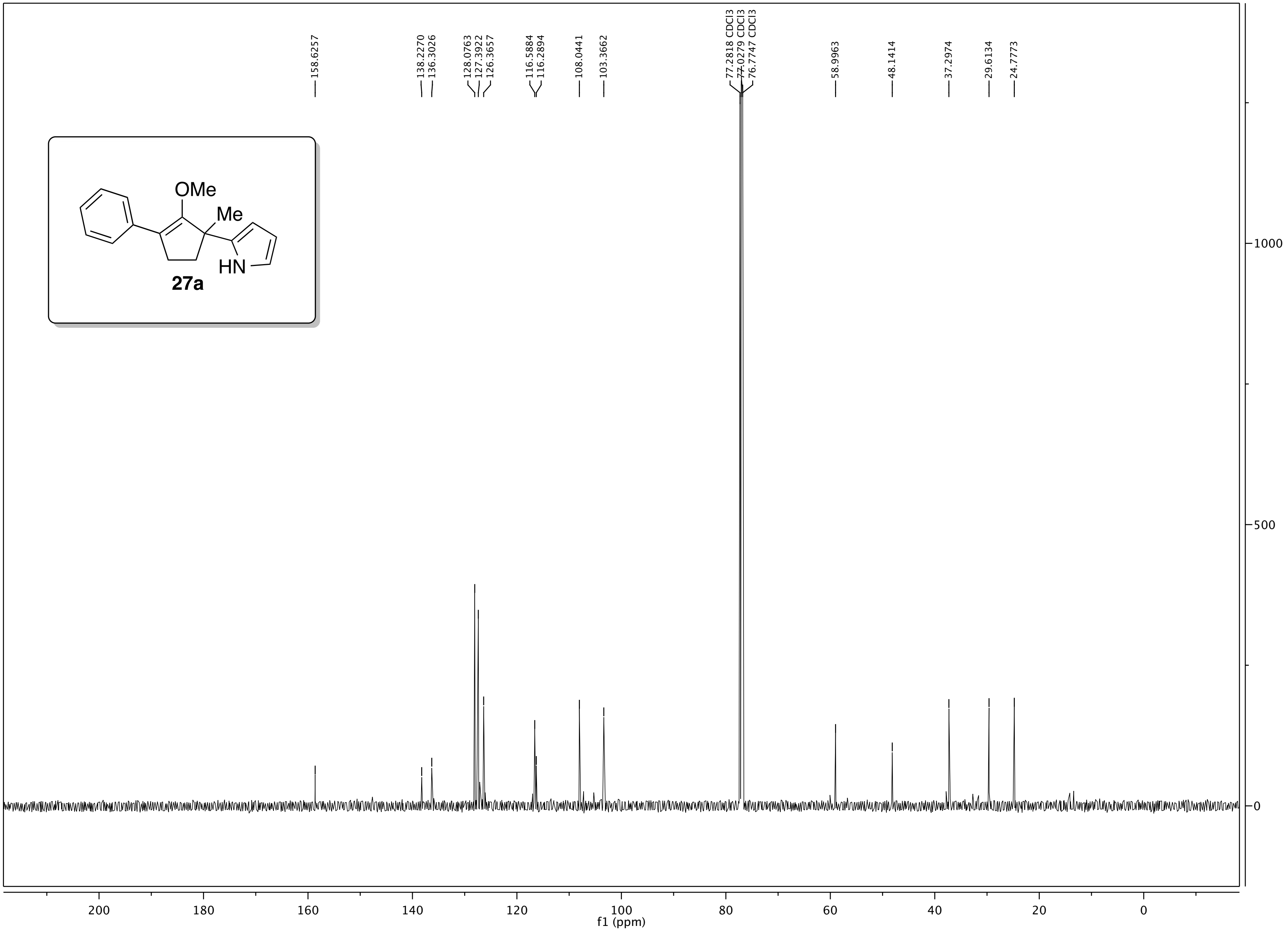
158.6257
138.2270
136.3026
128.0763
127.3922
126.3657
116.5884
116.2894
108.0441
103.3662
77.2818 CDCl3
77.0279 CDCl3
76.7747 CDCl3
58.9963
48.1414
37.2974
29.6134
24.7773
OMe
Me
HN
27a
1000
500
0
200
180
160
140
120
100
80
60
40
20
0
f1 (ppm)

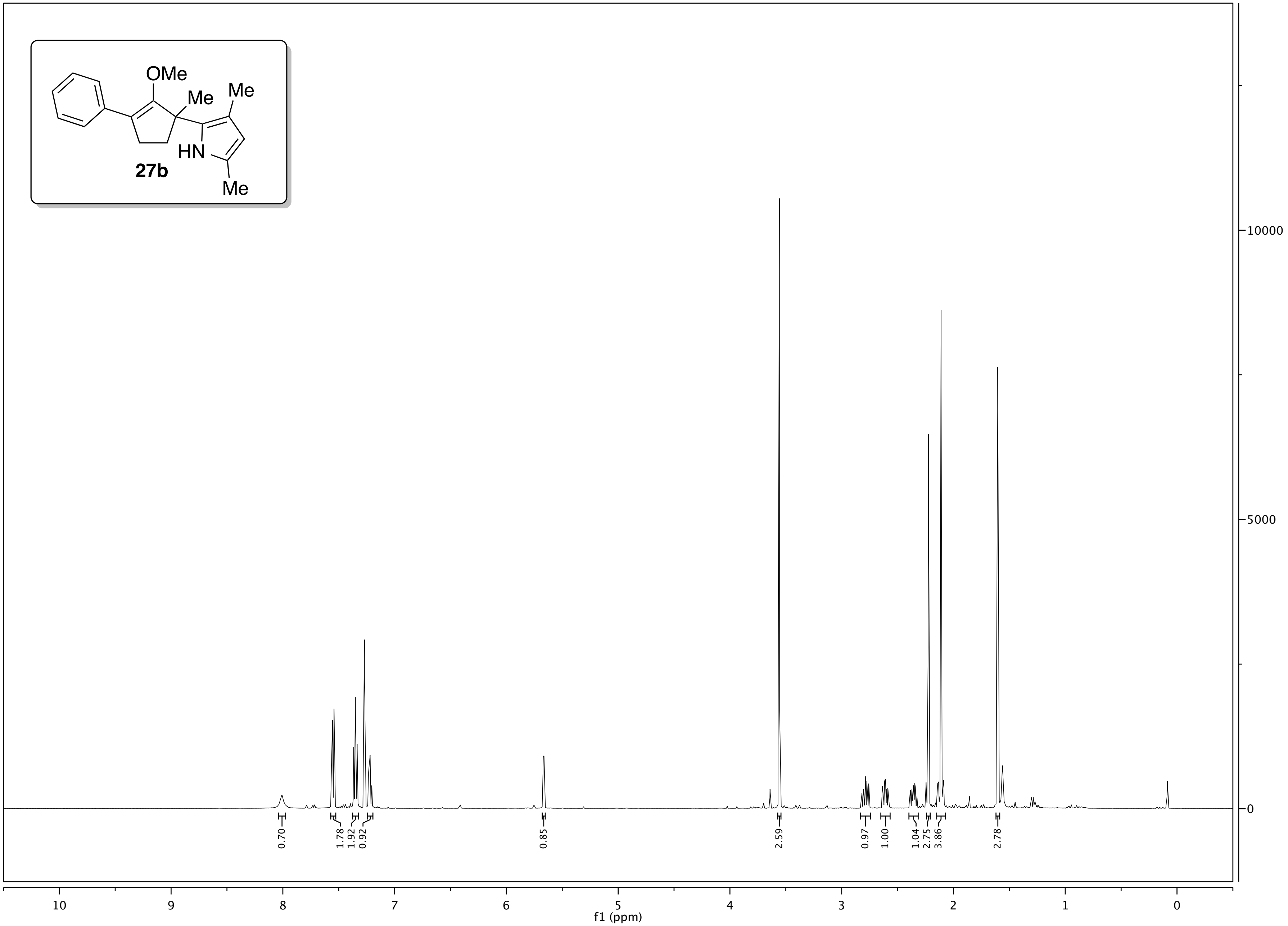
OMe
Me
Me
HN
Me
27b
0.70
1.78
1.92
0.92
0.85
2.59
0.97
1.00
1.04
2.75
3.86
2.78
10000
5000
0
10
9
8
7
6
5
4
3
2
1
0
f1 (ppm)

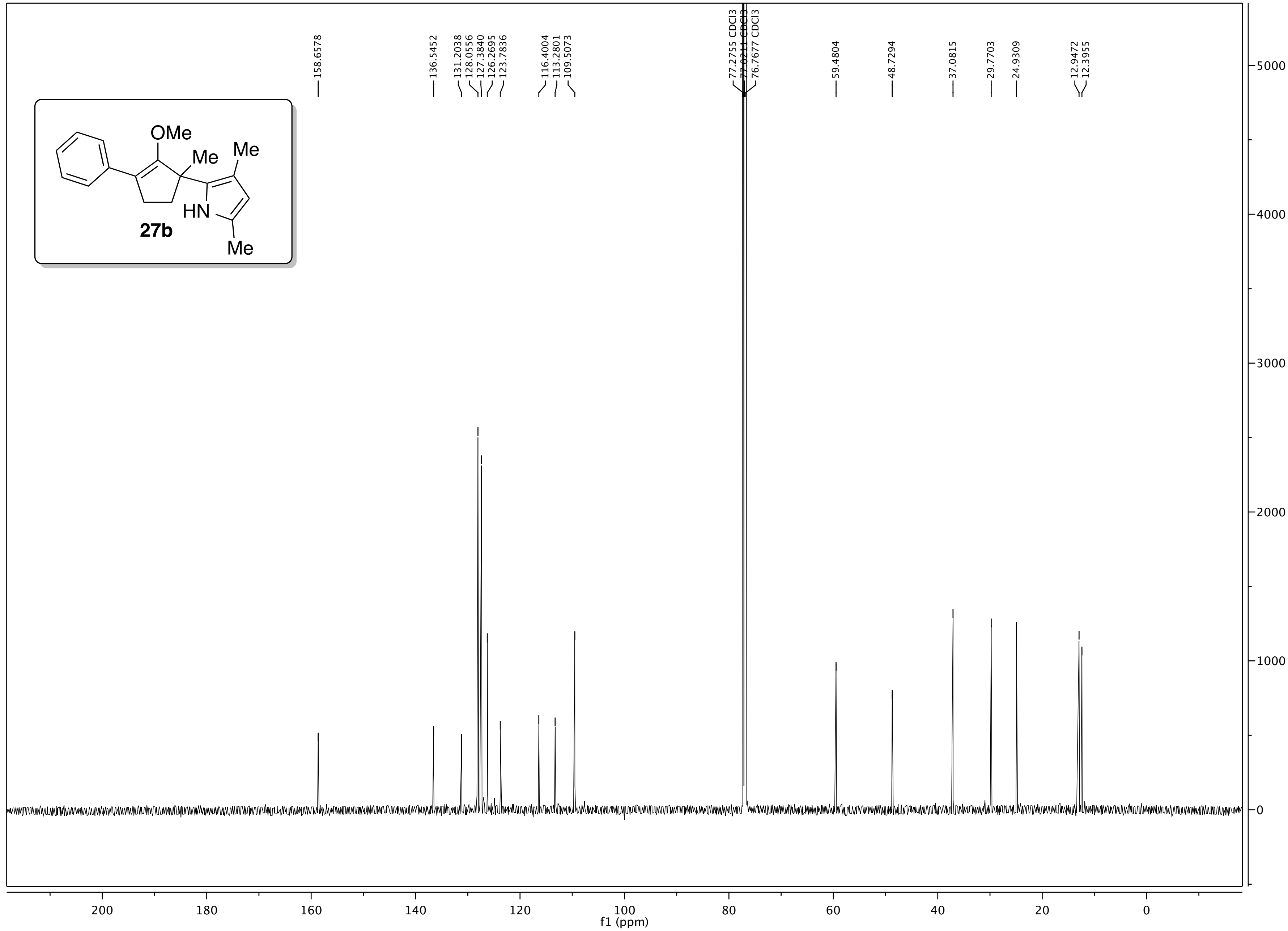

158.6578
136.5452
131.2038
128.0556
127.3840
126.2695
123.7836
116.4004
113.2801
109.5073
77.2755 CDCl3
77.0211 CDCl3
76.7677 CDCl3
59.4804
48.7294
37.0815
29.7703
24.9309
12.9472
12.3955
OMe
Me
Me
HN
Me
27b
f1 (ppm)

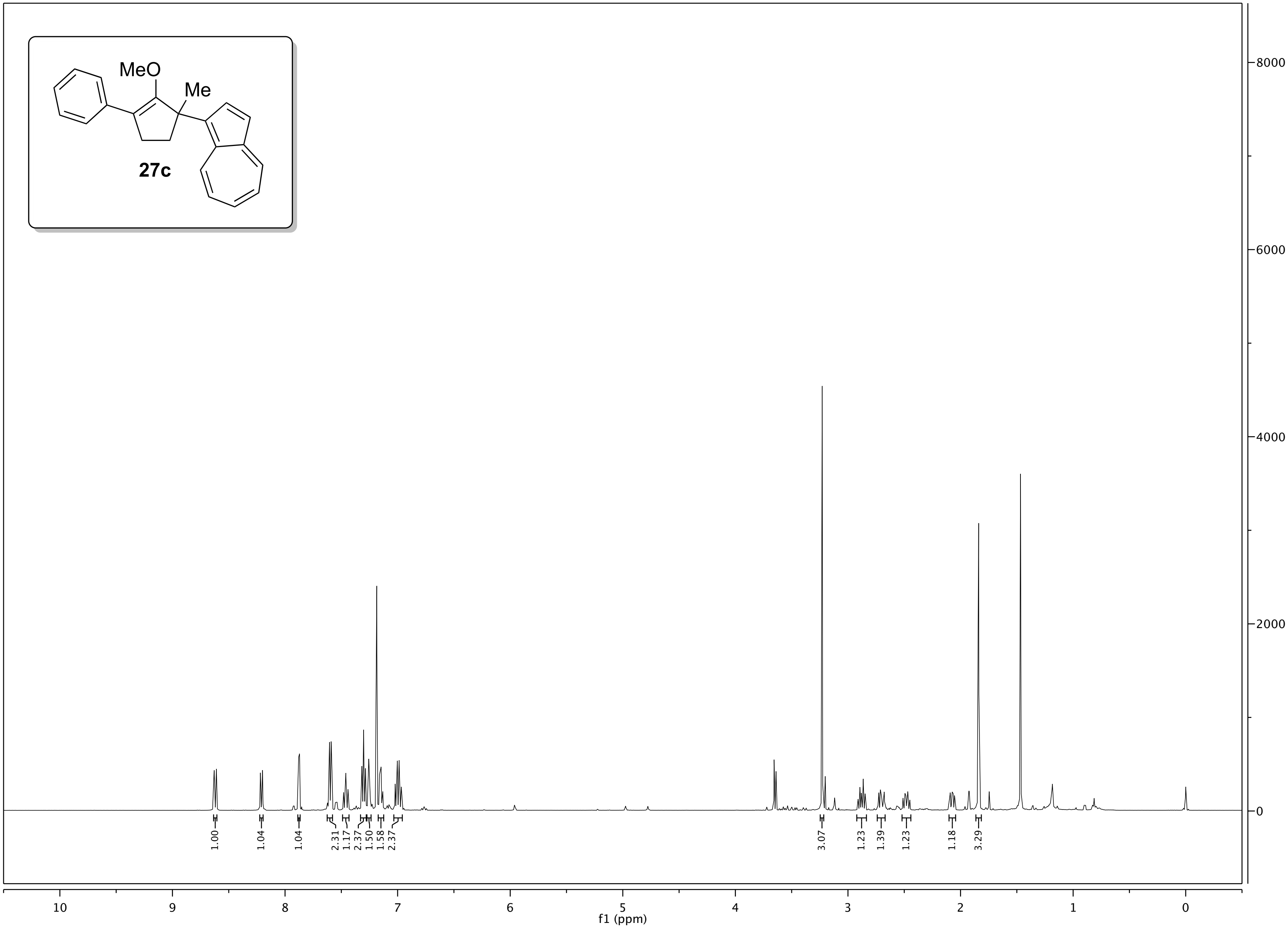
MeO
Me
27c
f1 (ppm)

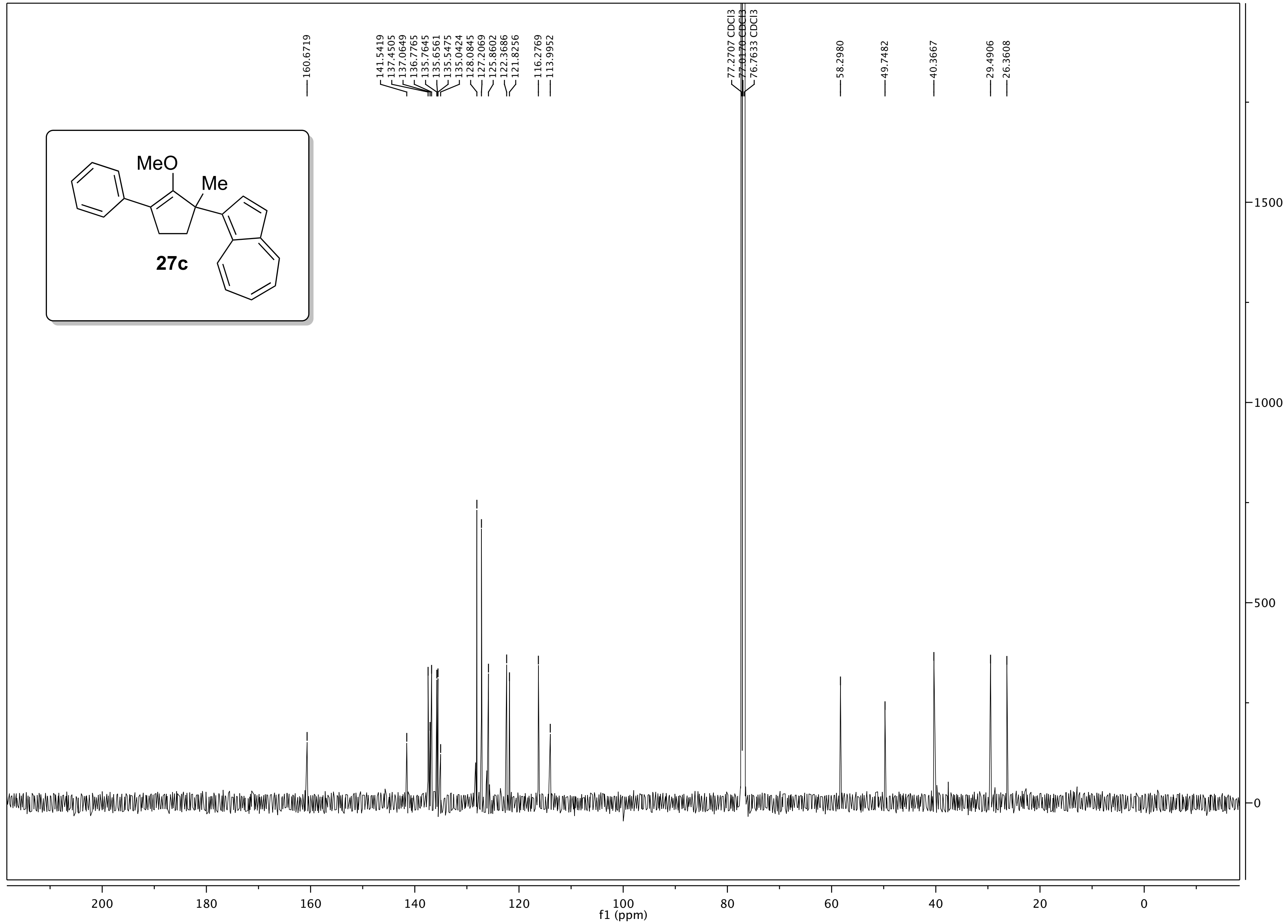

160.6719
141.5419
137.4505
137.0649
136.7765
135.7645
135.6561
135.5475
135.0424
128.0845
127.2069
125.8602
122.3686
121.8256
116.2769
113.9952
77.2707 CDCl3
77.0170 CDCl3
76.7633 CDCl3
58.2980
49.7482
40.3667
29.4906
26.3608
MeO
Me
27c
1500
1000
500
0
200
180
160
140
120
100
80
60
40
20
0
f1 (ppm)

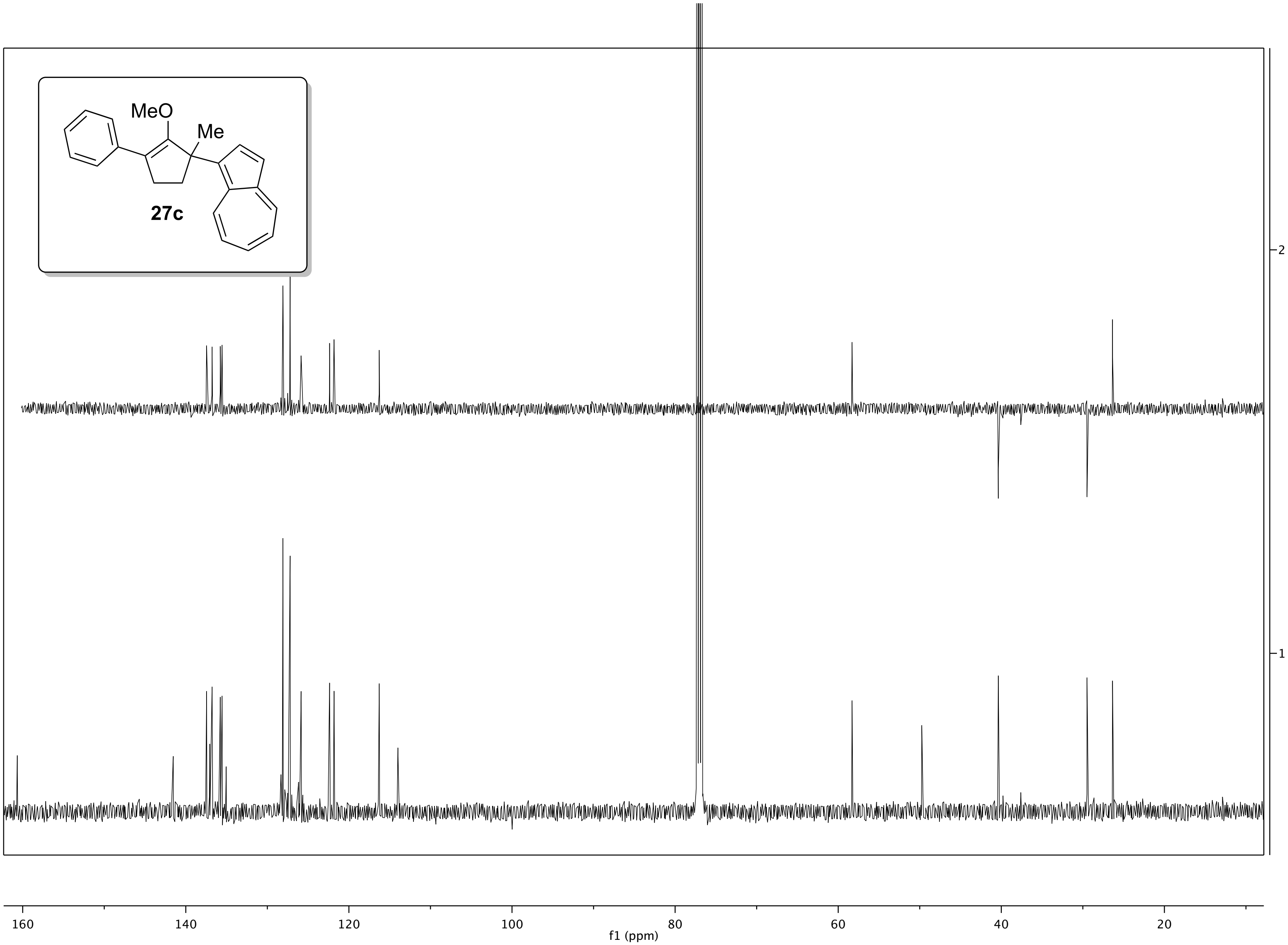
MeO
Me
27c
f1 (ppm)
160
140
120
100
80
60
40
20
2
1

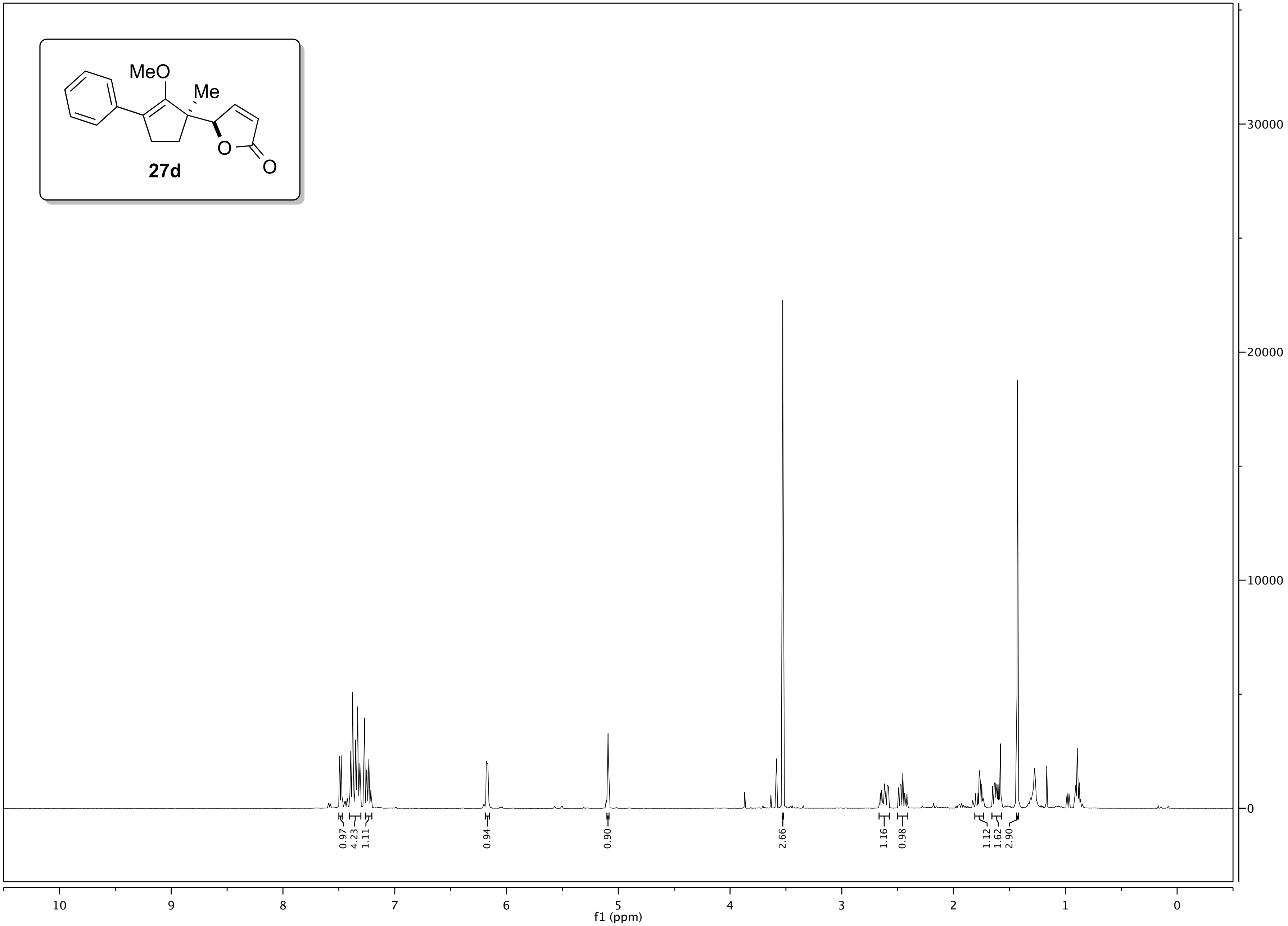
MeO
Me
O
O
27d
0.97
4.23
1.11
0.94
0.90
2.66
1.16
0.98
1.12
1.62
2.90
30000
20000
10000
0
10
9
8
7
6
5
4
3
2
1
0
f1 (ppm)

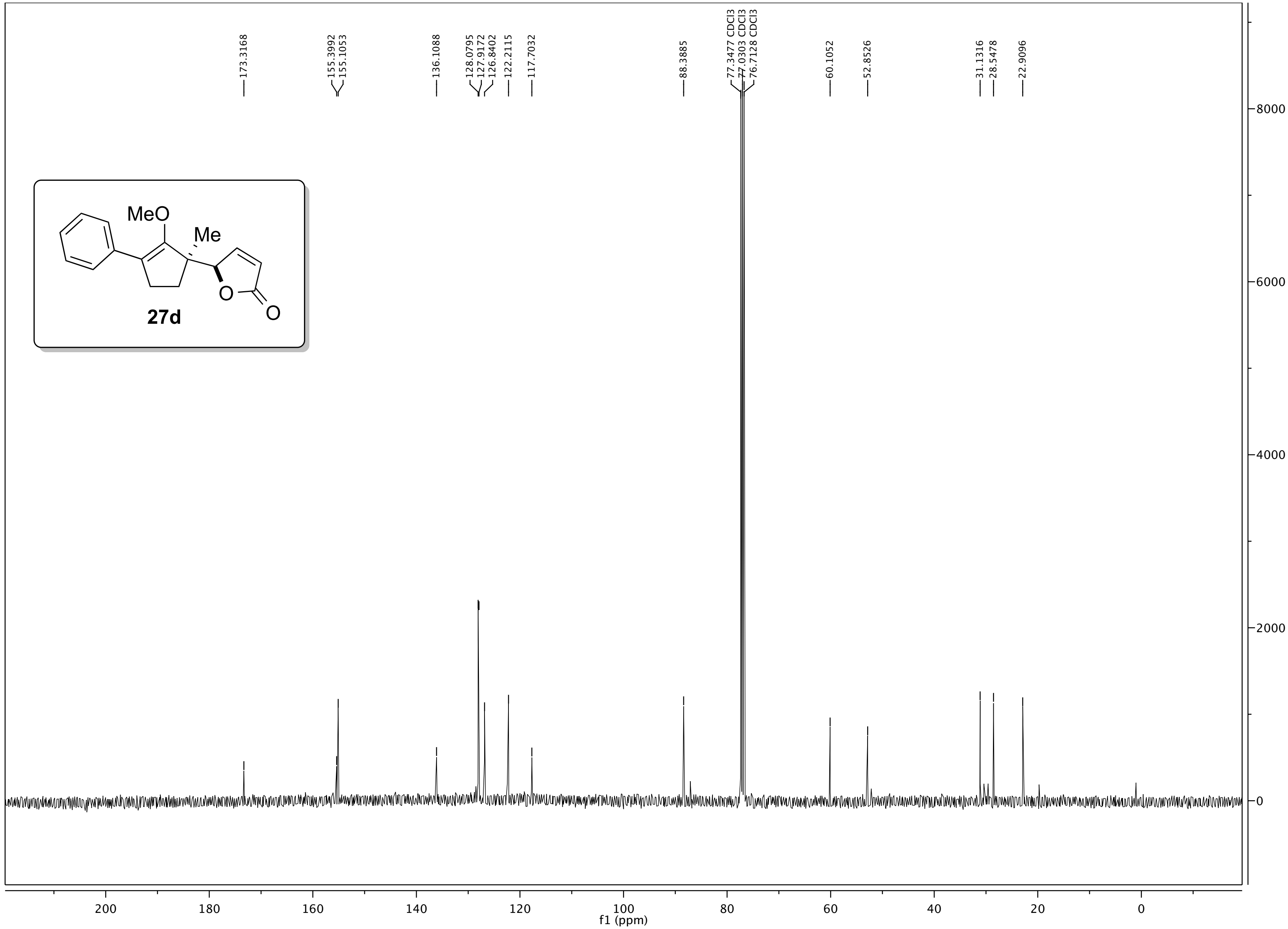
173.3168
155.3992
155.1053
136.1088
128.0795
127.9172
126.8402
122.2115
117.7032
88.3885
77.3477 CDCl3
77.0303 CDCl3
76.7128 CDCl3
60.1052
52.8526
31.1316
28.5478
22.9096
MeO
Me
O
O
27d
f1 (ppm)

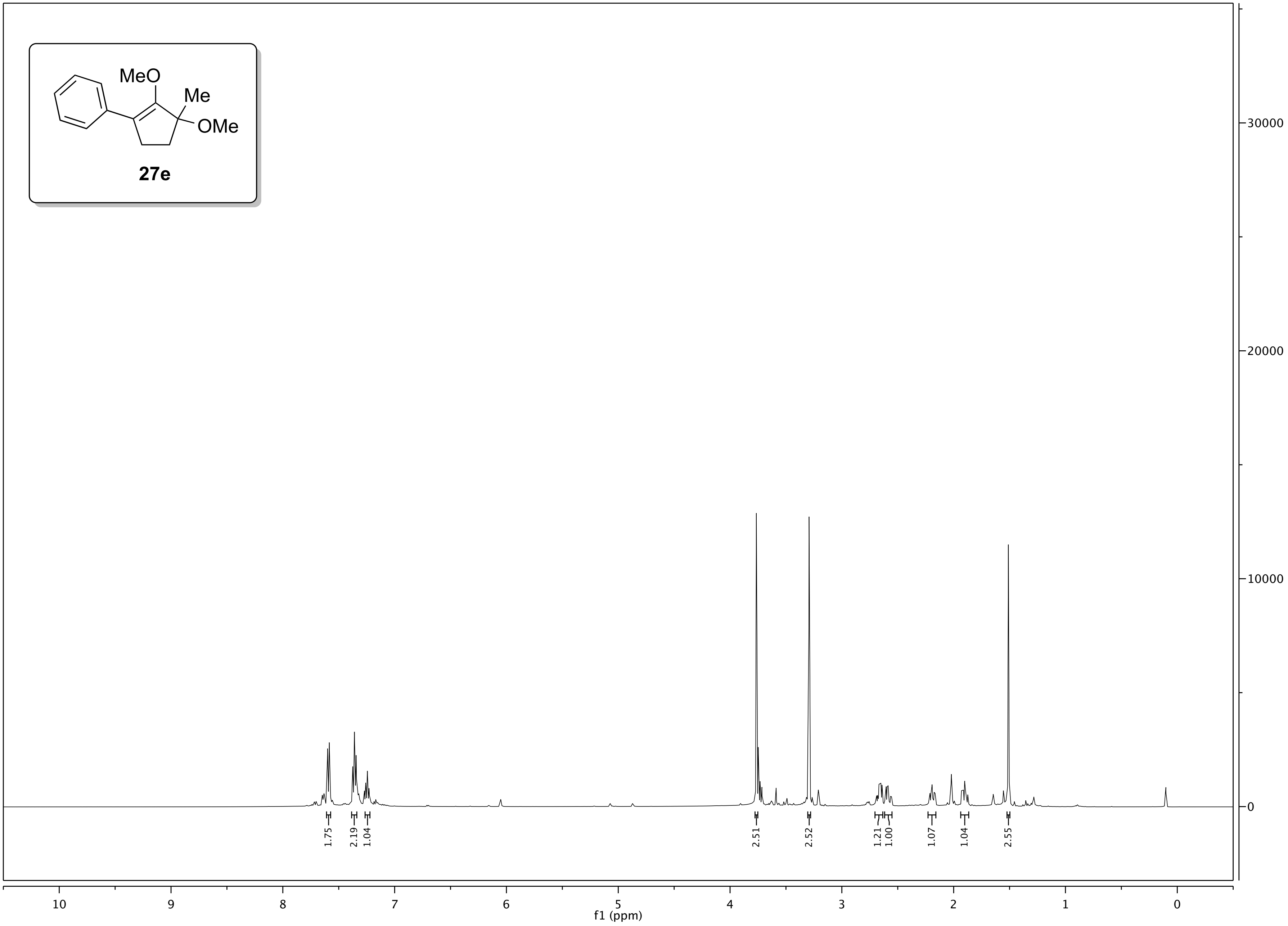
MeO
Me
OMe
27e
30000
20000
10000
0
1.75
2.19
1.04
2.51
2.52
1.21
1.00
1.07
1.04
2.55
10
9
8
7
6
5
4
3
2
1
0
f1 (ppm)

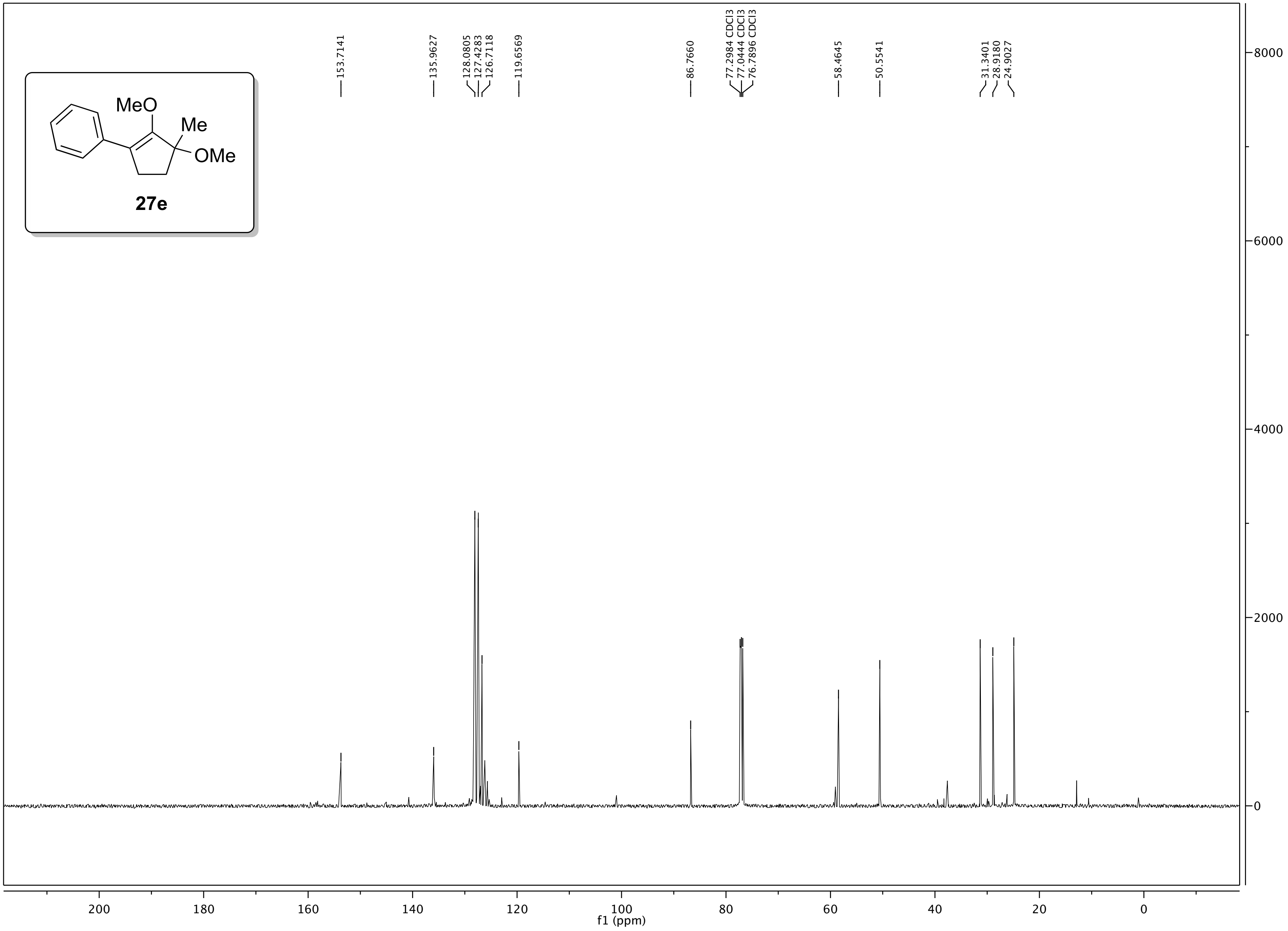

MeO
Me
OMe
27e
153.7141
135.9627
128.0805
127.4283
126.7118
119.6569
86.7660
77.2984 CDCl3
77.0444 CDCl3
76.7896 CDCl3
58.4645
50.5541
31.3401
28.9180
24.9027
8000
6000
4000
2000
0
200
180
160
140
120
100
80
60
40
20
0
f1 (ppm)

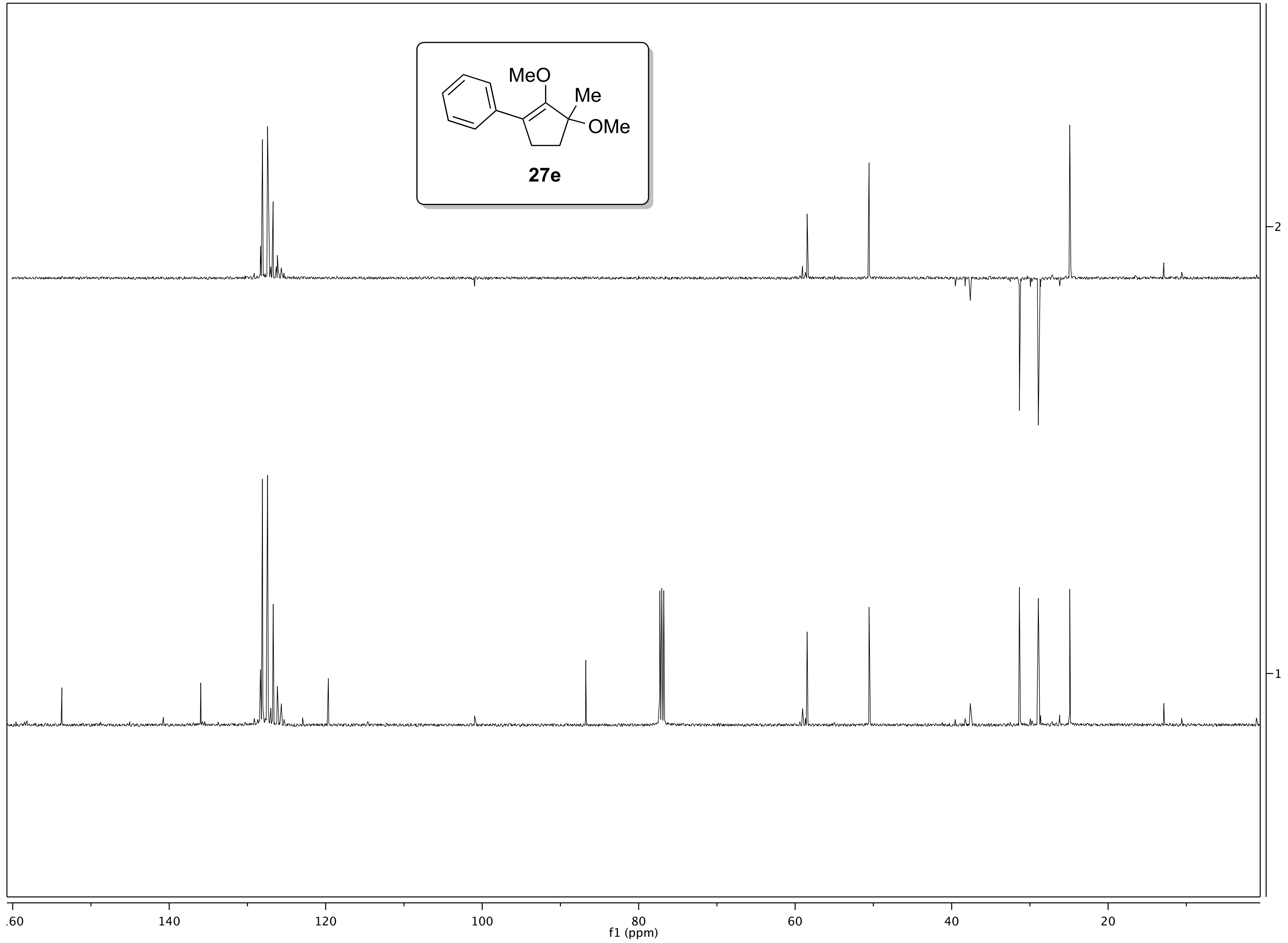
MeO
Me
OMe
27e
2
1
.60
140
120
100
80
60
40
20
f1 (ppm)

HMBC

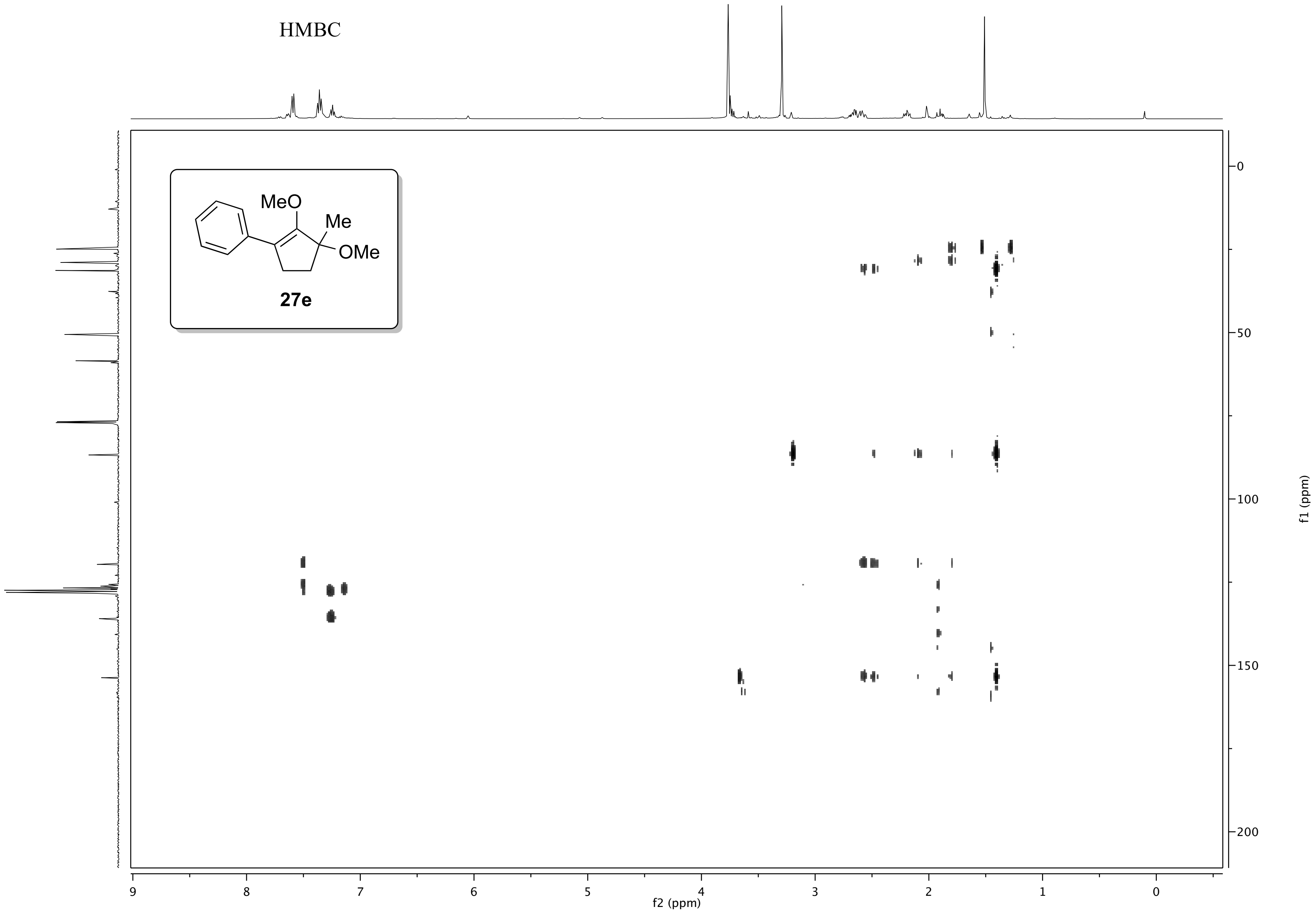

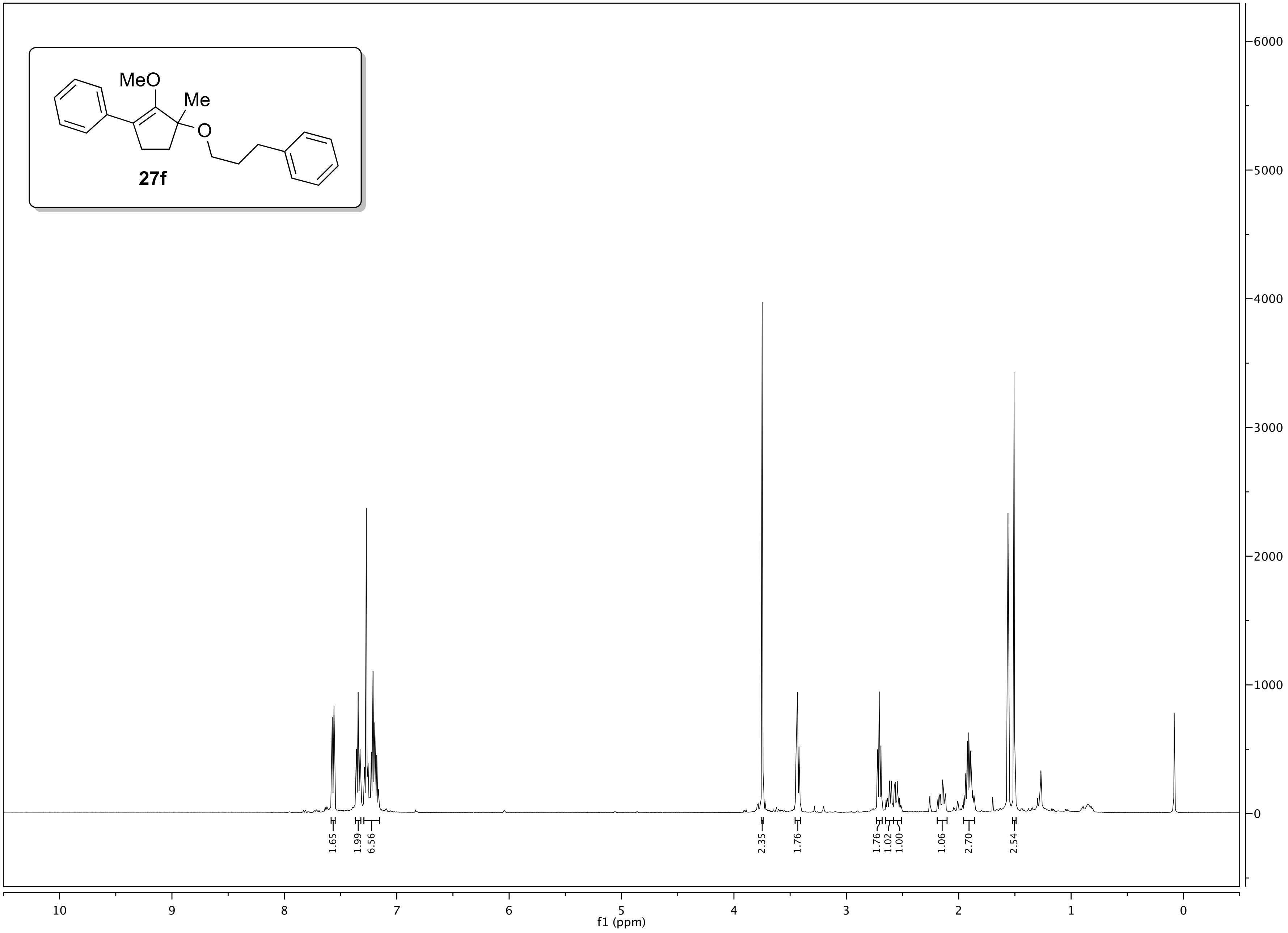
MeO
Me
O
27f
1.65
1.99
6.56
2.35
1.76
1.76
1.02
1.00
1.06
2.70
2.54
10
9
8
7
6
5
4
3
2
1
0
f1 (ppm)
6000
5000
4000
3000
2000
1000
0

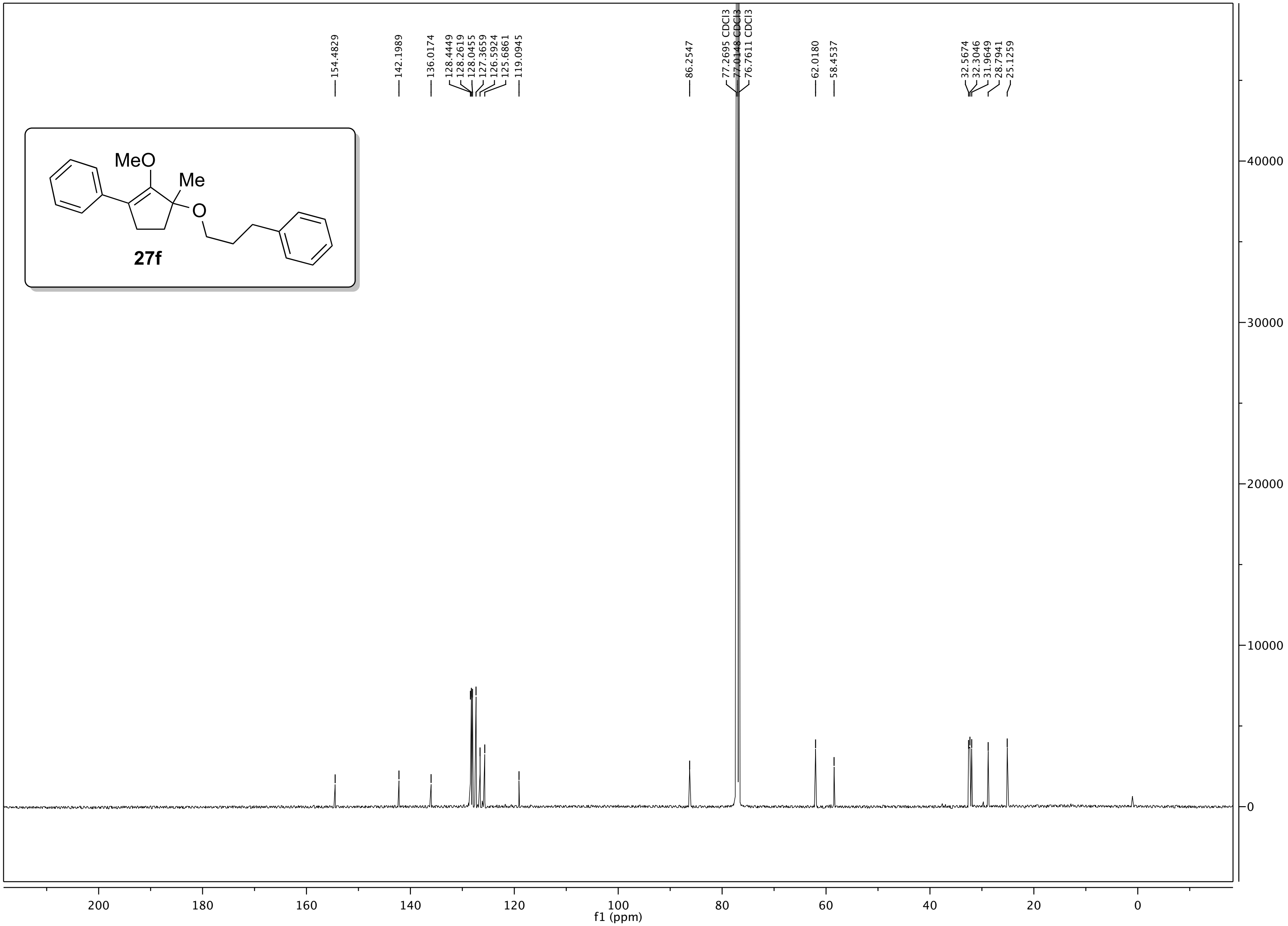
154.4829
142.1989
136.0174
128.4449
128.2619
128.0455
127.3659
126.5924
125.6861
119.0945
86.2547
77.2695 CDCl3
77.0148 CDCl3
76.7611 CDCl3
62.0180
58.4537
32.5674
32.3046
31.9649
28.7941
25.1259
MeO
Me
O
27f
40000
30000
20000
10000
0
200
180
160
140
120
100
80
60
40
20
0
f1 (ppm)

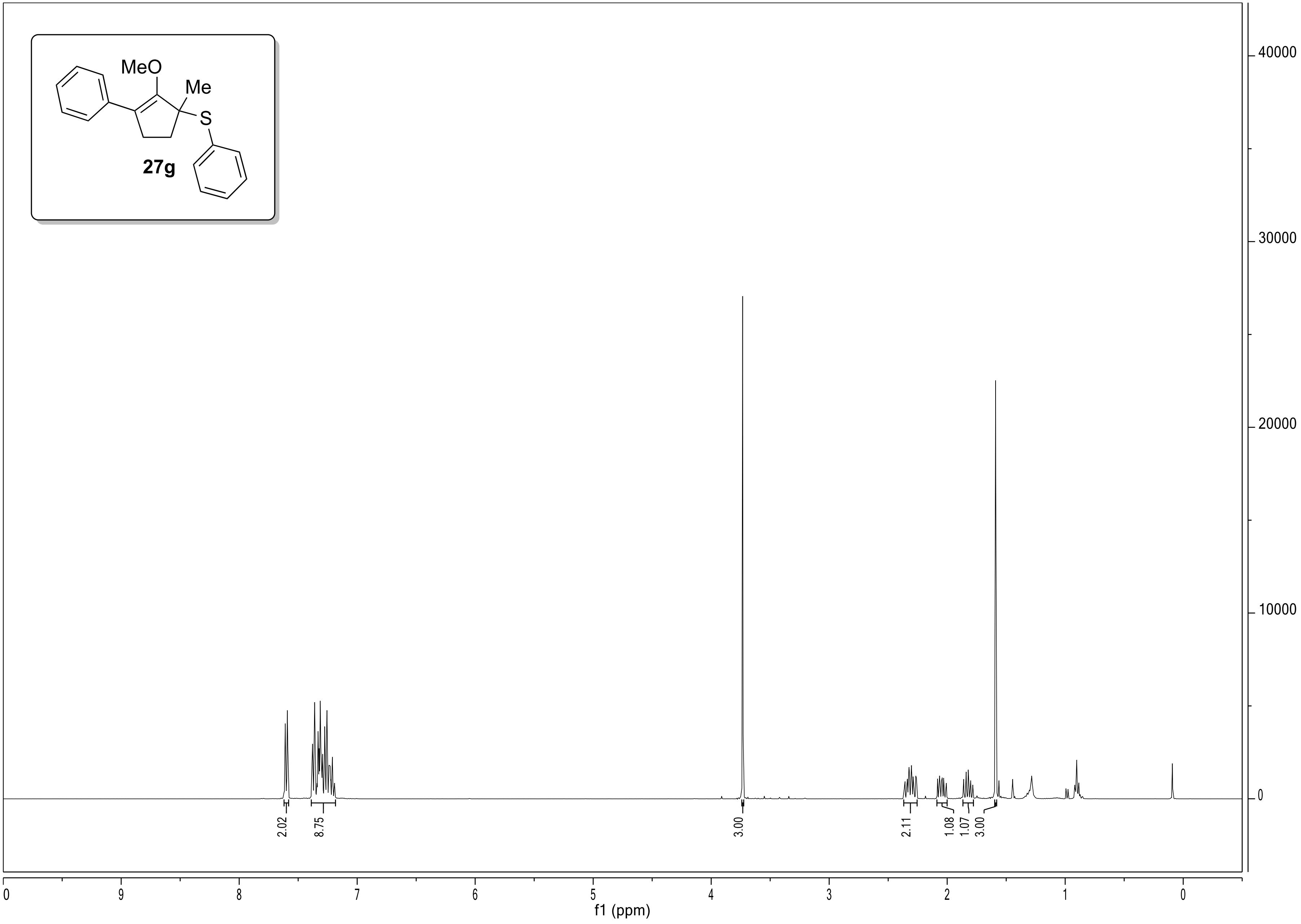
MeO
Me
S
27g
40000
30000
20000
10000
0
2.02
8.75
3.00
2.11
1.08
1.07
3.00
0
9
8
7
6
5
4
3
2
1
0
f1 (ppm)

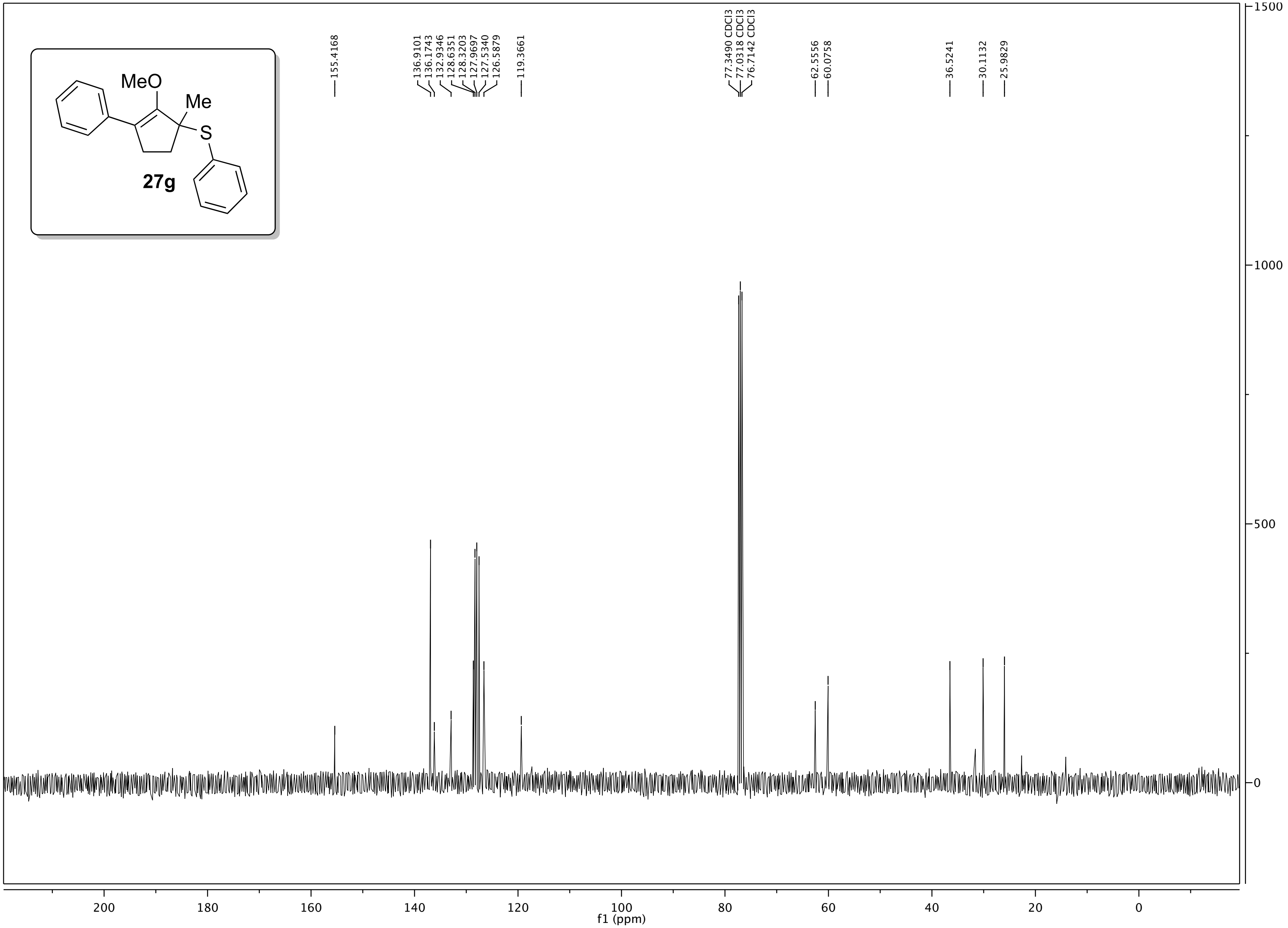
MeO
Me
S
27g
155.4168
136.9101
136.1743
132.9346
128.6351
128.3203
127.9697
127.5340
126.5879
119.3661
77.3490 CDCl3
77.0318 CDCl3
76.7142 CDCl3
62.5556
60.0758
36.5241
30.1132
25.9829
f1 (ppm)

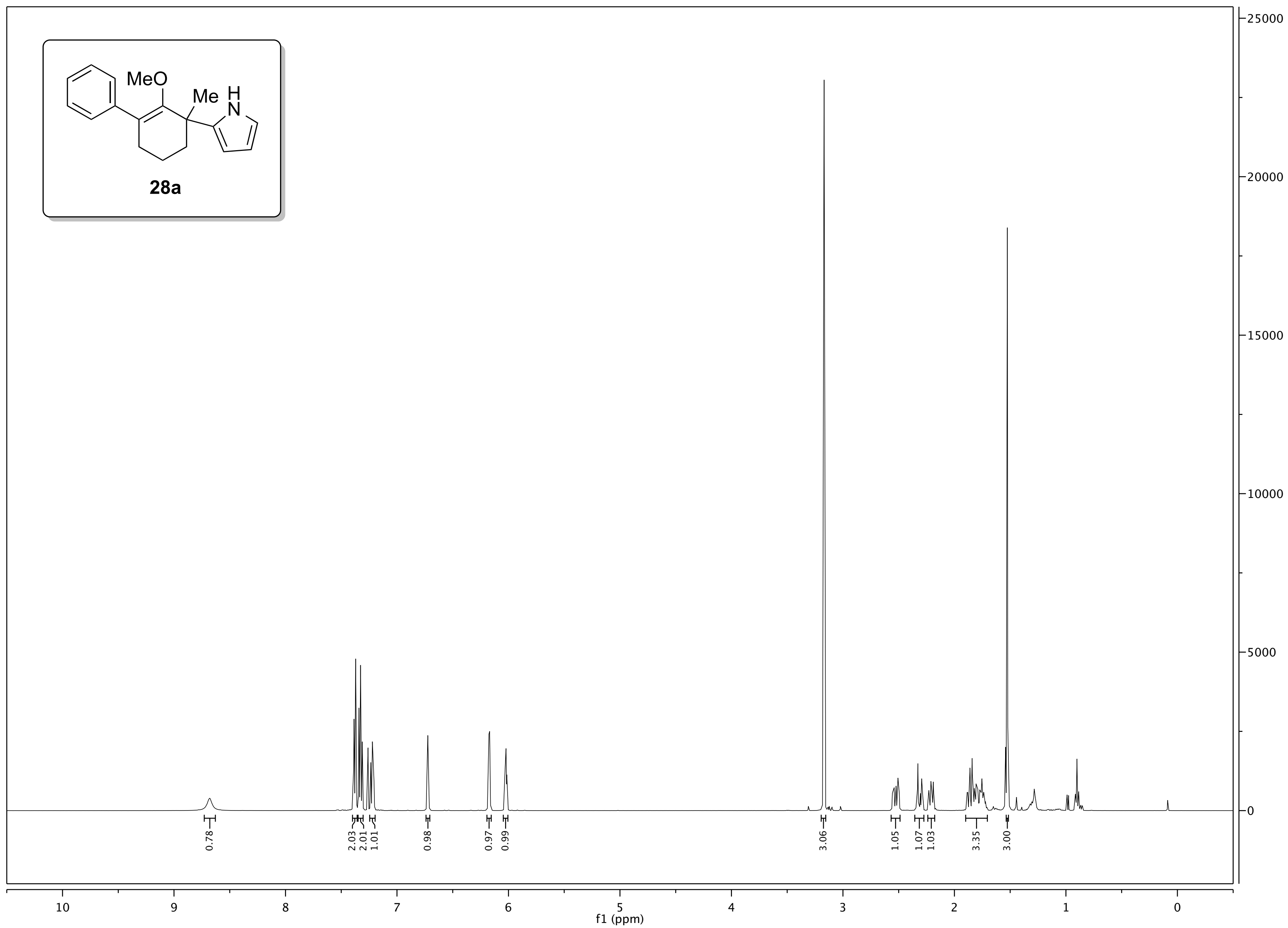
MeO
Me
H
N
28a
f1 (ppm)

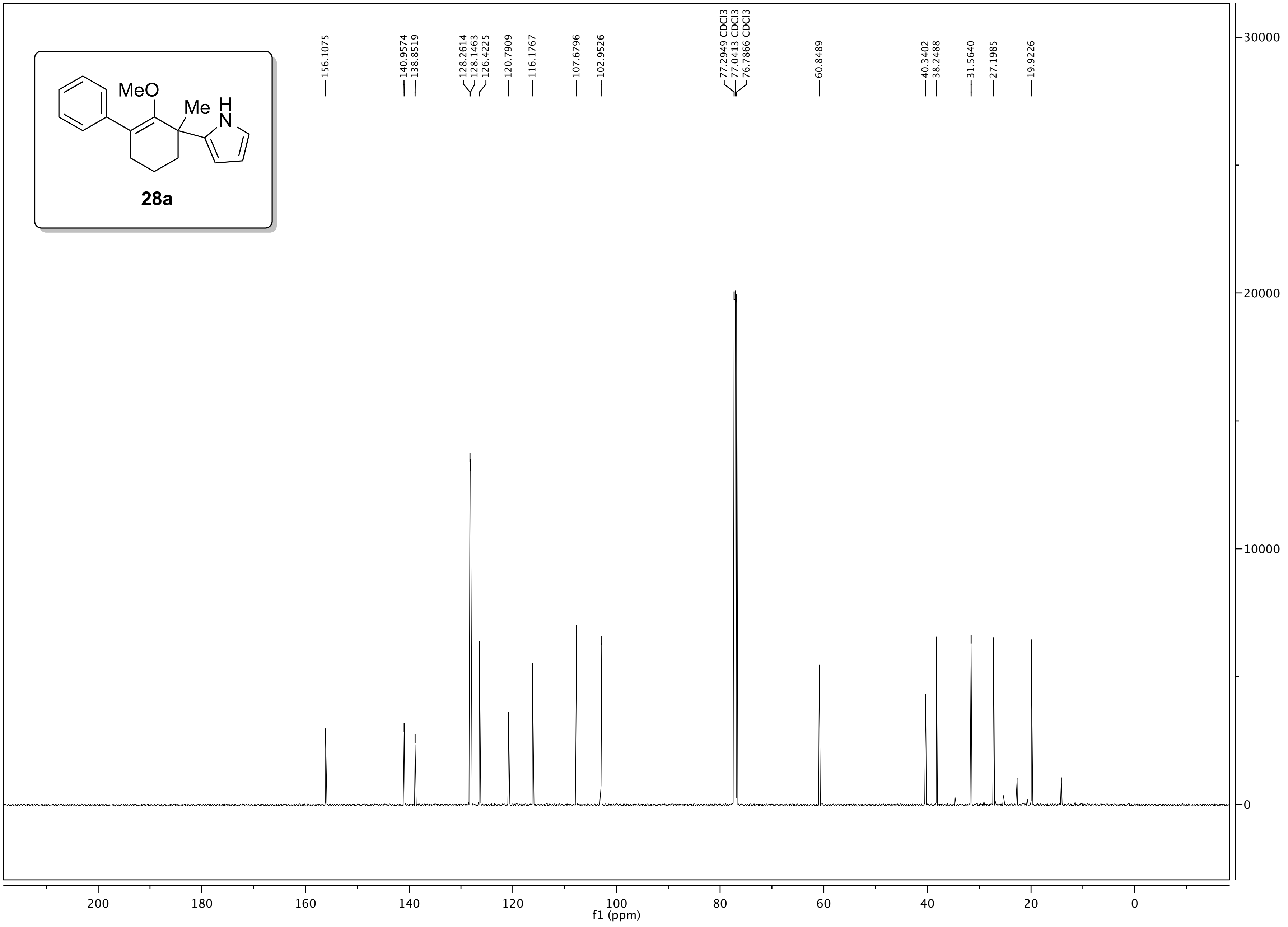

MeO
Me
H
N
28a
156.1075
140.9574
138.8519
128.2614
128.1463
126.4225
120.7909
116.1767
107.6796
102.9526
77.2949 CDCl3
77.0413 CDCl3
76.7866 CDCl3
60.8489
40.3402
38.2488
31.5640
27.1985
19.9226
30000
20000
10000
0
200
180
160
140
120
100
80
60
40
20
0
f1 (ppm)

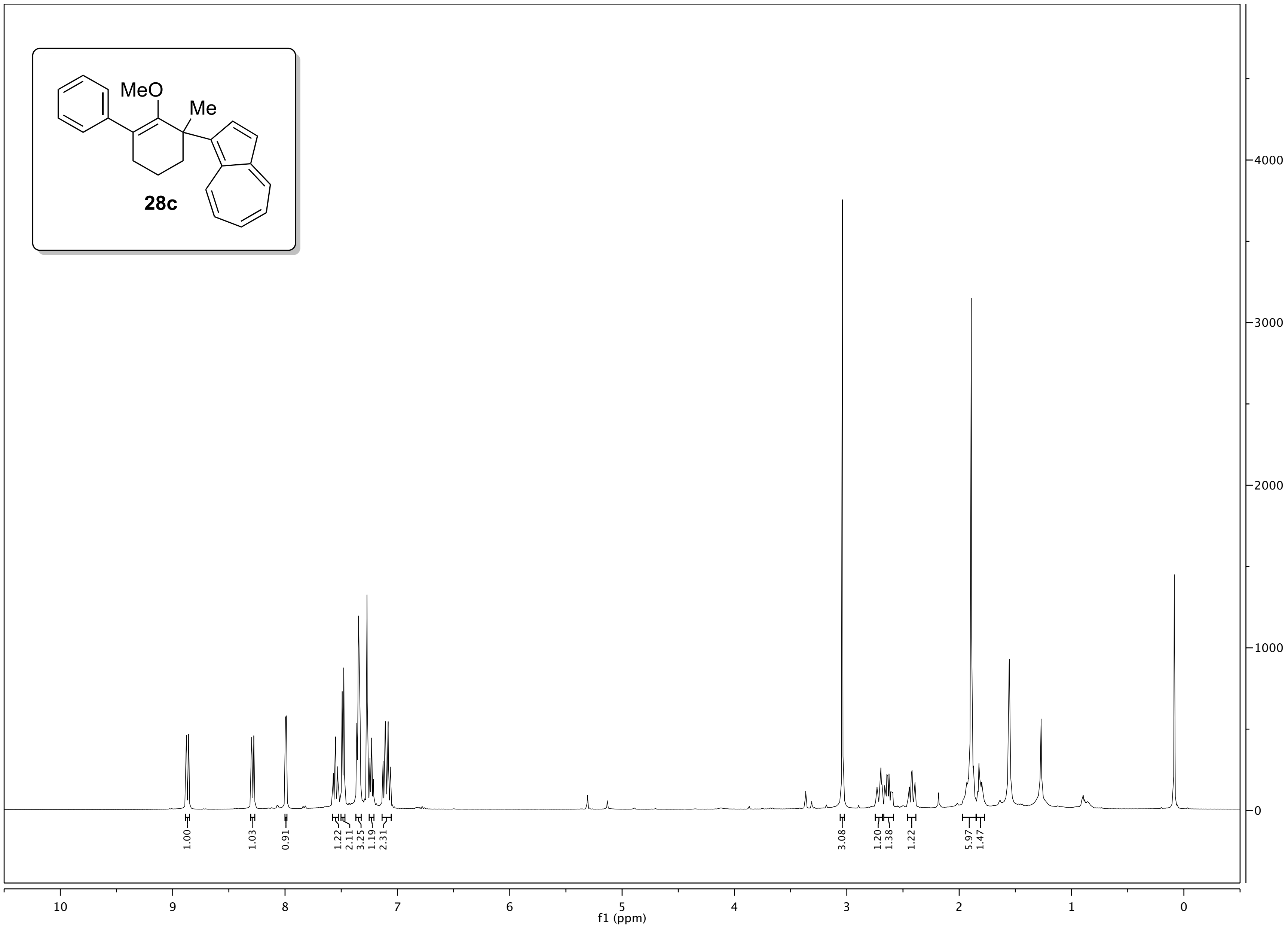
MeO
Me
28c
f1 (ppm)

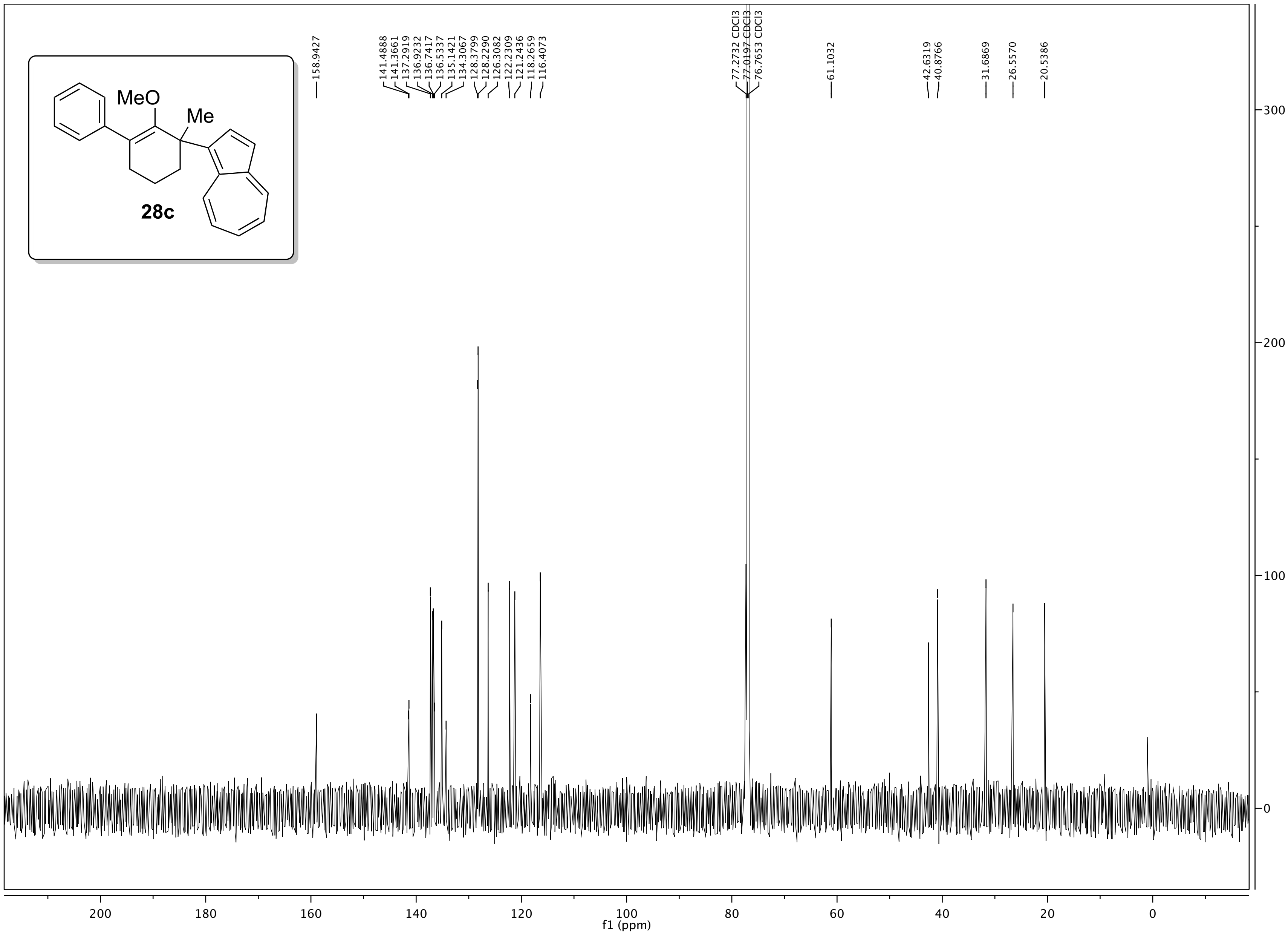
MeO
Me
28c
158.9427
141.4888
141.3661
137.2919
136.9232
136.7417
136.5337
135.1421
134.3067
128.3799
128.2290
126.3082
122.2309
121.2436
118.2659
116.4073
77.2732 CDCl3
77.0197 CDCl3
76.7653 CDCl3
61.1032
42.6319
40.8766
31.6869
26.5570
20.5386
300
200
100
0
200
180
160
140
120
100
80
60
40
20
0
f1 (ppm)

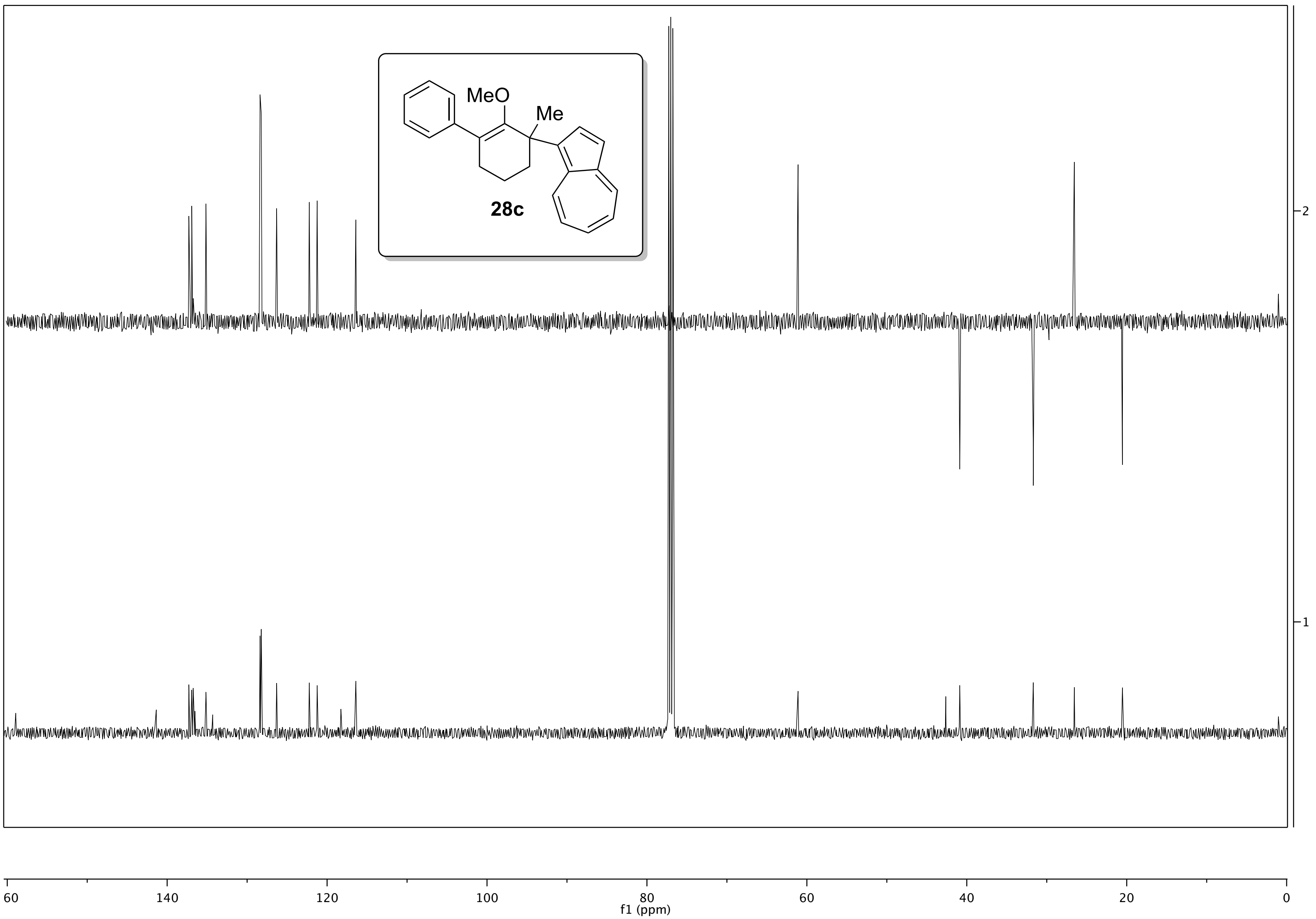
MeO
Me
28c
f1 (ppm)

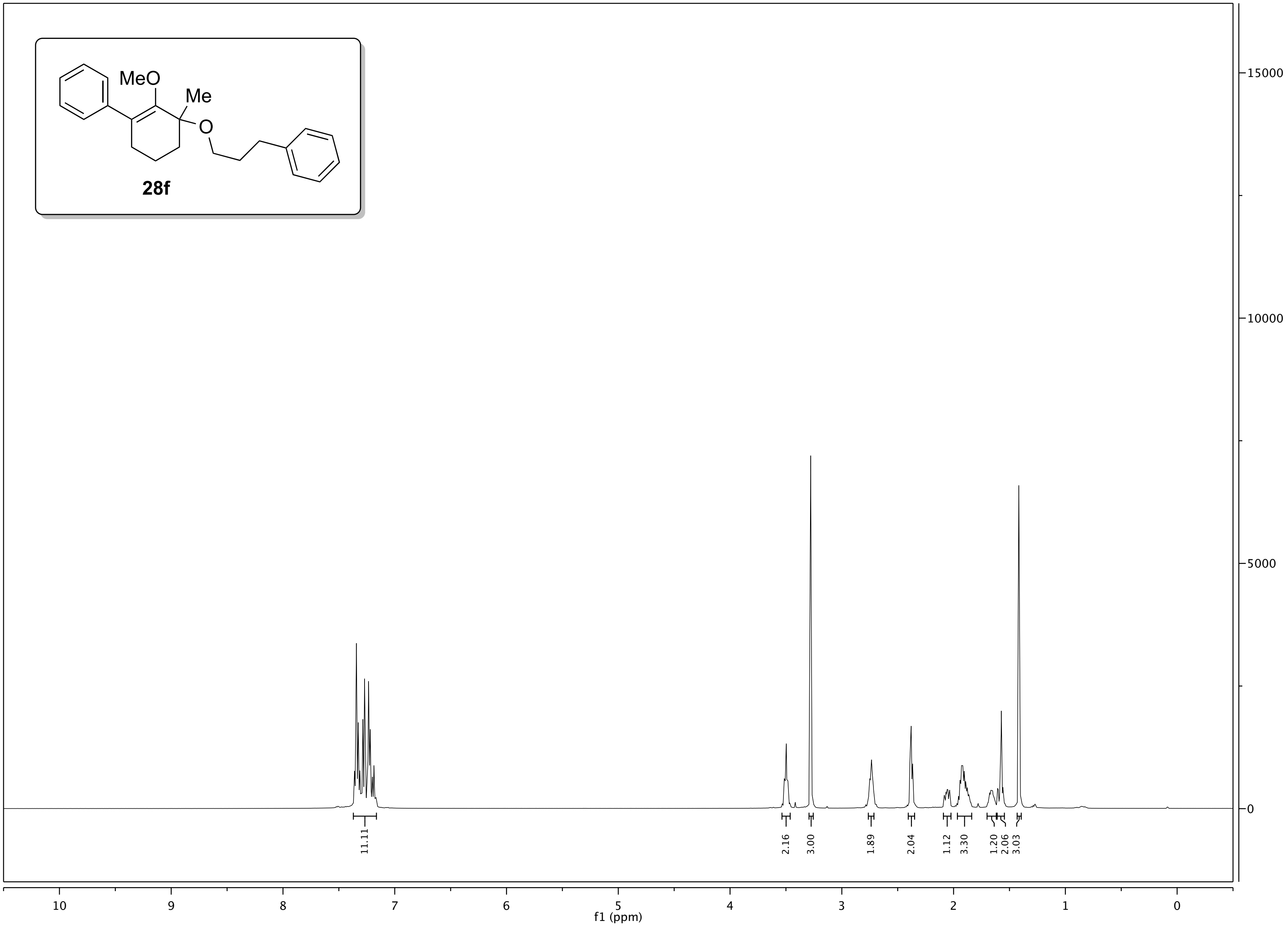
MeO
Me
O
28f
11.11
2.16
3.00
1.89
2.04
1.12
3.30
1.20
2.06
3.03
15000
10000
5000
0
10
9
8
7
6
5
4
3
2
1
0
f1 (ppm)

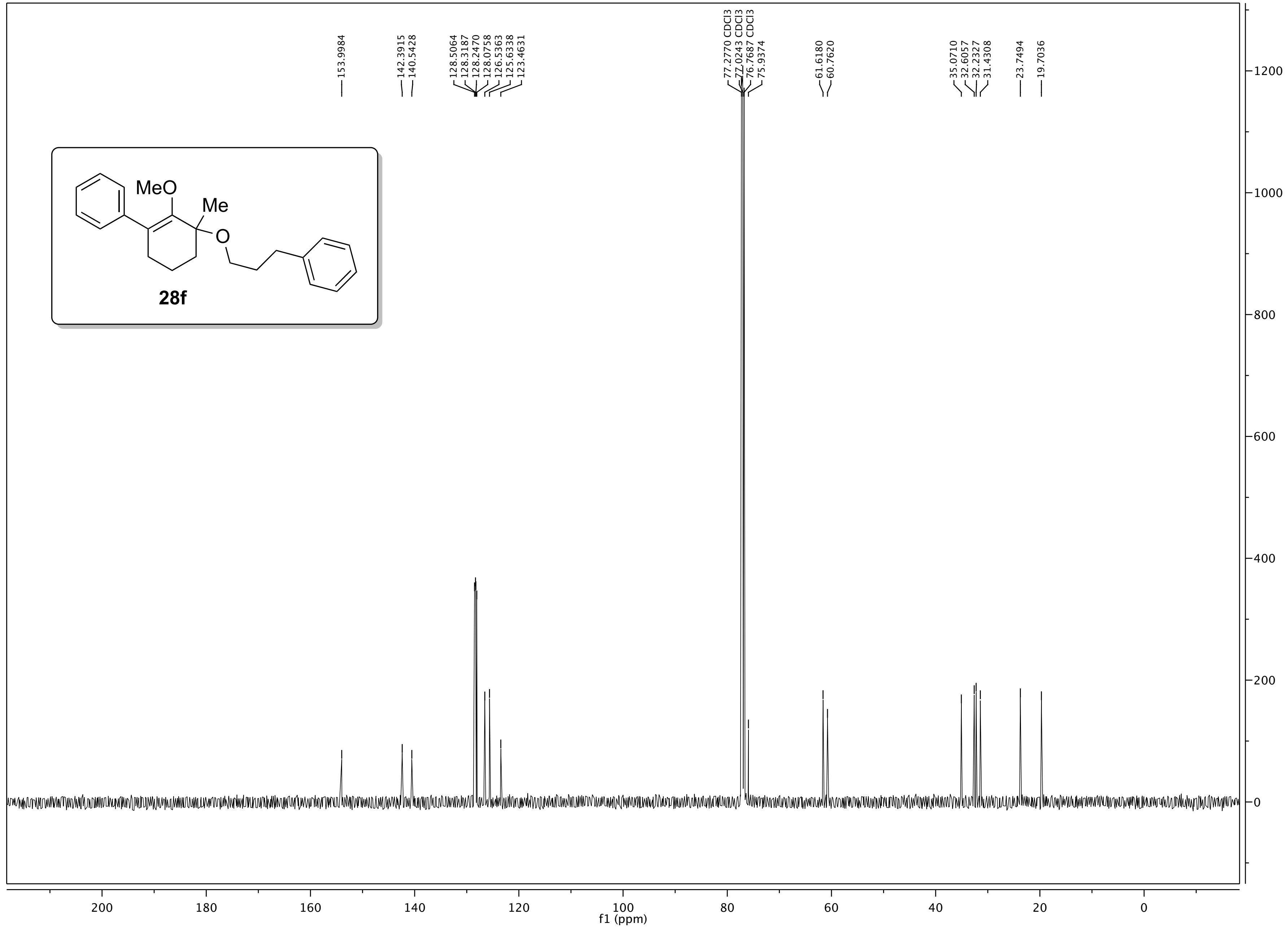

153.9984
142.3915
140.5428
128.5064
128.3187
128.2470
128.0758
126.5363
125.6338
123.4631
77.2770 CDCl3
77.0243 CDCl3
76.7687 CDCl3
75.9374
61.6180
60.7620
35.0710
32.6057
32.2327
31.4308
23.7494
19.7036
MeO
Me
O
28f
f1 (ppm)

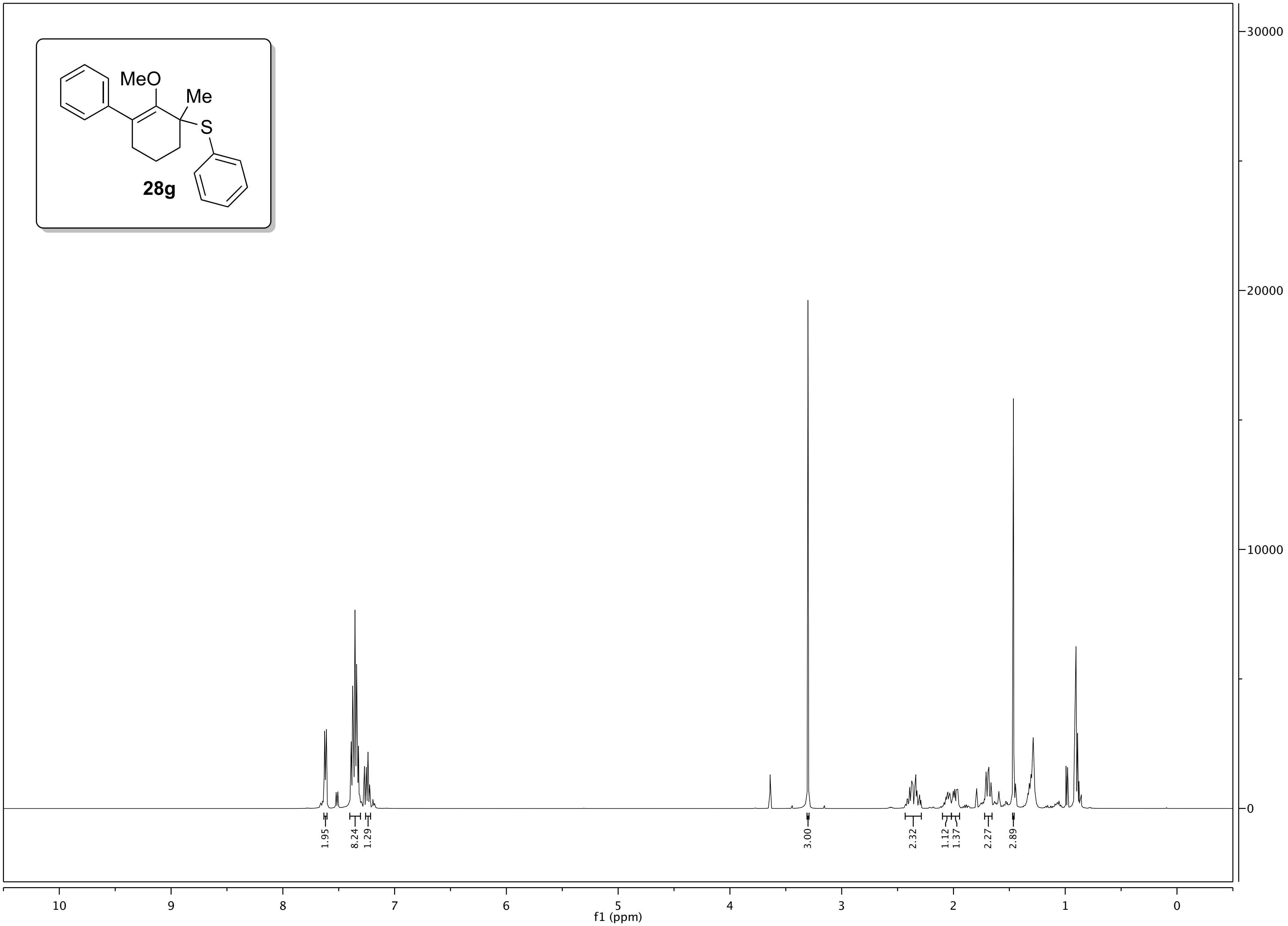
MeO
Me
S
28g
30000
20000
10000
0
1.95
8.24
1.29
3.00
2.32
1.12
1.37
2.27
2.89
10
9
8
7
6
5
4
3
2
1
0
f1 (ppm)

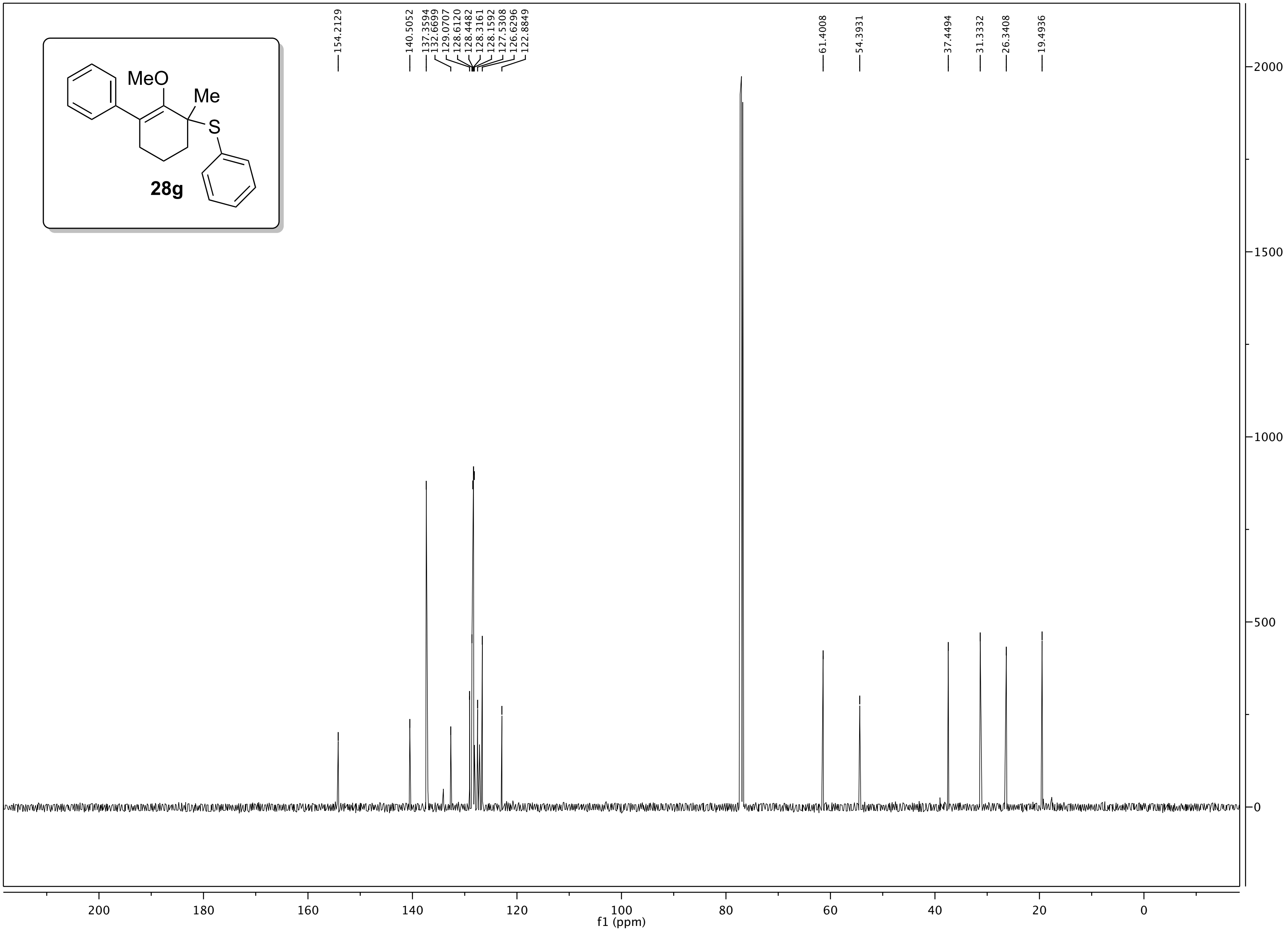
MeO
Me
S
28g
154.2129
140.5052
137.3594
132.6699
129.0707
128.6120
128.4482
128.3161
128.1592
127.5308
126.6296
122.8849
61.4008
54.3931
37.4494
31.3332
26.3408
19.4936
f1 (ppm)

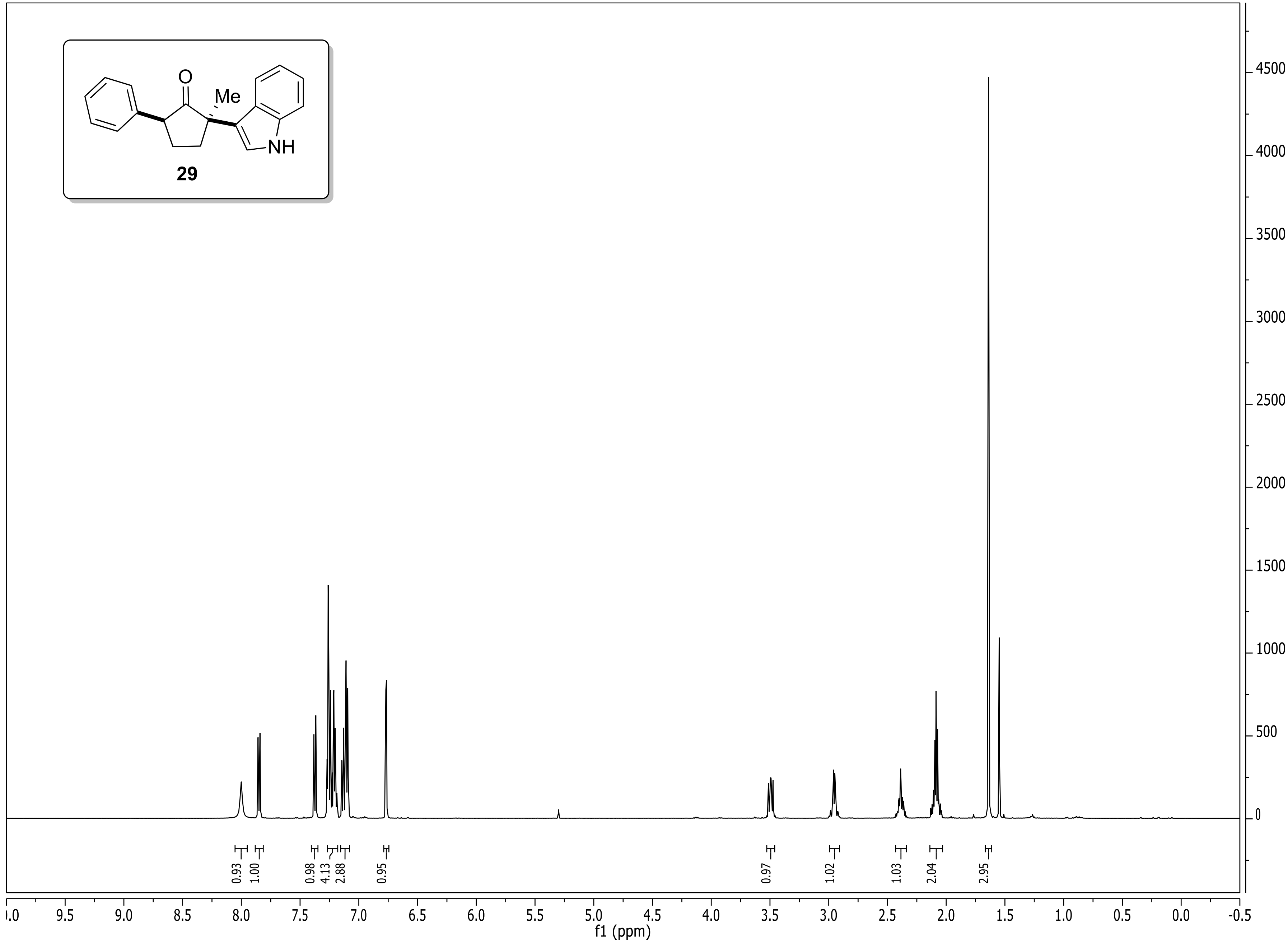

O
Me
NH
29
4500
4000
3500
3000
2500
2000
1500
1000
500
0
0.93
1.00
0.98
4.13
2.88
0.95
0.97
1.02
1.03
2.04
2.95
10.0
9.5
9.0
8.5
8.0
7.5
7.0
6.5
6.0
5.5
5.0
4.5
4.0
3.5
3.0
2.5
2.0
1.5
1.0
0.5
0.0
-0.5
f1 (ppm)

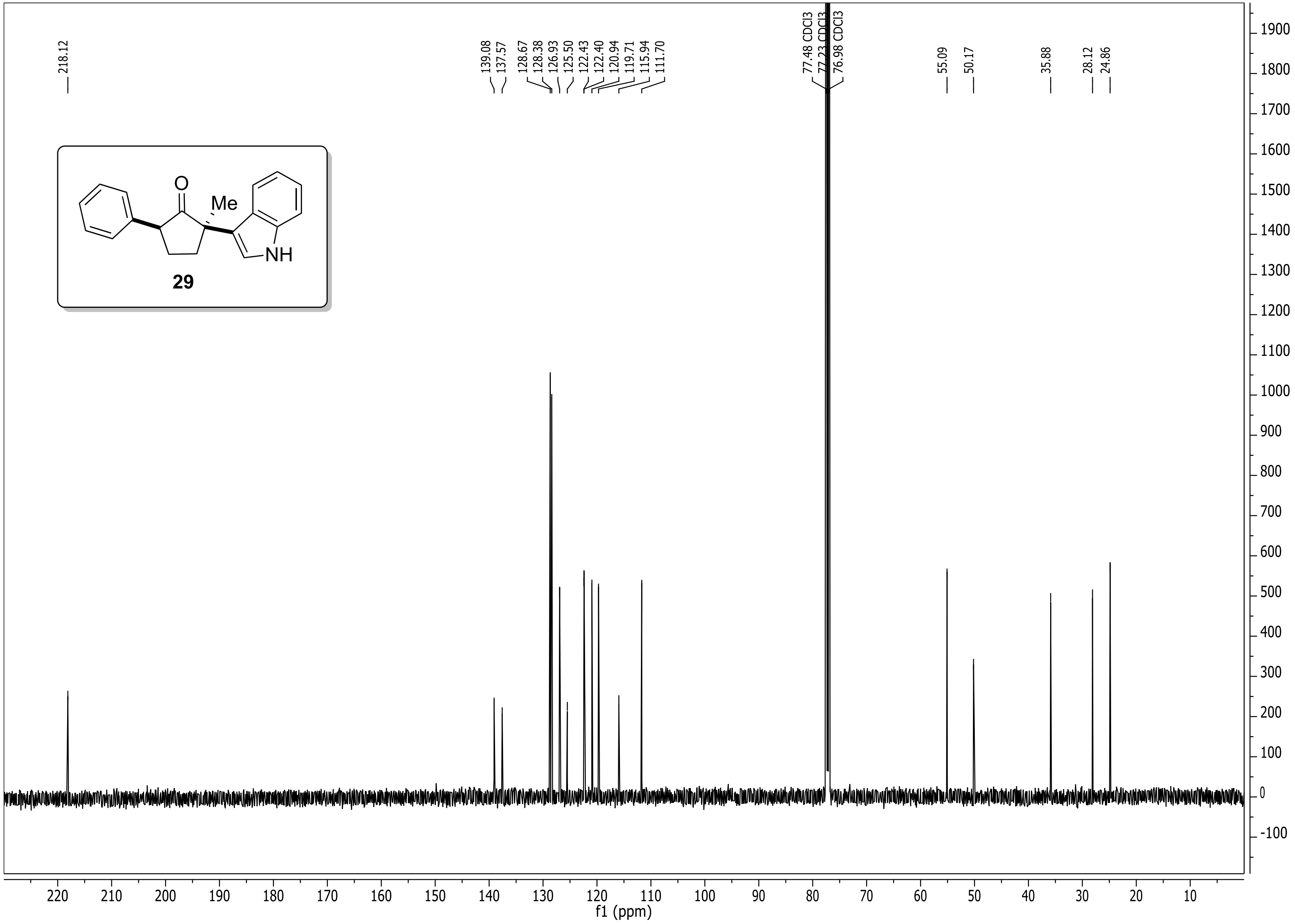
218.12
139.08
137.57
128.67
128.38
126.93
125.50
122.43
122.40
120.94
119.71
115.94
111.70
77.48 CDCl3
77.23 CDCl3
76.98 CDCl3
55.09
50.17
35.88
28.12
24.86
O
Me
NH
29
f1 (ppm)

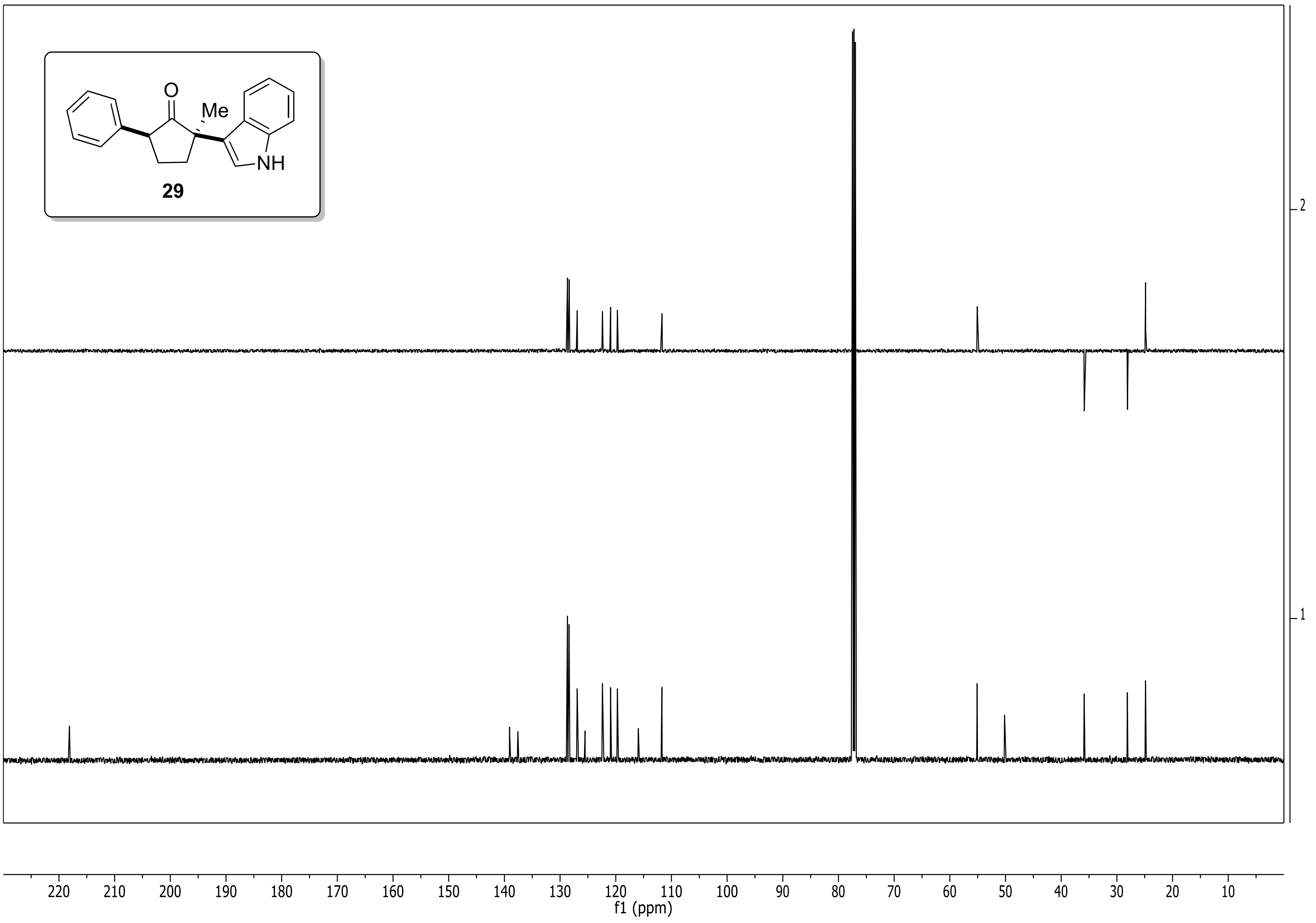
Me
NH
O
29
2
1
220
210
200
190
180
170
160
150
140
130
120
110
100
90
80
70
60
50
40
30
20
10
f1 (ppm)

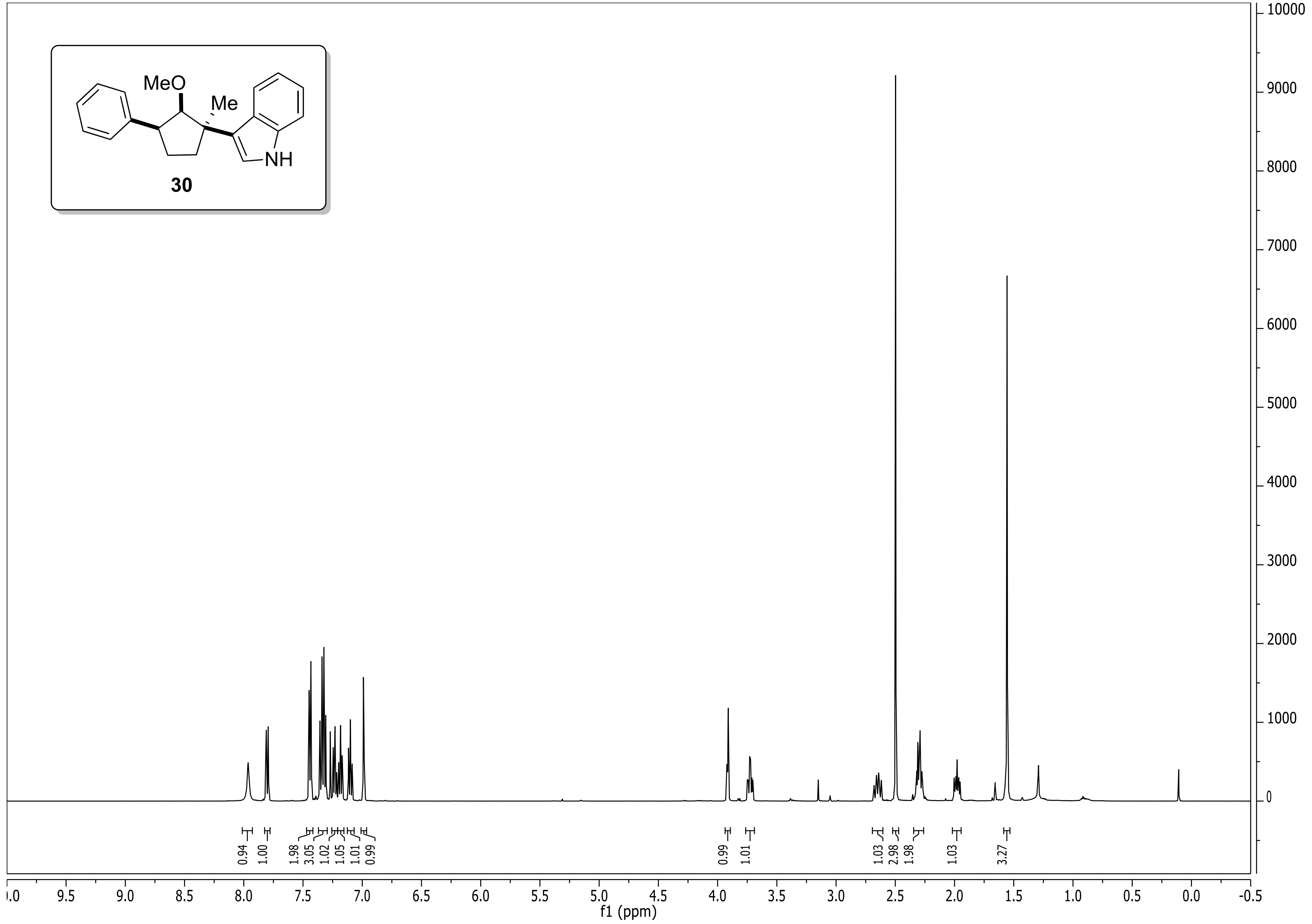
MeO
Me
NH
30
f1 (ppm)

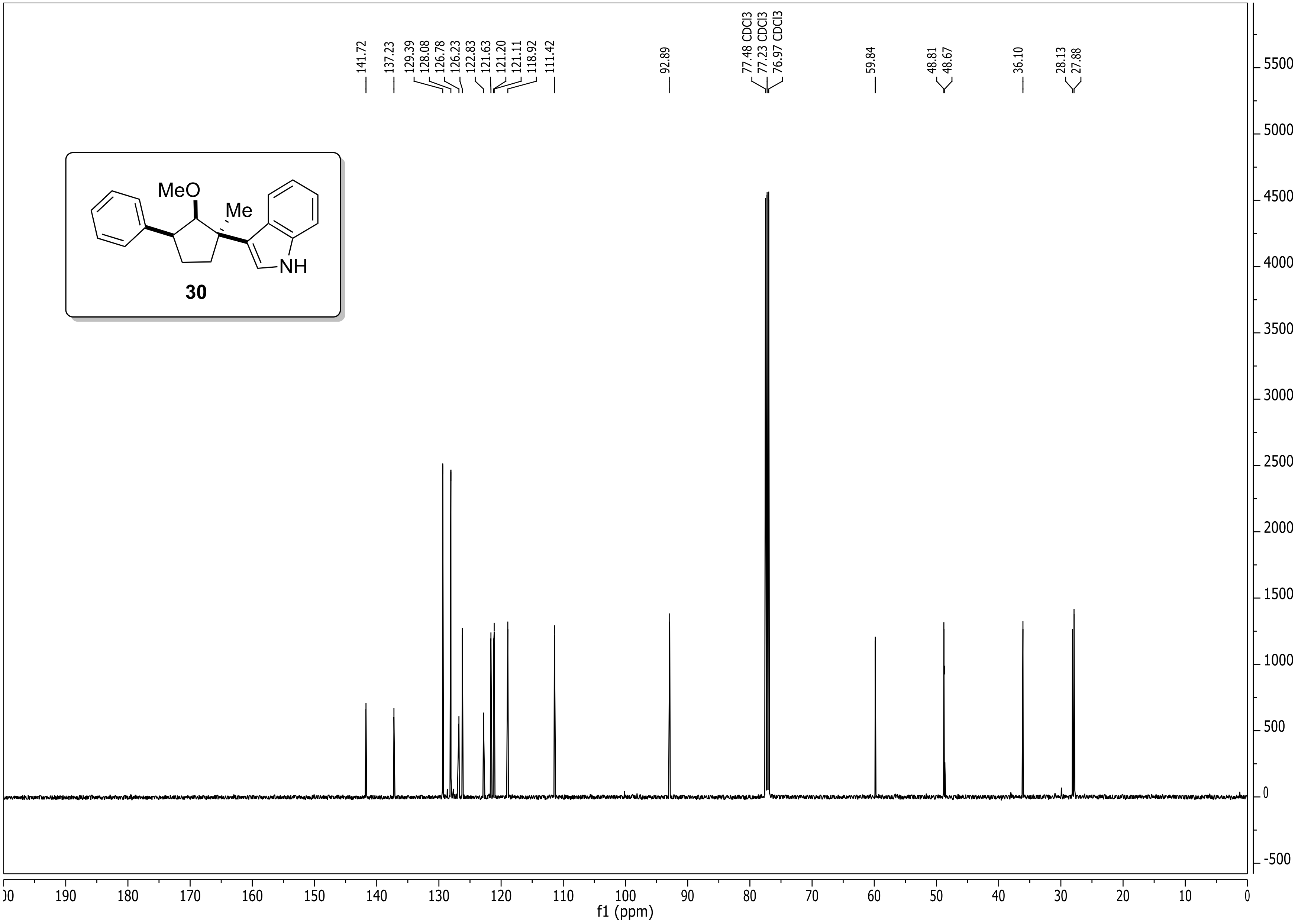

141.72
137.23
129.39
128.08
126.78
126.23
122.83
121.63
121.20
121.11
118.92
111.42
92.89
77.48 CDCl3
77.23 CDCl3
76.97 CDCl3
59.84
48.81
48.67
36.10
28.13
27.88
MeO
Me
NH
30
f1 (ppm)

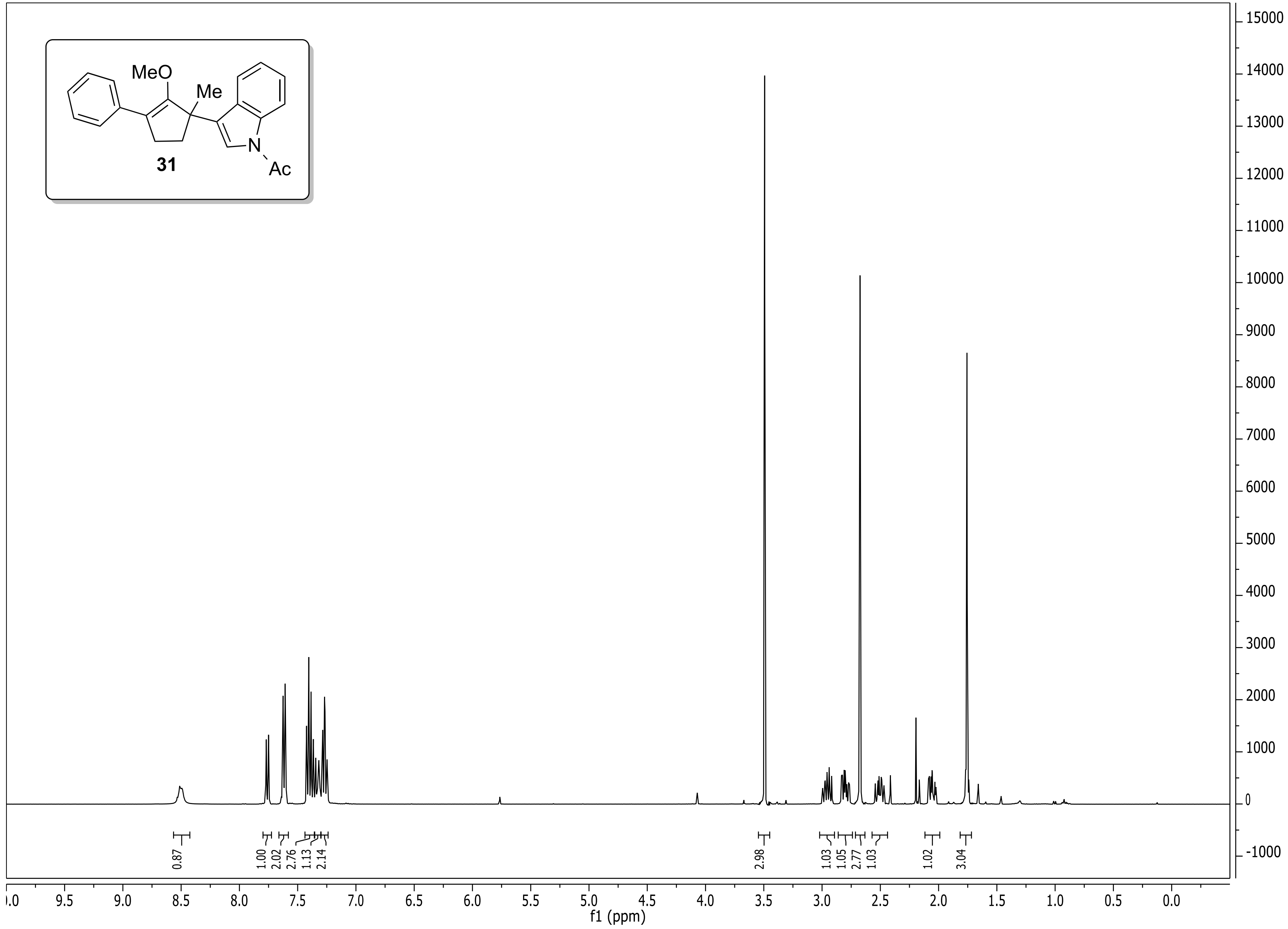
MeO
Me
N
Ac
31
0.87
1.00
2.02
2.76
1.13
2.14
2.98
1.03
1.05
2.77
1.03
1.02
3.04
f1 (ppm)

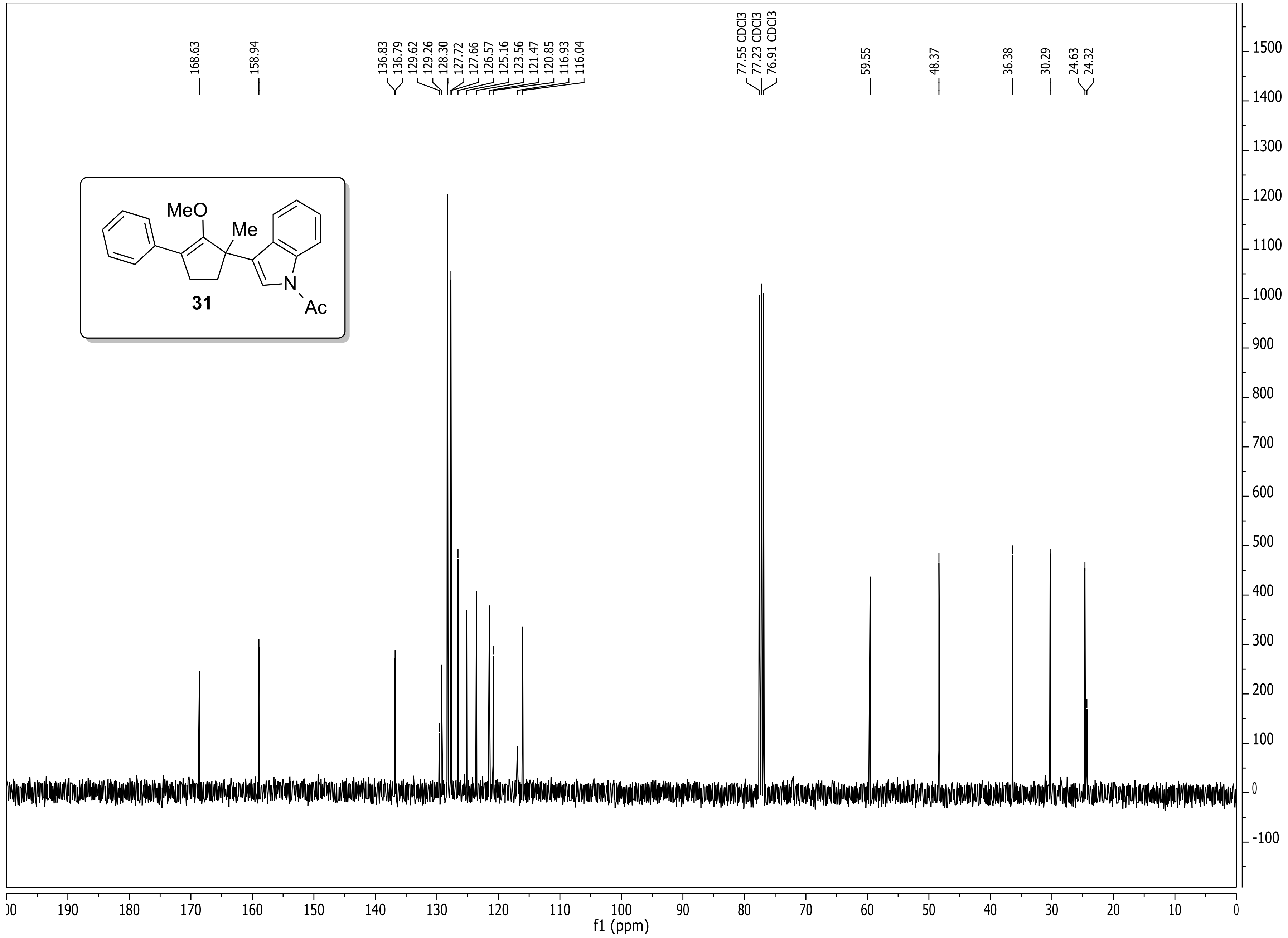
168.63
158.94
136.83
136.79
129.62
129.26
128.30
127.72
127.66
126.57
125.16
123.56
121.47
120.85
116.93
116.04
77.55 CDCl3
77.23 CDCl3
76.91 CDCl3
59.55
48.37
36.38
30.29
24.63
24.32
MeO
Me
N
Ac
31
f1 (ppm)

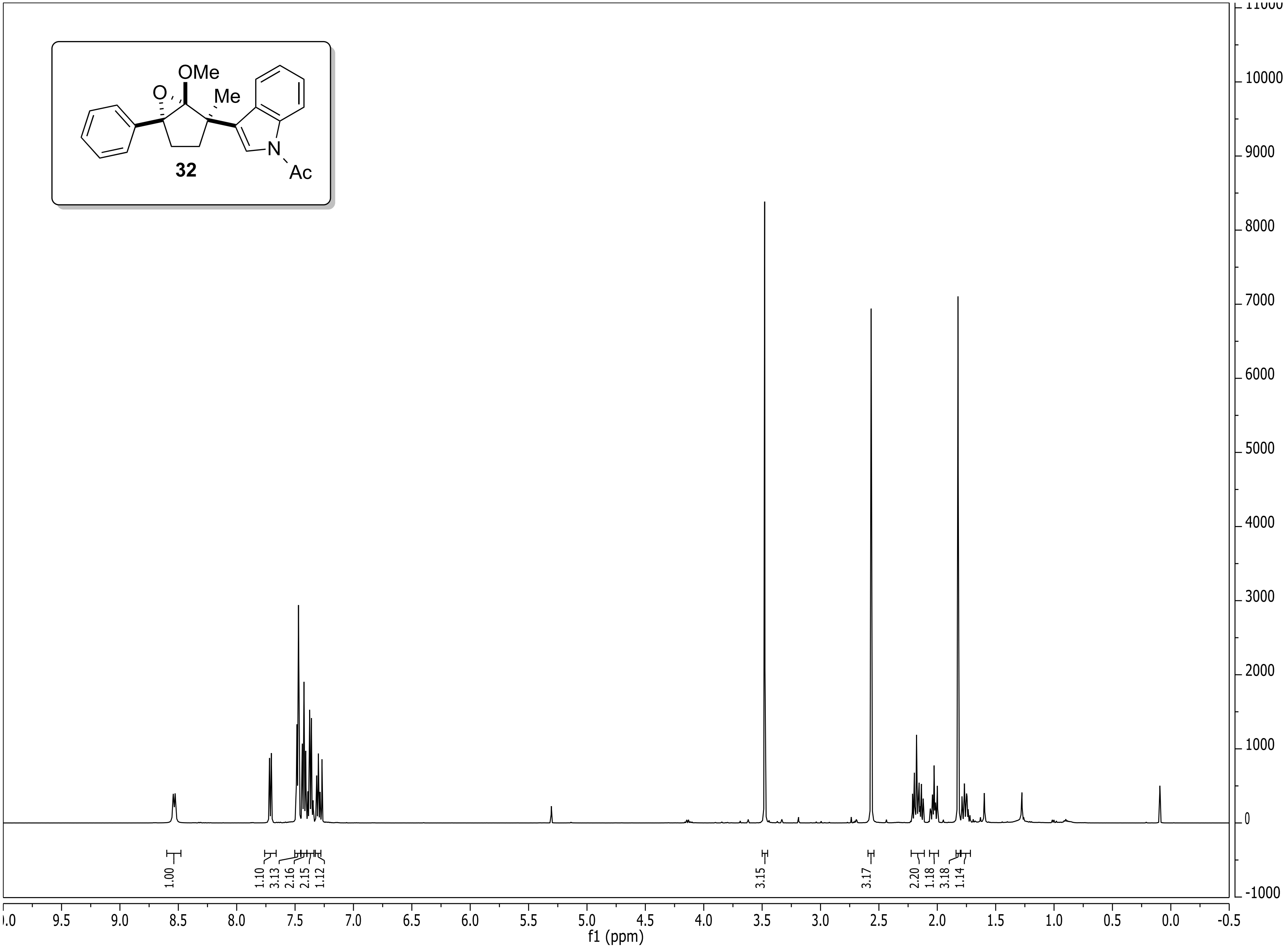
OMe
O
Me
N
Ac
32
1.00
1.10
3.13
2.16
2.15
1.12
3.15
3.17
2.20
1.18
3.18
1.14
f1 (ppm)

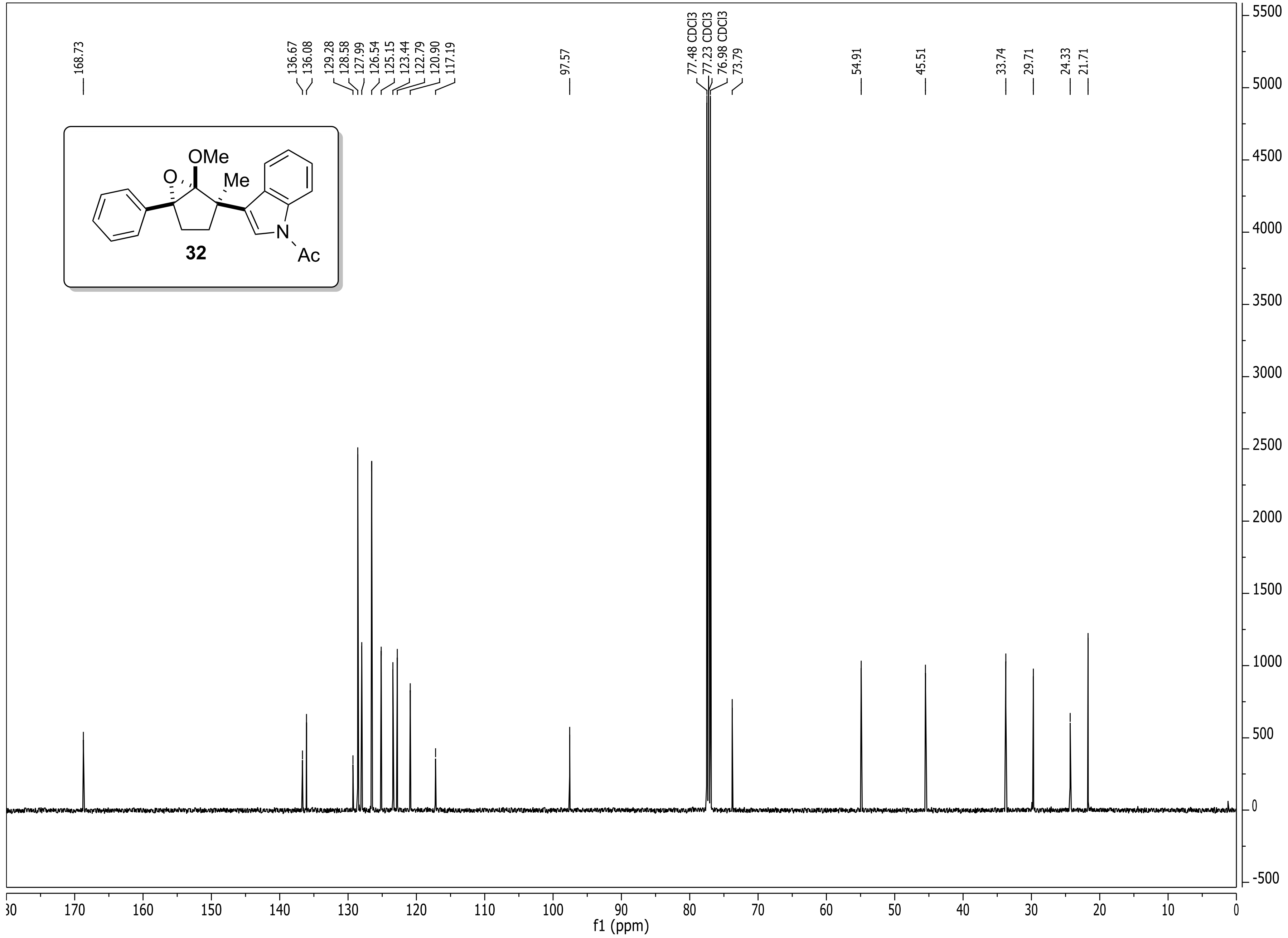

168.73
136.67
136.08
129.28
128.58
127.99
126.54
125.15
123.44
122.79
120.90
117.19
97.57
77.48 CDCl3
77.23 CDCl3
76.98 CDCl3
73.79
54.91
45.51
33.74
29.71
24.33
21.71
OMe
O
Me
N
Ac
32
f1 (ppm)

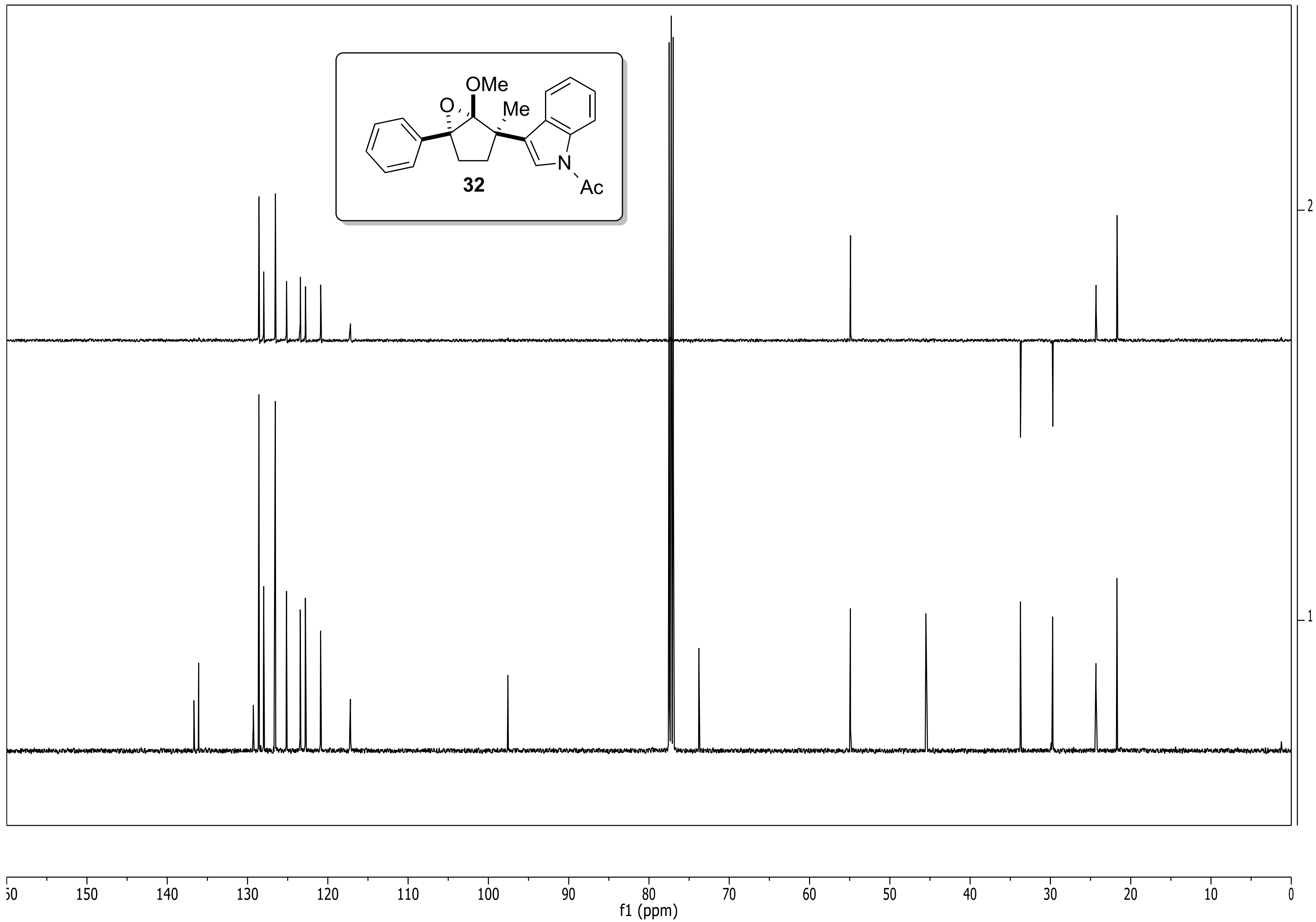

OMe
O
Me
N
Ac
32
2
1
150
140
130
120
110
100
90
80
70
60
50
40
30
20
10
0
f1 (ppm)

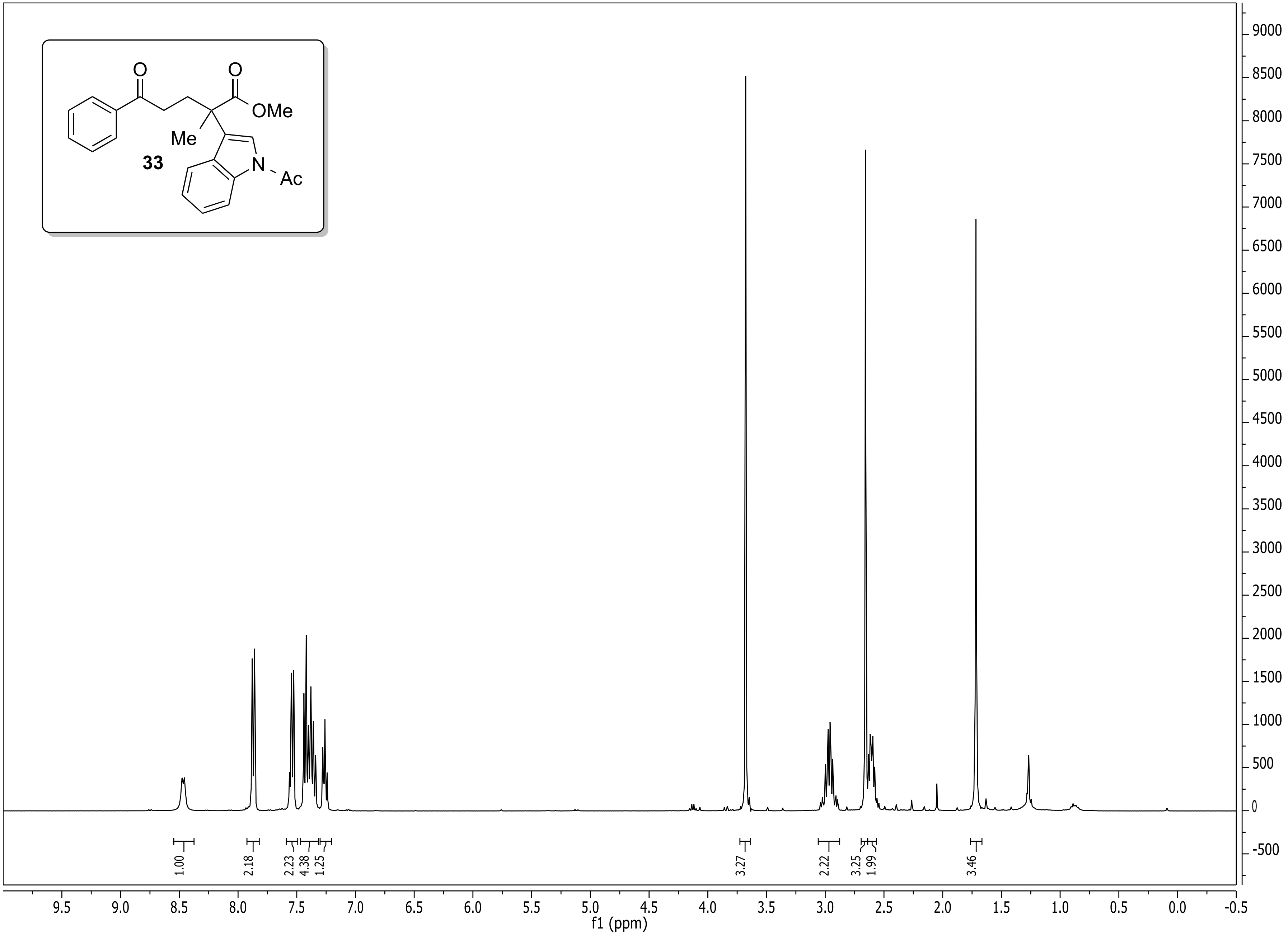
O
O
OMe
Me
33
N
Ac
1.00
2.18
2.23
4.38
1.25
3.27
2.22
3.25
1.99
3.46
9.5 9.0 8.5 8.0 7.5 7.0 6.5 6.0 5.5 5.0 4.5 4.0 3.5 3.0 2.5 2.0 1.5 1.0 0.5 0.0 -0.5
f1 (ppm)
9000 8500 8000 7500 7000 6500 6000 5500 5000 4500 4000 3500 3000 2500 2000 1500 1000 500 0 -500

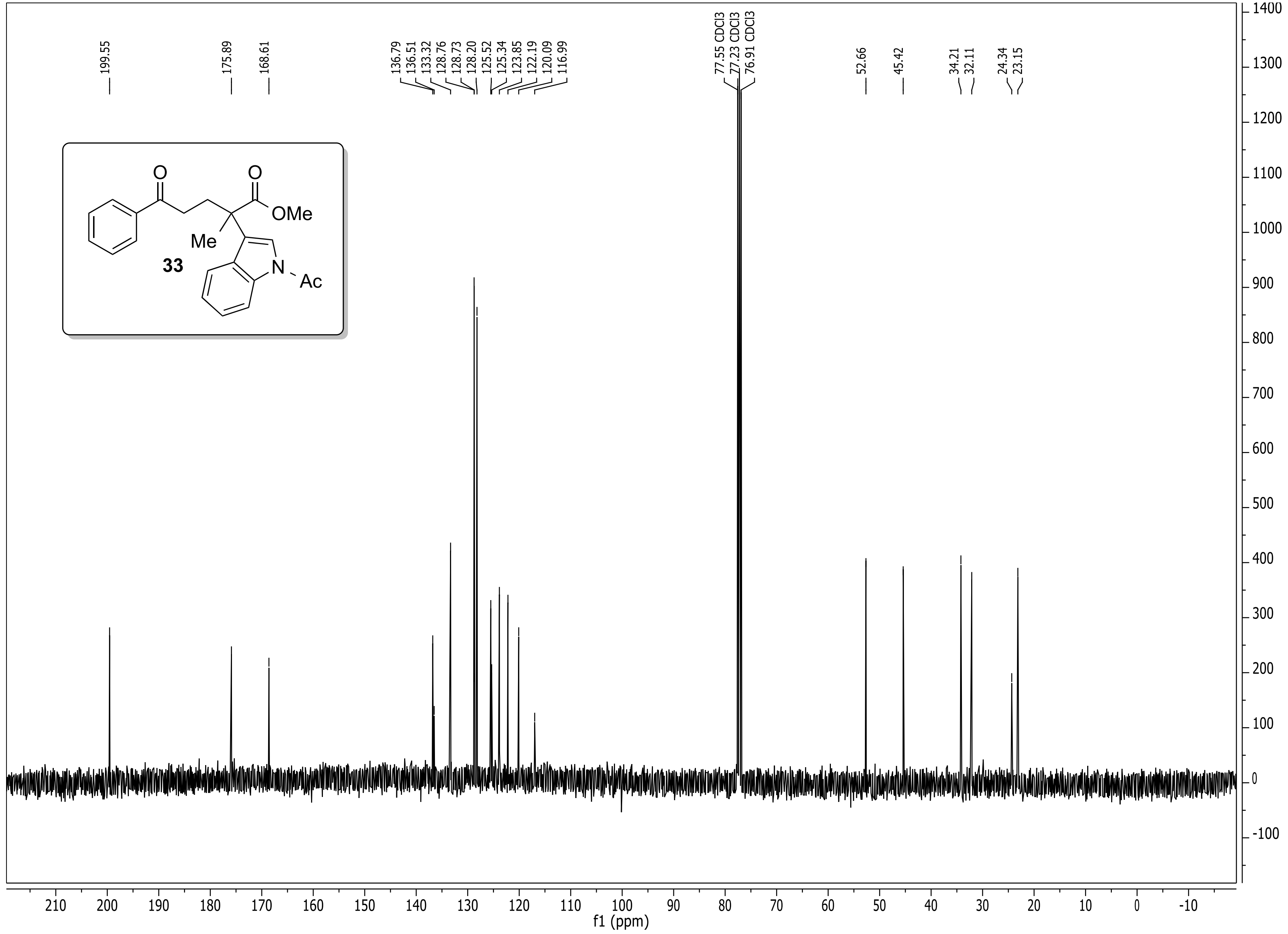
199.55
175.89
168.61
136.79
136.51
133.32
128.76
128.73
128.20
125.52
125.34
123.85
122.19
120.09
116.99
77.55 CDCl3
77.23 CDCl3
76.91 CDCl3
52.66
45.42
34.21
32.11
24.34
23.15
O
O
OMe
Me
33
N
Ac
f1 (ppm)
